# Genomic epidemiology of SARS-CoV-2 in Esteio, Rio Grande do Sul, Brazil

**DOI:** 10.1186/s12864-021-07708-w

**Published:** 2021-05-20

**Authors:** Vincius Bonetti Franceschi, Gabriel Dickin Caldana, Amanda de Menezes Mayer, Gabriela Bettella Cybis, Carla Andretta Moreira Neves, Patrcia Aline Grhs Ferrareze, Meriane Demoliner, Paula Rodrigues de Almeida, Juliana Schons Gularte, Alana Witt Hansen, Matheus Nunes Weber, Juliane Deise Fleck, Ricardo Ariel Zimerman, Lvia Kmetzsch, Fernando Rosado Spilki, Claudia Elizabeth Thompson

**Affiliations:** 1 Center of Biotechnology, Graduate Program in Cell and Molecular Biology (PPGBCM), Universidade Federal do Rio Grande do Sul (UFRGS), Porto Alegre, RS Brazil; 2 Graduate Program in Health Sciences, Universidade Federal de Cincias da Sade de Porto Alegre (UFCSPA), Porto Alegre, RS Brazil; 3 Department of Statistics, Universidade Federal do Rio Grande do Sul (UFRGS), Porto Alegre, RS Brazil; 4 Molecular Microbiology Laboratory, Universidade Feevale, Novo Hamburgo, RS Brazil; 5 Irmandade Santa Casa de Misericrdia de Porto Alegre, Porto Alegre, RS Brazil; 6 Department of Pharmacosciences, Universidade Federal de Cincias da Sade de Porto Alegre (UFCSPA), 245/200C Sarmento Leite St, Porto Alegre, RS 90050-170 Brazil

**Keywords:** COVID-19, Severe acute respiratory syndrome coronavirus 2, Infectious diseases, Sequencing, Molecular epidemiology

## Abstract

**Background:**

Brazil is the third country most affected by Coronavirus disease-2019 (COVID-19), but viral evolution in municipality resolution is still poorly understood in Brazil and it is crucial to understand the epidemiology of viral spread. We aimed to track molecular evolution and spread of Severe acute respiratory syndrome coronavirus 2 (SARS-CoV-2) in Esteio (Southern Brazil) using phylogenetics and phylodynamics inferences from 21 new genomes in global and regional context. Importantly, the case fatality rate (CFR) in Esteio (3.26%) is slightly higher compared to the Rio Grande do Sul (RS) state (2.56%) and the entire Brazil (2.74%).

**Results:**

We provided a comprehensive view of mutations from a representative sampling from May to October 2020, highlighting two frequent mutations in spike glycoprotein (D614G and V1176F), an emergent mutation (E484K) in spike Receptor Binding Domain (RBD) characteristic of the B.1.351 and P.1 lineages, and the adjacent replacement of 2 amino acids in Nucleocapsid phosphoprotein (R203K and G204R). E484K was found in two genomes from mid-October, which is the earliest description of this mutation in Southern Brazil. Lineages containing this substitution must be subject of intense surveillance due to its association with immune evasion. We also found two epidemiologically-related clusters, including one from patients of the same neighborhood. Phylogenetics and phylodynamics analysis demonstrates multiple introductions of the Brazilian most prevalent lineages (B.1.1.33 and B.1.1.248) and the establishment of Brazilian lineages ignited from the Southeast to other Brazilian regions.

**Conclusions:**

Our data show the value of correlating clinical, epidemiological and genomic information for the understanding of viral evolution and its spatial distribution over time. This is of paramount importance to better inform policy making strategies to fight COVID-19.

**Supplementary Information:**

The online version contains supplementary material available at 10.1186/s12864-021-07708-w.

## Background

In December 2019, the causative agent of Coronavirus disease-2019 (COVID-19) pandemic named Severe acute respiratory syndrome coronavirus 2 (SARS-CoV-2), emerged in Wuhan, Hubei, China [1]. As of 28 April, 2021, there are 148.963.836 confirmed cases and 3.140.213 million deaths in 192 countries [2]. Unprecedented international efforts of viral sequencing have allowed the submission of ~1.3 million genomes in the Global Initiative on Sharing All Influenza Data (GISAID) up to date [3], which are now available for studies of genomic epidemiology to follow the evolutionary history and dynamics of SARS-CoV-2 through space and time. In this sense, some important studies were already conducted in highly-affected countries, including USA [4,7], Italy [8], Netherlands [9], Australia [10, 11], and Brazil [12,14].

By using a nomenclature developed to capture local and global patterns of genetic diversity of the virus, two main lineages (A and B) were identified, both originated in Wuhan and with simultaneous spreading around the world [15]. The dynamics of viral transmission in the Brazilian territory was investigated through the sequencing of ~500 genomes until the end of April, 2020. It was determined that: (i) B.1 and derived lineages were predominant at the beginning of the pandemic; (ii) >100 independent international introductions occurred in the country; (iii) a significant movement of the virus among the Brazilian regions was observed after international travel restrictions; and (iv) non-pharmacological measures were able to reduce the reproduction number (R_0_) from >4 to 1 [12].

The genetic diversity of SARS-CoV-2 has been extensively studied, evidencing the presence of recurrent mutations such as S: D614G, S:E484K, S:N501Y across the world [16,18], related to increased pathogenicity and transmissibility (higher viral loads, increased replication on lung epithelial cells, and enhanced binding affinity) [19,24]. Furthermore, the E484K mutation was associated with immune evasion from neutralizing antibodies produced in response to currently available vaccines [25,27]. In addition to SARS-CoV-2 mutations, co-infection with other pathogens (e. g. *Staphylococcus aureus*, *Haemophilus influenzae*, rhinovirus/enterovirus, respiratory syncytial virus, and seasonal coronaviruses) may be associated with poor clinical outcomes, although these rates appear to be limited [28,31].

The viral mutations are not the only factor affecting the COVID-19 pathology and SARS-CoV-2 infectious capacity. Human host factors such as: (i) rare genetic variants governing interferon immunity [32], (ii) DNA polymorphisms in key host factors (e. g. Angiotensin-converting enzyme 2 [ACE2] and transmembrane protease serine 2 [TMPRSS2]) [33, 34], (iii) heritage and ethnicity [35], (iv) the presence of comorbidities (hypertension, diabetes, obesity, and immunological diseases) [36, 37] were already associated to increased disease severity, although more integrative studies are still needed to identify the relative contribution of each of these factors. By analyzing 27 candidate genes and Human leukocyte antigen (HLA) alleles in 954 admixed Brazilian exomes, 395 nonsynonymous variants were found. Of these, six were previously associated with the rate of infection or clinical prognosis of COVID-19. Seventy were identified exclusively in the Brazilian sample, and seven (10%) of these were predicted to affect protein function using in silico analysis [38].

As of March 10, 2020, a 60-year-old man who had been in Italy, became the first confirmed case in the southernmost state of Brazil (Rio Grande do Sul - RS) [39], which is the most populous state in the South Region of Brazil and the fifth in the whole country (~11.5 million inhabitants) [40]. As of April 28, 2021, Brazil has ~9.7% of worldwide cases (~14.4 million) and is the third worst-hit country [2]. The RS State reported ~956,030 cases and 24,458 deaths, with ~8% of cases requiring hospitalization [39]. The municipality of Esteio, located in the metropolitan region of RS capital, reported 9272 cases (total population: 83,202) and 302 deaths [41]. As Esteio is a commuter town, many workers move to and return from the state capital every day. Importantly, the case fatality rate (CFR) in Esteio (3.26%) was slightly higher compared to the RS state (2.56%), and both are greater than previous CFR estimates (~1%) [42, 43].

Thus, we aimed to characterize the main circulating lineages in Esteio (RS, Brazil) and their relationship with global, national and regional lineages using phylogenetics and phylodynamics inference from 21 SARS-CoV-2 genome sequences, including the investigation of putative viral mutations related to poor outcomes. Additionally, due to our typical subtropical climate and therefore high occurrence of respiratory infections, we investigated the occurrence of co-infections with other viral pathogens in these samples. The choice of a small municipality as the target of this study was important since we could more easily and precisely follow infected individuals, allowing a more detailed surveillance on the spread of the virus and detection of variability.

## Results

SARS-CoV-2 genomes were obtained with an average coverage depth of 1380.51 (median: 213.28, standard deviation: 2296.16) (AdditionalFile1). All consensus genomes passed the quality control steps. Considering the 21 samples (Table1), 52.4% of the patients were female and the mean age was 41.3years (range: 1972years). The mean Cycle threshold (Ct) values was 16.12 (range: 12.5319.94). None of the patients reported interstate or international travels. Regarding clinical status, 90.5% of patients were classified as mild infection and 9.5% as moderate.

**Table 1:** Epidemiological data of the 21 sequenced samples from Esteio, RS, Brazil

GISAID Accession	Ct value	Collection month	Age range	Sex	Clinical status	Pangolin Lineage	Nextstrain Clade
EPI_ISL_831678	16.15	May 2020	2030	M	Mild	B.1.1.33	20B
EPI_ISL_831474	16.48	June 2020	2030	M	Mild	B.1.1.33	20B
EPI_ISL_831645	16.72	June 2020	60+	M	Moderate	B.1.1.248	20B
EPI_ISL_831646	16.45	June 2020	5060	F	Mild	B.1.1.33	20B
EPI_ISL_831660	15.53	June 2020	2030	F	Mild	B.1.1.248	20B
EPI_ISL_831681	16.72	July 2020	4050	F	Mild	B.1.1	19A
EPI_ISL_831683	15.50	July 2020	3040	F	Moderate	B.1.1.33	20B
EPI_ISL_831685	16.65	July 2020	1020	F	Mild	B.1.1.33	20B
EPI_ISL_831688	15.52	July 2020	4050	M	Mild	B.1.1.248	20B
EPI_ISL_831689	14.37	August 2020	3040	M	Mild	B.1.1.248	20B
EPI_ISL_831892	15.12	August 2020	60+	M	Mild	B.1.1.33	20B
EPI_ISL_831898	14.14	August 2020	4050	M	Mild	B.1.1.33	20B
EPI_ISL_831913	12.53	August 2020	3040	F	Mild	B.1.1.49	20B
EPI_ISL_831938	15.32	August 2020	5060	M	Mild	B.1.1.248	20B
EPI_ISL_831939	15.58	September 2020	4050	M	Mild	B.1.1	20B
EPI_ISL_831940	17.10	September 2020	2030	F	Mild	B.1.1.33	20B
EPI_ISL_832009	14.99	September 2020	3040	F	Mild	B.1.1.248	20B
EPI_ISL_832010	18.31	October 2020	2030	F	Mild	B.1.1.248	20B
EPI_ISL_832011	17.33	October 2020	40	M	Mild	B.1.1.248	20B
EPI_ISL_832012	19.94	October 2020	60+	F	Mild	B.1.1.33	20B
EPI_ISL_832013	18.15	October 2020	4050	F	Mild	B.1.1	20B

All samples were nasopharyngeal swabs collected from patients of the municipality of Esteio. *Sample ID* Sample identifier; *M* Male; *F* Female

### Virome analysis

To investigate whether the severity of the infection presented by the patients could be linked to co-infection with another respiratory viral pathogen, we analyzed the viral composition of these samples. We found through taxonomic classification at the level of nucleotides and amino acids that none of the investigated patients had a viral infection other than COVID-19. All samples had high assignment (>99%) to the *Betacoronavirus* genus.

### SARS-CoV-2 mutations found in the patient samples and lineages

**Fig. 1:**
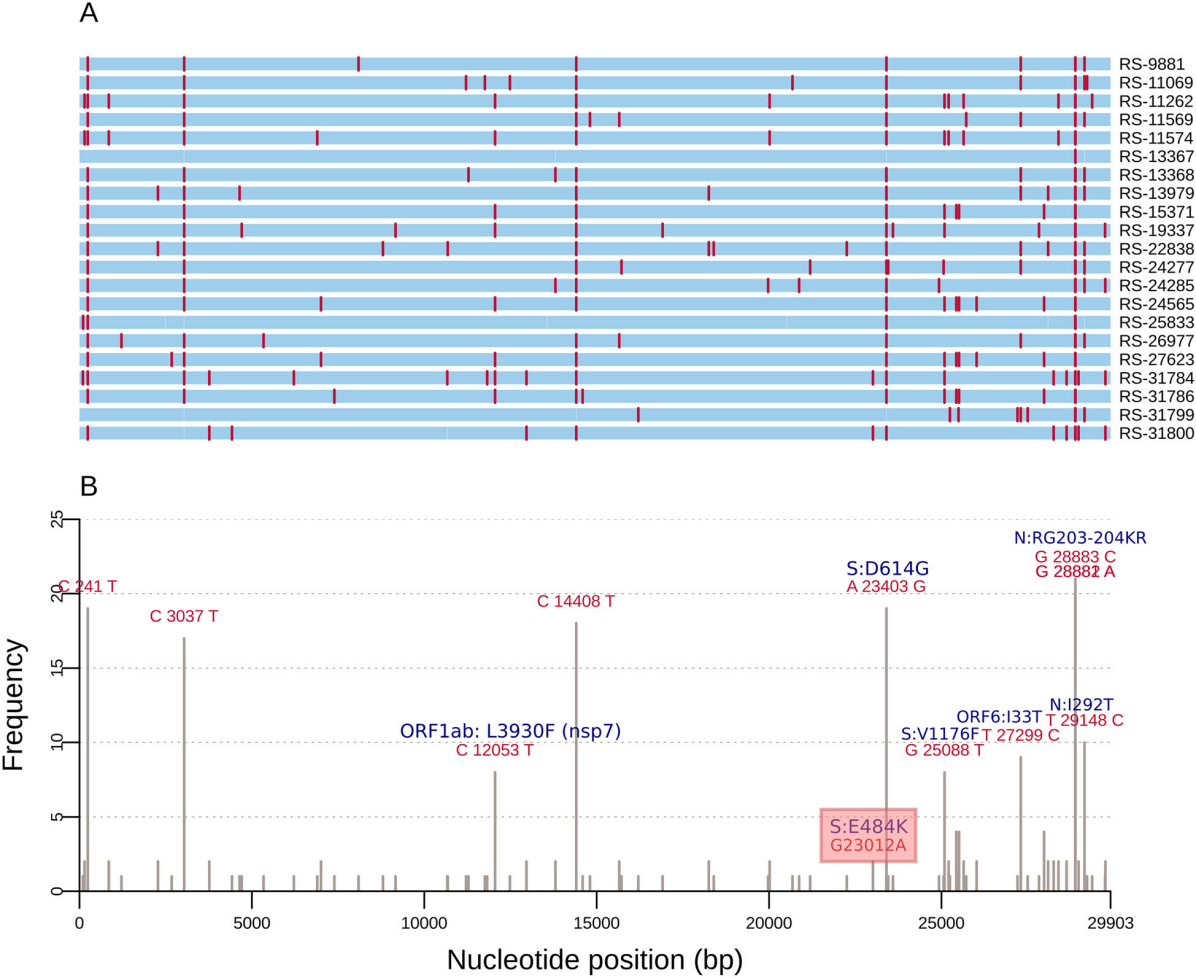
Mutations of SARS-CoV-2 genomes from patients of Esteio, RS, Brazil. **a** Genome map for the 21 genomes sequenced (indicated in the right). SNPs are colored in red. **b** Frequency of SNPs per SARS-CoV-2 genome position among the 21 genome sequences. These mutations correspond to the red lines in (**a**) and only those represented by >5 genomes are indicated above the bars, regardless of being synonymous or missense. The abbreviations of genes affected and respective amino acid changes (where applicable) are indicated above the nucleotide changes

The number of SNPs per genome ranged from 1 to 19 (mean: 12.8, median: 14.0) (Fig.1a). All genomes were different from each other. We identified 80 different SNPs in the 21 genomes analyzed. Thirty two (40.0%) of them were observed in more than one sample (Supplementary Table1). Of these, 18 (56.2%) were missense (non-synonymous). High frequency (>5 genomes) missense mutations were observed in the following positions (absolute nucleotide position: amino acid inside the gene): ORF1ab (C12053T: L3930F), Surface (S) glycoprotein (A23403G: D614G; G25088T: V1176F), ORF6 (T27299C: I33T), and Nucleocapsid (N) protein (GGG28881-28883ACC: RG203-204KR; T29148C: I292T) (Fig. 1b). A new mutation in the Receptor Binding Domain (RBD) of the spike protein (G23012A: E484) was found in two genomes (9.5%) (GISAID IDs: EPI_ISL_832010 and EPI_ISL_832013) from mid-October 2020. Since the municipality of Esteio has a higher CFR (3.26) than the national CFR (as the Brazilian states of So Paulo, Amazonas, Pernambuco, and Rio de Janeiro that were highly affected by the pandemic) (Supplementary Table2), it is possible that the emergence of new viral mutations and lineages combined with genetic factors in these populations [38, 44] are partially associated with differential COVID-19 severity.

We were able to identify four different viral lineages, all descendants of lineage B (Table 1). Two lineages associated with community-transmission in Brazil, B.1.1.33 (*n*=9; 42.9%) and B.1.1.248 (reassigned later to B.1.1.28) (*n*=8; 38.1%) were the most prevalent. All B.1.1.33 sequences shared T27299C (ORF6:I33T), GGG28881-28883AAC (N:RG203-204KR), and T29148C (N:I292T) mutations. All B.1.1.248 sequences shared C241T (5 UTR), C3037T (ORF1ab nsp3:F924), C12053T (ORF1ab nsp7:L3930F), C14408T (ORF1ab RdRp:L4715), A23403G (S:D614G), G25088T (S:V1176F), and GGG28881-28883AAC (N:RG203-204KR) replacements.

**Fig. 2:**
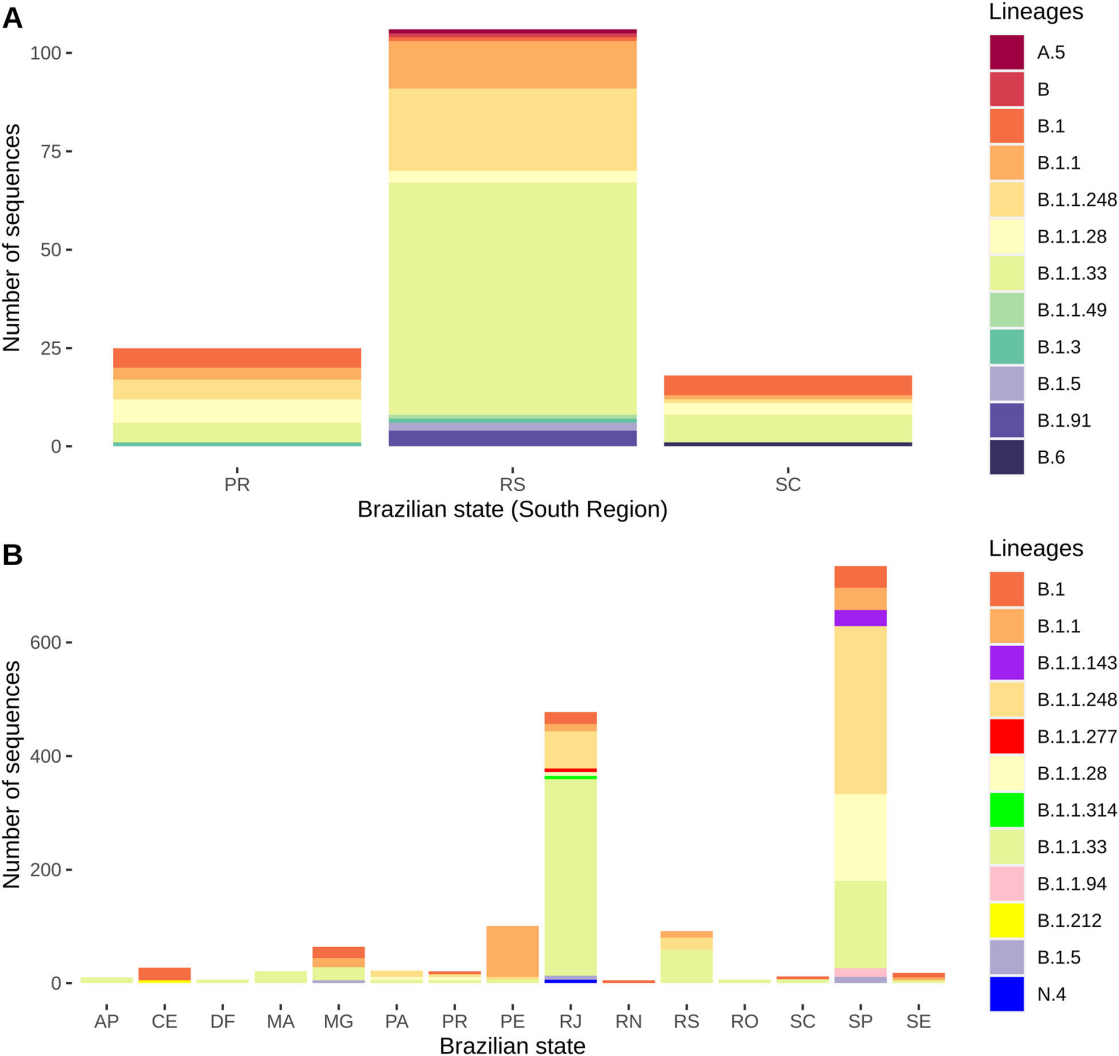
Most prevalent lineages in Brazil considering all 1778 Brazilian sequences available in the GISAID database. **a** All sampled viral lineages from Southern Brazil, including Paran (PR), Rio Grande do Sul (RS) and Santa Catarina (SC) states. The RS sequences include the 21 viral genomes from this work and 85 from GISAID. **b** Most prevalent lineages from Brazilian states. Only states that have more 5 sequences labelled as the lineage under consideration were included. AP=Amap; CE=Cear; DF=Distrito Federal; MA=Maranho; MG=Minas Gerais; PA=Par; PE=Pernambuco; RJ=Rio de Janeiro; RN=Rio Grande do Norte; RO=Rondnia; SP=So Paulo; SE=Sergipe

Both lineages are represented by >70% of Brazilian sequences in global context (https://cov-lineages.org/). They are the most representative lineages in the South Region of Brazil and in the whole country (Fig.2, Supplementary Figures 1, 2 and 3). Three genomes were classified as B.1.1 lineage, which are the most globally widespread lineages characterized by RG203-204KR mutations in the nucleocapsid phosphoprotein (https://cov-lineages.org [15];). Finally, a relatively rare lineage (B.1.1.49) mostly found in Wales and Denmark was also assigned.

After inspecting these sequences assigned to global lineages (B.1.1 and B.1.1.49), we verified that in all cases there were characteristic mutations of B.1.1.248 and B.1.1.33 lineages flagged as undetermined bases (N character; depth of coverage (DP)<10) in the consensus genome. After reclassifying these sequences using low coverage variants, two were attributed to B.1.1 lineage, one to B.1.1.248 and one to B.1.1.33 (AdditionalFile2). Therefore, due to the absence of confirmed relationships with other countries and the presence of other defining-lineage variants with low coverage, it seems probable that these sequences are also the result of community transmission in Brazil and were not introduced independently in the municipality of Esteio from other countries.

We also detected two novel epidemiologically-related clusters until then unknown. Two patients had three unique mutations in genome positions 25,207 (in the S2 subunit of spike), 25,642 (ORF3a), and 28,393 (Nucleocapsid), all synonymous substitutions. The patients both live in the same neighborhood, about 100m from each other and did the test in a 2-day interval. We also identified another cluster of four patients characterized by three unique mutations: 25429 (ORF3a: V13L), 25,509 (ORF3a), and 27,976 (ORF8: H28R). The tests of these patients were performed in a 3-month interval (July 15October 13), suggesting a fixation of these mutations through time, possibly forming a new sublineage. The two clusters are linked to viruses belonging to B.1.1.248 lineage, suggesting the existence of specific mutation signatures even within lineages.

### Phylogenetics analysis

After running the Nextstrain pipeline for quality control and subsampling, we obtained 3758 time-, geographical- and genetic-representative genomes to proceed phylogenetic inferences. Of these, 393 were from Africa, 800 were from Asia, 1203 from Europe, 235 from North America, 127 from Oceania, and 1000 from South America. Considering the latter, 609 were from Brazil and 98 from Rio Grande do Sul (21 from this study plus 77 from GISAID that passed the quality control criteria) (AdditionalFile3).

**Fig. 3:**
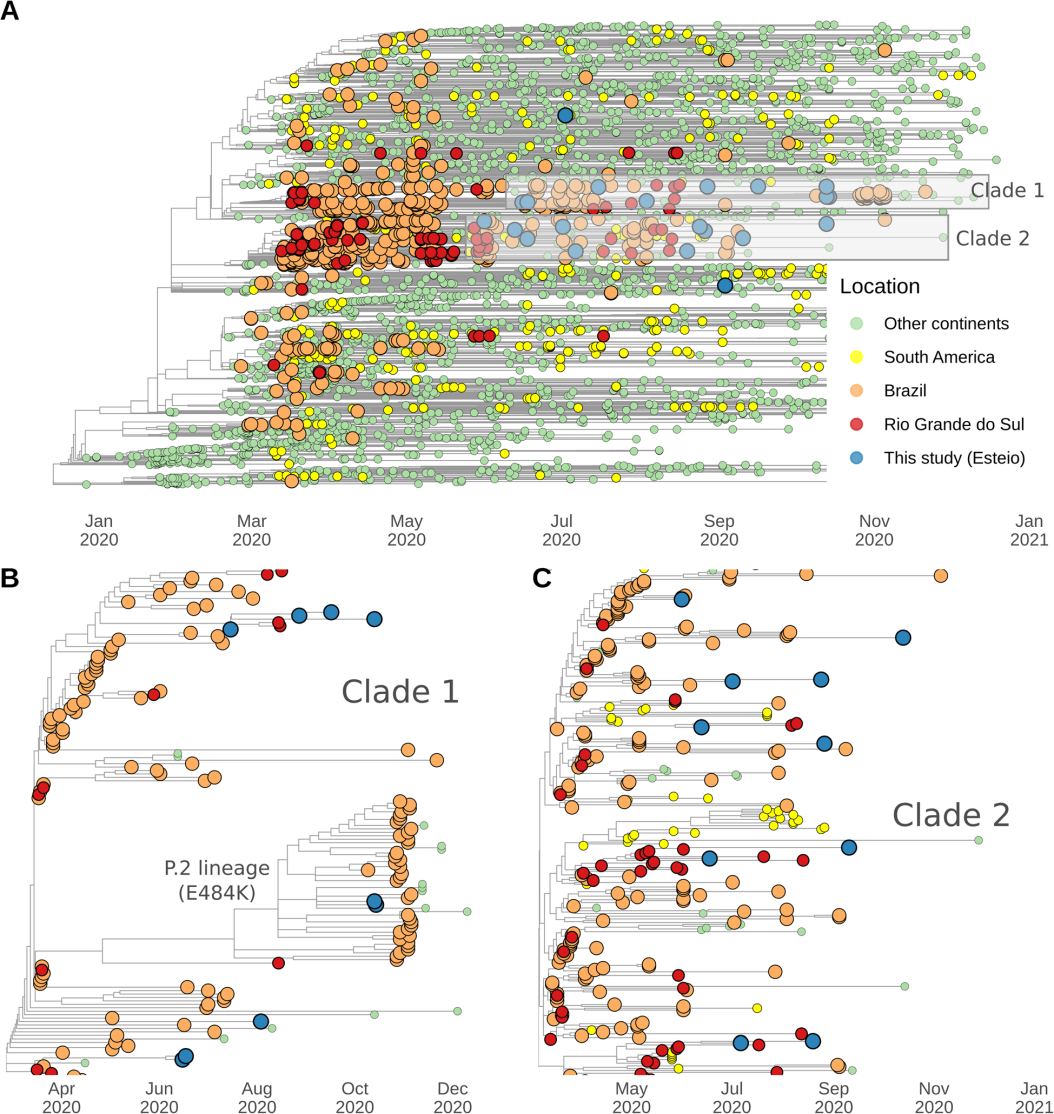
Time-resolved phylogeny inferred from our 21 sequences combined with other 3758 worldwide genomes sampled between December 2019 and December 2020. Other sequences from the RS state (*n*=77) were obtained from the GISAID database. **a** Worldwide distribution of sequences in the subsampled tree, highlighting genomes from this study. **b** Zoom-in on sequences from this study belonging to Clade 1 / lineage B.1.1.248 (upper clade in **a**). **c** Zoom-in on sequences from this study belonging to Clade 2 / lineage B.1.1.33 (bottom clade in **a**). In (**b**) and (**c**), the clade is initiated from the T_MRCA_ of the sequences presented in this study and it does not refer to all the sequences assigned to specific lineages

The time-resolved ML phylogeny confirmed that the majority of the time-representative sequences from Esteio are the result of community transmission within Brazil. The sequences grouped mostly in two clades (Fig.3a, Supplementary Figure 4) corresponding to lineages B.1.1.248 and B.1.1.33. Clade 1 comprised 147 sequences: 20 from RS, 58 from SP, 46 from RJ, 2 from PR, and 2 from SC, therefore mostly widespread in Brazilian states (Fig. 3b). Clade 2 included 277 genomes: 50 from RS, 72 from RJ, 20 from SP, 5 from SC, 30 from Chile, 10 from Argentina, and 6 from Uruguay (Fig. 3c). These results suggest a clade distributed through South American countries. Esteio sequences are relatively evenly distributed through these clades mostly represented by Brazilian and RS genomes. Exceptions to this observation were the two previously described epidemiologically-linked clusters, whose sequences grouped together in the B.1.1.248 lineage as expected (Fig. 3b). Given the low mutation rate of SARS-CoV-2 (6.5910^4^ substitutions/site/year, ~19 mutations per year) (Supplementary Figure 5), we believe that this would indicate at least three introductions of lineage B.1.1.248 and six introductions of lineage B.1.1.33 in the municipality of Esteio, probably from other locations in Brazil, and a national movement of the virus even to more distant places like the southernmost state of Brazil. Likewise, despite the large representativeness of Brazilian samples within these two major clades, we also found other sequences from Asia, Europe, Oceania, and South America. Therefore, sequences from these clades seem to have been directly transmitted from Brazil to other countries.

### Phylodynamics and phylogeographic analysis

The estimate for population exponential growth rate for B.1.1.248 was 1.141 (95% Highest Posterior Density (HPD) interval: 0.44361.8268), while for B.1.1.33 it was 2.5871 (95% HPD: 2.0623.0872). This can be taken as preliminary evidence that the B.1.1.33 lineage initially spread faster than B.1.1.248, since most of the coalescent events inform earlier periods of the pandemic (FebruaryMay, 2020). However, for better population dynamic inference, further analysis with more appropriate prior models for population dynamics would be required.

The Bayesian model estimates for the substitution rate are 7.2810^4^ subst/site/year (95% HPD: 6.3210^4^ - 8.2510^4^) for B.1.1.248 and 6.1610^4^ subst/site/year (95% HPD: 5.6110^4^ - 6.7610^4^) for B.1.1.33. While both intervals overlap with the overall estimate of the time-resolved ML tree built from 3758 representative genomes (6.5910^4^ subst/site/year), the B.1.1.248 lineage seems to have higher mutation rates. However, the phylogeographic model estimates similar overall migration rates for both lineages, 0.1710 (95% HPD: 0.06510.3523) for B.1.1.33 and 0.1980 (95% HPD: 0.08120.5002) for B.1.1.248.

**Fig. 4:**
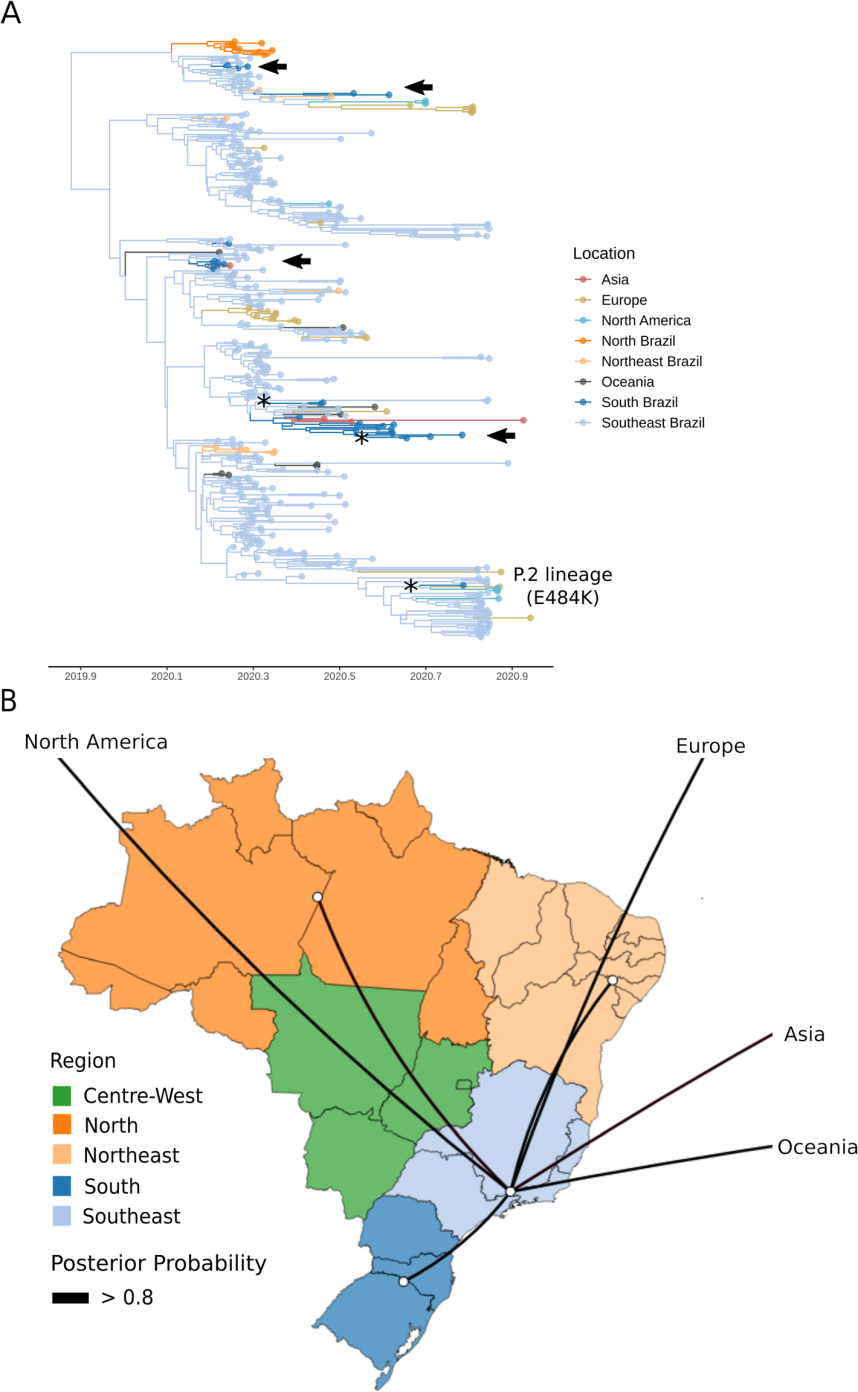
Spatiotemporal diffusion of B.1.1.248 lineage across Brazilian regions and other continents. **a** Time-resolved MCC tree of 405 genomes belonging to B.1.1.248 lineage. **b** Well-supported transition rates between Brazilian regions and other continents in discrete phylogeographic reconstructions using BSSVS procedure and Bayes Factor tests. Transition rates with posterior probabilities <0.5 were cut-off. The following states belong to each Brazilian region: Centre-West: DF, GO, MS, MT. North: AC, AM, AP, PA, RO, RR, TO. Northeast: AL, BA, CE, MA, PB, PE, PI, RN, SE. South: PR, RS, SC. Southeast: ES, MG, RJ, SP. The RS state is located in the Southern region. Subclades represented by >1 Southern Brazilian sequence are indicated by arrows and introductions in Esteio are represented by asterisks. The introduction of P.2 lineage (E484K mutation) is indicated

**Fig. 5:**
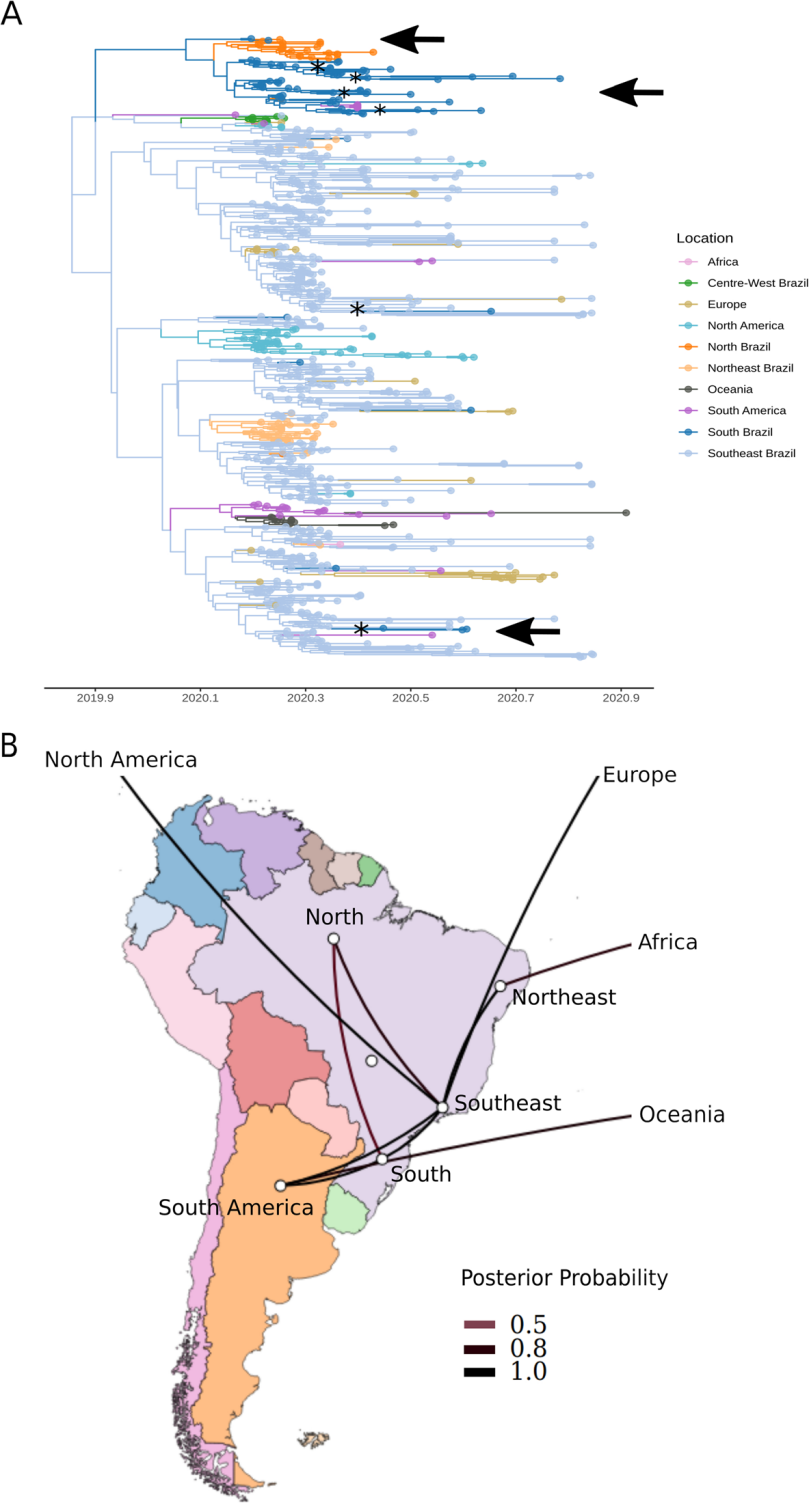
Spatiotemporal diffusion of B.1.1.33 lineage across Brazilian regions, South America and other continents. **a** Time-resolved MCC tree of 725 genomes belonging to B.1.1.33 lineage. **b** Well-supported transition rates between Brazilian regions and other continents in discrete phylogeographic reconstructions using BSSVS procedure and Bayes Factor tests. Transition rates with posterior probabilities <0.5 were cut-off. The subclades represented by >1 Southern Brazilian sequence are indicated by arrows and introductions in Esteio are represented by asterisks

Time-measured phylogeographic analysis highlighted the major contribution of Southeast in Brazilian and worldwide diffusion of both lineages (Figs. 4 and 5). Southeast is a common source of B.1.1.248 migrations, since we identified transition events between this region and Northern, Northeast, and Southern Brazil, as well as Asia, Europe, North America and Oceania (Bayes Factor (BF)>30; Posterior Probability (PP)>0.8) (Fig. 4a and b). The four subclades from Southern Brazil in the B.1.1.248 Maximum-Clade Credibility (MCC) tree were probably introduced from Southeast (Fig. 4a and b), and we were able to confirm that at least three independent introductions occurred in the municipality of Esteio as suggested previously by the ML analysis (Fig. 4a). Most importantly, the introduction of the P.2 lineage that harbors the E484K mutation was dated on September 09, 2020 (95% HPD: September 09October 05, 2020) probably introduced from the Rio de Janeiro state. Interestingly, sequences from the USA and England formed a monophyletic clade with our sequence, demonstrating the spread from Brazil to other countries (Fig. 4a).

Southeast also seems to be determinant for the viral diffusion of B.1.1.33 lineage. Of note, the tree reconstruction showed important migrations from Southeast to Northern, Northeast, and Southern Brazil, as well as Europe, North and South America (BF>30; PP>0.8) (Fig. 5a and b). Well-supported rates were also identified between Northeast and Africa (BF=31.10; PP=0.79) and between South America and Oceania (BF=56.99; PP=0.87). Viruses belonging to this lineage appear to have a major contribution in Southern Brazil epidemics, since its sequences formed a monophyletic clade with >50 sequences in the B.1.1.33 MCC tree (Fig. 5a). Furthermore, this analysis confirmed that Southern Brazil is a probable source of importations of available B.1.1.33 sequences to South-American countries (BF=1467.97; PP=0.99) and Northern Brazil (BF=9.45; PP=0.53) (Fig. 5a and b). Of note, we also validated that at least 6 introductions should have happened in the municipality of Esteio, two from the Southeast and four from other municipalities of Southern Brazil (especially from the RS state).

## Discussion

In the present work, we accessed SARS-CoV-2 mutations, circulating lineages and phylogenetic patterns of SARS-CoV-2 from a time- and age-representative set of patients admitted in a municipal healthcare system from the Southern region of Brazil, the third country most affected by the Covid-19 pandemic. As the study was conducted in a small municipality, we were able to track two clusters of viral mutations in epidemiologically-linked patients, highlighting the importance of viral dissemination in small areas of the community.

The SARS-CoV-2 spike (S) glycoprotein mediates the interaction with the ACE2 receptor in the host cells and it is the primary target of neutralizing antibodies [45]. There are structural unique spike features that contribute to its pandemic capacity: (i) a flat sialic acid-binding domain enables faster viral surfing over the epithelial surface before receptor interaction; (ii) tight and nearly perfect binding to the ACE2 entry receptor; (iii) the capacity to use furin and other proteases for cell entry [46]. A mutation in S (D614G) was recently associated with higher viral loads [19], increased replication on human lung epithelial cells [23], and younger age of patients [22], but not with the disease severity [19, 22]. This mutation is associated with an abolition in the hydrogen bond between the aspartate originally located at 614 position and a threonine residue in the 859 neighbouring protomer of the spike trimer, thus increasing the probability of RBD being found in the open state [47]. This promotes the binding with the ACE2 receptor leading to enhanced infectivity [23]. Importantly, this replacement was detected in 90.5% of our samples, compatible with the dominance of this variant in global context until December 14, 2020 (221,700 sequences [48];). Also concerning the S protein, we identified the V1176F variant in 38.1% of our samples and in all of them there was a co-occurrence with D614G. Importantly, 349/535 (65.2%) of the sequences isolated in the world including this replacement were from Brazil [48]. More recently (April 28, 2021), 1167 (53.7%) of worldwide sequences were from Brazil, representing mostly the spread of B.1.1.28 and P.1 lineages that harbor this mutation. This substitution is located on the C-terminal portion (S2 subunit), more specifically on the heptad repeat 2 (HP2), which is a major target of adaptive evolution in MERS-CoV-related viruses and carry sites associated with expanded host range in other coronaviruses [49].

Another replacement in the RDB of S protein (E484K) was also assigned. Until mid-December 2020, 157 genomes have this mutation globally, 114 (72.6%) isolated from South Africa, where a new lineage (B.1.351 or 501Y.V2) characterized by three RBD mutations (K417N, E484K and N501Y) recently emerged [50]. E484K also emerged independently in multiple lineages, including P.1, P.2 and B.1.1.33 firstly identified in Brazil between late 2020 and early 2021 [51]. As of 28 April, 2021, 38,436 genome sequences harbor this mutation, which are found in ~10% of the sequences generated to date on average (https://outbreak.info/situation-reports?muts=S%3AE484K) In Brazil, >90% of the sequenced samples since February 2021 carry this mutation, which is present in P.1 and P.2 emergent lineages (https://outbreak.info/situation-reports?muts=S%3AE484K&selected=BRA&loc=BRA). Recent evidence showed that E484K replacement enables viral escape from neutralizing monoclonal antibodies or polyclonal sera [25,27] facilitating reinfection by emerging lineages harboring this mutation as reported in Brazil [52]. Importantly, two sequences (9.5%) from this study had four of five shared mutations with a new lineage (subsequently called P.2) reported in the Rio de Janeiro state (synonymous C28253T (F120F; ORF8), missense G28628T (A119S) and G28975T (M234I) in N protein and C29754U (3 UTR)), in addition to E484K RBD replacement [53]. Our sequences from Esteio were sampled in mid-October, as the first cases of the RJ lineage (Additional File 2). Phylodynamics inferences pointed that this lineage emerged in early July, approximately four months before the detection of its first genomes [53]. Moreover, its identification in a small municipality from the RS state (located 1.5 thousand kilometers from RJ) demonstrates that it emerged months before October and is already widely distributed in the Brazilian territory, but went unnoticed so far by the lack of appropriate genomic surveillance in Brazil.

The substitution of a negatively charged amino acid (Glutamic Acid) for the positively charged Lysine has a profound impact upon a highly flexible loop at the RBD. More specifically, it creates a strong ion interaction between lysine and amino acid 75 of hACE-2 (the main SARS-CoV-2 receptor). This link is not present in the wild type E484. Shifting key elements of RBD responsible for interactions may have a major impact in the risk of immune evasion. Also E484K could be related to enhanced infectivity, which may be associated with the rapid dissemination of these escape mutants [54]. The publication of a recent reinfection case of SARS-CoV-2 harboring E484K and the presence of this mutation in COVID-19 patients during the current second wave in Northern Brazil [55] are highly suggestive that this mutation is critical for viral evolution and thus must be investigated thoroughly [52].

The ORF6 accessory protein plays a critical role in antagonizing host antiviral responses and viral replication. Therefore, it potentially inhibits both type I interferon (IFN) production and downstream signaling [56, 57]. We have found the ORF6:I33T mutation in 42.9% of our samples, raising its potential association with immune suppression. The N protein packages the genomic RNA, playing a fundamental role during viral self-assembly [58]. It is also associated with replication-transcription complexes [59], and used as a target for diagnostic and immunogenic applications. Interestingly, a tri-nucleotide mutation in the Nucleocapsid gene (RG203KR) resulting in a double amino acid change was observed in high frequency in this study (~100), and are characteristic of B.1.1 and derived lineages that spread rapidly around the world [48]. The missense mutation in ORF1ab (L3930F) was reported in other 429 sequences (125 from Brazil) and also in two sequences of lineage B.1.1.248 from the Philippines [60]. Interestingly, the I33F and I292T mutations that were found in ORF6 and N, respectively, have been considered dominant mutations in the SARS-CoV-2 sequences from Brazil [61, 62]. The co-occurrence of these mutations represents the signature of Clade 2 (subsequently named B.1.1.33 lineage), which were one of the three most prevalent Brazilian viral lineage groups in the beginning of the pandemic, highly widespread in 16 states from Brazil [12]. It is important to emphasize that functional studies are necessary to characterize the effect of each viral mutation on transmissibility and pathogenicity. It is expected that most mutations will not have a great impact on viral evolution and its relationship with the human host. However, some of them may increase the viral fitness or represent some viral advantage in the pathogen-host interaction and, consequently, may become fixed in the population. Further studies to elucidate the interaction between human gene variants (especially in the ACE2 receptor) and SARS-CoV-2 mutations are necessary to establish the possible impacts of spike amino acid replacements with regard to ACE2 binding and function [63].

The knowledge of ACE2 physiological functions and specific features could explain how comorbidities like hypertension, diabetes, obesity, and immunological diseases can enhance the severity of symptoms. Thus, modulation of ACE2s function might promote pulmonary inflammation, thrombosis, obesity-induced hypertension, and cardiac failure, which are especially unfavorable to COVID-19 patients [36]. The expression of other cell receptors potentially involved with COVID-19 infection depending on age, gender, and characteristics such as obesity, smoking, and polymorphisms can contribute to patterns of severe symptoms [64]. Moreover, the hypertension-associated elevated immunological activity is a noteworthy factor that promotes the increased risk of hypertensive patients for critical COVID-19 outcomes. The increased immune activation can be observed in these patients and might elucidate the hyperinflammatory response (cytokine storm) [37].

The Brazilian population was mainly formed through an admixture process that comprises mostly European, sub-Saharan African, and Native-American ancestry [65,67]. The broad spectrum of symptoms, days to symptoms onset, and the unpredictability of outcome in COVID-19 patients can also be linked with the vastly admixed Brazilian inhabitants. Moreover, recent reports identified HLA alleles previously associated with SARS-CoV-2 counteraction [38], and showed positive selection in genes associated with obesity, type II diabetes, lipid levels, and waist circumference [44]. Regarding COVID-19, epigenetics, specific variants, ACE2 and TMPRSS2 polymorphisms, ethnicity, as well as inborn immunity errors, have been reported worldwide. This suggests that different host genetic backgrounds might contribute to discrepancies in SARS-CoV-2 aggressiveness [32,35].

We observed a higher CFR in the municipality of Esteio when compared to the RS state and the majority of Brazilian states, which might be linked to the emergence of new viral variants. However, COVID-19-related mortality is determined by both intrinsic factors of the infected individuals (age, comorbidities, and genetic characteristics) [68] and extrinsic aspects such as the access to healthcare assistance (hospital beds, mechanical ventilators, medicines). Additionally, the Southern Brazilian states (Rio Grande do Sul, Santa Catarina, and Paran) have important determinants of mortality: older population than other regions and highest historical incidence of SARS in the country [69]. Their proximity to the states of So Paulo and Rio de Janeiro (that account for ~30% of the national population) also facilitates travel between the two regions and the rapid dissemination of emerging lineages.

Importantly, CFR is highly influenced by the underreporting of confirmed cases and deaths. States with low testing capacity tend to generate higher CFRs, and recently many deaths with an undetermined cause have been reported in Brazil, which also affects the quality of the records [70]. Therefore, the analysis of lethality should take this combination of factors into account [71]. In the case of Esteio, it is the municipality that tested more proportionally to the number of cases (38,416 tests per 100,000 inhabitants), thus these underreporting biases should be less significant, in contrast to the observed in other Brazilian states.

Compared to three other studies conducted in Brazil [12], in the states of Minas Gerais [14] and Pernambuco [13] in the early phase of pandemic which showed the introduction of viral lineages from other continents (mainly Europe) by international returning travelers , this study suggests a minor role of international lineages in the ongoing viral transmission in Esteio. We speculate a trend towards the perpetuation and diversification of the lineages found in this study (B.1.1.248 and B.1.1.33) inside Brazil. The dissemination of these lineages were also reported in the Uruguayan-Brazilian border, driving viral introductions mainly from Southeast and Southern Brazil (especially RS state) to Uruguay [72]. In this study, we found consistent results, mainly regarding B.1.1.33 diffusion from Southern and Southeast to South-American countries (*e. g. Argentina*, Chile and Uruguay). These lineages have already formed new sublineages (https://cov-lineages.org/lineages.html). B.1.1.33 has evolved in 10 new sublineages (N.1 to N.10). Furthermore, B.1.1.248 has evolved in P.1 (lineage first identified in Manaus [55] associated with a constellation of spike mutations like B.1.1.7 [73] and B.1.351 [50]), P.2 (lineage firstly identified in RJ state and also found in this study), and P.3. Importantly, all these three sublineages harbor the E484K mutation, which arose independently in both of them and appear to be evolving under diversifying positive selection [50, 51].

We built a time-resolved phylogeny prioritizing sequences that are genetically and spatially closer, but maintaining a global representativity of viral spread. This allowed us to confidently identify that our sequences fell into two main clades, with a broad presence of Brazilian and local sequences. We also inferred the spatiotemporal diffusion of these main lineages in regional and global context, finding the key role of Southeast in disseminating these lineages across Brazilian states and other continents. We also found a broader clade represented by Southern Brazil sequences and its important contribution in disseminating B.1.1.33 to South American countries. Moreover, we found four broader clades in the B.1.1.248 MCC phylogeny, suggesting multiple introduction events from Southeast followed by community transmission.

Our evolutionary rate estimates for both the broader ML tree and lineage-specific MCC trees (6 to 710^4^ subst/site/year) were slightly smaller than previous findings (8 to 910^4^ subst/site/year) [74, 75]. These differences have contributed to the estimates of the T_MRCA_ for both B.1.1.248 and B.1.1.33, which were dated to late 2019, contrasting with the first description of these lineages [12]. Furthermore, other probable sources of these inconsistencies are: closely related samples having the same age (phylo-temporal clustering), among-lineage rate variation and non-random sampling [76]. Although it is possible that different branches of the phylogeny have different rates, when we used a model that allows different rates across the tree (uncorrelated lognormal relaxed clock), the T_MRCA_ estimates have remained unchanged.

An important caveat for the phylodynamic analysis is that samples are not equally distributed geographically or temporally. This is a consequence of episodic sampling efforts prompted by research resource availability, and does not necessarily resemble a representative uniform sample. Unequal temporal distribution implies that some of the conclusions are disproportionately influenced by events in heavily sampled periods (FebruaryMay). Additionally, a large proportion of the samples come from the Southeastern region of Brazil. While this is in fact a heavily hit region and an economic and travel hub for the country, other regions such as the North are underrepresented. Thus, the strong support for the Southeast as prime center for viral dispersion and location of the root of both clades might be somewhat inflated, and epidemiological links between other regions could be downplayed due to undersampling. However, a study from the beginning of pandemic (FebruaryMarch 2020) estimated that the main destinations of the international passengers arriving to Brazil were So Paulo (46.1%), Rio de Janeiro (21%) and Belo Horizonte (4.1%), three capitals from the Southeast and therefore routes for COVID-19 importation [77]. Moreover, during mid-February and mid-March, SARS-CoV-2 spread mostly locally and within-state borders. In contrast, during mid-March and mid-April there was an ignition of the epidemic from the Southeast region to other states [12], which is consistent with our findings.

Some limitations should be considered. Firstly, it was not possible to analyze a larger sample size. Moreover, the low quantity and spatial representativity of sequences from the RS state to contextualize our sequences limited the inference of events of introduction and movement of the virus with municipal and state resolution. Still in this respect, we have observed a dramatic drop in the sequencing efforts from Brazil after April 2020 [12], which made it difficult to measure the main circulating lineages in the country during our investigation period (MayOctober, 2020) and may introduce confounding factors.

Since the E484K mutation identified in this study has been associated with loss of neutralizing activity from convalescent plasma (immune evasion) and enhanced interaction with hACE-2, lineages containing this substitution must be the subject of intense surveillance. More specifically, it is critical that immune strategies such as convalescent plasma and vaccines be tested against these new variants. Attempts to demonstrate activity against S mutants should be a priority effort for all vaccine and monoclonal antibody makers. Second generation immune therapies might have to be directed at more conservative neutralizing binding sites (such as S2 fusion domain) or elicit strong cellular response in order to keep on long term protection. Finally, human genetic factors, patient heritage and health conditions should also be studied in an integrated way for a broader understanding of vaccine effectiveness in different populations.

## Conclusions

Our results provide a comprehensive view of SARS-CoV-2 mutations from a time- and age-representative sample from May to October 2020, highlighting two frequent mutations in spike glycoprotein (D614G and V1176F), an emergent mutation in spike RBD (E484K) characteristic of B.1.351 and P.1 lineages, and the adjacent replacement of 2 amino acids in Nucleocapsid phosphoprotein (R203K and G204R). In particular, to our best knowledge, we described the earliest SARS-CoV-2 sequences harboring E484K in Southern Brazil. A significant viral diversity was evidenced by the absence of identical isolates in our samples. Furthermore, we identified patterns of SARS-CoV-2 viral diversity inside Southern Brazil, demonstrating the major role of community transmission in viral spreading and the establishment of Brazilian lineages ignited from the Southeast to other Brazilian regions. Our data show the value of correlating clinical, epidemiological and genomic information for the understanding of viral evolution and its spatial distribution over time. This is of paramount importance to better inform policy making strategies to fight COVID-19.

## Methods

### Sample collection and clinical testing

Nasopharyngeal samples were obtained from patients of the Hospital So Camilo, Secretaria Municipal de Esteio and Vigilncia em Sade from Esteio, RS, Brazil. Nasopharyngeal swabs were collected and placed in Viral Transport Medium (VTM, Copan Universal Transport Medium). Samples were transported to the Molecular Microbiology Laboratory from Feevale University and tested on the same day for SARS-CoV-2 by reverse-transcriptase quantitative polymerase chain reaction (RTq-PCR). Remnant samples were stored at 80C. SARS-CoV-2 diagnosis was performed using Real Time Reverse-transcriptase Polymerase Chain Reaction (Charit RT-qPCR assays). The RTq-PCR assay used primers and probes recommended by the World Health Organization (WHO) targeting the Nucleocapsid (N1 and N2) genes [78].

We selected 21 samples with RT-qPCR positive results, collected from May 31 to October 13, 2020 from patients residing in the municipality of Esteio, RS, Brazil. We included patients who presented symptoms such as fever, cough, sore throat, dyspnea, anosmia, fatigue, diarrhea and/or vomiting. The clinical status classification was based on the COVID-19 Clinical management guide recommended by the WHO [79]. Additionally, samples were selected based on cycle threshold (Ct) values 20. Electronic medical records were reviewed to compile epidemiological metadata (e. g., date of collection, sex, age, symptoms, exposure history, and clinical status).

### RNA extraction, library preparation and sequencing

We submitted the RT-qPCR positive for SARS-CoV-2 swabs to genomic RNA extraction. This process was performed in the automated nucleic acid purification system KingFisher Duo Prime Purification System (ThermoFisher Scientific, Waltham, USA) along with the MagMax CORE Nucleic Acid Purification Kit (ThermoFisher Scientific, Waltham, USA).

The extracted and purified genomic RNA was transcribed to cDNA using Maxima H Minus Double-Stranded cDNA Synthesis Kit, catalog number K2561 (ThermoFisher Scientific, Waltham, USA) following the manufacturers instructions. Library preparation was achieved using Nextera Flex for Enrichment with RNA Probes (Illumina, San Diego, USA). Briefly, we performed tagmentation in a pre-programmed thermocycler incubation temperature, until holding at 10C. This step uses the Enrichment Bead-Linked Transposomes (Enrichment BLT, eBLT) to tagment DNA followed by post tagmentation clean up. The PCR procedure adds pre-paired 10 base pair adapters and sequences required for sequencing cluster generation. The viral cDNA was used as input for multiple overlapping PCR reactions that spanned the viral genome (Enhanced PCR Mix reagent and nuclease-free water). The amplified tagmented DNA was cleaned with AMPure XP magnetic beads (Beckman Coulter Inc., Indianapolis, USA) to remove shorter DNA fragments and other impurities. We then quantified the cleaned libraries employing Qubit dsDNA BR Assay Kit (Thermo Fisher Scientific, Waltham, USA).

Sequencing was performed on an Illumina Miseq (Illumina, San Diego, USA) using Reagent Kit v3 with 150cycles in a paired-end run, following the manufacturers instructions. All experiments were performed in a biosafety level 2 laboratory.

### Consensus calling

Reference mapping and consensus calling was performed using an in-house developed pipeline managed with Snakemake [80]. Briefly, quality control was performed FastQC v0.11.9 and low-quality reads and adapters were removed using Trimmomatic v0.39 [81]. PCR duplicates were discarded using Picard MarkDuplicates v2.23.8 (https://broadinstitute.github.io/picard/). Reads were mapped to the reference SARS-CoV-2 genome (GenBank accession number NC_045512.2) using burrowswheeler aligner (BWA-MEM) v0.7.17 [82] and unmapped reads were discarded. Consensus sequences were generated using bcftools mpileup combined with bcftools consensus v1.9 [83]. Positions covered by fewer than 10 reads (DP<10) were considered a gap in coverage and converted to ns. Coverage values for each genome were calculated using bedtools v2.26.0 [84] and plotted using the karyoploteR v1.12.4 package [85]. Finally, we assessed genome consensus sequence quality using Nextclade v0.8.1 (https://clades.nextstrain.org/) and CoV-GLUE (http://cov-glue.cvr.gla.ac.uk/ [48];)

### Virome analysis

As the respiratory panel kit used allows the detection of ~40 respiratory viral pathogens, the viral composition of each sample (all mapped and unmapped reads against reference) was verified using Kaiju v1.7.3 [86] and Kraken v2.0.7-beta [87] against a reference database of viral sequences. The viral database for each tool was built with the following commands, respectively: kaiju-makedb -s visuses and kraken2-build --download-library viral. Taxonomic classification interactive charts were visualized using Krona [88].

### Mutation analysis

Sequence positions in this work refer to GenBank RefSeq sequence NC_045512.2, a genome isolated and sequenced from Wuhan (China), early in the pandemic. Single Nucleotide Polymorphisms (SNPs) and insertions/deletions (INDELs) were assessed in each sample by using snippy variant calling and core genome alignment pipeline v4.6.0 (https://github.com/tseemann/snippy), which uses FreeBayes v1.3.2 [89] variant caller and snpEff v5.0 [90] to annotate and predict the effects of variants on genes and proteins. Genome map and histogram of SNPs were generated after running MAFFT v7.471 alignment using a modified code from Lu et al. 2020 (https://github.com/laduplessis/SARS-CoV-2_Guangdong_genomic_epidemiology/). Moreover, we identified global virus lineages using Nextclade v0.8.1 (https://clades.nextstrain.org/) and Pangolin v2.1.3 (https://github.com/cov-lineages/pangolin [15];).

### Phylogenetics analysis

All available SARS-CoV-2 genomes (285,411 sequences) were obtained from GISAID on December 24, 2020. Available sequences were then subjected to analysis inside the NextStrain ncov pipeline (https://github.com/nextstrain/ncov [91];). Briefly, this pipeline uses the augur toolkit to (i) exclude short and low quality sequences or those with incomplete sampling date; (ii) align filtered sequences using MAFFT v7.471 [92]; (iii) mask uninformative sites and ends from the alignment; (iv) perform context subsampling using genetically closely-related genomes to our focal subset prioritizing sequences geographically closer to RS state, Brazil; (v) build maximum likelihood (ML) phylogenetics tree using IQ-TREE v2.0.3 [93], employing the General time reversible model (GTR) with unequal rates and base frequencies [94], (vi) generate a time-scaled tree resolving polytomies and internal nodes with TreeTime v0.7.6, and under a strict clock under a skyline coalescent prior with a rate of 810^4^ substitutions per site per year [95]; (vii) label clades, assign mutations and infer geographic movements; and (viii) export results to JSON format to enable interactive visualization through Auspice. The ML tree was inspected in TempEst v1.5.3 [96] to investigate the temporal signal through regression of root-to-tip genetic divergence against sampling dates.

### Phylodynamic and phylogeographic analysis

All global sequences (until December 24, 2020) belonging to lineages B.1.1.248 (*n*=405) and B.1.1.33 (*n*=725), found in high frequency in this study, were recovered from the filtered MAFFT alignment performed inside Nextstrain ncov pipeline in the previous step. The T_MRCA_ and the spatial diffusion of these important circulating lineages through Brazil were separately estimated for each lineage using a Bayesian Markov Chain Monte Carlo (MCMC) approach as implemented in BEAST v1.10.4 [97], using the BEAGLE library v3 [98] to save computational time. Time-scaled Bayesian trees were estimated in BEAST using: HKY+ nucleotide substitution model, a strict molecular clock model with a Continuous Time Markov Chain (CTMC) prior (mean rate=810^4^) for the clock rate [99], and a parametric exponential growth model.

Two MCMC chains were run for at least 120 million generations and convergence of the MCMC chains was inspected using Tracer v1.7.1 [100]. After removal of 10% burn-in, log and tree files were combined using LogCombiner v1.10.4 [97] to ensure stationarity and good mixing. Maximum clade credibility (MCC) summary trees were generated using TreeAnnotator v1.10.4 [97]. MCC trees were visualized using FigTree v1.4 (http://tree.bio.ed.ac.uk/software/figtree/) and additional annotations were performed in ggtree R package v2.0.4 [101].

Viral migrations across time were reconstructed using a reversible discrete asymmetric phylogeographic model [102] in order to estimate locations of each internal node of the phylogeny. SpreaD3 [103] was used to map spatiotemporal information embedded in MCC trees. A discretization scheme of 10 possible states defined as Brazilian regions (Centre-West, North, Northeast, South, and Southeast) or other continents (Africa, Europe, North America, Oceania, and South America) was applied. For map plotting, latitudes and longitudes were attributed to a randomly selected point next to the center of each region or continent. Location exchange rates that dominate the diffusion process were identified using the Bayesian stochastic search variable selection (BSSVS) procedure [102] using Bayes Factor tests to identify well-supported rates.

## Supplementary Information

**Additional file 1.**

**Additional file 2.**

**Additional file 3.**

**Additional file 4.**

## Data Availability

The FASTQ data generated in this study have been submitted to the NCBI SRA database (https://www.ncbi.nlm.nih.gov/sra) under BioProject accession number PRJNA707583. Consensus genomes have been submitted to the GISAID database (https://www.gisaid.org/) under accession numbers listed in Table 1 (EPI_ISL_831678, EPI_ISL_831474, EPI_ISL_831645, EPI_ISL_831646, EPI_ISL_831660, EPI_ISL_831681, EPI_ISL_831683, EPI_ISL_831685, EPI_ISL_831688, EPI_ISL_831689, EPI_ISL_831892, EPI_ISL_831898, EPI_ISL_831913, EPI_ISL_831938, EPI_ISL_831939, EPI_ISL_831940, EPI_ISL_832009, EPI_ISL_832010, EPI_ISL_832011, EPI_ISL_832012, EPI_ISL_832013). The publicly available sequences used are listed in the Additional File 3. An interactive Microreact visualization is available at https://microreact.org/project/9ia4CJbx1mF52Ew1g7e9uT. Additional information used and/or analysed during the current study are available from the corresponding author on reasonable request.

## Abbreviations

ACE2: Angiotensin-converting enzyme 2
BF: Bayes Factor
BSSVS: Bayesian Stochastic Search Variable Selection
CFR: Case fatality rate
CTMC: Continuous Time Markov Chain
CONEP: Brazilians National Ethics Committee (Comisso Nacional de tica em Pesquisa)
DP: Depth of coverage
GISAID: Global Initiative on Sharing Avian Influenza Data
GTR: General time reversible model
HPD: Highest Posterior Density
INDEL: Insertion/deletion
MCC: Maximum clade credibility
MCMC: Markov chain Monte Carlo
ML: Maximum Likelihood
ORF: Open Reading Frame
PP: Posterior Probability
R_0_: Reproduction number
RBD: Receptor Binding Domain
RS: Rio Grande do Sul state, Southern Brazil
RTq-PCR: Real Time Reverse-transcriptase Polymerase Chain Reaction
SARS-CoV-2: *Severe acute respiratory syndrome coronavirus 2*
SNP: Single Nucleotide Polymorphism
TMPRSS2: transmembrane protease serine 2
VTM: Virus Transport Medium
WHO: World Health Organization
