# Supplementary material for: Genomic epidemiology of SARS-CoV-2 in Esteio, Rio Grande do Sul, Brazil
Source: BMC Genomics. 2021 May 20;22:371. doi: 10.1186/s12864-021-07708-w (PMC8136996; doi:10.1186/s12864-021-07708-w)

Additional Figure 1 . Sequencing depth of coverage for Sample 1

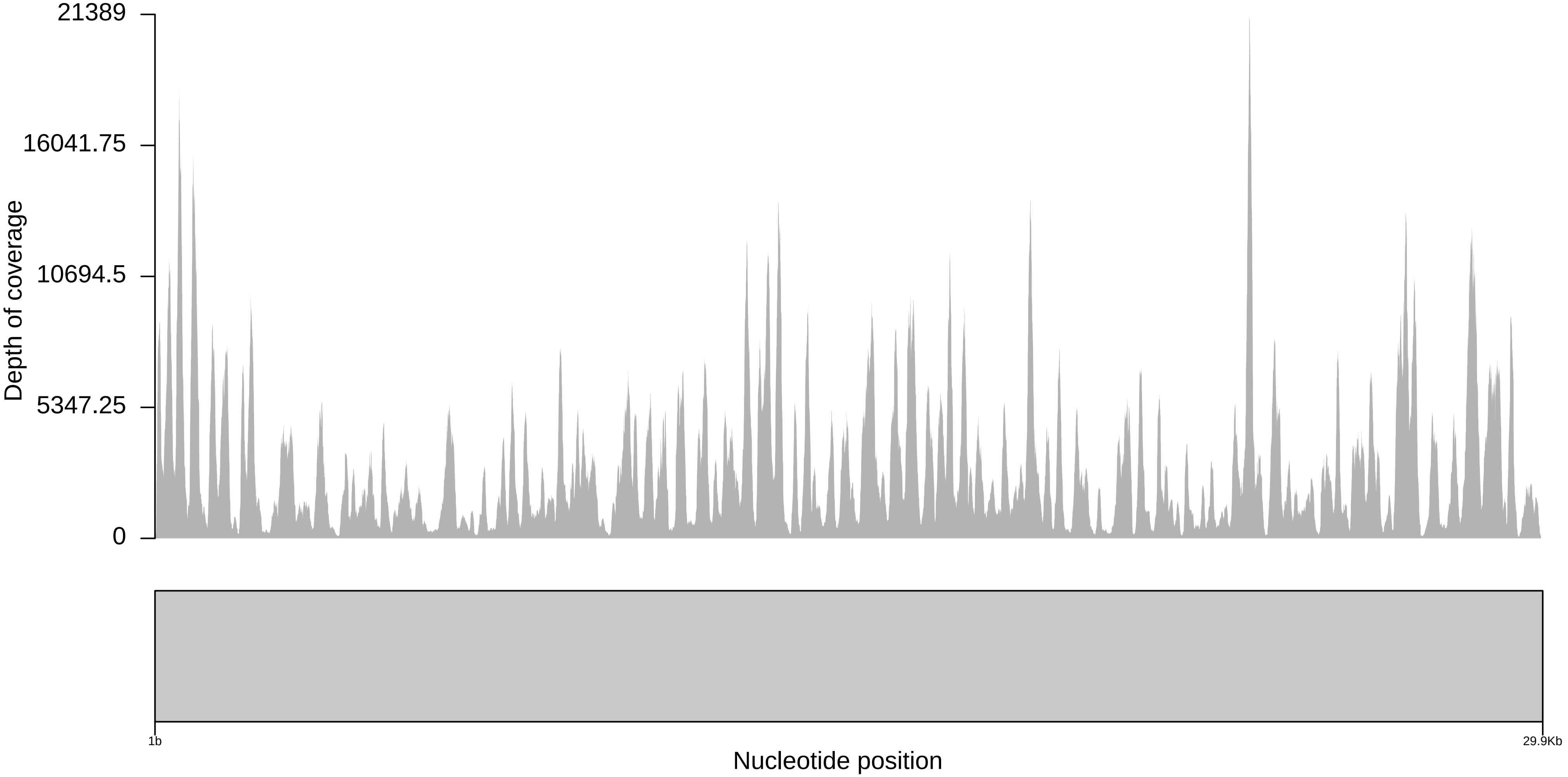

Additional Figure 2 . Sequencing depth of coverage for Sample 2

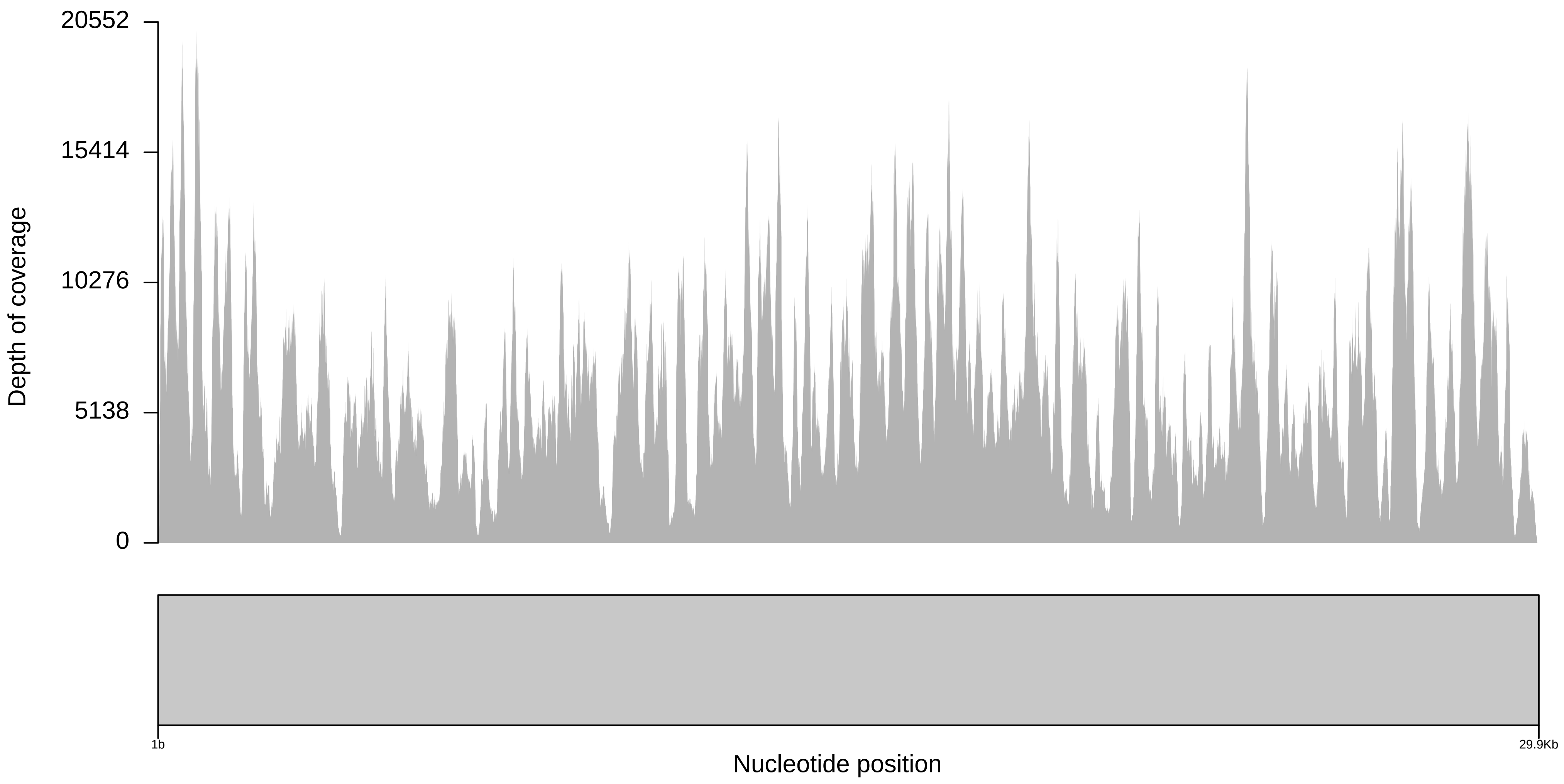

Additional Figure 3 . Sequencing depth of coverage for Sample 3

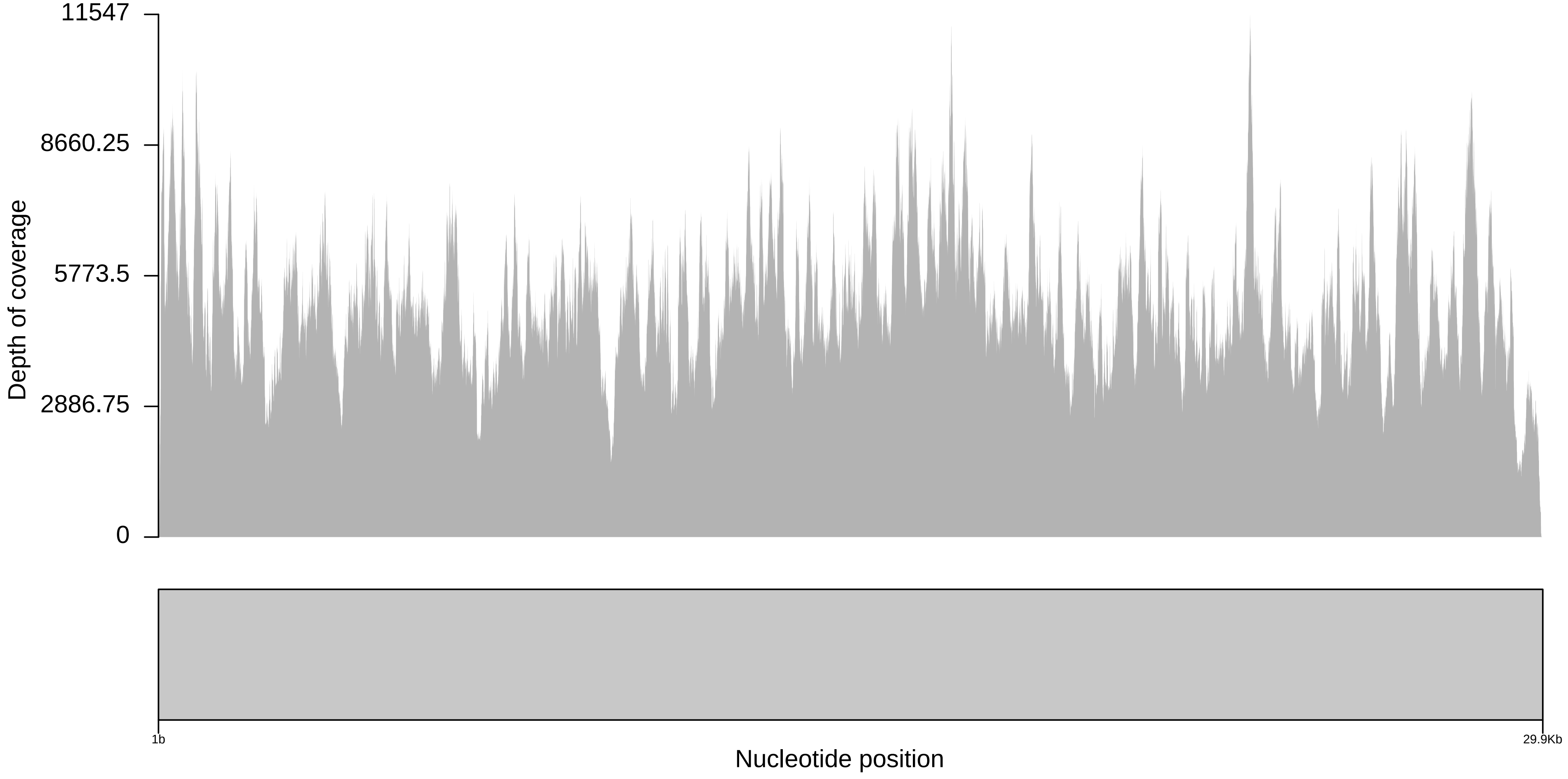

Additional Figure 4 . Sequencing depth of coverage for Sample 4

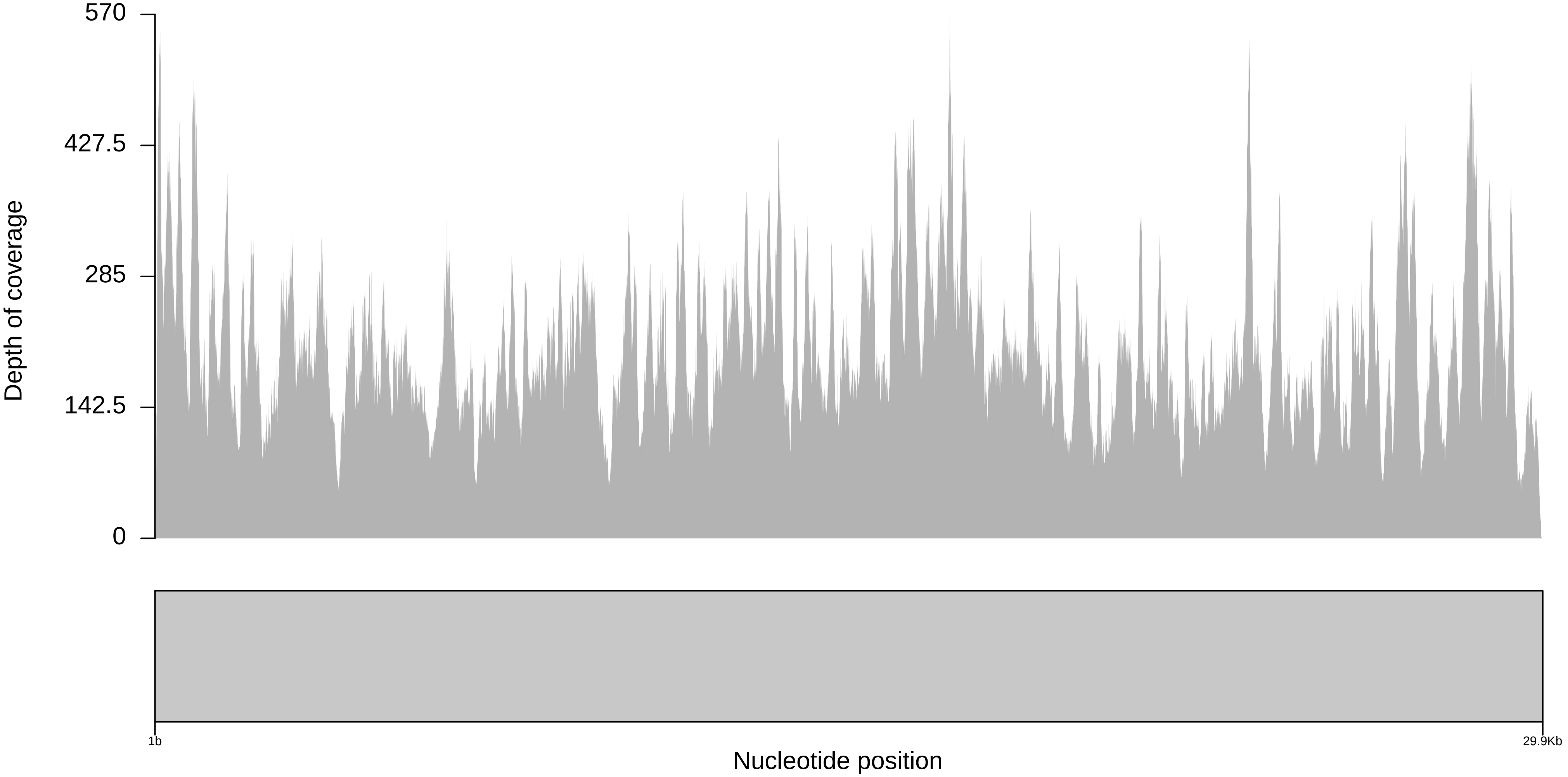

Additional Figure 5 . Sequencing depth of coverage for Sample 5

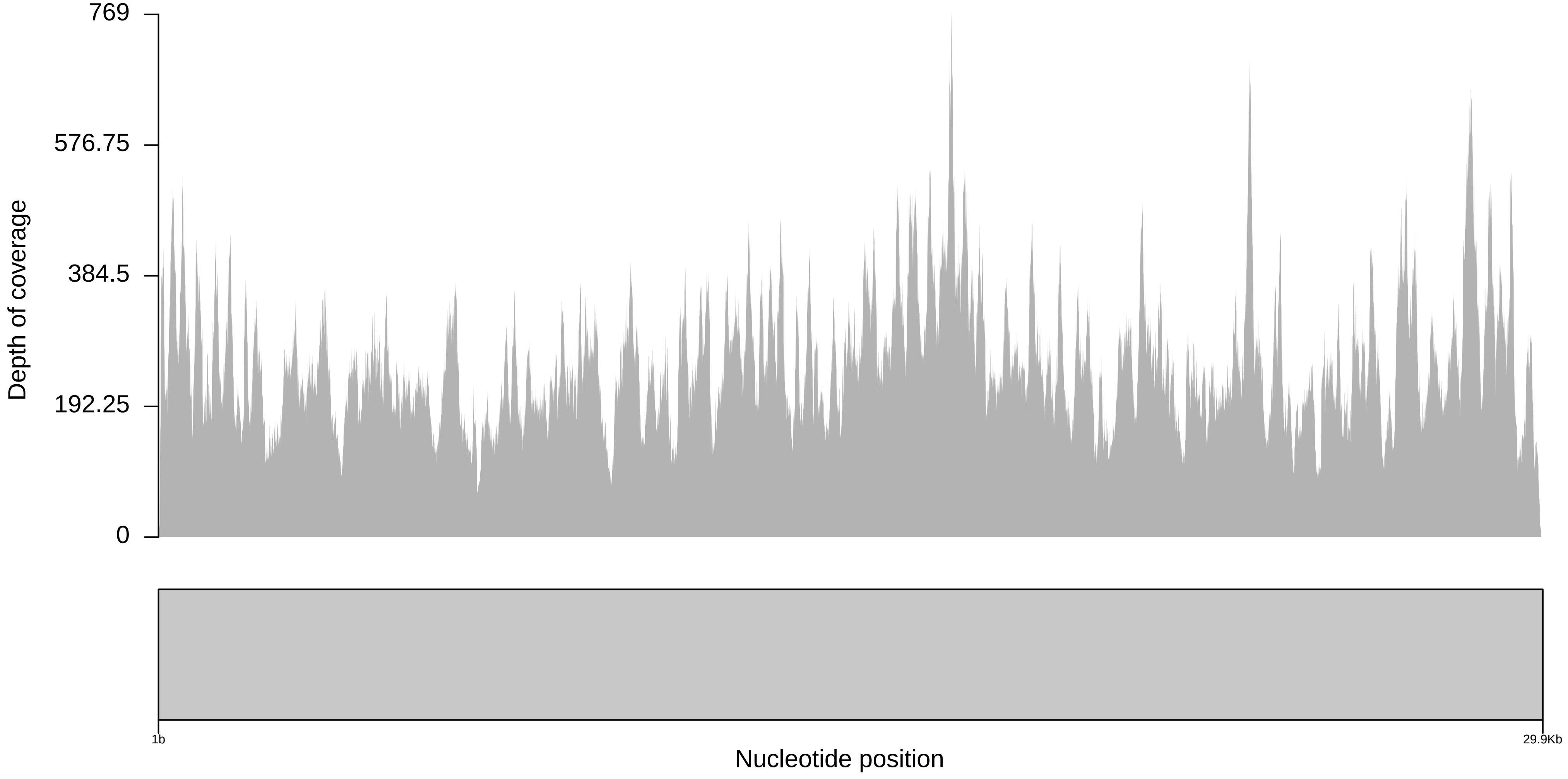

Additional Figure 6 . Sequencing depth of coverage for Sample 6

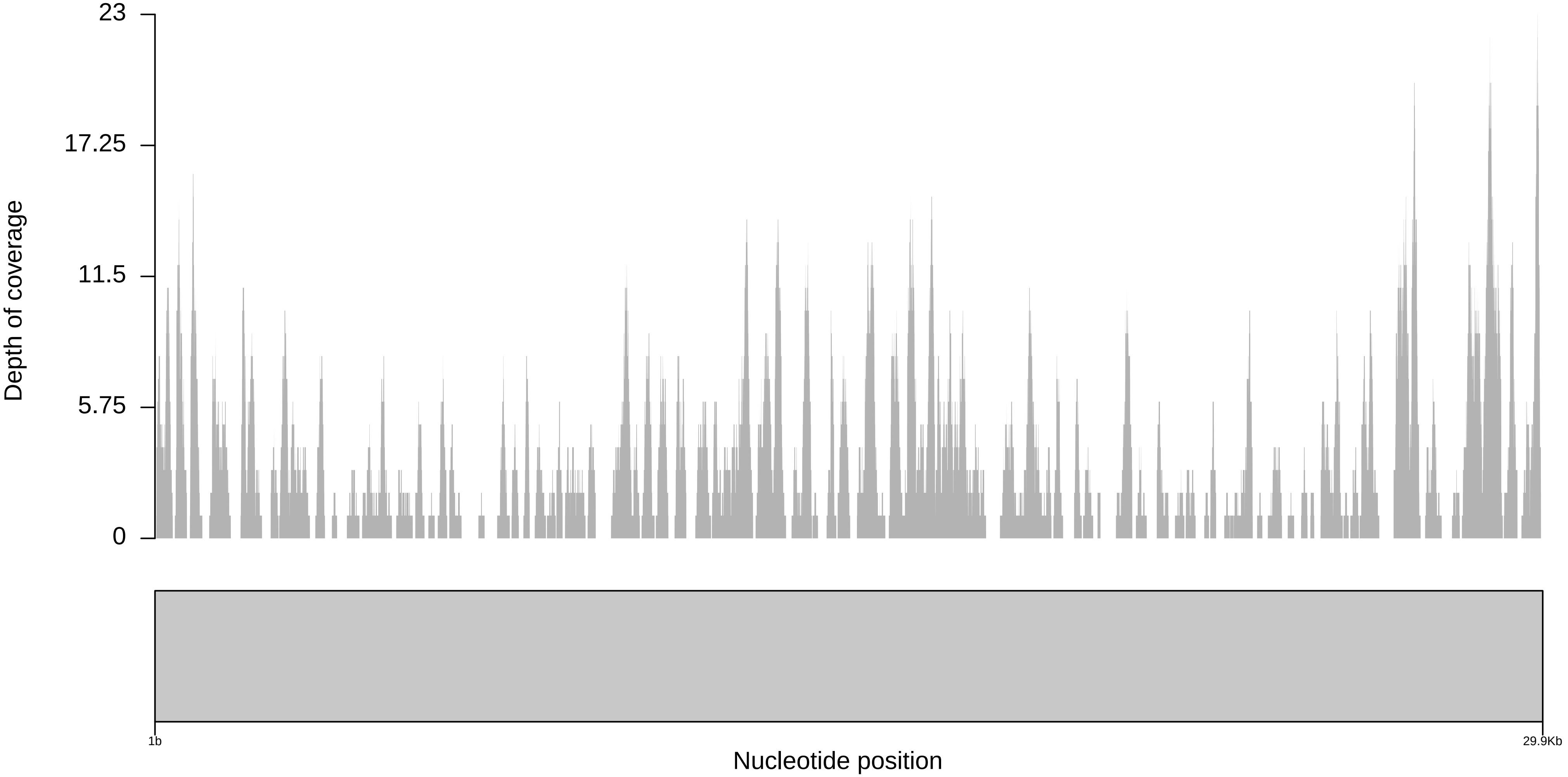

Additional Figure 7 . Sequencing depth of coverage for Sample 7

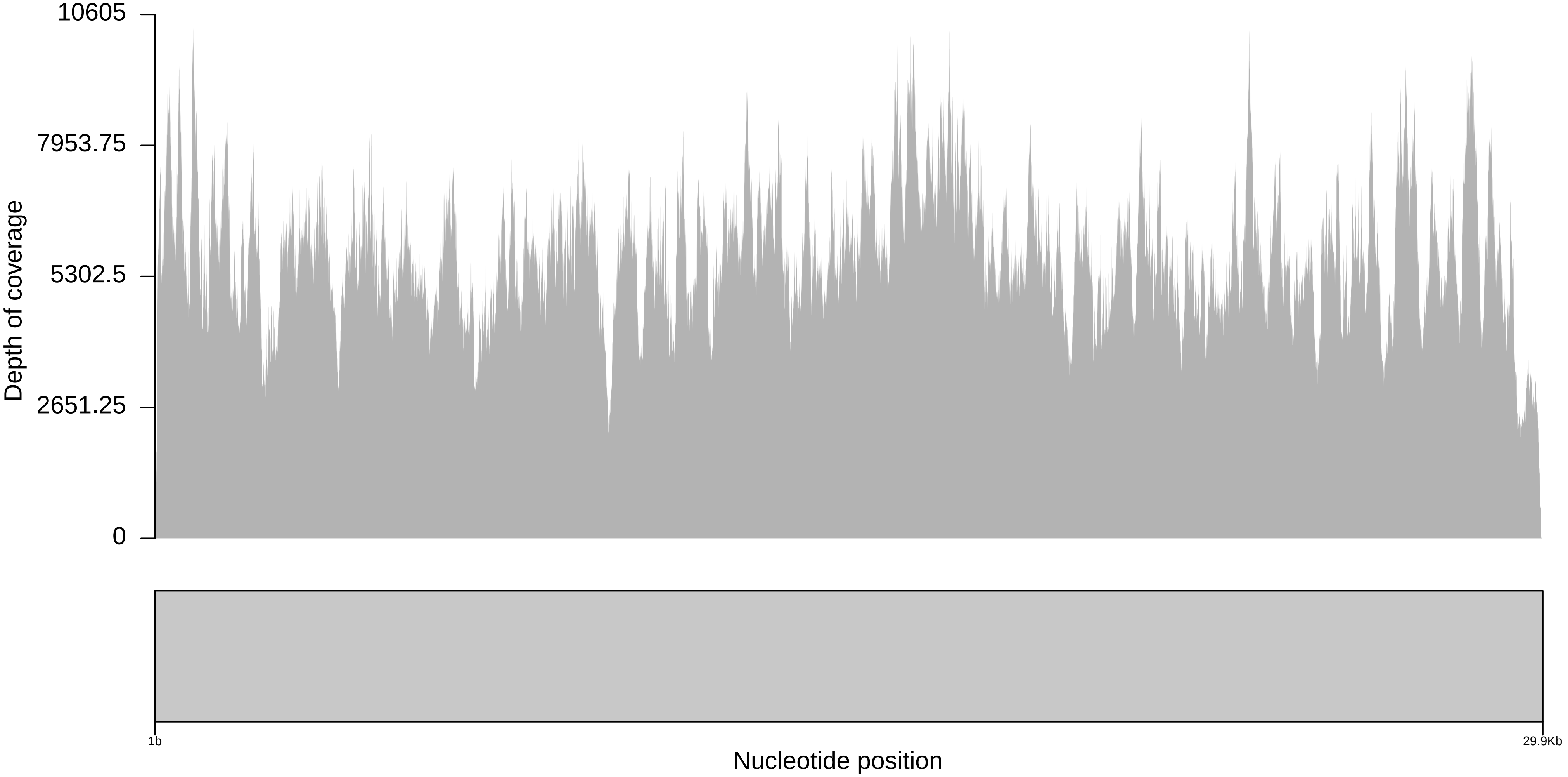

Additional Figure 8 . Sequencing depth of coverage for Sample 8

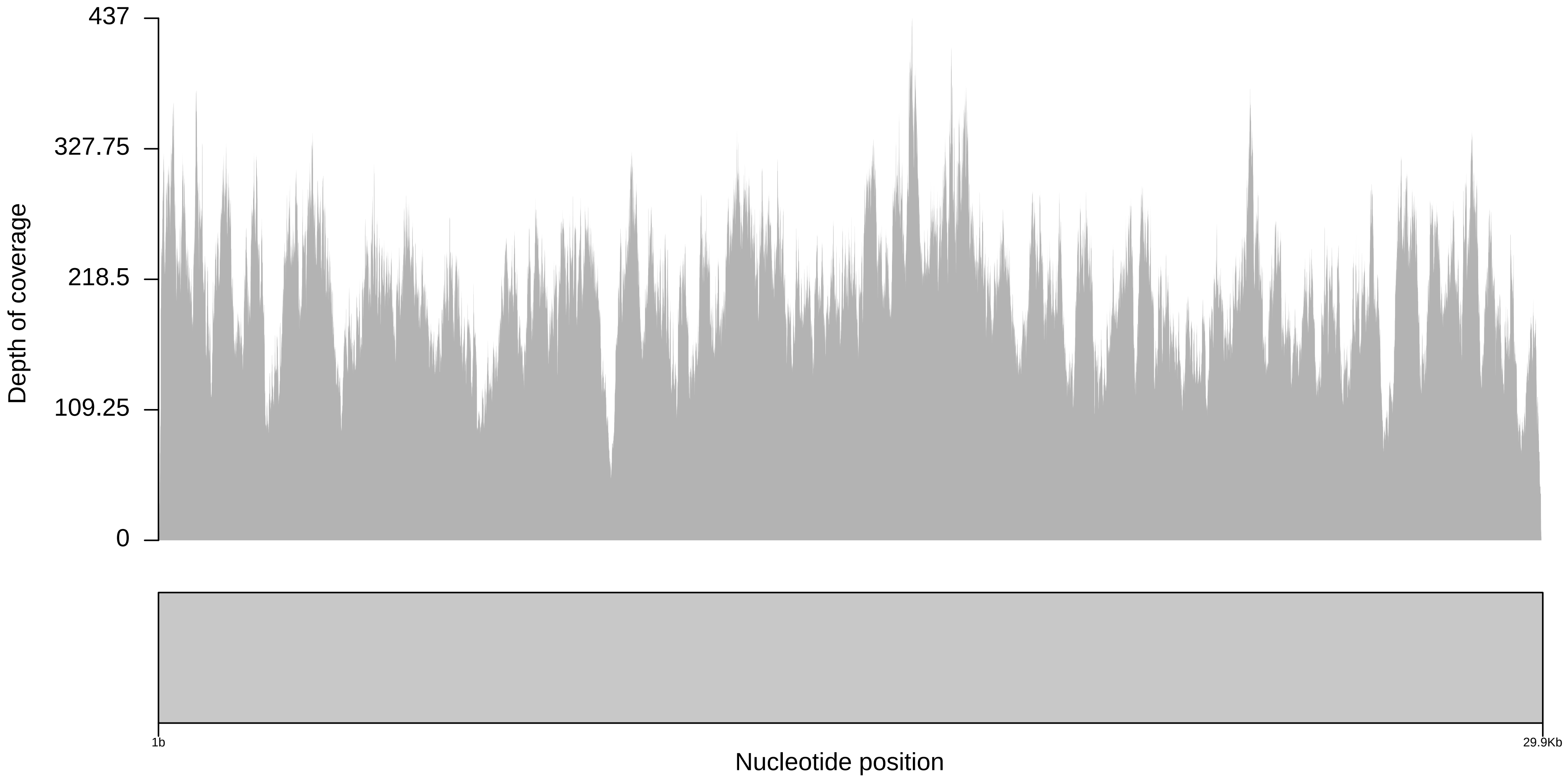

Additional Figure 9 . Sequencing depth of coverage for Sample 9

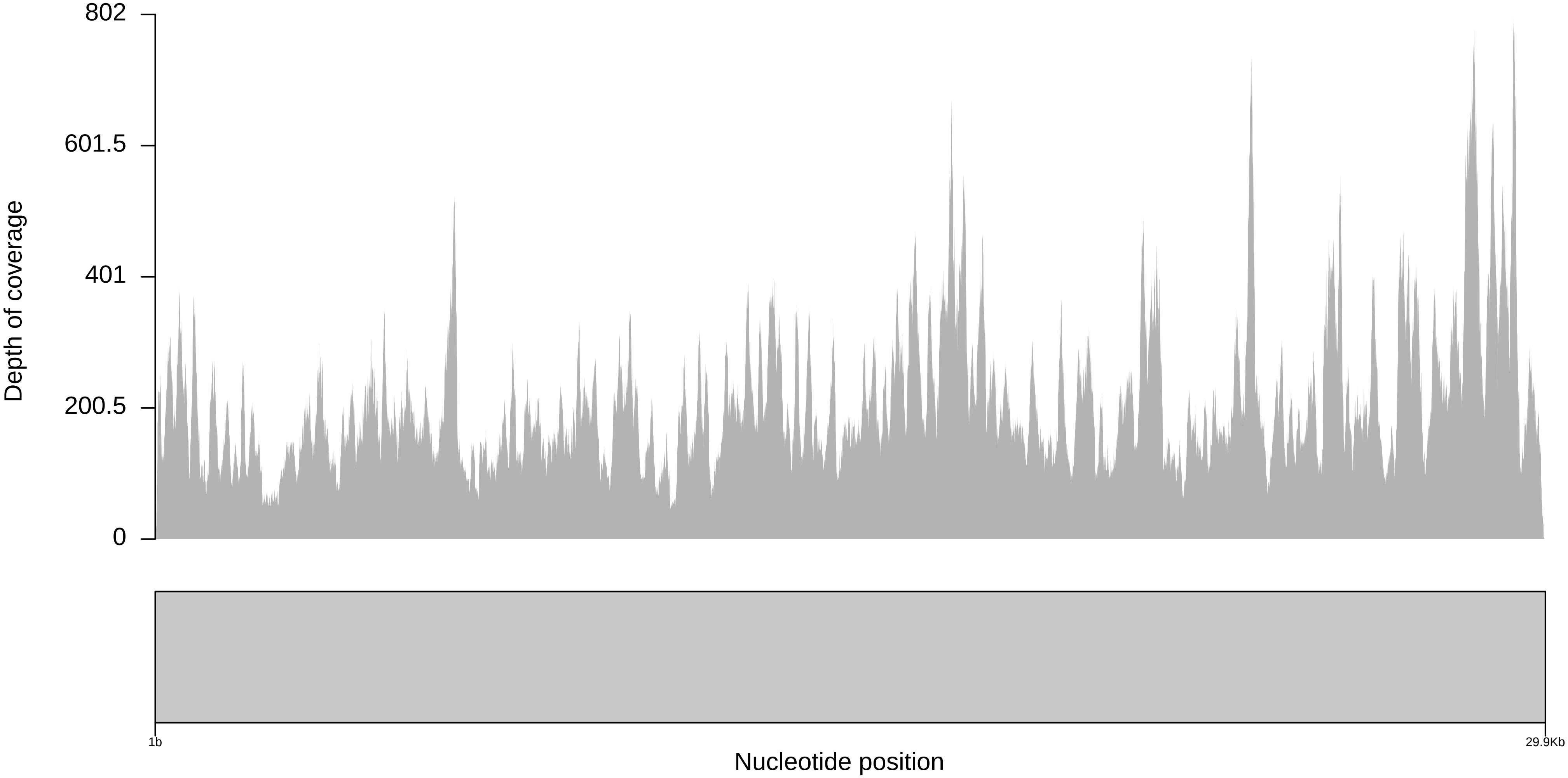

Additional Figure 10 . Sequencing depth of coverage for Sample 10

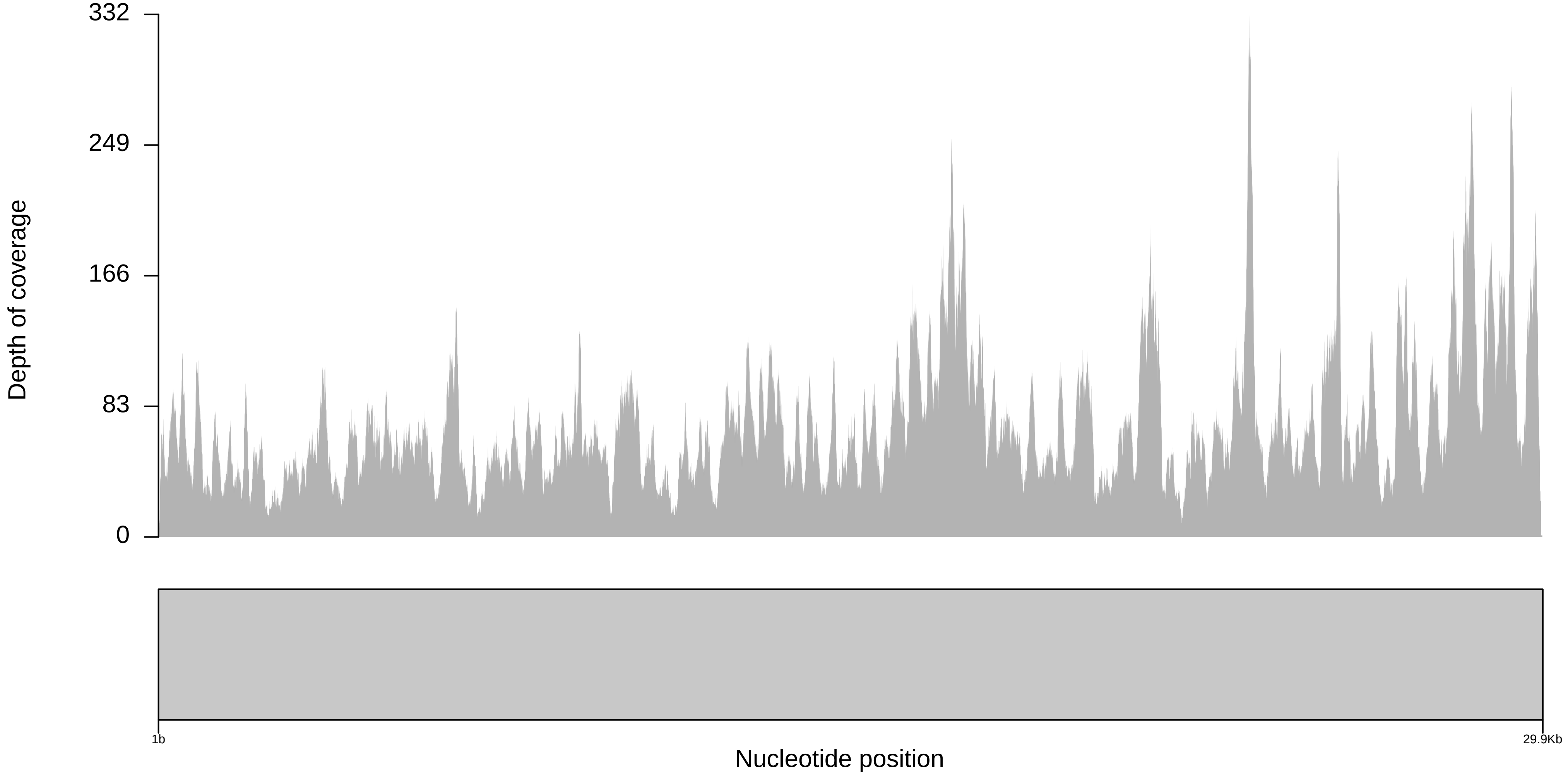

Additional Figure 11 . Sequencing depth of coverage for Sample 11

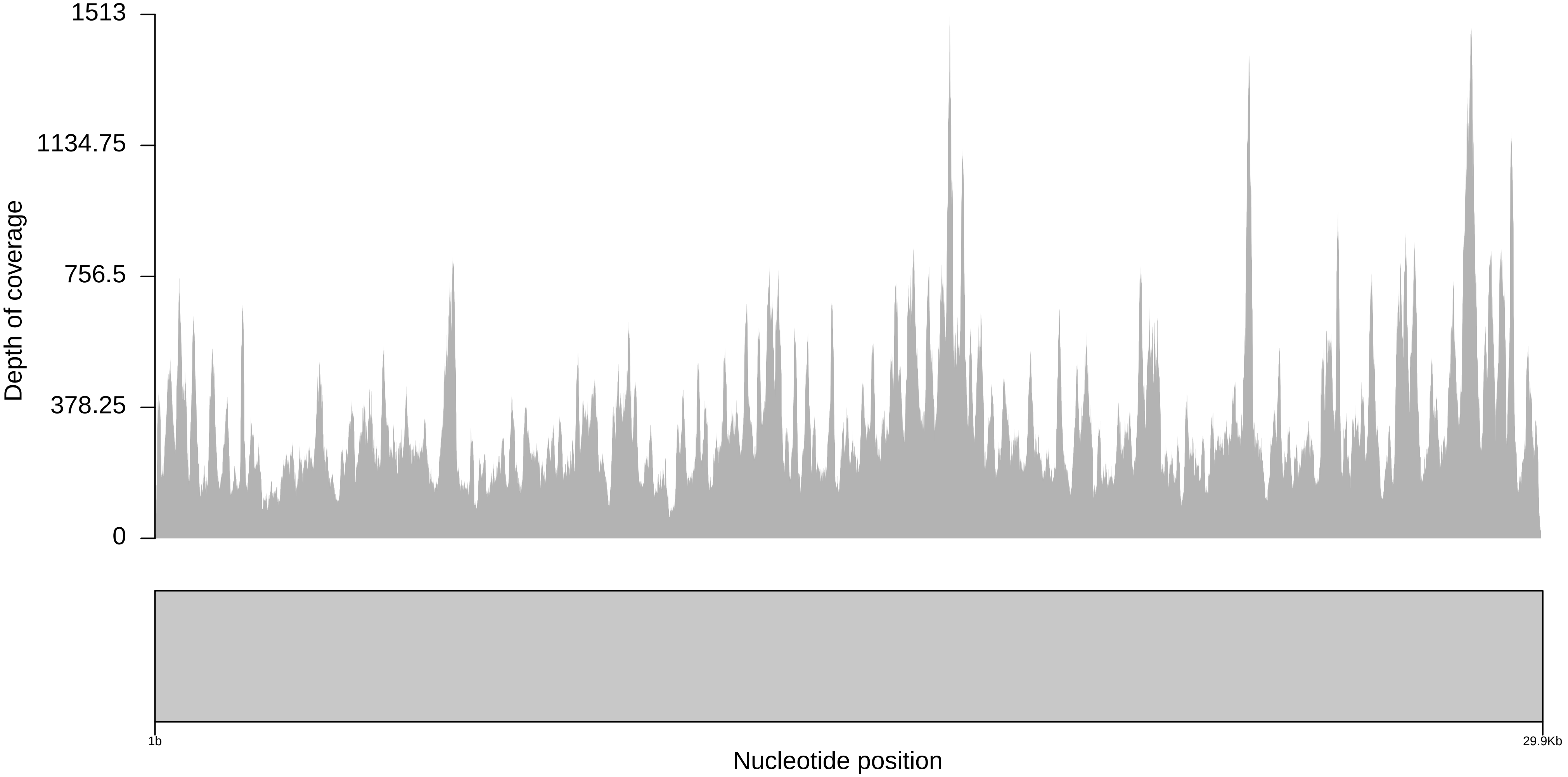

Additional Figure 12 . Sequencing depth of coverage for Sample 12

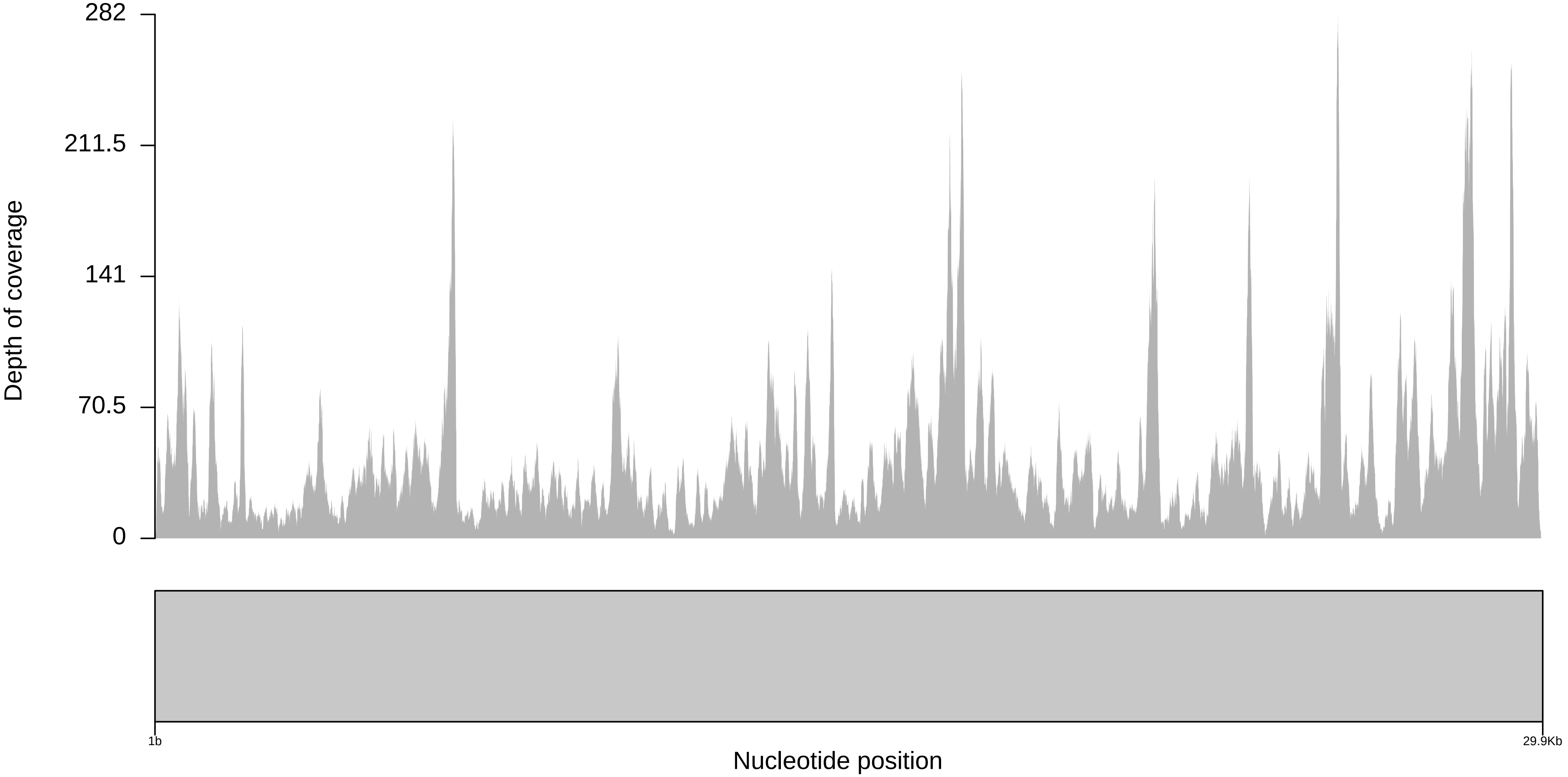

Additional Figure 13 . Sequencing depth of coverage for Sample 13

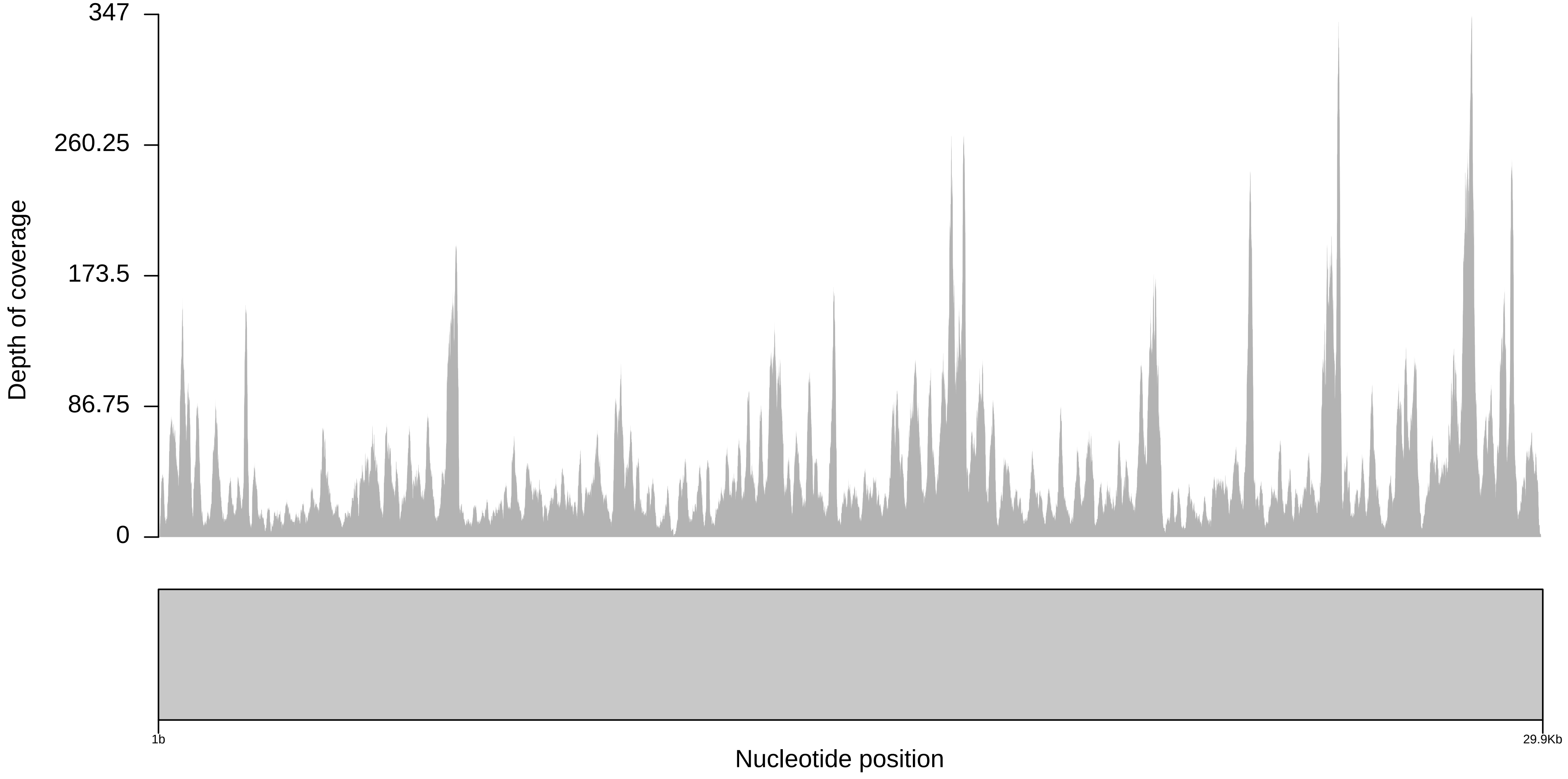

Additional Figure 14 . Sequencing depth of coverage for Sample 14

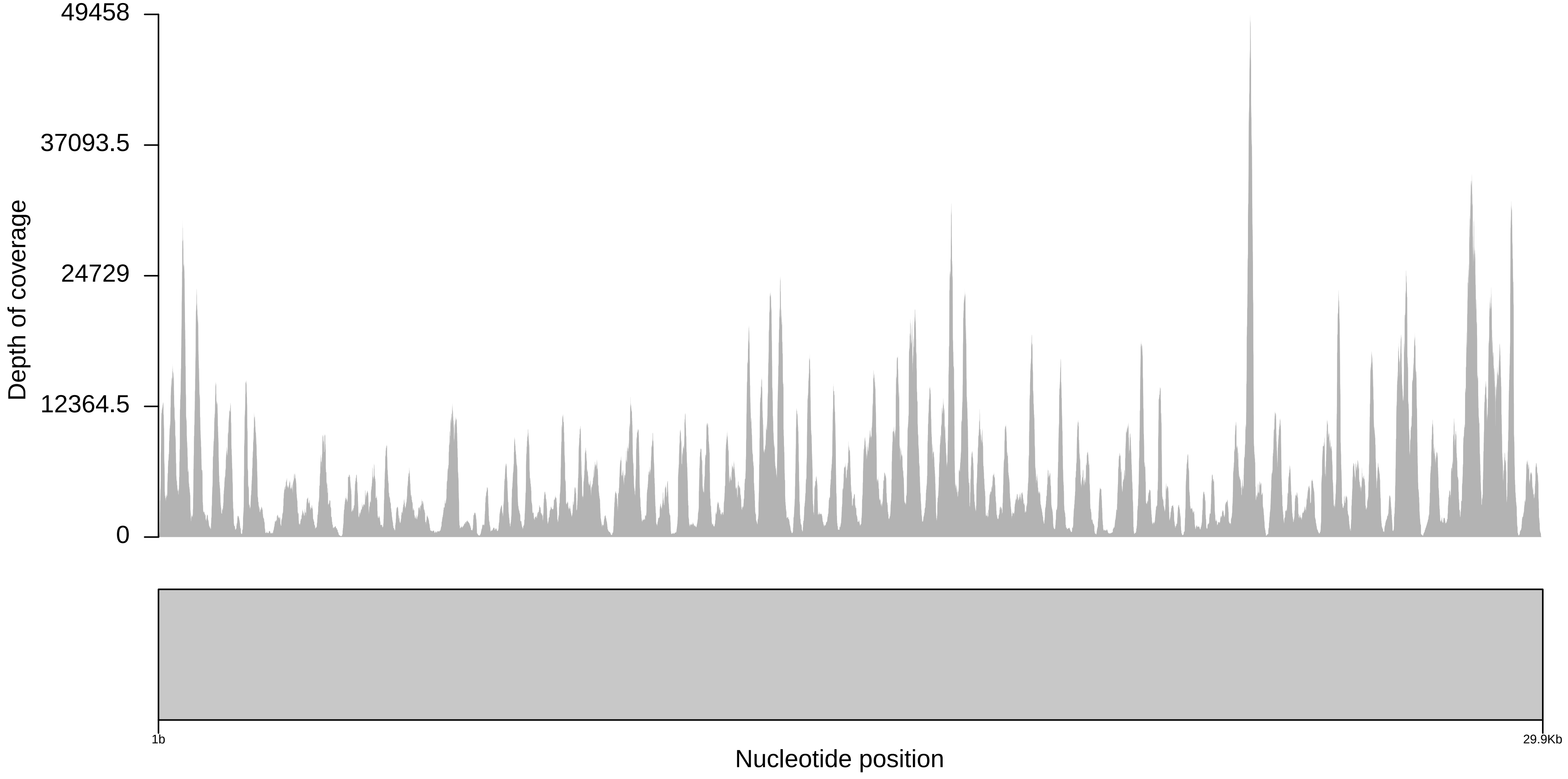

Additional Figure 15 . Sequencing depth of coverage for Sample 15

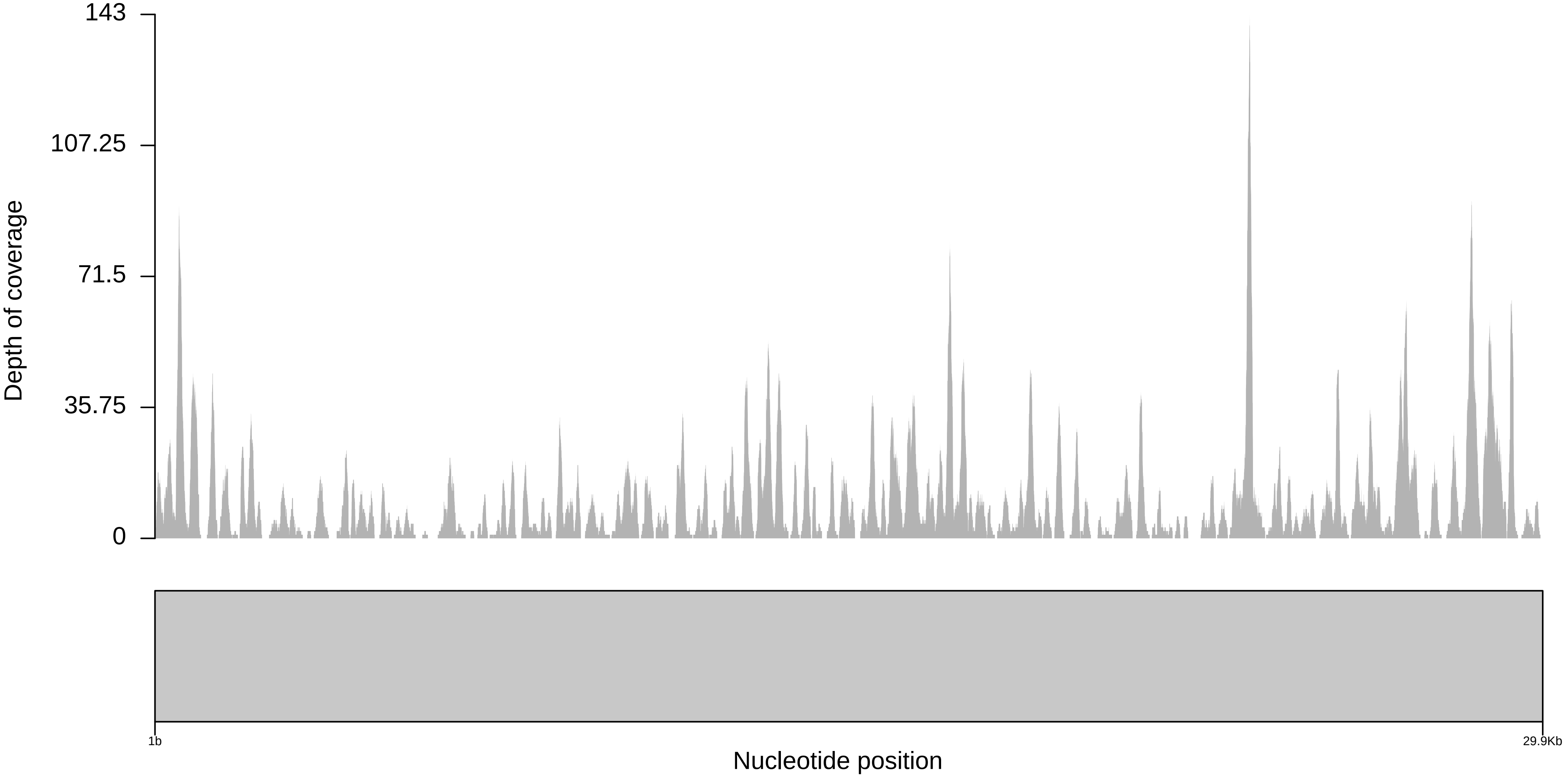

Additional Figure 16 . Sequencing depth of coverage for Sample 16

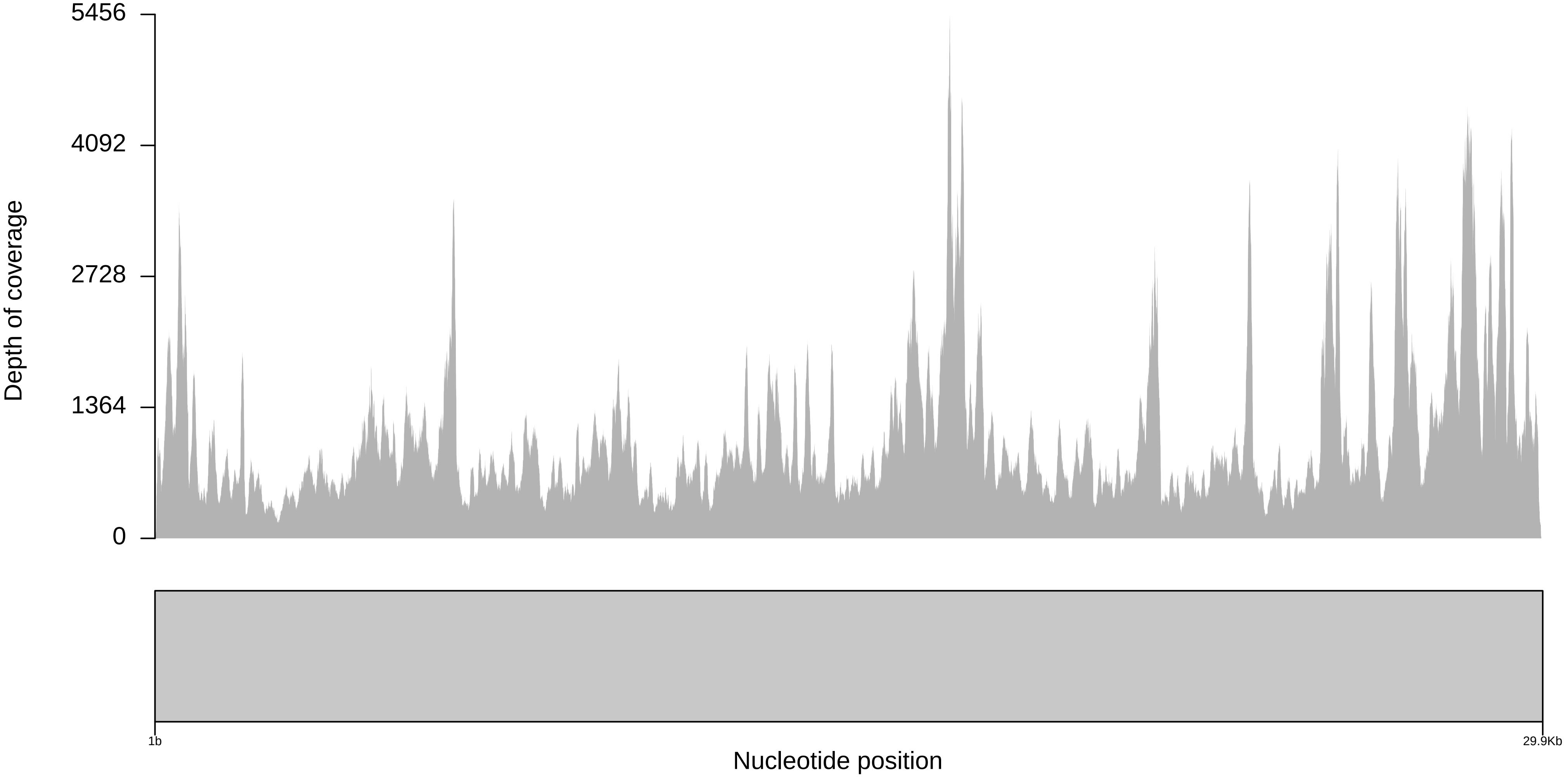

Additional Figure 17 . Sequencing depth of coverage for Sample 17

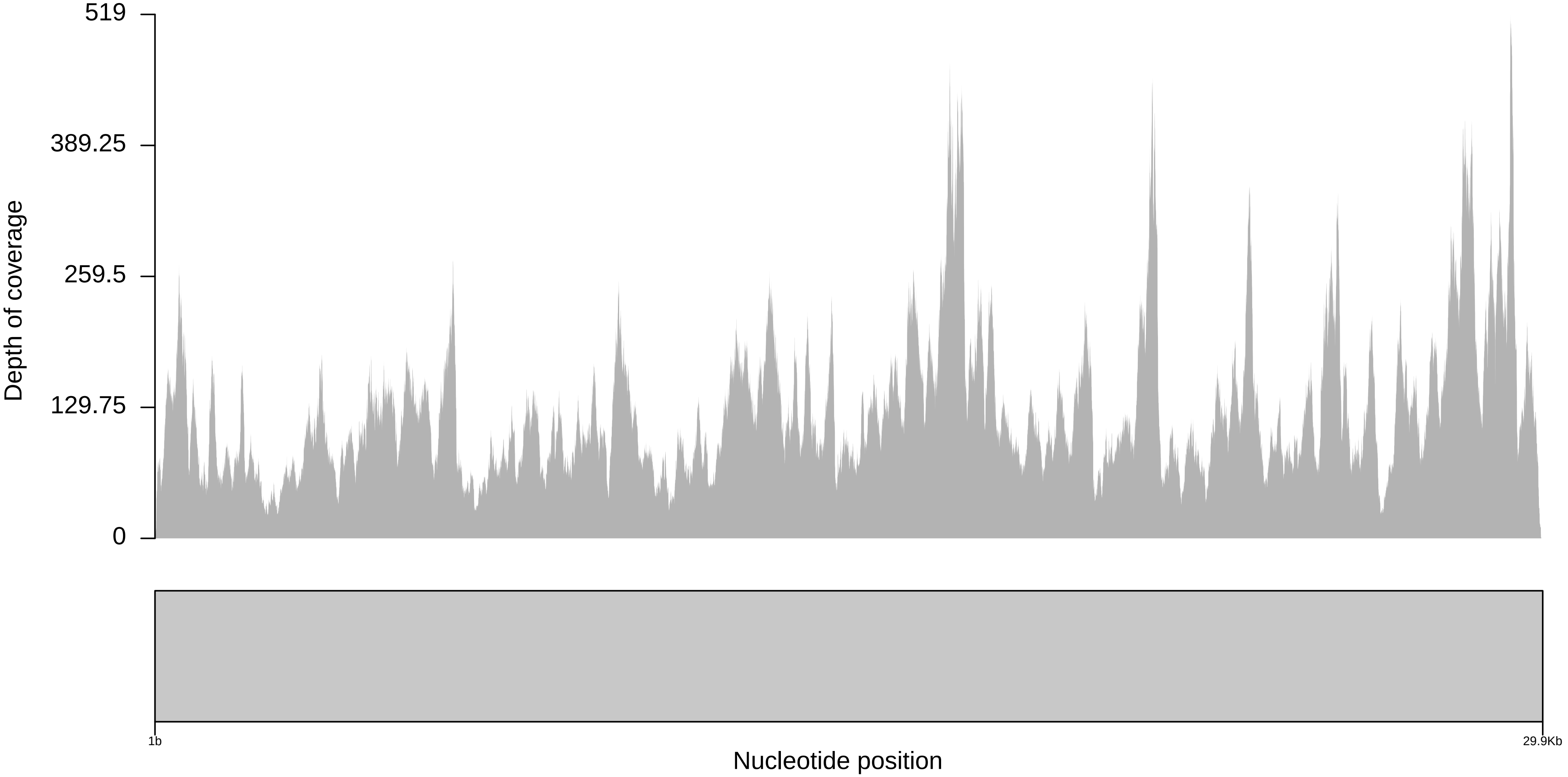

Additional Figure 18 . Sequencing depth of coverage for Sample 18

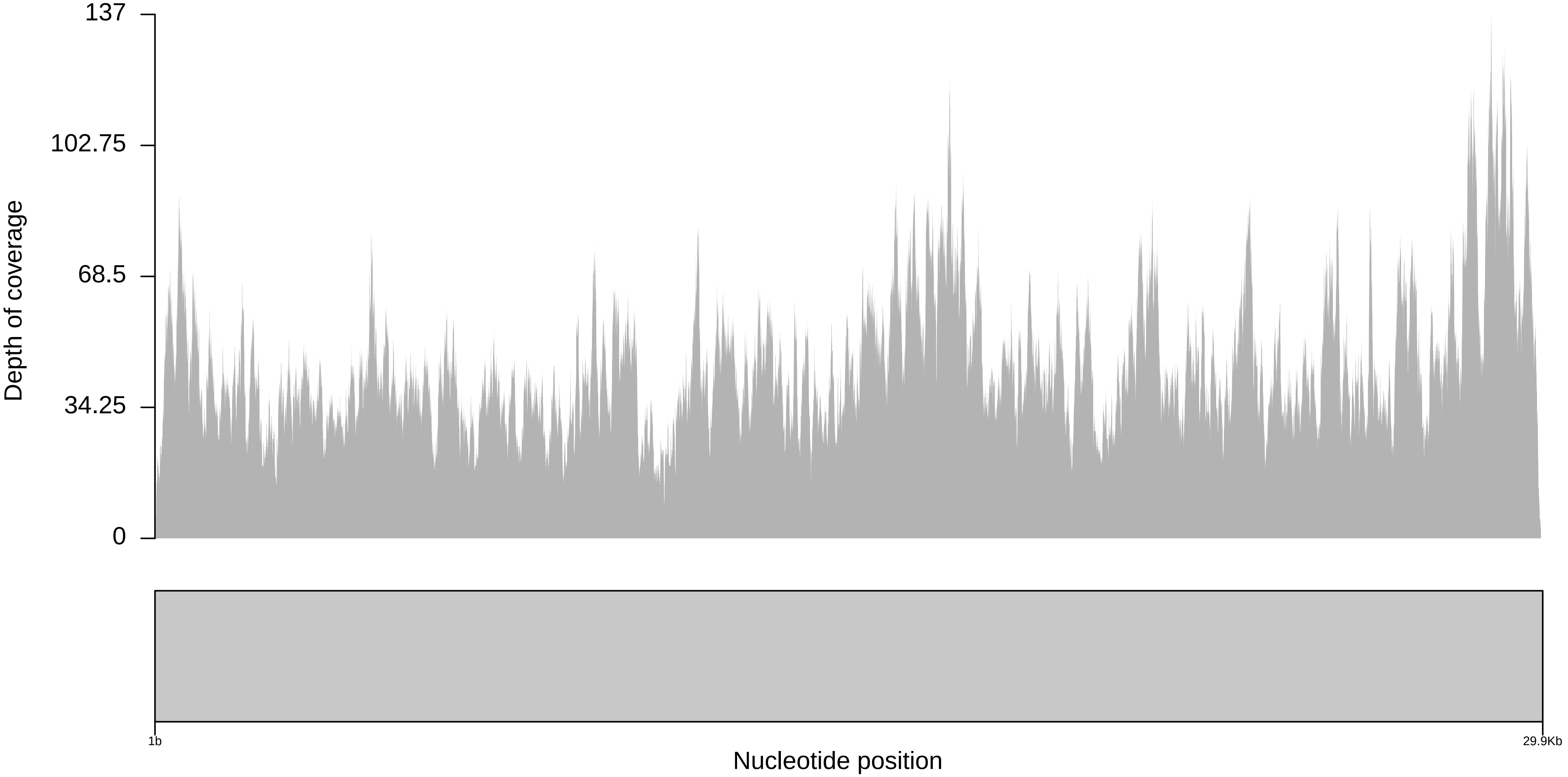

Additional Figure 19 . Sequencing depth of coverage for Sample 19

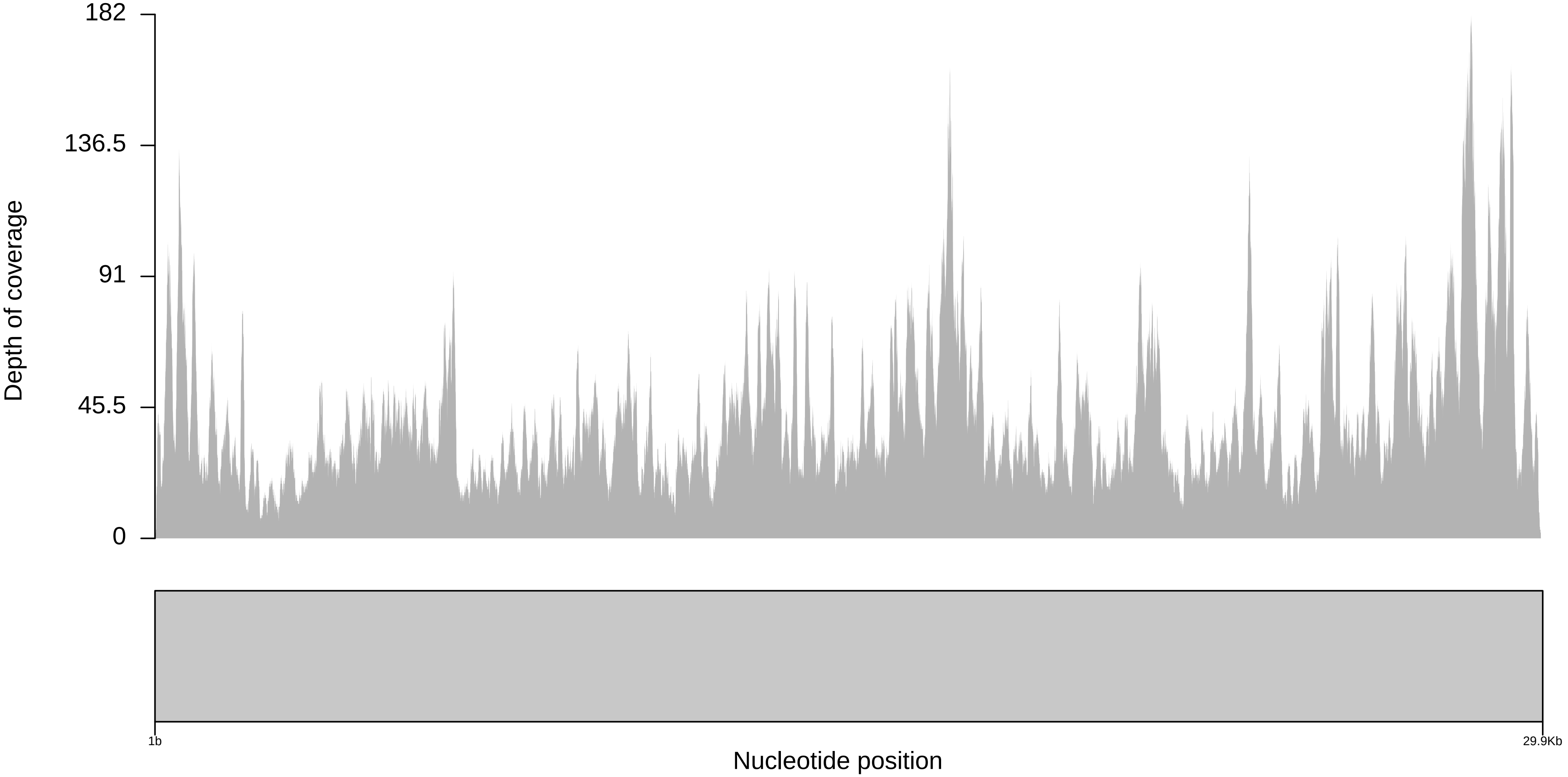

Additional Figure 20 . Sequencing depth of coverage for Sample 20

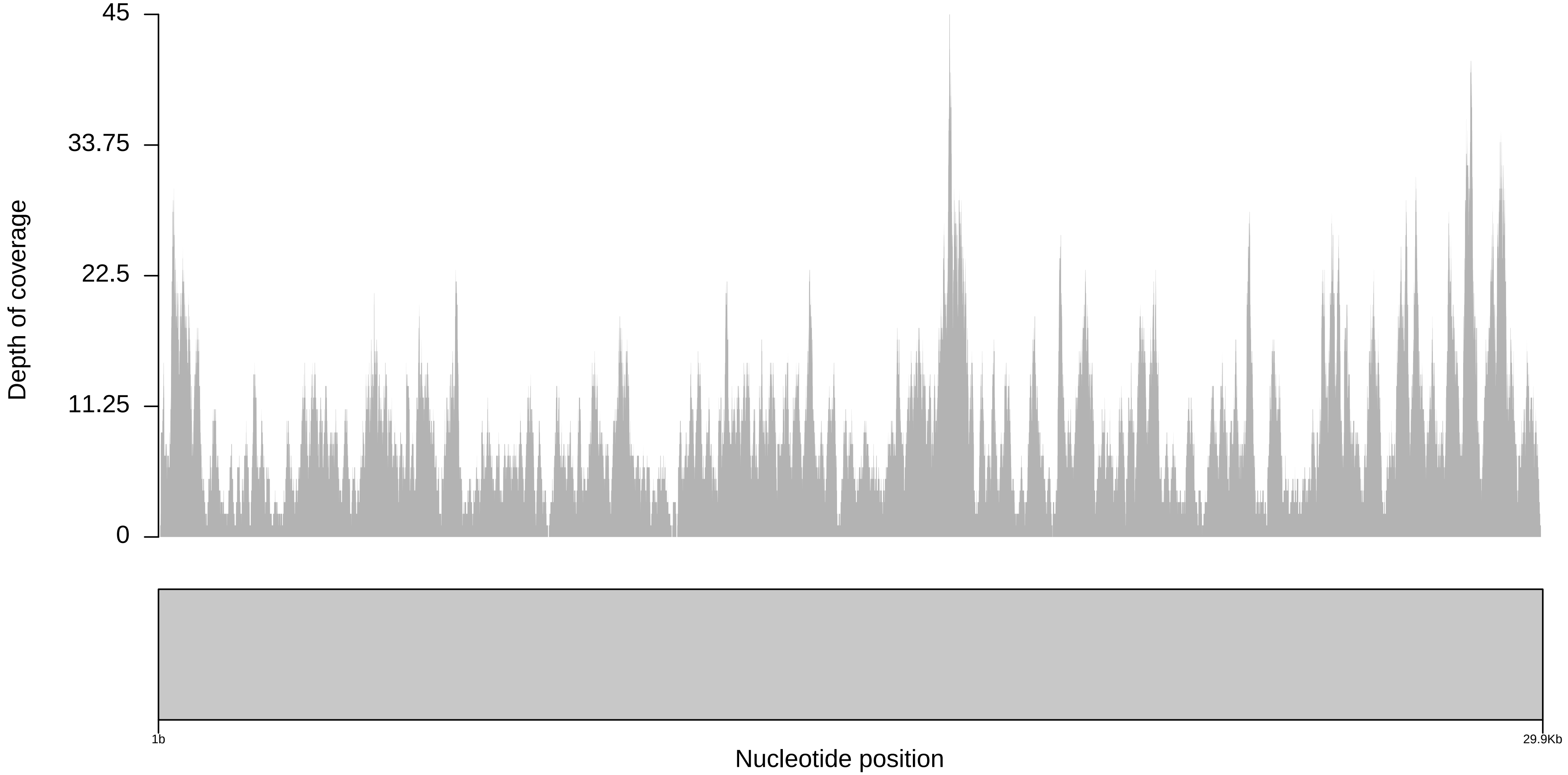

Additional Figure 21 . Sequencing depth of coverage for Sample 21

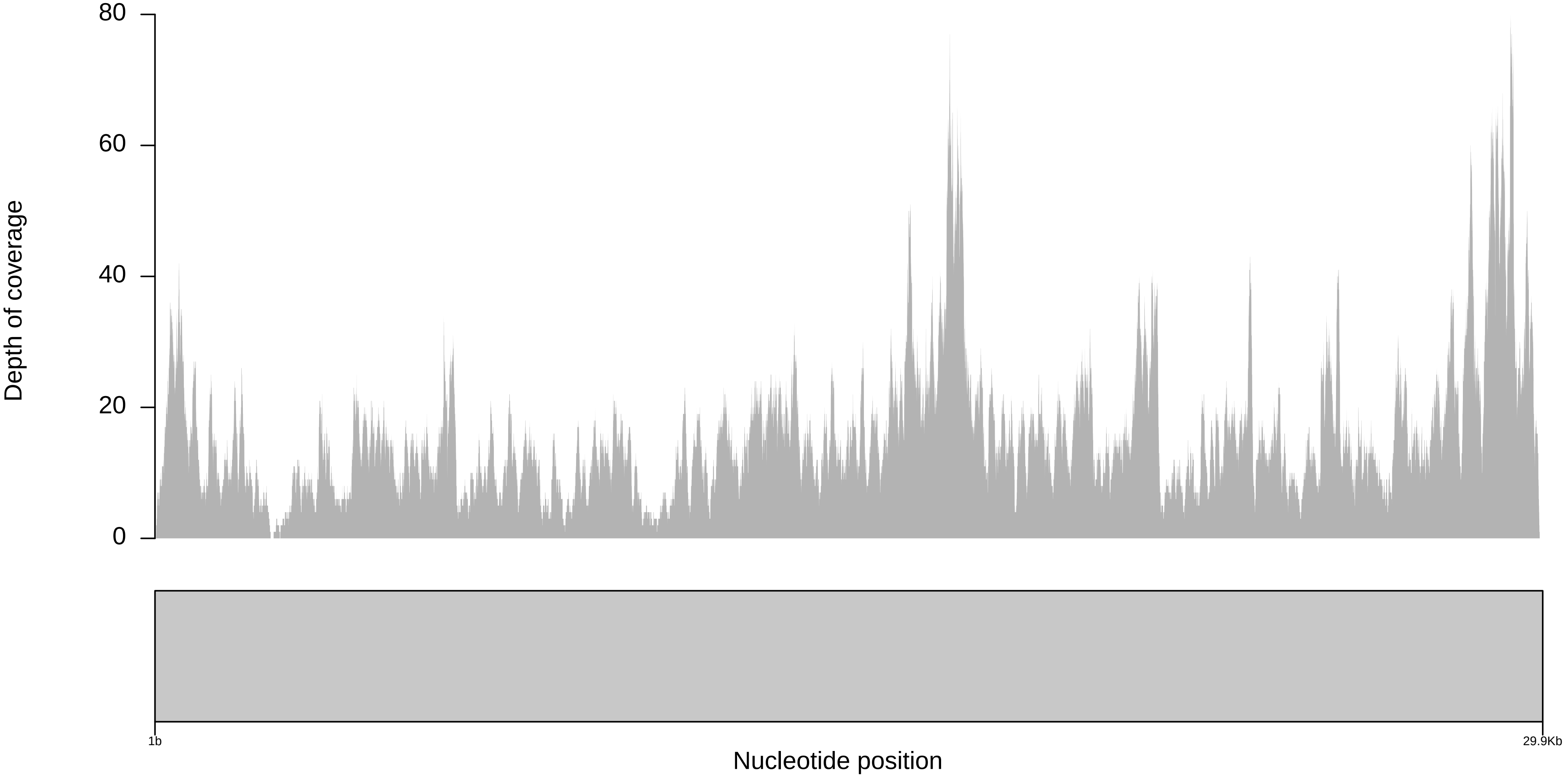

Supplement: Supplementary file 1 — Additional file 1. [file 12864_2021_7708_MOESM1_ESM.pdf]
