# Supplementary material for: Genomic epidemiology of SARS-CoV-2 in Esteio, Rio Grande do Sul, Brazil
Source: BMC Genomics. 2021 May 20;22:371. doi: 10.1186/s12864-021-07708-w (PMC8136996; doi:10.1186/s12864-021-07708-w)
Supplement: Supplementary file 2 — Additional file 2. [file 12864_2021_7708_MOESM2_ESM.pdf]

Supplemental File 2. Detailed view of mutations across all samples. Nucleotide mutation positions are represented as rows. Each mutation has its own color. Cluster 1 and cluster 2 specific-mutations are colored in black and burnt yellow, respectively. Mutations related to Rio de Janeiro's new lineage (P.2) are colored in red.

Sample	1	2	3	4	5	6	7	8	9	10	11	12	13	14	15	16	17	18	19	20	21
	241	241	147	241	147	28883	241	241	241	241	241	241	241	241	106	241	241	241	241	16207	241
	3037	3037	241	3037	241		3037	2276	3037	3037	2276	3037	3037	3037	241	1218	2675	3037	3037	25245	3766
	8094	11211	850	14408	850		11283	3037	12053	4706	3037	14408	13804	7005	23403	3037	3037	3766	7393	25494	4423
	14408	11758	3037	14805	3037		13804	4644	14408	9167	8802	15720	14408	12053	28881	5339	7005	6218	12053	27205	12964
	23403	12484	12053	15654	6896		14408	14408	23403	12053	10681	21191	19972	14408	28882	14408	12053	10667	14408	27299	14408
	27299	14408	14408	23403	12053		23400	18252	25088	14408	14408	23403	20871	23403	28883	15654	14408	11824	14593	27501	23012
	28881	20679	20016	25718	14408		27299	23403	25429	16912	18252	23455	23403	25088		23403	23403	12053	23403	28881	23403
	28882	23403	23403	27299	20016		28881	27299	25509	23403	18395	25062	24928	25429		27299	25088	12964	25088	28882	28253
	28883	27299	25088	28881	23403		28882	28093	27976	23593	22254	27299	28881	25509		28881	25429	14408	25429	28883	28628
Mutations	29148	28881	25207	28882	25088		28883	28881	28881	25088	23403	28881	28882	26019		28882	25509	23012	25509	29148	28881
		28882	25642	28883	25207		29148	28882	28882	27828	27299	28882	28883	27976		28883	26019	23403	27976		28882
		28883	28393	29148	25642			28883	28883	28881	28093	28883	29148	28881		29148	27976	25088	28881		28883
		29148	28881		28393			29148		28882	28881	29148	29750	28882			28881	28253	28882		28975
		29224	28882		28881					28883	28882			28883			28882	28628	28883		29754
			28883		28882					29744	28883						28883	28881			
			29370		28883						29148							28882			
																		28883			
																		28975			
																		29754			
Lineage	B.1.1.33	B.1.1.33	B.1.1.248	B.1.1.33	B.1.1.248	B.1.1	B.1.1.33	B.1.1.33	B.1.1.248	B.1.1.248	B.1.1.33	B.1.1.33	B.1.1.49	B.1.1.248	B.1.1	B.1.1.33	B.1.1.248	B.1.1.248	B.1.1.248	B.1.1.33	B.1.1
# SNPs	10	14	16	12	16	1	11	13	12	15	16	13	13	14	6	15	15	19	14	10	14

Low depth SNPs (DP<10)	241 (9)<br>3037 (3)<br>10302 (1)<br>13804 (3)<br>15237 (4)<br>19255 (4)<br>23403 (3)<br>28881 (9)<br>28882 (9)<br>29148 (4)			27299 (6)	2488 (1)<br>3037 (1)<br>3334 (2)<br>5835 (2)<br>6286 (5)<br>13560 (3)<br>20511 (2)<br>21396 (2)<br>26645 (4)<br>28093 (5)<br>29148 (5)			100 (7)<br>3037 (7)<br>10667 (2)<br>11074 (3)<br>11824 (7)<br>12053 (9)<br>25088 (9)
Lineage considering low depth SNPs	B.1.1			B.1.1.33	B.1.1			B.1.1.28
