# Supplementary material for: Genomic epidemiology of SARS-CoV-2 in Esteio, Rio Grande do Sul, Brazil
Source: BMC Genomics. 2021 May 20;22:371. doi: 10.1186/s12864-021-07708-w (PMC8136996; doi:10.1186/s12864-021-07708-w)
Supplement: Supplementary file 3 — Additional file 3. [file 12864_2021_7708_MOESM3_ESM.pdf]

We gratefully acknowledge the following Authors from the Originating laboratories responsible for obtaining the specimens and the Submitting laboratories where genetic sequence data were generated and shared via the GISAID Initiative, on which this research is based.

Accession No.	Collected	Originating Lab	Submitting Lab	Authors
EPI_ISL_402125	2019-12-26	National Institute for Communicable Disease Control and Prevention (ICDC) Chinese Center for Disease Control and Prevention (China CDC)	National Institute for Communicable Disease Control and Prevention (ICDC) Chinese Center for Disease Control and Prevention (China CDC)	Zhang et al
EPI_ISL_406798	2019-12-26	General Hospital of Central Theater Command of People's Liberation Army of China	BGI & Institute of Microbiology, Chinese Academy of Sciences & Shandong First Medical University & Shandong Academy of Medical Sciences & General Hospital of Central Theater Command of People's Liberation Army of China	Weijun Chen et al
EPI_ISL_529214	2019-12-30	Beijing Institute of Microbiology and Epidemiology	Beijing Institute of Microbiology and Epidemiology	Fan et al
EPI_ISL_529216	2019-12-30	Beijing Institute of Microbiology and Epidemiology	Beijing Institute of Microbiology and Epidemiology	Fan et al
EPI_ISL_403929	2019-12-30	Institute of Pathogen Biology, Chinese Academy of Medical Sciences & Peking Union Medical College	Institute of Pathogen Biology, Chinese Academy of Medical Sciences & Peking Union Medical College	Lili Ren et al
EPI_ISL_402129	2019-12-30	Wuhan Jinyintan Hospital	Wuhan Institute of Virology, Chinese Academy of Sciences	Peng Zhou et al
EPI_ISL_403962	2020-01-08	Bamrasnaradura Hospital	1. Department of Medical Sciences, Ministry of Public Health, Thailand 2. Thai Red Cross Emerging Infectious Diseases - Health Science Centre 3. Department of Disease Control, Ministry of Public Health, Thailand	Pilailuk et al
EPI_ISL_410301	2020-01-13	National Influenza Centre, National Public Health Laboratory, Kathmandu, Nepal	The University of Hong Kong	Ranjit Sah et al
EPI_ISL_403963	2020-01-13	Bamrasnaradura Hospital	1. Department of Medical Sciences, Ministry of Public Health, Thailand 2. Thai Red Cross Emerging Infectious Diseases - Health Science Centre 3. Department of Disease Control, Ministry of Public Health, Thailand	Pilailuk et al
EPI_ISL_447910	2020-01-15	n/a	National Institute of Health. Department of medical Sciences, Ministry of Public Health, Thailand	Pilailuk et al
EPI_ISL_407313	2020-01-19	Hangzhou Center for Disease Control and Prevention	Hangzhou Center for Disease Control and Prevention	Jun Li et al
EPI_ISL_479799	2020-01-20	Sapporo City Institute of Public Health	Pathogen Genomics Center, National Institute of Infectious Diseases	Tsuyoshi Sekizuka et al
EPI_ISL_479800	2020-01-20	Sapporo City Institute of Public Health	Pathogen Genomics Center, National Institute of Infectious Diseases	Tsuyoshi Sekizuka et al
EPI_ISL_421232	2020-01-21	Hangzhou Center for Diseases Control and Prevention	Hangzhou Center for Diseases Control and Prevention	Jun Li et al
EPI_ISL_421227	2020-01-21	Hangzhou Center for Diseases Control and Prevention	Hangzhou Center for Diseases Control and Prevention	Jun Li et al
EPI_ISL_417064	2020-01-21	Prince of Wales Hospital	Hong Kong Department of Health	Alan K.L. Tsang et al
EPI_ISL_404253	2020-01-21	IL Department of Public Health Chicago Laboratory	Pathogen Discovery, Respiratory Viruses Branch, Division of Viral Diseases, Centers for Disease Control and Prevention	Ying Tao et al
EPI_ISL_408976	2020-01-22	Centre for Infectious Diseases and Microbiology Laboratory Services	NSW Health Pathology - Institute of Clinical Pathology and Medical Research; Westmead Hospital; University of Sydney	Rockett R et al
EPI_ISL_406036	2020-01-22	California Department of Public Health	Pathogen Discovery, Respiratory Viruses Branch, Division of Viral Diseases, Centers for Disease Control and Prevention	Anna Uehara et al
EPI_ISL_418269	2020-01-22	Microbiology and Immunology department, Pasteur institute in Ho Chi Minh city	Microbiology and Immunology department, Pasteur institute in Ho Chi Minh city	Cao et al
EPI_ISL_418267	2020-01-22	Microbiology and Immunology department, Pasteur institute in Ho Chi Minh city	Microbiology and Immunology department, Pasteur institute in Ho Chi Minh city	Nguyen et al
EPI_ISL_425177	2020-01-23	Public Health Ontario	Public Health Agency of Canada - National Microbiology Laboratory	Amrit S. Boese et al
EPI_ISL_413015	2020-01-23	Public Health Ontario Laboratory	National Microbiology Laboratory	Shari Tyson et al
EPI_ISL_410720	2020-01-23	Department of Infectious and Tropical Diseases, Bichat Claude Bernard Hospital, Paris	National Reference Center for Viruses of Respiratory Infections, Institut Pasteur, Paris	Mélanie Albert et al

EPI_ISL_406596	2020-01-23	Department of Infectious and Tropical Diseases, Bichat Claude Bernard Hospital, Paris	National Reference Center for Viruses of Respiratory Infections, Institut Pasteur, Paris	Mélanie Albert et al
EPI_ISL_434563	2020-01-23	unknown	Microbiology, The University of Hong Kong	To et al
EPI_ISL_410532	2020-01-23	Dept. of Pathology, National Institute of Infectious Diseases	Pathogen Genomics Center, National Institute of Infectious Diseases	Tsuyoshi Sekizuka et al
EPI_ISL_406973	2020-01-23	Singapore General Hospital	National Public Health Laboratory	Mak et al
EPI_ISL_482678	2020-01-23	Singapore General Hospital	Department of Microbiology	Nurdyana Abdul Rahman et al
EPI_ISL_407893	2020-01-24	Centre for Infectious Diseases and Microbiology Laboratory Services	NSW Health Pathology - Institute of Clinical Pathology and Medical Research; Westmead Hospital; University of Sydney	Eden J-S et al
EPI_ISL_416410	2020-01-24	Victorian Infectious Diseases Reference Laboratory (VIDRL)	Victorian Infectious Diseases Reference Laboratory and Microbiological Diagnostic Unit Public Health Laboratory, Doherty Institute	Caly L. et al
EPI_ISL_434564	2020-01-24	unknown	Microbiology, The University of Hong Kong	To et al
EPI_ISL_416829	2020-01-24	National Public Health Laboratory	Malaysia Genome Institute	Mohd Noor Mat Isa et al
EPI_ISL_416866	2020-01-24	National Public Health Laboratory	Malaysia Genome Institute	Mohd Noor Mat Isa et al
EPI_ISL_416884	2020-01-24	National Public Health Laboratory	Malaysia Genome Institute	Mohd Noor Mat Isa et al
EPI_ISL_451392	2020-01-24	West China Hospital of Sichuan University	State Key Laboratory of Biotherapy of Sichuan University	Baowen Du et al
EPI_ISL_411926	2020-01-24	Taiwan Centers for Disease Control	Taiwan Centers for Disease Control	Ji-Rong Yang et al
EPI_ISL_416427	2020-01-24	National Influenza Center, National Institute of Hygiene and Epidemiology (NIHE)	National Influenza Center, National Institute of Hygiene and Epidemiology (NIHE)	Le Quynh Mai et al
EPI_ISL_408668	2020-01-24	National Influenza Center - National Institute of Hygiene and Epidemiology (NIHE)	National Influenza Center - National Institute of Hygiene and Epidemiology (NIHE)	Ung Thi Hong Trang et al
EPI_ISL_408977	2020-01-25	Serology, Virology and OTDS Laboratories (SAViD), NSW Health Pathology Randwick	NSW Health Pathology - Institute of Clinical Pathology and Medical Research; Centre for Infectious Diseases and Microbiology Laboratory Services; Westmead Hospital; University of Sydney	Eden J-S et al
EPI_ISL_416411	2020-01-25	Victorian Infectious Diseases Reference Laboratory (VIDRL)	Victorian Infectious Diseases Reference Laboratory and Microbiological Diagnostic Unit Public Health Laboratory, Doherty Institute	Caly L. et al
EPI_ISL_407084	2020-01-25	Department of Virology III, National Institute of Infectious Diseases	Pathogen Genomics Center, National Institute of Infectious Diseases	Tsuyoshi Sekizuka et al
EPI_ISL_410531	2020-01-25	Dept. of Pathology, National Institute of Infectious Diseases	Pathogen Genomics Center, National Institute of Infectious Diseases	Tsuyoshi Sekizuka et al
EPI_ISL_407987	2020-01-25	Singapore General Hospital	Programme in Emerging Infectious Diseases, Duke-NUS Medical School	Danielle E Anderson et al
EPI_ISL_407193	2020-01-25	Korea Centers for Disease Control & Prevention (KCDC) Center for Laboratory Control of Infectious Diseases Division of Viral Diseases	Korea Centers for Disease Control & Prevention (KCDC) Center for Laboratory Control of Infectious Diseases Division of Viral Diseases	Jeong-Min Kim et al
EPI_ISL_411915	2020-01-25	Laboratory Medicine	Department of Laboratory Medicine, Lin-Kou Chang Gung Memorial Hospital, Taoyuan, Taiwan.	Kuo-Chien Tsao et al
EPI_ISL_447914	2020-01-25	n/a	National Institute of Health. Department of medical Sciences, Ministry of Public Health, Thailand	Pilailuk et al
EPI_ISL_437621	2020-01-25	unknown	Faculty of Medicine	Rodpan et al
EPI_ISL_493170	2020-01-26	National Virus Resource Center, Chinese Academy of Sciences, Wuhan 430071, China	Computational Virology Group, Center for Bacteria and Viruses Resources and Bioinformation, Wuhan Institute of Virology, Chinese Academy of Sciences，Wuhan 430071, China	Jianjun Chen et al
EPI_ISL_411902	2020-01-27	Virology Unit, Institut Pasteur du Cambodge.	Virology Unit, Institut Pasteur du Cambodge (Sequencing done by: Jessica E Manning/Jennifer A Bohl at Malaria and Vector Research Research Laboratory, National Institute of Allergy and Infectious Diseases and Vida Ahyong from Chan-Zuckerberg Biohub)	Erik A Karlsson et al

EPI_ISL_413522	2020-01-27	Indian Council of Medical Research - National Institute of Virology	National Influenza Center, Indian Council of Medical Research - National Institute of Virology	Potdar V et al
EPI_ISL_418996	2020-01-27	National Public Health Laboratory, National Centre for Infectious Diseases	National Public Health Laboratory, National Centre for Infectious Diseases	Mak TM et al
EPI_ISL_410044	2020-01-27	California Department of Public Health	Pathogen Discovery, Respiratory Viruses Branch, Division of Viral Diseases, Centers for Disease Control and Prevention	Jing Zhang et al
EPI_ISL_411219	2020-01-28	Department of Infectious and Tropical Diseases, Bichat Claude Bernard Hospital, Paris	Laboratoire Virpath, CIRI U111, UCBL1, INSERM, CNRS, ENS Lyon	Olivier Terrier et al
EPI_ISL_411220	2020-01-28	Department of Infectious and Tropical Diseases, Bichat Claude Bernard Hospital, Paris	Laboratoire Virpath, CIRI U111, UCBL1, INSERM, CNRS, ENS Lyon	Olivier Terrier et al
EPI_ISL_450200	2020-01-28	Department of Virology	Department of Virology	Boehmer et al
EPI_ISL_450198	2020-01-28	Department of Virology	Department of Virology	Boehmer et al
EPI_ISL_406862	2020-01-28	Charité Universitätsmedizin Berlin, Institute of Virology; Institut für Mikrobiologie der Bundeswehr, Munich	Charité Universitätsmedizin Berlin, Institute of Virology	Victor M Corman et al
EPI_ISL_430443	2020-01-28	Institute for Medical Research, Infectious Disease Research Centre, National Institutes of Health, Ministry of Health Malaysia	Institute for Medical Research, Infectious Disease Research Centre, National Institutes of Health, Ministry of Health Malaysia	Suppiah.J et al
EPI_ISL_411927	2020-01-28	Taiwan Centers for Disease Control	Taiwan Centers for Disease Control	Ji-Rong Yang et al
EPI_ISL_582125	2020-01-28	Sheikh Khalifa Medical City	Molecular Surveillance lab Sheikh Khalifa Medical City	Amirtharaj Francis et al
EPI_ISL_582126	2020-01-28	Sheikh Khalifa Medical City	Molecular Surveillance lab Sheikh Khalifa Medical City	Amirtharaj Francis et al
EPI_ISL_407071	2020-01-29	Respiratory Virus Unit, Microbiology Services Colindale, Public Health England	Respiratory Virus Unit, Microbiology Services Colindale, Public Health England	Monica Galiano et al
EPI_ISL_407073	2020-01-29	Respiratory Virus Unit, Microbiology Services Colindale, Public Health England	Respiratory Virus Unit, Microbiology Services Colindale, Public Health England	Monica Galiano et al
EPI_ISL_610245	2020-01-29	Virology, National Institute for Biological Standards and Control	Virology, National Institute for Biological Standards and Control	Mee et al
EPI_ISL_407079	2020-01-29	Lapland Central Hospital	Department of Virology, University of Helsinki and Helsinki University Hospital, Helsinki, Finland	Teemu Smura et al
EPI_ISL_408431	2020-01-29	Sorbonne Université, Inserm et Assistance Publique-Hôpitaux de Paris (Pitié Salpétrière)	National Reference Center for Viruses of Respiratory Infections, Institut Pasteur, Paris	Mélanie Albert et al
EPI_ISL_450199	2020-01-29	Department of Virology	Department of Virology	Boehmer et al
EPI_ISL_497827	2020-01-29	Department of Microbiology, The University of Hong Kong	Department of Microbiology, The University of Hong Kong	Kelvin K.W. To et al
EPI_ISL_412974	2020-01-29	Department of Infectious Diseases, Istituto Superiore di Sanità, Rome, Italy	Virology Laboratory, Scientific Department, Army Medical Center	Paola Stefanelli et al
EPI_ISL_410546	2020-01-29	INMI Lazzaro Spallanzani IRCCS	Laboratory of Virology, INMI Lazzaro Spallanzani IRCCS	Maria R. Capobianchi et al
EPI_ISL_410545	2020-01-29	INMI Lazzaro Spallanzani IRCCS	Laboratory of Virology, INMI Lazzaro Spallanzani IRCCS	Maria R. Capobianchi et al
EPI_ISL_418994	2020-01-29	National Public Health Laboratory, National Centre for Infectious Diseases	National Public Health Laboratory, National Centre for Infectious Diseases	Mak TM et al
EPI_ISL_408010	2020-01-29	California Department of Health	Pathogen Discovery, Respiratory Viruses Branch, Division of Viral Diseases, Centers for Disease Control and Prevention	Ying Tao et al
EPI_ISL_435137	2020-01-29	Mohammed Bin Rashid University of Medicine and Health Sciences	Al Jalila Genomics Center	Ahmad Abou Tayoun et al
EPI_ISL_450209	2020-01-30	Department of Virology	Department of Virology	Boehmer et al
EPI_ISL_450409	2020-01-30	unknown	Microbiology	To et al
EPI_ISL_451302	2020-01-30	Laboratory of Virology, INMI Lazzaro Spallanzani IRCCS	Laboratory of Virology, INMI Lazzaro Spallanzani IRCCS	Cesare E.M. Gruber et al
EPI_ISL_416885	2020-01-30	National Public Health Laboratory	Malaysia Genome Institute	Mohd Noor Mat Isa et al
EPI_ISL_412870	2020-01-30	Division of Viral Diseases, Center for Laboratory Control of Infectious Diseases, Korea Centers for Diseases Control and Prevention	Division of Viral Diseases, Center for Laboratory Control of Infectious Diseases, Korea Centers for Diseases Control and Prevention	Jeong-Min Kim et al
EPI_ISL_425118	2020-01-30	Division of Viral Diseases, Center for Laboratory Control of Infectious Diseases, Korea Centers for Diseases Control and Prevention	Division of Viral Diseases, Center for Laboratory Control of Infectious Diseases, Korea Centers for Diseases Control and Prevention	Jeong-Min Kim et al

EPI_ISL_582608	2020-01-30	Sheikh Khalifa Medical City	Molecular/Surveillance lab Sheikh Khalifa Medical City	Amirtharaj Francis et al
EPI_ISL_413523	2020-01-31	Indian Council of Medical Research-National Institute of Virology	National Influenza Center, Indian Council of Medical Research-National Institute of Virology	Potdar V et al
EPI_ISL_412871	2020-01-31	Division of Viral Diseases, Center for Laboratory Control of Infectious Diseases, Korea Centers for Diseases Control and Prevention	Division of Viral Diseases, Center for Laboratory Control of Infectious Diseases, Korea Centers for Diseases Control and Prevention	Jeong-Min Kim et al
EPI_ISL_497963	2020-01-31	Division of Viral Diseases, Center for Laboratory Control of Infectious Diseases, Korea Centers for Diseases Control and Prevention	Division of Viral Diseases, Center for Laboratory Control of Infectious Diseases, Korea Centers for Diseases Control and Prevention	Jeong-Min Kim et al
EPI_ISL_476139	2020-01-31	Folkhalsomyndigheten	The Public Health Agency of Sweden	Oskar Karlsson Lindsjo et al
EPI_ISL_428489	2020-01-31	Centers for Disease Control, R.O.C. (Taiwan)	Centers for Disease Control, R.O.C. (Taiwan)	Ji-Rong Yang et al
EPI_ISL_428488	2020-01-31	Centers for Disease Control, R.O.C. (Taiwan)	Centers for Disease Control, R.O.C. (Taiwan)	Ji-Rong Yang et al
EPI_ISL_408670	2020-01-31	Wisconsin Department of Health Services	Pathogen Discovery, Respiratory Viruses Branch, Division of Viral Diseases, Centers for Disease Control and Prevention	Jing Zhang et al
EPI_ISL_654866	2020-01-31	Pasteur Institute in Ho Chi Minh city	Department of Microbiology and Immunology - Pasteur Institute in Ho Chi Minh city	Cao Minh Thắng et al
EPI_ISL_452360	2020-02-01	Laboratory of Infectious Diseases Center of Beijing Ditan Hospital	Laboratory of Infectious Diseases Center of Beijing Ditan Hospital	Siyuan Yang et al
EPI_ISL_412872	2020-02-01	Division of Viral Diseases, Center for Laboratory Control of Infectious Diseases, Korea Centers for Diseases Control and Prevention	Division of Viral Diseases, Center for Laboratory Control of Infectious Diseases, Korea Centers for Diseases Control and Prevention	Jeong-Min Kim et al
EPI_ISL_447918	2020-02-01	n/a	National Institute of Health. Department of medical Sciences, Ministry of Public Health, Thailand	Pilailuk et al
EPI_ISL_437624	2020-02-01	unknown	Faculty of Medicine	Rodpan et al
EPI_ISL_419734	2020-02-02	Victorian Infectious Diseases Reference Laboratory (VIDRL)	Victorian Infectious Diseases Reference Laboratory and Microbiological Diagnostic Unit Public Health Laboratory, Doherty Institute	Caly L. et al
EPI_ISL_426164	2020-02-02	Division of Viral Diseases, Center for Laboratory Control of Infectious Diseases, Korea Centers for Diseases Control and Prevention	Division of Viral Diseases, Center for Laboratory Control of Infectious Diseases, Korea Centers for Diseases Control and Prevention	Jeong-Min Kim et al
EPI_ISL_407976	2020-02-03	KU Leuven, Clinical and Epidemiological Virology	KU Leuven, Clinical and Epidemiological Virology	Bert Vanmechelen et al
EPI_ISL_417180	2020-02-03	Department of Pathology, United Christian Hospital	Department of Health Technology and Informatics, Faculty of Health and Social Science, The Hong Kong Polytechnic University	Kenneth Siu-Sing LEUNG et al
EPI_ISL_437617	2020-02-03	unknown	Faculty of Medicine	Rodpan et al
EPI_ISL_426166	2020-02-04	Division of Viral Diseases, Center for Laboratory Control of Infectious Diseases, Korea Centers for Diseases Control and Prevention	Division of Viral Diseases, Center for Laboratory Control of Infectious Diseases, Korea Centers for Diseases Control and Prevention	Jeong-Min Kim et al
EPI_ISL_417518	2020-02-04	Laboratory Medicine	Department of Laboratory Medicine, Lin-Kou Chang Gung Memorial Hospital, Taoyuan, Taiwan	Kuo-Chien Tsao et al
EPI_ISL_447919	2020-02-04	n/a	National Institute of Health. Department of medical Sciences, Ministry of Public Health, Thailand	Pilailuk et al
EPI_ISL_414040	2020-02-05	Respiratory Virus Unit, Microbiology Services Colindale, Public Health England	Respiratory Virus Unit, Microbiology Services Colindale, Public Health England	Monica Galiano et al
EPI_ISL_426169	2020-02-05	Division of Viral Diseases, Center for Laboratory Control of Infectious Diseases, Korea Centers for Diseases Control and Prevention	Division of Viral Diseases, Center for Laboratory Control of Infectious Diseases, Korea Centers for Diseases Control and Prevention	Jeong-Min Kim et al
EPI_ISL_410218	2020-02-05	Department of Laboratory Medicine, National Taiwan University Hospital	Microbial Genomics Core Lab, National Taiwan University Centers of Genomic and Precision Medicine	Shiou-Hwei Yeh et al
EPI_ISL_416907	2020-02-06	National Public Health Laboratory	Malaysia Genome Institute	Mohd Noor Mat Isa et al
EPI_ISL_422428	2020-02-06	National Public Health Laboratory, National Centre for Infectious Diseases	National Public Health Laboratory, National Centre for Infectious Diseases	Mak TM et al
EPI_ISL_457726	2020-02-06	TSGH-CP molecular lab	TSGH-CP molecular lab	Cherng-Lih Perng et al
EPI_ISL_457730	2020-02-06	TSGH-CP molecular lab	TSGH-CP molecular lab	Cherng-Lih Perng et al

EPI_ISL_414043	2020-02-07	Respiratory Virus Unit, Microbiology Services Colindale, Public Health England	Respiratory Virus Unit, Microbiology Services Colindale, Public Health England	Monica Galiano et al
EPI_ISL_648043	2020-02-07	Department of Laboratory Medicine, Tan Tock Seng Hospital	Department of Laboratory Medicine, Tan Tock Seng Hospital	Chen YYC et al
EPI_ISL_437616	2020-02-07	unknown	Faculty of Medicine	Rodpan et al
EPI_ISL_582609	2020-02-07	Sheikh Khalifa Medical City	Molecular/Surveillance lab Sheikh Khalifa Medical City	Amirtharaj Francis et al
EPI_ISL_414041	2020-02-08	Respiratory Virus Unit, Microbiology Services Colindale, Public Health England	Respiratory Virus Unit, Microbiology Services Colindale, Public Health England	Monica Galiano et al
EPI_ISL_414042	2020-02-08	Respiratory Virus Unit, Microbiology Services Colindale, Public Health England	Respiratory Virus Unit, Microbiology Services Colindale, Public Health England	Monica Galiano et al
EPI_ISL_414044	2020-02-08	Respiratory Virus Unit, Microbiology Services Colindale, Public Health England	Respiratory Virus Unit, Microbiology Services Colindale, Public Health England	Monica Galiano et al
EPI_ISL_410486	2020-02-08	CNR Virus des Infections Respiratoires - France SUD	CNR Virus des Infections Respiratoires - France SUD	Bal et al
EPI_ISL_648071	2020-02-08	Department of Laboratory Medicine, Tan Tock Seng Hospital	Department of Laboratory Medicine, Tan Tock Seng Hospital	Chen YYC et al
EPI_ISL_457733	2020-02-08	TSGH-CP molecular lab	TSGH-CP molecular lab	Cherng-Lih Perng et al
EPI_ISL_435134	2020-02-08	Mohammed Bin Rashid University of Medicine and Health Sciences	Al Jalila Genomics Center	Ahmad Abou Tayoun et al
EPI_ISL_417185	2020-02-09	Department of Pathology, United Christian Hospital	Department of Health Technology and Informatics, Faculty of Health and Social Science, The Hong Kong Polytechnic University	Kenneth Siu-Sing LEUNG et al
EPI_ISL_416404	2020-02-09	Shanghai Public Health Clinical Center, Shanghai Medical College, Fudan University	National Research Center for Translational Medicine (Shanghai), Ruijin Hospital affiliated to Shanghai Jiao Tong University School of Medicine & Shanghai Public Health Clinical Center	Shengyue Wang et al
EPI_ISL_462293	2020-02-09	National Public Health Laboratory, National Centre for Infectious Diseases	National Public Health Laboratory, National Centre for Infectious Diseases	Mak TM et al
EPI_ISL_410537	2020-02-09	Singapore General Hospital, Molecular Laboratory, Division of Pathology	Programme in Emerging Infectious Diseases, Duke-NUS Medical School	Danielle E Anderson et al
EPI_ISL_451388	2020-02-10	West China Hospital of Sichuan University	State Key Laboratory of Biotherapy of Sichuan University	Baowen Du et al
EPI_ISL_411955	2020-02-10	California Department of Public Health	Pathogen Discovery, Respiratory Viruses Branch, Division of Viral Diseases, Centers for Disease Control and Prevention	Krista Queen et al
EPI_ISL_416429	2020-02-11	National Influenza Center, National Institute of Hygiene and Epidemiology (NIHE)	National Influenza Center, National Institute of Hygiene and Epidemiology (NIHE)	Le Quynh Mai et al
EPI_ISL_430444	2020-02-12	Institute for Medical Research, Infectious Disease Research Centre, National Institutes of Health, Ministry of Health Malaysia	Institute for Medical Research, Infectious Disease Research Centre, National Institutes of Health, Ministry of Health Malaysia	Suppiah.J et al
EPI_ISL_419227	2020-02-15	Department of Clinical Pathology, Pamela Youde Nethersole Eastern Hospital	Department of Health Technology and Informatics, Faculty of Health and Social Science, The Hong Kong Polytechnic University	Kenneth Siu-Sing LEUNG et al
EPI_ISL_416575	2020-02-15	Japanese Quarantine Stations	Pathogen Genomics Center, National Institute of Infectious Diseases	Tsuyoshi Sekizuka et al
EPI_ISL_416577	2020-02-15	Japanese Quarantine Stations	Pathogen Genomics Center, National Institute of Infectious Diseases	Tsuyoshi Sekizuka et al
EPI_ISL_496615	2020-02-15	Gorgas Memorial Laboratory of Health Studies	Gorgas Memorial Laboratory of Health Studies	Danilo Franco et al
EPI_ISL_412965	2020-02-16	BCCDC Public Health Laboratory	BCCDC Public Health Laboratory	Harrigan et al
EPI_ISL_419228	2020-02-17	Department of Clinical Pathology, Pamela Youde Nethersole Eastern Hospital	Department of Health Technology and Informatics, Faculty of Health and Social Science, The Hong Kong Polytechnic University	Kenneth Siu-Sing LEUNG et al
EPI_ISL_416632	2020-02-17	Japanese Quarantine Stations	Pathogen Genomics Center, National Institute of Infectious Diseases	Tsuyoshi Sekizuka et al
EPI_ISL_496609	2020-02-17	Gorgas Memorial Laboratory of Health Studies	Gorgas Memorial Laboratory of Health Studies	Danilo Franco et al
EPI_ISL_490006	2020-02-18	King Fahad Medical City	King Fahad Medical City	Alosaimi et al
EPI_ISL_415577	2020-02-20	BCCDC Public Health Laboratory	BCCDC Public Health Laboratory	Harrigan et al
EPI_ISL_605797	2020-02-20	National Virus Reference Laboratory	Irish Coronavirus Sequencing Consortium - Helixworks	Sachin Chalapati et al
EPI_ISL_412973	2020-02-20	Department of Infectious Diseases, Istituto Superiore di Sanità, Roma , Italy	Virology Laboratory, Scientific Department, Army Medical Center	Paola Stefanelli et al

EPI_ISL_490007	2020-02-20	King Fahad Medical City	King Fahad Medical City	Alosaimi et al
EPI_ISL_451306	2020-02-21	Molecular Virology Unit, Fondazione IRCCS Policlinico San Matteo , Pavia	Laboratory of Virology, INMI Lazzaro Spallanzani IRCCS	Antonio Piralla et al
EPI_ISL_727998	2020-02-21	Fujita Health University Okazaki Medical Center	Department of Virology and Parasitology, Fujita Health University School of Medicine	Takayuki Murata et al
EPI_ISL_454420	2020-02-21	Rafik Hariri University Hospital	Rafik Hariri University Hospital	Rita Feghali et al
EPI_ISL_460091	2020-02-22	Molecular Virology Unit, Fondazione IRCCS Policlinico San Matteo , Pavia	Laboratory of Virology, INMI Lazzaro Spallanzani IRCCS	Antonino Di Caro et al
EPI_ISL_496607	2020-02-22	Gorgas Memorial Laboratory of Health Studies	Gorgas Memorial Laboratory of Health Studies	Danilo Franco et al
EPI_ISL_582620	2020-02-22	Sheikh Khalifa Medical City	Molecular/Surveillance lab Sheikh Khalifa Medical City	Amirtharaj Francis et al
EPI_ISL_419242	2020-02-23	Department of Clinical Pathology, Tuen Mun Hospital	Department of Health Technology and Informatics, Faculty of Health and Social Science, The Hong Kong Polytechnic University	Kenneth Siu-Sing LEUNG et al
EPI_ISL_419211	2020-02-23	Central Virology Laboratory	Israel Institute for Biological Research	Inbar Cohen-Gihon et al
EPI_ISL_457701	2020-02-23	Oman-NIC	Oman-NIC	Samira Al-Maruqi et al
EPI_ISL_490008	2020-02-23	King Fahad Medical City	King Fahad Medical City	Alosaimi et al
EPI_ISL_425117	2020-02-23	Division of Viral Diseases, Center for Laboratory Control of Infectious Diseases, Korea Centers for Diseases Control and Prevention	Division of Viral Diseases, Center for Laboratory Control of Infectious Diseases, Korea Centers for Diseases Control and Prevention	Jeong-Min Kim et al
EPI_ISL_412862	2020-02-23	California Department of Public Health	Pathogen Discovery, Respiratory Viruses Branch, Division of Viral Diseases, Centers for Disease Control and Prevention	Krista Queen et al
EPI_ISL_437932	2020-02-24	Institut für Virologie am Department für Hygiene, Mikrobiologie und Public Health	Bergthaler laboratory, CeMM Research Center for Molecular Medicine of the Austrian Academy of Sciences	Alexandra Popa et al
EPI_ISL_417447	2020-02-24	Laboratory of Infectious Diseases, Department of Biomedical and Clinical Sciences L. Sacco, University of Milan	Laboratory of Infectious Diseases, Department of Biomedical and Clinical Sciences L. Sacco, University of Milan	Gianguglielmo Zehender et al
EPI_ISL_457704	2020-02-24	Oman-NIC	Oman-NIC	Samira Al-Maruqi et al
EPI_ISL_413996	2020-02-24	Laboratoire de Virologie, HUG	Swiss National Reference Centre for Influenza	LAUBSCHER Florian et al. et al
EPI_ISL_412964	2020-02-25	Hospital Israelita Albert Einstein	Instituto Adolfo Lutz Interdisciplinary Procedures Center Strategic Laboratory	Jaqueline Goes de Jesus et al
EPI_ISL_412971	2020-02-25	HUS Diagnostiikkakeskus, Hallinto	Department of Virology Faculty of Medicine, Medicum University of Helsinki	Teemu Smura et al
EPI_ISL_414497	2020-02-25	Center of Medical Microbiology, Virology, and Hospital Hygiene, University of Duesseldorf	Center of Medical Microbiology, Virology, and Hospital Hygiene, University of Duesseldorf	Ortwin Adams et al
EPI_ISL_501220	2020-02-25	Department of Medical Microbiology, University Malaya Medical Centre	Department of Medical Microbiology, Faculty of Medicine, University of Malaya	Yoong Min CHONG et al
EPI_ISL_435131	2020-02-25	Mohammed Bin Rashid University of Medicine and Health Sciences	Al Jalila Genomics Center	Ahmad Abou Tayoun et al
EPI_ISL_419655	2020-02-26	Center for Virology, Medical University of Vienna	Bergthaler laboratory, CeMM Research Center for Molecular Medicine of the Austrian Academy of Sciences	Alexandra Popa et al
EPI_ISL_419656	2020-02-26	Center for Virology, Medical University of Vienna	Bergthaler laboratory, CeMM Research Center for Molecular Medicine of the Austrian Academy of Sciences	Alexandra Popa et al
EPI_ISL_450747	2020-02-26	Sunnybrook Health Sciences Centre	Department of Laboratory Medicine and Molecular Diagnostics, Sunnybrook Health Sciences Centre	Jalees A. Nasir et al
EPI_ISL_416142	2020-02-26	Department of Virus and Microbiological Special diagnostics, Statens Serum Institut, Copenhagen, Denmark.	Statens Serum Institute	Morten Rasmussen et al
EPI_ISL_414624	2020-02-26	Centre Hositalier Universitaire de Rouen Laboratoire de Virologie	National Reference Center for Viruses of Respiratory Infections, Institut Pasteur, Paris	Mélnie Albert et al
EPI_ISL_414498	2020-02-26	Center of Medical Microbiology, Virology, and Hospital Hygiene, University of Duesseldorf	Center of Medical Microbiology, Virology, and Hospital Hygiene, University of Duesseldorf	Ortwin Adams et al
EPI_ISL_414504	2020-02-26	Center of Medical Microbiology, Virology, and Hospital Hygiene, University of Duesseldorf	Center of Medical Microbiology, Virology, and Hospital Hygiene, University of Duesseldorf	Ortwin Adams et al
EPI_ISL_460089	2020-02-26	Molecular Virology Unit, Fondazione IRCCS Policlinico San Matteo , Pavia	Laboratory of Virology, INMI Lazzaro Spallanzani IRCCS	Antonino Di Caro et al

EPI_ISL_490010	2020-02-26	King Fahad Medical City	King Fahad Medical City	Alosaimi et al
EPI_ISL_418247	2020-02-26	HOSPITAL GENERAL DE SEGOVIA	Instituto de Salud Carlos III	Iglesias-Caballero et al
EPI_ISL_475544	2020-02-26	Karolinska Universitetslaboratoriet	The Public Health Agency of Sweden	Oskar Karlsson Lindsjo et al
EPI_ISL_419554	2020-02-26	California Department of Public Health	Pathogen Discovery, Respiratory Viruses Branch, Division of Viral Diseases, Centers for Disease Control and Prevention	Ying Tao et al
EPI_ISL_435580	2020-02-26	Santa Clara County Public Health Department	Chiu Laboratory, University of California, San Francisco	Xianding Deng et al
EPI_ISL_470851	2020-02-27	PathWest Laboratory Medicine WA	PathWest Laboratory Medicine WA	Chisha Sikazwe et al
EPI_ISL_437993	2020-02-27	Center for Virology, Medical University of Vienna	Bergthaler laboratory, CeMM Research Center for Molecular Medicine of the Austrian Academy of Sciences	Alexandra Popa et al
EPI_ISL_415578	2020-02-27	BCCDC Public Health Laboratory	BCCDC Public Health Laboratory	Harrigan et al
EPI_ISL_415641	2020-02-27	R. G. Lugar Center for Public Health Research, National Center for Disease Control and Public Health (NCDC) of Georgia.	R. G. Lugar Center for Public Health Research, National Center for Disease Control and Public Health (NCDC) of Georgia.	Nato Kotaria et al
EPI_ISL_414505	2020-02-27	Center of Medical Microbiology, Virology, and Hospital Hygiene, University of Duesseldorf	Center of Medical Microbiology, Virology, and Hospital Hygiene, University of Duesseldorf	Ortwin Adams et al
EPI_ISL_417765	2020-02-27	The National University Hospital of Iceland	deCODE genetics	Daniel F Gudbjartsson et al
EPI_ISL_649094	2020-02-27	Israel Central Virology laboratory	Israel Central Virology laboratory	Neta Zuckerman et al
EPI_ISL_450512	2020-02-27	Rafik Hariri University Hospital	Rafik Hariri University Hospital	Rita Feghali et al
EPI_ISL_412972	2020-02-27	Instituto Nacional de Enfermedades Respiratorias	Instituto de Diagnostico y Referencia Epidemiologicos (INDRE)	Ramirez-Gonzalez Ernesto et al
EPI_ISL_476559	2020-02-27	unknown	Laboratoire Sciences et Technologies de la Santé (STS) Institut Supérieur des Sciences de la Santé Université Hassan 1er, Settat, Morocco	Hajar Lemriss et al
EPI_ISL_547499	2020-02-27	Dutch COVID-19 response team	National Institute for Public Health and the Environment (RIVM)	Adam Meijer et al
EPI_ISL_454750	2020-02-27	Dutch COVID-19 response team	National Institute for Public Health and the Environment (RIVM)	Adam Meijer et al
EPI_ISL_413490	2020-02-27	Auckland Hospital	Institute of Environmental Science and Research (ESR)	Matt Storey et al
EPI_ISL_413550	2020-02-27	Centre for Human and Zoonotic Virology (CHAZVY), College of Medicine University of Lagos/Lagos University Teaching Hospital (LUTH), part of the Laboratory Network of the Nigeria Centre for Disease Control (NCDC)	African Centre of Excellence for Genomics of Infectious Diseases (ACEGID), Redeemer's University, Ede, Osun State, Nigeria	Oluniyi P.E. et al
EPI_ISL_417483	2020-02-27	Oslo University Hospital, Department of Medical Microbiology	Norwegian Institute of Public Health	Kathrine Stene-Johansen et al
EPI_ISL_417490	2020-02-27	Oslo University Hospital, Department of Medical Microbiology	Norwegian Institute of Public Health, Department of Virology	Kathrine Stene-Johansen et al
EPI_ISL_496617	2020-02-27	Gorgas Memorial Laboratory of Health Studies	Gorgas Memorial Laboratory of Health Studies	Danilo Franco et al
EPI_ISL_490011	2020-02-27	King Fahad Medical City	King Fahad Medical City	Alosaimi et al
EPI_ISL_530042	2020-02-27	Hospital Universitario La Paz	Hospital Universitario La Paz	María Rodríguez et al
EPI_ISL_419689	2020-02-27	Servicio de Microbiología. Consorcio Hospital General Universitario de Valencia	Sequencing and Bioinformatics Service and Molecular Epidemiology Research Group. FISABIO-Public Health	Neris Garcia-Gonzalez et al
EPI_ISL_416484	2020-02-27	Servicio de Microbiología. Consorcio Hospital General Universitario de Valencia	Sequencing and Bioinformatics Service and Molecular Epidemiology Research Group. FISABIO-Public Health	Maria Dolores Ocete et al
EPI_ISL_430847	2020-02-27	HS mikrobiologi virus	The Public Health Agency of Sweden	Zhibing Yun et al
EPI_ISL_413561	2020-02-27	California Department of Public Health	Chiu Laboratory, University of California, San Francisco	Xianding Deng et al
EPI_ISL_413594	2020-02-28	Centre for Infectious Diseases and Microbiology Laboratory Services	NSW Health Pathology - Institute of Clinical Pathology and Medical Research; Westmead Hospital; University of Sydney	Rockett R et al
EPI_ISL_413596	2020-02-28	Centre for Infectious Diseases and Microbiology - Public Health	NSW Health Pathology - Institute of Clinical Pathology and Medical Research; Westmead Hospital; University of Sydney	Rockett R et al
EPI_ISL_427643	2020-02-28	Centre for Infectious Diseases and Microbiology Public Health	NSW Health Pathology - Institute of Clinical Pathology and Medical Research; Westmead Hospital; University of Sydney	Timms V et al
EPI_ISL_437994	2020-02-28	Center for Virology, Medical University of Vienna	Bergthaler laboratory, CeMM Research Center for Molecular Medicine of the Austrian Academy of Sciences	Alexandra Popa et al
EPI_ISL_413016	2020-02-28	Hospital Israelita Albert Einstein	Instituto Adolfo Lutz, Interdisciplinary Procedures Center, Strategic Laboratory	Jaqueline Goes de Jesus et al

EPI_ISL_415580	2020-02-28	BCCDC Public Health Laboratory	BCCDC Public Health Laboratory	Harrigan et al
EPI_ISL_416143	2020-02-28	Department of Virus and Microbiological Special diagnostics, Statens Serum Institut, Copenhagen, Denmark.	ViFU	Morten Rasmussen et al
EPI_ISL_415644	2020-02-28	R. G. Lugar Center for Public Health Research, National Center for Disease Control and Public Health (NCDC) of Georgia.	R. G. Lugar Center for Public Health Research, National Center for Disease Control and Public Health (NCDC) of Georgia.	Nato Kotaria et al
EPI_ISL_729478	2020-02-28	Charité Universitätsmedizin Berlin, Institut für Virologie/Labor Berlin	Charité Universitätsmedizin Berlin, Institut für Virologie	Victor M Corman et al
EPI_ISL_728208	2020-02-28	Fujita Health University Okazaki Medical Center	Fujita health University, Department of Microbiology	Takayuki Murata et al
EPI_ISL_452139	2020-02-28	Instituto de Diagnostico y Referencia Epidemiologicos (INDRE)	Instituto de diagnóstico y Referencia Epidemiologicos (INDRE)	Ramirez-Gonzalez Ernesto et al
EPI_ISL_413570	2020-02-28	RIVM	Erasmus Medical Center	David Nieuwenhuijse et al
EPI_ISL_454753	2020-02-28	Dutch COVID-19 response team	National Institute for Public Health and the Environment (RIVM)	Adam Meijer et al
EPI_ISL_420135	2020-02-28	Oslo University Hospital, Department of Medical Microbiology	Norwegian Institute of Public Health, Department of Virology	Kathrine Stene-Johansen et al
EPI_ISL_496605	2020-02-28	Gorgas Memorial Laboratory of Health Studies	Gorgas Memorial Laboratory of Health Studies	Danilo Franco et al
EPI_ISL_613446	2020-02-28	Institut Pasteur de la Guadeloupe	Institut Pasteur de la Guadeloupe	Marion Barbet et al
EPI_ISL_418206	2020-02-28	Institut Pasteur Dakar	Institut Pasteur de Dakar	Ndongo Dia et al
EPI_ISL_418243	2020-02-28	HOSPITAL UNIVERSITARIO VIRGEN DE LAS NIEVES	Instituto de Salud Carlos III	Iglesias-Caballero et al
EPI_ISL_475545	2020-02-28	Karolinska Universitetslaboratoriet	The Public Health Agency of Sweden	Oskar Karlsson Lindsjo et al
EPI_ISL_475546	2020-02-28	Karolinska Universitetslaboratoriet	The Public Health Agency of Sweden	Oskar Karlsson Lindsjo et al
EPI_ISL_475547	2020-02-28	Karolinska Universitetslaboratoriet	The Public Health Agency of Sweden	Oskar Karlsson Lindsjo et al
EPI_ISL_415700	2020-02-28	University Hospitals of Geneva Laboratory of Virology	University Hospitals of Geneva Laboratory of Virology	Laubscher F. et al
EPI_ISL_415454	2020-02-28	University Hospitals of Geneva Laboratory of Virology	University Hospitals of Geneva Laboratory of Virology	Laubscher F. et al
EPI_ISL_582626	2020-02-28	Sheikh Khalifa Medical City	Molecular/Surveillance lab Sheikh Khalifa Medical City	Amirtharaj Francis et al
EPI_ISL_454928	2020-02-28	Wuhan Chain Medical Labs (CMLabs)	State Key Laboratory of Biotherapy of Sichuan University	Baowen Du et al
EPI_ISL_452332	2020-02-29	Laboratory of Infectious Diseases Center of Beijing Ditan Hospital	Laboratory of Infectious Diseases Center of Beijing Ditan Hospital	Siyuan Yang et al
EPI_ISL_415159	2020-02-29	KU Leuven, Clinical and Epidemiological Virology	KU Leuven, Clinical and Epidemiological Virology	Bert Vanmechelen et al
EPI_ISL_415128	2020-02-29	LACEN/ES - Laboratório Central de Saúde Pública do Espírito Santo	Instituto Oswaldo Cruz FIOCRUZ - Laboratory of Respiratory Viruses and Measles (LVRS)	Paola Resende et al
EPI_ISL_414016	2020-02-29	Hospital São Joaquim Beneficencia Portuguesa	Instituto Adolfo Lutz, Interdiciplinary Procedures Center, Strategic Laboratory	Claudio Tavares Sacchi et al
EPI_ISL_414015	2020-02-29	Hospital São Joaquim Beneficencia Portuguesa	Instituto Adolfo Lutz, Interdiciplinary Procedures Center, Strategic Laboratory	Claudio Tavares Sacchi et al
EPI_ISL_416742	2020-02-29	NRL for Influenza, Centrum Epidemiology and Microbiology of National Institute of Public Health, Czech Republic	Charite Universitaetsmedizin Berlin, Institute of Virology	Victor M Corman et al
EPI_ISL_629083	2020-02-29	Laboratoire du Centre Hospitalier Annecy Genevois	CNR Virus des Infections Respiratoires - France SUD	Antonin Bal et al
EPI_ISL_629085	2020-02-29	Laboratoire du Centre Hospitalier Annecy Genevois	CNR Virus des Infections Respiratoires - France SUD	Antonin Bal et al
EPI_ISL_693388	2020-02-29	CHU de Nice - Hôpital Archet 2	CNR Virus des Infections Respiratoires - France SUD	Antonin Bal et al
EPI_ISL_430469	2020-02-29	Hellenic Pasteur Institute, Public Health Laboratories	Hellenic Pasteur Institute, Public Health Laboratories, Unit of Bioinformatics and Applied Genomics	Vasiliki Pogka et al
EPI_ISL_419562	2020-02-29	Laboratoire National de Santé, Microbiology, Virology	Laboratoire National de Santé, Microbiology, Epidemiology and Microbial Genomics	Anke Wienecke-Baldacchino et al
EPI_ISL_413593	2020-02-29	Laboratoire National de Santé	Erasmus Medical Center	David Nieuwenhuijse et al
EPI_ISL_430441	2020-02-29	Institute for Medical Research, Infectious Disease Research Centre, National Institutes of Health, Ministry of Health Malaysia	Institute for Medical Research, Infectious Disease Research Centre, National Institutes of Health, Ministry of Health Malaysia	Suppiah.J et al
EPI_ISL_430442	2020-02-29	Institute for Medical Research, Infectious Disease Research Centre, National Institutes of Health, Ministry of Health Malaysia	Institute for Medical Research, Infectious Disease Research Centre, National Institutes of Health, Ministry of Health Malaysia	Suppiah.J et al
EPI_ISL_413574	2020-02-29	MHC West-Brabant	Erasmus Medical Center	David Nieuwenhuijse et al
EPI_ISL_417488	2020-02-29	Oslo University Hospital, Department of Medical Microbiology	Norwegian Institute of Public Health, Department of Virology	Kathrine Stene-Johansen et al

EPI_ISL_417489	2020-02-29	Oslo University Hospital, Department of Medical Microbiology	Norwegian Institute of Public Health, Department of Virology	Kathrine Stene-Johansen et al
EPI_ISL_415457	2020-02-29	University Hospitals of Geneva Laboratory of Virology	University Hospitals of Geneva Laboratory of Virology	Laubscher F. et al
EPI_ISL_413022	2020-02-29	Division of Infectious Diseases, University Hospital Zurich	Institute of Medical Virology, University of Zurich	Stefan Schmutz et al
EPI_ISL_430793	2020-03-01	Laboratorio Análisis Clínicos, Unidad de Servicios Diagnósticos, Swiss Medical Group	Área de Secuenciación del Laboratorio de Virología del Hospital de Niños	Nabaes Jodar et al
EPI_ISL_509712	2020-03-01	Belize Ministry of Health	Pathogen Discovery, Respiratory Viruses Branch, Division of Viral Diseases, Centers for Disease Control and Prevention	Jing Zhang et al
EPI_ISL_454918	2020-03-01	Wuhan Chain Medical Labs (CMLabs)	State Key Laboratory of Biotherapy of Sichuan University	Baowen Du et al
EPI_ISL_647977	2020-03-01	National Microbiology Reference Laboratory	Quadram Institute Bioscience	Thanh Le Viet et al
EPI_ISL_647978	2020-03-01	National Microbiology Reference Laboratory	Quadram Institute Bioscience	Thanh Le Viet et al
EPI_ISL_418241	2020-03-02	NIC Viral Respiratory Unit - Institut Pasteur of Algeria	National Reference Center for Viruses of Respiratory Infections, Institut Pasteur, Paris	Mélanie Albert et al
EPI_ISL_420037	2020-03-02	NIC Viral Respiratory Unit - Institut Pasteur of Algeria	National Reference Center for Viruses of Respiratory Infections, Institut Pasteur, Paris	Mélanie Albert et al
EPI_ISL_416153	2020-03-02	Department of Virus and Microbiological Special diagnostics, Statens Serum Institut, Copenhagen, Denmark.	ViFU	Morten Rasmussen et al
EPI_ISL_596453	2020-03-02	Booali laboratory, Qom, Iran. Department of Virology, School of Public Health, Tehran University of Medical Sciences, Tehran, Iran.	Genetics Research Center, University of Social Welfare and Rehabilitation Sciences	Zohreh Fattahi et al
EPI_ISL_649064	2020-03-02	Israel Central Virology laboratory	Israel Central Virology laboratory	Neta Zuckerman et al
EPI_ISL_416542	2020-03-02	Dasman Diabetes Institute	Dasman Diabetes Institute	Fahd Al-Mulla et al
EPI_ISL_707791	2020-03-02	1-Laboratory of Microbiology, National Reference Lab, Charles Nicolle Hospital; 2-University of Tunis ElManar, Faculty of Medicine of Tunis, LR99ES09, Tunis, Tunisia	1-Clinical and Experimental Pharmacology Lab, LR16SP02, National Center of Pharmacovigilance, University of Tunis El Manar, Tunis, Tunisia. 2-Neurodegenerative diseases and psychiatric troubles, LR18SP03, Razi Hospital, University of Tunis El Manar, Tunis, Tunisia. 3- Ministry of Health, National Observatory of New and Emerging Diseases, 1006, Tunis, Tunisia	Ilhem Boutiba-Ben Boubaker et al
EPI_ISL_454904	2020-03-02	Wuhan Chain Medical Labs (CMLabs)	State Key Laboratory of Biotherapy of Sichuan University	Baowen Du et al
EPI_ISL_416141	2020-03-03	Department of Virus and Microbiological Special diagnostics, Statens Serum Institut, Copenhagen, Denmark.	Statens Serum Institute	Morten Rasmussen et al
EPI_ISL_414587	2020-03-03	UCD National Virus Reference Laboratory	UCD National Virus Reference Laboratory	Michael Carr et al
EPI_ISL_459965	2020-03-03	Institut Pasteur du Maroc	Institut Pasteur du Maroc	Marion Barbet et al
EPI_ISL_414437	2020-03-03	Dutch COVID-19 response team	Erasmus Medical Center	David Nieuwenhuijse et al
EPI_ISL_434697	2020-03-03	unknown	National Institute of Health. Department of medical Sciences, Ministry of Public Health, Thailand	Pilailuk et al
EPI_ISL_452351	2020-03-04	Laboratory of Infectious Diseases Center of Beijing Ditan Hospital	Laboratory of Infectious Diseases Center of Beijing Ditan Hospital	Siyuan Yang et al
EPI_ISL_417422	2020-03-04	KU Leuven, Clinical and Epidemiological Virology	KU Leuven, Clinical and Epidemiological Virology	Joan Marti-Carerras et al
EPI_ISL_415105	2020-03-04	Laboratório Central de Saúde Pública Professor Gonçalo Moniz – LACEN/BA	Instituto Oswaldo Cruz FIOCRUZ - Laboratory of Respiratory Viruses and Measles (LVRS)	Paola Resende et al
EPI_ISL_414045	2020-03-04	LACEN RJ - Laboratório Central de Saúde Pública Noel Nutels	Instituto Oswaldo Cruz FIOCRUZ - Laboratory of Respiratory Viruses and Measles (LVRS)	Paola Resende et al
EPI_ISL_416032	2020-03-04	National Influenza Center - Instituto Adolfo Lutz	Instituto Adolfo Lutz, Interdiciplinary Procedures Center, Strategic Laboratory	Claudio Tavares Sacchi et al
EPI_ISL_523812	2020-03-04	Universidad Iberoamericana, Instituto de Medicina Tropical & Salud Global	International Centre for Genetic Engineering and Biotechnology (ICGEB) and ARGO Open Lab Platform	Robert Paulino-Ramirez et al
EPI_ISL_414646	2020-03-04	Department of Virology and Immunology, University of Helsinki and Helsinki University Hospital, Huslab Finland	Department of Virology, Faculty of Medicine, University of Helsinki, Helsinki, Finland	Teemu Smura et al
EPI_ISL_416494	2020-03-04	Centre Hositalier Universitaire de Rouen Laboratoire de Virologie	National Reference Center for Viruses of Respiratory Infections, Institut Pasteur, Paris	Mélnie Albert et al
EPI_ISL_729481	2020-03-04	Charité Universitätsmedizin Berlin, Institut für Virologie/Labor Berlin	Charité Universitätsmedizin Berlin, Institut für Virologie	Victor M Corman et al

EPI_ISL_414487	2020-03-04	UCD National Virus Reference Laboratory	UCD National Virus Reference Laboratory	Michael Carr et al
EPI_ISL_649099	2020-03-04	Israel Central Virology laboratory	Israel Central Virology laboratory	Neta Zuckerman et al
EPI_ISL_450508	2020-03-04	Rafik Hariri University Hospital	Rafik Hariri University Hospital	Rita Feghali et al
EPI_ISL_450511	2020-03-04	Rafik Hariri University Hospital	Rafik Hariri University Hospital	Rita Feghali et al
EPI_ISL_501181	2020-03-04	Department of Medical Microbiology, University Malaya Medical Centre	Department of Medical Microbiology, Faculty of Medicine, University of Malaya	Yoong Min CHONG et al
EPI_ISL_424667	2020-03-04	Laboratorio Estatal de Salud Publica del Estado de México	Instituto de Diagnóstico y Referencia Epidemiológicos	Irma López Martínez et al
EPI_ISL_420136	2020-03-04	Akershus University Hospital, Department for Microbiology and Infectious Disease Control	Norwegian Institute of Public Health, Department of Virology	Kathrine Stene-Johansen et al
EPI_ISL_417444	2020-03-04	Department of Healthcare Biotechnology, National University of Sciences and Technology (NUST)	Department of Healthcare Biotechnology, National University of Sciences and Technology (NUST)	Javed et al
EPI_ISL_596502	2020-03-04	Palestinian Ministry of Health	Molecular Genetics Lab	Nouar Qutob et al
EPI_ISL_454871	2020-03-04	Karolinska Universitetslaboratoriet	The Public Health Agency of Sweden	Anna-Malin Linde et al
EPI_ISL_420367	2020-03-05	KU Leuven, Clinical and Epidemiological Virology	KU Leuven, Clinical and Epidemiological Virology	Joan Marti-Carreras et al
EPI_ISL_418797	2020-03-05	KU Leuven, Clinical and Epidemiological Virology	KU Leuven, Clinical and Epidemiological Virology	Bert Vanmechelen et al
EPI_ISL_416743	2020-03-05	NRL for Influenza, Centrum Epidemiology and Microbiology of National Institute of Public Health, Czech Republic	Charite Universitaetsmedizin Berlin, Institute of Virology	Victor M Corman et al
EPI_ISL_417851	2020-03-05	The National University Hospital of Iceland	deCODE genetics	Daniel F Gudbjartsson et al
EPI_ISL_430440	2020-03-05	Institute for Medical Research, Infectious Disease Research Centre, National Institutes of Health, Ministry of Health Malaysia	Institute for Medical Research, Infectious Disease Research Centre, National Institutes of Health, Ministry of Health Malaysia	Suppiah.J et al
EPI_ISL_648072	2020-03-05	Department of Laboratory Medicine, Tan Tock Seng Hospital	Department of Laboratory Medicine, Tan Tock Seng Hospital	Chen YYC et al
EPI_ISL_427293	2020-03-06	LACEN-BA - Laboratório Central de Saúde Pública Professor Gonçalo Moniz	Instituto Oswaldo Cruz FIOCRUZ - Laboratory of Respiratory Viruses and Measles (LVRS)	Paola Resende et al
EPI_ISL_415152	2020-03-06	Gorgas Memorial Institute for Health Studies	Gorgas Memorial Institute for Health Studies	Danilo Franco et al
EPI_ISL_417877	2020-03-06	Institute of Virology, Biomedical Research Center of the Slovak Academy of Sciences, Bratislava; Public Health Authority of the Slovak Republic, Bratislava	Institute of Virology, Biomedical Research Center of the Slovak Academy of Sciences, Bratislava; Comenius University Science Park, Bratislava	Monika Slávikova et al
EPI_ISL_417879	2020-03-06	Institute of Virology, Biomedical Research Center of the Slovak Academy of Sciences, Bratislava; Public Health Authority of the Slovak Republic, Bratislava	Institute of Virology, Biomedical Research Center of the Slovak Academy of Sciences, Bratislava; Comenius University Science Park, Bratislava	Monika Slávikova et al
EPI_ISL_420294	2020-03-06	Institute of Microbiology and Immunology, Faculty of Medicine, University of Ljubljana	Institute of Microbiology and Immunology, Faculty of Medicine, University of Ljubljana	Samo Zakotnik et al
EPI_ISL_430297	2020-03-06	National Institute for Communicable Diseases of the National Health Laboratory Service	National Institute for Communicable Diseases of the National Health Laboratory Service	Allam M et al
EPI_ISL_429140	2020-03-06	Klinisk mikrobiologi Orebro	The Public Health Agency of Sweden	Martin Sundqvist et al
EPI_ISL_420600	2020-03-07	Servicio Virosis Respiratorias-Departamento Virología-INEI	Instituto Nacional Enfermedades Infecciosas C.G.Malbran	Baumeister E. et al
EPI_ISL_649093	2020-03-07	Israel Central Virology laboratory	Israel Central Virology laboratory	Neta Zuckerman et al
EPI_ISL_596504	2020-03-07	Palestinian Ministry of Health	Molecular Genetics Lab	Nouar Qutob et al
EPI_ISL_496613	2020-03-07	Gorgas Memorial Laboratory of Health Studies	Gorgas Memorial Laboratory of Health Studies	Danilo Franco et al
EPI_ISL_417878	2020-03-07	Institute of Virology, Biomedical Research Center of the Slovak Academy of Sciences, Bratislava; Public Health Authority of the Slovak Republic, Bratislava	Institute of Virology, Biomedical Research Center of the Slovak Academy of Sciences, Bratislava; Comenius University Science Park, Bratislava	Monika Slávikova et al
EPI_ISL_454890	2020-03-07	Karolinska Universitetslaboratoriet	The Public Health Agency of Sweden	Anna-Malin Linde et al
EPI_ISL_416428	2020-03-07	National Influenza Center, National Institute of Hygiene and Epidemiology (NIHE)	National Influenza Center, National Institute of Hygiene and Epidemiology (NIHE)	Le Quynh Mai et al
EPI_ISL_435303	2020-03-07	National Hospital of Tropical Diseases	Oxford University Clinical Research Unit, Hanoi, Vietnam	Nguyen Thi Tam et al
EPI_ISL_418242	2020-03-08	NIC Viral Respiratory Unit - Institut Pasteur of Algeria	National Reference Center for Viruses of Respiratory Infections, Institut Pasteur, Paris	Mélanie Albert et al

EPI_ISL_596454	2020-03-08	Infectious Disease and Tropical Medicine Research Center, Resistant Tuberculosis Institute, Zahedan University of Medical Sciences, Zahedan, Iran.	Genetics Research Center, University of Social Welfare and Rehabilitation Sciences	Zohreh Fattahi et al
EPI_ISL_649073	2020-03-08	Israel Central Virology laboratory	Israel Central Virology laboratory	Neta Zuckerman et al
EPI_ISL_496625	2020-03-08	Gorgas Memorial Laboratory of Health Studies	Gorgas Memorial Laboratory of Health Studies	Danilo Franco et al
EPI_ISL_496626	2020-03-08	Gorgas Memorial Laboratory of Health Studies	Gorgas Memorial Laboratory of Health Studies	Danilo Franco et al
EPI_ISL_417880	2020-03-08	Institute of Virology, Biomedical Research Center of the Slovak Academy of Sciences, Bratislava; Public Health Authority of the Slovak Republic, Bratislava	Institute of Virology, Biomedical Research Center of the Slovak Academy of Sciences, Bratislava; Comenius University Science Park, Bratislava	Monika Slávikova et al
EPI_ISL_635209	2020-03-08	Institute of Microbiology and Immunology, Faculty of Medicine, University of Ljubljana	Institute of Microbiology and Immunology, Faculty of Medicine, University of Ljubljana	Samo Zakotnik et al
EPI_ISL_635212	2020-03-08	Institute of Microbiology and Immunology, Faculty of Medicine, University of Ljubljana	Institute of Microbiology and Immunology, Faculty of Medicine, University of Ljubljana	Samo Zakotnik et al
EPI_ISL_430862	2020-03-08	The Public Health Agency of Sweden	The Public Health Agency of Sweden	Oskar Karlsson Lindsjo et al
EPI_ISL_455895	2020-03-08	Karolinska Universitetslaboratoriet	The Public Health Agency of Sweden	Anna-Malin Linde et al
EPI_ISL_654890	2020-03-08	Pasteur Institute in Ho Chi Minh city	Department of Microbiology and Immunology - Pasteur Institute in Ho Chi Minh city	Đỗ Thái Hùng et al
EPI_ISL_729804	2020-03-09	Laboratorio Central de Saude Publica do Estado do Rio Grande do Sul (LACEN-RS)	Laboratory of Respiratory Viruses and Measles, Oswaldo Cruz Institute, FIOCRUZ	Paola Resende et al
EPI_ISL_671269	2020-03-09	Department of Virus and Microbiological Special Diagnostics, Statens Serum Institut, Copenhagen, Denmark	Albertsen Lab, Department of Chemistry and Bioscience, Aalborg University, Denmark	Danish Covid-19 Genome Consortium et al
EPI_ISL_417482	2020-03-09	Institute of Microbiology, Universidad San Francisco de Quito	Institute of Microbiology, Universidad San Francisco de Quito	Sully Márquez et al
EPI_ISL_442523	2020-03-09	Pasteur Institute of Iran	Kawsar Human Genetic Research Company	Sirous Zeinali et al
EPI_ISL_450793	2020-03-09	Jamaica Ministry of Health and Wellness	Pathogen Discovery, Respiratory Viruses Branch, Division of Viral Diseases, Centers for Disease Control and Prevention	Yan Li et al
EPI_ISL_635213	2020-03-09	Institute of Microbiology and Immunology, Faculty of Medicine, University of Ljubljana	Institute of Microbiology and Immunology, Faculty of Medicine, University of Ljubljana	Samo Zakotnik et al
EPI_ISL_435308	2020-03-09	National Hospital of Tropical Diseases	Oxford University Clinical Research Unit, Hanoi, Vietnam	Nguyen Thi Tam et al
EPI_ISL_427305	2020-03-10	LACEN-SC - Laboratorio Central de Santa Catarina	Instituto Oswaldo Cruz FIOCRUZ - Laboratory of Respiratory Viruses and Measles (LVRS)	Paola Resende et al
EPI_ISL_427306	2020-03-10	LACEN-SC - Laboratorio Central de Santa Catarina	Instituto Oswaldo Cruz FIOCRUZ - Laboratory of Respiratory Viruses and Measles (LVRS)	Paola Resende et al
EPI_ISL_480310	2020-03-10	National Reference Laboratory "Influenza and acute respiratory diseases"	NRL-HIV	Ivan Ivanov et al
EPI_ISL_547450	2020-03-10	Dutch COVID-19 response team	National Institute for Public Health and the Environment (RIVM)	Adam Meijer et al
EPI_ISL_547451	2020-03-10	Dutch COVID-19 response team	National Institute for Public Health and the Environment (RIVM)	Adam Meijer et al
EPI_ISL_547452	2020-03-10	Dutch COVID-19 response team	National Institute for Public Health and the Environment (RIVM)	Adam Meijer et al
EPI_ISL_636492	2020-03-10	Dutch COVID-19 response team	National Institute for Public Health and the Environment (RIVM)	Adam Meijer et al
EPI_ISL_636493	2020-03-10	Dutch COVID-19 response team	National Institute for Public Health and the Environment (RIVM)	Adam Meijer et al
EPI_ISL_429273	2020-03-10	Department of Clinical Microbiology, Copenhagen University Hospital, Hvidovre, Kettegaard Alle 30, 2650 Hvidovre.	Albertsen lab, Department of Chemistry and Bioscience, Aalborg University, Denmark	Rasmus Kirkegaard et al
EPI_ISL_415643	2020-03-10	R. G. Lugar Center for Public Health Research, National Center for Disease Control and Public Health (NCDC) of Georgia.	R. G. Lugar Center for Public Health Research, National Center for Disease Control and Public Health (NCDC) of Georgia.	Nato Kotaria et al
EPI_ISL_415642	2020-03-10	R. G. Lugar Center for Public Health Research, National Center for Disease Control and Public Health (NCDC) of Georgia.	R. G. Lugar Center for Public Health Research, National Center for Disease Control and Public Health (NCDC) of Georgia.	Nato Kotaria et al
EPI_ISL_418584	2020-03-10	UCD National Virus Reference Laboratory	UCD National Virus Reference Laboratory	Michael Carr et al
EPI_ISL_424670	2020-03-10	Laboratorio Estatal de Salud Publica del Estado de Queretaro	Instituto de Diagnóstico y Referencia Epidemiologicos	Gisela Barrera Badillo et al
EPI_ISL_590915	2020-03-10	Hospital of Southern Norway - Kristiansand, Department of Medical Microbiology	Norwegian Institute of Public Health, Department of Virology	Kathrine Stene-Johansen et al
EPI_ISL_418210	2020-03-10	Institut Pasteur Dakar	Institut Pasteur de Dakar	Ndongo Dia et al

EPI_ISL_428671	2020-03-10	Centre for Dengue Research	Centre for Dengue Research	Chandima Jeewandara et al
EPI_ISL_435133	2020-03-10	Mohammed Bin Rashid University of Medicine and Health Sciences	Al Jalila Genomics Center	Ahmad Abou Tayoun et al
EPI_ISL_435312	2020-03-10	National Hospital of Tropical Diseases	Oxford University Clinical Research Unit, Hanoi, Vietnam	Nguyen Thi Tam et al
EPI_ISL_483543	2020-03-11	Kingdom of Bahrein Ministry of Health	Erasmus Medical Center	Bas Oude Munnink et al
EPI_ISL_443187	2020-03-11	National Virology Reference Laboratory	National Public Health Laboratory, National Centre for Infectious Diseases	Mak Tze Minn et al
EPI_ISL_435674	2020-03-11	National Virology Reference Laboratory	National Public Health Laboratory, National Centre for Infectious Diseases	Mak Tze Minn et al
EPI_ISL_435675	2020-03-11	National Virology Reference Laboratory	National Public Health Laboratory, National Centre for Infectious Diseases	Mak Tze Minn et al
EPI_ISL_418842	2020-03-11	BCCDC Public Health Laboratory	BCCDC Public Health Laboratory	Harrigan et al
EPI_ISL_463741	2020-03-11	Department of Molecular Virology, Cyprus Institute of Neurology and Genetics	Department of Molecular Virology, Cyprus Institute of Neurology and Genetics	Jan Richter et al
EPI_ISL_452009	2020-03-11	Department of Clinical Microbiology, Copenhagen University Hospital, Hvidovre, Kettegaard Alle 30, 2650 Hvidovre.	Albertsen lab, Department of Chemistry and Bioscience, Aalborg University, Denmark	Rasmus Kirkegaard et al
EPI_ISL_416480	2020-03-11	R. G. Lugar Center for Public Health Research, National Center for Disease Control and Public Health (NCDC) of Georgia.	R. G. Lugar Center for Public Health Research, National Center for Disease Control and Public Health (NCDC) of Georgia.	Ann Machablishvili et al
EPI_ISL_437908	2020-03-11	Laboratory of Microbiology, Medical School, National and Kapodistrian University of Athens	Laboratory of Biology, Department of Medicine, Democritus University of Thrace	Kassela K. et al
EPI_ISL_431102	2020-03-11	Department of MicroBiology,Gandhi Medical College and Hospital,Secendrabad,Hyderabad,India	Department of Microbiology, Gandhi Medical College and Hospital, Secendrabad, Hyderabad	Nagamani K et al
EPI_ISL_450792	2020-03-11	Jamaica Ministry of Health and Wellness	Pathogen Discovery, Respiratory Viruses Branch, Division of Viral Diseases, Centers for Disease Control and Prevention	Krista Queen et al
EPI_ISL_450794	2020-03-11	Jamaica Ministry of Health and Wellness	Pathogen Discovery, Respiratory Viruses Branch, Division of Viral Diseases, Centers for Disease Control and Prevention	Yan Li et al
EPI_ISL_419301	2020-03-11	Saitama Prefectural Institute of Public Health	Pathogen Genomics Center, National Institute of Infectious Diseases	Tsuyoshi Sekizuka et al
EPI_ISL_450515	2020-03-11	Rafik Hariri University Hospital	Rafik Hariri University Hospital	Rita Feghali et al
EPI_ISL_523968	2020-03-11	National Agency for Public Health, Republic of Moldova	Charite Universitatsmedizin Berlin, Institute of Virology	Victor M Corman et al
EPI_ISL_456163	2020-03-11	PathLab Bay of Plenty	Institute of Environmental Science and Research (ESR)	Matt Storey et al
EPI_ISL_420146	2020-03-11	Furst Medical Laboratory	Norwegian Institute of Public Health, Department of Virology	Kathrine Stene-Johansen et al
EPI_ISL_523810	2020-03-11	Laboratorio de Referencia Nacional de Virus Respiratorio. Centro Nacional de Salud Publica. Instituto Nacional de Salud Peru.	Laboratorio de Referencia Nacional de Biotecnología y Biología Molecular. Centro Nacional de Salud Publica. Instituto Nacional de Salud Peru.	Carlos Padilla Rojas et al
EPI_ISL_507012	2020-03-11	unknown	Infectious Diseases Research, King Abdullah International Medical Research Center (KAIMRC)	Alghoribi et al
EPI_ISL_418211	2020-03-11	Institut Pasteur Dakar	Institut Pasteur de Dakar	Ndongo Dia et al
EPI_ISL_418212	2020-03-11	Institut Pasteur Dakar	Institut Pasteur de Dakar	Ndongo Dia et al
EPI_ISL_435144	2020-03-11	Hospital Universitario La Paz	Hospital Universitario 12 de Octubre	Dahdouh et al
EPI_ISL_447253	2020-03-11	TSGH-CP molecular lab	TSGH-CP molecular lab	Cherng-Lih Perng et al
EPI_ISL_500636	2020-03-12	Area of Virology, Serology and Virology Division (SAViD), New South Wales Health Pathology Randwick	Area of Virology, Serology and Virology Division (SAViD), New South Wales Health Pathology Randwick	Rawlinson et al
EPI_ISL_729819	2020-03-12	Laboratorio Central de Saude Publica do Estado do Rio Grande do Sul (LACEN-RS)	Laboratory of Respiratory Viruses and Measles, Oswaldo Cruz Institute, FIOCRUZ	Paola Resende et al
EPI_ISL_541370	2020-03-12	LACEN/SC	Laboratory of Respiratory Viruses and Measles, Oswaldo Cruz Institute, FIOCRUZ	Paola Resende et al
EPI_ISL_541375	2020-03-12	LACEN/SE	Laboratory of Respiratory Viruses and Measles, Oswaldo Cruz Institute, FIOCRUZ	Paola Resende et al
EPI_ISL_418846	2020-03-12	BCCDC Public Health Laboratory	BCCDC Public Health Laboratory	Harrigan et al

EPI_ISL_547453	2020-03-12	Dutch COVID-19 response team	National Institute for Public Health and the Environment (RIVM)	Adam Meijer et al
EPI_ISL_644700	2020-03-12	CHU Nîmes	CNR Virus des Infections Respiratoires - France SUD	Antonin Bal et al
EPI_ISL_529961	2020-03-12	Husada Utama Hospital	Institute of Tropical Disease, Universitas Airlangga	Krisnoadi Rahardjo et al
EPI_ISL_419302	2020-03-12	Saitama Prefectural Institute of Public Health	Pathogen Genomics Center, National Institute of Infectious Diseases	Tsuyoshi Sekizuka et al
EPI_ISL_419303	2020-03-12	Saitama Prefectural Institute of Public Health	Pathogen Genomics Center, National Institute of Infectious Diseases	Tsuyoshi Sekizuka et al
EPI_ISL_419309	2020-03-12	Chiba Prefectural Institute of Public Health	Pathogen Genomics Center, National Institute of Infectious Diseases	Tsuyoshi Sekizuka et al
EPI_ISL_455456	2020-03-12	Instituto de Diagnostico y Referencia Epidemiologicos (INDRE)	Instituto de Diagnostico y Referencia Epidemiologicos (INDRE)	Rodriguez-Maldonado Abril et al
EPI_ISL_523966	2020-03-12	National Agency for Public Health, Republic of Moldova	Charite Universitatsmedizin Berlin, Institute of Virology	Victor M Corman et al
EPI_ISL_661189	2020-03-12	Scientific Veterinary Institute Novi Sad	Veterinary Specialized Institute "Kraljevo", Serbia	Vidanovic et al
EPI_ISL_539496	2020-03-13	Hospital Nostra Senyora de Meritxell	Instituto de Salud Carlos III	Iglesias-Caballero et al
EPI_ISL_547445	2020-03-13	Dutch COVID-19 response team	National Institute for Public Health and the Environment (RIVM)	Adam Meijer et al
EPI_ISL_426580	2020-03-13	Instituto Sabin	Laboratory of Virology	Fernando L Melo et al
EPI_ISL_427294	2020-03-13	Instituto Oswaldo Cruz FIOCRUZ - Laboratory of Respiratory Viruses and Measles (LVRS)	Instituto Oswaldo Cruz FIOCRUZ - Laboratory of Respiratory Viruses and Measles (LVRS)	Paola Resende et al
EPI_ISL_427295	2020-03-13	Instituto Oswaldo Cruz FIOCRUZ - Laboratory of Respiratory Viruses and Measles (LVRS)	Instituto Oswaldo Cruz FIOCRUZ - Laboratory of Respiratory Viruses and Measles (LVRS)	Paola Resende et al
EPI_ISL_470574	2020-03-13	Hermes Pardini	Bioinformatics Laboratory / LNCC	Alexandra Gerber et al
EPI_ISL_429671	2020-03-13	Central Public Health Laboratory/Octávio Magalhães Institute (IOM) from the Ezequiel Dias Foundation (FUNED)	Instituto Octávio Magalhães / Fundação Ezequiel Dias (IOM/Funed)	Talita Adelino et al
EPI_ISL_429672	2020-03-13	Central Public Health Laboratory/Octávio Magalhães Institute (IOM) from the Ezequiel Dias Foundation (FUNED)	Instituto Octávio Magalhães / Fundação Ezequiel Dias (IOM/Funed)	Talita Adelino et al
EPI_ISL_541371	2020-03-13	LACEN/SC	Laboratory of Respiratory Viruses and Measles, Oswaldo Cruz Institute, FIOCRUZ	Paola Resende et al
EPI_ISL_486427	2020-03-13	unknown	Clinical Laboratory, Hospital Israelita Albert Einstein	Amgarteb et al
EPI_ISL_418849	2020-03-13	BCCDC Public Health Laboratory	BCCDC Public Health Laboratory	Harrigan et al
EPI_ISL_418857	2020-03-13	BCCDC Public Health Laboratory	BCCDC Public Health Laboratory	Harrigan et al
EPI_ISL_418859	2020-03-13	BCCDC Public Health Laboratory	BCCDC Public Health Laboratory	Harrigan et al
EPI_ISL_454578	2020-03-13	University Hospital for Infectious Diseases "Dr. Fran Mihaljević", Research Unit	University of Zagreb, Centre for research and knowledge transfer in biotechnology	Ivan-Christian Kurolt et al
EPI_ISL_605780	2020-03-13	CEIRS Data Processing and Coordinating Center, St. Jude Center of Excellence for Influenza Research and Surveillance (CEIRS)	CEIRS Data Processing and Coordinating Center, St. Jude Center of Excellence for Influenza Research and Surveillance (CEIRS)	Roshdy et al
EPI_ISL_416482	2020-03-13	R. G. Lugar Center for Public Health Research, National Center for Disease Control and Public Health (NCDC) of Georgia.	R. G. Lugar Center for Public Health Research, National Center for Disease Control and Public Health (NCDC) of Georgia.	Adam Kotorashvili et al
EPI_ISL_613424	2020-03-13	Institut Pasteur de la Guadeloupe	Institut Pasteur de la Guadeloupe	Marion Barbet et al
EPI_ISL_509695	2020-03-13	Guatemala Ministry of Public Health	Pathogen Discovery, Respiratory Viruses Branch, Division of Viral Diseases, Centers for Disease Control and Prevention	Ying Tao et al
EPI_ISL_419582	2020-03-13	Laboratoire National de Santé, Microbiology, Virology	Laboratoire National de Santé, Microbiology, Epidemiology and Microbial Genomics	Anke Wienecke-Baldacchino et al
EPI_ISL_523960	2020-03-13	National Agency for Public Health, Republic of Moldova	Charite Universitatsmedizin Berlin, Institute of Virology	Victor M Corman et al
EPI_ISL_613429	2020-03-13	Institut Pasteur de la Guadeloupe	Institut Pasteur de la Guadeloupe	Marion Barbet et al
EPI_ISL_444493	2020-03-13	Departamento de Laboratorios de Salud Publica (DLSP, Division Epidemiologia, Ministerio de Salud Publica)	Facultad de Ciencias (Sección Genética Evolutiva, Sección Virología).	Panzera et al
EPI_ISL_476184	2020-03-14	DB Diagnósticos do Brasil	Instituto de Medicina Tropical da Univesidade de São Paulo	Samples: Nelson Gaburo Jr et al
EPI_ISL_470578	2020-03-14	Hermes Pardini	Bioinformatics Laboratory / LNCC	Alexandra Gerber et al
EPI_ISL_470579	2020-03-14	Hermes Pardini	Bioinformatics Laboratory / LNCC	Alexandra Gerber et al

EPI_ISL_524798	2020-03-14	Evandro Chagas Institute	Evandro Chagas Institute	Santos et al
EPI_ISL_468752	2020-03-14	Center for Genome Regulation (CRG)	Center for Mathematical Modeling and Center for Genome Regulation. Santiago, Chile	Gaete A et al
EPI_ISL_653752	2020-03-14	Instituto Nacional de Salud, Bogotá, Colombia	Instituto Nacional de Salud, Bogotá, Colombia	Katherine Laiton-Donato et al
EPI_ISL_418410	2020-03-14	Department of Virology and Immunology, University of Helsinki and Helsinki University Hospital, Huslab Finland	Department of Virology, Faculty of Medicine, University of Helsinki, Helsinki, Finland	Teemu Smura et al
EPI_ISL_450795	2020-03-14	Jamaica Ministry of Health and Wellness	Pathogen Discovery, Respiratory Viruses Branch, Division of Viral Diseases, Centers for Disease Control and Prevention	Yan Li et al
EPI_ISL_523962	2020-03-14	National Agency for Public Health, Republic of Moldova	Charite Universitatsmedizin Berlin, Institute of Virology	Victor M Corman et al
EPI_ISL_523964	2020-03-14	National Agency for Public Health, Republic of Moldova	Charite Universitatsmedizin Berlin, Institute of Virology	Victor M Corman et al
EPI_ISL_525206	2020-03-14	Laboratorio de Referencia Nacional de Virus Respiratorio. Centro Nacional de Salud Publica. Instituto Nacional de Salud Peru.	Laboratorio de Referencia Nacional de Biotecnología y Biología Molecular. Centro Nacional de Salud Publica. Instituto Nacional de Salud Peru.	Carlos Padilla Rojas et al
EPI_ISL_536552	2020-03-14	Instituto Nacional de Salud	Laboratorio de Infecciones Respiratorias Agudas	Eduardo Juscamayta Lopez et al
EPI_ISL_547904	2020-03-14	Laboratorio de Infecciones Respiratorias Agudas. Centro Nacional de Salud Publica, Instituto Nacional de Salud	Laboratorio de Infecciones Respiratorias Agudas. Centro Nacional de Salud Publica, Instituto Nacional de Salud	Juscamayta et al
EPI_ISL_507004	2020-03-14	Department of Laboratory Medicine, Tan Tock Seng Hospital	Department of Laboratory Medicine, Tan Tock Seng Hospital	Chen YYC et al
EPI_ISL_548978	2020-03-14	National Public Health Laboratory, National Centre for Infectious Diseases	National Public Health Laboratory, National Centre for Infectious Diseases	Mak TM et al
EPI_ISL_635239	2020-03-14	Institute of Microbiology and Immunology, Faculty of Medicine, University of Ljubljana	Institute of Microbiology and Immunology, Faculty of Medicine, University of Ljubljana	Samo Zakotnik et al
EPI_ISL_427392	2020-03-14	TSGH-CP molecular lab	TSGH-CP molecular lab	Cherng-Lih Perng et al
EPI_ISL_450197	2020-03-14	National Institute of Health. Department of medical Sciences, Ministry of Public Health, Thailand	National Institute of Health. Department of medical Sciences, Ministry of Public Health, Thailand	Pilailuk et al
EPI_ISL_435142	2020-03-14	Mohammed Bin Rashid University of Medicine and Health Sciences	Al Jalila Genomics Center	Ahmad Abou Tayoun et al
EPI_ISL_437977	2020-03-15	Institut für Virologie am Department für Hygiene, Mikrobiologie und Public Health	Bergthaler laboratory, CeMM Research Center for Molecular Medicine of the Austrian Academy of Sciences	Alexandra Popa et al
EPI_ISL_483546	2020-03-15	Kingdom of Bahrein Ministry of Health	Erasmus Medical Center	Bas Oude Munnink et al
EPI_ISL_476822	2020-03-15	Laboratoire des Fièvres Hémorragiques Virales du Benin	Charité-Universitätsmedizin Berlin	Yadouleton et al
EPI_ISL_429675	2020-03-15	Central Public Health Laboratory/Octávio Magalhães Institute (IOM) from the Ezequiel Dias Foundation (FUNED)	Instituto Octávio Magalhães / Fundação Ezequiel Dias (IOM/Funed)	Talita Adelino et al
EPI_ISL_445274	2020-03-15	LABORATORIO TORRE MEDICA LTDA.	Instituto de Salud Publica de Chile	Andrés E Castillo et al
EPI_ISL_602532	2020-03-15	Institute for Virology, University Hospital Essen	Center of Medical Microbiology, Virology, and Hospital Hygiene, University of Duesseldorf	Olympia E. Anastasiou et al
EPI_ISL_613423	2020-03-15	Institut Pasteur de la Guadeloupe	Institut Pasteur de la Guadeloupe	Marion Barbet et al
EPI_ISL_509710	2020-03-15	Guatemala Ministry of Public Health	Pathogen Discovery, Respiratory Viruses Branch, Division of Viral Diseases, Centers for Disease Control and Prevention	Jing Zhang et al
EPI_ISL_509697	2020-03-15	Guatemala Ministry of Public Health	Pathogen Discovery, Respiratory Viruses Branch, Division of Viral Diseases, Centers for Disease Control and Prevention	Ying Tao et al
EPI_ISL_509698	2020-03-15	Guatemala Ministry of Public Health	Pathogen Discovery, Respiratory Viruses Branch, Division of Viral Diseases, Centers for Disease Control and Prevention	Ying Tao et al
EPI_ISL_474960	2020-03-15	Israel Central Virology laboratory	Israel Central Virology laboratory	Neta Zuckerman et al
EPI_ISL_422424	2020-03-15	Jaber Al Ahmad Al Sabah Hospital	Dasman diabetes Institute	Fahd Al-Mulla et al
EPI_ISL_459967	2020-03-15	Institut Pasteur du Maroc	Institut Pasteur du Maroc	Marion Barbet et al
EPI_ISL_469248	2020-03-15	Special Infectious Agents Unit	Special Infectious Agents Unit	Azhar et al
EPI_ISL_498026	2020-03-15	Division of Viral Diseases, Center for Laboratory Control of Infectious Diseases, Korea Centers for Diseases Control and Prevention	Division of Viral Diseases, Center for Laboratory Control of Infectious Diseases, Korea Centers for Diseases Control and Prevention	Jeong-Min Kim et al

EPI_ISL_435127	2020-03-15	Mohammed Bin Rashid University of Medicine and Health Sciences	Al Jalila Genomics Center	Ahmad Abou Tayoun et al
EPI_ISL_417034	2020-03-16	Laboratorio de Ecologia de Doencas Transmissiveis na Amazonia, Instituto Leonidas e Maria Deane - Fiocruz Amazonia	Laboratorio de Ecologia de Doencas Transmissiveis na Amazonia, Instituto Leonidas e Maria Deane - Fiocruz Amazonia	Valdinete Nascimento et al
EPI_ISL_476224	2020-03-16	DB Diagnósticos do Brasil	Instituto de Medicina Tropical da Univesidade de São Paulo	Samples: Nelson Gaburo Jr et al
EPI_ISL_476182	2020-03-16	DB Diagnósticos do Brasil	Instituto de Medicina Tropical da Univesidade de São Paulo	Samples: Nelson Gaburo Jr et al
EPI_ISL_429676	2020-03-16	Central Public Health Laboratory/Octávio Magalhães Institute (IOM) from the Ezequiel Dias Foundation (FUNED)	Instituto Octávio Magalhães / Fundação Ezequiel Dias (IOM/Funed)	Talita Adelino et al
EPI_ISL_429679	2020-03-16	Central Public Health Laboratory/Octávio Magalhães Institute (IOM) from the Ezequiel Dias Foundation (FUNED)	Instituto Octávio Magalhães / Fundação Ezequiel Dias (IOM/Funed)	Talita Adelino et al
EPI_ISL_476194	2020-03-16	DB Diagnósticos do Brasil	Instituto de Medicina Tropical da Univesidade de São Paulo	Samples: Nelson Gaburo Jr et al
EPI_ISL_541342	2020-03-16	LACEN/PR	Laboratory of Respiratory Viruses and Measles, Oswaldo Cruz Institute, FIOCRUZ	Paola Resende et al
EPI_ISL_476191	2020-03-16	DB Diagnósticos do Brasil	Instituto de Medicina Tropical da Univesidade de São Paulo	Samples: Nelson Gaburo Jr et al
EPI_ISL_476178	2020-03-16	DB Diagnósticos do Brasil	Instituto de Medicina Tropical da Univesidade de São Paulo	Samples: Nelson Gaburo Jr et al
EPI_ISL_476180	2020-03-16	DB Diagnósticos do Brasil	Instituto de Medicina Tropical da Univesidade de São Paulo	Samples: Nelson Gaburo Jr et al
EPI_ISL_528637	2020-03-16	LVM/UFRJ	Bioinformatics Laboratory / LNCC	Gustavo D. P. Silva et al
EPI_ISL_528638	2020-03-16	LVM/UFRJ	Bioinformatics Laboratory / LNCC	Gustavo D. P. Silva et al
EPI_ISL_729806	2020-03-16	Laboratorio Central de Saude Publica do Estado do Rio Grande do Sul (LACEN-RS)	Laboratory of Respiratory Viruses and Measles, Oswaldo Cruz Institute, FIOCRUZ	Paola Resende et al
EPI_ISL_476192	2020-03-16	DB Diagnósticos do Brasil	Instituto de Medicina Tropical da Univesidade de São Paulo	Samples: Nelson Gaburo Jr et al
EPI_ISL_476195	2020-03-16	DB Diagnósticos do Brasil	Instituto de Medicina Tropical da Univesidade de São Paulo	Samples: Nelson Gaburo Jr et al
EPI_ISL_476211	2020-03-16	DB Diagnósticos do Brasil	Instituto de Medicina Tropical da Univesidade de São Paulo	Samples: Nelson Gaburo Jr et al
EPI_ISL_574292	2020-03-16	Instituto Nacional de Investigación en Salud Pública	Instituto Nacional de Investigación en Salud Pública	Leandro Patino Patino et al
EPI_ISL_416481	2020-03-16	R. G. Lugar Center for Public Health Research, National Center for Disease Control and Public Health (NCDC) of Georgia.	R. G. Lugar Center for Public Health Research, National Center for Disease Control and Public Health (NCDC) of Georgia.	Gvantsa Chanturia et al
EPI_ISL_613431	2020-03-16	Institut Pasteur de la Guadeloupe	Institut Pasteur de la Guadeloupe	Marion Barbet et al
EPI_ISL_610164	2020-03-16	Department of Health Technology and Informatics, The Hong Kong Polytechnic University	Department of Health Technology and Informatics, The Hong Kong Polytechnic University	Siu et al
EPI_ISL_497845	2020-03-16	Department of Microbiology, The University of Hong Kong	Department of Microbiology, The University of Hong Kong	Kelvin K.W. To et al
EPI_ISL_417764	2020-03-16	The National University Hospital of Iceland	deCODE genetics	Daniel F Gudbjartsson et al
EPI_ISL_529962	2020-03-16	Universitas Airlangga Hospital	Institute of Tropical Disease, Universitas Airlangga	Jezzy R Dewantari et al
EPI_ISL_450796	2020-03-16	Jamaica Ministry of Health and Wellness	Pathogen Discovery, Respiratory Viruses Branch, Division of Viral Diseases, Centers for Disease Control and Prevention	Yan Li et al
EPI_ISL_429997	2020-03-16	Biolab Diagnostic Laboratories	Andersen lab at Scripps Research	Issa Abu-Dayyeh et al
EPI_ISL_421652	2020-03-16	Dasman Diabetes Institute	Dasman Diabetes Institute	Fahd Al-Mulla et al
EPI_ISL_422426	2020-03-16	JABER AL AHMAD AL SABAH HOSPITAL – KUWAIT CITY	Dasman Diabetes Institute	Fahd Al-Mulla et al
EPI_ISL_422427	2020-03-16	JABER AL AHMAD AL SABAH HOSPITAL – KUWAIT CITY	Dasman Diabetes Institute	Fahd Al-Mulla et al
EPI_ISL_419588	2020-03-16	Laboratoire National de Santé, Microbiology, Virology	Laboratoire National de Santé, Microbiology, Epidemiology and Microbial Genomics	Anke Wienecke-Baldacchino et al
EPI_ISL_451958	2020-03-16	Jamil-ur-Rahman Center for Genome Research, Dr. Panjwani Center for Molecular Medicine and Drug Research, International Center for Chemical and Biological Sciences, University of Karachi	Jamil-ur-Rahman Center for Genome Research, Dr. Panjwani Center for Molecular Medicine and Drug Research, International Center for Chemical and Biological Sciences, University of Karachi	Shakeel et al
EPI_ISL_596505	2020-03-16	Palestinian Ministry of Health	Molecular Genetics Lab	Nouar Qutob et al
EPI_ISL_596506	2020-03-16	Palestinian Ministry of Health	Molecular Genetics Lab	Nouar Qutob et al
EPI_ISL_536536	2020-03-16	Instituto Nacional de Salud	Laboratorio de Infecciones Respiratorias Agudas	Eduardo Juscamayta Lopez et al

EPI_ISL_511042	2020-03-16	Instituto Nacional de Saude (INSA)	Instituto Nacional de Saude (INSA)	Borges et al et al
EPI_ISL_511043	2020-03-16	Instituto Nacional de Saude (INSA)	Instituto Nacional de Saude (INSA)	Borges et al et al
EPI_ISL_428670	2020-03-16	Centre for Dengue Research	Centre for Dengue Research	Chandima Jeewandara et al
EPI_ISL_528133	2020-03-16	University Hospital Basel, Clinical Virology	University Hospital Basel, Clinical Bacteriology	Madlen Stange et al
EPI_ISL_654016	2020-03-16	Laboratory of Microbiology, National Reference Lab, Charles Nicolle Hospital; 2-University of Tunis ElManar, Faculty of Medicine of Tunis, LR99ES09, Tunis, Tunisia	1-Clinical and Experimental Pharmacology Lab, LR16SP02, National Center of Pharmacovigilance, University of Tunis El Manar, Tunis, Tunisia. 2-Neurodegenerative diseases and psychiatric troubles, LR18SP03, Razi Hospital, University of Tunis El Manar, Tunis, Tunisia. 3- Ministry of Health, National Observatory of New and Emerging Diseases, 1006, Tunis, Tunisia	Ilhem Boutiba-Ben Boubaker et al
EPI_ISL_435125	2020-03-16	Mohammed Bin Rashid University of Medicine and Health Sciences	Al Jalila Genomics Center	Ahmad Abou Tayoun et al
EPI_ISL_510529	2020-03-16	School of Veterinary Medicine, Disease Control	School of Veterinary Medicine, Disease Control	Simulundu et al
EPI_ISL_547446	2020-03-17	Dutch COVID-19 response team	National Institute for Public Health and the Environment (RIVM)	Adam Meijer et al
EPI_ISL_427731	2020-03-17	Centre for Infectious Diseases and Microbiology Public Health	NSW Health Pathology - Institute of Clinical Pathology and Medical Research; Westmead Hospital; University of Sydney	Gray K et al
EPI_ISL_419853	2020-03-17	Victorian Infectious Diseases Reference Laboratory (VIDRL)	Victorian Infectious Diseases Reference Laboratory and Microbiological Diagnostic Unit Public Health Laboratory, Doherty Institute	Caly L. et al
EPI_ISL_476833	2020-03-17	Laboratoire des Fièvres Hémorragiques Virales du Benin	Charité-Universitätsmedizin Berlin	Yadouleton et al
EPI_ISL_476824	2020-03-17	Laboratoire des Fièvres Hémorragiques Virales du Benin	Charité-Universitätsmedizin Berlin	Yadouleton et al
EPI_ISL_450873	2020-03-17	Evandro Chagas Institute	Evandro Chagas Institute	Santos et al
EPI_ISL_417925	2020-03-17	Laboratório Simili	Bioinformatics Laboratory / LNCC	Filipe Romero et al
EPI_ISL_470580	2020-03-17	Hermes Pardini	Bioinformatics Laboratory / LNCC	Alexandra Gerber et al
EPI_ISL_429684	2020-03-17	Central Public Health Laboratory/Octávio Magalhães Institute (IOM) from the Ezequiel Dias Foundation (FUNED)	Instituto Octávio Magalhães / Fundação Ezequiel Dias (IOM/Funed)	Talita Adelino et al
EPI_ISL_429688	2020-03-17	Central Public Health Laboratory/Octávio Magalhães Institute (IOM) from the Ezequiel Dias Foundation (FUNED)	Instituto Octávio Magalhães / Fundação Ezequiel Dias (IOM/Funed)	Talita Adelino et al
EPI_ISL_429689	2020-03-17	Central Public Health Laboratory/Octávio Magalhães Institute (IOM) from the Ezequiel Dias Foundation (FUNED)	Instituto Octávio Magalhães / Fundação Ezequiel Dias (IOM/Funed)	Talita Adelino et al
EPI_ISL_541340	2020-03-17	LACEN/PR	Laboratory of Respiratory Viruses and Measles, Oswaldo Cruz Institute, FIOCRUZ	Paola Resende et al
EPI_ISL_541341	2020-03-17	LACEN/PR	Laboratory of Respiratory Viruses and Measles, Oswaldo Cruz Institute, FIOCRUZ	Paola Resende et al
EPI_ISL_729805	2020-03-17	Laboratorio Central de Saude Publica do Estado do Rio Grande do Sul (LACEN-RS)	Laboratory of Respiratory Viruses and Measles, Oswaldo Cruz Institute, FIOCRUZ	Paola Resende et al
EPI_ISL_524462	2020-03-17	Hospital Metropolitano	Instituto Adolfo Lutz, Interdiciplinary Procedures Center, Strategic Laboratory	Claudio Tavares Sacchi et al
EPI_ISL_459862	2020-03-17	Center for Genome Regulation (CRG)	Center for Mathematical Modeling and Center for Genome Regulation. Santiago, Chile	Gaete A et al
EPI_ISL_445317	2020-03-17	UNIV.DE CHILE HOSP.CLINICO	Instituto de Salud Publica de Chile	Andrés E Castillo et al
EPI_ISL_445319	2020-03-17	HOSPITAL FELIX BULNES	Instituto de Salud Publica de Chile	Andrés E Castillo et al
EPI_ISL_445321	2020-03-17	HOSPITAL FELIX BULNES	Instituto de Salud Publica de Chile	Andrés E Castillo et al
EPI_ISL_445362	2020-03-17	BUPA SERVICIOS CLINICOS S.A	Instituto de Salud Publica de Chile	Andrés E Castillo et al
EPI_ISL_456139	2020-03-17	Instituto Nacional de Salud - Unidad de Secuenciación y Análisis Genómico	Instituto Nacional de Salud, Universidad Cooperativa de Colombia, Instituto Alexander von Humboldt, Imperial College-London, London School of Hygiene & Tropical Medicine	Katherine Laiton-Donato et al
EPI_ISL_512654	2020-03-17	Hospital Dr. Rafael A. Calderon Guardia [San Jose/San Jose]	Inciensa, Instituto Costarricense de Investigación y Enseñanza en Nutrición y Salud	Francisco Duarte et al
EPI_ISL_707782	2020-03-17	CHU Poitiers	CNR Virus des Infections Respiratoires - France SUD	Antonin Bal et al

EPI_ISL_428855	2020-03-17	MRCG at LSHTM Geomics lab	MRCG at LSHTM Genomics lab	Sesay et al et al
EPI_ISL_451937	2020-03-17	Max von Pettenkofer Institute, Virology, National Reference Center for Retroviruses, LMU München	Laboratory for Functional Genome Analysis, Dept. Genomics, Gene Center of the LMU Munich	Max Muenchhoff et al
EPI_ISL_417569	2020-03-17	The National University Hospital of Iceland	deCODE genetics	Daniel F Gudbjartsson et al
EPI_ISL_417610	2020-03-17	The National University Hospital of Iceland	deCODE genetics	Daniel F Gudbjartsson et al
EPI_ISL_450325	2020-03-17	NIV Pune	CSIR-Centre for Cellular and Molecular Biology	Dr V A Potdar et al
EPI_ISL_429226	2020-03-17	Presidio Ospedaliero Santo Spirito	Istituto Zooprofilattico Sperimentale dell'Abruzzo e Molise "G. Caporale"	Lorusso A et al
EPI_ISL_429994	2020-03-17	Biolab Diagnostic Laboratories	Andersen lab at Scripps Research	Issa Abu-Dayyeh et al
EPI_ISL_429999	2020-03-17	Biolab Diagnostic Laboratories	Andersen lab at Scripps Research	Issa Abu-Dayyeh et al
EPI_ISL_430014	2020-03-17	Biolab Diagnostic Laboratories	Andersen lab at Scripps Research	Issa Abu-Dayyeh et al
EPI_ISL_457845	2020-03-17	KEMRI-CGMR-C	KEMRI-Wellcome Trust Research Programme/KEMRI-CGMR-C Kilifi	Githinji G. et al 2020 et al
EPI_ISL_486423	2020-03-17	Latvijas Infektoloģijas centrs	Latvian Biomedical Research and Study Centre	Ivars Silamiķelis et al
EPI_ISL_421760	2020-03-17	Laboratoire National de Sante, Microbiology, Virology	Laboratoire National de Sante, Microbiology, Epidemiology and Microbial Genomics	Anke Wienecke-Baldacchino et al
EPI_ISL_501221	2020-03-17	Department of Medical Microbiology, University Malaya Medical Centre	Department of Medical Microbiology, Faculty of Medicine, University of Malaya	Yoong Min CHONG et al
EPI_ISL_516922	2020-03-17	Department for Molecular Diagnostics, Centre for Medical Microbiology, Institute of Public Health of Montenegro	Charité Universitätsmedizin Berlin, Institut für Virologie	Victor M Corman et al
EPI_ISL_420151	2020-03-17	Nordland Hospital - Bodo, Laboratory Department, Molecular Biology Unit	Norwegian Institute of Public Health, Department of Virology	Kathrine Stene-Johansen et al
EPI_ISL_536543	2020-03-17	Instituto Nacional de Salud	Laboratorio de Infecciones Respiratorias Agudas	Eduardo Juscamayta Lopez et al
EPI_ISL_420069	2020-03-17	Institut Pasteur Dakar	Institut Pasteur de Dakar	Ndongo Dia et al
EPI_ISL_426631	2020-03-17	TSGH-CP molecular lab	TSGH-CP molecular lab	Cherng-Lih Perng et al
EPI_ISL_500716	2020-03-17	BSL3 Lab Pendik Veterinary Control Institute	Department of Medicinal Genetics, Bursa Uludağ University, Faculty of medicine By Sehime Gülsün Temel, Adem Alemdar, Kadir Yeşilbağ	Mustafa HASOKSUZ et al
EPI_ISL_491476	2020-03-17	BSL3 Lab, Pendik Veterinary Control Enstitue	Genomic Laboratory (GLAB), Istanbul Technical University	Mustafa HASOKSUZ et al
EPI_ISL_526805	2020-03-17	Virginia DCLS	Virginia DCLS	Virginia DCLS et al
EPI_ISL_426479	2020-03-17	Microbial Genomics Laboratory, Institut Pasteur Montevideo	Microbial Genomics Laboratory, Institut Pasteur Montevideo	Cecilia Salazar et al
EPI_ISL_457944	2020-03-17	Laboratorio de Biología Molecular Asociación Española Primera en Salud	Departments of Pathology and Medicine, New York University School of Medicine	Maria Victoria Elizondo et al
EPI_ISL_454954	2020-03-17	Wuhan Chain Medical Labs (CMLabs)	State Key Laboratory of Biotherapy of Sichuan University	Baowen Du et al
EPI_ISL_547447	2020-03-18	Dutch COVID-19 response team	National Institute for Public Health and the Environment (RIVM)	Adam Meijer et al
EPI_ISL_483549	2020-03-18	Kingdom of Bahrein Ministry of Health	Erasmus Medical Center	Bas Oude Munnink et al
EPI_ISL_483550	2020-03-18	Kingdom of Bahrein Ministry of Health	Erasmus Medical Center	Bas Oude Munnink et al
EPI_ISL_458139	2020-03-18	Evandro Chagas Institute	Evandro Chagas Institute	Santos et al
EPI_ISL_427292	2020-03-18	LACEN-AL - Laboratorio Central de Alagoas	Instituto Oswaldo Cruz FIOCRUZ - Laboratory of Respiratory Viruses and Measles (LVRS)	Paola Resende et al
EPI_ISL_470575	2020-03-18	Hermes Pardini	Bioinformatics Laboratory / LNCC	Alexandra Gerber et al
EPI_ISL_623104	2020-03-18	Simile Medicina Diagnóstica	Bioinformatics Laboratory / LNCC	Carolina M Voloch et al
EPI_ISL_729807	2020-03-18	Laboratorio Central de Saude Publica do Estado do Rio Grande do Sul (LACEN-RS)	Laboratory of Respiratory Viruses and Measles, Oswaldo Cruz Institute, FIOCRUZ	Paola Resende et al
EPI_ISL_470652	2020-03-18	Hermes Pardini	Bioinformatics Laboratory / LNCC	Alexandra Gerber et al
EPI_ISL_470653	2020-03-18	Hermes Pardini	Bioinformatics Laboratory / LNCC	Alexandra Gerber et al
EPI_ISL_470654	2020-03-18	Hermes Pardini	Bioinformatics Laboratory / LNCC	Alexandra Gerber et al

EPI_ISL_515527	2020-03-18	Hospital Santa Clara	Instituto Adolfo Lutz, Interdiciplinary Procedures Center, Strategic Laboratory	Claudio Tavares Sacchi et al
EPI_ISL_450506	2020-03-18	Clinical Laboratory, Hospital Israelita Albert Einstein	Clinical Laboratory, Hospital Israelita Albert Einstein	Malta et al
EPI_ISL_456142	2020-03-18	Instituto Nacional de Salud - Unidad de Secuenciación y Análisis Genómico	Instituto Nacional de Salud, Universidad Cooperativa de Colombia, Instituto Alexander von Humboldt, Imperial College-London, London School of Hygiene & Tropical Medicine	Katherine Laiton-Donato et al
EPI_ISL_480322	2020-03-18	Hospital Nacional de Niños	Charité Virology-University of Costa Rica	Andres Moreira-Soto et al
EPI_ISL_454581	2020-03-18	University Hospital for Infectious Diseases "Dr. Fran Mihaljević", Research Unit	University of Zagreb, Centre for research and knowledge transfer in biotechnology	Ivan-Christian Kurolt et al
EPI_ISL_454583	2020-03-18	University Hospital for Infectious Diseases "Dr. Fran Mihaljević", Research Unit	University of Zagreb, Centre for research and knowledge transfer in biotechnology	Ivan-Christian Kurolt et al
EPI_ISL_426360	2020-03-18	Laboratory of Molecular Genetics, 2nd Faculty of Medicine, Charles University in Prague, Prague, Czech Republic	Laboratory of Molecular Genetics, 2nd Faculty of Medicine, Charles University in Prague, Prague, Czech Republic	Lenka Kramna et al
EPI_ISL_417946	2020-03-18	Viral Respiratory Lab, National Institute for Biomedical Research (INRB)	Pathogen Sequencing Lab, National Institute for Biomedical Research (INRB)	Placide Mbala-Kingebeni et al
EPI_ISL_430820	2020-03-18	Center of Scientific Excellence for Influenza Viruses, National Research Centre (NRC), Egypt.	Center of Scientific Excellence for Influenza Viruses, National Research Centre (NRC), Egypt.	Mohamed Ahmed Ali et al
EPI_ISL_430819	2020-03-18	Center of Scientific Excellence for Influenza Viruses,National Research Centre (NRC), Egypt.	Center of Scientific Excellence for Influenza Viruses,National Research Centre (NRC), Egypt.	Mohamed Ahmed Ali et al
EPI_ISL_420043	2020-03-18	CMIP	National Reference Center for Viruses of Respiratory Infections, Institut Pasteur, Paris	Mélanie Albert et al
EPI_ISL_613419	2020-03-18	Institut Pasteur de la Guadeloupe	Institut Pasteur de la Guadeloupe	Marion Barbet et al
EPI_ISL_497820	2020-03-18	Department of Microbiology, The University of Hong Kong	Department of Microbiology, The University of Hong Kong	Kelvin K.W. To et al
EPI_ISL_497840	2020-03-18	Department of Microbiology, The University of Hong Kong	Department of Microbiology, The University of Hong Kong	Kelvin K.W. To et al
EPI_ISL_528925	2020-03-18	Ospedale Regionale San Salvatore-L'Aquila	Istitituto Zooprofilattico Sperimentale dell'Abruzzo e Molise "G.Caporale"	Lorusso A et al
EPI_ISL_419305	2020-03-18	Saitama Prefectural Institute of Public Health	Pathogen Genomics Center, National Institute of Infectious Diseases	Tsuyoshi Sekizuka et al
EPI_ISL_498552	2020-03-18	Lebanese American University	Lebanese American University	Abi Habib et al
EPI_ISL_417530	2020-03-18	Laboratoire Nationale de Santé, Microbiology, Virology	Laboratoire Nationale de Santé, Microbiology, Epidemiology and Microbial Genomics	Anke Wienecke-Baldacchino et al
EPI_ISL_501223	2020-03-18	Department of Medical Microbiology, University Malaya Medical Centre	Department of Medical Microbiology, Faculty of Medicine, University of Malaya	Yoong Min CHONG et al
EPI_ISL_455454	2020-03-18	Instituto de Diagnostico y Referencia Epidemiologicos (INDRE)	Instituto de Diagnostico y Referencia Epidemiologicos (INDRE)	Mendieta-Condado Edgar et al
EPI_ISL_516923	2020-03-18	Department for Molecular Diagnostics, Centre for Medical Microbiology, Institute of Public Health of Montenegro	Charité Universitätsmedizin Berlin, Institut für Virologie	Victor M Corman et al
EPI_ISL_457979	2020-03-18	Oman-NIC	Oman-NIC	Samira Al-Maruqi et al
EPI_ISL_436227	2020-03-18	Servicio de Microbiología. Consorcio Hospital General Universitario de Valencia	Sequencing and Bioinformatics Service and Molecular Epidemiology Research Group. FISABIO-Public Health	Griselda De Marco et al
EPI_ISL_574972	2020-03-18	Viollier AG	Department of Biosystems Science and Engineering, ETH Zürich	Christian Beisel et al
EPI_ISL_444275	2020-03-18	Laboratory Medicine	Department of Laboratory Medicine, Lin-Kou Chang Gung Memorial Hospital, Taoyuan, Taiwan	Kuo-Chien Tsao et al
EPI_ISL_463001	2020-03-18	unknown	Clinical virology	Fares et al
EPI_ISL_475832	2020-03-19	Austrian Agency for Health and Food Safety (AGES)	Bergthaler laboratory, CeMM Research Center for Molecular Medicine of the Austrian Academy of Sciences	Alexandra Popa et al
EPI_ISL_476829	2020-03-19	Laboratoire des Fièvres Hémorragiques Virales du Benin	Charité-Universitätsmedizin Berlin	Yadouleton et al
EPI_ISL_470582	2020-03-19	Hermes Pardini	Bioinformatics Laboratory / LNCC	Alexandra Gerber et al
EPI_ISL_470599	2020-03-19	Hermes Pardini	Bioinformatics Laboratory / LNCC	Alexandra Gerber et al
EPI_ISL_524801	2020-03-19	Evandro Chagas Institute	Evandro Chagas Institute	Santos et al
EPI_ISL_470613	2020-03-19	Hermes Pardini	Bioinformatics Laboratory / LNCC	Alexandra Gerber et al

EPI_ISL_541344	2020-03-19	LACEN/PR	Laboratory of Respiratory Viruses and Measles, Oswaldo Cruz Institute, FIOCRUZ	Paola Resende et al
EPI_ISL_729808	2020-03-19	Laboratorio Central de Saude Publica do Estado do Rio Grande do Sul (LACEN-RS)	Laboratory of Respiratory Viruses and Measles, Oswaldo Cruz Institute, FIOCRUZ	Paola Resende et al
EPI_ISL_729809	2020-03-19	Laboratorio Central de Saude Publica do Estado do Rio Grande do Sul (LACEN-RS)	Laboratory of Respiratory Viruses and Measles, Oswaldo Cruz Institute, FIOCRUZ	Paola Resende et al
EPI_ISL_476188	2020-03-19	DB Diagnósticos do Brasil	Instituto de Medicina Tropical da Univesidade de São Paulo	Samples: Nelson Gaburo Jr et al
EPI_ISL_541374	2020-03-19	LACEN/SE	Laboratory of Respiratory Viruses and Measles, Oswaldo Cruz Institute, FIOCRUZ	Paola Resende et al
EPI_ISL_541376	2020-03-19	LACEN/SE	Laboratory of Respiratory Viruses and Measles, Oswaldo Cruz Institute, FIOCRUZ	Paola Resende et al
EPI_ISL_515554	2020-03-19	Pronto Socorro Municipal de Perus	Instituto Adolfo Lutz, Interdiciplinary Procedures Center, Strategic Laboratory	Claudio Tavares Sacchi et al
EPI_ISL_476201	2020-03-19	DB Diagnósticos do Brasil	Instituto de Medicina Tropical da Univesidade de São Paulo	Samples: Nelson Gaburo Jr et al
EPI_ISL_629026	2020-03-19	Centro de Biotecnología Vegetal, Universidad Andrés Bello, Center for Genome Regulation	Center for Mathematical Modeling and Center for Genome Regulation. Santiago, Chile	Bastias M et al
EPI_ISL_513312	2020-03-19	Public Health, United States Air Force School of Aerospace Medicine	Public Health, United States Air Force School of Aerospace Medicine	Fries et al
EPI_ISL_605113	2020-03-19	National Virus Reference Laboratory	Irish Coronavirus Sequencing Consortium - Helixworks	Sachin Chalapati et al
EPI_ISL_498551	2020-03-19	Lebanese American University	Lebanese American University	Abi Habib et al
EPI_ISL_516924	2020-03-19	Department for Molecular Diagnostics, Centre for Medical Microbiology, Institute of Public Health of Montenegro	Charité Universitätsmedizin Berlin, Institut für Virologie	Victor M Corman et al
EPI_ISL_460715	2020-03-19	Dutch COVID-19 response team	Erasmus Medical Center	Bas Oude Munnink et al
EPI_ISL_496695	2020-03-19	Gorgas Memorial Laboratory of Health Studies	Gorgas Memorial Laboratory of Health Studies	Danilo Franco et al
EPI_ISL_462277	2020-03-19	National Public Health Laboratory, National Centre for Infectious Diseases	National Public Health Laboratory, National Centre for Infectious Diseases	Mak TM et al
EPI_ISL_428672	2020-03-19	Centre for Dengue Research	Centre for Dengue Research	Chandima Jeewandara et al
EPI_ISL_574953	2020-03-19	Viollier AG	Department of Biosystems Science and Engineering, ETH Zürich	Christian Beisel et al
EPI_ISL_547448	2020-03-20	Dutch COVID-19 response team	National Institute for Public Health and the Environment (RIVM)	Adam Meijer et al
EPI_ISL_636513	2020-03-20	Dutch COVID-19 response team	National Institute for Public Health and the Environment (RIVM)	Adam Meijer et al
EPI_ISL_509711	2020-03-20	Belize Ministry of Health	Pathogen Discovery, Respiratory Viruses Branch, Division of Viral Diseases, Centers for Disease Control and Prevention	Jing Zhang et al
EPI_ISL_462455	2020-03-20	Clinical Center, University of Sarajevo	Charite Universitatsmedizin Berlin, Institute of Virology	Victor M Corman et al
EPI_ISL_462457	2020-03-20	Clinical Center, University of Sarajevo	Charite Universitatsmedizin Berlin, Institute of Virology	Victor M Corman et al
EPI_ISL_470583	2020-03-20	Hermes Pardini	Bioinformatics Laboratory / LNCC	Alexandra Gerber et al
EPI_ISL_429695	2020-03-20	Central Public Health Laboratory/Octávio Magalhães Institute (IOM) from the Ezequiel Dias Foundation (FUNED)	Instituto Octávio Magalhães / Fundação Ezequiel Dias (IOM/Funed)	Talita Adelino et al
EPI_ISL_429701	2020-03-20	Central Public Health Laboratory/Octávio Magalhães Institute (IOM) from the Ezequiel Dias Foundation (FUNED)	Instituto Octávio Magalhães / Fundação Ezequiel Dias (IOM/Funed)	Talita Adelino et al
EPI_ISL_450874	2020-03-20	Evandro Chagas Institute	Evandro Chagas Institute	Santos et al
EPI_ISL_541343	2020-03-20	LACEN/PR	Laboratory of Respiratory Viruses and Measles, Oswaldo Cruz Institute, FIOCRUZ	Paola Resende et al
EPI_ISL_427302	2020-03-20	Instituto Oswaldo Cruz FIOCRUZ - Laboratory of Respiratory Viruses and Measles (LVRS)	Instituto Oswaldo Cruz FIOCRUZ - Laboratory of Respiratory Viruses and Measles (LVRS)	Paola Resende et al
EPI_ISL_470651	2020-03-20	Hermes Pardini	Bioinformatics Laboratory / LNCC	Alexandra Gerber et al
EPI_ISL_729810	2020-03-20	Laboratorio Central de Saude Publica do Estado do Rio Grande do Sul (LACEN-RS)	Laboratory of Respiratory Viruses and Measles, Oswaldo Cruz Institute, FIOCRUZ	Paola Resende et al
EPI_ISL_729812	2020-03-20	Laboratorio Central de Saude Publica do Estado do Rio Grande do Sul (LACEN-RS)	Laboratory of Respiratory Viruses and Measles, Oswaldo Cruz Institute, FIOCRUZ	Paola Resende et al

EPI_ISL_541373	2020-03-20	LACEN/SE	Laboratory of Respiratory Viruses and Measles, Oswaldo Cruz Institute, FIOCRUZ	Paola Resende et al
EPI_ISL_471546	2020-03-20	AMA DR Jose Soares Hungria	Instituto Adolfo Lutz, Interdiciplinary Procedures Center, Strategic Laboratory	Claudio Tavares Sacchi et al
EPI_ISL_486429	2020-03-20	unknown	Clinical Laboratory, Hospital Israelita Albert Einstein	Malta et al
EPI_ISL_456146	2020-03-20	Instituto Nacional de Salud - Unidad de Secuenciación y Análisis Genómico	Instituto Nacional de Salud, Universidad Cooperativa de Colombia, Instituto Alexander von Humboldt, Imperial College-London, London School of Hygiene & Tropical Medicine	Katherine Laiton-Donato et al
EPI_ISL_454588	2020-03-20	University Hospital for Infectious Diseases "Dr. Fran Mihaljević", Research Unit	University of Zagreb, Centre for research and knowledge transfer in biotechnology	Ivan-Christian Kurolt et al
EPI_ISL_425287	2020-03-20	Department of Pathology, University of Cambridge	COVID-19 Genomics UK (COG-UK) Consortium	Luke W Meredith et al
EPI_ISL_471160	2020-03-20	MRCG at LSHTM Genomics lab	MRCG at LSHTM Genomics lab	Sesay et al et al
EPI_ISL_437258	2020-03-20	Max von Pettenkofer Institute, Virology, National Reference Center for Retroviruses, LMU München	Laboratory for Functional Genome Analysis, Dept. Genomics, Gene Center of the LMU Munich	Max Muenchhoff et al
EPI_ISL_445000	2020-03-20	Naval Health Research Center	Naval Medical Research Center Biological Defense Research Directorate	Logan Voegtly et al
EPI_ISL_424471	2020-03-20	The National University Hospital of Iceland	deCODE genetics	Daniel F Gudbjartsson et al
EPI_ISL_431117	2020-03-20	Department of Microbiology, Gandhi Medical College and Hospital, Secendrabad, Hyderabad, India	Department of Microbiology, Gandhi Medical College and Hospital, Secendrabad, Hyderabad, India	Thrilok Chander B et al
EPI_ISL_508862	2020-03-20	Virology Unit, Institut Pasteur de Madagascar	Virology Unit, Institut Pasteur de Madagascar	Christian Ranaivoson et al
EPI_ISL_455435	2020-03-20	Instituto de Diagnostico y Referencia Epidemiologicos (INDRE)	Instituto de Diagnostico y Referencia Epidemiologicos (INDRE)	Garces-Ayala Fabiola. Taboada Ramírez Blanca. Ramirez-Gonzalez Ernesto et al
EPI_ISL_459973	2020-03-20	Institut Pasteur du Maroc	Institut Pasteur du Maroc	Marion Barbet et al
EPI_ISL_456194	2020-03-20	PathLab Bay of Plenty	Institute of Environmental Science and Research (ESR)	Matt Storey et al
EPI_ISL_447837	2020-03-20	Dept. of Medical Microbiology, Stavanger University Hospital, Helse Stavanger HF,	Norwegian Institute of Public Health, Department of Virology	Kathrine Stene-Johansen et al
EPI_ISL_457986	2020-03-20	Oman-NIC	Oman-NIC	Samira Al-Maruqi et al
EPI_ISL_457987	2020-03-20	Oman-NIC	Oman-NIC	Samira Al-Maruqi et al
EPI_ISL_536557	2020-03-20	Instituto Nacional de Salud	Laboratorio de Infecciones Respiratorias Agudas	Eduardo Juscamayta Lopez et al
EPI_ISL_540987	2020-03-20	Laboratorio de Referencia Nacional de Virus Respiratorios, Instituto Nacional de Salud Peru	Laboratorio de Genómica Microbiana, Universidad Peruana Cayetano Heredia	Pablo Tsukayama et al
EPI_ISL_536549	2020-03-20	Instituto Nacional de Salud	Laboratorio de Infecciones Respiratorias Agudas	Eduardo Juscamayta Lopez et al
EPI_ISL_428885	2020-03-20	State Research Center of Virology and Biotechnology VECTOR, Department of Collection of Microorganisms	State Research Center of Virology and Biotechnology VECTOR, Department of Collection of Microorganisms	Oleg V. Pyankov et al
EPI_ISL_420074	2020-03-20	Institut Pasteur Dakar	Institut Pasteur de Dakar	Ndongo Dia et al
EPI_ISL_498034	2020-03-20	Division of Viral Diseases, Center for Laboratory Control of Infectious Diseases, Korea Centers for Diseases Control and Prevention	Division of Viral Diseases, Center for Laboratory Control of Infectious Diseases, Korea Centers for Diseases Control and Prevention	Jeong-Min Kim et al
EPI_ISL_498035	2020-03-20	Division of Viral Diseases, Center for Laboratory Control of Infectious Diseases, Korea Centers for Diseases Control and Prevention	Division of Viral Diseases, Center for Laboratory Control of Infectious Diseases, Korea Centers for Diseases Control and Prevention	Jeong-Min Kim et al
EPI_ISL_455593	2020-03-20	National Institute of Health. Department of medical Sciences, Ministry of Public Health, Thailand	National Institute of Health. Department of medical Sciences, Ministry of Public Health, Thailand	Pilailuk et al
EPI_ISL_462459	2020-03-21	Clinical Center, University of Sarajevo	Charite Universitatsmedizin Berlin, Institute of Virology	Victor M Corman et al
EPI_ISL_476218	2020-03-21	DB Diagnósticos do Brasil	Instituto de Medicina Tropical da Univesidade de São Paulo	Samples: Nelson Gaburo Jr et al
EPI_ISL_435676	2020-03-21	National Virology Reference Laboratory	National Public Health Laboratory, National Centre for Infectious Diseases	Mak Tze Minn et al
EPI_ISL_435677	2020-03-21	National Virology Reference Laboratory	National Public Health Laboratory, National Centre for Infectious Diseases	Mak Tze Minn et al

EPI_ISL_480297	2020-03-21	National Reference Laboratory "Influenza and acute respiratory diseases"	NRL-HIV	Ivan Ivanov et al
EPI_ISL_682298	2020-03-21	Respiratory virus Laboratory, Chinese Academy of Medical Science	Respiratory virus Laboratory, Chinese Academy of Medical Science	Li et al
EPI_ISL_445302	2020-03-21	INSTITUTO MEDICO LEGAL	Instituto de Salud Publica de Chile	Andrés E Castillo et al
EPI_ISL_445367	2020-03-21	ASISTENCIA PUBLICA DR.ALEJANDRO DEL RIO	Instituto de Salud Publica de Chile	Andrés E Castillo et al
EPI_ISL_480324	2020-03-21	Hospital Nacional de Niños	Charité Virology-University of Costa Rica	Andres Moreira-Soto et al
EPI_ISL_512658	2020-03-21	Area De Salud Orotina-San Mateo [Orotina/Alajuela]	Inciensa, Instituto Costarricense de Investigación y Enseñanza en Nutrición y Salud	Francisco Duarte et al
EPI_ISL_471159	2020-03-21	MRCG at LSHTM Genomics lab	MRCG at LSHTM Genomics lab	Sesay et al et al
EPI_ISL_428856	2020-03-21	MRCG at LSHTM Genomics Lab	MRCG at LSHTM Genomics lab	Sesay et al et al
EPI_ISL_509703	2020-03-21	Guatemala Ministry of Public Health	Pathogen Discovery, Respiratory Viruses Branch, Division of Viral Diseases, Centers for Disease Control and Prevention	Ying Tao et al
EPI_ISL_435426	2020-03-21	Virological Research Group, Szentágothai Research Centre	Bioinformatics Research Group, Szentágothai Research Centre	Péter Urbán et al
EPI_ISL_457834	2020-03-21	National Public Health Laboratory	KEMRI-Wellcome Trust Research Programme/KEMRI-CGMR-C Kilifi	Githinji G. et al 2020 et al
EPI_ISL_459975	2020-03-21	Institut Pasteur du Maroc	Institut Pasteur du Maroc	Marion Barbet et al
EPI_ISL_568516	2020-03-21	Laboratorio de Referencia Nacional de Virus Respiratorios, Instituto Nacional de Salud Peru	Laboratorio de Genómica Microbiana, Universidad Peruana Cayetano Heredia	Pablo Tsukayama et al
EPI_ISL_430456	2020-03-21	Rizal Medical Center	Research Institute for Tropical Medicine	Medado et al
EPI_ISL_468134	2020-03-21	[Romania, Bucharest] National Institute for Infectious Diseases “Prof. Dr. Matei Balș”	[Romania, Bucharest] National Institute for Infectious Diseases “Prof. Dr. Matei Balș”	Leontina Banica et al
EPI_ISL_439063	2020-03-21	West of Scotland Specialist Virology Centre, NHSGGC / MRC-University of Glasgow Centre for Virus Research	COVID-19 Genomics UK (COG-UK) Consortium	Ana da Silva Filipe et al
EPI_ISL_537988	2020-03-21	Servicio de Microbiología. Hospital General Universitario de Castellón	SeqCOVID-SPAIN consortium/IBV(CSIC)	Rosario Moreno et al
EPI_ISL_457952	2020-03-21	Laboratorio de Biología Molecular Asociación Española Primera en Salud	Departments of Pathology and Medicine, New York University School of Medicine	Maria Victoria Elizondo et al
EPI_ISL_420598	2020-03-22	Servicio Virosis Respiratorias-Departamento Virología-INEI	Instituto Nacional Enfermedades Infecciosas C.G.Malbran	Baumeister E. et al
EPI_ISL_547449	2020-03-22	Dutch COVID-19 response team	National Institute for Public Health and the Environment (RIVM)	Adam Meijer et al
EPI_ISL_438084	2020-03-22	Center for Virology, Medical University of Vienna	Bergthaler laboratory, CeMM Research Center for Molecular Medicine of the Austrian Academy of Sciences	Alexandra Popa et al
EPI_ISL_427298	2020-03-22	Instituto Oswaldo Cruz FIOCRUZ - Laboratory of Respiratory Viruses and Measles (LVRS)	Instituto Oswaldo Cruz FIOCRUZ - Laboratory of Respiratory Viruses and Measles (LVRS)	Paola Resende et al
EPI_ISL_429702	2020-03-22	Central Public Health Laboratory/Octávio Magalhães Institute (IOM) from the Ezequiel Dias Foundation (FUNED)	Instituto Octávio Magalhães / Fundação Ezequiel Dias (IOM/Funed)	Talita Adelino et al
EPI_ISL_729811	2020-03-22	Laboratorio Central de Saude Publica do Estado do Rio Grande do Sul (LACEN-RS)	Laboratory of Respiratory Viruses and Measles, Oswaldo Cruz Institute, FIOCRUZ	Paola Resende et al
EPI_ISL_463742	2020-03-22	Department of Molecular Virology, Cyprus Institute of Neurology and Genetics	Department of Molecular Virology, Cyprus Institute of Neurology and Genetics	Jan Richter et al
EPI_ISL_420035	2020-03-22	Viral Respiratory Lab, National Institute for Biomedical Research (INRB)	Pathogen Sequencing Lab, National Institute for Biomedical Research (INRB)	Placide Mbala-Kingebeni et al
EPI_ISL_420838	2020-03-22	Viral Respiratory Lab, National Institute for Biomedical Research (INRB)	Pathogen Sequencing Lab, National Institute for Biomedical Research (INRB)	Placide Mbala-Kingebeni et al
EPI_ISL_420841	2020-03-22	Viral Respiratory Lab, National Institute for Biomedical Research (INRB)	Pathogen Sequencing Lab, National Institute for Biomedical Research (INRB)	Placide Mbala-Kingebeni et al
EPI_ISL_574293	2020-03-22	Instituto Nacional de Investigación en Salud Pública	Instituto Nacional de Investigación en Salud Publica	Leandro Patino Patino et al
EPI_ISL_539573	2020-03-22	Centre de Recherches Medicales de Lambarene (CERMEL)	Department of Emerging Infectious Diseases, Institute of Tropical Medicine, Nagasaki University	Haruka Abe et al
EPI_ISL_613435	2020-03-22	Institut Pasteur de la Guadeloupe	Institut Pasteur de la Guadeloupe	Marion Barbet et al

EPI_ISL_435045	2020-03-22	Laboratory of Applied Genetics	RSE "National Center for Biotechnology"	Shevtsov et al
EPI_ISL_486425	2020-03-22	Latvijas Infektoloģijas centrs	Latvian Biomedical Research and Study Centre	Ivars Silamiķelis et al
EPI_ISL_486426	2020-03-22	Latvijas Infektoloģijas centrs	Latvian Biomedical Research and Study Centre	Ivars Silamiķelis et al
EPI_ISL_508863	2020-03-22	Virology Unit, Institut Pasteur de Madagascar	Virology Unit, Institut Pasteur de Madagascar	Christian Ranaivoson et al
EPI_ISL_430843	2020-03-22	Bethany Hospital	Research Institute for Tropical Medicine	Medado et al
EPI_ISL_468135	2020-03-22	[Romania, Bucharest] National Institute for Infectious Diseases “Prof. Dr. Matei Balș”	[Romania, Bucharest] National Institute for Infectious Diseases “Prof. Dr. Matei Balș”	Leontina Banica et al
EPI_ISL_436400	2020-03-22	Servicio de Microbiologia. Hospital Clinico Universitario de Valencia	Sequencing and Bioinformatics Service and Molecular Epidemiology Research Group. FISABIO-Public Health	Vicente Soriano Chirona et al
EPI_ISL_528323	2020-03-22	University Hospital Basel, Clinical Virology	University Hospital Basel, Clinical Bacteriology	Madlen Stange et al
EPI_ISL_476200	2020-03-23	DB Diagnósticos do Brasil	Instituto de Medicina Tropical da Univesidade de São Paulo	Samples: Nelson Gaburo Jr et al
EPI_ISL_427296	2020-03-23	Instituto Oswaldo Cruz FIOCRUZ - Laboratory of Respiratory Viruses and Measles (LVRS)	Instituto Oswaldo Cruz FIOCRUZ - Laboratory of Respiratory Viruses and Measles (LVRS)	Paola Resende et al
EPI_ISL_427297	2020-03-23	Instituto Oswaldo Cruz FIOCRUZ - Laboratory of Respiratory Viruses and Measles (LVRS)	Instituto Oswaldo Cruz FIOCRUZ - Laboratory of Respiratory Viruses and Measles (LVRS)	Paola Resende et al
EPI_ISL_470584	2020-03-23	Hermes Pardini	Bioinformatics Laboratory / LNCC	Alexandra Gerber et al
EPI_ISL_470615	2020-03-23	Laboratorio de Virologia Molecular / UFRJ	Bioinformatics Laboratory / LNCC	Alexandra Gerber et al
EPI_ISL_476450	2020-03-23	Hospital da Clínicas da Faculdade de Medicina da Universidade de São Paulo	Instituto de Medicina Tropical da Univesidade de São Paulo	Samples: Ingra Morales Claro et al
EPI_ISL_497744	2020-03-23	Instituto Nacional de Salud, Bogotá, Colombia	Instituto Nacional de Salud, Bogotá, Colombia	Katherine Laiton-Donato et al
EPI_ISL_428901	2020-03-23	State Research Center of Virology and Biotechnology VECTOR, Department of Collection of Microorganisms	State Research Center of Virology and Biotechnology VECTOR, Department of Collection of Microorganisms	Sergey A. Bodnev et al
EPI_ISL_463744	2020-03-23	Department of Molecular Virology, Cyprus Institute of Neurology and Genetics	Department of Molecular Virology, Cyprus Institute of Neurology and Genetics	Jan Richter et al
EPI_ISL_574294	2020-03-23	Instituto Nacional de Investigacion en Salud Pública	Instituto Nacional de Investigación en Salud Pública	Leandro Patino Patino et al
EPI_ISL_681702	2020-03-23	Zurita & Zurita Laboratorios	Zurita & Zurita Laboratorios	Gabriela Sevillano et al
EPI_ISL_517449	2020-03-23	Liverpool Clinical Laboratories	COVID-19 Genomics UK (COG-UK) Consortium	Sam Haldenby et al
EPI_ISL_723090	2020-03-23	Institute of Medical Genetics and Applied Genomics	Institute of Medical Genetics and Applied Genomics	Caspar Gross et al
EPI_ISL_528927	2020-03-23	Ospedale "Giuseppe Mazzini"-Teramo	Istitituto Zooprofilattico Sperimentale dell'Abruzzo e Molise "G.Caporale"	Lorusso A et al
EPI_ISL_528928	2020-03-23	Ospedale "Giuseppe Mazzini"-Teramo	Istitituto Zooprofilattico Sperimentale dell'Abruzzo e Molise "G.Caporale"	Lorusso A et al
EPI_ISL_437089	2020-03-23	Latvijas Infektoloģijas centrs	Latvian Biomedical Research and Study Centre	Ivars Silamiķelis et al
EPI_ISL_501179	2020-03-23	Department of Medical Microbiology, University Malaya Medical Centre	Department of Medical Microbiology, Faculty of Medicine, University of Malaya	Yoong Min CHONG et al
EPI_ISL_422928	2020-03-23	Dutch COVID-19 response team	Erasmus Medical Center	Bas Oude Munnink et al
EPI_ISL_422933	2020-03-23	Dutch COVID-19 response team	Erasmus Medical Center	Bas Oude Munnink et al
EPI_ISL_579156	2020-03-23	LabPLUS	Institute of Environmental Science and Research (ESR)	Xiaoyun Ren et al
EPI_ISL_527874	2020-03-23	Nigeria Centre for Disease Control (NCDC)	African Centre of Excellence for Genomics of Infectious Diseases (ACEGID), Redeemer's University, Ede, Osun State, Nigeria	Oluniyi P.E. et al et al
EPI_ISL_430845	2020-03-23	Pasig City General Hospital	Research Institute for Tropical Medicine	Medado et al
EPI_ISL_528364	2020-03-23	University Hospital Basel, Clinical Virology	University Hospital Basel, Clinical Bacteriology	Madlen Stange et al
EPI_ISL_457953	2020-03-23	Laboratorio de Biología Molecular Asociación Española Primera en Salud	Departments of Pathology and Medicine, New York University School of Medicine	Maria Victoria Elizondo et al
EPI_ISL_457954	2020-03-23	Laboratorio de Biología Molecular Asociación Española Primera en Salud	Departments of Pathology and Medicine, New York University School of Medicine	Maria Victoria Elizondo et al
EPI_ISL_583622	2020-03-24	Pathologie-Labor Dr. Obrist- Dr. Brunhuber	Bergthaler laboratory, CeMM Research Center for Molecular Medicine of the Austrian Academy of Sciences	Alexandra Popa et al

EPI_ISL_524797	2020-03-24	Evandro Chagas Institute	Evandro Chagas Institute	Santos et al
EPI_ISL_636836	2020-03-24	Laboratório de Imunofarmacologia - Instituto Oswaldo Cruz	Laboratório de Imunofarmacologia - Instituto Oswaldo Cruz	Souza et al
EPI_ISL_476210	2020-03-24	DB Diagnósticos do Brasil	Instituto de Medicina Tropical da Univesidade de São Paulo	Samples: Nelson Gaburo Jr et al
EPI_ISL_476278	2020-03-24	DB Diagnósticos do Brasil	Instituto de Medicina Tropical da Univesidade de São Paulo	Samples: Nelson Gaburo Jr et al
EPI_ISL_515555	2020-03-24	Hospital Geral de Vila Nova Cachoeirinha	Instituto Adolfo Lutz, Interdiciplinary Procedures Center, Strategic Laboratory	Claudio Tavares Sacchi et al
EPI_ISL_476486	2020-03-24	Hospital da Clínicas da Faculdade de Medicina da Universidade de São Paulo	Instituto de Medicina Tropical da Univesidade de São Paulo	Samples: Ingra Morales Claro et al
EPI_ISL_476489	2020-03-24	Hospital da Clínicas da Faculdade de Medicina da Universidade de São Paulo	Instituto de Medicina Tropical da Univesidade de São Paulo	Samples: Ingra Morales Claro et al
EPI_ISL_613707	2020-03-24	Laboratory of Molecular Biology, Blood Center of Ribeirão Preto	Laboratory of Molecular Biology, Blood Center of Ribeirão Preto, Faculty of Medicine of Ribeirão Preto, University of São Paulo	Svetoslav N Slavov et al
EPI_ISL_513313	2020-03-24	Public Health, United States Air Force School of Aerospace Medicine	Public Health, United States Air Force School of Aerospace Medicine	Fries et al
EPI_ISL_420636	2020-03-24	Respiratory Virus Unit, Microbiology Services Colindale, Public Health England	Respiratory Virus Unit, Microbiology Services Colindale, Public Health England	Monica Galiano et al
EPI_ISL_648318	2020-03-24	Laboratorio de Investigaciones de Baney	University Hospital Basel, Clinical Bacteriology	Carlos Cortes et al
EPI_ISL_444610	2020-03-24	U.S. Naval Medical Research Center Biological Defense Research Directorate	U.S. Naval Medical Research Center Biological Defense Research Directorate	Voegtly et al
EPI_ISL_444999	2020-03-24	Naval Health Research Center	Naval Medical Research Center Biological Defense Research Directorate	Logan Voegtly et al
EPI_ISL_457847	2020-03-24	KEMRI-CGMR-C	KEMRI-Wellcome Trust Research Programme/KEMRI-CGMR-C Kilifi	Githinji G. et al 2020 et al
EPI_ISL_457848	2020-03-24	KEMRI-CGMR-C	KEMRI-Wellcome Trust Research Programme/KEMRI-CGMR-C Kilifi	Githinji G. et al 2020 et al
EPI_ISL_437090	2020-03-24	Latvijas Infektoloģijas centrs	Latvian Biomedical Research and Study Centre	Ivars Silamiķelis et al
EPI_ISL_428917	2020-03-24	State Research Center of Virology and Biotechnology VECTOR, Department of Collection of Microorganisms	State Research Center of Virology and Biotechnology VECTOR, Department of Collection of Microorganisms	Sergey A. Bodnev et al
EPI_ISL_574498	2020-03-24	National Public Health Laboratory, National Centre for Infectious Diseases	National Public Health Laboratory, National Centre for Infectious Diseases	Tze Minn Mak et al
EPI_ISL_467444	2020-03-24	Molecular Diagnostics Services (MDS)	KRISP, KZN Research Innovation and Sequencing Platform	Giandhari J et al
EPI_ISL_506972	2020-03-24	Division of Viral Diseases, Center for Laboratory Control of Infectious Diseases, Korea Centers for Diseases Control and Prevention	Division of Viral Diseases, Center for Laboratory Control of Infectious Diseases, Korea Centers for Diseases Control and Prevention	Jeong-Min Kim et al
EPI_ISL_458286	2020-03-24	unknown	Bundeswehr Institute of Microbiology	Handrick et al
EPI_ISL_583573	2020-03-25	Austrian Agency for Health and Food Safety (AGES)	Bergthaler laboratory, CeMM Research Center for Molecular Medicine of the Austrian Academy of Sciences	Alexandra Popa et al
EPI_ISL_476826	2020-03-25	Laboratoire des Fièvres Hémorragiques Virales du Benin	Charité-Universitätsmedizin Berlin	Yadouleton et al
EPI_ISL_462465	2020-03-25	Clinical Center, University of Sarajevo	Charite Universitatsmedizin Berlin, Institute of Virology	Victor M Corman et al
EPI_ISL_470617	2020-03-25	Laboratorio de Virologia Molecular / UFRJ	Bioinformatics Laboratory / LNCC	Alexandra Gerber et al
EPI_ISL_456076	2020-03-25	LACEN RJ - Laboratório Central de Saúde Pública Noel Nutels	Laboratory of Respiratory Viruses and Measles, Oswaldo Cruz Institute, FIOCRUZ	Paola Resende et al
EPI_ISL_456077	2020-03-25	LACEN RJ - Laboratório Central de Saúde Pública Noel Nutels	Laboratory of Respiratory Viruses and Measles, Oswaldo Cruz Institute, FIOCRUZ	Paola Resende et al
EPI_ISL_427303	2020-03-25	Instituto Oswaldo Cruz FIOCRUZ - Laboratory of Respiratory Viruses and Measles (LVRS)	Instituto Oswaldo Cruz FIOCRUZ - Laboratory of Respiratory Viruses and Measles (LVRS)	Paola Resende et al
EPI_ISL_729813	2020-03-25	Laboratorio Central de Saude Publica do Estado do Rio Grande do Sul (LACEN-RS)	Laboratory of Respiratory Viruses and Measles, Oswaldo Cruz Institute, FIOCRUZ	Paola Resende et al
EPI_ISL_729814	2020-03-25	Laboratorio Central de Saude Publica do Estado do Rio Grande do Sul (LACEN-RS)	Laboratory of Respiratory Viruses and Measles, Oswaldo Cruz Institute, FIOCRUZ	Paola Resende et al

EPI_ISL_523982	2020-03-25	Hospital do Servidor Público Estadual Francisco Morato de Oliveira	Instituto Adolfo Lutz, Interdiciplinary Procedures Center, Strategic Laboratory	Claudio Tavares Sacchi et al
EPI_ISL_515528	2020-03-25	Hospital Sao Paulo de Ensino da Unifesp	Instituto Adolfo Lutz, Interdiciplinary Procedures Center, Strategic Laboratory	Claudio Tavares Sacchi et al
EPI_ISL_536399	2020-03-25	Laboratory of Immunovirology. Universidad de Antioquia	Instituto Nacional de Salud - Unidad de Secuenciación y Genómica	Wbeimar Aguilar-Jimenez et al
EPI_ISL_434539	2020-03-25	Area de Salud Orotina	Inciensa, Instituto Costarricense de Investigación y Enseñanza en Nutrición y Salud	Duarte et al
EPI_ISL_463743	2020-03-25	Department of Molecular Virology, Cyprus Institute of Neurology and Genetics	Department of Molecular Virology, Cyprus Institute of Neurology and Genetics	Jan Richter et al
EPI_ISL_420854	2020-03-25	Viral Respiratory Lab, National Institute for Biomedical Research (INRB)	Pathogen Sequencing Lab, National Institute for Biomedical Research (INRB)	Placide Mbala-Kingebeni et al
EPI_ISL_422390	2020-03-25	NMIMR, Department of Virology	WACCBIP, University of Ghana	Joyce M. Ngoi et al
EPI_ISL_435427	2020-03-25	Virological Research Group, Szentágothai Research Centre	Bioinformatics Research Group, Szentágothai Research Centre	Péter Urbán et al
EPI_ISL_435428	2020-03-25	Virological Research Group, Szentágothai Research Centre	Bioinformatics Research Group, Szentágothai Research Centre	Péter Urbán et al
EPI_ISL_457825	2020-03-25	Army Medical Research Center - Scientific Department	Army Medical and Veterinary Research Center	Silvia Fillo et al
EPI_ISL_454571	2020-03-25	National Center of Expertise	National Center for Expertise, National Center for Biotechnology, Kazakhstan	Abdaliyev Askar et al
EPI_ISL_454572	2020-03-25	National Center of Expertise	National Center for Expertise, Kazakhstan National Center for Biotechnology, Kazakhstan	Abdaliyev Askar et al
EPI_ISL_435046	2020-03-25	Laboratory of Applied Genetics	RSE "National Center for Biotechnology"	Shevtsov et al
EPI_ISL_435047	2020-03-25	Laboratory of Applied Genetics	RSE "National Center for Biotechnology"	Shevtsov et al
EPI_ISL_467666	2020-03-25	Virology lab, NIC, NCCD, Ulaanbaatar, Mongolia	National Centre for Communicable Deseases (NCCD)	Naranzul Ts et al
EPI_ISL_579462	2020-03-25	Canterbury Health Laboratories	Institute of Environmental Science and Research (ESR)	Xiaoyun Ren et al
EPI_ISL_527876	2020-03-25	Nigeria Centre for Disease Control (NCDC)	African Centre of Excellence for Genomics of Infectious Diseases (ACEGID), Redeemer's University, Ede, Osun State, Nigeria	Oluniyi P.E. et al et al
EPI_ISL_527880	2020-03-25	Nigeria Centre for Disease Control (NCDC)	African Centre of Excellence for Genomics of Infectious Diseases (ACEGID), Redeemer's University, Ede, Osun State, Nigeria	Oluniyi P.E. et al et al
EPI_ISL_457703	2020-03-25	Oman-NIC	Department of Microbiology and Immunology- SQUH	Fahad Zadjali et al
EPI_ISL_468142	2020-03-25	[Romania, Bucharest] National Institute for Infectious Diseases “Prof. Dr. Matei Balș”	[Romania, Bucharest] National Institute for Infectious Diseases “Prof. Dr. Matei Balș”	Leontina Banica et al
EPI_ISL_468144	2020-03-25	[Romania, Bucharest] National Institute for Infectious Diseases “Prof. Dr. Matei Balș”	[Romania, Bucharest] National Institute for Infectious Diseases “Prof. Dr. Matei Balș”	Leontina Banica et al
EPI_ISL_427338	2020-03-25	WHO National Influenza Centre Russian Federation	WHO National Influenza Centre Russian Federation	Andrey Komissarov et al
EPI_ISL_613447	2020-03-25	Institut Pasteur de la Guadeloupe	Institut Pasteur de la Guadeloupe	Marion Barbet et al
EPI_ISL_437497	2020-03-25	Pathogen Genomics Lab King Abdullah University of Science and Technology(KAUST)	Pathogen Genomics Lab King Abdullah University of Science and Technology(KAUST)	Sara Mfarrej et al
EPI_ISL_467443	2020-03-25	NHLS-IALCH	KRISP, KZN Research Innovation and Sequencing Platform	Giandhari J et al
EPI_ISL_447257	2020-03-25	TSGH-CP molecular lab	TSGH-CP molecular lab	Cherng-Lih Perng et al
EPI_ISL_451183	2020-03-25	Uganda Virus Research Institute	MRC/UVRI & LSHTM Uganda Research Unit	Dan Lule Bugembe et al
EPI_ISL_451196	2020-03-25	Uganda Virus Research Institute	MRC/UVRI & LSHTM Uganda Research Unit	Dan Lule Bugembe et al
EPI_ISL_457956	2020-03-25	Laboratorio de Biología Molecular Asociación Española Primera en Salud	Departments of Pathology and Medicine, New York University School of Medicine	Maria Victoria Elizondo et al
EPI_ISL_457958	2020-03-25	Laboratorio de Biología Molecular Asociación Española Primera en Salud	Departments of Pathology and Medicine, New York University School of Medicine	Maria Victoria Elizondo et al
EPI_ISL_430795	2020-03-26	Laboratorio de Virología del Hospital de Niños Dr. Ricardo Gutierrez	Área de Secuenciación del Laboratorio de Virología del Hospital de Niños	Nabaes Jodar et al
EPI_ISL_527016	2020-03-26	Area of Virology, Serology and Virology Division (SAViD), New South Wales Health Pathology Randwick	Area of Virology, Serology and Virology Division (SAViD), New South Wales Health Pathology Randwick	Rawlinson et al
EPI_ISL_600554	2020-03-26	Institute of Epidemiology Disease Control And Research	Institute for Developing Science and Health Initiatives	Lauren Cowley et al

EPI_ISL_600562	2020-03-26	Institute of Epidemiology Disease Control And Research	Institute for Developing Science and Health Initiatives	Lauren Cowley et al
EPI_ISL_541346	2020-03-26	LACEN/PR	Laboratory of Respiratory Viruses and Measles, Oswaldo Cruz Institute, FIOCRUZ	Paola Resende et al
EPI_ISL_470618	2020-03-26	Laboratorio de Virologia Molecular / UFRJ	Bioinformatics Laboratory / LNCC	Alexandra Gerber et al
EPI_ISL_427304	2020-03-26	Instituto Oswaldo Cruz FIOCRUZ - Laboratory of Respiratory Viruses and Measles (LVRS)	Instituto Oswaldo Cruz FIOCRUZ - Laboratory of Respiratory Viruses and Measles (LVRS)	Paola Resende et al
EPI_ISL_476297	2020-03-26	DB Diagnósticos do Brasil	Instituto de Medicina Tropical da Univesidade de São Paulo	Samples: Nelson Gaburo Jr et al
EPI_ISL_468312	2020-03-26	Hospital Municipal Dr Ignacio Proenca de Gouvea	Instituto Adolfo Lutz, Interdiciplinary Procedures Center, Strategic Laboratory	Claudio Tavares Sacchi et al
EPI_ISL_468314	2020-03-26	CTA Centro de Testagem e Aconselhamento	Instituto Adolfo Lutz, Interdiciplinary Procedures Center, Strategic Laboratory	Claudio Tavares Sacchi et al
EPI_ISL_476244	2020-03-26	Hospital da Clínicas da Faculdade de Medicina da Universidade de São Paulo	Instituto de Medicina Tropical da Univesidade de São Paulo	Samples: Ingra Morales Claro et al
EPI_ISL_498158	2020-03-26	Instituto Nacional de Salud, Bogotá, Colombia	Instituto Nacional de Salud, Bogotá, Colombia	Katherine Laiton-Donato et al
EPI_ISL_444008	2020-03-26	PHE South West Regional Laboratory, National Infection Service	Wellcome Sanger Institute for the COVID-19 Genomics UK (COG-UK) consortium	Stephanie Hutchings et al
EPI_ISL_444998	2020-03-26	Naval Health Research Center	Naval Medical Research Center Biological Defense Research Directorate	Logan Voegtly et al
EPI_ISL_437190	2020-03-26	RS Pondok Indah Hospital – Pondok Indah	Eijkman Institute for Molecular Biology, Ministry of Research and Technology/National Agency for Research and Innovation	Edison Johar et al
EPI_ISL_442044	2020-03-26	Kawsar Human Genetic Research Center	Kawsar Human Genetic Research Center	Mohammad Ali Khosravi et al
EPI_ISL_677635	2020-03-26	Virology Unit, Institut Pasteur de Madagascar	Virology Unit, Institut Pasteur de Madagascar	Christian Ranaivoson et al
EPI_ISL_677636	2020-03-26	Virology Unit, Institut Pasteur de Madagascar	Virology Unit, Institut Pasteur de Madagascar	Christian Ranaivoson et al
EPI_ISL_434555	2020-03-26	National Institutes of Health, University of the Philippines Manila	Philippine Genome Center	Carlo M. Lapid et al
EPI_ISL_470896	2020-03-26	Russian State Collection of Viruses	Pathogenic Microorganisms Variability Laboratory	Alexey Shchetinin et al
EPI_ISL_506980	2020-03-26	Division of Viral Diseases, Center for Laboratory Control of Infectious Diseases, Korea Centers for Diseases Control and Prevention	Division of Viral Diseases, Center for Laboratory Control of Infectious Diseases, Korea Centers for Diseases Control and Prevention	Jeong-Min Kim et al
EPI_ISL_450458	2020-03-26	Stanford clinical virology lab	Chan-Zuckerberg Biohub	Benjamin Pinksy et al
EPI_ISL_451184	2020-03-26	Uganda Virus Research Institute	MRC/UVRI & LSHTM Uganda Research Unit	Dan Lule Bugembe et al
EPI_ISL_457959	2020-03-26	Laboratorio de Biología Molecular Asociación Española Primera en Salud	Departments of Pathology and Medicine, New York University School of Medicine	Maria Victoria Elizondo et al
EPI_ISL_430796	2020-03-27	Departamento de Biología y genética molecular, IACA Laboratorios.	Área de Secuenciación del Laboratorio de Virología del Hospital de Niños	Nabaes Jodar et al
EPI_ISL_430797	2020-03-27	Departamento de Biología y genética molecular, IACA Laboratorios.	Área de Secuenciación del Laboratorio de Virología del Hospital de Niños	Nabaes Jodar et al
EPI_ISL_509713	2020-03-27	Belize Ministry of Health	Pathogen Discovery, Respiratory Viruses Branch, Division of Viral Diseases, Centers for Disease Control and Prevention	Jing Zhang et al
EPI_ISL_462469	2020-03-27	Clinical Center, University of Sarajevo	Charite Universitatsmedizin Berlin, Institute of Virology	Victor M Corman et al
EPI_ISL_470619	2020-03-27	Laboratorio de Virologia Molecular / UFRJ	Bioinformatics Laboratory / LNCC	Alexandra Gerber et al
EPI_ISL_729815	2020-03-27	Laboratorio Central de Saude Publica do Estado do Rio Grande do Sul (LACEN-RS)	Laboratory of Respiratory Viruses and Measles, Oswaldo Cruz Institute, FIOCRUZ	Paola Resende et al
EPI_ISL_541372	2020-03-27	LACEN/SE	Laboratory of Respiratory Viruses and Measles, Oswaldo Cruz Institute, FIOCRUZ	Paola Resende et al
EPI_ISL_426892	2020-03-27	Motol University Hospital	Institute of Applied Biotechnologies a.s.	Petr Brož et al
EPI_ISL_426893	2020-03-27	Motol University Hospital	Institute of Applied Biotechnologies a.s.	Petr Brož et al
EPI_ISL_426895	2020-03-27	Motol University Hospital	Institute of Applied Biotechnologies a.s.	Petr Brož et al
EPI_ISL_539574	2020-03-27	Centre de Recherches Medicales de Lambarene (CERMEL)	Department of Emerging Infectious Diseases, Institute of Tropical Medicine, Nagasaki University	Haruka Abe et al

EPI_ISL_539575	2020-03-27	Centre de Recherches Medicales de Lambarene (CERMEL)	Department of Emerging Infectious Diseases, Institute of Tropical Medicine, Nagasaki University	Haruka Abe et al
EPI_ISL_539576	2020-03-27	Centre de Recherches Medicales de Lambarene (CERMEL)	Department of Emerging Infectious Diseases, Institute of Tropical Medicine, Nagasaki University	Haruka Abe et al
EPI_ISL_515083	2020-03-27	Department of Biochemistry, Cell and Molecular Biology	WACCBIP, University of Ghana	Ngoi et al
EPI_ISL_515182	2020-03-27	Kumasi Centre for Collaborative Research in Tropical Medicine, Kumasi.	Institute of Virology, Charité – Universitätsmedizin Berlin	Augustina Sylverken et al
EPI_ISL_515183	2020-03-27	Kumasi Centre for Collaborative Research in Tropical Medicine, Kumasi.	Institute of Virology, Charité – Universitätsmedizin Berlin	Augustina Sylverken et al
EPI_ISL_501242	2020-03-27	Hellenic Pasteur Institute, National Influenza Reference laboratory of Southern Greece & Unit of Bioinformatics and Applied Genomics	Hellenic Pasteur Institute, National Influenza Reference laboratory of Southern Greece & Unit of Bioinformatics and Applied Genomics	Vasiliki Pogka et al
EPI_ISL_477623	2020-03-27	University of Szeged, Institute of Clinical Microbiology	National Laboratory of Virology, Szentágothai Research Centre	Endre Gábor Tóth et al
EPI_ISL_437191	2020-03-27	RS Pondok Indah Hospital – Pondok Indah	Eijkman Institute for Molecular Biology, Ministry of Research and Technology/National Agency for Research and Innovation	Edison Johar et al
EPI_ISL_455413	2020-03-27	Nigeria Centre for Disease Control (NCDC)	African Centre of Excellence for Genomics of Infectious Diseases (ACEGID), Redeemer's University, Ede, Osun State, Nigeria	Oluniyi P.E. et al
EPI_ISL_511523	2020-03-27	Instituto Nacional de Saude (INSA)	Instituto Nacional de Saude (INSA)	Borges et al et al
EPI_ISL_428913	2020-03-27	State Research Center of Virology and Biotechnology VECTOR, Department of Collection of Microorganisms	State Research Center of Virology and Biotechnology VECTOR, Department of Collection of Microorganisms	Oleg V. Pyankov et al
EPI_ISL_467441	2020-03-27	NHLS-IALCH	KRISP, KZN Research Innovation and Sequencing Platform	Giandhari J et al
EPI_ISL_467442	2020-03-27	NHLS-IALCH	KRISP, KZN Research Innovation and Sequencing Platform	Giandhari J et al
EPI_ISL_512872	2020-03-27	Ramathibodi Hospital	COVID-19 Network Investigations (CONI) Alliance	Elizabeth Batty et al
EPI_ISL_463005	2020-03-27	unknown	Clinical virology	Fares et al
EPI_ISL_437311	2020-03-27	Ministry of Health Turkey	Ministry of Health Turkey	Fatma Bayrakdar et al
EPI_ISL_437313	2020-03-27	Ministry of Health Turkey	Ministry of Health Turkey	Fatma Bayrakdar et al
EPI_ISL_451185	2020-03-27	Uganda Virus Research Institute	MRC/UVRI & LSHTM Uganda Research Unit	Dan Lule Bugembe et al
EPI_ISL_454956	2020-03-27	Wuhan Chain Medical Labs (CMLabs)	State Key Laboratory of Biotherapy of Sichuan University	Baowen Du et al
EPI_ISL_560386	2020-03-28	National Health Laboratory	Botswana Institute for Technology Research and innovation	Kefentse Arnold Tumedi et al
EPI_ISL_476281	2020-03-28	DB Diagnósticos do Brasil	Instituto de Medicina Tropical da Univesidade de São Paulo	Samples: Nelson Gaburo Jr et al
EPI_ISL_541345	2020-03-28	LACEN/PR	Laboratory of Respiratory Viruses and Measles, Oswaldo Cruz Institute, FIOCRUZ	Paola Resende et al
EPI_ISL_476282	2020-03-28	DB Diagnósticos do Brasil	Instituto de Medicina Tropical da Univesidade de São Paulo	Samples: Nelson Gaburo Jr et al
EPI_ISL_634833	2020-03-28	Laboratoire de virologie, CHU de Grenoble - CS 10217 - 38043 Grenoble cedex 25	CNR Virus des Infections Respiratoires - France SUD	Antonin Bal et al
EPI_ISL_515085	2020-03-28	Department of Biochemistry, Cell and Molecular Biology	WACCBIP, University of Ghana	Ngoi et al
EPI_ISL_451310	2020-03-28	Hellenic Pasteur Institute, National Influenza Reference laboratory of Southern Greece & Unit of Bioinformatics and Applied Genomics	Hellenic Pasteur Institute, National Influenza Reference laboratory of Southern Greece & Unit of Bioinformatics and Applied Genomics	Vasiliki Pogka et al
EPI_ISL_568693	2020-03-28	RS Freeport Tembagapura	Eijkman Institute for Molecular Biology, Ministry of Research and Technology/National Agency for Research and Innovation	Frilasita A Yudhaputri et al
EPI_ISL_594186	2020-03-28	Department of Pathology, School of Medicine, Imam Khomeini Hospital, Tehran University of Medical Sciences	Genetics Research Center, University of Social Welfare and Rehabilitation Sciences	Zohreh Fattahi et al
EPI_ISL_457997	2020-03-28	Oman-NIC	Oman-NIC	Samira Al-Maruqi et al
EPI_ISL_434558	2020-03-28	National Institutes of Health, University of the Philippines Manila	Philippine Genome Center	Carlo M. Lapid et al
EPI_ISL_454172	2020-03-28	unknown	Instituto Nacional de Saude (INSA)	Borges et al et al
EPI_ISL_454311	2020-03-28	unknown	Instituto Nacional de Saude (INSA)	Borges et al et al
EPI_ISL_468145	2020-03-28	[Romania, Bucharest] National Institute for Infectious Diseases “Prof. Dr. Matei Balș”	[Romania, Bucharest] National Institute for Infectious Diseases “Prof. Dr. Matei Balș”	Leontina Banica et al

EPI_ISL_613448	2020-03-28	Institut Pasteur de la Guadeloupe	Institut Pasteur de la Guadeloupe	Marion Barbet et al
EPI_ISL_613449	2020-03-28	Institut Pasteur de la Guadeloupe	Institut Pasteur de la Guadeloupe	Marion Barbet et al
EPI_ISL_613452	2020-03-28	Institut Pasteur de la Guadeloupe	Institut Pasteur de la Guadeloupe	Marion Barbet et al
EPI_ISL_429050	2020-03-28	UCSF Clinical Microbiology Laboratory	Chan-Zuckerberg Biohub	CZB Cliahub Consortium et al
EPI_ISL_434133	2020-03-28	Washington State Department of Health	Seattle Flu Study	Chu et al et al
EPI_ISL_437806	2020-03-28	UW Virology Lab	UW Virology Lab	Pavitra Roychoudhury et al
EPI_ISL_457961	2020-03-28	Laboratorio de Biología Molecular Asociación Española Primera en Salud	Departments of Pathology and Medicine, New York University School of Medicine	Maria Victoria Elizondo et al
EPI_ISL_458203	2020-03-29	KU Leuven, Rega Institute, Clinical and Epidemiological Virology	KU Leuven, Rega Institute, Clinical and Epidemiological Virology	Tony Wawina-Bokalanga et al
EPI_ISL_476283	2020-03-29	DB Diagnósticos do Brasil	Instituto de Medicina Tropical da Univesidade de São Paulo	Samples: Nelson Gaburo Jr et al
EPI_ISL_636838	2020-03-29	Laboratório de Imunofarmacologia - Instituto Oswaldo Cruz	Laboratório de Imunofarmacologia - Instituto Oswaldo Cruz	Souza et al
EPI_ISL_476259	2020-03-29	Hospital da Clínicas da Faculdade de Medicina da Universidade de São Paulo	Instituto de Medicina Tropical da Univesidade de São Paulo	Samples: Ingra Morales Claro et al
EPI_ISL_471554	2020-03-29	Hospital Bosque da Saúde	Instituto Adolfo Lutz, Interdiciplinary Procedures Center, Strategic Laboratory	Claudio Tavares Sacchi et al
EPI_ISL_481640	2020-03-29	Department of Virology and Immunology, University of Helsinki and Helsinki University Hospital, Huslab Finland	Department of Virology, Faculty of Medicine, University of Helsinki, Helsinki, Finland	Teemu Smura et al
EPI_ISL_481648	2020-03-29	Department of Virology and Immunology, University of Helsinki and Helsinki University Hospital, Huslab Finland	Department of Virology, Faculty of Medicine, University of Helsinki, Helsinki, Finland	Teemu Smura et al
EPI_ISL_471158	2020-03-29	MRCG at LSHTM Genomics lab	MRCG at LSHTM Genomics lab	Sesay et al et al
EPI_ISL_451311	2020-03-29	Hellenic Pasteur Institute, National Influenza Reference laboratory of Southern Greece & Unit of Bioinformatics and Applied Genomics	Hellenic Pasteur Institute, National Influenza Reference laboratory of Southern Greece & Unit of Bioinformatics and Applied Genomics	Vasiliki Pogka et al
EPI_ISL_610177	2020-03-29	Department of Health Technology and Informatics, The Hong Kong Polytechnic University	Department of Health Technology and Informatics, The Hong Kong Polytechnic University	Siu et al
EPI_ISL_435430	2020-03-29	Virological Research Group, Szentágothai Research Centre	Bioinformatics Research Group, Szentágothai Research Centre	Péter Urbán et al
EPI_ISL_457843	2020-03-29	National Public Health Laboratory	KEMRI-Wellcome Trust Research Programme/KEMRI-CGMR-C Kilifi	Githinji G. et al 2020 et al
EPI_ISL_455412	2020-03-29	Nigeria Centre for Disease Control (NCDC)	African Centre of Excellence for Genomics of Infectious Diseases (ACEGID), Redeemer's University, Ede, Osun State, Nigeria	Oluniyi P.E. et al
EPI_ISL_596512	2020-03-29	Palestinian Ministry of Health	Molecular Genetics Lab	Nouar Qutob et al
EPI_ISL_451982	2020-03-29	1. ViroGenetics - BSL3 Laboratory of Virology, Małopolska Centre of Biotechnology, Jagiellonian University; 2. II Department of Internal Medicine, Faculty of Medicine, Jagiellonian University Medical College; 3. DIAGNOSTYKA Ltd.	1. ViroGenetics - BSL3 Laboratory of Virology, Małopolska Centre of Biotechnology, Jagiellonian University; 2. II Department of Internal Medicine, Faculty of Medicine, Jagiellonian University Medical College.	Marek Sanak et al
EPI_ISL_451984	2020-03-29	1. ViroGenetics - BSL3 Laboratory of Virology, Małopolska Centre of Biotechnology, Jagiellonian University; 2. II Department of Internal Medicine, Faculty of Medicine, Jagiellonian University Medical College; 3. DIAGNOSTYKA Ltd.	1. ViroGenetics - BSL3 Laboratory of Virology, Małopolska Centre of Biotechnology, Jagiellonian University; 2. II Department of Internal Medicine, Faculty of Medicine, Jagiellonian University Medical College.	Marek Sanak et al
EPI_ISL_451985	2020-03-29	1. ViroGenetics - BSL3 Laboratory of Virology, Małopolska Centre of Biotechnology, Jagiellonian University; 2. II Department of Internal Medicine, Faculty of Medicine, Jagiellonian University Medical College; 3. DIAGNOSTYKA Ltd.	1. ViroGenetics - BSL3 Laboratory of Virology, Małopolska Centre of Biotechnology, Jagiellonian University; 2. II Department of Internal Medicine, Faculty of Medicine, Jagiellonian University Medical College.	Marek Sanak et al
EPI_ISL_613451	2020-03-29	Institut Pasteur de la Guadeloupe	Institut Pasteur de la Guadeloupe	Marion Barbet et al
EPI_ISL_452585	2020-03-29	Servicio de Microbiología y Parasitología clínica. UCEIMP. Hospital Universitario Virgen del Rocío/IBIS/CSIC/US.	SeqCOVID-SPAIN consortium/IBV(CSIC)	Guillermo Martín Gutiérrez et al
EPI_ISL_613462	2020-03-29	Microbiology, Koc University	Microbiology, Koc University	Nurtop et al
EPI_ISL_430799	2020-03-30	Laboratorio de Virología del Hospital de Niños Dr. Ricardo Gutierrez	Área de Secuenciación del Laboratorio de Virología del Hospital de Niños	Nabaes Jodar et al
EPI_ISL_430800	2020-03-30	Laboratorio de Virología del Hospital de Niños Dr. Ricardo Gutierrez	Área de Secuenciación del Laboratorio de Virología del Hospital de Niños	Nabaes Jodar et al

EPI_ISL_639787	2020-03-30	unknown	Public Health Virology Laboratory, Forensic and Scientific Services (PHV-FSS)	Son Nguyen et al. et al
EPI_ISL_483553	2020-03-30	Kingdom of Bahrein Ministry of Health	Erasmus Medical Center	Bas Oude Munnink et al
EPI_ISL_492028	2020-03-30	Child Health Research Foundation	Child Health Research Foundation	Senjuti Saha et al
EPI_ISL_476288	2020-03-30	DB Diagnósticos do Brasil	Instituto de Medicina Tropical da Univesidade de São Paulo	Samples: Nelson Gaburo Jr et al
EPI_ISL_476289	2020-03-30	DB Diagnósticos do Brasil	Instituto de Medicina Tropical da Univesidade de São Paulo	Samples: Nelson Gaburo Jr et al
EPI_ISL_476290	2020-03-30	DB Diagnósticos do Brasil	Instituto de Medicina Tropical da Univesidade de São Paulo	Samples: Nelson Gaburo Jr et al
EPI_ISL_470620	2020-03-30	Laboratorio de Virologia Molecular / UFRJ	Bioinformatics Laboratory / LNCC	Alexandra Gerber et al
EPI_ISL_456071	2020-03-30	Laboratory of Respiratory Viruses and Measles, Oswaldo Cruz Institute, FIOCRUZ	Laboratory of Respiratory Viruses and Measles, Oswaldo Cruz Institute, FIOCRUZ	Paola Resende et al
EPI_ISL_476292	2020-03-30	DB Diagnósticos do Brasil	Instituto de Medicina Tropical da Univesidade de São Paulo	Samples: Nelson Gaburo Jr et al
EPI_ISL_527862	2020-03-30	Hospital Municipal de Urgência	Instituto Adolfo Lutz, Interdiciplinary Procedures Center, Strategic Laboratory	Claudio Tavares Sacchi et al
EPI_ISL_480303	2020-03-30	National Reference Laboratory "Influenza and acute respiratory diseases"	NRL-HIV	Ivan Ivanov et al
EPI_ISL_480307	2020-03-30	National Reference Laboratory "Influenza and acute respiratory diseases"	NRL-HIV	Ivan Ivanov et al
EPI_ISL_498156	2020-03-30	Instituto Nacional de Salud, Bogotá, Colombia	Instituto Nacional de Salud, Bogotá, Colombia	Katherine Laiton-Donato et al
EPI_ISL_454606	2020-03-30	Institute for Public Health	Laboratory for advanced genomics	Filip Rokić et al
EPI_ISL_422563	2020-03-30	Institute of Microbiology Universidad San Francisco de Quito	Institute of Microbiology Universidad San Francisco de Quito	Belen Prado-Vivar et al
EPI_ISL_477014	2020-03-30	Institute of Microbiology, Universidad San Francisco de Quito	Institute of Microbiology, Universidad San Francisco de Quito	Belen Prado-Vivar et al
EPI_ISL_481658	2020-03-30	Department of Virology and Immunology, University of Helsinki and Helsinki University Hospital, Huslab Finland	Department of Virology, Faculty of Medicine, University of Helsinki, Helsinki, Finland	Teemu Smura et al
EPI_ISL_455643	2020-03-30	ICMR-National Institute of Cholera and Enteric Diseases	National Institute of Biomedical Genomics	Arindam Maitra et al
EPI_ISL_644319	2020-03-30	CEPHR / Vincent's Hospital	Irish Coronavirus Sequencing Consortium - National Virus Reference Laboratory	Michael Carr et al
EPI_ISL_430009	2020-03-30	Biolab Diagnostic Laboratories	Andersen lab at Scripps Research	Issa Abu-Dayyeh et al
EPI_ISL_469053	2020-03-30	LNR National Reference Laboratory, Mohammed VI University of Health Sciences	Medical Biotechnology Laboratory, Rabat Medical and Pharmacy School, Mohammed The Vth University in Rabat	Meriem LAAMARTI et al
EPI_ISL_579143	2020-03-30	Southern Community Labs Dunedin	Institute of Environmental Science and Research (ESR)	Xiaoyun Ren et al
EPI_ISL_437754	2020-03-30	Pathogen Genomics Lab King Abdullah University of Science and Technology(KAUST)	Pathogen Genomics Lab King Abdullah University of Science and Technology(KAUST)	Sharif Hala et al
EPI_ISL_437755	2020-03-30	Pathogen Genomics Lab King Abdullah University of Science and Technology(KAUST)	Pathogen Genomics Lab King Abdullah University of Science and Technology(KAUST)	Sharif Hala et al
EPI_ISL_512811	2020-03-30	Kenema Government Hospital, Ministry of Health and Sanitation	Kenema Government Hospital, Ministry of Health and Sanitation	Goba et al
EPI_ISL_445380	2020-03-30	Ramathibodi Hospital	COVID-19 Network Investigations (CONI) Alliance	Elizabeth Batty et al
EPI_ISL_456600	2020-03-30	National Health Laboratory, Timor-Leste	Microbiological Diagnostic Unit Public Health Laboratory, The Peter Doherty Institute for Infection and Immunity	Soares da Silva et al
EPI_ISL_451191	2020-03-30	Uganda Virus Research Institute	MRC/UVRI & LSHTM Uganda Research Unit	Dan Lule Bugembe et al
EPI_ISL_520722	2020-03-30	Mohammed Bin Rashid University of Medicine and Health Sciences	Al Jalila Genomics Center	Ahmad Abou Tayoun et al
EPI_ISL_430801	2020-03-31	Laboratorio de Virología del Hospital de Niños Dr. Ricardo Gutierrez	Área de Secuenciación del Laboratorio de Virología del Hospital de Niños	Nabaes Jodar et al
EPI_ISL_430802	2020-03-31	Departamento de Biología y genética molecular, IACA Laboratorios.	Área de Secuenciación del Laboratorio de Virología del Hospital de Niños	Nabaes Jodar et al
EPI_ISL_458234	2020-03-31	KU Leuven, Rega Institute, Clinical and Epidemiological Virology	KU Leuven, Rega Institute, Clinical and Epidemiological Virology	Tony Wawina-Bokalanga et al
EPI_ISL_476304	2020-03-31	DB Diagnósticos do Brasil	Instituto de Medicina Tropical da Univesidade de São Paulo	Samples: Nelson Gaburo Jr et al
EPI_ISL_476305	2020-03-31	DB Diagnósticos do Brasil	Instituto de Medicina Tropical da Univesidade de São Paulo	Samples: Nelson Gaburo Jr et al

EPI_ISL_476307	2020-03-31	DB Diagnósticos do Brasil	Instituto de Medicina Tropical da Univesidade de São Paulo	Samples: Nelson Gaburo Jr et al
EPI_ISL_476309	2020-03-31	DB Diagnósticos do Brasil	Instituto de Medicina Tropical da Univesidade de São Paulo	Samples: Nelson Gaburo Jr et al
EPI_ISL_476311	2020-03-31	DB Diagnósticos do Brasil	Instituto de Medicina Tropical da Univesidade de São Paulo	Samples: Nelson Gaburo Jr et al
EPI_ISL_476312	2020-03-31	DB Diagnósticos do Brasil	Instituto de Medicina Tropical da Univesidade de São Paulo	Samples: Nelson Gaburo Jr et al
EPI_ISL_476313	2020-03-31	DB Diagnósticos do Brasil	Instituto de Medicina Tropical da Univesidade de São Paulo	Samples: Nelson Gaburo Jr et al
EPI_ISL_476314	2020-03-31	DB Diagnósticos do Brasil	Instituto de Medicina Tropical da Univesidade de São Paulo	Samples: Nelson Gaburo Jr et al
EPI_ISL_470621	2020-03-31	Laboratorio de Virologia Molecular / UFRJ	Bioinformatics Laboratory / LNCC	Alexandra Gerber et al
EPI_ISL_476316	2020-03-31	DB Diagnósticos do Brasil	Instituto de Medicina Tropical da Univesidade de São Paulo	Samples: Nelson Gaburo Jr et al
EPI_ISL_476317	2020-03-31	DB Diagnósticos do Brasil	Instituto de Medicina Tropical da Univesidade de São Paulo	Samples: Nelson Gaburo Jr et al
EPI_ISL_515522	2020-03-31	UPA 24HS de Itatiba	Instituto Adolfo Lutz, Interdiciplinary Procedures Center, Strategic Laboratory	Claudio Tavares Sacchi et al
EPI_ISL_447761	2020-03-31	Instituto Nacional de Salud, Bogotá, Colombia	Grupo de Investigaciones Microbiológicas-UR (GIMUR), Departamento de Biología, Facultad de Ciencias Naturales, Universidad del Rosario, Bogotá, Colombia Instituto Nacional de Salud, Bogotá, Colombia Icahn School of Medicine at Mount Sinai, New York, USA	Juan David Ramírez et al
EPI_ISL_498159	2020-03-31	Instituto Nacional de Salud, Bogotá, Colombia	Instituto Nacional de Salud, Bogotá, Colombia	Katherine Laiton-Donato et al
EPI_ISL_437911	2020-03-31	Laboratory of Microbiology, Medical School, National and Kapodistrian University of Athens	Laboratory of Biology, Department of Medicine, Democritus University of Thrace	Kassela K. et al
EPI_ISL_508368	2020-03-31	Institute of Post Graduate Medical Education & Research	National Institute of Biomedical Genomics	Arindam Maitra et al
EPI_ISL_429786	2020-03-31	Laboratoire National de Sante, Microbiology, Virology	Laboratoire National de Sante, Microbiology, Epidemiology and Microbial Genomics	Anke Wienecke-Baldacchino et al
EPI_ISL_460719	2020-03-31	Dutch COVID-19 response team	Erasmus Medical Center	Bas Oude Munnink et al
EPI_ISL_451973	2020-03-31	1. ViroGenetics - BSL3 Laboratory of Virology, Małopolska Centre of Biotechnology, Jagiellonian University; 2. II Department of Internal Medicine, Faculty of Medicine, Jagiellonian University Medical College; 3. DIAGNOSTYKA Ltd.	1. ViroGenetics - BSL3 Laboratory of Virology, Małopolska Centre of Biotechnology, Jagiellonian University; 2. II Department of Internal Medicine, Faculty of Medicine, Jagiellonian University Medical College.	Marek Sanak et al
EPI_ISL_451975	2020-03-31	1. ViroGenetics - BSL3 Laboratory of Virology, Małopolska Centre of Biotechnology, Jagiellonian University; 2. II Department of Internal Medicine, Faculty of Medicine, Jagiellonian University Medical College; 3. DIAGNOSTYKA Ltd.	1. ViroGenetics - BSL3 Laboratory of Virology, Małopolska Centre of Biotechnology, Jagiellonian University; 2. II Department of Internal Medicine, Faculty of Medicine, Jagiellonian University Medical College.	Marek Sanak et al
EPI_ISL_676576	2020-03-31	Scientific Veterinary Institute Novi Sad	Veterinary Specialized Institute "Kraljevo", Serbia	Vidanovic et al
EPI_ISL_512812	2020-03-31	Kenema Government Hospital, Ministry of Health and Sanitation	Kenema Government Hospital, Ministry of Health and Sanitation	Goba et al
EPI_ISL_577742	2020-03-31	Institute of Virology, Biomedical Research Center of the Slovak Academy of Sciences, Bratislava	Faculty of Natural Sciences, Comenius University, Bratislava	Broňa Brejová et al
EPI_ISL_525479	2020-03-31	Centre for Dengue Research	Centre for Dengue Research	Chandima Jeewandara et al
EPI_ISL_428673	2020-03-31	Centre for Dengue Research	Centre for Dengue Research	Chandima Jeewandara et al
EPI_ISL_430803	2020-04-01	Laboratorio de Virología del Hospital de Niños Dr. Ricardo Gutierrez	Área de Secuenciación del Laboratorio de Virología del Hospital de Niños	Nabaes Jodar et al
EPI_ISL_430804	2020-04-01	Laboratorio de Virología del Hospital de Niños Dr. Ricardo Gutierrez	Área de Secuenciación del Laboratorio de Virología del Hospital de Niños	Nabaes Jodar et al
EPI_ISL_430805	2020-04-01	Departamento de Biología y genética molecular, IACA Laboratorios.	Área de Secuenciación del Laboratorio de Virología del Hospital de Niños	Nabaes Jodar et al
EPI_ISL_430806	2020-04-01	Departamento de Biología y genética molecular, IACA Laboratorios.	Área de Secuenciación del Laboratorio de Virología del Hospital de Niños	Nabaes Jodar et al
EPI_ISL_450732	2020-04-01	Hospital AZ Rivierenland	Institute of Tropical Medicine	Philippe Selhorst et al
EPI_ISL_462472	2020-04-01	Clinical Center, University of Sarajevo	Charite Universitatsmedizin Berlin, Institute of Virology	Victor M Corman et al
EPI_ISL_470568	2020-04-01	Hermes Pardini	Bioinformatics Laboratory / LNCC	Alexandra Gerber et al
EPI_ISL_470569	2020-04-01	Hermes Pardini	Bioinformatics Laboratory / LNCC	Alexandra Gerber et al

EPI_ISL_672672	2020-04-01	DB Diagnosticos do Brasil	Laboratório de Parasitologia Médica - Instituto de Medicina Tropical - Universidade de São Paulo	Brazil-UK Centre for Arbovirus Discovery Diagnosis Genomics et al
EPI_ISL_470600	2020-04-01	Hermes Pardini	Bioinformatics Laboratory / LNCC	Alexandra Gerber et al
EPI_ISL_470601	2020-04-01	Hermes Pardini	Bioinformatics Laboratory / LNCC	Alexandra Gerber et al
EPI_ISL_470602	2020-04-01	Hermes Pardini	Bioinformatics Laboratory / LNCC	Alexandra Gerber et al
EPI_ISL_470603	2020-04-01	Hermes Pardini	Bioinformatics Laboratory / LNCC	Alexandra Gerber et al
EPI_ISL_476318	2020-04-01	DB Diagnósticos do Brasil	Instituto de Medicina Tropical da Univesidade de São Paulo	Samples: Nelson Gaburo Jr et al
EPI_ISL_476326	2020-04-01	DB Diagnósticos do Brasil	Instituto de Medicina Tropical da Univesidade de São Paulo	Samples: Nelson Gaburo Jr et al
EPI_ISL_476319	2020-04-01	DB Diagnósticos do Brasil	Instituto de Medicina Tropical da Univesidade de São Paulo	Samples: Nelson Gaburo Jr et al
EPI_ISL_476324	2020-04-01	DB Diagnósticos do Brasil	Instituto de Medicina Tropical da Univesidade de São Paulo	Samples: Nelson Gaburo Jr et al
EPI_ISL_515524	2020-04-01	PS Municipal Dr Lauro Ribas Braga	Instituto Adolfo Lutz, Interdiciplinary Procedures Center, Strategic Laboratory	Claudio Tavares Sacchi et al
EPI_ISL_476373	2020-04-01	Hospital da Clínicas da Faculdade de Medicina da Universidade de São Paulo	Instituto de Medicina Tropical da Univesidade de São Paulo	Samples: Ingra Morales Claro et al
EPI_ISL_445349	2020-04-01	HOSPITAL SAN JUAN DE DIOS	Instituto de Salud Publica de Chile	Andrés E Castillo et al
EPI_ISL_445352	2020-04-01	HOSPITAL DEL PROFESOR	Instituto de Salud Publica de Chile	Andrés E Castillo et al
EPI_ISL_447767	2020-04-01	Instituto Nacional de Salud, Bogotá, Colombia	Grupo de Investigaciones Microbiológicas-UR (GIMUR), Departamento de Biología, Facultad de Ciencias Naturales, Universidad del Rosario, Bogotá, Colombia Instituto Nacional de Salud, Bogotá, Colombia Icahn School of Medicine at Mount Sinai, New York, USA	Juan David Ramírez et al
EPI_ISL_498163	2020-04-01	Instituto Nacional de Salud, Bogotá, Colombia	Instituto Nacional de Salud, Bogotá, Colombia	Katherine Laiton-Donato et al
EPI_ISL_480326	2020-04-01	Hospital Nacional de Niños	Charité Virology-University of Costa Rica	Andres Moreira-Soto et al
EPI_ISL_463745	2020-04-01	Department of Molecular Virology, Cyprus Institute of Neurology and Genetics	Department of Molecular Virology, Cyprus Institute of Neurology and Genetics	Jan Richter et al
EPI_ISL_584079	2020-04-01	The National Institute of Public Health	State Veterinary Institute Prague	Nagy et al
EPI_ISL_420144	2020-04-01	Department for Virology, Molecular Biology and Genome Research, R. G. Lugar Center for Public Health Research, National Center for Disease Control and Public Health (NCDC) of Georgia.	Department for Virology, Molecular Biology and Genome Research, R. G. Lugar Center for Public Health Research, National Center for Disease Control and Public Health (NCDC) of Georgia.	Gvantsa Chanturia et al
EPI_ISL_501251	2020-04-01	Hellenic Pasteur Institute, National Influenza Reference laboratory of Southern Greece & Unit of Bioinformatics and Applied Genomics	Hellenic Pasteur Institute, National Influenza Reference laboratory of Southern Greece & Unit of Bioinformatics and Applied Genomics	Vasiliki Pogka et al
EPI_ISL_451957	2020-04-01	Molecular Pathology Division, Department of Pathology, Hong Kong Sanatorium & Hospital	Molecular Pathology Division, Department of Pathology, Hong Kong Sanatorium & Hospital	Chun Hang AU et al
EPI_ISL_497818	2020-04-01	Department of Microbiology, The University of Hong Kong	Department of Microbiology, The University of Hong Kong	Kelvin K.W. To et al
EPI_ISL_480202	2020-04-01	Toyama Institute of Health	Pathogen Genomics Center, National Institute of Infectious Diseases	Tsuyoshi Sekizuka et al
EPI_ISL_428956	2020-04-01	Laboratoire National de Sante, Microbiology, Virology	Laboratoire National de Sante, Microbiology, Epidemiology and Microbial Genomics	Anke Wienecke-Baldacchino et al
EPI_ISL_718164	2020-04-01	Ministry of Health Hospitals	Institute of Health and Community Medicine	David Perera et al
EPI_ISL_469054	2020-04-01	LNR National Reference Laboratory, Mohammed VI University of Health Sciences	Medical Biotechnology Laboratory, Rabat Medical and Pharmacy School, Mohammed The Vth University in Rabat	Meriem LAAMARTI et al
EPI_ISL_728236	2020-04-01	LNR National Reference Laboratory, Mohammed VI University of Health Sciences	Medical Biotechnology Laboratory, Rabat Medical and Pharmacy School, Mohammed The Vth University in Rabat	Souad KARTTI et al
EPI_ISL_632908	2020-04-01	Genomic Sciences, Rehman Medical Institute	Genomic Sciences, Rehman Medical Institute	Ali et al
EPI_ISL_524473	2020-04-01	Laboratorio de Referencia Nacional de Virus Respiratorio. Centro Nacional de Salud Publica. Instituto Nacional de Salud Peru.	Laboratorio de Referencia Nacional de Biotecnología y Biología Molecular. Centro Nacional de Salud Publica. Instituto Nacional de Salud Peru.	Carlos Padilla Rojas et al

EPI_ISL_451971	2020-04-01	1. ViroGenetics - BSL3 Laboratory of Virology, Małopolska Centre of Biotechnology, Jagiellonian University; 2. II Department of Internal Medicine, Faculty of Medicine, Jagiellonian University Medical College; 3. DIAGNOSTYKA Ltd.	1. ViroGenetics - BSL3 Laboratory of Virology, Małopolska Centre of Biotechnology, Jagiellonian University; 2. II Department of Internal Medicine, Faculty of Medicine, Jagiellonian University Medical College.	Marek Sanak et al
EPI_ISL_455441	2020-04-01	1. ViroGenetics - BSL3 Laboratory of Virology, Małopolska Centre of Biotechnology, Jagiellonian University; 2. II Department of Internal Medicine, Faculty of Medicine, Jagiellonian University Medical College; 3. Narodowy Instytut Zdrowia Publicznego – Państwowy Zakład Higieny (NIZP-PZH)	1. ViroGenetics - BSL3 Laboratory of Virology, Małopolska Centre of Biotechnology, Jagiellonian University; 2. II Department of Internal Medicine, Faculty of Medicine, Jagiellonian University Medical College; 3. Narodowy Instytut Zdrowia Publicznego – Państwowy Zakład Higieny (NIZP-PZH).	Katarzyna Pancer et al
EPI_ISL_455448	2020-04-01	1. ViroGenetics - BSL3 Laboratory of Virology, Małopolska Centre of Biotechnology, Jagiellonian University; 2. II Department of Internal Medicine, Faculty of Medicine, Jagiellonian University Medical College; 3. Narodowy Instytut Zdrowia Publicznego – Państwowy Zakład Higieny (NIZP-PZH)	1. ViroGenetics - BSL3 Laboratory of Virology, Małopolska Centre of Biotechnology, Jagiellonian University; 2. II Department of Internal Medicine, Faculty of Medicine, Jagiellonian University Medical College; 3. Narodowy Instytut Zdrowia Publicznego – Państwowy Zakład Higieny (NIZP-PZH).	Katarzyna Pancer et al
EPI_ISL_462434	2020-04-01	unknown	Laboratory Diagnostic	Vidanovic et al
EPI_ISL_661200	2020-04-01	Scientific Veterinary Institute Novi Sad	Veterinary Specialized Institute "Kraljevo", Serbia	Vidanovic et al
EPI_ISL_635283	2020-04-01	Institute of Microbiology and Immunology, Faculty of Medicine, University of Ljubljana	Institute of Microbiology and Immunology, Faculty of Medicine, University of Ljubljana	Tomaž Mark Zorec et al
EPI_ISL_506990	2020-04-01	Division of Viral Diseases, Center for Laboratory Control of Infectious Diseases, Korea Centers for Diseases Control and Prevention	Division of Viral Diseases, Center for Laboratory Control of Infectious Diseases, Korea Centers for Diseases Control and Prevention	Jeong-Min Kim et al
EPI_ISL_485395	2020-04-01	University of Ulsan College of Medicine and Asan Medical Center	University of Ulsan College of Medicine and Asan Medical Center	Kuenyoul Park et al
EPI_ISL_447519	2020-04-01	Servicio de Microbiologia. Hospital Clinico Universitario de Valencia	Sequencing and Bioinformatics Service and Molecular Epidemiology Research Group. FISABIO-Public Health	Sandra Carbo et al
EPI_ISL_447593	2020-04-01	TSGH-CP molecular lab	TSGH-CP molecular lab	Cherng-Lih Perng et al
EPI_ISL_447018	2020-04-01	Ramathibodi Hospital	COVID-19 Network Investigations (CONI) Alliance	Elizabeth Batty et al
EPI_ISL_447019	2020-04-01	Ramathibodi Hospital	COVID-19 Network Investigations (CONI) Alliance	Elizabeth Batty et al
EPI_ISL_654881	2020-04-01	Pasteur Institute in Ho Chi Minh city	Department of Microbiology and Immunology - Pasteur Institute in Ho Chi Minh city	Lương Chấn Quang et al
EPI_ISL_539308	2020-04-01	KWR Watercycle Research Institute	Erasmus Medical Center	Ray Izquierdo-Lara et al
EPI_ISL_430807	2020-04-02	Laboratorio de Virología del Hospital de Niños Dr. Ricardo Gutierrez	Área de Secuenciación del Laboratorio de Virología del Hospital de Niños	Nabaes Jodar et al
EPI_ISL_583762	2020-04-02	Dr. Gernot Walder GmbH	Bergthaler laboratory, CeMM Research Center for Molecular Medicine of the Austrian Academy of Sciences	Alexandra Popa et al
EPI_ISL_470570	2020-04-02	Hermes Pardini	Bioinformatics Laboratory / LNCC	Alexandra Gerber et al
EPI_ISL_470571	2020-04-02	Hermes Pardini	Bioinformatics Laboratory / LNCC	Alexandra Gerber et al
EPI_ISL_470572	2020-04-02	Hermes Pardini	Bioinformatics Laboratory / LNCC	Alexandra Gerber et al
EPI_ISL_470573	2020-04-02	Hermes Pardini	Bioinformatics Laboratory / LNCC	Alexandra Gerber et al
EPI_ISL_476331	2020-04-02	DB Diagnósticos do Brasil	Instituto de Medicina Tropical da Univesidade de São Paulo	Samples: Nelson Gaburo Jr et al
EPI_ISL_476333	2020-04-02	DB Diagnósticos do Brasil	Instituto de Medicina Tropical da Univesidade de São Paulo	Samples: Nelson Gaburo Jr et al
EPI_ISL_476336	2020-04-02	DB Diagnósticos do Brasil	Instituto de Medicina Tropical da Univesidade de São Paulo	Samples: Nelson Gaburo Jr et al
EPI_ISL_476327	2020-04-02	DB Diagnósticos do Brasil	Instituto de Medicina Tropical da Univesidade de São Paulo	Samples: Nelson Gaburo Jr et al
EPI_ISL_476328	2020-04-02	DB Diagnósticos do Brasil	Instituto de Medicina Tropical da Univesidade de São Paulo	Samples: Nelson Gaburo Jr et al
EPI_ISL_476329	2020-04-02	DB Diagnósticos do Brasil	Instituto de Medicina Tropical da Univesidade de São Paulo	Samples: Nelson Gaburo Jr et al
EPI_ISL_476330	2020-04-02	DB Diagnósticos do Brasil	Instituto de Medicina Tropical da Univesidade de São Paulo	Samples: Nelson Gaburo Jr et al
EPI_ISL_470604	2020-04-02	Hermes Pardini	Bioinformatics Laboratory / LNCC	Alexandra Gerber et al
EPI_ISL_470605	2020-04-02	Hermes Pardini	Bioinformatics Laboratory / LNCC	Alexandra Gerber et al
EPI_ISL_470606	2020-04-02	Hermes Pardini	Bioinformatics Laboratory / LNCC	Alexandra Gerber et al
EPI_ISL_470614	2020-04-02	Hermes Pardini	Bioinformatics Laboratory / LNCC	Alexandra Gerber et al

EPI_ISL_476335	2020-04-02	DB Diagnósticos do Brasil	Instituto de Medicina Tropical da Univesidade de São Paulo	Samples: Nelson Gaburo Jr et al
EPI_ISL_515523	2020-04-02	PS Municipal Dr Lauro Ribas Braga	Instituto Adolfo Lutz, Interdiciplinary Procedures Center, Strategic Laboratory	Claudio Tavares Sacchi et al
EPI_ISL_538348	2020-04-02	Kingston Health Sciences Centre / Queen's University	Ontario Institute for Cancer Research	Prameet M. Sheth et al
EPI_ISL_538349	2020-04-02	Kingston Health Sciences Centre / Queen's University	Ontario Institute for Cancer Research	Prameet M. Sheth et al
EPI_ISL_426889	2020-04-02	Motol University Hospital	Institute of Applied Biotechnologies a.s.	Petr Brož et al
EPI_ISL_508968	2020-04-02	Centre Hospitalier de Bourg en Bresse	CNR Virus des Infections Respiratoires - France SUD	Antonin Bal et al
EPI_ISL_487370	2020-04-02	Hellenic Pasteur Institute, National Influenza Reference laboratory of Southern Greece & Unit of Bioinformatics and Applied Genomics	Hellenic Pasteur Institute, National Influenza Reference laboratory of Southern Greece & Unit of Bioinformatics and Applied Genomics	Vasiliki Pogka et al
EPI_ISL_480203	2020-04-02	Toyama Institute of Health	Pathogen Genomics Center, National Institute of Infectious Diseases	Tsuyoshi Sekizuka et al
EPI_ISL_430013	2020-04-02	Biolab Diagnostic Laboratories	Andersen lab at Scripps Research	Issa Abu-Dayyeh et al
EPI_ISL_517687	2020-04-02	Laboratorio de Referencia Nacional de Virus Respiratorio. Centro Nacional de Salud Publica. Instituto Nacional de Salud Peru.	Laboratorio de Referencia Nacional de Biotecnología y Biología Molecular. Centro Nacional de Salud Publica. Instituto Nacional de Salud Peru.	Carlos Padilla Rojas et al
EPI_ISL_514317	2020-04-02	Laboratorio de Referencia Nacional de Virus Respiratorio. Instituto Nacional de Salud Perú	Laboratorio de Referencia Nacional de Biotecnología y Biología Molecular. Instituto Nacional de Salud Perú	Carlos Padilla Rojas et al
EPI_ISL_451980	2020-04-02	1. ViroGenetics - BSL3 Laboratory of Virology, Małopolska Centre of Biotechnology, Jagiellonian University; 2. II Department of Internal Medicine, Faculty of Medicine, Jagiellonian University Medical College; 3. DIAGNOSTYKA Ltd.	1. ViroGenetics - BSL3 Laboratory of Virology, Małopolska Centre of Biotechnology, Jagiellonian University; 2. II Department of Internal Medicine, Faculty of Medicine, Jagiellonian University Medical College.	Marek Sanak et al
EPI_ISL_613453	2020-04-02	Institut Pasteur de la Guadeloupe	Institut Pasteur de la Guadeloupe	Marion Barbet et al
EPI_ISL_613454	2020-04-02	Institut Pasteur de la Guadeloupe	Institut Pasteur de la Guadeloupe	Marion Barbet et al
EPI_ISL_467434	2020-04-02	AMPATH-DBN	KRISP, KZN Research Innovation and Sequencing Platform	Giandhari J et al
EPI_ISL_506988	2020-04-02	Division of Viral Diseases, Center for Laboratory Control of Infectious Diseases, Korea Centers for Diseases Control and Prevention	Division of Viral Diseases, Center for Laboratory Control of Infectious Diseases, Korea Centers for Diseases Control and Prevention	Jeong-Min Kim et al
EPI_ISL_506989	2020-04-02	Division of Viral Diseases, Center for Laboratory Control of Infectious Diseases, Korea Centers for Diseases Control and Prevention	Division of Viral Diseases, Center for Laboratory Control of Infectious Diseases, Korea Centers for Diseases Control and Prevention	Jeong-Min Kim et al
EPI_ISL_581675	2020-04-02	University Hospital Basel, Clinical Virology	University Hospital Basel, Clinical Bacteriology	Madlen Stange et al
EPI_ISL_436108	2020-04-02	TSGH-CP molecular lab	TSGH-CP molecular lab	Cherng-Lih Perng et al
EPI_ISL_436099	2020-04-02	TSGH-CP molecular lab	TSGH-CP molecular lab	Cherng-Lih Perng et al
EPI_ISL_447022	2020-04-02	Ramathibodi Hospital	COVID-19 Network Investigations (CONI) Alliance	Elizabeth Batty et al
EPI_ISL_447025	2020-04-02	Ramathibodi Hospital	COVID-19 Network Investigations (CONI) Alliance	Elizabeth Batty et al
EPI_ISL_476702	2020-04-02	Incubadora Venezolana de Ciencia, Venezuela	Incubadora Venezolana de Ciencia, Venezuela / Instituto Nacional de Salud, Bogotá, Colombia / Grupo de Investigaciones Microbiológicas-UR (GIMUR), Departamento de Biología, Facultad de Ciencias Naturales, Universidad del Rosario, Bogotá, Colombia / Icahn School of Medicine at Mount Sinai, New York, USA	Alberto Paniz-Mondolfi et al
EPI_ISL_455707	2020-04-02	National Hospital of Tropical Diseases	Oxford University Clinical Research Unit, Hanoi, Vietnam	Nguyen Thi Tam et al
EPI_ISL_475828	2020-04-03	Institut für Virologie am Department für Hygiene, Mikrobiologie und Public Health	Bergthaler laboratory, CeMM Research Center for Molecular Medicine of the Austrian Academy of Sciences	Alexandra Popa et al
EPI_ISL_475896	2020-04-03	Zentralinstitut für medizinische und chemische Labordiagnostik, Universitätskliniken Insbruck	Bergthaler laboratory, CeMM Research Center for Molecular Medicine of the Austrian Academy of Sciences	Alexandra Popa et al
EPI_ISL_462235	2020-04-03	KU Leuven, Rega Institute, Clinical and Epidemiological Virology	KU Leuven, Rega Institute, Clinical and Epidemiological Virology	Tony Wawina-Bokalanga et al
EPI_ISL_424631	2020-04-03	Department of Clinical Microbiology	GIGA Medical Genomics	Keith Durkin et al
EPI_ISL_524792	2020-04-03	Evandro Chagas Institute	Evandro Chagas Institute	Santos et al

EPI_ISL_458138	2020-04-03	Evandro Chagas Institute	Evandro Chagas Institute	Santos et al
EPI_ISL_470576	2020-04-03	Hermes Pardini	Bioinformatics Laboratory / LNCC	Alexandra Gerber et al
EPI_ISL_470585	2020-04-03	Hermes Pardini	Bioinformatics Laboratory / LNCC	Alexandra Gerber et al
EPI_ISL_470595	2020-04-03	Simile	Bioinformatics Laboratory / LNCC	Alexandra Gerber et al
EPI_ISL_470608	2020-04-03	Hermes Pardini	Bioinformatics Laboratory / LNCC	Alexandra Gerber et al
EPI_ISL_470609	2020-04-03	Hermes Pardini	Bioinformatics Laboratory / LNCC	Alexandra Gerber et al
EPI_ISL_470610	2020-04-03	Hermes Pardini	Bioinformatics Laboratory / LNCC	Alexandra Gerber et al
EPI_ISL_470611	2020-04-03	Hermes Pardini	Bioinformatics Laboratory / LNCC	Alexandra Gerber et al
EPI_ISL_470612	2020-04-03	Hermes Pardini	Bioinformatics Laboratory / LNCC	Alexandra Gerber et al
EPI_ISL_476359	2020-04-03	DB Diagnósticos do Brasil	Instituto de Medicina Tropical da Univesidade de São Paulo	Samples: Nelson Gaburo Jr et al
EPI_ISL_729816	2020-04-03	Laboratorio Central de Saude Publica do Estado do Rio Grande do Sul (LACEN-RS)	Laboratory of Respiratory Viruses and Measles, Oswaldo Cruz Institute, FIOCRUZ	Paola Resende et al
EPI_ISL_729817	2020-04-03	Laboratorio Central de Saude Publica do Estado do Rio Grande do Sul (LACEN-RS)	Laboratory of Respiratory Viruses and Measles, Oswaldo Cruz Institute, FIOCRUZ	Paola Resende et al
EPI_ISL_470655	2020-04-03	Hermes Pardini	Bioinformatics Laboratory / LNCC	Alexandra Gerber et al
EPI_ISL_445373	2020-04-03	HOSPITAL SAN JUAN DE DIOS	Instituto de Salud Publica de Chile	Andrés E Castillo et al
EPI_ISL_498167	2020-04-03	Instituto Nacional de Salud, Bogotá, Colombia	Instituto Nacional de Salud, Bogotá, Colombia	Katherine Laiton-Donato et al
EPI_ISL_444849	2020-04-03	Department of Virus and Microbiological Special Diagnostics, Statens Serum Institut, Copenhagen, Denmark, Artillerivej 5, 2300 Copenahgen S	Albertsen lab, Department of Chemistry and Bioscience, Aalborg University, Denmark	Rasmus Kirkegaard et al
EPI_ISL_444854	2020-04-03	Department of Virus and Microbiological Special Diagnostics, Statens Serum Institut, Copenhagen, Denmark, Artillerivej 5, 2300 Copenahgen S	Albertsen lab, Department of Chemistry and Bioscience, Aalborg University, Denmark	Rasmus Kirkegaard et al
EPI_ISL_539851	2020-04-03	Pok Oi Hospital	Hong Kong Department of Health	Alan K.L. Tsang et al
EPI_ISL_458080	2020-04-03	CSIR-Centre for Cellular and Molecular Biology	CSIR-Centre for Cellular and Molecular Biology	Sakshi Shambhavi et al
EPI_ISL_582033	2020-04-03	Department of Pathology, School of Medicine, Imam Khomeini Hospital, Tehran University of Medical Sciences	Genetics Research Center. University Of Social Welfare And Rehabilitation Sciences	Zohreh Fattahi et al
EPI_ISL_480197	2020-04-03	Toyama Institute of Health	Pathogen Genomics Center, National Institute of Infectious Diseases	Tsuyoshi Sekizuka et al
EPI_ISL_512814	2020-04-03	Kenema Government Hospital, Ministry of Health and Sanitation	Kenema Government Hospital, Ministry of Health and Sanitation	Goba et al
EPI_ISL_512815	2020-04-03	Kenema Government Hospital, Ministry of Health and Sanitation	Kenema Government Hospital, Ministry of Health and Sanitation	Goba et al
EPI_ISL_572329	2020-04-03	Public Health Authority of the Slovak Republic, Bratislava	Faculty of Natural Sciences, Comenius University in Bratislava	Dominika Fričová et al
EPI_ISL_635286	2020-04-03	Institute of Microbiology and Immunology, Faculty of Medicine, University of Ljubljana	Institute of Microbiology and Immunology, Faculty of Medicine, University of Ljubljana	Tomaž Mark Zorec et al
EPI_ISL_467433	2020-04-03	AMPATH-DBN	KRISP, KZN Research Innovation and Sequencing Platform	Giandhari J et al
EPI_ISL_467451	2020-04-03	AMPATH-DBN	KRISP, KZN Research Innovation and Sequencing Platform	Giandhari J et al
EPI_ISL_429207	2020-04-03	University Hospitals of Geneva Laboratory of Virology	University Hospitals of Geneva Laboratory of Virology	Laubscher F. et al
EPI_ISL_508822	2020-04-03	Florida Bureau of Public Health Laboratories	Florida Bureau of Public Health Laboratories	Sarah Schmedes et al
EPI_ISL_457965	2020-04-03	Laboratorio de Biología Molecular Asociación Española Primera en Salud	Departments of Pathology and Medicine, New York University School of Medicine	Maria Victoria Elizondo et al
EPI_ISL_424649	2020-04-04	Department of Clinical Microbiology	GIGA Medical Genomics	Keith Durkin et al
EPI_ISL_476825	2020-04-04	Laboratoire des Fièvres Hémorragiques Virales du Benin	Charité-Universitätsmedizin Berlin	Yadouleton et al
EPI_ISL_470577	2020-04-04	Hermes Pardini	Bioinformatics Laboratory / LNCC	Alexandra Gerber et al
EPI_ISL_470586	2020-04-04	Hermes Pardini	Bioinformatics Laboratory / LNCC	Alexandra Gerber et al

EPI_ISL_447802	2020-04-04	Instituto Nacional de Salud, Bogotá, Colombia	Grupo de Investigaciones Microbiológicas-UR (GIMUR), Departamento de Biología, Facultad de Ciencias Naturales, Universidad del Rosario, Bogotá, Colombia Instituto Nacional de Salud, Bogotá, Colombia Icahn School of Medicine at Mount Sinai, New York, USA	Juan David Ramírez et al
EPI_ISL_447798	2020-04-04	Instituto Nacional de Salud, Bogotá, Colombia	Grupo de Investigaciones Microbiológicas-UR (GIMUR), Departamento de Biología, Facultad de Ciencias Naturales, Universidad del Rosario, Bogotá, Colombia Instituto Nacional de Salud, Bogotá, Colombia Icahn School of Medicine at Mount Sinai, New York, USA	Juan David Ramírez et al
EPI_ISL_447796	2020-04-04	Instituto Nacional de Salud, Bogotá, Colombia	Grupo de Investigaciones Microbiológicas-UR (GIMUR), Departamento de Biología, Facultad de Ciencias Naturales, Universidad del Rosario, Bogotá, Colombia Instituto Nacional de Salud, Bogotá, Colombia Icahn School of Medicine at Mount Sinai, New York, USA	Juan David Ramírez et al
EPI_ISL_480327	2020-04-04	Hospital Nacional de Niños	Charité Virology-University of Costa Rica	Andres Moreira-Soto et al
EPI_ISL_629097	2020-04-04	Laboratoire du Centre Hospitalier Annecy Genevois	CNR Virus des Infections Respiratoires - France SUD	Antonin Bal et al
EPI_ISL_479572	2020-04-04	NIV Influenza	NIV Influenza	Potdar V et al
EPI_ISL_480093	2020-04-04	Department of Infectious Diseases, Kobe Institute of Health	Pathogen Genomics Center, National Institute of Infectious Diseases	Tsuyoshi Sekizuka et al
EPI_ISL_430008	2020-04-04	Biolab Diagnostic Laboratories	Andersen lab at Scripps Research	Issa Abu-Dayyeh et al
EPI_ISL_428942	2020-04-04	Laboratoire National de Sante, Microbiology, Virology	Laboratoire National de Sante, Microbiology, Epidemiology and Microbial Genomics	Anke Wienecke-Baldacchino et al
EPI_ISL_579220	2020-04-04	Canterbury Health Laboratories	Institute of Environmental Science and Research (ESR)	Xiaoyun Ren et al
EPI_ISL_456354	2020-04-04	Canterbury Health Laboratories	Institute of Environmental Science and Research (ESR)	Matt Storey et al
EPI_ISL_613455	2020-04-04	Institut Pasteur de la Guadeloupe	Institut Pasteur de la Guadeloupe	Marion Barbet et al
EPI_ISL_469125	2020-04-04	National Public Health Laboratory, National Centre for Infectious Diseases	National Public Health Laboratory, National Centre for Infectious Diseases	Mak TM et al
EPI_ISL_467468	2020-04-04	AMPATH-DBN	KRISP, KZN Research Innovation and Sequencing Platform	Giandhari J et al
EPI_ISL_510507	2020-04-04	Servicio de Microbiología. Hospital Universitario Donostia. OSI Donostialdea. Área de Enfermedades Infecciosas, Grupo de Infección Respiratoria y Resistencia Antimicrobiana. Instituto de Investigación Sanitaria Biodonostia	SeqCOVID-SPAIN consortium/IBV(CSIC)	Gustavo Cilla et al
EPI_ISL_510509	2020-04-04	Servicio de Microbiología. Hospital Universitario Donostia. OSI Donostialdea. Área de Enfermedades Infecciosas, Grupo de Infección Respiratoria y Resistencia Antimicrobiana. Instituto de Investigación Sanitaria Biodonostia	SeqCOVID-SPAIN consortium/IBV(CSIC)	Gustavo Cilla et al
EPI_ISL_471248	2020-04-04	Wisconsin State Laboratory of Hygiene Communicable Disease Division	Wisconsin State Laboratory of Hygiene Communicable Disease Division	Kelsey R. Florek et al
EPI_ISL_427131	2020-04-05	Victorian Infectious Diseases Reference Laboratory (VIDRL)	Microbiological Diagnostic Unit Public Health Laboratory and Victorian Infectious Diseases Reference Laboratory, Doherty Institute	Caly L. et al
EPI_ISL_483562	2020-04-05	Kingdom of Bahrein Ministry of Health	Erasmus Medical Center	Bas Oude Munnink et al
EPI_ISL_560387	2020-04-05	National Health Laboratory	Botswana Institute for Technology Research and Innovation	Kefentse Arnold Tumedi et al
EPI_ISL_458142	2020-04-05	Evandro Chagas Institute	Evandro Chagas Institute	Santos et al
EPI_ISL_470587	2020-04-05	Hermes Pardini	Bioinformatics Laboratory / LNCC	Alexandra Gerber et al
EPI_ISL_445370	2020-04-05	HOSPITAL DE CARABINEROS	Instituto de Salud Publica de Chile	Andrés E Castillo et al
EPI_ISL_447811	2020-04-05	Instituto Nacional de Salud, Bogotá, Colombia	Grupo de Investigaciones Microbiológicas-UR (GIMUR), Departamento de Biología, Facultad de Ciencias Naturales, Universidad del Rosario, Bogotá, Colombia Instituto Nacional de Salud, Bogotá, Colombia Icahn School of Medicine at Mount Sinai, New York, USA	Juan David Ramírez et al

EPI_ISL_433731	2020-04-05	Department of Pathology, University of Cambridge	COVID-19 Genomics UK (COG-UK) Consortium	Luke W Meredith et al
EPI_ISL_457890	2020-04-05	KEMRI-CGMR-C	KEMRI-Wellcome Trust Research Programme/KEMRI-CGMR-C Kilifi	Githinji G. et al 2020 et al
EPI_ISL_603085	2020-04-05	Lithuanian University of Health Sciences Hospital, Department of Laboratory Medicine	Lithuanian University of Health Sciences, Molecular cardiology lab.	Lukas Zemaitis et al
EPI_ISL_579181	2020-04-05	Southern Community Labs Dunedin	Institute of Environmental Science and Research (ESR)	Xiaoyun Ren et al
EPI_ISL_475811	2020-04-06	Center for Virology, Medical University of Vienna	Bergthaler laboratory, CeMM Research Center for Molecular Medicine of the Austrian Academy of Sciences	Alexandra Popa et al
EPI_ISL_483640	2020-04-06	Kingdom of Bahrein Ministry of Health	Erasmus Medical Center	Bas Oude Munnink et al
EPI_ISL_476828	2020-04-06	Laboratoire des Fièvres Hémorragiques Virales du Benin	Charité-Universitätsmedizin Berlin	Yadouleton et al
EPI_ISL_476369	2020-04-06	DB Diagnósticos do Brasil	Instituto de Medicina Tropical da Univesidade de São Paulo	Samples: Nelson Gaburo Jr et al
EPI_ISL_458149	2020-04-06	Evandro Chagas Institute	Evandro Chagas Institute	Santos et al
EPI_ISL_672666	2020-04-06	DB Diagnosticos do Brasil	Laboratório de Parasitologia Médica - Instituto de Medicina Tropical - Universidade de São Paulo	Brazil-UK Centre for Arbovirus Discovery Diagnosis Genomics et al
EPI_ISL_672667	2020-04-06	DB Diagnosticos do Brasil	Laboratório de Parasitologia Médica - Instituto de Medicina Tropical - Universidade de São Paulo	Brazil-UK Centre for Arbovirus Discovery Diagnosis Genomics et al
EPI_ISL_470588	2020-04-06	Hermes Pardini	Bioinformatics Laboratory / LNCC	Alexandra Gerber et al
EPI_ISL_476370	2020-04-06	DB Diagnósticos do Brasil	Instituto de Medicina Tropical da Univesidade de São Paulo	Samples: Nelson Gaburo Jr et al
EPI_ISL_729818	2020-04-06	Laboratorio Central de Saude Publica do Estado do Rio Grande do Sul (LACEN-RS)	Laboratory of Respiratory Viruses and Measles, Oswaldo Cruz Institute, FIOCRUZ	Paola Resende et al
EPI_ISL_722084	2020-04-06	Hospital das Clínicas Universidade de São Paulo Medical School	Laboratório de Parasitologia Médica - Instituto de Medicina Tropical - Universidade de São Paulo	Brazil-UK Centre for Arbovirus Discovery Diagnosis Genomics et al
EPI_ISL_445369	2020-04-06	HOSPITAL DE CARABINEROS	Instituto de Salud Publica de Chile	Andrés E Castillo et al
EPI_ISL_456156	2020-04-06	Instituto Nacional de Salud - Unidad de Secuenciación y Análisis Genómico	Instituto Nacional de Salud, Universidad Cooperativa de Colombia, Instituto Alexander von Humboldt, Imperial College-London, London School of Hygiene & Tropical Medicine	Katherine Laiton-Donato et al
EPI_ISL_480328	2020-04-06	Laboratorio LABIN	Charité Virology-University of Costa Rica	Andres Moreira-Soto et al
EPI_ISL_437291	2020-04-06	Max von Pettenkofer Institute, Virology, National Reference Center for Retroviruses, LMU München	Laboratory for Functional Genome Analysis, Dept. Genomics, Gene Center of the LMU Munich	Max Muenchhoff et al
EPI_ISL_466874	2020-04-06	Max von Pettenkofer Institute, Virology, National Reference Center for Retroviruses, LMU München	Laboratory for Functional Genome Analysis, Dept. Genomics, Gene Center of the LMU Munich	Max Muenchhoff et al
EPI_ISL_681926	2020-04-06	National Virus Reference Laboratory	Irish Coronavirus Sequencing Consortium - Teagasc Moorepark	Paul Cotter et al
EPI_ISL_483725	2020-04-06	Israel Central Virology laboratory	Israel Central Virology laboratory	Neta Zuckerman et al
EPI_ISL_450188	2020-04-06	Biolab Diagnostic Laboratories	Andersen lab at Scripps Research	Issa Abu-Dayyeh et al
EPI_ISL_450189	2020-04-06	Biolab Diagnostic Laboratories	Andersen lab at Scripps Research	Issa Abu-Dayyeh et al
EPI_ISL_603087	2020-04-06	Lithuanian University of Health Sciences Hospital, Department of Laboratory Medicine	Lithuanian University of Health Sciences, Molecular cardiology lab.	Lukas Zemaitis et al
EPI_ISL_541854	2020-04-06	Lithuanian University of Health Sciences Hospital, Department of Laboratory Medicine	Lithuanian University of Health Sciences, Laboratory of Molecular Cardiology	Lukas Zemaitis et al
EPI_ISL_487448	2020-04-06	CICM-Mali	Bundeswehr Institut of Microbiology	Kouriba et al
EPI_ISL_459984	2020-04-06	Institut Pasteur du Maroc	Institut Pasteur du Maroc	Marion Barbet et al
EPI_ISL_664103	2020-04-06	Dept. of Microbiology and Infection Control, Akershus University Hospital HF	Dept. of Microbiology and Infection Control, Akershus University Hospital HF	Hege Vangstein Aamot et al
EPI_ISL_666604	2020-04-06	Dept. of Microbiology and Infection Control, Akershus University Hospital HF	Dept. of Microbiology and Infection Control, Akershus University Hospital HF	Hege Vangstein Aamot et al
EPI_ISL_666606	2020-04-06	Dept. of Microbiology and Infection Control, Akershus University Hospital HF	Dept. of Microbiology and Infection Control, Akershus University Hospital HF	Hege Vangstein Aamot et al
EPI_ISL_513056	2020-04-06	Pathogen Genomics Lab King Abdullah University of Science and Technology(KAUST)	Pathogen Genomics Lab King Abdullah University of Science and Technology(KAUST)	Rahul P Salunke et al

EPI_ISL_577735	2020-04-06	Institute of Virology, Biomedical Research Center of the Slovak Academy of Sciences, Bratislava	Faculty of Natural Sciences, Comenius University, Bratislava	Kristína Boršová et al
EPI_ISL_577736	2020-04-06	Institute of Virology, Biomedical Research Center of the Slovak Academy of Sciences, Bratislava	Faculty of Natural Sciences, Comenius University, Bratislava	Viktória Hodorová et al
EPI_ISL_635292	2020-04-06	Institute of Microbiology and Immunology, Faculty of Medicine, University of Ljubljana	Institute of Microbiology and Immunology, Faculty of Medicine, University of Ljubljana	Tomaž Mark Zorec et al
EPI_ISL_447027	2020-04-06	Ramathibodi Hospital	COVID-19 Network Investigations (CONI) Alliance	Elizabeth Batty et al
EPI_ISL_483638	2020-04-07	Kingdom of Bahrein Ministry of Health	Erasmus Medical Center	Bas Oude Munnink et al
EPI_ISL_560385	2020-04-07	National Health Laboratory	Botswana Institute for Technology Research and Innovation	Kefentse Arnold Tumedi et al
EPI_ISL_458140	2020-04-07	Evandro Chagas Institute	Evandro Chagas Institute	Santos et al
EPI_ISL_500460	2020-04-07	LACEN/PE	WallauLab, Aggeu Magalhaes Institute	Marcelo Henrique Santos Paiva et al
EPI_ISL_500486	2020-04-07	LACEN/PE	WallauLab, Aggeu Magalhaes Institute	Marcelo Henrique Santos Paiva et al
EPI_ISL_672681	2020-04-07	DB Diagnosticos do Brasil	Laboratório de Parasitologia Médica - Instituto de Medicina Tropical - Universidade de São Paulo	Brazil-UK Centre for Arbovirus Discovery Diagnosis Genomics et al
EPI_ISL_672684	2020-04-07	DB Diagnosticos do Brasil	Laboratório de Parasitologia Médica - Instituto de Medicina Tropical - Universidade de São Paulo	Brazil-UK Centre for Arbovirus Discovery Diagnosis Genomics et al
EPI_ISL_491934	2020-04-07	Centro de Investigaciones, Universidad de Especialidades Espíritu Santo	Institute of Microbiology, Universidad San Francisco de Quito	Derly Andrade et al
EPI_ISL_527819	2020-04-07	Centro de Investigaciones, Universidad de Especialidades Espíritu Santo	Institute of Microbiology, Universidad San Francisco de Quito	Derly Andrade et al
EPI_ISL_448804	2020-04-07	Oxford Viromics, NDM, University of Oxford; Oxford University Hospitals; Basingstoke and North Hampshire Hospital	COVID-19 Genomics UK (COG-UK) Consortium	Tanya Golubchik et al
EPI_ISL_437292	2020-04-07	Max von Pettenkofer Institute, Virology, National Reference Center for Retroviruses, LMU München	Laboratory for Functional Genome Analysis, Dept. Genomics, Gene Center of the LMU Munich	Max Muenchhoff et al
EPI_ISL_497823	2020-04-07	Department of Microbiology, The University of Hong Kong	Department of Microbiology, The University of Hong Kong	Kelvin K.W. To et al
EPI_ISL_681918	2020-04-07	National Virus Reference Laboratory	Irish Coronavirus Sequencing Consortium - Teagasc Moorepark	Paul Cotter et al
EPI_ISL_480205	2020-04-07	Department of Infectious Diseases, Kobe Institute of Health	Pathogen Genomics Center, National Institute of Infectious Diseases	Tsuyoshi Sekizuka et al
EPI_ISL_450186	2020-04-07	Biolab Diagnostic Laboratories	Andersen lab at Scripps Research	Issa Abu-Dayyeh et al
EPI_ISL_428958	2020-04-07	Laboratoire National de Sante, Microbiology, Virology	Laboratoire National de Sante, Microbiology, Epidemiology and Microbial Genomics	Anke Wienecke-Baldacchino et al
EPI_ISL_455158	2020-04-07	Dutch COVID-19 response team	Erasmus Medical Center	Bas Oude Munnink et al
EPI_ISL_451981	2020-04-07	1. ViroGenetics - BSL3 Laboratory of Virology, Małopolska Centre of Biotechnology, Jagiellonian University; 2. II Department of Internal Medicine, Faculty of Medicine, Jagiellonian University Medical College; 3. DIAGNOSTYKA Ltd.	1. ViroGenetics - BSL3 Laboratory of Virology, Małopolska Centre of Biotechnology, Jagiellonian University; 2. II Department of Internal Medicine, Faculty of Medicine, Jagiellonian University Medical College.	Marek Sanak et al
EPI_ISL_613456	2020-04-07	Institut Pasteur de la Guadeloupe	Institut Pasteur de la Guadeloupe	Marion Barbet et al
EPI_ISL_648093	2020-04-07	Department of Laboratory Medicine, Tan Tock Seng Hospital	Department of Laboratory Medicine, Tan Tock Seng Hospital	Chen YYC et al
EPI_ISL_462413	2020-04-07	National Public Health Laboratory, National Centre for Infectious Diseases	National Public Health Laboratory, National Centre for Infectious Diseases	Mak TM et al
EPI_ISL_480332	2020-04-07	Microbial Genomics Laboratory, Institut Pasteur de Montevideo	Microbial Genomics Laboratory, Institut Pasteur de Montevideo	Cecilia Salazar et al
EPI_ISL_480334	2020-04-07	Microbial Genomics Laboratory, Institut Pasteur de Montevideo	Microbial Genomics Laboratory, Institut Pasteur de Montevideo	Cecilia Salazar et al
EPI_ISL_480335	2020-04-07	Microbial Genomics Laboratory, Institut Pasteur de Montevideo	Microbial Genomics Laboratory, Institut Pasteur de Montevideo	Cecilia Salazar et al
EPI_ISL_451558	2020-04-08	Medlab Pathology	NSW Health Pathology - Institute of Clinical Pathology and Medical Research; Westmead Hospital; University of Sydney	CIDM-PH et al. et al
EPI_ISL_483560	2020-04-08	Kingdom of Bahrein Ministry of Health	Erasmus Medical Center	Bas Oude Munnink et al
EPI_ISL_483564	2020-04-08	Kingdom of Bahrein Ministry of Health	Erasmus Medical Center	Bas Oude Munnink et al
EPI_ISL_468077	2020-04-08	Child Health Research Foundation	Child Health Research Foundation	Senjuti Saha et al
EPI_ISL_467300	2020-04-08	General Hospital "Abdulah Nakas"	Alea Genetic Center	Rijad Konjhodzic et al

EPI_ISL_560390	2020-04-08	National Health Laboratory	Botswana Institute for Technology Research and Innovation	Kefentse Arnold Tumedi et al
EPI_ISL_722139	2020-04-08	DB Diagnosticos do Brasil	Laboratório de Parasitologia Médica - Instituto de Medicina Tropical - Universidade de São Paulo	Brazil-UK Centre for Arbovirus Discovery Diagnosis Genomics et al
EPI_ISL_722146	2020-04-08	DB Diagnosticos do Brasil	Laboratório de Parasitologia Médica - Instituto de Medicina Tropical - Universidade de São Paulo	Brazil-UK Centre for Arbovirus Discovery Diagnosis Genomics et al
EPI_ISL_470590	2020-04-08	Simile	Bioinformatics Laboratory / LNCC	Alexandra Gerber et al
EPI_ISL_470594	2020-04-08	Simile	Bioinformatics Laboratory / LNCC	Alexandra Gerber et al
EPI_ISL_572334	2020-04-08	LACEN/PE	WallauLab, Aggeu Magalhaes Institute	Marcelo Henrique Santos Paiva et al
EPI_ISL_500473	2020-04-08	LACEN/PE	WallauLab, Aggeu Magalhaes Institute	Marcelo Henrique Santos Paiva et al
EPI_ISL_500477	2020-04-08	LACEN/PE	WallauLab, Aggeu Magalhaes Institute	Marcelo Henrique Santos Paiva et al
EPI_ISL_500482	2020-04-08	LACEN/PE	WallauLab, Aggeu Magalhaes Institute	Marcelo Henrique Santos Paiva et al
EPI_ISL_500483	2020-04-08	LACEN/PE	WallauLab, Aggeu Magalhaes Institute	Marcelo Henrique Santos Paiva et al
EPI_ISL_500484	2020-04-08	LACEN/PE	WallauLab, Aggeu Magalhaes Institute	Marcelo Henrique Santos Paiva et al
EPI_ISL_500485	2020-04-08	LACEN/PE	WallauLab, Aggeu Magalhaes Institute	Marcelo Henrique Santos Paiva et al
EPI_ISL_717838	2020-04-08	Laboratorio de Virologia Molecular / UFRJ	Bioinformatics Laboratory / LNCC	Carolina M Voloch et al
EPI_ISL_513514	2020-04-08	Programa de Oncovirologia, Instituto Nacional de Câncer	Programa de Oncovirologia, Instituto Nacional de Câncer	Juliana D. Siqueira et al
EPI_ISL_480321	2020-04-08	Laboratorio Clínico San José	Charité Virology-University of Costa Rica	Andres Moreira-Soto et al
EPI_ISL_491119	2020-04-08	The National Institute of Public Health	The National Institute of Public Health and State Veterinary Institute Prague	Nagy et al
EPI_ISL_478772	2020-04-08	Oxford Viromics, NDM, University of Oxford; Oxford University Hospitals; Basingstoke and North Hampshire Hospital	COVID-19 Genomics UK (COG-UK) Consortium	Tanya Golubchik et al
EPI_ISL_508997	2020-04-08	CNR Virus des Infections Respiratoires - France SUD	CNR Virus des Infections Respiratoires - France SUD	Antonin Bal et al
EPI_ISL_629099	2020-04-08	Laboratoire du Centre Hospitalier Annecy Genevois	CNR Virus des Infections Respiratoires - France SUD	Antonin Bal et al
EPI_ISL_476075	2020-04-08	University of Debrecen, Department of Medical Microbiology	National Laboratory of Virology, Szentágothai Research Centre	Endre Gábor Tóth et al
EPI_ISL_434505	2020-04-08	Laboratoire National de Sante, Microbiology, Virology	Laboratoire National de Sante, Microbiology, Epidemiology and Microbial Genomics	Anke Wienecke-Baldacchino et al
EPI_ISL_449786	2020-04-08	Ostfold Hospital Trust - Kalnes, Centre for Laboratory Medicine, Section for gene technology and infection serology	Norwegian Institute of Public Health, Department of Virology	Kathrine Stene-Johansen et al
EPI_ISL_512818	2020-04-08	Kenema Government Hospital, Ministry of Health and Sanitation	Kenema Government Hospital, Ministry of Health and Sanitation	Goba et al
EPI_ISL_506994	2020-04-08	Division of Viral Diseases, Center for Laboratory Control of Infectious Diseases, Korea Centers for Diseases Control and Prevention	Division of Viral Diseases, Center for Laboratory Control of Infectious Diseases, Korea Centers for Diseases Control and Prevention	Jeong-Min Kim et al
EPI_ISL_548248	2020-04-08	Ostersund klinisk mikrobiologi	The Public Health Agency of Sweden	Anna-Malin Linde et al
EPI_ISL_513316	2020-04-09	South Eastern Area Laboratory Services (SEALS)	NSW Health Pathology - Institute of Clinical Pathology and Medical Research; Westmead Hospital; University of Sydney	CIDM-PH et al. et al
EPI_ISL_509714	2020-04-09	Belize Ministry of Health	Pathogen Discovery, Respiratory Viruses Branch, Division of Viral Diseases, Centers for Disease Control and Prevention	Jing Zhang et al
EPI_ISL_524795	2020-04-09	Evandro Chagas Institute	Evandro Chagas Institute	Santos et al
EPI_ISL_722136	2020-04-09	DB Diagnosticos do Brasil	Laboratório de Parasitologia Médica - Instituto de Medicina Tropical - Universidade de São Paulo	Brazil-UK Centre for Arbovirus Discovery Diagnosis Genomics et al
EPI_ISL_722140	2020-04-09	DB Diagnosticos do Brasil	Laboratório de Parasitologia Médica - Instituto de Medicina Tropical - Universidade de São Paulo	Brazil-UK Centre for Arbovirus Discovery Diagnosis Genomics et al
EPI_ISL_470593	2020-04-09	Simile	Bioinformatics Laboratory / LNCC	Alexandra Gerber et al
EPI_ISL_500462	2020-04-09	LACEN/PE	WallauLab, Aggeu Magalhaes Institute	Marcelo Henrique Santos Paiva et al
EPI_ISL_500463	2020-04-09	LACEN/PE	WallauLab, Aggeu Magalhaes Institute	Marcelo Henrique Santos Paiva et al
EPI_ISL_722013	2020-04-09	Hospital das Clínicas Universidade de São Paulo Medical School	Laboratório de Parasitologia Médica - Instituto de Medicina Tropical - Universidade de São Paulo	Brazil-UK Centre for Arbovirus Discovery Diagnosis Genomics et al
EPI_ISL_498164	2020-04-09	Instituto Nacional de Salud, Bogotá, Colombia	Instituto Nacional de Salud, Bogotá, Colombia	Katherine Laiton-Donato et al

EPI_ISL_468656	2020-04-09	Institute for Public Health	Laboratory for advanced genomics	Filip Rokić et al
EPI_ISL_454574	2020-04-09	nstitute for Public Health	Laboratory for advanced genomics	Filip Rokić et al
EPI_ISL_435032	2020-04-09	Viral Respiratory Lab, National Institute for Biomedical Research (INRB)	Pathogen Sequencing Lab, National Institute for Biomedical Research (INRB)	Placide Mbala-Kingebeni et al
EPI_ISL_476072	2020-04-09	University of Debrecen, Department of Medical Microbiology	National Laboratory of Virology, Szentágothai Research Centre	Endre Gábor Tóth et al
EPI_ISL_474977	2020-04-09	Israel Central Virology laboratory	Israel Central Virology laboratory	Neta Zuckerman et al
EPI_ISL_474959	2020-04-09	Israel Central Virology laboratory	Israel Central Virology laboratory	Neta Zuckerman et al
EPI_ISL_718149	2020-04-09	Ministry of Health Hospitals	Institute of Health and Community Medicine	David Perera et al
EPI_ISL_730031	2020-04-09	Nigeria Centre for Disease Control (NCDC)	African Centre of Excellence for Genomics of Infectious Diseases (ACEGID), Redeemer's University, Ede, Osun State, Nigeria	Oluniyi P.E. et al et al
EPI_ISL_496875	2020-04-09	Gorgas Memorial Laboratory of Health Studies	Gorgas Memorial Laboratory of Health Studies	Danilo Franco et al
EPI_ISL_496877	2020-04-09	Gorgas Memorial Laboratory of Health Studies	Gorgas Memorial Laboratory of Health Studies	Danilo Franco et al
EPI_ISL_496878	2020-04-09	Gorgas Memorial Laboratory of Health Studies	Gorgas Memorial Laboratory of Health Studies	Danilo Franco et al
EPI_ISL_496880	2020-04-09	Gorgas Memorial Laboratory of Health Studies	Gorgas Memorial Laboratory of Health Studies	Danilo Franco et al
EPI_ISL_481235	2020-04-09	Institut Pasteur Dakar	Institut Pasteur de Dakar	Ndongo Dia et al
EPI_ISL_635297	2020-04-09	Institute of Microbiology and Immunology, Faculty of Medicine, University of Ljubljana	Institute of Microbiology and Immunology, Faculty of Medicine, University of Ljubljana	Tomaž Mark Zorec et al
EPI_ISL_457967	2020-04-09	Laboratorio de Biología Molecular Asociación Española Primera en Salud	Departments of Pathology and Medicine, New York University School of Medicine	Maria Victoria Elizondo et al
EPI_ISL_476704	2020-04-09	Incubadora Venezolana de Ciencia, Venezuela	Incubadora Venezolana de Ciencia, Venezuela / Instituto Nacional de Salud, Bogotá, Colombia / Grupo de Investigaciones Microbiológicas-UR (GIMUR), Departamento de Biología, Facultad de Ciencias Naturales, Universidad del Rosario, Bogotá, Colombia / Icahn School of Medicine at Mount Sinai, New York, USA	Alberto Paniz-Mondolfi et al
EPI_ISL_455709	2020-04-09	National Hospital of Tropical Diseases	Oxford University Clinical Research Unit, Hanoi, Vietnam	Nguyen Thi Tam et al
EPI_ISL_455710	2020-04-09	National Hospital of Tropical Diseases	Oxford University Clinical Research Unit, Hanoi, Vietnam	Nguyen Thi Tam et al
EPI_ISL_583764	2020-04-10	Dr. Gernot Walder GmbH	Bergthaler laboratory, CeMM Research Center for Molecular Medicine of the Austrian Academy of Sciences	Alexandra Popa et al
EPI_ISL_468591	2020-04-10	Institute for Public Health	Laboratory for advanced genomics	Filip Rokić et al
EPI_ISL_481529	2020-04-10	Department of Virology and Immunology, University of Helsinki and Helsinki University Hospital, Huslab Finland	Department of Virology, Faculty of Medicine, University of Helsinki, Helsinki, Finland	Teemu Smura et al
EPI_ISL_718161	2020-04-10	Ministry of Health Hospitals	Institute of Health and Community Medicine	David Perera et al
EPI_ISL_487455	2020-04-10	CICM-Mali	Bundeswehr Institut of Microbiology	Kouriba et al
EPI_ISL_455242	2020-04-10	Dutch COVID-19 response team	Erasmus Medical Center	Bas Oude Munnink et al
EPI_ISL_512817	2020-04-10	Kenema Government Hospital, Ministry of Health and Sanitation	Kenema Government Hospital, Ministry of Health and Sanitation	Goba et al
EPI_ISL_660413	2020-04-10	Klinsisk mikrobiologi Linkoping	The Public Health Agency of Sweden	Anna-Malin Linde et al
EPI_ISL_480429	2020-04-10	Laboratorio de Biología Molecular Asociación Española Primera en Salud	Departments of Pathology and Medicine, New York University School of Medicine	Maria Victoria Elizondo et al
EPI_ISL_430813	2020-04-11	Laboratorio de Virología del Hospital de Niños Dr. Ricardo Gutierrez	Área de Secuenciación del Laboratorio de Virología del Hospital de Niños	Nabaes Jodar et al
EPI_ISL_451594	2020-04-11	Childrens Hospital Westmead	NSW Health Pathology - Institute of Clinical Pathology and Medical Research; Westmead Hospital; University of Sydney	CIDM-PH et al. et al
EPI_ISL_462461	2020-04-11	Clinical Center, University of Sarajevo	Charite Universitatsmedizin Berlin, Institute of Virology	Victor M Corman et al
EPI_ISL_672670	2020-04-11	DB Diagnosticos do Brasil	Laboratório de Parasitologia Médica - Instituto de Medicina Tropical - Universidade de São Paulo	Brazil-UK Centre for Arbovirus Discovery Diagnosis Genomics et al
EPI_ISL_672710	2020-04-11	Institute of Tropical Medicine at the University of São Paulo (IMT-USP)	Laboratório de Parasitologia Médica - Instituto de Medicina Tropical - Universidade de São Paulo	Brazil-UK Centre for Arbovirus Discovery Diagnosis Genomics et al
EPI_ISL_672712	2020-04-11	Institute of Tropical Medicine at the University of São Paulo (IMT-USP)	Laboratório de Parasitologia Médica - Instituto de Medicina Tropical - Universidade de São Paulo	Brazil-UK Centre for Arbovirus Discovery Diagnosis Genomics et al

EPI_ISL_586459	2020-04-11	Toronto Invasive Bacterial Diseases Network	McMaster University	Allison McGeer et al
EPI_ISL_463746	2020-04-11	Department of Molecular Virology, Cyprus Institute of Neurology and Genetics	Department of Molecular Virology, Cyprus Institute of Neurology and Genetics	Jan Richter et al
EPI_ISL_458083	2020-04-11	Adi Husada Undaan Hospital	Institute of Tropical Disease, Universitas Airlangga	Rima R Prasetya et al
EPI_ISL_568687	2020-04-11	RSUP Fatmawati	Eijkman Institute for Molecular Biology, Ministry of Research and Technology/National Agency for Research and Innovation	Frilasita A Yudhaputri et al
EPI_ISL_594187	2020-04-11	Department of Pathology, School of Medicine, Imam Khomeini Hospital, Tehran University of Medical Sciences	Genetics Research Center, University of Social Welfare and Rehabilitation Sciences	Zohreh Fattahi et al
EPI_ISL_525564	2020-04-11	Istituto Zooprofilattico Sperimentale Puglia e Basilicata; Dipartimento di Bioscienze, Biotecnologie e Biofarmaceutica dell'Università degli Studi di Bari "A.Moro"; Istituto di Biomembrane. Bioenergetica e Biotecnologie Molecolari del Consiglio Nazionale delle Ricerche di Bari	Beaconlab (Bioinformatics, Evolution and Comparative Genomics lab), Dept of Biosciences, University on Milan	Parisi A. et al
EPI_ISL_525565	2020-04-11	Istituto Zooprofilattico Sperimentale Puglia e Basilicata; Dipartimento di Bioscienze, Biotecnologie e Biofarmaceutica dell'Università degli Studi di Bari "A.Moro"; Istituto di Biomembrane. Bioenergetica e Biotecnologie Molecolari del Consiglio Nazionale delle Ricerche di Bari	Beaconlab (Bioinformatics, Evolution and Comparative Genomics lab), Dept of Biosciences, University on Milan	Parisi A. et al
EPI_ISL_525567	2020-04-11	Istituto Zooprofilattico Sperimentale Puglia e Basilicata; Dipartimento di Bioscienze, Biotecnologie e Biofarmaceutica dell'Università degli Studi di Bari "A.Moro"; Istituto di Biomembrane. Bioenergetica e Biotecnologie Molecolari del Consiglio Nazionale delle Ricerche di Bari	Beaconlab (Bioinformatics, Evolution and Comparative Genomics lab), Dept of Biosciences, University on Milan	Parisi A. et al
EPI_ISL_541869	2020-04-11	Lithuanian University of Health Sciences Hospital, Department of Laboratory Medicine	Lithuanian University of Health Sciences, Laboratory of Molecular Cardiology	Lukas Zemaitis et al
EPI_ISL_434493	2020-04-11	Laboratoire National de Sante, Microbiology, Virology	Laboratoire National de Sante, Microbiology, Epidemiology and Microbial Genomics	Anke Wienecke-Baldacchino et al
EPI_ISL_490048	2020-04-11	Institute for Medical Research, Infectious Disease Research Centre, National Institutes of Health, Ministry of Health Malaysia	Institute for Medical Research, Infectious Disease Research Centre, National Institutes of Health, Ministry of Health Malaysia	Suppiah J et al
EPI_ISL_514226	2020-04-11	Laboratorio de Referencia Nacional de Virus Respiratorio. Instituto Nacional de Salud Perú	Laboratorio de Referencia Nacional de Biotecnología y Biología Molecular. Instituto Nacional de Salud Perú	Carlos Padilla Rojas et al
EPI_ISL_678276	2020-04-11	Mikrobiologie, RARI	Mikrobiologie, RARI	Krasnov et al
EPI_ISL_486872	2020-04-11	Institut Pasteur Dakar	Institut Pasteur de Dakar	Ndongo Dia et al
EPI_ISL_512819	2020-04-11	Kenema Government Hospital, Ministry of Health and Sanitation	Kenema Government Hospital, Ministry of Health and Sanitation	Goba et al
EPI_ISL_648100	2020-04-11	Department of Laboratory Medicine, Tan Tock Seng Hospital	Department of Laboratory Medicine, Tan Tock Seng Hospital	Chen YYC et al
EPI_ISL_648101	2020-04-11	Department of Laboratory Medicine, Tan Tock Seng Hospital	Department of Laboratory Medicine, Tan Tock Seng Hospital	Chen YYC et al
EPI_ISL_468899	2020-04-11	Servicio de Microbiología. Hospital Universitario Donostia. OSI Donostialdea. Área de Enfermedades Infecciosas, Grupo de Infección Respiratoria y Resistencia Antimicrobiana. Instituto de Investigación Sanitaria Biodonostia.	SeqCOVID-SPAIN consortium/IBV(CSIC)	Gustavo Cilla et al
EPI_ISL_456606	2020-04-11	National Health Laboratory, Timor-Leste	Microbiological Diagnostic Unit Public Health Laboratory, The Peter Doherty Institute for Infection and Immunity	Soares da Silva et al
EPI_ISL_707793	2020-04-11	1-Laboratory of Microbiology, National Reference Lab, Charles Nicolle Hospital; 2-University of Tunis ElManar, Faculty of Medicine of Tunis, LR99ES09, Tunis, Tunisia	1-Clinical and Experimental Pharmacology Lab, LR16SP02, National Center of Pharmacovigilance, University of Tunis El Manar, Tunis, Tunisia. 2-Neurodegenerative diseases and psychiatric troubles, LR18SP03, Razi Hospital, University of Tunis El Manar, Tunis, Tunisia. 3- Ministry of Health, National Observatory of New and Emerging Diseases, 1006, Tunis, Tunisia	Ilhem Boutiba-Ben Boubaker et al
EPI_ISL_457968	2020-04-11	Laboratorio de Biología Molecular Asociación Española Primera en Salud	Departments of Pathology and Medicine, New York University School of Medicine	Maria Victoria Elizondo et al
EPI_ISL_722143	2020-04-12	DB Diagnosticos do Brasil	Laboratório de Parasitologia Médica - Instituto de Medicina Tropical - Universidade de São Paulo	Brazil-UK Centre for Arbovirus Discovery Diagnosis Genomics et al
EPI_ISL_463956	2020-04-12	Laboratoire de microbiologie, Hopital de Verdun	Smith Laboratory, Centre de Recherche CHU Sainte-Justine	Martin Smith et al

EPI_ISL_481540	2020-04-12	Department of Virology and Immunology, University of Helsinki and Helsinki University Hospital, Huslab Finland	Department of Virology, Faculty of Medicine, University of Helsinki, Helsinki, Finland	Teemu Smura et al
EPI_ISL_487457	2020-04-12	CICM-Mali	Bundeswehr Institut of Microbiology	Kouriba et al
EPI_ISL_487460	2020-04-12	CICM-Mali	Bundeswehr Institut of Microbiology	Kouriba et al
EPI_ISL_514227	2020-04-12	Laboratorio de Referencia Nacional de Virus Respiratorio. Instituto Nacional de Salud. Peru	Laboratorio de Referencia Nacional de Biotecnologia y Biologia Molecular.Instituto Nacional de Salud.Peru	Carlos Padilla Rojas et al
EPI_ISL_516646	2020-04-12	Laboratorio de Referencia Nacional de Virus Respiratorio. Centro Nacional de Salud Publica. Instituto Nacional de Salud Peru.	Laboratorio de Referencia Nacional de Biotecnología y Biología Molecular. Centro Nacional de Salud Publica. Instituto Nacional de Salud Peru.	Carlos Padilla Rojas et al
EPI_ISL_468149	2020-04-12	[Romania, Bucharest] National Institute for Infectious Diseases “Prof. Dr. Matei Balș”	[Romania, Bucharest] National Institute for Infectious Diseases “Prof. Dr. Matei Balș”	Leontina Banica et al
EPI_ISL_683671	2020-04-12	IdISSC/Hospital Clínico San Carlos de Madrid	SeqCOVID-SPAIN consortium/IBV(CSIC)	Alberto Delgado-Iribarren et al
EPI_ISL_463007	2020-04-12	Department of Laboratory Medicine, National Taiwan University Hospital	Microbial Genomics Core Lab, National Taiwan University Centers of Genomic and Precision Medicine	Shiou-Hwei Yeh et al
EPI_ISL_456604	2020-04-12	National Health Laboratory, Timor-Leste	Microbiological Diagnostic Unit Public Health Laboratory, The Peter Doherty Institute for Infection and Immunity	Soares da Silva et al
EPI_ISL_456605	2020-04-12	National Health Laboratory, Timor-Leste	Microbiological Diagnostic Unit Public Health Laboratory, The Peter Doherty Institute for Infection and Immunity	Soares da Silva et al
EPI_ISL_623105	2020-04-13	Simile Medicina Diagnóstica	Bioinformatics Laboratory / LNCC	Carolina M Voloch et al
EPI_ISL_470589	2020-04-13	Simile	Bioinformatics Laboratory / LNCC	Alexandra Gerber et al
EPI_ISL_500479	2020-04-13	LACEN/PE	WallauLab, Aggeu Magalhaes Institute	Marcelo Henrique Santos Paiva et al
EPI_ISL_500874	2020-04-13	LACEN/PE	WallauLab, Aggeu Magalhaes Institute	Marcelo Henrique Santos Paiva et al
EPI_ISL_500875	2020-04-13	LACEN/PE	WallauLab, Aggeu Magalhaes Institute	Marcelo Henrique Santos Paiva et al
EPI_ISL_541352	2020-04-13	Laboratory of Respiratory Viruses and Measles, Oswaldo Cruz Institute, FIOCRUZ	Laboratory of Respiratory Viruses and Measles, Oswaldo Cruz Institute, FIOCRUZ	Paola Resende et al
EPI_ISL_722144	2020-04-13	DB Diagnosticos do Brasil	Laboratório de Parasitologia Médica - Instituto de Medicina Tropical - Universidade de São Paulo	Brazil-UK Centre for Arbovirus Discovery Diagnosis Genomics et al
EPI_ISL_450648	2020-04-13	Laboratoire de microbiologie, Hopital de Verdun	Smith Laboratory, Centre de Recherche CHU Sainte-Justine	Martin Smith et al
EPI_ISL_574609	2020-04-13	RSJPD Harapan Kita	Eijkman Institute for Molecular Biology, Ministry of Research and Technology/National Agency for Research and Innovation	Frilasita A Yudhaputri et al
EPI_ISL_594185	2020-04-13	Department of Pathology, School of Medicine, Imam Khomeini Hospital, Tehran University of Medical Sciences	Genetics Research Center, University of Social Welfare and Rehabilitation Sciences	Zohreh Fattahi et al
EPI_ISL_603097	2020-04-13	Lithuanian University of Health Sciences Hospital, Department of Laboratory Medicine	Lithuanian University of Health Sciences, Molecular cardiology lab.	Lukas Zemaitis et al
EPI_ISL_718153	2020-04-13	Ministry of Health Hospitals	Institute of Health and Community Medicine	David Perera et al
EPI_ISL_457982	2020-04-13	Oman-NIC	Department of Microbiology and Immunology-SQUH	Fahad Zadjali et al
EPI_ISL_527789	2020-04-13	Laboratorio de Referencia Nacional de Virus Respiratorio. Centro Nacional de Salud Publica. Instituto Nacional de Salud Peru.	Laboratorio de Referencia Nacional de Biotecnología y Biología Molecular. Centro Nacional de Salud Publica. Instituto Nacional de Salud Peru.	Carlos Padilla Rojas et al
EPI_ISL_676593	2020-04-13	Scientific Veterinary Institute Novi Sad	Veterinary Specialized Institute "Kraljevo", Serbia	Vidanovic et al
EPI_ISL_577737	2020-04-13	Institute of Virology, Biomedical Research Center of the Slovak Academy of Sciences, Bratislava	Faculty of Natural Sciences, Comenius University, Bratislava	Kristína Boršová et al
EPI_ISL_445231	2020-04-13	Uppsala Narakut Aleris	The Public Health Agency of Sweden	Annika Nilsson et al
EPI_ISL_447621	2020-04-13	Department of Laboratory Medicine, National Taiwan University Hospital	Microbial Genomics Core Lab, National Taiwan University Centers of Genomic and Precision Medicine	Shiou-Hwei Yeh et al
EPI_ISL_480345	2020-04-13	Microbial Genomics Laboratory, Institut Pasteur de Montevideo	Microbial Genomics Laboratory, Institut Pasteur de Montevideo	Cecilia Salazar et al
EPI_ISL_561935	2020-04-14	Victorian Infectious Diseases Reference Laboratory (VIDRL)	VIDRL and MDU-PHL	Caly et al
EPI_ISL_560389	2020-04-14	National Health Laboratory	Botswana Institute for Technology Research and Innovation	Kefentse Arnold Tumedi et al
EPI_ISL_672675	2020-04-14	DB Diagnosticos do Brasil	Laboratório de Parasitologia Médica - Instituto de Medicina Tropical - Universidade de São Paulo	Brazil-UK Centre for Arbovirus Discovery Diagnosis Genomics et al

EPI_ISL_722135	2020-04-14	DB Diagnosticos do Brasil	Laboratório de Parasitologia Médica - Instituto de Medicina Tropical - Universidade de São Paulo	Brazil-UK Centre for Arbovirus Discovery Diagnosis Genomics et al
EPI_ISL_672717	2020-04-14	Institute of Tropical Medicine at the University of São Paulo (IMT-USP)	Laboratório de Parasitologia Médica - Instituto de Medicina Tropical - Universidade de São Paulo	Brazil-UK Centre for Arbovirus Discovery Diagnosis Genomics et al
EPI_ISL_672718	2020-04-14	Institute of Tropical Medicine at the University of São Paulo (IMT-USP)	Laboratório de Parasitologia Médica - Instituto de Medicina Tropical - Universidade de São Paulo	Brazil-UK Centre for Arbovirus Discovery Diagnosis Genomics et al
EPI_ISL_480300	2020-04-14	National Reference Laboratory "Influenza and acute respiratory diseases"	NRL-HIV	Ivan Ivanov et al
EPI_ISL_434572	2020-04-14	The National Institute of Public Health Center for Epidemiology and Microbiology	The National Institute of Public Health Center for Epidemiology and Microbiology	Alexander Nagy et al
EPI_ISL_497865	2020-04-14	Department of Microbiology, The University of Hong Kong	Department of Microbiology, The University of Hong Kong	Kelvin K.W. To et al
EPI_ISL_476077	2020-04-14	University of Debrecen, Department of Medical Microbiology	National Laboratory of Virology, Szentágothai Research Centre	Endre Gábor Tóth et al
EPI_ISL_644333	2020-04-14	CEPHR / Vincent's Hospital	Irish Coronavirus Sequencing Consortium - National Virus Reference Laboratory	Michael Carr et al
EPI_ISL_454576	2020-04-14	Laboratory of virology, National Center of Expertise	Laboratory of molecular-genetic research, National Center of Expertise, Kazakhstan National Center for Biotechnology, Kazakhstan	Abdaliyev Askar et al
EPI_ISL_450519	2020-04-14	E. Gulbja Laboratorija	Latvian Biomedical Research and Study Centre	Ivars Silamiķelis et al
EPI_ISL_487465	2020-04-14	CICM-Mali	Bundeswehr Institut of Microbiology	Kouriba et al
EPI_ISL_664106	2020-04-14	Dept. of Microbiology and Infection Control, Akershus University Hospital HF	Dept. of Microbiology and Infection Control, Akershus University Hospital HF	Hege Vangstein Aamot et al
EPI_ISL_496907	2020-04-14	Gorgas Memorial Laboratory of Health Studies	Gorgas Memorial Laboratory of Health Studies	Danilo Franco et al
EPI_ISL_456607	2020-04-14	National Health Laboratory, Timor-Leste	Microbiological Diagnostic Unit Public Health Laboratory, The Peter Doherty Institute for Infection and Immunity	Soares da Silva et al
EPI_ISL_457969	2020-04-14	Laboratorio de Biología Molecular Asociación Española Primera en Salud	Departments of Pathology and Medicine, New York University School of Medicine	Maria Victoria Elizondo et al
EPI_ISL_575330	2020-04-15	National Institute for Viral Disease Control and Prevention, China CDC	National Institute for Viral Disease Control and Prevention, China CDC	Rongbao Gao et al
EPI_ISL_476830	2020-04-15	Laboratoire des Fièvres Hémorragiques Virales du Benin	Charité-Universitätsmedizin Berlin	Yadouleton et al
EPI_ISL_476831	2020-04-15	Laboratoire des Fièvres Hémorragiques Virales du Benin	Charité-Universitätsmedizin Berlin	Yadouleton et al
EPI_ISL_458144	2020-04-15	Evandro Chagas Institute	Evandro Chagas Institute	Santos et al
EPI_ISL_458143	2020-04-15	Evandro Chagas Institute	Evandro Chagas Institute	Santos et al
EPI_ISL_470592	2020-04-15	Simile	Bioinformatics Laboratory / LNCC	Alexandra Gerber et al
EPI_ISL_524787	2020-04-15	Evandro Chagas Institute	Evandro Chagas Institute	Santos et al
EPI_ISL_722149	2020-04-15	DB Diagnosticos do Brasil	Laboratório de Parasitologia Médica - Instituto de Medicina Tropical - Universidade de São Paulo	Brazil-UK Centre for Arbovirus Discovery Diagnosis Genomics et al
EPI_ISL_583490	2020-04-15	Hospital Estadual Sumare	Instituto Adolfo Lutz, Interdiciplinary Procedures Center, Strategic Laboratory	Claudio Tavares Sacchi et al
EPI_ISL_583492	2020-04-15	Santa Casa Anna Cintra	Instituto Adolfo Lutz, Interdiciplinary Procedures Center, Strategic Laboratory	Claudio Tavares Sacchi et al
EPI_ISL_437341	2020-04-15	Viral Respiratory Lab, National Institute for Biomedical Research (INRB)	Pathogen Sequencing Lab, National Institute for Biomedical Research (INRB)	Placide Mbala-Kingebeni et al
EPI_ISL_452058	2020-04-15	Department of Clinical Microbiology, Copenhagen University Hospital, Hvidovre, Kettegaard Alle 30, 2650 Hvidovre.	Albertsen lab, Department of Chemistry and Bioscience, Aalborg University, Denmark	Rasmus Kirkegaard et al
EPI_ISL_481556	2020-04-15	Department of Virology and Immunology, University of Helsinki and Helsinki University Hospital, Huslab Finland	Department of Virology, Faculty of Medicine, University of Helsinki, Helsinki, Finland	Teemu Smura et al
EPI_ISL_481559	2020-04-15	Department of Virology and Immunology, University of Helsinki and Helsinki University Hospital, Huslab Finland	Department of Virology, Faculty of Medicine, University of Helsinki, Helsinki, Finland	Teemu Smura et al

EPI_ISL_470877	2020-04-15	Department for Virology, Molecular Biology and Genome Research, R. G. Lugar Center for Public Health Research, National Center for Disease Control and Public Health (NCDC) of Georgia.	Department for Virology, Molecular Biology and Genome Research, R. G. Lugar Center for Public Health Research, National Center for Disease Control and Public Health (NCDC) of Georgia.	Gvantsa Brachveli et al
EPI_ISL_613706	2020-04-15	Laboratorio Biologia Molecolare Sars Cov2 - UOC Laboratorio Analisi - Servizio Medicina di Laboratorio, Ospedale "San Francesco" - ATS-ASSL Nuoro	Laboratorio specialistico UOC Ematologia - Ospedale "San Francesco" - ATS-ASSL Nuoro	Piras Giovanna et al
EPI_ISL_454577	2020-04-15	Laboratory of virology, National Center of Expertise	Laboratory of molecular-genetic research, National Center of Expertise, Kazakhstan National Center for Biotechnology, Kazakhstan	Abdaliyev Askar et al
EPI_ISL_457921	2020-04-15	KEMRI-CGMR-C	KEMRI-Wellcome Trust Research Programme/KEMRI-CGMR-C Kilifi	Githinji G. et al 2020 et al
EPI_ISL_456384	2020-04-15	LabPLUS	Institute of Environmental Science and Research (ESR)	Matt Storey et al
EPI_ISL_468151	2020-04-15	[Romania, Bucharest] National Institute for Infectious Diseases “Prof. Dr. Matei Balș”	[Romania, Bucharest] National Institute for Infectious Diseases “Prof. Dr. Matei Balș”	Leontina Banica et al
EPI_ISL_430110	2020-04-15	WHO National Influenza Centre Russian Federation	WHO National Influenza Centre Russian Federation	Andrey Komissarov et al
EPI_ISL_581703	2020-04-15	University Hospital Basel, Clinical Virology	University Hospital Basel, Clinical Bacteriology	Madlen Stange et al
EPI_ISL_460618	2020-04-15	unknown	Physiology	Pence et al
EPI_ISL_481225	2020-04-16	Lab voor klinische biologie	Onderzoeksgroep Virologie	Laurens Lambrechts et al
EPI_ISL_560388	2020-04-16	National Health Laboratory	Botswana Institute for Technology Research and Innovation	Kefentse Arnold Tumedi et al
EPI_ISL_470596	2020-04-16	Simile	Bioinformatics Laboratory / LNCC	Alexandra Gerber et al
EPI_ISL_623108	2020-04-16	Laboratorio de Virologia Molecular / UFRJ	Bioinformatics Laboratory / LNCC	Carolina M Voloch et al
EPI_ISL_513530	2020-04-16	Programa de Oncovirologia, Instituto Nacional de Câncer	Programa de Oncovirologia, Instituto Nacional de Câncer	Juliana D. Siqueira et al
EPI_ISL_513532	2020-04-16	Programa de Oncovirologia, Instituto Nacional de Câncer	Programa de Oncovirologia, Instituto Nacional de Câncer	Juliana D. Siqueira et al
EPI_ISL_513541	2020-04-16	Programa de Oncovirologia, Instituto Nacional de Câncer	Programa de Oncovirologia, Instituto Nacional de Câncer	Juliana D. Siqueira et al
EPI_ISL_437348	2020-04-16	Viral Respiratory Lab, National Institute for Biomedical Research (INRB)	Pathogen Sequencing Lab, National Institute for Biomedical Research (INRB)	Placide Mbala-Kingebeni et al
EPI_ISL_534803	2020-04-16	Oxford Viromics, NDM, University of Oxford; Oxford University Hospitals; Basingstoke and North Hampshire Hospital	COVID-19 Genomics UK (COG-UK) Consortium	Tanya Golubchik et al
EPI_ISL_640230	2020-04-16	MVZ Laborärzte Singen	MVZ Laborärzte Singen	Jonas Schmidt et al
EPI_ISL_444969	2020-04-16	Guangzhou Eighth People's Hospital (Jiahe Sector)	Institute of Human Virology, Zhongshan School of Medicine, Sun Yat-sen University	Junsong Zhang et al
EPI_ISL_526232	2020-04-16	Hungarian Defence Forces Military Medical Centre	National Laboratory of Virology, Szentágothai Research Centre	Endre Gábor Tóth et al
EPI_ISL_454579	2020-04-16	Laboratory of virology, National Center of Expertise	Laboratory of molecular-genetic research, National Center of Expertise, Kazakhstan National Center for Biotechnology, Kazakhstan	Abdaliyev Askar et al
EPI_ISL_454580	2020-04-16	Laboratory of virology, National Center of Expertise	Laboratory of molecular-genetic research, National Center of Expertise, Kazakhstan National Center for Biotechnology, Kazakhstan	Abdaliyev Askar et al
EPI_ISL_527787	2020-04-16	Laboratorio de Referencia Nacional de Virus Respiratorio. Centro Nacional de Salud Publica. Instituto Nacional de Salud Peru.	Laboratorio de Referencia Nacional de Biotecnología y Biología Molecular. Centro Nacional de Salud Publica. Instituto Nacional de Salud Peru.	Carlos Padilla Rojas et al
EPI_ISL_468152	2020-04-16	[Romania, Bucharest] National Institute for Infectious Diseases “Prof. Dr. Matei Balș”	[Romania, Bucharest] National Institute for Infectious Diseases “Prof. Dr. Matei Balș”	Leontina Banica et al
EPI_ISL_450247	2020-04-16	WHO National Influenza Centre Russian Federation	WHO National Influenza Centre Russian Federation	Andrey Komissarov et al
EPI_ISL_460617	2020-04-16	unknown	Physiology	Pence et al
EPI_ISL_468066	2020-04-16	Physiology, Istanbul Medeniyet University	Physiology, Istanbul Medeniyet University	Pence et al
EPI_ISL_460619	2020-04-16	unknown	Physiology	Pence et al
EPI_ISL_430814	2020-04-17	Laboratorio de Virología del Hospital de Niños Dr. Ricardo Gutierrez	Área de Secuenciación del Laboratorio de Virología del Hospital de Niños	Nabaes Jodar et al

EPI_ISL_470591	2020-04-17	Simile	Bioinformatics Laboratory / LNCC	Alexandra Gerber et al
EPI_ISL_623140	2020-04-17	Laboratorio de Virologia Molecular / UFRJ	Bioinformatics Laboratory / LNCC	Carolina M Voloch et al
EPI_ISL_513549	2020-04-17	Programa de Oncovirologia, Instituto Nacional de Câncer	Programa de Oncovirologia, Instituto Nacional de Câncer	Juliana D. Siqueira et al
EPI_ISL_527860	2020-04-17	Hospital Municipal de Parelheiros Josanias Castanha Braga	Instituto Adolfo Lutz, Interdiciplinary Procedures Center, Strategic Laboratory	Claudio Tavares Sacchi et al
EPI_ISL_463747	2020-04-17	Department of Molecular Virology, Cyprus Institute of Neurology and Genetics	Department of Molecular Virology, Cyprus Institute of Neurology and Genetics	Jan Richter et al
EPI_ISL_471267	2020-04-17	Hospital IESS Babahoyo	Institute of Microbiology, Universidad San Francisco de Quito	Sully Márquez et al
EPI_ISL_481245	2020-04-17	Hospital IESS Babahoyo	Institute of Microbiology, Universidad San Francisco de Quito	Belén Prado-Vivar et al
EPI_ISL_481246	2020-04-17	Hospital IESS Babahoyo	Institute of Microbiology, Universidad San Francisco de Quito	Belén Prado-Vivar et al
EPI_ISL_481247	2020-04-17	Hospital IESS Babahoyo	Institute of Microbiology, Universidad San Francisco de Quito	Belén Prado-Vivar et al
EPI_ISL_481248	2020-04-17	Hospital IESS Babahoyo	Institute of Microbiology, Universidad San Francisco de Quito	Belén Prado-Vivar et al
EPI_ISL_481579	2020-04-17	Department of Virology and Immunology, University of Helsinki and Helsinki University Hospital, Huslab Finland	Department of Virology, Faculty of Medicine, University of Helsinki, Helsinki, Finland	Teemu Smura et al
EPI_ISL_529018	2020-04-17	Ospedale "Giuseppe Mazzini"-Teramo	Istitituto Zooprofilattico Sperimentale dell'Abruzzo e Molise "G.Caporale"	Lorusso A et al
EPI_ISL_468153	2020-04-17	[Romania, Bucharest] National Institute for Infectious Diseases “Prof. Dr. Matei Balș”	[Romania, Bucharest] National Institute for Infectious Diseases “Prof. Dr. Matei Balș”	Leontina Banica et al
EPI_ISL_456612	2020-04-17	National Health Laboratory, Timor-Leste	Microbiological Diagnostic Unit Public Health Laboratory, The Peter Doherty Institute for Infection and Immunity	Soares da Silva et al
EPI_ISL_471854	2020-04-17	Michigan Department of Health and Human Services, Bureau of Laboratories	Michigan Department of Health and Human Services, Bureau of Laboratories	Blankenship HM et al
EPI_ISL_520669	2020-04-17	Mohammed Bin Rashid University of Medicine and Health Sciences	Al Jalila Genomics Center	Ahmad Abou Tayoun et al
EPI_ISL_430815	2020-04-18	Laboratorio de Virología del Hospital de Niños Dr. Ricardo Gutierrez	Área de Secuenciación del Laboratorio de Virología del Hospital de Niños	Nabaes Jodar et al
EPI_ISL_430817	2020-04-18	Laboratorio de Virología del Hospital de Niños Dr. Ricardo Gutierrez	Área de Secuenciación del Laboratorio de Virología del Hospital de Niños	Nabaes Jodar et al
EPI_ISL_491139	2020-04-18	Oman-National Influenza Center	Biotechnology & OMICs Laboratory	Samiha Al-Kharusi et al
EPI_ISL_468155	2020-04-18	[Romania, Bucharest] National Institute for Infectious Diseases “Prof. Dr. Matei Balș”	[Romania, Bucharest] National Institute for Infectious Diseases “Prof. Dr. Matei Balș”	Leontina Banica et al
EPI_ISL_520665	2020-04-18	Mohammed Bin Rashid University of Medicine and Health Sciences	Al Jalila Genomics Center	Ahmad Abou Tayoun et al
EPI_ISL_520671	2020-04-18	Mohammed Bin Rashid University of Medicine and Health Sciences	Al Jalila Genomics Center	Ahmad Abou Tayoun et al
EPI_ISL_523990	2020-04-19	AMA Jardim Peri	Instituto Adolfo Lutz, Interdiciplinary Procedures Center, Strategic Laboratory	Claudio Tavares Sacchi et al
EPI_ISL_480309	2020-04-19	National Reference Laboratory "Influenza and acute respiratory diseases"	NRL-HIV	Ivan Ivanov et al
EPI_ISL_525474	2020-04-19	Centre for Dengue Research	Centre for Dengue Research, USJ, SL	Chandima Jeewandara et al
EPI_ISL_582634	2020-04-19	Sheikh Khalifa Medical City	Molecular/Surveillance lab Sheikh Khalifa Medical City	Amirtharaj Francis et al
EPI_ISL_582633	2020-04-19	Sheikh Khalifa Medical City	Molecular/Surveillance lab Sheikh Khalifa Medical City	Amirtharaj Francis et al
EPI_ISL_458145	2020-04-20	Evandro Chagas Institute	Evandro Chagas Institute	Santos et al
EPI_ISL_572335	2020-04-20	LACEN/PE	WallauLab, Aggeu Magalhaes Institute	Marcelo Henrique Santos Paiva et al
EPI_ISL_729834	2020-04-20	Laboratorio Central de Saude Publica do Estado do Rio Grande do Sul (LACEN-RS)	Laboratory of Respiratory Viruses and Measles, Oswaldo Cruz Institute, FIOCRUZ	Paola Resende et al
EPI_ISL_541388	2020-04-20	LACEN/SE	Laboratory of Respiratory Viruses and Measles, Oswaldo Cruz Institute, FIOCRUZ	Paola Resende et al
EPI_ISL_541389	2020-04-20	LACEN/SE	Laboratory of Respiratory Viruses and Measles, Oswaldo Cruz Institute, FIOCRUZ	Paola Resende et al

EPI_ISL_541392	2020-04-20	LACEN/SE	Laboratory of Respiratory Viruses and Measles, Oswaldo Cruz Institute, FIOCRUZ	Paola Resende et al
EPI_ISL_541394	2020-04-20	LACEN/SE	Laboratory of Respiratory Viruses and Measles, Oswaldo Cruz Institute, FIOCRUZ	Paola Resende et al
EPI_ISL_541395	2020-04-20	LACEN/SE	Laboratory of Respiratory Viruses and Measles, Oswaldo Cruz Institute, FIOCRUZ	Paola Resende et al
EPI_ISL_541396	2020-04-20	LACEN/SE	Laboratory of Respiratory Viruses and Measles, Oswaldo Cruz Institute, FIOCRUZ	Paola Resende et al
EPI_ISL_622512	2020-04-20	Department of Virus and Microbiological Special Diagnostics, Statens Serum Institut, Denmark	Albertsen lab, Department of Chemistry and Bioscience, Aalborg University, Denmark	Danish Covid-19 Genome Consortia et al
EPI_ISL_622645	2020-04-20	Department of Virus and Microbiological Special Diagnostics, Statens Serum Institut, Denmark	Albertsen lab, Department of Chemistry and Bioscience, Aalborg University, Denmark	Danish Covid-19 Genome Consortia et al
EPI_ISL_447333	2020-04-20	Clinical Virology Unit, Hadassah Hebrew University Medical Center	Stern Lab	Stern Lab et al
EPI_ISL_729938	2020-04-20	Nigeria Centre for Disease Control (NCDC)	African Centre of Excellence for Genomics of Infectious Diseases (ACEGID), Redeemer's University, Ede, Osun State, Nigeria	Oluniyi P.E. et al et al
EPI_ISL_486873	2020-04-20	Institut Pasteur Dakar	Institut Pasteur de Dakar	Ndongo Dia et al
EPI_ISL_451199	2020-04-20	Uganda Virus Research Institute	MRC/UVRI & LSHTM Uganda Research Unit	Dan Lule Bugembe et al
EPI_ISL_462466	2020-04-21	Clinical Center, University of Sarajevo	Charite Universitatsmedizin Berlin, Institute of Virology	Victor M Corman et al
EPI_ISL_541393	2020-04-21	LACEN/SE	Laboratory of Respiratory Viruses and Measles, Oswaldo Cruz Institute, FIOCRUZ	Paola Resende et al
EPI_ISL_539780	2020-04-21	National Institute of Public Health (Czech Republic)	State Veterinary Institute Prague	Nagy et al
EPI_ISL_479095	2020-04-21	Oxford Viromics, NDM, University of Oxford; Oxford University Hospitals; Basingstoke and North Hampshire Hospital	COVID-19 Genomics UK (COG-UK) Consortium	Tanya Golubchik et al
EPI_ISL_648315	2020-04-21	Laboratorio de Investigaciones de Baney	University Hospital Basel, Clinical Bacteriology	Carlos Cortes et al
EPI_ISL_629107	2020-04-21	Laboratoire du Centre Hospitalier Annecy Genevois	CNR Virus des Infections Respiratoires - France SUD	Antonin Bal et al
EPI_ISL_459977	2020-04-21	Institut Pasteur du Maroc	Institut Pasteur du Maroc	Marion Barbet et al
EPI_ISL_459983	2020-04-21	Institut Pasteur du Maroc	Institut Pasteur du Maroc	Marion Barbet et al
EPI_ISL_512980	2020-04-21	Pathogen Genomics Lab King Abdullah University of Science and Technology(KAUST)	Pathogen Genomics Lab King Abdullah University of Science and Technology(KAUST)	Sara Mfarrej et al
EPI_ISL_486871	2020-04-21	Institut Pasteur Dakar	Institut Pasteur de Dakar	Ndongo Dia et al
EPI_ISL_509705	2020-04-21	Wisconsin Department of Health Services	Pathogen Discovery, Respiratory Viruses Branch, Division of Viral Diseases, Centers for Disease Control and Prevention	Ying Tao et al
EPI_ISL_476496	2020-04-22	Hospital Garrahan	Héritas	Dalmacio Pereyra et al
EPI_ISL_462470	2020-04-22	Clinical Center, University of Sarajevo	Charite Universitatsmedizin Berlin, Institute of Virology	Victor M Corman et al
EPI_ISL_572349	2020-04-22	LACEN/PE	WallauLab, Aggeu Magalhaes Institute	Marcelo Henrique Santos Paiva et al
EPI_ISL_623130	2020-04-22	Laboratorio de Virologia Molecular / UFRJ	Bioinformatics Laboratory / LNCC	Carolina M Voloch et al
EPI_ISL_623142	2020-04-22	Laboratorio de Virologia Molecular / UFRJ	Bioinformatics Laboratory / LNCC	Carolina M Voloch et al
EPI_ISL_513556	2020-04-22	Programa de Oncovirologia, Instituto Nacional de Câncer	Programa de Oncovirologia, Instituto Nacional de Câncer	Juliana D. Siqueira et al
EPI_ISL_513557	2020-04-22	Programa de Oncovirologia, Instituto Nacional de Câncer	Programa de Oncovirologia, Instituto Nacional de Câncer	Juliana D. Siqueira et al
EPI_ISL_513560	2020-04-22	Programa de Oncovirologia, Instituto Nacional de Câncer	Programa de Oncovirologia, Instituto Nacional de Câncer	Juliana D. Siqueira et al
EPI_ISL_513561	2020-04-22	Programa de Oncovirologia, Instituto Nacional de Câncer	Programa de Oncovirologia, Instituto Nacional de Câncer	Juliana D. Siqueira et al
EPI_ISL_527867	2020-04-22	Pronto Socorro Municipal - Balneario São José	Instituto Adolfo Lutz, Interdiciplinary Procedures Center, Strategic Laboratory	Claudio Tavares Sacchi et al
EPI_ISL_527869	2020-04-22	Hospital Municipal Carmen Prudente	Instituto Adolfo Lutz, Interdiciplinary Procedures Center, Strategic Laboratory	Claudio Tavares Sacchi et al
EPI_ISL_468321	2020-04-22	Hospital Universitario da USP	Instituto Adolfo Lutz, Interdiciplinary Procedures Center, Strategic Laboratory	Claudio Tavares Sacchi et al
EPI_ISL_722072	2020-04-22	Hospital das Clínicas Universidade de São Paulo Medical School	Laboratório de Parasitologia Médica - Instituto de Medicina Tropical - Universidade de São Paulo	Brazil-UK Centre for Arbovirus Discovery Diagnosis Genomics et al

EPI_ISL_722009	2020-04-22	Hospital das Clínicas Universidade de São Paulo Medical School	Laboratório de Parasitologia Médica - Instituto de Medicina Tropical - Universidade de São Paulo	Brazil-UK Centre for Arbovirus Discovery Diagnosis Genomics et al
EPI_ISL_491945	2020-04-22	Instituto Nacional de Investigación en Salud Pública - INSPI	INSPI - Charité	Alfredo Bruno Caicedo et al
EPI_ISL_501248	2020-04-22	Hellenic Pasteur Institute, National Influenza Reference laboratory of Southern Greece & Unit of Bioinformatics and Applied Genomics	Hellenic Pasteur Institute, National Influenza Reference laboratory of Southern Greece & Unit of Bioinformatics and Applied Genomics	Vasiliki Pogka et al
EPI_ISL_501249	2020-04-22	Hellenic Pasteur Institute, National Influenza Reference laboratory of Southern Greece & Unit of Bioinformatics and Applied Genomics	Hellenic Pasteur Institute, National Influenza Reference laboratory of Southern Greece & Unit of Bioinformatics and Applied Genomics	Vasiliki Pogka et al
EPI_ISL_477013	2020-04-22	University of Debrecen, Department of Medical Microbiology	National Laboratory of Virology, Szentágothai Research Centre	Endre Gábor Tóth et al
EPI_ISL_457867	2020-04-22	KEMRI-CGMR-C	KEMRI-Wellcome Trust Research Programme/KEMRI-CGMR-C Kilifi	Githinji G. et al 2020 et al
EPI_ISL_496365	2020-04-22	Infectolab	Andersen lab at Scripps Research	SEARCH Alliance San Diego with Samuel Navarro Alvarez et al
EPI_ISL_496367	2020-04-22	Infectolab	Andersen lab at Scripps Research	SEARCH Alliance San Diego with Samuel Navarro Alvarez et al
EPI_ISL_512844	2020-04-22	Department of Medical Research	DMR_Myanmar	Myat Htut Nyunt et al
EPI_ISL_491162	2020-04-22	Oman-National Influenza Center	Biotechnology & OMICs Laboratory	Sajjad Asaf et al
EPI_ISL_677939	2020-04-22	Pathogen Genomics Lab King Abdullah University of Science and Technology(KAUST)	Pathogen Genomics Lab King Abdullah University of Science and Technology(KAUST)	Sara Mfarrej et al
EPI_ISL_524796	2020-04-23	Evandro Chagas Institute	Evandro Chagas Institute	Santos et al
EPI_ISL_458146	2020-04-23	Evandro Chagas Institute	Evandro Chagas Institute	Santos et al
EPI_ISL_523986	2020-04-23	Ama Dr Jose Soares Hungria	Instituto Adolfo Lutz, Interdiciplinary Procedures Center, Strategic Laboratory	Claudio Tavares Sacchi et al
EPI_ISL_476417	2020-04-23	Laboratório de Patologia Clínica - UNICAMP	Laboratório de Estudos de Vírus Emergentes - UNICAMP	José Luiz Proença-Modena et al
EPI_ISL_648321	2020-04-23	Laboratorio de Investigaciones de Baney	University Hospital Basel, Clinical Bacteriology	Carlos Cortes et al
EPI_ISL_574610	2020-04-23	Puskesmas Tambora	Eijkman Institute for Molecular Biology, Ministry of Research and Technology/National Agency for Research and Innovation	Frilasita A Yudhaputri et al
EPI_ISL_454582	2020-04-23	Laboratory of virology, National Center of Expertise	Laboratory of molecular-genetic research, National Center of Expertise, Kazakhstan National Center for Biotechnology, Kazakhstan	Abdaliyev Askar et al
EPI_ISL_491434	2020-04-23	Laboratorio de Referencia Nacional de Virus Respiratorio. Instituto Nacional de Salud Perú	Laboratorio de Referencia Nacional de Biotecnología y Biología Molecular. Instituto Nacional de Salud Perú	Carlos Padilla Rojas et al
EPI_ISL_491435	2020-04-23	Laboratorio de Referencia Nacional de Virus Respiratorio. Instituto Nacional de Salud Perú	Laboratorio de Referencia Nacional de Biotecnología y Biología Molecular. Instituto Nacional de Salud Perú	Carlos Padilla Rojas et al
EPI_ISL_512946	2020-04-23	Pathogen Genomics Lab King Abdullah University of Science and Technology(KAUST)	Pathogen Genomics Lab King Abdullah University of Science and Technology(KAUST)	Sara Mfarrej et al
EPI_ISL_462437	2020-04-23	unknown	Laboratory Diagnostic	Vidanovic et al
EPI_ISL_654883	2020-04-23	Pasteur Institute in Ho Chi Minh city	Department of Microbiology and Immunology - Pasteur Institute in Ho Chi Minh city	Nguyễn Thanh Long et al
EPI_ISL_524788	2020-04-24	Evandro Chagas Institute	Evandro Chagas Institute	Santos et al
EPI_ISL_458147	2020-04-24	Evandro Chagas Institute	Evandro Chagas Institute	Santos et al
EPI_ISL_623114	2020-04-24	Laboratorio de Virologia Molecular / UFRJ	Bioinformatics Laboratory / LNCC	Carolina M Voloch et al
EPI_ISL_513567	2020-04-24	Programa de Oncovirologia, Instituto Nacional de Câncer	Programa de Oncovirologia, Instituto Nacional de Câncer	Juliana D. Siqueira et al
EPI_ISL_541386	2020-04-24	LACEN/SE	Laboratory of Respiratory Viruses and Measles, Oswaldo Cruz Institute, FIOCRUZ	Paola Resende et al
EPI_ISL_541387	2020-04-24	LACEN/SE	Laboratory of Respiratory Viruses and Measles, Oswaldo Cruz Institute, FIOCRUZ	Paola Resende et al
EPI_ISL_648326	2020-04-24	Laboratorio de Investigaciones de Baney	University Hospital Basel, Clinical Bacteriology	Carlos Cortes et al

EPI_ISL_501230	2020-04-24	Hellenic Pasteur Institute, Public Health Laboratories	Hellenic Pasteur Institute, National Influenza Reference laboratory of Southern Greece & Unit of Bioinformatics and Applied Genomics	Vasiliki Pogka et al
EPI_ISL_508347	2020-04-24	Institute of Post Graduate Medical Education & Research	National Institute of Biomedical Genomics	Arindam Maitra et al
EPI_ISL_467376	2020-04-24	RSUP Fatmawati	Eijkman Institute for Molecular Biology, Ministry of Research and Technology/National Agency for Research and Innovation	Edison Johar et al
EPI_ISL_457879	2020-04-24	KEMRI-CGMR-C	KEMRI-Wellcome Trust Research Programme/KEMRI-CGMR-C Kilifi	Githinji G. et al 2020 et al
EPI_ISL_527886	2020-04-24	Nigeria Centre for Disease Control (NCDC)	African Centre of Excellence for Genomics of Infectious Diseases (ACEGID), Redeemer's University, Ede, Osun State, Nigeria	Oluniyi P.E. et al et al
EPI_ISL_635272	2020-04-24	Institute of Microbiology and Immunology, Faculty of Medicine, University of Ljubljana	Institute of Microbiology and Immunology, Faculty of Medicine, University of Ljubljana	Tomaž Mark Zorec et al
EPI_ISL_512639	2020-04-24	National Laboratory for Influenza/Virology reference laboratory, Public Health Center of the Ministry of Health of Ukraine	Respiratory Virus Unit, Microbiology Services Colindale, Public Health England	PHE Covid Sequencing Team et al
EPI_ISL_512640	2020-04-24	National Laboratory for Influenza/Virology reference laboratory, Public Health Center of the Ministry of Health of Ukraine	Respiratory Virus Unit, Microbiology Services Colindale, Public Health England	PHE Covid Sequencing Team et al
EPI_ISL_524794	2020-04-25	Evandro Chagas Institute	Evandro Chagas Institute	Santos et al
EPI_ISL_480306	2020-04-25	National Reference Laboratory "Influenza and acute respiratory diseases"	NRL-HIV	Ivan Ivanov et al
EPI_ISL_471411	2020-04-25	Viral Respiratory Lab, National Institute for Biomedical Research (INRB)	Pathogen Sequencing Lab, National Institute for Biomedical Research (INRB)	Placide Mbala-Kingebeni et al
EPI_ISL_471412	2020-04-25	Viral Respiratory Lab, National Institute for Biomedical Research (INRB)	Pathogen Sequencing Lab, National Institute for Biomedical Research (INRB)	Placide Mbala-Kingebeni et al
EPI_ISL_491944	2020-04-25	Instituto Nacional de Investigación en Salud Pública - INSPI	INSPI - Charité	Alfredo Bruno Caicedo et al
EPI_ISL_481483	2020-04-25	Department for Virology, Molecular Biology and Genome Research, R. G. Lugar Center for Public Health Research, National Center for Disease Control and Public Health (NCDC) of Georgia.	Department for Virology, Molecular Biology and Genome Research, R. G. Lugar Center for Public Health Research, National Center for Disease Control and Public Health (NCDC) of Georgia.	Nino Berishvili et al
EPI_ISL_515103	2020-04-25	Department of Biochemistry, Cell and Molecular Biology	WACCBIP, University of Ghana	Ngoi et al
EPI_ISL_475017	2020-04-25	Israel Central Virology laboratory	Israel Central Virology laboratory	Neta Zuckerman et al
EPI_ISL_456388	2020-04-25	LabPLUS	Institute of Environmental Science and Research (ESR)	Matt Storey et al
EPI_ISL_491140	2020-04-25	Oman-National Influenza Center	Biotechnology & OMICs Laboratory	Samiha Al-Kharusi et al
EPI_ISL_486863	2020-04-25	Institut Pasteur Dakar	Institut Pasteur de Dakar	Ndongo Dia et al
EPI_ISL_480431	2020-04-25	Laboratorio de Biología Molecular Asociación Española Primera en Salud	Departments of Pathology and Medicine, New York University School of Medicine	Maria Victoria Elizondo et al
EPI_ISL_458141	2020-04-26	Evandro Chagas Institute	Evandro Chagas Institute	Santos et al
EPI_ISL_541377	2020-04-26	LACEN/SE	Laboratory of Respiratory Viruses and Measles, Oswaldo Cruz Institute, FIOCRUZ	Paola Resende et al
EPI_ISL_541378	2020-04-26	LACEN/SE	Laboratory of Respiratory Viruses and Measles, Oswaldo Cruz Institute, FIOCRUZ	Paola Resende et al
EPI_ISL_541379	2020-04-26	LACEN/SE	Laboratory of Respiratory Viruses and Measles, Oswaldo Cruz Institute, FIOCRUZ	Paola Resende et al
EPI_ISL_541385	2020-04-26	LACEN/SE	Laboratory of Respiratory Viruses and Measles, Oswaldo Cruz Institute, FIOCRUZ	Paola Resende et al
EPI_ISL_480299	2020-04-26	National Reference Laboratory "Influenza and acute respiratory diseases"	NRL-HIV	Ivan Ivanov et al
EPI_ISL_644270	2020-04-26	CEPHR / Vincent's Hospital	Irish Coronavirus Sequencing Consortium - National Virus Reference Laboratory	Michael Carr et al
EPI_ISL_523603	2020-04-26	Dutch COVID-19 response team	Erasmus Medical Center	Bas Oude Munnink et al
EPI_ISL_527885	2020-04-26	Nigeria Centre for Disease Control (NCDC)	African Centre of Excellence for Genomics of Infectious Diseases (ACEGID), Redeemer's University, Ede, Osun State, Nigeria	Oluniyi P.E. et al et al

EPI_ISL_458148	2020-04-27	Evandro Chagas Institute	Evandro Chagas Institute	Santos et al
EPI_ISL_572353	2020-04-27	LACEN/PE	WallauLab, Aggeu Magalhaes Institute	Marcelo Henrique Santos Paiva et al
EPI_ISL_623148	2020-04-27	Laboratorio de Virologia Molecular / UFRJ	Bioinformatics Laboratory / LNCC	Carolina M Voloch et al
EPI_ISL_541380	2020-04-27	LACEN/SE	Laboratory of Respiratory Viruses and Measles, Oswaldo Cruz Institute, FIOCRUZ	Paola Resende et al
EPI_ISL_541381	2020-04-27	LACEN/SE	Laboratory of Respiratory Viruses and Measles, Oswaldo Cruz Institute, FIOCRUZ	Paola Resende et al
EPI_ISL_541382	2020-04-27	LACEN/SE	Laboratory of Respiratory Viruses and Measles, Oswaldo Cruz Institute, FIOCRUZ	Paola Resende et al
EPI_ISL_541383	2020-04-27	LACEN/SE	Laboratory of Respiratory Viruses and Measles, Oswaldo Cruz Institute, FIOCRUZ	Paola Resende et al
EPI_ISL_541384	2020-04-27	LACEN/SE	Laboratory of Respiratory Viruses and Measles, Oswaldo Cruz Institute, FIOCRUZ	Paola Resende et al
EPI_ISL_463748	2020-04-27	Department of Molecular Virology, Cyprus Institute of Neurology and Genetics	Department of Molecular Virology, Cyprus Institute of Neurology and Genetics	Jan Richter et al
EPI_ISL_483156	2020-04-27	Robert Koch Institute, ZBS1 Highly Pathogenic Viruses, Berlin, Germany	Robert Koch Institute, Bioinformatics MF1, Berlin, Germany	Janine Michel et al
EPI_ISL_457891	2020-04-27	KEMRI-CGMR-C	KEMRI-Wellcome Trust Research Programme/KEMRI-CGMR-C Kilifi	Githinji G. et al 2020 et al
EPI_ISL_486421	2020-04-27	Centrālā laboratorija	Latvian Biomedical Research and Study Centre	Ivars Silamiķelis et al
EPI_ISL_496354	2020-04-27	Infectolab	Andersen lab at Scripps Research	SEARCH Alliance San Diego with Samuel Navarro Alvarez et al
EPI_ISL_730001	2020-04-27	Nigeria Centre for Disease Control (NCDC)	African Centre of Excellence for Genomics of Infectious Diseases (ACEGID), Redeemer's University, Ede, Osun State, Nigeria	Oluniyi P.E. et al et al
EPI_ISL_491136	2020-04-27	Oman-National Influenza Center	Biotechnology & OMICs Laboratory	Samiha Al-Kharusi et al
EPI_ISL_468251	2020-04-27	Viollier AG	Department of Biosystems Science and Engineering, ETH Zürich	Christian Beisel et al
EPI_ISL_482386	2020-04-27	Providence St. Joseph Health Molecular Genomics Laboratory	Providence St. Joseph Health Molecular Genomics Laboratory	Alexa K Dowdell et al
EPI_ISL_451197	2020-04-27	Uganda Virus Research Institute	MRC/UVRI & LSHTM Uganda Research Unit	Dan Lule Bugembe et al
EPI_ISL_451198	2020-04-27	Uganda Virus Research Institute	MRC/UVRI & LSHTM Uganda Research Unit	Dan Lule Bugembe et al
EPI_ISL_524790	2020-04-28	Evandro Chagas Institute	Evandro Chagas Institute	Santos et al
EPI_ISL_524783	2020-04-28	Evandro Chagas Institute	Evandro Chagas Institute	Santos et al
EPI_ISL_629012	2020-04-28	Centro de Biotecnología Vegetal, Universidad Andrés Bello, Center for Genome Regulation	Center for Mathematical Modeling and Center for Genome Regulation. Santiago, Chile	Bastias M et al
EPI_ISL_629015	2020-04-28	Centro de Biotecnología Vegetal, Universidad Andrés Bello, Center for Genome Regulation	Center for Mathematical Modeling and Center for Genome Regulation. Santiago, Chile	Bastias M et al
EPI_ISL_629016	2020-04-28	Centro de Biotecnología Vegetal, Universidad Andrés Bello, Center for Genome Regulation	Center for Mathematical Modeling and Center for Genome Regulation. Santiago, Chile	Bastias M et al
EPI_ISL_526956	2020-04-28	Instituto Nacional de Salud, Bogotá, Colombia	Instituto Nacional de Salud, Bogotá, Colombia	Katherine Laiton-Donato et al
EPI_ISL_482760	2020-04-28	Medical Ain Shams Research Institute (MASRI), Ain Shams University	Medical Ain Shams Research Institute (MASRI), Ain Shams University	Hesham Elghazaly et al
EPI_ISL_648335	2020-04-28	Laboratorio de Investigaciones de Baney	University Hospital Basel, Clinical Bacteriology	Carlos Cortes et al
EPI_ISL_644277	2020-04-28	CEPHR / Vincent's Hospital	Irish Coronavirus Sequencing Consortium - National Virus Reference Laboratory	Michael Carr et al
EPI_ISL_450520	2020-04-28	Centrālā Laboratorija	Latvian Biomedical Research and Study Centre	Ivars Silamiķelis et al
EPI_ISL_496348	2020-04-28	Infectolab	Andersen lab at Scripps Research	SEARCH Alliance San Diego with Samuel Navarro Alvarez et al
EPI_ISL_523197	2020-04-28	Dutch COVID-19 response team	Erasmus Medical Center	Bas Oude Munnink et al
EPI_ISL_511103	2020-04-28	Instituto Nacional de Saude (INSA)	Instituto Nacional de Saude (INSA)	Borges et al et al
EPI_ISL_462436	2020-04-28	unknown	Laboratory Diagnostic	Vidanovic et al

EPI_ISL_525476	2020-04-28	Centre for Dengue Research	Centre for Dengue Research	Chandima Jeewandara et al
EPI_ISL_450829	2020-04-28	Narhalsan Sjobo vardcentral	The Public Health Agency of Sweden	Lovisa Hjerten et al
EPI_ISL_605907	2020-04-29	NGS Lab, DNA SOLUTION LTD.	NGS Lab, DNA SOLUTION LTD.	Khan et al
EPI_ISL_605904	2020-04-29	NGS Lab, DNA SOLUTION LTD.	NGS Lab, DNA SOLUTION LTD.	Khan et al
EPI_ISL_605908	2020-04-29	NGS Lab, DNA SOLUTION LTD.	NGS Lab, DNA SOLUTION LTD.	Khan et al
EPI_ISL_605900	2020-04-29	NGS Lab, DNA SOLUTION LTD.	NGS Lab, DNA SOLUTION LTD.	Khan et al
EPI_ISL_524784	2020-04-29	Evandro Chagas Institute	Evandro Chagas Institute	Santos et al
EPI_ISL_524793	2020-04-29	Evandro Chagas Institute	Evandro Chagas Institute	Santos et al
EPI_ISL_524791	2020-04-29	Evandro Chagas Institute	Evandro Chagas Institute	Santos et al
EPI_ISL_623162	2020-04-29	Laboratorio de Virologia Molecular / UFRJ	Bioinformatics Laboratory / LNCC	Carolina M Voloch et al
EPI_ISL_541390	2020-04-29	LACEN/SE	Laboratory of Respiratory Viruses and Measles, Oswaldo Cruz Institute, FIOCRUZ	Paola Resende et al
EPI_ISL_541391	2020-04-29	LACEN/SE	Laboratory of Respiratory Viruses and Measles, Oswaldo Cruz Institute, FIOCRUZ	Paola Resende et al
EPI_ISL_480224	2020-04-29	National Reference Laboratory "Influenza and acute respiratory diseases"	NRL-HIV	Ivan Ivanov et al
EPI_ISL_468755	2020-04-29	Laboratorio de Biologia Molecular, Facultad de Medicina, Universidad de Atacama	Center for Mathematical Modeling and Center for Genome Regulation. Santiago, Chile	Gaete A et al
EPI_ISL_491948	2020-04-29	Instituto Nacional de Investigación en Salud Pública - INSPI	INSPI - Charité	Alfredo Bruno Caicedo et al
EPI_ISL_447056	2020-04-29	Department for Virology, Molecular Biology and Genome Research, R. G. Lugar Center for Public Health Research, National Center for Disease Control and Public Health (NCDC) of Georgia.	Department for Virology, Molecular Biology and Genome Research, R. G. Lugar Center for Public Health Research, National Center for Disease Control and Public Health (NCDC) of Georgia.	Gvantsa Brachveli et al
EPI_ISL_447055	2020-04-29	Department for Virology, Molecular Biology and Genome Research, R. G. Lugar Center for Public Health Research, National Center for Disease Control and Public Health (NCDC) of Georgia.	Department for Virology, Molecular Biology and Genome Research, R. G. Lugar Center for Public Health Research, National Center for Disease Control and Public Health (NCDC) of Georgia.	Meri Pantsulaia et al
EPI_ISL_447047	2020-04-29	GMERS Medical College and Hospital, Gandhinagar	Gujarat Biotechnology Research Centre	Disha Patel et al
EPI_ISL_486420	2020-04-29	Centrālā laboratorija	Latvian Biomedical Research and Study Centre	Ivars Silamiķelis et al
EPI_ISL_496374	2020-04-29	Infectolab	Andersen lab at Scripps Research	SEARCH Alliance San Diego with Samuel Navarro Alvarez et al
EPI_ISL_454215	2020-04-29	unknown	Instituto Nacional de Saude (INSA)	Borges et al et al
EPI_ISL_454216	2020-04-29	unknown	Instituto Nacional de Saude (INSA)	Borges et al et al
EPI_ISL_454217	2020-04-29	unknown	Instituto Nacional de Saude (INSA)	Borges et al et al
EPI_ISL_512975	2020-04-29	Pathogen Genomics Lab King Abdullah University of Science and Technology(KAUST)	Pathogen Genomics Lab King Abdullah University of Science and Technology(KAUST)	Sara Mfarrej et al
EPI_ISL_577738	2020-04-29	Institute of Virology, Biomedical Research Center of the Slovak Academy of Sciences, Bratislava	Faculty of Natural Sciences, Comenius University, Bratislava	Viktória Hodorová et al
EPI_ISL_495419	2020-04-29	Kafkas University, Faculty of Medicine, Department of Medical Microbiology	Kafkas University, Faculty of Medicine, Department of Medical Microbiology	Murat Karamese et al
EPI_ISL_492040	2020-04-30	Instituto de Biologia do Exército	Laboratório Metabolismo Macromolecular FirminoTorres de Castro, Instituto de Biofísica Carlos Chagas Filho, Universidade Federal do Rio de Janeiro	Bianca Catarina Azevedo Cabral et al
EPI_ISL_468747	2020-04-30	Facultad de Medicina UC	Center for Mathematical Modeling and Center for Genome Regulation. Santiago, Chile	Gaete A et al
EPI_ISL_482759	2020-04-30	Medical Ain Shams Research Institute (MASRI), Ain Shams University	Medical Ain Shams Research Institute (MASRI), Ain Shams University	Hesham Elghazaly et al
EPI_ISL_482761	2020-04-30	Medical Ain Shams Research Institute (MASRI), Ain Shams University	Medical Ain Shams Research Institute (MASRI), Ain Shams University	Hesham Elghazaly et al

EPI_ISL_483035	2020-04-30	Medical Ain Shams Research Institute (MASRI), Ain Shams University	Medical Ain Shams Research Institute (MASRI), Ain Shams University	Hesham Elghazaly et al
EPI_ISL_482765	2020-04-30	Medical Ain Shams Research Institute (MASRI), Ain Shams University	Medical Ain Shams Research Institute (MASRI), Ain Shams University	Hesham Elghazaly et al
EPI_ISL_648341	2020-04-30	Laboratorio de Investigaciones de Baney	University Hospital Basel, Clinical Bacteriology	Carlos Cortes et al
EPI_ISL_471161	2020-04-30	MRCG at LSHTM Genomics lab	MRCG at LSHTM Genomics lab	Sesay et al et al
EPI_ISL_471162	2020-04-30	MRCG at LSHTM Genomics lab	MRCG at LSHTM Genomics lab	Sesay et al et al
EPI_ISL_471163	2020-04-30	MRCG at LSHTM Genomics lab	MRCG at LSHTM Genomics lab	Sesay et al et al
EPI_ISL_471164	2020-04-30	MRCG at LSHTM Genomics lab	MRCG at LSHTM Genomics lab	Sesay et al et al
EPI_ISL_471165	2020-04-30	MRCG at LSHTM Genomics lab	MRCG at LSHTM Genomics lab	Sesay et al et al
EPI_ISL_444481	2020-04-30	B.J. Medical College and Civil hospital	Gujarat Biotechnology Research Centre	Kairavi Joshi et al
EPI_ISL_486437	2020-04-30	Centrālā laboratorija	Latvian Biomedical Research and Study Centre	Ivars Silamiķelis et al
EPI_ISL_454220	2020-04-30	unknown	Instituto Nacional de Saude (INSA)	Borges et al et al
EPI_ISL_507236	2020-04-30	WHO National Influenza Centre Russian Federation	WHO National Influenza Centre Russian Federation	Andrey Komissarov et al
EPI_ISL_507246	2020-04-30	WHO National Influenza Centre Russian Federation	WHO National Influenza Centre Russian Federation	Andrey Komissarov et al
EPI_ISL_700512	2020-04-30	Guguletu CHC wc GDH	NHLS/UCT	Arash Iranzadeh et al
EPI_ISL_676525	2020-04-30	Uppsala klinisk mikrobiologi	The Public Health Agency of Sweden	Department of Microbiology et al
EPI_ISL_468284	2020-04-30	Viollier AG	Department of Biosystems Science and Engineering, ETH Zürich	Christian Beisel et al
EPI_ISL_451201	2020-04-30	Uganda Virus Research Institute	MRC/UVRI & LSHTM Uganda Research Unit	Dan Lule Bugembe et al
EPI_ISL_455066	2020-05-01	Pathology West - NSW Health Pathology	NSW Health Pathology - Institute of Clinical Pathology and Medical Research; Westmead Hospital; University of Sydney	CIDM-PH et al. et al
EPI_ISL_524786	2020-05-01	Evandro Chagas Institute	Evandro Chagas Institute	Santos et al
EPI_ISL_722086	2020-05-01	Hospital das Clínicas Universidade de São Paulo Medical School	Laboratório de Parasitologia Médica - Instituto de Medicina Tropical - Universidade de São Paulo	Brazil-UK Centre for Arbovirus Discovery Diagnosis Genomics et al
EPI_ISL_722062	2020-05-01	Hospital das Clínicas Universidade de São Paulo Medical School	Laboratório de Parasitologia Médica - Instituto de Medicina Tropical - Universidade de São Paulo	Brazil-UK Centre for Arbovirus Discovery Diagnosis Genomics et al
EPI_ISL_468758	2020-05-01	Laboratorio de Biologia Molecular, Facultad de Medicina, Universidad de Atacama	Center for Mathematical Modeling and Center for Genome Regulation. Santiago, Chile	Gaete A et al
EPI_ISL_468759	2020-05-01	Laboratorio de Biologia Molecular, Facultad de Medicina, Universidad de Atacama	Center for Mathematical Modeling and Center for Genome Regulation. Santiago, Chile	Gaete A et al
EPI_ISL_629020	2020-05-01	Centro de Biotecnología Vegetal, Universidad Andrés Bello, Center for Genome Regulation	Center for Mathematical Modeling and Center for Genome Regulation. Santiago, Chile	Bastias M et al
EPI_ISL_629018	2020-05-01	Centro de Biotecnología Vegetal, Universidad Andrés Bello, Center for Genome Regulation	Center for Mathematical Modeling and Center for Genome Regulation. Santiago, Chile	Bastias M et al
EPI_ISL_465602	2020-05-01	Respiratory Virus Unit, Microbiology Services Colindale, Public Health England	Respiratory Virus Unit, Microbiology Services Colindale, Public Health England	PHE Covid Sequencing Team et al
EPI_ISL_636477	2020-05-01	CNR Virus des Infections Respiratoires - France SUD	CNR Virus des Infections Respiratoires - France SUD	Antonin Bal et al
EPI_ISL_561016	2020-05-01	MRCG at LSHTM Genomics lab	MRCG at LSHTM Genomics lab	Abdul Karim sesay et al
EPI_ISL_477169	2020-05-01	Department for Virology, Molecular Biology and Genome Research, R. G. Lugar Center for Public Health Research, National Center for Disease Control and Public Health (NCDC) of Georgia.	Department for Virology, Molecular Biology and Genome Research, R. G. Lugar Center for Public Health Research, National Center for Disease Control and Public Health (NCDC) of Georgia.	Tata Imnadze et al
EPI_ISL_481380	2020-05-01	Department for Virology, Molecular Biology and Genome Research, R. G. Lugar Center for Public Health Research, National Center for Disease Control and Public Health (NCDC) of Georgia.	Department for Virology, Molecular Biology and Genome Research, R. G. Lugar Center for Public Health Research, National Center for Disease Control and Public Health (NCDC) of Georgia.	Ana Papkiauri et al
EPI_ISL_644253	2020-05-01	CEPHR / Mater Hospital	Irish Coronavirus Sequencing Consortium - National Virus Reference Laboratory	Michael Carr et al

EPI_ISL_644284	2020-05-01	CEPHR / Vincent's Hospital	Irish Coronavirus Sequencing Consortium - National Virus Reference Laboratory	Michael Carr et al
EPI_ISL_469288	2020-05-01	Keio University Hospital	Keio University Hospital	Kenjiro Kosaki et al
EPI_ISL_637075	2020-05-01	Department of Infectious Diseases and Immunology, National Hospital Organization Nagoya Medical Center	Clinical Research Center, National Hospital Organization Nagoya Medical Center	Yoshihiro Nakata et al
EPI_ISL_579426	2020-05-01	Canterbury Health Laboratories	Institute of Environmental Science and Research (ESR)	Xiaoyun Ren et al
EPI_ISL_454221	2020-05-01	unknown	Instituto Nacional de Saude (INSA)	Borges et al et al
EPI_ISL_648667	2020-05-01	Department of Laboratory Medicine, Tan Tock Seng Hospital	Department of Laboratory Medicine, Tan Tock Seng Hospital	Chen YYC et al
EPI_ISL_468294	2020-05-01	Viollier AG	Department of Biosystems Science and Engineering, ETH Zürich	Christian Beisel et al
EPI_ISL_468298	2020-05-01	Viollier AG	Department of Biosystems Science and Engineering, ETH Zürich	Christian Beisel et al
EPI_ISL_480253	2020-05-01	Genomic Laboratory (GLAB) (Conjoint lab of Health Directorate of Istanbul and Istanbul Technical University)	Genomic Laboratory (GLAB), Istanbul Technical University	Ilker Karacan et al
EPI_ISL_451202	2020-05-01	Uganda Virus Research Institute	MRC/UVRI & LSHTM Uganda Research Unit	Dan Lule Bugembe et al
EPI_ISL_647980	2020-05-01	National Microbiology Reference Laboratory	Quadram Institute Bioscience	Thanh Le Viet et al
EPI_ISL_572366	2020-05-02	LACEN/PE	WallauLab, Aggeu Magalhaes Institute	Marcelo Henrique Santos Paiva et al
EPI_ISL_572367	2020-05-02	LACEN/PE	WallauLab, Aggeu Magalhaes Institute	Marcelo Henrique Santos Paiva et al
EPI_ISL_693196	2020-05-02	Hospital Santa Clara	Instituto Adolfo Lutz, Interdiciplinary Procedures Center, Strategic Laboratory	Claudio Tavares Sacchi et al
EPI_ISL_534316	2020-05-02	OS Mun Santana Lauro Ribas Braga	Instituto Adolfo Lutz, Interdiciplinary Procedures Center, Strategic Laboratory	Claudio Tavares Sacchi et al
EPI_ISL_722040	2020-05-02	Hospital das Clínicas Universidade de São Paulo Medical School	Laboratório de Parasitologia Médica - Instituto de Medicina Tropical - Universidade de São Paulo	Brazil-UK Centre for Arbovirus Discovery Diagnosis Genomics et al
EPI_ISL_629022	2020-05-02	Centro de Biotecnología Vegetal, Universidad Andrés Bello, Center for Genome Regulation	Center for Mathematical Modeling and Center for Genome Regulation. Santiago, Chile	Bastias M et al
EPI_ISL_491941	2020-05-02	Centro de Investigaciones, Universidad de Especialidades Espíritu Santo	Institute of Microbiology, Universidad San Francisco de Quito	Derly Andrade et al
EPI_ISL_468060	2020-05-02	Egyptian National Cancer Institute (ENCI)	Egyptian National Cancer Institute (ENCI)	Zekri et al
EPI_ISL_468056	2020-05-02	Egyptian National Cancer Institute (ENCI)	Egyptian National Cancer Institute (ENCI)	Zekri et al
EPI_ISL_468057	2020-05-02	Egyptian National Cancer Institute (ENCI)	Egyptian National Cancer Institute (ENCI)	Zekri et al
EPI_ISL_644211	2020-05-02	CEPHR / Vincent's Hospital	Irish Coronavirus Sequencing Consortium - National Virus Reference Laboratory	Michael Carr et al
EPI_ISL_528934	2020-05-02	Agenzia di Tutela della Salute di Bergamo	Istitituto Zooprofilattico Sperimentale dell'Abruzzo e Molise "G.Caporale"	Lorusso A et al
EPI_ISL_579403	2020-05-02	LabPLUS	Institute of Environmental Science and Research (ESR)	Xiaoyun Ren et al
EPI_ISL_536502	2020-05-02	Instituto Nacional de Salud	Laboratorio de Infecciones Respiratorias Agudas	Eduardo Juscamayta Lopez et al
EPI_ISL_572375	2020-05-03	LACEN/PE	WallauLab, Aggeu Magalhaes Institute	Marcelo Henrique Santos Paiva et al
EPI_ISL_572358	2020-05-03	LACEN/PE	WallauLab, Aggeu Magalhaes Institute	Marcelo Henrique Santos Paiva et al
EPI_ISL_572359	2020-05-03	LACEN/PE	WallauLab, Aggeu Magalhaes Institute	Marcelo Henrique Santos Paiva et al
EPI_ISL_572360	2020-05-03	LACEN/PE	WallauLab, Aggeu Magalhaes Institute	Marcelo Henrique Santos Paiva et al
EPI_ISL_572361	2020-05-03	LACEN/PE	WallauLab, Aggeu Magalhaes Institute	Marcelo Henrique Santos Paiva et al
EPI_ISL_572362	2020-05-03	LACEN/PE	WallauLab, Aggeu Magalhaes Institute	Marcelo Henrique Santos Paiva et al
EPI_ISL_572363	2020-05-03	LACEN/PE	WallauLab, Aggeu Magalhaes Institute	Marcelo Henrique Santos Paiva et al
EPI_ISL_526950	2020-05-03	Instituto Nacional de Salud, Bogotá, Colombia	Instituto Nacional de Salud, Bogotá, Colombia	Katherine Laiton-Donato et al
EPI_ISL_526933	2020-05-03	Instituto Nacional de Salud, Bogotá, Colombia	Instituto Nacional de Salud, Bogotá, Colombia	Katherine Laiton-Donato et al
EPI_ISL_648343	2020-05-03	Laboratorio de Investigaciones de Baney	University Hospital Basel, Clinical Bacteriology	Carlos Cortes et al

EPI_ISL_501233	2020-05-03	Hellenic Pasteur Institute, National Influenza Reference laboratory of Southern Greece & Unit of Bioinformatics and Applied Genomics	Hellenic Pasteur Institute, National Influenza Reference laboratory of Southern Greece & Unit of Bioinformatics and Applied Genomics	Vasiliki Pogka et al
EPI_ISL_644341	2020-05-03	CEPHR / Vincent's Hospital	Irish Coronavirus Sequencing Consortium - National Virus Reference Laboratory	Michael Carr et al
EPI_ISL_481107	2020-05-03	Hospital General Universitario Gregorio Marañón	SeqCOVID-SPAIN consortium/IBV(CSIC)	Laura Pérez-Lago et al
EPI_ISL_475904	2020-05-04	Zentralinstitut für medizinische und chemische Labordiagnostik, Universitätskliniken Insbruck	Bergthaler laboratory, CeMM Research Center for Molecular Medicine of the Austrian Academy of Sciences	Alexandra Popa et al
EPI_ISL_623119	2020-05-04	Laboratorio de Virologia Molecular / UFRJ	Bioinformatics Laboratory / LNCC	Carolina M Voloch et al
EPI_ISL_623141	2020-05-04	Laboratorio de Virologia Molecular / UFRJ	Bioinformatics Laboratory / LNCC	Carolina M Voloch et al
EPI_ISL_623161	2020-05-04	Laboratorio de Virologia Molecular / UFRJ	Bioinformatics Laboratory / LNCC	Carolina M Voloch et al
EPI_ISL_513574	2020-05-04	Programa de Oncovirologia, Instituto Nacional de Câncer	Programa de Oncovirologia, Instituto Nacional de Câncer	Juliana D. Siqueira et al
EPI_ISL_513575	2020-05-04	Programa de Oncovirologia, Instituto Nacional de Câncer	Programa de Oncovirologia, Instituto Nacional de Câncer	Juliana D. Siqueira et al
EPI_ISL_513576	2020-05-04	Programa de Oncovirologia, Instituto Nacional de Câncer	Programa de Oncovirologia, Instituto Nacional de Câncer	Juliana D. Siqueira et al
EPI_ISL_513578	2020-05-04	Programa de Oncovirologia, Instituto Nacional de Câncer	Programa de Oncovirologia, Instituto Nacional de Câncer	Juliana D. Siqueira et al
EPI_ISL_513579	2020-05-04	Programa de Oncovirologia, Instituto Nacional de Câncer	Programa de Oncovirologia, Instituto Nacional de Câncer	Juliana D. Siqueira et al
EPI_ISL_693198	2020-05-04	Santa Casa de Misericordia de Sao Paulo - Hospital Central	Instituto Adolfo Lutz, Interdiciplinary Procedures Center, Strategic Laboratory	Claudio Tavares Sacchi et al
EPI_ISL_534314	2020-05-04	Hospital Universitario da USP de SP	Instituto Adolfo Lutz, Interdiciplinary Procedures Center, Strategic Laboratory	Claudio Tavares Sacchi et al
EPI_ISL_681683	2020-05-04	Molecular Medicine Laboratory, University of Magallanes	Centro Asistencial Docente y de Investigacion, Universidad de Magallanes	Jorge González et al
EPI_ISL_513616	2020-05-04	Viral Respiratory Lab, National Institute for Biomedical Research (INRB)	Pathogen Sequencing Lab, National Institute for Biomedical Research (INRB)	Placide Mbala-Kingebeni et al
EPI_ISL_622579	2020-05-04	Department of Virus and Microbiological Special Diagnostics, Statens Serum Institut, Denmark	Albertsen lab, Department of Chemistry and Bioscience, Aalborg University, Denmark	Danish Covid-19 Genome Consortia et al
EPI_ISL_622590	2020-05-04	Department of Virus and Microbiological Special Diagnostics, Statens Serum Institut, Denmark	Albertsen lab, Department of Chemistry and Bioscience, Aalborg University, Denmark	Danish Covid-19 Genome Consortia et al
EPI_ISL_614787	2020-05-04	Department of Virus and Microbiological Special Diagnostics, Statens Serum Institut, Denmark	Albertsen lab, Department of Chemistry and Bioscience, Aalborg University, Denmark	Danish Covid-19 Genome Consortia et al
EPI_ISL_481681	2020-05-04	Department of Virology and Immunology, University of Helsinki and Helsinki University Hospital, Huslab Finland	Department of Virology, Faculty of Medicine, University of Helsinki, Helsinki, Finland	Teemu Smura et al
EPI_ISL_660707	2020-05-04	CHU Montpellier	CNR Virus des Infections Respiratoires - France SUD	Antonin Bal et al
EPI_ISL_466906	2020-05-04	Max von Pettenkofer Institute, Virology, National Reference Center for Retroviruses, LMU München	Laboratory for Functional Genome Analysis, Dept. Genomics, Gene Center of the LMU Munich	Max Muenchhoff et al
EPI_ISL_454585	2020-05-04	Laboratory of virology, National Center of Expertise	Laboratory of molecular-genetic research, National Center for Expertise, Kazakhstan National Center for Biotechnology, Kazakhstan	Abdaliyev Askar et al
EPI_ISL_568717	2020-05-04	KEMRI-Wellcome Trust Research Programme/KEMRI-CGMR-C Kilifi	KEMRI-Wellcome Trust Research Programme/KEMRI-CGMR-C Kilifi	Githinji et al 2020 et al
EPI_ISL_625456	2020-05-04	Virology Unit, Institut Pasteur de Madagascar	Virology Unit, Institut Pasteur de Madagascar	Christian Ranaivoson et al
EPI_ISL_493339	2020-05-04	Instituto de Diagnostico y Referencia Epidemiologicos (INDRE)	Instituto de Diagnostico y Referencia Epidemiologicos (INDRE)	Gisela Barrera-Badillo et al
EPI_ISL_481210	2020-05-04	Ostfold Hospital Trust - Kalnes, Centre for Laboratory Medicine, Section for gene technology and infection serology	Norwegian Institute of Public Health, Department of Virology	Kathrine Stene-Johansen et al
EPI_ISL_471176	2020-05-04	Hospital of Southern Norway - Kristiansand, Department of Medical Microbiology	Norwegian Institute of Public Health, Department of Virology	Kathrine Stene-Johansen et al
EPI_ISL_666603	2020-05-04	Dept. of Microbiology and Infection Control, Akershus University Hospital HF	Dept. of Microbiology and Infection Control, Akershus University Hospital HF	Hege Vangstein Aamot et al
EPI_ISL_536508	2020-05-04	Instituto Nacional de Salud	Laboratorio de Infecciones Respiratorias Agudas	Eduardo Juscamayta Lopez et al
EPI_ISL_536515	2020-05-04	Instituto Nacional de Salud	Laboratorio de Infecciones Respiratorias Agudas	Eduardo Juscamayta Lopez et al

EPI_ISL_547941	2020-05-04	Laboratorio de Infecciones Respiratorias Agudas. Centro Nacional de Salud Publica, Instituto Nacional de Salud	Laboratorio de Infecciones Respiratorias Agudas. Centro Nacional de Salud Publica, Instituto Nacional de Salud	Juscamayta et al
EPI_ISL_547948	2020-05-04	Laboratorio de Infecciones Respiratorias Agudas. Centro Nacional de Salud Publica, Instituto Nacional de Salud	Laboratorio de Infecciones Respiratorias Agudas. Centro Nacional de Salud Publica, Instituto Nacional de Salud	Juscamayta et al
EPI_ISL_547954	2020-05-04	Laboratorio de Infecciones Respiratorias Agudas. Centro Nacional de Salud Publica, Instituto Nacional de Salud	Laboratorio de Infecciones Respiratorias Agudas. Centro Nacional de Salud Publica, Instituto Nacional de Salud	Juscamayta et al
EPI_ISL_547957	2020-05-04	Laboratorio de Infecciones Respiratorias Agudas. Centro Nacional de Salud Publica, Instituto Nacional de Salud	Laboratorio de Infecciones Respiratorias Agudas. Centro Nacional de Salud Publica, Instituto Nacional de Salud	Juscamayta et al
EPI_ISL_700508	2020-05-04	Heideveld CDC wc HVP	NHLS/UCT	Arash Iranzadeh et al
EPI_ISL_700491	2020-05-04	Guguletu CHC wc GDH	NHLS/UCT	Arash Iranzadeh et al
EPI_ISL_530074	2020-05-04	Hospital Universitario La Paz	Hospital Universitario La Paz	María Rodríguez et al
EPI_ISL_481105	2020-05-04	Hospital General Universitario Gregorio Marañón	SeqCOVID-SPAIN consortium/IBV(CSIC)	Laura Pérez-Lago et al
EPI_ISL_510822	2020-05-04	Klinisk mikrobiologi centralsjukhuset Karlstad	The Public Health Agency of Sweden	Oskar Karlsson Lindsjo et al
EPI_ISL_480267	2020-05-04	Genomic Laboratory (GLAB) (Conjoint lab of Health Directorate of Istanbul and Istanbul Technical University)	Genomic Laboratory (GLAB), Istanbul Technical University	Ilker Karacan et al
EPI_ISL_610278	2020-05-04	New Mexico Department of Health Scientific Laboratory Division	Center for Global Health, University of New Mexico Health Sciences Center	Daryl Domman et al
EPI_ISL_455058	2020-05-05	Pathology West - NSW Health Pathology	NSW Health Pathology - Institute of Clinical Pathology and Medical Research; Westmead Hospital; University of Sydney	CIDM-PH et al. et al
EPI_ISL_455068	2020-05-05	Childrens Hospital Westmead	NSW Health Pathology - Institute of Clinical Pathology and Medical Research; Westmead Hospital; University of Sydney	CIDM-PH et al. et al
EPI_ISL_467989	2020-05-05	SA Pathology	SA Pathology	Lex Leong et al
EPI_ISL_477125	2020-05-05	Child Health Research Foundation	Child Health Research Foundation	Senjuti Saha et al
EPI_ISL_524789	2020-05-05	Evandro Chagas Institute	Evandro Chagas Institute	Santos et al
EPI_ISL_524785	2020-05-05	Evandro Chagas Institute	Evandro Chagas Institute	Santos et al
EPI_ISL_623151	2020-05-05	Laboratorio de Virologia Molecular / UFRJ	Bioinformatics Laboratory / LNCC	Carolina M Voloch et al
EPI_ISL_623165	2020-05-05	Laboratorio de Virologia Molecular / UFRJ	Bioinformatics Laboratory / LNCC	Carolina M Voloch et al
EPI_ISL_513581	2020-05-05	Programa de Oncovirologia, Instituto Nacional de Câncer	Programa de Oncovirologia, Instituto Nacional de Câncer	Juliana D. Siqueira et al
EPI_ISL_513583	2020-05-05	Programa de Oncovirologia, Instituto Nacional de Câncer	Programa de Oncovirologia, Instituto Nacional de Câncer	Juliana D. Siqueira et al
EPI_ISL_514135	2020-05-05	Rondônia Central Public Health Laboratory (LACEN/RO), vinculated to State Health Secretariat of Rondônia (SESAU/RO)	Molecular Virology Laboratory of Oswaldo Cruz Foundation of Rondônia	Luan Felipo Botelho-Souza et al
EPI_ISL_514136	2020-05-05	Rondônia Central Public Health Laboratory (LACEN/RO), vinculated to State Health Secretariat of Rondônia (SESAU/RO)	Molecular Virology Laboratory of Oswaldo Cruz Foundation of Rondônia	Luan Felipo Botelho-Souza et al
EPI_ISL_514137	2020-05-05	Rondônia Central Public Health Laboratory (LACEN/RO), vinculated to State Health Secretariat of Rondônia (SESAU/RO)	Molecular Virology Laboratory of Oswaldo Cruz Foundation of Rondônia	Luan Felipo Botelho-Souza et al
EPI_ISL_534320	2020-05-05	Hospital do Serv Pub ESTAFCO Morato de Oliveira	Instituto Adolfo Lutz, Interdiciplinary Procedures Center, Strategic Laboratory	Claudio Tavares Sacchi et al
EPI_ISL_534317	2020-05-05	Hospital Geral de Itapevi	Instituto Adolfo Lutz, Interdiciplinary Procedures Center, Strategic Laboratory	Claudio Tavares Sacchi et al
EPI_ISL_722077	2020-05-05	Hospital das Clínicas Universidade de São Paulo Medical School	Laboratório de Parasitologia Médica - Instituto de Medicina Tropical - Universidade de São Paulo	Brazil-UK Centre for Arbovirus Discovery Diagnosis Genomics et al
EPI_ISL_525467	2020-05-05	Universidad Iberoamericana	International Centre for Genetic Engineering and Biotechnology (ICGEB) and ARGO Open Lab Platform	Robert Paulino-Ramirez et al
EPI_ISL_482763	2020-05-05	Medical Ain Shams Research Institute (MASRI), Ain Shams University	Medical Ain Shams Research Institute (MASRI), Ain Shams University	Hesham Elghazaly et al
EPI_ISL_482764	2020-05-05	Medical Ain Shams Research Institute (MASRI), Ain Shams University	Medical Ain Shams Research Institute (MASRI), Ain Shams University	Hesham Elghazaly et al
EPI_ISL_481697	2020-05-05	Department of Virology and Immunology, University of Helsinki and Helsinki University Hospital, Huslab Finland	Department of Virology, Faculty of Medicine, University of Helsinki, Helsinki, Finland	Teemu Smura et al

EPI_ISL_481703	2020-05-05	Department of Virology and Immunology, University of Helsinki and Helsinki University Hospital, Huslab Finland	Department of Virology, Faculty of Medicine, University of Helsinki, Helsinki, Finland	Teemu Smura et al
EPI_ISL_481704	2020-05-05	Department of Virology and Immunology, University of Helsinki and Helsinki University Hospital, Huslab Finland	Department of Virology, Faculty of Medicine, University of Helsinki, Helsinki, Finland	Teemu Smura et al
EPI_ISL_476024	2020-05-05	Laboratoire de Recherche et d'Analyses Médicales de la Gendarmerie Royale	Laboratoire de Recherche et d'Analyses Médicales de la Gendarmerie Royale	Sanaâ Lemriss et al
EPI_ISL_536518	2020-05-05	Instituto Nacional de Salud	Laboratorio de Infecciones Respiratorias Agudas	Eduardo Juscamayta Lopez et al
EPI_ISL_547960	2020-05-05	Laboratorio de Infecciones Respiratorias Agudas. Centro Nacional de Salud Publica, Instituto Nacional de Salud	Laboratorio de Infecciones Respiratorias Agudas. Centro Nacional de Salud Publica, Instituto Nacional de Salud	Juscamayta et al
EPI_ISL_451655	2020-05-05	State Sanitary Inspectorate	Laboratory of Recombinant Vaccines	Lukasz Rabalski et al
EPI_ISL_451658	2020-05-05	State Sanitary Inspectorate	Laboratory of Recombinant Vaccines	Lukasz Rabalski et al
EPI_ISL_451659	2020-05-05	State Sanitary Inspectorate	Laboratory of Recombinant Vaccines	Lukasz Rabalski et al
EPI_ISL_451660	2020-05-05	State Sanitary Inspectorate	Laboratory of Recombinant Vaccines	Lukasz Rabalski et al
EPI_ISL_451662	2020-05-05	State Sanitary Inspectorate	Laboratory of Recombinant Vaccines	Lukasz Rabalski et al
EPI_ISL_511105	2020-05-05	Instituto Nacional de Saude (INSA)	Instituto Nacional de Saude (INSA)	Borges et al et al
EPI_ISL_676585	2020-05-05	Scientific Veterinary Institute Novi Sad	Veterinary Specialized Institute "Kraljevo", Serbia	Vidanovic et al
EPI_ISL_648748	2020-05-05	Department of Laboratory Medicine, Tan Tock Seng Hospital	Department of Laboratory Medicine, Tan Tock Seng Hospital	Chen YYC et al
EPI_ISL_648753	2020-05-05	Department of Laboratory Medicine, Tan Tock Seng Hospital	Department of Laboratory Medicine, Tan Tock Seng Hospital	Chen YYC et al
EPI_ISL_648758	2020-05-05	Department of Laboratory Medicine, Tan Tock Seng Hospital	Department of Laboratory Medicine, Tan Tock Seng Hospital	Chen YYC et al
EPI_ISL_522441	2020-05-05	Center for Laboratory Control of Infectious Diseases, Korea Centers for Diseases Control and Prevention	Center for Laboratory Control of Infectious Diseases, Korea Centers for Diseases Control and Prevention	Junyoung Kim et al
EPI_ISL_578186	2020-05-05	Hospital General Juan Ramón Jiménez	Instituto de Salud Carlos III	Iglesias-Caballero et al
EPI_ISL_541398	2020-05-05	Laboratório de Virologia Comparada e Ambiental- LVCA-IOC	Laboratory of Respiratory Viruses and Measles, Oswaldo Cruz Institute, FIOCRUZ	Paola Resende et al
EPI_ISL_572381	2020-05-06	LACEN/PE	WallauLab, Aggeu Magalhaes Institute	Marcelo Henrique Santos Paiva et al
EPI_ISL_572382	2020-05-06	LACEN/PE	WallauLab, Aggeu Magalhaes Institute	Marcelo Henrique Santos Paiva et al
EPI_ISL_572371	2020-05-06	LACEN/PE	WallauLab, Aggeu Magalhaes Institute	Marcelo Henrique Santos Paiva et al
EPI_ISL_572372	2020-05-06	LACEN/PE	WallauLab, Aggeu Magalhaes Institute	Marcelo Henrique Santos Paiva et al
EPI_ISL_729820	2020-05-06	Laboratorio Central de Saude Publica do Estado do Rio Grande do Sul (LACEN-RS)	Laboratory of Respiratory Viruses and Measles, Oswaldo Cruz Institute, FIOCRUZ	Paola Resende et al
EPI_ISL_729821	2020-05-06	Laboratorio Central de Saude Publica do Estado do Rio Grande do Sul (LACEN-RS)	Laboratory of Respiratory Viruses and Measles, Oswaldo Cruz Institute, FIOCRUZ	Paola Resende et al
EPI_ISL_729797	2020-05-06	Laboratorio Central de Saude Publica do Estado do Rio Grande do Sul (LACEN-RS)	Laboratory of Respiratory Viruses and Measles, Oswaldo Cruz Institute, FIOCRUZ	Paola Resende et al
EPI_ISL_729822	2020-05-06	Laboratorio Central de Saude Publica do Estado do Rio Grande do Sul (LACEN-RS)	Laboratory of Respiratory Viruses and Measles, Oswaldo Cruz Institute, FIOCRUZ	Paola Resende et al
EPI_ISL_534319	2020-05-06	Hospital do Serv Pub ESTAFCO Morato de Oliveira	Instituto Adolfo Lutz, Interdiciplinary Procedures Center, Strategic Laboratory	Claudio Tavares Sacchi et al
EPI_ISL_525468	2020-05-06	Universidad Iberoamericana	International Centre for Genetic Engineering and Biotechnology (ICGEB) and ARGO Open Lab Platform	Robert Paulino-Ramirez et al
EPI_ISL_525469	2020-05-06	Universidad Iberoamericana	International Centre for Genetic Engineering and Biotechnology (ICGEB) and ARGO Open Lab Platform	Robert Paulino-Ramirez et al
EPI_ISL_574611	2020-05-06	RS Premier Bintaro	Eijkman Institute for Molecular Biology, Ministry of Research and Technology/National Agency for Research and Innovation	Frilasita A Yudhaputri et al
EPI_ISL_536521	2020-05-06	Instituto Nacional de Salud	Laboratorio de Infecciones Respiratorias Agudas	Eduardo Juscamayta Lopez et al
EPI_ISL_577739	2020-05-06	Institute of Virology, Biomedical Research Center of the Slovak Academy of Sciences, Bratislava	Faculty of Natural Sciences, Comenius University, Bratislava	Kristína Boršová et al
EPI_ISL_467001	2020-05-06	Viollier AG	Department of Biosystems Science and Engineering, ETH Zürich	Christian Beisel et al
EPI_ISL_463653	2020-05-06	Washington State Department of Health	Seattle Flu Study	Chu et al et al

EPI_ISL_463669	2020-05-06	Washington State Department of Health	Seattle Flu Study	Chu et al et al
EPI_ISL_476561	2020-05-07	Hospital Garrahan	Héritas	Roberta Crespo et al
EPI_ISL_475936	2020-05-07	Universitaetsklinik für Innere Medizin II Innsbruck	Bergthaler laboratory, CeMM Research Center for Molecular Medicine of the Austrian Academy of Sciences	Alexandra Popa et al
EPI_ISL_462275	2020-05-07	KU Leuven, Rega Institute, Clinical and Epidemiological Virology	KU Leuven, Rega Institute, Clinical and Epidemiological Virology	Tony Wawina-Bokalanga et al
EPI_ISL_572379	2020-05-07	LACEN/PE	WallauLab, Aggeu Magalhaes Institute	Marcelo Henrique Santos Paiva et al
EPI_ISL_572380	2020-05-07	LACEN/PE	WallauLab, Aggeu Magalhaes Institute	Marcelo Henrique Santos Paiva et al
EPI_ISL_623158	2020-05-07	Laboratorio de Virologia Molecular / UFRJ	Bioinformatics Laboratory / LNCC	Carolina M Voloch et al
EPI_ISL_480302	2020-05-07	National Reference Laboratory "Influenza and acute respiratory diseases"	NRL-HIV	Ivan Ivanov et al
EPI_ISL_526932	2020-05-07	Instituto Nacional de Salud, Bogotá, Colombia	Instituto Nacional de Salud, Bogotá, Colombia	Katherine Laiton-Donato et al
EPI_ISL_700399	2020-05-07	Centre hospitalier Métropole Savoie	CNR Virus des Infections Respiratoires - France SUD	Antonin Bal et al
EPI_ISL_469291	2020-05-07	Keio University Hospital	Keio University Hospital	Kenjiro Kosaki et al
EPI_ISL_469292	2020-05-07	Keio University Hospital	Keio University Hospital	Kenjiro Kosaki et al
EPI_ISL_486411	2020-05-07	Centrālā laboratorija	Latvian Biomedical Research and Study Centre	Ivars Silamiķelis et al
EPI_ISL_523439	2020-05-07	Dutch COVID-19 response team	Erasmus Medical Center	Bas Oude Munnink et al
EPI_ISL_456838	2020-05-07	West of Scotland Specialist Virology Centre, NHSGGC / MRC-University of Glasgow Centre for Virus Research	COVID-19 Genomics UK (COG-UK) Consortium	Ana da Silva Filipe et al
EPI_ISL_517616	2020-05-07	Academic Hospital Paramaribo	Erasmus Medical Center	Bas Oude Munnink et al
EPI_ISL_469058	2020-05-07	Narhalsan Sjobo vardcentral	The Public Health Agency of Sweden	Oskar Karlsson Lindsjo et al
EPI_ISL_467007	2020-05-07	Viollier AG	Department of Biosystems Science and Engineering, ETH Zürich	Christian Beisel et al
EPI_ISL_480279	2020-05-07	Genomic Laboratory (GLAB) (Conjoint lab of Health Directorate of Istanbul and Istanbul Technical University)	Genomic Laboratory (GLAB), Istanbul Technical University	Ilker Karacan et al
EPI_ISL_480432	2020-05-07	Laboratorio de Biología Molecular Asociación Española Primera en Salud	Departments of Pathology and Medicine, New York University School of Medicine	Maria Victoria Elizondo et al
EPI_ISL_455963	2020-05-08	Department of Clinical Microbiology	GIGA Medical Genomics	Keith Durkin et al
EPI_ISL_524799	2020-05-08	Evandro Chagas Institute	Evandro Chagas Institute	Santos et al
EPI_ISL_510535	2020-05-08	Molecular Virology, Instituto Carlos Chagas / Fiocruz Paraná	Universidade Federal do Parana (UFPR)	Suzukawa et al
EPI_ISL_623149	2020-05-08	Laboratorio de Virologia Molecular / UFRJ	Bioinformatics Laboratory / LNCC	Carolina M Voloch et al
EPI_ISL_729823	2020-05-08	Laboratorio Central de Saude Publica do Estado do Rio Grande do Sul (LACEN-RS)	Laboratory of Respiratory Viruses and Measles, Oswaldo Cruz Institute, FIOCRUZ	Paola Resende et al
EPI_ISL_660450	2020-05-08	Laboratoire de Microbiologie CHU Souro Sanou	Centre Muraz	Abdoul-Salam Ouedraogo et al
EPI_ISL_586333	2020-05-08	Toronto Invasive Bacterial Diseases Network	McMaster University	Allison McGeer et al
EPI_ISL_491947	2020-05-08	Instituto Nacional de Investigación en Salud Pública - INSPI	INSPI - Charité	Alfredo Bruno Caicedo et al
EPI_ISL_491953	2020-05-08	Instituto Nacional de Investigación en Salud Pública - INSPI	INSPI - Charité	Alfredo Bruno Caicedo et al
EPI_ISL_491950	2020-05-08	Instituto Nacional de Investigación en Salud Pública - INSPI	INSPI - Charité	Alfredo Bruno Caicedo et al
EPI_ISL_481722	2020-05-08	Department of Virology and Immunology, University of Helsinki and Helsinki University Hospital, Huslab Finland	Department of Virology, Faculty of Medicine, University of Helsinki, Helsinki, Finland	Teemu Smura et al
EPI_ISL_660740	2020-05-08	Unité des Virus Émergents	CNR Virus des Infections Respiratoires - France SUD	Antonin Bal et al
EPI_ISL_501250	2020-05-08	Hellenic Pasteur Institute, National Influenza Reference laboratory of Southern Greece & Unit of Bioinformatics and Applied Genomics	Hellenic Pasteur Institute, National Influenza Reference laboratory of Southern Greece & Unit of Bioinformatics and Applied Genomics	Vasiliki Pogka et al
EPI_ISL_498270	2020-05-08	Department of Microbiology, The University of Hong Kong	Department of Microbiology, The University of Hong Kong	Kelvin K.W. To et al
EPI_ISL_526236	2020-05-08	Hungarian Defence Forces Military Medical Centre	National Laboratory of Virology, Szentágothai Research Centre	Endre Gábor Tóth et al
EPI_ISL_459924	2020-05-08	Devki Devi Foundation, a unit of Max Healthcare	CSIR-IGIB/Max	Rajesh Pandey# et al

EPI_ISL_528941	2020-05-08	Agenzia di Tutela della Salute di Bergamo	Istituto Zooprofilattico Sperimentale dell'Abruzzo e Molise "G.Caporale"	Lorusso A et al
EPI_ISL_528942	2020-05-08	Agenzia di Tutela della Salute di Bergamo	Istituto Zooprofilattico Sperimentale dell'Abruzzo e Molise "G.Caporale"	Lorusso A et al
EPI_ISL_528945	2020-05-08	Agenzia di Tutela della Salute di Bergamo	Istituto Zooprofilattico Sperimentale dell'Abruzzo e Molise "G.Caporale"	Lorusso A et al
EPI_ISL_528946	2020-05-08	Agenzia di Tutela della Salute di Bergamo	Istituto Zooprofilattico Sperimentale dell'Abruzzo e Molise "G.Caporale"	Lorusso A et al
EPI_ISL_486417	2020-05-08	Centrala laboratorija	Latvian Biomedical Research and Study Centre	Ivars Silamiķelis et al
EPI_ISL_445075	2020-05-08	Laboratoire National de Sante, Microbiology, Virology	Laboratoire National de Sante, Microbiology, Epidemiology and Microbial Genomics	Anke Wienecke-Baldacchino et al
EPI_ISL_482737	2020-05-08	LNR National Reference Laboratory, Mohammed VI University of Health Sciences	Medical Biotechnology Laboratory, Rabat Medical and Pharmacy School, Mohammed The Vth University in Rabat	Meriem LAAMARTI et al
EPI_ISL_468156	2020-05-08	[Romania, Bucharest] National Institute for Infectious Diseases “Prof. Dr. Matei Balș”	[Romania, Bucharest] National Institute for Infectious Diseases “Prof. Dr. Matei Balș”	Leontina Banica et al
EPI_ISL_512636	2020-05-08	National Laboratory for Influenza/Virology reference laboratory, Public Health Center of the Ministry of Health of Ukraine	Respiratory Virus Unit, Microbiology Services Colindale, Public Health England	PHE Covid Sequencing Team et al
EPI_ISL_476565	2020-05-09	Hospital de Pediatría "Prof. Dr. Juan P Garrahan"	Héritas	Andrea Mangano et al
EPI_ISL_455965	2020-05-09	Department of Clinical Microbiology	GIGA Medical Genomics	Keith Durkin et al
EPI_ISL_455966	2020-05-09	Department of Clinical Microbiology	GIGA Medical Genomics	Keith Durkin et al
EPI_ISL_572384	2020-05-09	LACEN/PE	WallauLab, Aggeu Magalhaes Institute	Marcelo Henrique Santos Paiva et al
EPI_ISL_729798	2020-05-09	Laboratorio Central de Saude Publica do Estado do Rio Grande do Sul (LACEN-RS)	Laboratory of Respiratory Viruses and Measles, Oswaldo Cruz Institute, FIOCRUZ	Paola Resende et al
EPI_ISL_454575	2020-05-09	Laboratory of virology, National Center of Expertise	Laboratory of molecular-genetic research, National Center for Expertise, Kazakhstan National Center for Biotechnology, Kazakhstan	Abdaliyev Askar et al
EPI_ISL_568733	2020-05-09	KEMRI-Wellcome Trust Research Programme/KEMRI-CGMR-C Kilifi	KEMRI-Wellcome Trust Research Programme/KEMRI-CGMR-C Kilifi	Githinji et al 2020 et al
EPI_ISL_486418	2020-05-09	Centrālā laboratorija	Latvian Biomedical Research and Study Centre	Ivars Silamiķelis et al
EPI_ISL_579406	2020-05-09	North Shore Hospital	Institute of Environmental Science and Research (ESR)	Xiaoyun Ren et al
EPI_ISL_579407	2020-05-09	North Shore Hospital	Institute of Environmental Science and Research (ESR)	Xiaoyun Ren et al
EPI_ISL_527882	2020-05-09	Nigeria Centre for Disease Control (NCDC)	African Centre of Excellence for Genomics of Infectious Diseases (ACEGID), Redeemer's University, Ede, Osun State, Nigeria	Oluniyi P.E. et al et al
EPI_ISL_572385	2020-05-10	LACEN/PE	WallauLab, Aggeu Magalhaes Institute	Marcelo Henrique Santos Paiva et al
EPI_ISL_572386	2020-05-10	LACEN/PE	WallauLab, Aggeu Magalhaes Institute	Marcelo Henrique Santos Paiva et al
EPI_ISL_729824	2020-05-10	Laboratorio Central de Saude Publica do Estado do Rio Grande do Sul (LACEN-RS)	Laboratory of Respiratory Viruses and Measles, Oswaldo Cruz Institute, FIOCRUZ	Paola Resende et al
EPI_ISL_729825	2020-05-10	Laboratorio Central de Saude Publica do Estado do Rio Grande do Sul (LACEN-RS)	Laboratory of Respiratory Viruses and Measles, Oswaldo Cruz Institute, FIOCRUZ	Paola Resende et al
EPI_ISL_722006	2020-05-10	Hospital das Clínicas Universidade de São Paulo Medical School	Laboratório de Parasitologia Médica - Instituto de Medicina Tropical - Universidade de São Paulo	Brazil-UK Centre for Arbovirus Discovery Diagnosis Genomics et al
EPI_ISL_660451	2020-05-10	Laboratoire de Microbiologie CHU Souro Sanou	Centre Muraz	Abdoul-Salam Ouedraogo et al
EPI_ISL_584073	2020-05-10	The National Institute of Public Health	State Veterinary Institute Prague	Nagy et al
EPI_ISL_568756	2020-05-10	KEMRI-Wellcome Trust Research Programme/KEMRI-CGMR-C Kilifi	KEMRI-Wellcome Trust Research Programme/KEMRI-CGMR-C Kilifi	Githinji et al 2020 et al
EPI_ISL_486413	2020-05-10	Centrālā laboratorija	Latvian Biomedical Research and Study Centre	Ivars Silamiķelis et al
EPI_ISL_718166	2020-05-10	Ministry of Health Hospitals	Institute of Health and Community Medicine	David Perera et al
EPI_ISL_635279	2020-05-10	Institute of Microbiology and Immunology, Faculty of Medicine, University of Ljubljana	Institute of Microbiology and Immunology, Faculty of Medicine, University of Ljubljana	Tomaž Mark Zorec et al
EPI_ISL_475516	2020-05-10	Uppsala Narakut Aleris	The Public Health Agency of Sweden	Oskar Karlsson Lindsjo et al

EPI_ISL_583693	2020-05-11	Center for Virology, Medical University of Vienna	Bergthaler laboratory, CeMM Research Center for Molecular Medicine of the Austrian Academy of Sciences	Alexandra Popa et al
EPI_ISL_447904	2020-05-11	National Institute of Biotechnology	National Institute of Biotechnology	Md. Moniruzzaman et al
EPI_ISL_458133	2020-05-11	National Institute of Biotechnology	Bioinformatics Division, National Institute of Biotechnology	Mohammad Uzzal Hossain et al
EPI_ISL_572388	2020-05-11	LACEN/PE	WallauLab, Aggeu Magalhaes Institute	Marcelo Henrique Santos Paiva et al
EPI_ISL_623143	2020-05-11	Laboratorio de Virologia Molecular / UFRJ	Bioinformatics Laboratory / LNCC	Carolina M Voloch et al
EPI_ISL_514131	2020-05-11	Rondônia Central Public Health Laboratory (LACEN/RO), vinculated to State Health Secretariat of Rondônia (SESAU/RO)	Molecular Virology Laboratory of Oswaldo Cruz Foundation of Rondônia	Luan Felipo Botelho-Souza et al
EPI_ISL_514132	2020-05-11	Rondônia Central Public Health Laboratory (LACEN/RO), vinculated to State Health Secretariat of Rondônia (SESAU/RO)	Molecular Virology Laboratory of Oswaldo Cruz Foundation of Rondônia	Luan Felipo Botelho-Souza et al
EPI_ISL_514133	2020-05-11	Rondônia Central Public Health Laboratory (LACEN/RO), vinculated to State Health Secretariat of Rondônia (SESAU/RO)	Molecular Virology Laboratory of Oswaldo Cruz Foundation of Rondônia	Luan Felipo Botelho-Souza et al
EPI_ISL_514134	2020-05-11	Rondônia Central Public Health Laboratory (LACEN/RO), vinculated to State Health Secretariat of Rondônia (SESAU/RO)	Molecular Virology Laboratory of Oswaldo Cruz Foundation of Rondônia	Luan Felipo Botelho-Souza et al
EPI_ISL_729826	2020-05-11	Laboratorio Central de Saude Publica do Estado do Rio Grande do Sul (LACEN-RS)	Laboratory of Respiratory Viruses and Measles, Oswaldo Cruz Institute, FIOCRUZ	Paola Resende et al
EPI_ISL_729794	2020-05-11	Laboratorio Central de Saude Publica do Estado do Rio Grande do Sul (LACEN-RS)	Laboratory of Respiratory Viruses and Measles, Oswaldo Cruz Institute, FIOCRUZ	Paola Resende et al
EPI_ISL_480308	2020-05-11	National Reference Laboratory "Influenza and acute respiratory diseases"	NRL-HIV	Ivan Ivanov et al
EPI_ISL_526949	2020-05-11	Instituto Nacional de Salud, Bogotá, Colombia	Instituto Nacional de Salud, Bogotá, Colombia	Katherine Laiton-Donato et al
EPI_ISL_501235	2020-05-11	Hellenic Pasteur Institute, National Influenza Reference laboratory of Southern Greece & Unit of Bioinformatics and Applied Genomics	Hellenic Pasteur Institute, National Influenza Reference laboratory of Southern Greece & Unit of Bioinformatics and Applied Genomics	Vasiliki Pogka et al
EPI_ISL_526220	2020-05-11	Hungarian Defence Forces Military Medical Centre	National Laboratory of Virology, Szentágothai Research Centre	Endre Gábor Tóth et al
EPI_ISL_526221	2020-05-11	Hungarian Defence Forces Military Medical Centre	National Laboratory of Virology, Szentágothai Research Centre	Endre Gábor Tóth et al
EPI_ISL_718165	2020-05-11	Ministry of Health Hospitals	Institute of Health and Community Medicine	David Perera et al
EPI_ISL_476026	2020-05-11	Laboratoire de Recherche et d'Analyses Médicales de la Gendarmerie Royale	Laboratoire de Recherche et d'Analyses Médicales de la Gendarmerie Royale	Sanaâ Lemriss et al
EPI_ISL_665258	2020-05-11	Dept. of Microbiology and Infection Control, Akershus University Hospital HF	Dept. of Microbiology and Infection Control, Akershus University Hospital HF	Hege Vangstein Aamot et al
EPI_ISL_548942	2020-05-11	Institute of Microbiology, University of Veterinary and Animal sciences	Institute of Microbiology, University of Veterinary and Animal sciences	Yaqub et al
EPI_ISL_548943	2020-05-11	Institute of Microbiology, University of Veterinary and Animal sciences	Institute of Microbiology, University of Veterinary and Animal sciences	Yaqub et al
EPI_ISL_548944	2020-05-11	Institute of Microbiology, University of Veterinary and Animal sciences	Institute of Microbiology, University of Veterinary and Animal sciences	Yaqub et al
EPI_ISL_548945	2020-05-11	Institute of Microbiology, University of Veterinary and Animal sciences	Institute of Microbiology, University of Veterinary and Animal sciences	Yaqub et al
EPI_ISL_548946	2020-05-11	Institute of Microbiology, University of Veterinary and Animal sciences	Institute of Microbiology, University of Veterinary and Animal sciences	Yaqub et al
EPI_ISL_729827	2020-05-12	Laboratorio Central de Saude Publica do Estado do Rio Grande do Sul (LACEN-RS)	Laboratory of Respiratory Viruses and Measles, Oswaldo Cruz Institute, FIOCRUZ	Paola Resende et al
EPI_ISL_729828	2020-05-12	Laboratorio Central de Saude Publica do Estado do Rio Grande do Sul (LACEN-RS)	Laboratory of Respiratory Viruses and Measles, Oswaldo Cruz Institute, FIOCRUZ	Paola Resende et al
EPI_ISL_722010	2020-05-12	Hospital das Clínicas Universidade de São Paulo Medical School	Laboratório de Parasitologia Médica - Instituto de Medicina Tropical - Universidade de São Paulo	Brazil-UK Centre for Arbovirus Discovery Diagnosis Genomics et al
EPI_ISL_722058	2020-05-12	Hospital das Clínicas Universidade de São Paulo Medical School	Laboratório de Parasitologia Médica - Instituto de Medicina Tropical - Universidade de São Paulo	Brazil-UK Centre for Arbovirus Discovery Diagnosis Genomics et al
EPI_ISL_722011	2020-05-12	Hospital das Clínicas Universidade de São Paulo Medical School	Laboratório de Parasitologia Médica - Instituto de Medicina Tropical - Universidade de São Paulo	Brazil-UK Centre for Arbovirus Discovery Diagnosis Genomics et al

EPI_ISL_722074	2020-05-12	Hospital das Clínicas Universidade de São Paulo Medical School	Laboratório de Parasitologia Médica - Instituto de Medicina Tropical - Universidade de São Paulo	Brazil-UK Centre for Arbovirus Discovery Diagnosis Genomics et al
EPI_ISL_561018	2020-05-12	MRCG at LSHTM Genomics lab	MRCG at LSHTM Genomics lab	Abdul Karim sesay et al
EPI_ISL_561019	2020-05-12	MRCG at LSHTM Genomics lab	MRCG at LSHTM Genomics lab	Abdul Karim sesay et al
EPI_ISL_459916	2020-05-12	Devki Devi Foundation, a unit of Max Healthcare	CSIR-IGIB/Max	Rajesh Pandey# et al
EPI_ISL_459894	2020-05-12	Laboratoire National de Sante, Microbiology, Virology	Laboratoire National de Sante, Microbiology, Epidemiology and Microbial Genomics	Anke Wienecke-Baldacchino et al
EPI_ISL_459905	2020-05-12	Laboratoire National de Sante, Microbiology, Virology	Laboratoire National de Sante, Microbiology, Epidemiology and Microbial Genomics	Anke Wienecke-Baldacchino et al
EPI_ISL_491969	2020-05-12	Oman-NIC	Department of Microbiology and Immunology-SQUH	Fahad Zadjali et al
EPI_ISL_513195	2020-05-12	Pathogen Genomics Lab King Abdullah University of Science and Technology(KAUST)	Pathogen Genomics Lab King Abdullah University of Science and Technology(KAUST)	Afrah Alsomali et al
EPI_ISL_583723	2020-05-13	Center for Virology, Medical University of Vienna	Bergthaler laboratory, CeMM Research Center for Molecular Medicine of the Austrian Academy of Sciences	Alexandra Popa et al
EPI_ISL_477007	2020-05-13	KU Leuven, Rega Institute, Clinical and Epidemiological Virology	KU Leuven, Rega Institute, Clinical and Epidemiological Virology	Tony Wawina-Bokalanga et al
EPI_ISL_524800	2020-05-13	Evandro Chagas Institute	Evandro Chagas Institute	Santos et al
EPI_ISL_729829	2020-05-13	Laboratorio Central de Saude Publica do Estado do Rio Grande do Sul (LACEN-RS)	Laboratory of Respiratory Viruses and Measles, Oswaldo Cruz Institute, FIOCRUZ	Paola Resende et al
EPI_ISL_722069	2020-05-13	Hospital das Clínicas Universidade de São Paulo Medical School	Laboratório de Parasitologia Médica - Instituto de Medicina Tropical - Universidade de São Paulo	Brazil-UK Centre for Arbovirus Discovery Diagnosis Genomics et al
EPI_ISL_480298	2020-05-13	National Reference Laboratory "Influenza and acute respiratory diseases"	NRL-HIV	Ivan Ivanov et al
EPI_ISL_541335	2020-05-13	The National Institute of Public Health	Sídlištní 136/24 165 03, Prague Czech Republic	Nagy et al
EPI_ISL_485812	2020-05-13	Institut für Virologie und Epidemiologie der Viruskrankheiten, Universitätsklinikum Tübingen	NGS Competence Center Tübingen, Institut für Medizinische Mikrobiologie und Hygiene, Universitätsklinikum Tübingen	Angelov et al. et al
EPI_ISL_485813	2020-05-13	Institut für Virologie und Epidemiologie der Viruskrankheiten, Universitätsklinikum Tübingen	NGS Competence Center Tübingen, Institut für Medizinische Mikrobiologie und Hygiene, Universitätsklinikum Tübingen	Angelov et al. et al
EPI_ISL_501236	2020-05-13	Hellenic Pasteur Institute, National Influenza Reference laboratory of Southern Greece & Unit of Bioinformatics and Applied Genomics	Hellenic Pasteur Institute, National Influenza Reference laboratory of Southern Greece & Unit of Bioinformatics and Applied Genomics	Vasiliki Pogka et al
EPI_ISL_671352	2020-05-13	National Virus Reference Laboratory	Irish Coronavirus Sequencing Consortium - Teagasc Moorepark	Calm Walsh et al
EPI_ISL_568790	2020-05-13	KEMRI-Wellcome Trust Research Programme/KEMRI-CGMR-C Kilifi	KEMRI-Wellcome Trust Research Programme/KEMRI-CGMR-C Kilifi	Githinji et al 2020 et al
EPI_ISL_569855	2020-05-13	Omsk Research Institute of Natural Focal Infections	WHO National Influenza Centre Russian Federation	Artem Fadeev et al
EPI_ISL_569822	2020-05-13	Omsk Research Institute of Natural Focal Infections	WHO National Influenza Centre Russian Federation	Artem Fadeev et al
EPI_ISL_644580	2020-05-13	Veterinary Specialized Institute "Kraljevo", Serbia	Veterinary Specialized Institute "Kraljevo", Serbia	Vidanovic et al
EPI_ISL_644581	2020-05-13	Veterinary Specialized Institute "Kraljevo", Serbia	Veterinary Specialized Institute "Kraljevo", Serbia	Vidanovic et al
EPI_ISL_467483	2020-05-13	Molecular Diagnostics Services (MDS)	KRISP, KZN Research Innovation and Sequencing Platform	Giandhari J et al
EPI_ISL_495767	2020-05-13	Washington State Department of Health	Seattle Flu Study	Deborah A. Nickerson et al
EPI_ISL_476568	2020-05-14	Hospital de Pediatría "Prof. Dr. Juan P Garrahan"	Héritas	Cristian Rohr et al
EPI_ISL_572396	2020-05-14	LACEN/PE	WallauLab, Aggeu Magalhaes Institute	Marcelo Henrique Santos Paiva et al
EPI_ISL_623131	2020-05-14	Laboratorio de Virologia Molecular / UFRJ	Bioinformatics Laboratory / LNCC	Carolina M Voloch et al
EPI_ISL_729830	2020-05-14	Laboratorio Central de Saude Publica do Estado do Rio Grande do Sul (LACEN-RS)	Laboratory of Respiratory Viruses and Measles, Oswaldo Cruz Institute, FIOCRUZ	Paola Resende et al
EPI_ISL_729831	2020-05-14	Laboratorio Central de Saude Publica do Estado do Rio Grande do Sul (LACEN-RS)	Laboratory of Respiratory Viruses and Measles, Oswaldo Cruz Institute, FIOCRUZ	Paola Resende et al
EPI_ISL_722017	2020-05-14	Hospital das Clínicas Universidade de São Paulo Medical School	Laboratório de Parasitologia Médica - Instituto de Medicina Tropical - Universidade de São Paulo	Brazil-UK Centre for Arbovirus Discovery Diagnosis Genomics et al
EPI_ISL_577631	2020-05-14	The National Institute of Public Health	State Veterinary Institute Prague	Nagy et al

EPI_ISL_514113	2020-05-14	Viral Respiratory Lab, National Institute for Biomedical Research (INRB)	Pathogen Sequencing Lab, National Institute for Biomedical Research (INRB)	Placide Mbala-Kingebeni et al
EPI_ISL_660351	2020-05-14	CHU de Saint-Étienne Hôpital Nord	CNR Virus des Infections Respiratoires - France SUD	Antonin Bal et al
EPI_ISL_526222	2020-05-14	Hungarian Defence Forces Military Medical Centre	National Laboratory of Virology, Szentágothai Research Centre	Endre Gábor Tóth et al
EPI_ISL_469295	2020-05-14	Keio University Hospital	Keio University Hospital	Kenjiro Kosaki et al
EPI_ISL_454497	2020-05-14	RSE "National Center for Biotechnology"	RSE "National Center for Biotechnology"	Alexandr Shevtsov et al
EPI_ISL_729945	2020-05-14	Nigeria Centre for Disease Control (NCDC)	African Centre of Excellence for Genomics of Infectious Diseases (ACEGID), Redeemer's University, Ede, Osun State, Nigeria	Oluniyi P.E. et al et al
EPI_ISL_730032	2020-05-14	Nigeria Centre for Disease Control (NCDC)	African Centre of Excellence for Genomics of Infectious Diseases (ACEGID), Redeemer's University, Ede, Osun State, Nigeria	Oluniyi P.E. et al et al
EPI_ISL_455471	2020-05-14	Laboratory for Respiratory Viruses, Cantacuzino National Military-Medical Institute for Research and Development	Cantacuzino Institute	M.Lazar et al
EPI_ISL_455475	2020-05-14	Laboratory for Respiratory Viruses, Cantacuzino National Military-Medical Institute for Research and Development	Cantacuzino Institute	M.Lazar et al
EPI_ISL_455474	2020-05-14	Laboratory for Respiratory Viruses, Cantacuzino National Military-Medical Institute for Research and Development	Cantacuzino Institute	M.Lazar et al
EPI_ISL_569818	2020-05-14	Omsk Research Institute of Natural Focal Infections	WHO National Influenza Centre Russian Federation	Artem Fadeev et al
EPI_ISL_498271	2020-05-15	Department of Microbiology, The University of Hong Kong	Department of Microbiology, The University of Hong Kong	Kelvin K.W. To et al
EPI_ISL_568798	2020-05-15	KEMRI-Wellcome Trust Research Programme/KEMRI-CGMR-C Kilifi	KEMRI-Wellcome Trust Research Programme/KEMRI-CGMR-C Kilifi	Githinji et al 2020 et al
EPI_ISL_458150	2020-05-15	ANOUAL	ANOUAL	Jouali Farah et al
EPI_ISL_512810	2020-05-15	National Laboratory for Influenza/Virology reference laboratory, Public Health Center of the Ministry of Health of Ukraine	Respiratory Virus Unit, Microbiology Services Colindale, Public Health England	PHE Covid Sequencing Team et al
EPI_ISL_698310	2020-05-15	Group 42 (G42) Healthcare, Abu Dhabi, United Arab Emirates; Department of Health, The United Arab Emirates	G42 Healthcare	Rong Liu et al
EPI_ISL_476563	2020-05-16	Hospital de Pediatría "Prof. Dr. Juan P Garrahan"	Héritas	Dalmacio Pereyra et al
EPI_ISL_577632	2020-05-16	The National Institute of Public Health	State Veterinary Institute Prague	Nagy et al
EPI_ISL_516806	2020-05-16	Rumah Sakit PKU Gamping	Genetics Working Group (Pokja Genetik) Faculty of Medicine, Public Health and Nursing Universitas Gadjah Mada (FK-KMK UGM); Disease Investigation Center Wates Ministry of Agriculture Indonesia; Department of Microbiology FK-KMK UGM; Laboratorium Diagnostik Yayasan Tahija World Mosquito Program (WMP) Yogyakarta Center for Tropical Medicine FK-KMK UGM; Integrated Research center FK-KMK UGM; Department of Computer Science and Electronics FMIPA UGM	Gunadi et al
EPI_ISL_729944	2020-05-16	Nigeria Centre for Disease Control (NCDC)	African Centre of Excellence for Genomics of Infectious Diseases (ACEGID), Redeemer's University, Ede, Osun State, Nigeria	Oluniyi P.E. et al et al
EPI_ISL_676589	2020-05-16	Scientific Veterinary Institute Novi Sad	Veterinary Specialized Institute "Kraljevo", Serbia	Vidanovic et al
EPI_ISL_512627	2020-05-16	National Laboratory for Influenza/Virology reference laboratory, Public Health Center of the Ministry of Health of Ukraine	Respiratory Virus Unit, Microbiology Services Colindale, Public Health England	PHE Covid Sequencing Team et al
EPI_ISL_480305	2020-05-17	National Reference Laboratory "Influenza and acute respiratory diseases"	NRL-HIV	Ivan Ivanov et al
EPI_ISL_455476	2020-05-17	Laboratory for Respiratory Viruses, Cantacuzino National Military-Medical Institute for Research and Development	Cantacuzino Institute	M.Lazar et al
EPI_ISL_639940	2020-05-17	Omsk Research Institute of Natural Focal Infections	WHO National Influenza Centre Russian Federation	Artem Fadeev et al
EPI_ISL_698384	2020-05-17	Group 42 (G42) Healthcare, Abu Dhabi, United Arab Emirates; Department of Health, The United Arab Emirates	G42 Healthcare	Rong Liu et al
EPI_ISL_476567	2020-05-18	Hospital de Pediatría "Prof. Dr. Juan P Garrahan"	Héritas	Dalmacio Pereyra et al
EPI_ISL_583692	2020-05-18	Center for Virology, Medical University of Vienna	Bergthaler laboratory, CeMM Research Center for Molecular Medicine of the Austrian Academy of Sciences	Alexandra Popa et al

EPI_ISL_672679	2020-05-18	DB Diagnosticos do Brasil	Laboratório de Parasitologia Médica - Instituto de Medicina Tropical - Universidade de São Paulo	Brazil-UK Centre for Arbovirus Discovery Diagnosis Genomics et al
EPI_ISL_729832	2020-05-18	Laboratorio Central de Saude Publica do Estado do Rio Grande do Sul (LACEN-RS)	Laboratory of Respiratory Viruses and Measles, Oswaldo Cruz Institute, FIOCRUZ	Paola Resende et al
EPI_ISL_527524	2020-05-18	Viral Respiratory Lab, National Institute for Biomedical Research (INRB)	Pathogen Sequencing Lab, National Institute for Biomedical Research (INRB)	Placide Mbala-Kingebeni et al
EPI_ISL_614856	2020-05-18	Department of Virus and Microbiological Special Diagnostics, Statens Serum Institut, Denmark	Albertsen lab, Department of Chemistry and Bioscience, Aalborg University, Denmark	Danish Covid-19 Genome Consortia et al
EPI_ISL_648351	2020-05-18	Laboratorio de Investigaciones de Baney	University Hospital Basel, Clinical Bacteriology	Carlos Cortes et al
EPI_ISL_596452	2020-05-18	Department of Pathology, School of Medicine, Imam Khomeini Hospital, Tehran University of Medical Sciences	Genetics Research Center, University of Social Welfare and Rehabilitation Sciences	Zohreh Fattahi et al
EPI_ISL_560392	2020-05-18	Vilnius University Hospital Santaros Klinikos, Vilnius University	Institute of Biotechnology, Life Sciences Center, Vilnius University and Thermo Fisher Scientific	Justinas Slikas et al
EPI_ISL_560401	2020-05-18	Vilnius University Hospital Santaros Klinikos, Vilnius University	Institute of Biotechnology, Life Sciences Center, Vilnius University and Thermo Fisher Scientific	Justinas Slikas et al
EPI_ISL_560402	2020-05-18	Vilnius University Hospital Santaros Klinikos, Vilnius University	Institute of Biotechnology, Life Sciences Center, Vilnius University and Thermo Fisher Scientific	Justinas Slikas et al
EPI_ISL_459895	2020-05-18	Laboratoire National de Sante, Microbiology, Virology	Laboratoire National de Sante, Microbiology, Epidemiology and Microbial Genomics	Anke Wienecke-Baldacchino et al
EPI_ISL_572398	2020-05-18	Laboratory of Biology and Identification of Arboviruses	Pathogenic Microorganisms Variability Laboratory	Alexey Shchetinin et al
EPI_ISL_495427	2020-05-18	Kafkas University, Faculty of Medicine, Department of Medical Microbiology	Kafkas University, Faculty of Medicine, Department of Medical Microbiology	Murat Karamese et al
EPI_ISL_698381	2020-05-18	Group 42 (G42) Healthcare, Abu Dhabi, United Arab Emirates; Department of Health, The United Arab Emirates	G42 Healthcare	Rong Liu et al
EPI_ISL_476571	2020-05-19	Hospital de Pediatría "Prof. Dr. Juan P Garrahan"	Héritas	Dalmacio Pereyra et al
EPI_ISL_729833	2020-05-19	Laboratorio Central de Saude Publica do Estado do Rio Grande do Sul (LACEN-RS)	Laboratory of Respiratory Viruses and Measles, Oswaldo Cruz Institute, FIOCRUZ	Paola Resende et al
EPI_ISL_471271	2020-05-19	Hospital Oncológico Solca Núcleo de Quito	Institute of Microbiology, Universidad San Francisco de Quito	Sully Márquez et al
EPI_ISL_471270	2020-05-19	Hospital Oncológico Solca Núcleo de Quito	Institute of Microbiology, Universidad San Francisco de Quito	Sully Márquez et al
EPI_ISL_499996	2020-05-19	Liverpool Clinical Laboratories	COVID-19 Genomics UK (COG-UK) Consortium	Sam Haldenby et al
EPI_ISL_648352	2020-05-19	Laboratorio de Investigaciones de Baney	University Hospital Basel, Clinical Bacteriology	Carlos Cortes et al
EPI_ISL_728298	2020-05-19	Jena University Hospital, Institute for Infectious Diseases and Infection Control	Institute of infectious medicine & hospital hygiene, CaSe-Group	Spott et al
EPI_ISL_459902	2020-05-19	Laboratoire National de Sante, Microbiology, Virology	Laboratoire National de Sante, Microbiology, Epidemiology and Microbial Genomics	Anke Wienecke-Baldacchino et al
EPI_ISL_718156	2020-05-19	Ministry of Health Hospitals	Institute of Health and Community Medicine	David Perera et al
EPI_ISL_635486	2020-05-19	Centro de Diagn√≥stico COVID-19 UABC Tijuana	Andersen lab at Scripps Research	SEARCH Alliance San Diego with Idanya Rubí Serafín Higuera et al
EPI_ISL_491985	2020-05-19	Oman-NIC	Department of Microbiology and Immunology-SQUH	Fahad Zadjali et al
EPI_ISL_491986	2020-05-19	Oman-NIC	Department of Microbiology and Immunology-SQUH	Fahad Zadjali et al
EPI_ISL_700499	2020-05-19	Heideveld CDC wc HVP	NHLS/UCT	Arash Iranzadeh et al
EPI_ISL_698272	2020-05-19	Group 42 (G42) Healthcare, Abu Dhabi, United Arab Emirates; Department of Health, The United Arab Emirates	G42 Healthcare	Rong Liu et al
EPI_ISL_698960	2020-05-19	Group 42 (G42) Healthcare, Abu Dhabi, United Arab Emirates; Department of Health, The United Arab Emirates	G42 Healthcare	Rong Liu et al
EPI_ISL_480434	2020-05-19	Laboratorio de Biología Molecular Asociación Española Primera en Salud	Departments of Pathology and Medicine, New York University School of Medicine	Maria Victoria Elizondo et al
EPI_ISL_480435	2020-05-19	Laboratorio de Biología Molecular Asociación Española Primera en Salud	Departments of Pathology and Medicine, New York University School of Medicine	Maria Victoria Elizondo et al
EPI_ISL_729835	2020-05-20	Laboratorio Central de Saude Publica do Estado do Rio Grande do Sul (LACEN-RS)	Laboratory of Respiratory Viruses and Measles, Oswaldo Cruz Institute, FIOCRUZ	Paola Resende et al

EPI_ISL_693199	2020-05-20	Hospital do Servidor Publico Estadual Francisco Morato de Oliveira	Instituto Adolfo Lutz, Interdiciplinary Procedures Center, Strategic Laboratory	Claudio Tavares Sacchi et al
EPI_ISL_526267	2020-05-20	Unity Health Toronto	Ontario Institute for Cancer Research	Ramzi Fattouh et al
EPI_ISL_629013	2020-05-20	Centro de Biotecnología Vegetal, Universidad Andrés Bello, Center for Genome Regulation	Center for Mathematical Modeling and Center for Genome Regulation. Santiago, Chile	Bastias M et al
EPI_ISL_527547	2020-05-20	Viral Respiratory Lab, National Institute for Biomedical Research (INRB)	Pathogen Sequencing Lab, National Institute for Biomedical Research (INRB)	Placide Mbala-Kingebeni et al
EPI_ISL_527532	2020-05-20	Viral Respiratory Lab, National Institute for Biomedical Research (INRB)	Pathogen Sequencing Lab, National Institute for Biomedical Research (INRB)	Placide Mbala-Kingebeni et al
EPI_ISL_559600	2020-05-20	Lighthouse Lab in Alderley Park	Wellcome Sanger Institute for the COVID-19 Genomics UK (COG-UK) consortium	The Lighthouse Lab in Alderley Park et al
EPI_ISL_508696	2020-05-20	Institut für Virologie und Epidemiologie der Viruskrankheiten, Universitätsklinikum Tübingen	NGS Competence Center Tübingen, Institut für Medizinische Mikrobiologie und Hygiene, Universitätsklinikum Tübingen	Angel Angelov et al
EPI_ISL_515099	2020-05-20	Department of Biochemistry, Cell and Molecular Biology	WACCBIP, University of Ghana	Ngoi et al
EPI_ISL_677674	2020-05-20	General Hospital - Prilep	Research Center for Genetic Engineering and Biotechnology "Georgi D. Efremov" , Macedonian Academy of Sciences and Arts	RCGEB - MASA et al
EPI_ISL_510627	2020-05-20	Division of Viral Diseases, Center for Laboratory Control of Infectious Diseases, Korea Centers for Diseases Control and Prevention	Division of Viral Diseases, Center for Laboratory Control of Infectious Diseases, Korea Centers for Diseases Control and Prevention	Jeong-Min Kim et al
EPI_ISL_560397	2020-05-21	Vilnius University Hospital Santaros Klinikos, Vilnius University	Institute of Biotechnology, Life Sciences Center, Vilnius University and Thermo Fisher Scientific	Justinas Slikas et al
EPI_ISL_491989	2020-05-21	Oman-NIC	Department of Microbiology and Immunology-SQUH	Fahad Zadjali et al
EPI_ISL_525426	2020-05-21	Oman-National Influenza Center	Biotechnology & OMICs Laboratory	Sajjad Asaf et al
EPI_ISL_530091	2020-05-21	Hospital Universitario La Paz	Hospital Universitario La Paz	María Rodríguez et al
EPI_ISL_495440	2020-05-21	Kafkas University, Faculty of Medicine, Department of Medical Microbiology	Kafkas University, Faculty of Medicine, Department of Medical Microbiology	Murat Karamese et al
EPI_ISL_476573	2020-05-22	Hospital de Pediatría "Prof. Dr. Juan P Garrahan"	Héritas	Dalmacio Pereyra et al
EPI_ISL_569988	2020-05-22	Unity Health Toronto	Ontario Institute for Cancer Research	Ramzi Fattouh et al
EPI_ISL_649155	2020-05-22	Laboratorio de Investigaciones de Baney	University Hospital Basel, Clinical Bacteriology	Carlos Cortes et al
EPI_ISL_561028	2020-05-22	MRCG at LSHTM Genomics lab	MRCG at LSHTM Genomics lab	Abdul Karim sesay et al
EPI_ISL_515101	2020-05-22	Department of Biochemistry, Cell and Molecular Biology	WACCBIP, University of Ghana	Ngoi et al
EPI_ISL_560394	2020-05-22	Vilnius University Hospital Santaros Klinikos, Vilnius University	Institute of Biotechnology, Life Sciences Center, Vilnius University and Thermo Fisher Scientific	Justinas Slikas et al
EPI_ISL_523694	2020-05-22	Dutch COVID-19 response team	Erasmus Medical Center	Bas Oude Munnink et al
EPI_ISL_635116	2020-05-22	Ostfold Hospital Trust - Kalnes, Centre for Laboratory Medicine, Section for gene technology and infection serology	Norwegian Institute of Public Health, Department of Virology	Kathrine Stene-Johansen et al
EPI_ISL_510636	2020-05-22	Division of Viral Diseases, Center for Laboratory Control of Infectious Diseases, Korea Centers for Diseases Control and Prevention	Division of Viral Diseases, Center for Laboratory Control of Infectious Diseases, Korea Centers for Diseases Control and Prevention	Jeong-Min Kim et al
EPI_ISL_462093	2020-05-23	National Institute of Laboratory Medicine and Referral Center	Genomic Research Lab, BCSIR	Abu Sayeed Mohammad Mahmud et al
EPI_ISL_469299	2020-05-23	National Institute of Laboratory Medicine and Referral Center	Genomic Research Lab, BCSIR	Iffat Jahan et al
EPI_ISL_515091	2020-05-23	Department of Biochemistry, Cell and Molecular Biology	WACCBIP, University of Ghana	Ngoi et al
EPI_ISL_481128	2020-05-23	Immunogenomics lab, Institute of Life Sciences, Bhubaneswar	Immunogenomics lab, Institute of Life Sciences, Bhubaneswar	Sunil Raghav et al
EPI_ISL_493340	2020-05-23	Instituto de Diagnostico y Referencia Epidemiologicos (INDRE)	Instituto de Diagnostico y Referencia Epidemiologicos (INDRE)	Gisela Barrera-Badillo et al
EPI_ISL_579425	2020-05-23	LabPLUS	Institute of Environmental Science and Research (ESR)	Xiaoyun Ren et al
EPI_ISL_541332	2020-05-24	The National Institute of Public Health	State Veterinary Institute Prague	Nagy et al
EPI_ISL_515092	2020-05-24	Department of Biochemistry, Cell and Molecular Biology	WACCBIP, University of Ghana	Ngoi et al
EPI_ISL_523443	2020-05-24	Dutch COVID-19 response team	Erasmus Medical Center	Bas Oude Munnink et al

EPI_ISL_523444	2020-05-24	Dutch COVID-19 response team	Erasmus Medical Center	Bas Oude Munnink et al
EPI_ISL_513012	2020-05-24	Pathogen Genomics Lab King Abdullah University of Science and Technology(KAUST)	Pathogen Genomics Lab King Abdullah University of Science and Technology(KAUST)	Afrah Alsomali et al
EPI_ISL_513021	2020-05-24	Pathogen Genomics Lab King Abdullah University of Science and Technology(KAUST)	Pathogen Genomics Lab King Abdullah University of Science and Technology(KAUST)	Afrah Alsomali et al
EPI_ISL_513029	2020-05-24	Pathogen Genomics Lab King Abdullah University of Science and Technology(KAUST)	Pathogen Genomics Lab King Abdullah University of Science and Technology(KAUST)	Rahul P Salunke et al
EPI_ISL_513035	2020-05-24	Pathogen Genomics Lab King Abdullah University of Science and Technology(KAUST)	Pathogen Genomics Lab King Abdullah University of Science and Technology(KAUST)	Rahul P Salunke et al
EPI_ISL_569954	2020-05-25	Unity Health Toronto	Ontario Institute for Cancer Research	Ramzi Fattouh et al
EPI_ISL_614841	2020-05-25	Department of Virus and Microbiological Special Diagnostics, Statens Serum Institut, Denmark	Albertsen lab, Department of Chemistry and Bioscience, Aalborg University, Denmark	Danish Covid-19 Genome Consortia et al
EPI_ISL_559387	2020-05-25	Lighthouse Lab in Alderley Park	Wellcome Sanger Institute for the COVID-19 Genomics UK (COG-UK) consortium	The Lighthouse Lab in Alderley Park et al
EPI_ISL_526223	2020-05-25	Hungarian Defence Forces Military Medical Centre	National Laboratory of Virology, Szentágothai Research Centre	Endre Gábor Tóth et al
EPI_ISL_491189	2020-05-25	Instituto Gulbenkian de Ciência	Instituto Gulbenkian de Ciência	João Costa et al
EPI_ISL_660417	2020-05-25	Klinisk mikrobiologi	The Public Health Agency of Sweden	Anna-Malin Linde et al
EPI_ISL_480338	2020-05-25	Microbial Genomics Laboratory, Institut Pasteur de Montevideo	Microbial Genomics Laboratory, Institut Pasteur de Montevideo	Cecilia Salazar et al
EPI_ISL_480339	2020-05-25	Microbial Genomics Laboratory, Institut Pasteur de Montevideo	Microbial Genomics Laboratory, Institut Pasteur de Montevideo	Cecilia Salazar et al
EPI_ISL_480340	2020-05-25	Microbial Genomics Laboratory, Institut Pasteur de Montevideo	Microbial Genomics Laboratory, Institut Pasteur de Montevideo	Cecilia Salazar et al
EPI_ISL_480341	2020-05-25	Microbial Genomics Laboratory, Institut Pasteur de Montevideo	Microbial Genomics Laboratory, Institut Pasteur de Montevideo	Cecilia Salazar et al
EPI_ISL_480346	2020-05-25	Microbial Genomics Laboratory, Institut Pasteur de Montevideo	Microbial Genomics Laboratory, Institut Pasteur de Montevideo	Cecilia Salazar et al
EPI_ISL_615121	2020-05-26	Hospital de Pediatría "Prof. Dr. Juan P Garrahan"	Héritas	Cristian Rohr et al
EPI_ISL_591523	2020-05-26	Medicina Norte U Chile - Servicio Medico Legal	Center for Mathematical Modeling and Center for Genome Regulation. Santiago, Chile	Gaggero A et al
EPI_ISL_591524	2020-05-26	Medicina Norte U Chile - Servicio Medico Legal	Center for Mathematical Modeling and Center for Genome Regulation. Santiago, Chile	Gaggero A et al
EPI_ISL_491949	2020-05-26	Instituto Nacional de Investigación en Salud Pública - INSPI	INSPI - Charité	Alfredo Bruno Caicedo et al
EPI_ISL_697796	2020-05-26	Universidad Regional Amazonica IKIAM	Institute of Microbiology, Universidad San Francisco de Quito	Fabian Aguilar et al
EPI_ISL_479547	2020-05-26	NIV Influenza	NIV Influenza	Potdar V et al
EPI_ISL_568690	2020-05-26	RSUP Fatmawati	Eijkman Institute for Molecular Biology, Ministry of Research and Technology/National Agency for Research and Innovation	Frilasita A Yudhaputri et al
EPI_ISL_482740	2020-05-26	LNR National Reference Laboratory, Mohammed VI University of Health Sciences	Medical Biotechnology Laboratory, Rabat Medical and Pharmacy School, Mohammed The Vth University in Rabat	Meriem LAAMARTI et al
EPI_ISL_523165	2020-05-26	Dutch COVID-19 response team	Erasmus Medical Center	Bas Oude Munnink et al
EPI_ISL_480436	2020-05-26	Laboratorio de Biología Molecular Asociación Española Primera en Salud	Departments of Pathology and Medicine, New York University School of Medicine	Maria Victoria Elizondo et al
EPI_ISL_480437	2020-05-26	Laboratorio de Biología Molecular Asociación Española Primera en Salud	Departments of Pathology and Medicine, New York University School of Medicine	Maria Victoria Elizondo et al
EPI_ISL_462753	2020-05-27	University Clinical Hospital of Mostar	University of Sarajevo Veterinary Faculty	Goletic et al
EPI_ISL_729836	2020-05-27	Laboratorio Central de Saude Publica do Estado do Rio Grande do Sul (LACEN-RS)	Laboratory of Respiratory Viruses and Measles, Oswaldo Cruz Institute, FIOCRUZ	Paola Resende et al
EPI_ISL_729795	2020-05-27	Laboratorio Central de Saude Publica do Estado do Rio Grande do Sul (LACEN-RS)	Laboratory of Respiratory Viruses and Measles, Oswaldo Cruz Institute, FIOCRUZ	Paola Resende et al
EPI_ISL_729796	2020-05-27	Laboratorio Central de Saude Publica do Estado do Rio Grande do Sul (LACEN-RS)	Laboratory of Respiratory Viruses and Measles, Oswaldo Cruz Institute, FIOCRUZ	Paola Resende et al
EPI_ISL_586352	2020-05-27	Toronto Invasive Bacterial Diseases Network	McMaster University	Allison McGeer et al
EPI_ISL_648307	2020-05-27	Laboratorio de Investigaciones de Baney	University Hospital Basel, Clinical Bacteriology	Carlos Cortes et al
EPI_ISL_515097	2020-05-27	Department of Biochemistry, Cell and Molecular Biology	WACCBIP, University of Ghana	Ngoi et al

EPI_ISL_568691	2020-05-27	RSUP Fatmawati	Eijkman Institute for Molecular Biology, Ministry of Research and Technology/National Agency for Research and Innovation	Frilasita A Yudhaputri et al
EPI_ISL_498238	2020-05-27	Institut Pasteur de Dakar	Institut Pasteur de Dakar	Ndongo Dia et al
EPI_ISL_498239	2020-05-27	Institut Pasteur de Dakar	Institut Pasteur de Dakar	Ndongo Dia et al
EPI_ISL_479594	2020-05-27	National Public Health Laboratory, National Centre for Infectious Diseases	National Public Health Laboratory, National Centre for Infectious Diseases	Mak TM et al
EPI_ISL_522448	2020-05-27	Center for Laboratory Control of Infectious Diseases, Korea Centers for Diseases Control and Prevention	Center for Laboratory Control of Infectious Diseases, Korea Centers for Diseases Control and Prevention	Junyoung Kim et al
EPI_ISL_476094	2020-05-27	Viollier AG	Department of Biosystems Science and Engineering, ETH Zürich	Christian Beisel et al
EPI_ISL_512604	2020-05-27	National Laboratory for Influenza/Virology reference laboratory, Public Health Center of the Ministry of Health of Ukraine	Respiratory Virus Unit, Microbiology Services Colindale, Public Health England	PHE Covid Sequencing Team et al
EPI_ISL_729837	2020-05-28	Laboratorio Central de Saude Publica do Estado do Rio Grande do Sul (LACEN-RS)	Laboratory of Respiratory Viruses and Measles, Oswaldo Cruz Institute, FIOCRUZ	Paola Resende et al
EPI_ISL_729800	2020-05-28	Laboratorio Central de Saude Publica do Estado do Rio Grande do Sul (LACEN-RS)	Laboratory of Respiratory Viruses and Measles, Oswaldo Cruz Institute, FIOCRUZ	Paola Resende et al
EPI_ISL_729840	2020-05-28	Laboratorio Central de Saude Publica do Estado do Rio Grande do Sul (LACEN-RS)	Laboratory of Respiratory Viruses and Measles, Oswaldo Cruz Institute, FIOCRUZ	Paola Resende et al
EPI_ISL_491954	2020-05-28	Instituto Nacional de Investigación en Salud Pública - INSPI	INSPI - Charité	Alfredo Bruno Caicedo et al
EPI_ISL_491946	2020-05-28	Instituto Nacional de Investigación en Salud Pública - INSPI	INSPI - Charité	Alfredo Bruno Caicedo et al
EPI_ISL_561032	2020-05-28	MRCG at LSHTM Genomics lab	MRCG at LSHTM Genomics lab	Abdul Karim sesay et al
EPI_ISL_482661	2020-05-28	National Centre for Disease control (NCDC)	NCDC/CSIR-IGIB	Pramod Kumar# et al
EPI_ISL_486438	2020-05-28	E. Gulbja laboratorija	Latvian Biomedical Research and Study Centre	Ivars Silamiķelis et al
EPI_ISL_693550	2020-05-28	Instituto Nacional de Saude (INSA)	Instituto Nacional de Saude (INSA)	Borges et al et al
EPI_ISL_676590	2020-05-28	Scientific Veterinary Institute Novi Sad	Veterinary Specialized Institute "Kraljevo", Serbia	Vidanovic et al
EPI_ISL_510815	2020-05-28	NA	The Public Health Agency of Sweden	Oskar Karlsson Lindsjo et al
EPI_ISL_512634	2020-05-28	National Laboratory for Influenza/Virology reference laboratory, Public Health Center of the Ministry of Health of Ukraine	Respiratory Virus Unit, Microbiology Services Colindale, Public Health England	PHE Covid Sequencing Team et al
EPI_ISL_636835	2020-05-29	Laboratório de Imunofarmacologia - Instituto Oswaldo Cruz	Laboratório de Imunofarmacologia - Instituto Oswaldo Cruz	Souza et al
EPI_ISL_636837	2020-05-29	Laboratório de Imunofarmacologia - Instituto Oswaldo Cruz	Laboratório de Imunofarmacologia - Instituto Oswaldo Cruz	Souza et al
EPI_ISL_729846	2020-05-29	Laboratorio Central de Saude Publica do Estado do Rio Grande do Sul (LACEN-RS)	Laboratory of Respiratory Viruses and Measles, Oswaldo Cruz Institute, FIOCRUZ	Paola Resende et al
EPI_ISL_729838	2020-05-29	Laboratorio Central de Saude Publica do Estado do Rio Grande do Sul (LACEN-RS)	Laboratory of Respiratory Viruses and Measles, Oswaldo Cruz Institute, FIOCRUZ	Paola Resende et al
EPI_ISL_729839	2020-05-29	Laboratorio Central de Saude Publica do Estado do Rio Grande do Sul (LACEN-RS)	Laboratory of Respiratory Viruses and Measles, Oswaldo Cruz Institute, FIOCRUZ	Paola Resende et al
EPI_ISL_591522	2020-05-29	Medicina Norte U Chile - Servicio Medico Legal	Center for Mathematical Modeling and Center for Genome Regulation. Santiago, Chile	Gaggero A et al
EPI_ISL_614349	2020-05-29	Molecular diagnostic unit for viral haemorrhagic fevers and emerging viruses, Bouaké CHU Laboratory	Project group Epidemology of Highly Pathogenic Microorganisms, Robert Koch-Institute	Chantal Akoua-Koffi et al
EPI_ISL_470876	2020-05-29	Department for Virology, Molecular Biology and Genome Research, R. G. Lugar Center for Public Health Research, National Center for Disease Control and Public Health (NCDC) of Georgia.	Department for Virology, Molecular Biology and Genome Research, R. G. Lugar Center for Public Health Research, National Center for Disease Control and Public Health (NCDC) of Georgia.	Giorgi Tomashvili et al
EPI_ISL_718135	2020-05-29	Ministry of Health Hospitals	Institute of Health and Community Medicine	David Perera et al
EPI_ISL_718134	2020-05-29	Ministry of Health Hospitals	Institute of Health and Community Medicine	David Perera et al
EPI_ISL_635503	2020-05-29	Centro de Diagn√≥stico COVID-19 UABC Tijuana	Andersen lab at Scripps Research	SEARCH Alliance San Diego with Idanya Rubí Serafín Higuera et al
EPI_ISL_493345	2020-05-29	Instituto de Diagnostico y Referencia Epidemiologicos (INDRE)	Instituto de Diagnostico y Referencia Epidemiologicos (INDRE)	Ernesto Ramirez-Gonzalez et al
EPI_ISL_522451	2020-05-29	Center for Laboratory Control of Infectious Diseases, Korea Centers for Diseases Control and Prevention	Center for Laboratory Control of Infectious Diseases, Korea Centers for Diseases Control and Prevention	Junyoung Kim et al

EPI_ISL_496075	2020-05-29	Washington State Department of Health	Seattle Flu Study	Deborah A. Nickerson et al
EPI_ISL_693232	2020-05-30	Hospital e Pronto Socorro Portinari	Instituto Adolfo Lutz, Interdiciplinary Procedures Center, Strategic Laboratory	Claudio Tavares Sacchi et al
EPI_ISL_614348	2020-05-30	Molecular diagnostic unit for viral haemorrhagic fevers and emerging viruses, Bouaké CHU Laboratory	Project group Epidemology of Highly Pathogenic Microorganisms, Robert Koch-Institute	Chantal Akoua-Koffi et al
EPI_ISL_614350	2020-05-30	Molecular diagnostic unit for viral haemorrhagic fevers and emerging viruses, Bouaké CHU Laboratory	Project group Epidemology of Highly Pathogenic Microorganisms, Robert Koch-Institute	Chantal Akoua-Koffi et al
EPI_ISL_730035	2020-05-30	Nigeria Centre for Disease Control (NCDC)	African Centre of Excellence for Genomics of Infectious Diseases (ACEGID), Redeemer's University, Ede, Osun State, Nigeria	Oluniyi P.E. et al et al
EPI_ISL_467499	2020-05-30	Molecular Diagnostics Services (MDS)	KRISP, KZN Research Innovation and Sequencing Platform	Giandhari J et al
EPI_ISL_561722	2020-05-31	Microbiological Diagnostic Unit - Public Health Laboratory (MDU-PHL)	MDU-PHL	Seemann et al
EPI_ISL_632285	2020-05-31	Communicable Disease Laboratory, Public Health Directorate	Communicable Disease Laboratory, Public Health Directorate	AlWasti et al
EPI_ISL_717910	2020-05-31	LACEN Dr. Francisco Rimolo Neto	Bioinformatics Laboratory / LNCC	Carolina M Voloch et al
EPI_ISL_729843	2020-05-31	Laboratorio Central de Saude Publica do Estado do Rio Grande do Sul (LACEN-RS)	Laboratory of Respiratory Viruses and Measles, Oswaldo Cruz Institute, FIOCRUZ	Paola Resende et al
EPI_ISL_574612	2020-05-31	RS Hermina Mekarsari	Eijkman Institute for Molecular Biology, Ministry of Research and Technology/National Agency for Research and Innovation	Frilasita A Yudhaputri et al
EPI_ISL_491224	2020-05-31	Instituto Gulbenkian de Ciência	Instituto Gulbenkian de Ciência	Cathy Paulino et al
EPI_ISL_466644	2020-06-01	National Institute of Laboratory Medicine and Referral Center	Genomic Research Lab, BCSIR	Abu Sayeed Mohammad Mahmud et al
EPI_ISL_475082	2020-06-01	Lab voor klinische biologie	Onderzoeksgroep Virologie	Nick Vereecke et al
EPI_ISL_717812	2020-06-01	Laboratorio de Virologia Molecular / UFRJ	Bioinformatics Laboratory / LNCC	Carolina M Voloch et al
EPI_ISL_717813	2020-06-01	Laboratorio de Virologia Molecular / UFRJ	Bioinformatics Laboratory / LNCC	Carolina M Voloch et al
EPI_ISL_717845	2020-06-01	Laboratorio de Virologia Molecular / UFRJ	Bioinformatics Laboratory / LNCC	Carolina M Voloch et al
EPI_ISL_717846	2020-06-01	Laboratorio de Virologia Molecular / UFRJ	Bioinformatics Laboratory / LNCC	Carolina M Voloch et al
EPI_ISL_717847	2020-06-01	Laboratorio de Virologia Molecular / UFRJ	Bioinformatics Laboratory / LNCC	Carolina M Voloch et al
EPI_ISL_717848	2020-06-01	Laboratorio de Virologia Molecular / UFRJ	Bioinformatics Laboratory / LNCC	Carolina M Voloch et al
EPI_ISL_717849	2020-06-01	Laboratorio de Virologia Molecular / UFRJ	Bioinformatics Laboratory / LNCC	Carolina M Voloch et al
EPI_ISL_717850	2020-06-01	Laboratorio de Virologia Molecular / UFRJ	Bioinformatics Laboratory / LNCC	Carolina M Voloch et al
EPI_ISL_717851	2020-06-01	Laboratorio de Virologia Molecular / UFRJ	Bioinformatics Laboratory / LNCC	Carolina M Voloch et al
EPI_ISL_717852	2020-06-01	Laboratorio de Virologia Molecular / UFRJ	Bioinformatics Laboratory / LNCC	Carolina M Voloch et al
EPI_ISL_717911	2020-06-01	LACEN Dr. Francisco Rimolo Neto	Bioinformatics Laboratory / LNCC	Carolina M Voloch et al
EPI_ISL_492048	2020-06-01	Instituto de Biologia do Exército	Laboratório Metabolismo Macromolecular FirminoTorres de Castro, Instituto de Biofísica Carlos Chagas Filho, Universidade Federal do Rio de Janeiro	Bianca Catarina Azevedo Cabral et al
EPI_ISL_492036	2020-06-01	Instituto de Biologia do Exército	Laboratório Metabolismo Macromolecular FirminoTorres de Castro, Instituto de Biofísica Carlos Chagas Filho, Universidade Federal do Rio de Janeiro	Bianca Catarina Azevedo Cabral et al
EPI_ISL_729841	2020-06-01	Laboratorio Central de Saude Publica do Estado do Rio Grande do Sul (LACEN-RS)	Laboratory of Respiratory Viruses and Measles, Oswaldo Cruz Institute, FIOCRUZ	Paola Resende et al
EPI_ISL_729842	2020-06-01	Laboratorio Central de Saude Publica do Estado do Rio Grande do Sul (LACEN-RS)	Laboratory of Respiratory Viruses and Measles, Oswaldo Cruz Institute, FIOCRUZ	Paola Resende et al
EPI_ISL_547571	2020-06-01	Hospital Municipal Antônio Giglio	Instituto Adolfo Lutz, Interdiciplinary Procedures Center, Strategic Laboratory	Claudio Tavares Sacchi et al
EPI_ISL_570017	2020-06-01	Unity Health Toronto	Ontario Institute for Cancer Research	Ramzi Fattouh et al
EPI_ISL_591084	2020-06-01	Viral Respiratory Lab, National Institute for Biomedical Research (INRB)	Pathogen Sequencing Lab, National Institute for Biomedical Research (INRB)	Placide Mbala-Kingebeni et al

EPI_ISL_591085	2020-06-01	Viral Respiratory Lab, National Institute for Biomedical Research (INRB)	Pathogen Sequencing Lab, National Institute for Biomedical Research (INRB)	Placide Mbala-Kingebeni et al
EPI_ISL_618019	2020-06-01	Department of Virus and Microbiological Special Diagnostics, Statens Serum Institut, Denmark	Albertsen lab, Department of Chemistry and Bioscience, Aalborg University, Denmark	Danish Covid-19 Genome Consortia et al
EPI_ISL_618015	2020-06-01	Department of Virus and Microbiological Special Diagnostics, Statens Serum Institut, Denmark	Albertsen lab, Department of Chemistry and Bioscience, Aalborg University, Denmark	Danish Covid-19 Genome Consortia et al
EPI_ISL_617972	2020-06-01	Department of Virus and Microbiological Special Diagnostics, Statens Serum Institut, Denmark	Albertsen lab, Department of Chemistry and Bioscience, Aalborg University, Denmark	Danish Covid-19 Genome Consortia et al
EPI_ISL_510526	2020-06-01	Biological prevention, army	Biological prevention, army	Seadawy et al
EPI_ISL_635779	2020-06-01	Biolab Diagnostic Laboratories	Andersen lab at Scripps Research	Issa Abu-Dayyeh et al
EPI_ISL_491231	2020-06-01	Instituto Gulbenkian de Ciência	Instituto Gulbenkian de Ciência	Joao Sobral et al
EPI_ISL_471416	2020-06-01	Laboratory for Respiratory Viruses, National Influenza Centre, Cantacuzino National Military-Medical Institute for Research and Development	Cantacuzino Institute	Luiza Ustea et al
EPI_ISL_467509	2020-06-01	NHLS-IALCH	KRISP, KZN Research Innovation and Sequencing Platform	Giandhari J et al
EPI_ISL_467510	2020-06-01	NHLS-IALCH	KRISP, KZN Research Innovation and Sequencing Platform	Giandhari J et al
EPI_ISL_647971	2020-06-01	National Microbiology Reference Laboratory	Quadram Institute Bioscience	Thanh Le Viet et al
EPI_ISL_523116	2020-06-01	Dutch COVID-19 response team	Erasmus Medical Center	OH consortium et al
EPI_ISL_717834	2020-06-02	LACEN Dr. Francisco Rimolo Neto	Bioinformatics Laboratory / LNCC	Carolina M Voloch et al
EPI_ISL_717912	2020-06-02	LACEN Dr. Francisco Rimolo Neto	Bioinformatics Laboratory / LNCC	Carolina M Voloch et al
EPI_ISL_717918	2020-06-02	LACEN Dr. Francisco Rimolo Neto	Bioinformatics Laboratory / LNCC	Carolina M Voloch et al
EPI_ISL_729847	2020-06-02	Laboratorio Central de Saude Publica do Estado do Rio Grande do Sul (LACEN-RS)	Laboratory of Respiratory Viruses and Measles, Oswaldo Cruz Institute, FIOCRUZ	Paola Resende et al
EPI_ISL_593536	2020-06-02	Eastern Ontario Regional Laboratory Association	McMaster University	Leanne Mortimer et al
EPI_ISL_478672	2020-06-02	Egyptian National Cancer Institute (ENCI)	Egyptian National Cancer Institute (ENCI)	Zekri et al
EPI_ISL_477161	2020-06-02	Egyptian National Cancer Institute (ENCI)	Egyptian National Cancer Institute (ENCI)	Zekri et al
EPI_ISL_475724	2020-06-02	Egyptian National Cancer Institute (ENCI)	Egyptian National Cancer Institute (ENCI)	Zekri et al
EPI_ISL_510532	2020-06-02	Biological prevention, army	Biological prevention, army	Seadawy et al
EPI_ISL_516889	2020-06-02	Israel Central Virology laboratory	Israel Central Virology laboratory	Neta Zuckerman et al
EPI_ISL_603157	2020-06-02	INMI Lazzaro Spallanzani IRCCS	INMI Lazzaro Spallanzani IRCCS	Francesco Messina et al
EPI_ISL_527887	2020-06-02	Nigeria Centre for Disease Control (NCDC)	African Centre of Excellence for Genomics of Infectious Diseases (ACEGID), Redeemer's University, Ede, Osun State, Nigeria	Oluniyi P.E. et al et al
EPI_ISL_514354	2020-06-02	General Hospital - Prilep	Research Center for Genetic Engineering and Biotechnology "Georgi D. Efremov" , Macedonian Academy of Sciences and Arts	RCGEB - MASA et al
EPI_ISL_468159	2020-06-02	unknown	Department of Virology, Public Health Laboratories Division, National Institute of Health	Massab Umair et al
EPI_ISL_468161	2020-06-02	Department of Virology, Public Health Laboratories Division, National Institute of Health	Department of Virology, Public Health Laboratories Division, National Institute of Health	Massab Umair et al
EPI_ISL_468160	2020-06-02	unknown	Department of Virology, Public Health Laboratories Division, National Institute of Health	Massab Umair et al
EPI_ISL_468163	2020-06-02	Department of Virology, Public Health Laboratories Division, National Institute of Health	Department of Virology, Public Health Laboratories Division, National Institute of Health	Massab Umair et al
EPI_ISL_482684	2020-06-02	Singapore General Hospital	Department of Microbiology	Nurdyana Abdul Rahman et al
EPI_ISL_482683	2020-06-02	Singapore General Hospital	Department of Microbiology	Nurdyana Abdul Rahman et al
EPI_ISL_482686	2020-06-02	Singapore General Hospital	Department of Microbiology	Nurdyana Abdul Rahman et al
EPI_ISL_717833	2020-06-03	LACEN Dr. Francisco Rimolo Neto	Bioinformatics Laboratory / LNCC	Carolina M Voloch et al
EPI_ISL_717836	2020-06-03	LACEN Dr. Francisco Rimolo Neto	Bioinformatics Laboratory / LNCC	Carolina M Voloch et al

EPI_ISL_591083	2020-06-03	Viral Respiratory Lab, National Institute for Biomedical Research (INRB)	Pathogen Sequencing Lab, National Institute for Biomedical Research (INRB)	Placide Mbala-Kingebeni et al
EPI_ISL_591086	2020-06-03	Viral Respiratory Lab, National Institute for Biomedical Research (INRB)	Pathogen Sequencing Lab, National Institute for Biomedical Research (INRB)	Placide Mbala-Kingebeni et al
EPI_ISL_472101	2020-06-03	Liverpool Clinical Laboratories	COVID-19 Genomics UK (COG-UK) Consortium	Sam Haldenby et al
EPI_ISL_683363	2020-06-03	CNR Virus des Infections Respiratoires - France SUD	CNR Virus des Infections Respiratoires - France SUD	Antonin Bal et al
EPI_ISL_481159	2020-06-03	Immunogenomics lab, Institute of Life Sciences, Bhubaneswar	Immunogenomics lab, Institute of Life Sciences, Bhubaneswar	Sunil Raghav et al
EPI_ISL_574613	2020-06-03	RS Harapan Bunda	Eijkman Institute for Molecular Biology, Ministry of Research and Technology/National Agency for Research and Innovation	Frilasita A Yudhaputri et al
EPI_ISL_707928	2020-06-03	Fujita Health University Hospital	Fujita Health University School of Medicine, Department of Microbiology	Masahiro Suzuki et al
EPI_ISL_560405	2020-06-03	Vilnius University Hospital Santaros Klinikos, Vilnius University	Institute of Biotechnology, Life Sciences Center, Vilnius University and Thermo Fisher Scientific	Justinas Slikas et al
EPI_ISL_582124	2020-06-03	Malaysia Genome Institute	Malaysia Genome Institute	Mohd Noor Mat Isa et al
EPI_ISL_492014	2020-06-03	Oman-NIC	Department of Microbiology and Immunology-SQUH	Fahad Zadjali et al
EPI_ISL_491242	2020-06-03	Instituto Gulbenkian de Ciência	Instituto Gulbenkian de Ciência	Joao Sobral et al
EPI_ISL_471417	2020-06-03	Laboratory for Respiratory Viruses, National Influenza Centre, Cantacuzino National Military-Medical Institute for Research and Development	Cantacuzino Institute	Luiza Ustea et al
EPI_ISL_471419	2020-06-03	Laboratory for Respiratory Viruses, National Influenza Centre, Cantacuzino National Military-Medical Institute for Research and Development	Cantacuzino Institute	Luiza Ustea et al
EPI_ISL_574492	2020-06-03	Programme in Emerging Infectious Diseases, Duke-NUS Medical School	National Public Health Laboratory, National Centre for Infectious Diseases	Tze Minn Mak et al
EPI_ISL_495436	2020-06-03	Kafkas University, Faculty of Medicine, Department of Medical Microbiology	Kafkas University, Faculty of Medicine, Department of Medical Microbiology	Murat Karamese et al
EPI_ISL_653748	2020-06-04	Instituto Nacional de Salud, Bogotá, Colombia	Instituto Nacional de Salud, Bogotá, Colombia	Katherine Laiton-Donato et al
EPI_ISL_614352	2020-06-04	Molecular diagnostic unit for viral haemorrhagic fevers and emerging viruses, Bouaké CHU Laboratory	Project group Epidemology of Highly Pathogenic Microorganisms, Robert Koch-Institute	Chantal Akoua-Koffi et al
EPI_ISL_471269	2020-06-04	Hospital Oncológico Solca Núcleo de Quito	Institute of Microbiology, Universidad San Francisco de Quito	Sully Márquez et al
EPI_ISL_648358	2020-06-04	Laboratorio de Investigaciones de Baney	University Hospital Basel, Clinical Bacteriology	Carlos Cortes et al
EPI_ISL_526224	2020-06-04	Hungarian Defence Forces Military Medical Centre	National Laboratory of Virology, Szentágothai Research Centre	Endre Gábor Tóth et al
EPI_ISL_495214	2020-06-04	CSIR-Centre for Cellular and Molecular Biology	CSIR-Centre for Cellular and Molecular Biology	Tulasi Nagabandi et al
EPI_ISL_501260	2020-06-04	National Virus Reference Laboratory	National Virus Reference Laboratory	Michael Carr et al
EPI_ISL_635780	2020-06-04	Biolab Diagnostic Laboratories	Andersen lab at Scripps Research	Issa Abu-Dayyeh et al
EPI_ISL_528743	2020-06-04	Malaysia Genome Institute	Malaysia Genome Institute	Mohd Noor Mat Isa et al
EPI_ISL_528744	2020-06-04	Malaysia Genome Institute	Malaysia Genome Institute	Mohd Noor Mat Isa et al
EPI_ISL_496854	2020-06-04	Gorgas Memorial Laboratory of Health Studies	Gorgas Memorial Laboratory of Health Studies	Danilo Franco et al
EPI_ISL_582789	2020-06-04	Orebro klinisk mikrobiologi	The Public Health Agency of Sweden	Anna-Malin Linde et al
EPI_ISL_548254	2020-06-04	Klinisk mikrobiologi NAL Trollhattan	The Public Health Agency of Sweden	Anna-Malin Linde et al
EPI_ISL_484699	2020-06-05	Department of Clinical Microbiology	GIGA Medical Genomics	Keith Durkin et al
EPI_ISL_514138	2020-06-05	Rondônia Central Public Health Laboratory (LACEN/RO), vinculated to State Health Secretariat of Rondônia (SESAU/RO)	Molecular Virology Laboratory of Oswaldo Cruz Foundation of Rondônia	Luan Felipo Botelho-Souza et al
EPI_ISL_568994	2020-06-05	MEPHI, Aix Marseille University	MEPHI, Aix Marseille University	Anthony LEVASSEUR et al
EPI_ISL_729556	2020-06-05	Charité Universitätsmedizin Berlin, Institut für Virologie/Labor Berlin	Charité Universitätsmedizin Berlin, Institut für Virologie	Victor M Corman et al
EPI_ISL_574615	2020-06-05	RS Harapan Bunda	Eijkman Institute for Molecular Biology, Ministry of Research and Technology/National Agency for Research and Innovation	Frilasita A Yudhaputri et al

EPI_ISL_516899	2020-06-05	Israel Central Virology laboratory	Israel Central Virology laboratory	Neta Zuckerman et al
EPI_ISL_568723	2020-06-05	KEMRI-Wellcome Trust Research Programme/KEMRI-CGMR-C Kilifi	KEMRI-Wellcome Trust Research Programme/KEMRI-CGMR-C Kilifi	Githinji et al 2020 et al
EPI_ISL_568725	2020-06-05	KEMRI-Wellcome Trust Research Programme/KEMRI-CGMR-C Kilifi	KEMRI-Wellcome Trust Research Programme/KEMRI-CGMR-C Kilifi	Githinji et al 2020 et al
EPI_ISL_525429	2020-06-05	Oman-National Influenza Center	Biotechnology & OMICs Laboratory	Samira Al-Mahruqi et al
EPI_ISL_511579	2020-06-05	Instituto Nacional de Saude (INSA)	Instituto Nacional de Saude (INSA)	Borges et al et al
EPI_ISL_510110	2020-06-05	Hospital General Universitario Gregorio Marañón	SeqCOVID-SPAIN consortium/IBV(CSIC)	Laura Pérez-Lago et al
EPI_ISL_560974	2020-06-05	Karolinska Universitetslaboratoriet	The Public Health Agency of Sweden	Anna-Malin Linde et al
EPI_ISL_483659	2020-06-05	Viollier AG	Department of Biosystems Science and Engineering, ETH Zürich	Christian Beisel et al
EPI_ISL_495429	2020-06-05	Kafkas University, Faculty of Medicine, Department of Medical Microbiology	Kafkas University, Faculty of Medicine, Department of Medical Microbiology	Murat Karamese et al
EPI_ISL_570015	2020-06-06	Unity Health Toronto	Ontario Institute for Cancer Research	Ramzi Fattouh et al
EPI_ISL_653759	2020-06-06	Instituto Nacional de Salud, Bogotá, Colombia	Instituto Nacional de Salud, Bogotá, Colombia	Katherine Laiton-Donato et al
EPI_ISL_581489	2020-06-06	Fondation Congolaise pour la recherche medicale (FCRM)	NGS Competence Center Tübingen, Institut für Medizinische Mikrobiologie und Hygiene, Universitätsklinikum Tübingen	Angel Angelov et al
EPI_ISL_581455	2020-06-06	Fondation Congolaise pour la recherche medicale (FCRM)	NGS Competence Center Tübingen, Institut für Medizinische Mikrobiologie und Hygiene, Universitätsklinikum Tübingen	Angel Angelov et al
EPI_ISL_581490	2020-06-06	Fondation Congolaise pour la recherche medicale (FCRM)	NGS Competence Center Tübingen, Institut für Medizinische Mikrobiologie und Hygiene, Universitätsklinikum Tübingen	Angel Angelov et al
EPI_ISL_603149	2020-06-06	INMI Lazzaro Spallanzani IRCCS	INMI Lazzaro Spallanzani IRCCS	Francesco Messina et al
EPI_ISL_501287	2020-06-06	Centrālā laboratorija	Latvian Biomedical Research and Study Centre	Ivars Silamiķelis et al
EPI_ISL_577876	2020-06-06	Dutch COVID-19 response team	Erasmus Medical Center	Bas Oude Munnink et al
EPI_ISL_645109	2020-06-06	Human Genome Variation Research Group, Malopolska Centre of Biotechnology	Human Genome Variation Research Group, Malopolska Centre of Biotechnology	Kowalski et al
EPI_ISL_645078	2020-06-06	Human Genome Variation Research Group, Malopolska Centre of Biotechnology	Human Genome Variation Research Group, Malopolska Centre of Biotechnology	Kowalski et al
EPI_ISL_645079	2020-06-06	Human Genome Variation Research Group, Malopolska Centre of Biotechnology	Human Genome Variation Research Group, Malopolska Centre of Biotechnology	Kowalski et al
EPI_ISL_645081	2020-06-06	Human Genome Variation Research Group, Malopolska Centre of Biotechnology	Human Genome Variation Research Group, Malopolska Centre of Biotechnology	Kowalski et al
EPI_ISL_645082	2020-06-06	Human Genome Variation Research Group, Malopolska Centre of Biotechnology	Human Genome Variation Research Group, Malopolska Centre of Biotechnology	Kowalski et al
EPI_ISL_507288	2020-06-06	WHO National Influenza Centre Russian Federation	WHO National Influenza Centre Russian Federation	Andrey Komissarov et al
EPI_ISL_495421	2020-06-06	Kafkas University, Faculty of Medicine, Department of Medical Microbiology	Kafkas University, Faculty of Medicine, Department of Medical Microbiology	Murat Karamese et al
EPI_ISL_495433	2020-06-06	Kafkas University, Faculty of Medicine, Department of Medical Microbiology	Kafkas University, Faculty of Medicine, Department of Medical Microbiology	Murat Karamese et al
EPI_ISL_475084	2020-06-07	National Institute of Laboratory Medicine and Referral Center	Genomic Research Lab, BCSIR	Md. Murshed Hasan Sarkar et al
EPI_ISL_603023	2020-06-07	Vigilância em Saúde Visa Sul	Instituto Adolfo Lutz, Interdiciplinary Procedures Center, Strategic Laboratory	Claudio Tavares Sacchi et al
EPI_ISL_471422	2020-06-07	Laboratory for Respiratory Viruses, National Influenza Centre, Cantacuzino National Military-Medical Institute for Research and Development	Cantacuzino Institute	Luiza Ustea et al
EPI_ISL_569803	2020-06-07	Omsk Research Institute of Natural Focal Infections	WHO National Influenza Centre Russian Federation	Artem Fadeev et al
EPI_ISL_591528	2020-06-08	Medicina Norte U Chile - Servicio Medico Legal	Center for Mathematical Modeling and Center for Genome Regulation. Santiago, Chile	Gaggero A et al
EPI_ISL_653761	2020-06-08	Instituto Nacional de Salud, Bogotá, Colombia	Instituto Nacional de Salud, Bogotá, Colombia	Katherine Laiton-Donato et al
EPI_ISL_636987	2020-06-08	Virology Lab, National Institute for Biomedical Research (INRB)	Project group Epidemology of Highly Pathogenic Microorganisms, Robert Koch-Institute	Jean-Jacques Muyembe Tamfum et al

EPI_ISL_618077	2020-06-08	Department of Virus and Microbiological Special Diagnostics, Statens Serum Institut, Denmark	Albertsen lab, Department of Chemistry and Bioscience, Aalborg University, Denmark	Danish Covid-19 Genome Consortia et al
EPI_ISL_523410	2020-06-08	Dutch COVID-19 response team	Erasmus Medical Center	Bas Oude Munnink et al
EPI_ISL_529067	2020-06-08	Laboratorio de Referencia Nacional de Virus Respiratorios, Instituto Nacional de Salud Peru	Laboratorio de Genómica Microbiana, Universidad Peruana Cayetano Heredia	Pablo Tsukayama et al
EPI_ISL_491259	2020-06-08	Instituto Gulbenkian de Ciência	Instituto Gulbenkian de Ciência	Joao Sobral et al
EPI_ISL_526282	2020-06-09	Unity Health Toronto	Ontario Institute for Cancer Research	Ramzi Fattouh et al
EPI_ISL_644252	2020-06-09	CEPHR / Mater Hospital	Irish Coronavirus Sequencing Consortium - National Virus Reference Laboratory	Michael Carr et al
EPI_ISL_516415	2020-06-09	Center for public health - Skopje	Research Center for Genetic Engineering and Biotechnology "Georgi D. Efremov" , Macedonian Academy of Sciences and Arts	RCGEB - MASA et al
EPI_ISL_492022	2020-06-09	Oman-NIC	Department of Microbiology and Immunology-SQUH	Fahad Zadjali et al
EPI_ISL_492024	2020-06-09	Oman-NIC	Department of Microbiology and Immunology-SQUH	Fahad Zadjali et al
EPI_ISL_491046	2020-06-09	Suceava County Emergency Hospital	"Stefan cel Mare" University Metagenomics Lab	Lobiuc Andrei et al
EPI_ISL_522350	2020-06-10	KU Leuven, Rega Institute, Clinical and Epidemiological Virology	KU Leuven, Rega Institute, Clinical and Epidemiological Virology	Tony Wawina-Bokalanga et al
EPI_ISL_541082	2020-06-10	The National Institute of Public Health	State Veterinary Institute Prague	Nagy et al
EPI_ISL_648362	2020-06-10	Laboratorio de Investigaciones de Baney	University Hospital Basel, Clinical Bacteriology	Carlos Cortes et al
EPI_ISL_603173	2020-06-10	INMI Lazzaro Spallanzani IRCCS	INMI Lazzaro Spallanzani IRCCS	Barbara Bartolini et al
EPI_ISL_644946	2020-06-10	Department of Infectious Diseases, Keio University School of Medicine, Tokyo, Japan	Center for Medical Genetics, Keio University School of Medicine, Tokyo, Japan	Kenjiro Kosaki et al
EPI_ISL_683835	2020-06-10	CICM	Malaria Research and Training Center (MRTC-Parasito)	Antoine Dara et al
EPI_ISL_708840	2020-06-10	National Institute of Blood Diseases (NIBD), Molecular Biology Lab	Genomics Lab NIBD	Samina Naz Mukry et al
EPI_ISL_476493	2020-06-10	Institut Pasteur Dakar	Institut Pasteur de Dakar	Ndongo Dia et al
EPI_ISL_699122	2020-06-10	Group 42 (G42) Healthcare, Abu Dhabi, United Arab Emirates; Department of Health, The United Arab Emirates	G42 Healthcare	Rong Liu et al
EPI_ISL_699135	2020-06-10	Group 42 (G42) Healthcare, Abu Dhabi, United Arab Emirates; Department of Health, The United Arab Emirates	G42 Healthcare	Rong Liu et al
EPI_ISL_469254	2020-06-11	National Institute for Viral Disease Control and Prevention, China CDC	Institute of Viral Disease Control and Prevention, China CDC	Wenjie Tan et al
EPI_ISL_469255	2020-06-11	National Institute for Viral Disease Control and Prevention, China CDC	Institute of Viral Disease Control and Prevention, China CDC	Xiang Zhao et al
EPI_ISL_603025	2020-06-11	UPA Central de Caraguatatuba	Instituto Adolfo Lutz, Interdiciplinary Procedures Center, Strategic Laboratory	Claudio Tavares Sacchi et al
EPI_ISL_591529	2020-06-11	Medicina Norte U Chile - Servicio Medico Legal	Center for Mathematical Modeling and Center for Genome Regulation. Santiago, Chile	Gaggero A et al
EPI_ISL_512661	2020-06-11	Area De Salud Los Chiles	Inciensa, Instituto Costarricense de Investigación y Enseñanza en Nutrición y Salud	Francisco Duarte et al
EPI_ISL_471529	2020-06-11	Department for Virology, Molecular Biology and Genome Research, R. G. Lugar Center for Public Health Research, National Center for Disease Control and Public Health (NCDC) of Georgia.	Department for Virology, Molecular Biology and Genome Research, R. G. Lugar Center for Public Health Research, National Center for Disease Control and Public Health (NCDC) of Georgia.	Meri Pantsulaia et al
EPI_ISL_596455	2020-06-11	Department of Pathology, School of Medicine, Imam Khomeini Hospital, Tehran University of Medical Sciences	Genetics Research Center, University of Social Welfare and Rehabilitation Sciences	Zohreh Fattahi et al
EPI_ISL_644947	2020-06-11	Department of Infectious Diseases, Keio University School of Medicine, Tokyo, Japan	Center for Medical Genetics, Keio University School of Medicine, Tokyo, Japan	Kenjiro Kosaki et al
EPI_ISL_677706	2020-06-11	Center for public health - Skopje	Research Center for Genetic Engineering and Biotechnology "Georgi D. Efremov" , Macedonian Academy of Sciences and Arts	RCGEB - MASA et al
EPI_ISL_516421	2020-06-11	Center for public health - Skopje	Research Center for Genetic Engineering and Biotechnology "Georgi D. Efremov" , Macedonian Academy of Sciences and Arts	RCGEB - MASA et al
EPI_ISL_539544	2020-06-11	Hospital Clínic	Instituto de Salud Carlos III	Iglesias-Caballero et al

EPI_ISL_475569	2020-06-11	Kungsholmsdoktorn	The Public Health Agency of Sweden	Oskar Karlsson Lindsjo et al
EPI_ISL_698121	2020-06-11	Group 42 (G42) Healthcare, Abu Dhabi, United Arab Emirates; Department of Health, The United Arab Emirates	G42 Healthcare	Rong Liu et al
EPI_ISL_469256	2020-06-11	National Institute for Viral Disease Control and Prevention, China CDC	National Institute for Viral Disease Control and Prevention, China CDC	Xiang Zhao et al
EPI_ISL_678320	2020-06-12	Area of Virology, Serology and Virology Division (SAViD), New South Wales Health Pathology Randwick	Virology Research Laboratory; Area of Virology, Serology and Virology Division (SAViD), New South Wales Health Pathology Randwick	Foster et al
EPI_ISL_490026	2020-06-12	South Eastern Area Laboratory Services (SEALS)	NSW Health Pathology - Institute of Clinical Pathology and Medical Research; Westmead Hospital; University of Sydney	CIDM-PH et al. et al
EPI_ISL_600512	2020-06-12	Institute of Epidemiology Disease Control And Research	Institute for Developing Science and Health Initiatives	Lauren Cowley et al
EPI_ISL_512662	2020-06-12	Area De Salud La Cruz	Inciensa, Instituto Costarricense de Investigación y Enseñanza en Nutrición y Salud	Francisco Duarte et al
EPI_ISL_614359	2020-06-12	Molecular diagnostic unit for viral haemorrhagic fevers and emerging viruses, Bouaké CHU Laboratory	Project group Epidemology of Highly Pathogenic Microorganisms, Robert Koch-Institute	Chantal Akoua-Koffi et al
EPI_ISL_648124	2020-06-12	UHAS COVID-19 Lab	UHAS COVID-19 Lab	Kwabena O. Duedu et al
EPI_ISL_648125	2020-06-12	UHAS COVID-19 Lab	UHAS COVID-19 Lab	Kwabena O. Duedu et al
EPI_ISL_501261	2020-06-12	National Virus Reference Laboratory	National Virus Reference Laboratory	Michael Carr et al
EPI_ISL_603144	2020-06-12	INMI Lazzaro Spallanzani IRCCS	INMI Lazzaro Spallanzani IRCCS	Cesare E.M. Gruber et al
EPI_ISL_603147	2020-06-12	INMI Lazzaro Spallanzani IRCCS	INMI Lazzaro Spallanzani IRCCS	Cesare E.M. Gruber et al
EPI_ISL_514166	2020-06-12	Florida Bureau of Public Health Laboratories	Florida Bureau of Public Health Laboratories	Sarah Schmedes et al
EPI_ISL_527012	2020-06-13	Area of Virology, Serology and Virology Division (SAViD), New South Wales Health Pathology Randwick	Area of Virology, Serology and Virology Division (SAViD), New South Wales Health Pathology Randwick	Rawlinson et al
EPI_ISL_614357	2020-06-13	Molecular diagnostic unit for viral haemorrhagic fevers and emerging viruses, Bouaké CHU Laboratory	Project group Epidemology of Highly Pathogenic Microorganisms, Robert Koch-Institute	Chantal Akoua-Koffi et al
EPI_ISL_538507	2020-06-13	Balai Penelitian dan Pengembangan Biomedis Papua	National Institute of Health Research and Development	Pawestri et al
EPI_ISL_538506	2020-06-13	Balai Penelitian dan Pengembangan Biomedis Papua	National Institute of Health Research and Development	Pawestri et al
EPI_ISL_644948	2020-06-13	Department of Infectious Diseases, Keio University School of Medicine, Tokyo, Japan	Center for Medical Genetics, Keio University School of Medicine, Tokyo, Japan	Kenjiro Kosaki et al
EPI_ISL_516618	2020-06-13	Instituto de Diagnostico y Referencia Epidemiologicos (INDRE)	Instituto de Diagnostico y Referencia Epidemiologicos (INDRE)	Gisela Barrera-Badillo et al
EPI_ISL_527911	2020-06-13	Nigeria Centre for Disease Control (NCDC)	African Centre of Excellence for Genomics of Infectious Diseases (ACEGID), Redeemer's University, Ede, Osun State, Nigeria	Oluniyi P.E. et al et al
EPI_ISL_476569	2020-06-13	Institut Pasteur Dakar	Institut Pasteur de Dakar	Ndongo Dia et al
EPI_ISL_497021	2020-06-13	Washington State Department of Health	Seattle Flu Study	Deborah A. Nickerson et al
EPI_ISL_569995	2020-06-14	Unity Health Toronto	Ontario Institute for Cancer Research	Ramzi Fattouh et al
EPI_ISL_594156	2020-06-14	Israel Institute for Biological Research	Israel Institute for Biological Research	Galia Zaide et al
EPI_ISL_523364	2020-06-14	Dutch COVID-19 response team	Erasmus Medical Center	Bas Oude Munnink et al
EPI_ISL_477152	2020-06-14	Institut Pasteur Dakar	Institut Pasteur de Dakar	Ndongo Dia et al
EPI_ISL_517621	2020-06-14	Academic Hospital Paramaribo	Erasmus Medical Center	Bas Oude Munnink et al
EPI_ISL_618139	2020-06-15	Department of Virus and Microbiological Special Diagnostics, Statens Serum Institut, Denmark	Albertsen lab, Department of Chemistry and Bioscience, Aalborg University, Denmark	Danish Covid-19 Genome Consortia et al
EPI_ISL_539783	2020-06-15	Universidad Regional Amazonica IKIAM	Institute of Microbiology, Universidad San Francisco de Quito	Fabian Aguilar et al
EPI_ISL_648365	2020-06-15	Laboratorio de Investigaciones de Baney	University Hospital Basel, Clinical Bacteriology	Carlos Cortes et al
EPI_ISL_501896	2020-06-15	Centrālā laboratorija	Latvian Biomedical Research and Study Centre	Ivars Silamiķelis et al
EPI_ISL_635521	2020-06-15	Centro de Diagn√≥stico COVID-19 UABC Tijuana	Andersen lab at Scripps Research	SEARCH Alliance San Diego with Idanya Rubí Serafín Higuera et al
EPI_ISL_485710	2020-06-15	Institut Pasteur Dakar	Institut Pasteur de Dakar	Ndongo Dia et al
EPI_ISL_676592	2020-06-15	Scientific Veterinary Institute Novi Sad	Veterinary Specialized Institute "Kraljevo", Serbia	Vidanovic et al

EPI_ISL_623087	2020-06-15	Klinisk mikrobiologi NAL Trollhattan	The Public Health Agency of Sweden	Anna-Malin Linde et al
EPI_ISL_514175	2020-06-15	Florida Bureau of Public Health Laboratories	Florida Bureau of Public Health Laboratories	Sarah Schmedes et al
EPI_ISL_614256	2020-06-15	Wyoming Public Health Laboratory	Center for Global Health, University of New Mexico Health Sciences Center	Daryl Domman et al
EPI_ISL_547575	2020-06-16	SVO Jundiaí	Instituto Adolfo Lutz, Interdiciplinary Procedures Center, Strategic Laboratory	Claudio Tavares Sacchi et al
EPI_ISL_693229	2020-06-16	Hospital 8 de Maio	Instituto Adolfo Lutz, Interdiciplinary Procedures Center, Strategic Laboratory	Claudio Tavares Sacchi et al
EPI_ISL_591530	2020-06-16	Medicina Norte U Chile - Servicio Medico Legal	Center for Mathematical Modeling and Center for Genome Regulation. Santiago, Chile	Gaggero A et al
EPI_ISL_577633	2020-06-16	The National Institute of Public Health	State Veterinary Institute Prague	Nagy et al
EPI_ISL_487415	2020-06-16	Labor Kneißler GmbH & Co. KG	Heinrich Pette Institute, Leibniz Institute for Experimental Virology	Thomas Günther et al
EPI_ISL_628761	2020-06-16	UHAS COVID-19 Lab	UHAS COVID-19 Lab	Kwabena O. Duedu et al
EPI_ISL_477243	2020-06-16	Institute for Stem Cell Science and Regenerative Medicine	National Centre for Biological Sciences	Farhan Ali et al
EPI_ISL_574619	2020-06-16	BTKLPP Kelas I Manado	Eijkman Institute for Molecular Biology, Ministry of Research and Technology/National Agency for Research and Innovation	Hidayat Trimarsanto et al
EPI_ISL_596451	2020-06-16	Department of Pathology, School of Medicine, Imam Khomeini Hospital, Tehran University of Medical Sciences	Genetics Research Center, University of Social Welfare and Rehabilitation Sciences	Zohreh Fattahi et al
EPI_ISL_501915	2020-06-16	Centrālā laboratorija	Latvian Biomedical Research and Study Centre	Ivars Silamiķelis et al
EPI_ISL_501922	2020-06-16	Centrālā laboratorija	Latvian Biomedical Research and Study Centre	Ivars Silamiķelis et al
EPI_ISL_498226	2020-06-16	LIC	LIC	LIC et al
EPI_ISL_661179	2020-06-16	Scientific Veterinary Institute Novi Sad	Veterinary Specialized Institute "Kraljevo", Serbia	Vidanovic et al
EPI_ISL_482871	2020-06-16	Molecular Diagnostics Services (MDS)	KRISP, KZN Research Innovation and Sequencing Platform	Giandhari J et al
EPI_ISL_482872	2020-06-16	Molecular Diagnostics Services (MDS)	KRISP, KZN Research Innovation and Sequencing Platform	Giandhari J et al
EPI_ISL_525481	2020-06-16	Centre for Dengue Research	Centre for Dengue Research	Chandima Jeewandara et al
EPI_ISL_681685	2020-06-17	Molecular Medicine Laboratory, University of Magallanes	Centro Asistencial Docente y de Investigacion, Universidad de Magallanes	Jorge González et al
EPI_ISL_526974	2020-06-17	Instituto Nacional de Salud, Bogotá, Colombia	Instituto Nacional de Salud, Bogotá, Colombia	Katherine Laiton-Donato et al
EPI_ISL_508214	2020-06-17	Government Medical College	National Institute of Biomedical Genomics	Arindam Maitra et al
EPI_ISL_516938	2020-06-17	Nicolae Testemitanu State University of Medicine and Pharmacy	International Centre for Genetic Engineering and Biotechnology (ICGEB) and ARGO Open Lab Platform for Genome Sequencing	Ulinici M et al
EPI_ISL_516936	2020-06-17	Nicolae Testemitanu State University of Medicine and Pharmacy	International Centre for Genetic Engineering and Biotechnology (ICGEB) and ARGO Open Lab Platform for Genome Sequencing	Ulinici M et al
EPI_ISL_527915	2020-06-17	Nigeria Centre for Disease Control (NCDC)	African Centre of Excellence for Genomics of Infectious Diseases (ACEGID), Redeemer's University, Ede, Osun State, Nigeria	Oluniyi P.E. et al et al
EPI_ISL_540953	2020-06-17	Laboratorio de Referencia Nacional de Virus Respiratorios, Instituto Nacional de Salud Peru	Laboratorio de Genómica Microbiana, Universidad Peruana Cayetano Heredia	Pablo Tsukayama et al
EPI_ISL_540926	2020-06-17	Laboratorio de Referencia Nacional de Virus Respiratorios, Instituto Nacional de Salud Peru	Laboratorio de Genómica Microbiana, Universidad Peruana Cayetano Heredia	Pablo Tsukayama et al
EPI_ISL_540934	2020-06-17	Laboratorio de Referencia Nacional de Virus Respiratorios, Instituto Nacional de Salud Peru	Laboratorio de Genómica Microbiana, Universidad Peruana Cayetano Heredia	Pablo Tsukayama et al
EPI_ISL_540969	2020-06-17	Laboratorio de Referencia Nacional de Virus Respiratorios, Instituto Nacional de Salud Peru	Laboratorio de Genómica Microbiana, Universidad Peruana Cayetano Heredia	Pablo Tsukayama et al
EPI_ISL_540981	2020-06-17	Laboratorio de Referencia Nacional de Virus Respiratorios, Instituto Nacional de Salud Peru	Laboratorio de Genómica Microbiana, Universidad Peruana Cayetano Heredia	Pablo Tsukayama et al
EPI_ISL_569810	2020-06-17	Omsk Research Institute of Natural Focal Infections	WHO National Influenza Centre Russian Federation	Artem Fadeev et al
EPI_ISL_517622	2020-06-17	Academic Hospital Paramaribo	Erasmus Medical Center	Bas Oude Munnink et al
EPI_ISL_480445	2020-06-18	National Institute of Laboratory Medicine and Referral Center	Genomic Research Lab, BCSIR	Md. Ahasan Habib et al
EPI_ISL_498227	2020-06-18	National Institute of Laboratory Medicine and Referral Center	Genomic Research Lab, BCSIR	Shahina Akter et al

EPI_ISL_498628	2020-06-18	Department of Clinical Microbiology	GIGA Medical Genomics	Keith Durkin et al
EPI_ISL_498629	2020-06-18	Department of Clinical Microbiology	GIGA Medical Genomics	Keith Durkin et al
EPI_ISL_574597	2020-06-18	Secretaria Municipal de Saude de Jarinu	Instituto Adolfo Lutz, Interdiciplinary Procedures Center, Strategic Laboratory	Claudio Tavares Sacchi et al
EPI_ISL_693213	2020-06-18	Hospital E Maternidade Municipal Governador Mario Covas	Instituto Adolfo Lutz, Interdiciplinary Procedures Center, Strategic Laboratory	Claudio Tavares Sacchi et al
EPI_ISL_681686	2020-06-18	Molecular Medicine Laboratory, University of Magallanes	Centro Asistencial Docente y de Investigacion, Universidad de Magallanes	Jorge González et al
EPI_ISL_631279	2020-06-18	MVZ DIAMEDIS Diagnostische Medizin Sennestadt GmbH	Bielefeld University	David Brandt et al
EPI_ISL_516934	2020-06-18	Nicolae Testemitanu State University of Medicine and Pharmacy	International Centre for Genetic Engineering and Biotechnology (ICGEB) and ARGO Open Lab Platform for Genome Sequencing	Ulinici M et al
EPI_ISL_540942	2020-06-18	Laboratorio de Referencia Nacional de Virus Respiratorios, Instituto Nacional de Salud Peru	Laboratorio de Genómica Microbiana, Universidad Peruana Cayetano Heredia	Pablo Tsukayama et al
EPI_ISL_540992	2020-06-18	Laboratorio de Referencia Nacional de Virus Respiratorios, Instituto Nacional de Salud Peru	Laboratorio de Genómica Microbiana, Universidad Peruana Cayetano Heredia	Pablo Tsukayama et al
EPI_ISL_568517	2020-06-18	Laboratorio de Referencia Nacional de Virus Respiratorios, Instituto Nacional de Salud Peru	Laboratorio de Genómica Microbiana, Universidad Peruana Cayetano Heredia	Pablo Tsukayama et al
EPI_ISL_482878	2020-06-18	Institut Pasteur Dakar	Institut Pasteur de Dakar	Ndongo Dia et al
EPI_ISL_478718	2020-06-19	Sydney South West Pathology Service (SSWPS) - Concord Repatriation General Hospital - NSW Health Pathology	NSW Health Pathology - Institute of Clinical Pathology and Medical Research; Westmead Hospital; University of Sydney	CIDM-PH et al. et al
EPI_ISL_574596	2020-06-19	CS II Dr. Antonio Vicoso Moreira de Rezende Sumare	Instituto Adolfo Lutz, Interdiciplinary Procedures Center, Strategic Laboratory	Claudio Tavares Sacchi et al
EPI_ISL_512666	2020-06-19	Area De Salud La Cruz	Inciensa, Instituto Costarricense de Investigación y Enseñanza en Nutrición y Salud	Francisco Duarte et al
EPI_ISL_512670	2020-06-19	Centro Nacional De Rehabilitacion Humberto Araya Rojas (Cenare)	Inciensa, Instituto Costarricense de Investigación y Enseñanza en Nutrición y Salud	Francisco Duarte et al
EPI_ISL_584075	2020-06-19	The National Institute of Public Health	State Veterinary Institute Prague	Nagy et al
EPI_ISL_558835	2020-06-19	Lighthouse Lab in Alderley Park	Wellcome Sanger Institute for the COVID-19 Genomics UK (COG-UK) consortium	The Lighthouse Lab in Alderley Park et al
EPI_ISL_558791	2020-06-19	Lighthouse Lab in Alderley Park	Wellcome Sanger Institute for the COVID-19 Genomics UK (COG-UK) consortium	The Lighthouse Lab in Alderley Park et al
EPI_ISL_708025	2020-06-19	Virology, Universitätsklinikum des Saarlandes	Epigenetics, Saarland University	Kathrin Kattler et al
EPI_ISL_653909	2020-06-19	Translational Health Science and Technology Institute	National Institute of Biomedical Genomics	Arindam Maitra et al
EPI_ISL_516620	2020-06-19	Instituto de Diagnostico y Referencia Epidemiologicos (INDRE)	Instituto de Diagnostico y Referencia Epidemiologicos (INDRE)	Gisela Barrera-Badillo et al
EPI_ISL_548104	2020-06-19	LabTests	Institute of Environmental Science and Research (ESR)	Xiaoyun Ren et al
EPI_ISL_486506	2020-06-19	Viollier AG	Department of Biosystems Science and Engineering, ETH Zürich	Christian Beisel et al
EPI_ISL_583496	2020-06-20	UPA Jandira	Instituto Adolfo Lutz, Interdiciplinary Procedures Center, Strategic Laboratory	Claudio Tavares Sacchi et al
EPI_ISL_603024	2020-06-20	Santa Casa de Misericordia de Araçatuba	Instituto Adolfo Lutz, Interdiciplinary Procedures Center, Strategic Laboratory	Claudio Tavares Sacchi et al
EPI_ISL_681687	2020-06-20	Molecular Medicine Laboratory, University of Magallanes	Centro Asistencial Docente y de Investigacion, Universidad de Magallanes	Jorge González et al
EPI_ISL_681688	2020-06-20	Molecular Medicine Laboratory, University of Magallanes	Centro Asistencial Docente y de Investigacion, Universidad de Magallanes	Jorge González et al
EPI_ISL_512667	2020-06-20	Area De Salud La Cruz	Inciensa, Instituto Costarricense de Investigación y Enseñanza en Nutrición y Salud	Francisco Duarte et al
EPI_ISL_548130	2020-06-20	Middlemore Hospital	Institute of Environmental Science and Research (ESR)	Xiaoyun Ren et al
EPI_ISL_492065	2020-06-20	Oman-National Influenza Center	Department of Microbiology and Immunology-SQUH Department of Microbiology and Immunology, Sultan Qaboos University Hopsital, P.O 35, Postal code 123	Samira Al-Maruqi et al

EPI_ISL_514937	2020-06-20	Division of Viral Diseases, Center for Laboratory Control of Infectious Diseases, Korea Centers for Diseases Control and Prevention	Division of Viral Diseases, Center for Laboratory Control of Infectious Diseases, Korea Centers for Diseases Control and Prevention	Jeong-Min Kim et al
EPI_ISL_693206	2020-06-21	Hospital Municipal Mario Gatti	Instituto Adolfo Lutz, Interdiciplinary Procedures Center, Strategic Laboratory	Claudio Tavares Sacchi et al
EPI_ISL_583502	2020-06-21	Serv de Vig Sanitaria Epidemio e CTRL de Zoonoses Guaruja	Instituto Adolfo Lutz, Interdiciplinary Procedures Center, Strategic Laboratory	Claudio Tavares Sacchi et al
EPI_ISL_660474	2020-06-21	Laboratoire de Microbiologie CHU Souro Sanou	Centre Muraz	Abdoul-Salam Ouedraogo et al
EPI_ISL_660475	2020-06-21	Laboratoire de Microbiologie CHU Souro Sanou	Centre Muraz	Abdoul-Salam Ouedraogo et al
EPI_ISL_660476	2020-06-21	Laboratoire de Microbiologie CHU Souro Sanou	Centre Muraz	Abdoul-Salam Ouedraogo et al
EPI_ISL_660477	2020-06-21	Laboratoire de Microbiologie CHU Souro Sanou	Centre Muraz	Abdoul-Salam Ouedraogo et al
EPI_ISL_660522	2020-06-21	Laboratoire de Microbiologie CHU Souro Sanou	Centre Muraz	Abdoul-Salam Ouedraogo et al
EPI_ISL_584076	2020-06-21	The National Institute of Public Health	State Veterinary Institute Prague	Nagy et al
EPI_ISL_584077	2020-06-21	The National Institute of Public Health	State Veterinary Institute Prague	Nagy et al
EPI_ISL_649163	2020-06-21	Laboratorio de Investigaciones de Baney	University Hospital Basel, Clinical Bacteriology	Carlos Cortes et al
EPI_ISL_635781	2020-06-21	Biolab Diagnostic Laboratories	Andersen lab at Scripps Research	Issa Abu-Dayyeh et al
EPI_ISL_548132	2020-06-21	Middlemore Hospital	Institute of Environmental Science and Research (ESR)	Xiaoyun Ren et al
EPI_ISL_613460	2020-06-21	Microbiology, Koc University	Microbiology, Koc University	Ozer et al
EPI_ISL_583503	2020-06-22	CTA Centro de Testagem e Aconselhamento	Instituto Adolfo Lutz, Interdiciplinary Procedures Center, Strategic Laboratory	Claudio Tavares Sacchi et al
EPI_ISL_693205	2020-06-22	Hospital de Campanha Covid-19 Assis	Instituto Adolfo Lutz, Interdiciplinary Procedures Center, Strategic Laboratory	Claudio Tavares Sacchi et al
EPI_ISL_552941	2020-06-22	Lighthouse Lab in Alderley Park	Wellcome Sanger Institute for the COVID-19 Genomics UK (COG-UK) consortium	The Lighthouse Lab in Alderley Park et al
EPI_ISL_610210	2020-06-22	Department of Health Technology and Informatics, The Hong Kong Polytechnic University	Department of Health Technology and Informatics, The Hong Kong Polytechnic University	Siu et al
EPI_ISL_497770	2020-06-22	Department of Microbiology, The University of Hong Kong	Department of Microbiology, The University of Hong Kong	Kelvin K.W. To et al
EPI_ISL_568872	2020-06-22	KEMRI-Wellcome Trust Research Programme/KEMRI-CGMR-C Kilifi	KEMRI-Wellcome Trust Research Programme/KEMRI-CGMR-C Kilifi	Githinji et al 2020 et al
EPI_ISL_579496	2020-06-22	Canterbury Health Laboratories	Institute of Environmental Science and Research (ESR)	Xiaoyun Ren et al
EPI_ISL_493352	2020-06-22	Oslo University Hospital, Department of Medical Microbiology	Norwegian Institute of Public Health, Department of Virology	Kathrine Stene-Johansen et al
EPI_ISL_596559	2020-06-22	Palestinian Ministry of Health	Molecular Genetics Lab	Nouar Qutob et al
EPI_ISL_596563	2020-06-22	Palestinian Ministry of Health	Molecular Genetics Lab	Nouar Qutob et al
EPI_ISL_540959	2020-06-22	Laboratorio de Referencia Nacional de Virus Respiratorios, Instituto Nacional de Salud Peru	Laboratorio de Genómica Microbiana, Universidad Peruana Cayetano Heredia	Pablo Tsukayama et al
EPI_ISL_491474	2020-06-22	Research Institute for Tropical Medicine	Research Institute for Tropical Medicine	Ma. Angelica Tujan et al
EPI_ISL_477180	2020-06-22	Department of Laboratory Medicine Tan Tock Seng Hospital	Department of Laboratory Medicine Tan Tock Seng Hospital	Chen YYC et al
EPI_ISL_514947	2020-06-22	Division of Viral Diseases, Center for Laboratory Control of Infectious Diseases, Korea Centers for Diseases Control and Prevention	Division of Viral Diseases, Center for Laboratory Control of Infectious Diseases, Korea Centers for Diseases Control and Prevention	Jeong-Min Kim et al
EPI_ISL_486887	2020-06-23	National Influenza Center, Bahrain	National Influenza Center, Bahrain	Zaed et al
EPI_ISL_510528	2020-06-23	Communicable Disease Laboratory, Public Health Directorate	Communicable Disease Laboratory, Public Health Directorate	Al Wasti et al
EPI_ISL_510531	2020-06-23	Communicable Disease Laboratory, Public Health Directorate	Communicable Disease Laboratory, Public Health Directorate	Shehab et al
EPI_ISL_485401	2020-06-23	Communicable Disease Laboratory, Public Health Directorate	Communicable Disease Laboratory, Public Health Directorate	Zaed et al
EPI_ISL_681690	2020-06-23	Molecular Medicine Laboratory, University of Magallanes	Centro Asistencial Docente y de Investigacion, Universidad de Magallanes	Jorge González et al
EPI_ISL_681691	2020-06-23	Molecular Medicine Laboratory, University of Magallanes	Centro Asistencial Docente y de Investigacion, Universidad de Magallanes	Jorge González et al

EPI_ISL_681689	2020-06-23	Molecular Medicine Laboratory, University of Magallanes	Centro Asistencial Docente y de Investigacion, Universidad de Magallanes	Jorge González et al
EPI_ISL_552924	2020-06-23	Lighthouse Lab in Alderley Park	Wellcome Sanger Institute for the COVID-19 Genomics UK (COG-UK) consortium	The Lighthouse Lab in Alderley Park et al
EPI_ISL_610211	2020-06-23	Department of Health Technology and Informatics, The Hong Kong Polytechnic University	Department of Health Technology and Informatics, The Hong Kong Polytechnic University	Siu et al
EPI_ISL_596543	2020-06-23	Palestinian Ministry of Health	Molecular Genetics Lab	Nouar Qutob et al
EPI_ISL_512890	2020-06-23	Pathogen Genomics Lab King Abdullah University of Science and Technology(KAUST)	Pathogen Genomics Lab King Abdullah University of Science and Technology(KAUST)	Raeece Naeem et al
EPI_ISL_636966	2020-06-23	Pathogen Genomics Lab King Abdullah University of Science and Technology(KAUST)	Pathogen Genomics Lab King Abdullah University of Science and Technology(KAUST)	Fathia Ben Rached et al
EPI_ISL_512906	2020-06-23	Pathogen Genomics Lab King Abdullah University of Science and Technology(KAUST)	Pathogen Genomics Lab King Abdullah University of Science and Technology(KAUST)	Fathia Ben Rached et al
EPI_ISL_513072	2020-06-23	Pathogen Genomics Lab King Abdullah University of Science and Technology(KAUST)	Pathogen Genomics Lab King Abdullah University of Science and Technology(KAUST)	Raeece Naeem et al
EPI_ISL_541010	2020-06-23	Servicio de Microbiología, Hospital Miguel Servet, Zaragoza	SeqCOVID-SPAIN consortium/Institute of Biomedicine of Valencia, IBV-CSIC	Antonio Rezusta López et al
EPI_ISL_541011	2020-06-23	Servicio de Microbiología, Hospital Miguel Servet, Zaragoza	SeqCOVID-SPAIN consortium/Institute of Biomedicine of Valencia, IBV-CSIC	Antonio Rezusta López et al
EPI_ISL_512597	2020-06-23	National Laboratory for Influenza/Virology reference laboratory, Public Health Center of the Ministry of Health of Ukraine	Respiratory Virus Unit, Microbiology Services Colindale, Public Health England	PHE Covid Sequencing Team et al
EPI_ISL_512599	2020-06-23	National Laboratory for Influenza/Virology reference laboratory, Public Health Center of the Ministry of Health of Ukraine	Respiratory Virus Unit, Microbiology Services Colindale, Public Health England	PHE Covid Sequencing Team et al
EPI_ISL_512601	2020-06-23	National Laboratory for Influenza/Virology reference laboratory, Public Health Center of the Ministry of Health of Ukraine	Respiratory Virus Unit, Microbiology Services Colindale, Public Health England	PHE Covid Sequencing Team et al
EPI_ISL_502779	2020-06-24	LACEN/PE	LABBE, Federal University of Pernambuco	WILSON JOSE DA SILVA JUNIOR et al
EPI_ISL_502875	2020-06-24	LACEN/PE	LABBE, Federal University of Pernambuco	WILSON JOSE DA SILVA JUNIOR et al
EPI_ISL_581491	2020-06-24	Fondation Congolaise pour la recherche medicale (FCRM)	NGS Competence Center Tübingen, Institut für Medizinische Mikrobiologie und Hygiene, Universitätsklinikum Tübingen	Angel Angelov et al
EPI_ISL_614372	2020-06-24	Molecular diagnostic unit for viral haemorrhagic fevers and emerging viruses, Bouaké CHU Laboratory	Project group Epidemology of Highly Pathogenic Microorganisms, Robert Koch-Institute	Chantal Akoua-Koffi et al
EPI_ISL_635540	2020-06-24	Centro de Diagn√≥stico COVID-19 UABC Tijuana	Andersen lab at Scripps Research	SEARCH Alliance San Diego with Idanya Rubí Serafín Higuera et al
EPI_ISL_676584	2020-06-24	Scientific Veterinary Institute Novi Sad	Veterinary Specialized Institute "Kraljevo", Serbia	Vidanovic et al
EPI_ISL_661184	2020-06-24	Scientific Veterinary Institute Novi Sad	Veterinary Specialized Institute "Kraljevo", Serbia	Vidanovic et al
EPI_ISL_514961	2020-06-24	Division of Viral Diseases, Center for Laboratory Control of Infectious Diseases, Korea Centers for Diseases Control and Prevention	Division of Viral Diseases, Center for Laboratory Control of Infectious Diseases, Korea Centers for Diseases Control and Prevention	Jeong-Min Kim et al
EPI_ISL_541014	2020-06-24	Servicio de Microbiología, Hospital Miguel Servet, Zaragoza	SeqCOVID-SPAIN consortium/Institute of Biomedicine of Valencia, IBV-CSIC	Antonio Rezusta López et al
EPI_ISL_486531	2020-06-24	Viollier AG	Department of Biosystems Science and Engineering, ETH Zürich	Christian Beisel et al
EPI_ISL_512612	2020-06-24	National Laboratory for Influenza/Virology reference laboratory, Public Health Center of the Ministry of Health of Ukraine	Respiratory Virus Unit, Microbiology Services Colindale, Public Health England	PHE Covid Sequencing Team et al
EPI_ISL_487273	2020-06-25	unknown	Communicable Disease Laboratory, Public Health Directorate	Zaed et al
EPI_ISL_581493	2020-06-25	Fondation Congolaise pour la recherche medicale (FCRM)	NGS Competence Center Tübingen, Institut für Medizinische Mikrobiologie und Hygiene, Universitätsklinikum Tübingen	Angel Angelov et al
EPI_ISL_648370	2020-06-25	Laboratorio de Investigaciones de Baney	University Hospital Basel, Clinical Bacteriology	Carlos Cortes et al
EPI_ISL_560637	2020-06-25	Labo Analyses Med	National Reference Center for Viruses of Respiratory Infections, Institut Pasteur, Paris	Sylvie Behillil et al
EPI_ISL_568997	2020-06-25	MEPHI, Aix Marseille University	MEPHI, Aix Marseille University	Anthony LEVASSEUR et al

EPI_ISL_573152	2020-06-25	Institute for Virology, University Hospital Duesseldorf, Medical Faculty, Heinrich-Heine-University Duesseldorf	Institute for Virology, University Hospital Duesseldorf, Medical Faculty, Heinrich-Heine-University Duesseldorf	Maximilian Damagnez et al
EPI_ISL_635777	2020-06-25	Biolab Diagnostic Laboratories	Andersen lab at Scripps Research	Issa Abu-Dayyeh et al
EPI_ISL_635545	2020-06-25	Centro de Diagn√≥stico COVID-19 UABC Tijuana	Andersen lab at Scripps Research	SEARCH Alliance San Diego with Idanya Rubí Serafín Higuera et al
EPI_ISL_523734	2020-06-25	Dutch COVID-19 response team	Erasmus Medical Center	Bas Oude Munnink et al
EPI_ISL_500778	2020-06-25	Foerde Hospital, Department of Microbiology	Norwegian Institute of Public Health, Department of Virology	Kathrine Stene-Johansen et al
EPI_ISL_491298	2020-06-25	Rural Health Unit - Calauan, Laguna	Research Institute for Tropical Medicine	Tujan et al
EPI_ISL_678091	2020-06-25	Pathogen Genomics Lab King Abdullah University of Science and Technology(KAUST)	Pathogen Genomics Lab King Abdullah University of Science and Technology(KAUST)	Muhammad Shuaib et al
EPI_ISL_661188	2020-06-25	Scientific Veterinary Institute Novi Sad	Veterinary Specialized Institute "Kraljevo", Serbia	Vidanovic et al
EPI_ISL_495526	2020-06-25	NHLS-IALCH	KRISP, KZN Research Innovation and Sequencing Platform	Giandhari J et al
EPI_ISL_522463	2020-06-25	Center for Laboratory Control of Infectious Diseases, Korea Centers for Diseases Control and Prevention	Center for Laboratory Control of Infectious Diseases, Korea Centers for Diseases Control and Prevention	Junyoung Kim et al
EPI_ISL_517626	2020-06-25	Academic Hospital Paramaribo	Erasmus Medical Center	Bas Oude Munnink et al
EPI_ISL_517643	2020-06-25	Academic Hospital Paramaribo	Erasmus Medical Center	Bas Oude Munnink et al
EPI_ISL_486528	2020-06-25	Viollier AG	Department of Biosystems Science and Engineering, ETH Zürich	Christian Beisel et al
EPI_ISL_653757	2020-06-26	Instituto Nacional de Salud, Bogotá, Colombia	Instituto Nacional de Salud, Bogotá, Colombia	Katherine Laiton-Donato et al
EPI_ISL_594188	2020-06-26	Department of Pathology, School of Medicine, Imam Khomeini Hospital, Tehran University of Medical Sciences	Genetics Research Center, University of Social Welfare and Rehabilitation Sciences	Zohreh Fattahi et al
EPI_ISL_730021	2020-06-26	Nigeria Centre for Disease Control (NCDC)	African Centre of Excellence for Genomics of Infectious Diseases (ACEGID), Redeemer's University, Ede, Osun State, Nigeria	Oluniyi P.E. et al et al
EPI_ISL_693567	2020-06-26	Instituto Nacional de Saude (INSA)	Instituto Nacional de Saude (INSA)	Borges et al et al
EPI_ISL_569776	2020-06-26	Omsk Research Institute of Natural Focal Infections	WHO National Influenza Centre Russian Federation	Artem Fadeev et al
EPI_ISL_639943	2020-06-26	Omsk Research Institute of Natural Focal Infections	WHO National Influenza Centre Russian Federation	Artem Fadeev et al
EPI_ISL_517647	2020-06-26	Academic Hospital Paramaribo	Erasmus Medical Center	Bas Oude Munnink et al
EPI_ISL_517648	2020-06-26	Academic Hospital Paramaribo	Erasmus Medical Center	Bas Oude Munnink et al
EPI_ISL_517650	2020-06-26	Academic Hospital Paramaribo	Erasmus Medical Center	Bas Oude Munnink et al
EPI_ISL_517651	2020-06-26	Academic Hospital Paramaribo	Erasmus Medical Center	Bas Oude Munnink et al
EPI_ISL_517652	2020-06-26	Academic Hospital Paramaribo	Erasmus Medical Center	Bas Oude Munnink et al
EPI_ISL_517653	2020-06-26	Academic Hospital Paramaribo	Erasmus Medical Center	Bas Oude Munnink et al
EPI_ISL_486521	2020-06-26	Viollier AG	Department of Biosystems Science and Engineering, ETH Zürich	Christian Beisel et al
EPI_ISL_512613	2020-06-26	National Laboratory for Influenza/Virology reference laboratory, Public Health Center of the Ministry of Health of Ukraine	Respiratory Virus Unit, Microbiology Services Colindale, Public Health England	PHE Covid Sequencing Team et al
EPI_ISL_699057	2020-06-26	Group 42 (G42) Healthcare, Abu Dhabi, United Arab Emirates; Department of Health, The United Arab Emirates	G42 Healthcare	Rong Liu et al
EPI_ISL_693231	2020-06-27	Pronto Socorro Municipal de Santa Branca	Instituto Adolfo Lutz, Interdiciplinary Procedures Center, Strategic Laboratory	Claudio Tavares Sacchi et al
EPI_ISL_729968	2020-06-27	Nigeria Centre for Disease Control (NCDC)	African Centre of Excellence for Genomics of Infectious Diseases (ACEGID), Redeemer's University, Ede, Osun State, Nigeria	Oluniyi P.E. et al et al
EPI_ISL_516427	2020-06-27	Clinical Hospital - Shtip	Research Center for Genetic Engineering and Biotechnology "Georgi D. Efremov" , Macedonian Academy of Sciences and Arts	RCGEB - MASA et al
EPI_ISL_699081	2020-06-27	Group 42 (G42) Healthcare, Abu Dhabi, United Arab Emirates; Department of Health, The United Arab Emirates	G42 Healthcare	Rong Liu et al
EPI_ISL_548133	2020-06-28	Middlemore Hospital	Institute of Environmental Science and Research (ESR)	Xiaoyun Ren et al
EPI_ISL_596545	2020-06-28	Palestinian Ministry of Health	Molecular Genetics Lab	Nouar Qutob et al
EPI_ISL_596549	2020-06-28	Palestinian Ministry of Health	Molecular Genetics Lab	Nouar Qutob et al

EPI_ISL_522105	2020-06-29	Victorian Infectious Diseases Reference Laboratory (VIDRL)	VIDRL and MDU-PHL	Caly L. et al
EPI_ISL_717787	2020-06-29	LACEN RJ - Noel Nutels	Bioinformatics Laboratory / LNCC	Carolina M Voloch et al
EPI_ISL_717797	2020-06-29	LACEN RJ - Noel Nutels	Bioinformatics Laboratory / LNCC	Carolina M Voloch et al
EPI_ISL_583500	2020-06-29	Centro de Saude I Tacito Leite de Carvalho e Silva	Instituto Adolfo Lutz, Interdiciplinary Procedures Center, Strategic Laboratory	Claudio Tavares Sacchi et al
EPI_ISL_708529	2020-06-29	Secretária Municipal de Saude de Fernandópolis	Instituto Adolfo Lutz, Interdiciplinary Procedures Center, Strategic Laboratory	Claudio Tavares Sacchi et al
EPI_ISL_681833	2020-06-29	Molecular diagnostic unit for viral haemorrhagic fevers and emerging viruses, Bouaké CHU Laboratory	Project group Epidemology of Highly Pathogenic Microorganisms, Robert Koch-Institute	Chantal Akoua-Koffi et al
EPI_ISL_560640	2020-06-29	Labo Analyses Med	National Reference Center for Viruses of Respiratory Infections, Institut Pasteur, Paris	Sylvie Behillil et al
EPI_ISL_516901	2020-06-29	Israel Central Virology laboratory	Israel Central Virology laboratory	Neta Zuckerman et al
EPI_ISL_516905	2020-06-29	Israel Central Virology laboratory	Israel Central Virology laboratory	Neta Zuckerman et al
EPI_ISL_493377	2020-06-29	Furst Medical Laboratory	Norwegian Institute of Public Health, Department of Virology	Kathrine Stene-Johansen et al
EPI_ISL_500776	2020-06-29	Foerde Hospital, Department of Microbiology	Norwegian Institute of Public Health, Department of Virology	Kathrine Stene-Johansen et al
EPI_ISL_500777	2020-06-29	Foerde Hospital, Department of Microbiology	Norwegian Institute of Public Health, Department of Virology	Kathrine Stene-Johansen et al
EPI_ISL_514209	2020-06-29	Florida Bureau of Public Health Laboratories	Florida Bureau of Public Health Laboratories	Sarah Schmedes et al
EPI_ISL_583883	2020-06-30	Austrian Agency for Health and Food Safety (AGES)	Bergthaler laboratory, CeMM Research Center for Molecular Medicine of the Austrian Academy of Sciences	Alexandra Popa et al
EPI_ISL_583884	2020-06-30	Austrian Agency for Health and Food Safety (AGES)	Bergthaler laboratory, CeMM Research Center for Molecular Medicine of the Austrian Academy of Sciences	Alexandra Popa et al
EPI_ISL_603028	2020-06-30	Hospital Municipal Santa Ana	Instituto Adolfo Lutz, Interdiciplinary Procedures Center, Strategic Laboratory	Claudio Tavares Sacchi et al
EPI_ISL_515525	2020-06-30	National Influenza Center - Instituto Adolfo Lutz	Instituto Adolfo Lutz, Interdiciplinary Procedures Center, Strategic Laboratory	Claudio Tavares Sacchi et al
EPI_ISL_693234	2020-06-30	Upa Vereador Jose Da Rocha Goncalves	Instituto Adolfo Lutz, Interdiciplinary Procedures Center, Strategic Laboratory	Claudio Tavares Sacchi et al
EPI_ISL_693238	2020-06-30	Secao Centro de Diagnostico Secedi	Instituto Adolfo Lutz, Interdiciplinary Procedures Center, Strategic Laboratory	Claudio Tavares Sacchi et al
EPI_ISL_653755	2020-06-30	Instituto Nacional de Salud, Bogotá, Colombia	Instituto Nacional de Salud, Bogotá, Colombia	Katherine Laiton-Donato et al
EPI_ISL_486845	2020-06-30	Institute of Microbiology, Universidad San Francisco de Quito	Institute of Microbiology, Universidad San Francisco de Quito	Belén Prado-Vivar et al
EPI_ISL_486846	2020-06-30	Institute of Microbiology, Universidad San Francisco de Quito	Institute of Microbiology, Universidad San Francisco de Quito	Belén Prado-Vivar et al
EPI_ISL_486847	2020-06-30	Institute of Microbiology, Universidad San Francisco de Quito	Institute of Microbiology, Universidad San Francisco de Quito	Belén Prado-Vivar et al
EPI_ISL_486849	2020-06-30	Institute of Microbiology, Universidad San Francisco de Quito	Institute of Microbiology, Universidad San Francisco de Quito	Belén Prado-Vivar et al
EPI_ISL_486850	2020-06-30	Institute of Microbiology, Universidad San Francisco de Quito	Institute of Microbiology, Universidad San Francisco de Quito	Belén Prado-Vivar et al
EPI_ISL_486851	2020-06-30	Institute of Microbiology, Universidad San Francisco de Quito	Institute of Microbiology, Universidad San Francisco de Quito	Belén Prado-Vivar et al
EPI_ISL_491936	2020-06-30	Institute of Microbiology, Universidad San Francisco de Quito	Institute of Microbiology, Universidad San Francisco de Quito	Belén Prado-Vivar et al
EPI_ISL_491937	2020-06-30	Institute of Microbiology, Universidad San Francisco de Quito	Institute of Microbiology, Universidad San Francisco de Quito	Belén Prado-Vivar et al
EPI_ISL_561213	2020-06-30	MRCG at LSHTM Genomics lab	MRCG at LSHTM Genomics lab	Abdul Karim sesay et al
EPI_ISL_561214	2020-06-30	MRCG at LSHTM Genomics lab	MRCG at LSHTM Genomics lab	Abdul Karim sesay et al
EPI_ISL_561216	2020-06-30	MRCG at LSHTM Genomics lab	MRCG at LSHTM Genomics lab	Abdul Karim sesay et al
EPI_ISL_561217	2020-06-30	MRCG at LSHTM Genomics lab	MRCG at LSHTM Genomics lab	Abdul Karim sesay et al
EPI_ISL_561218	2020-06-30	MRCG at LSHTM Genomics lab	MRCG at LSHTM Genomics lab	Abdul Karim sesay et al
EPI_ISL_582030	2020-06-30	Biology Department, College of Science, Al-Muthanna University	International Centre for Genetic Engineering and Biotechnology (ICGEB) and ARGO Open Lab Platform	Nihad Al-Rashedi et al
EPI_ISL_644954	2020-06-30	Department of Infectious Diseases, Keio University School of Medicine, Tokyo, Japan	Center for Medical Genetics, Keio University School of Medicine, Tokyo, Japan	Kenjiro Kosaki et al

EPI_ISL_577740	2020-06-30	Institute of Virology, Biomedical Research Center of the Slovak Academy of Sciences, Bratislava	Faculty of Natural Sciences, Comenius University, Bratislava	Broňa Brejová et al
EPI_ISL_577741	2020-06-30	Institute of Virology, Biomedical Research Center of the Slovak Academy of Sciences, Bratislava	Faculty of Natural Sciences, Comenius University, Bratislava	Broňa Brejová et al
EPI_ISL_522467	2020-06-30	Center for Laboratory Control of Infectious Diseases, Korea Centers for Diseases Control and Prevention	Center for Laboratory Control of Infectious Diseases, Korea Centers for Diseases Control and Prevention	Junyoung Kim et al
EPI_ISL_717855	2020-07-01	Laboratorio de Virologia Molecular / UFRJ	Bioinformatics Laboratory / LNCC	Carolina M Voloch et al
EPI_ISL_693208	2020-07-01	Hospital Municipal Antonio Giglio	Instituto Adolfo Lutz, Interdiciplinary Procedures Center, Strategic Laboratory	Claudio Tavares Sacchi et al
EPI_ISL_693209	2020-07-01	Hospital Municipal Antonio Giglio	Instituto Adolfo Lutz, Interdiciplinary Procedures Center, Strategic Laboratory	Claudio Tavares Sacchi et al
EPI_ISL_693237	2020-07-01	UPA Santa Isabel	Instituto Adolfo Lutz, Interdiciplinary Procedures Center, Strategic Laboratory	Claudio Tavares Sacchi et al
EPI_ISL_682236	2020-07-01	AREA DE SALUD LOS CHILES	Inciensa, Instituto Costarricense de Investigación y Enseñanza en Nutrición y Salud	Francisco Duarte et al
EPI_ISL_486842	2020-07-01	Institute of Microbiology, Universidad San Francisco de Quito	Institute of Microbiology, Universidad San Francisco de Quito	Belén Prado-Vivar et al
EPI_ISL_486843	2020-07-01	Institute of Microbiology, Universidad San Francisco de Quito	Institute of Microbiology, Universidad San Francisco de Quito	Belén Prado-Vivar et al
EPI_ISL_486844	2020-07-01	Institute of Microbiology, Universidad San Francisco de Quito	Institute of Microbiology, Universidad San Francisco de Quito	Belén Prado-Vivar et al
EPI_ISL_648322	2020-07-01	Laboratorio de Investigaciones de Baney	University Hospital Basel, Clinical Bacteriology	Carlos Cortes et al
EPI_ISL_561222	2020-07-01	MRCG at LSHTM Genomics lab	MRCG at LSHTM Genomics lab	Abdul Karim sesay et al
EPI_ISL_729563	2020-07-01	A. Krumbholz, Labor Dr. Krause und Kollegen MVZ GmbH, Kiel	Charité Universitätsmedizin Berlin, Institut für Virologie	Victor M Corman et al
EPI_ISL_501262	2020-07-01	National Virus Reference Laboratory	National Virus Reference Laboratory	Michael Carr et al
EPI_ISL_547920	2020-07-01	Laboratorio de Infecciones Respiratorias Agudas. Centro Nacional de Salud Publica, Instituto Nacional de Salud	Laboratorio de Infecciones Respiratorias Agudas. Centro Nacional de Salud Publica, Instituto Nacional de Salud	Juscamayta et al
EPI_ISL_547937	2020-07-01	Laboratorio de Infecciones Respiratorias Agudas. Centro Nacional de Salud Publica, Instituto Nacional de Salud	Laboratorio de Infecciones Respiratorias Agudas. Centro Nacional de Salud Publica, Instituto Nacional de Salud	Juscamayta et al
EPI_ISL_490057	2020-07-01	National Public Health Laboratory, National Centre for Infectious Diseases	National Public Health Laboratory, National Centre for Infectious Diseases	Mak TM et al
EPI_ISL_534235	2020-07-01	Karolinska universitetslaboratoriet SOLNA	The Public Health Agency of Sweden	Anna-Malin Linde et al
EPI_ISL_648173	2020-07-01	Klinisk mikrobiologi NAL Trollhattan	The Public Health Agency of Sweden	Anna-Malin Linde et al
EPI_ISL_528707	2020-07-01	Alsafar - Khalifa University Abu Dhabi	Alsafar - Khalifa University Abu Dhabi	Andreas Henschel et al
EPI_ISL_717856	2020-07-02	Laboratorio de Virologia Molecular / UFRJ	Bioinformatics Laboratory / LNCC	Carolina M Voloch et al
EPI_ISL_717858	2020-07-02	Laboratorio de Virologia Molecular / UFRJ	Bioinformatics Laboratory / LNCC	Carolina M Voloch et al
EPI_ISL_693236	2020-07-02	Hospital Santa Marcelina Sao Paulo	Instituto Adolfo Lutz, Interdiciplinary Procedures Center, Strategic Laboratory	Claudio Tavares Sacchi et al
EPI_ISL_693245	2020-07-02	UPA Santa Isabel	Instituto Adolfo Lutz, Interdiciplinary Procedures Center, Strategic Laboratory	Claudio Tavares Sacchi et al
EPI_ISL_512672	2020-07-02	Hospital De Niños Dr. Carlos Saenz Herrera [San Jose/San Jose]	Inciensa, Instituto Costarricense de Investigación y Enseñanza en Nutrición y Salud	Francisco Duarte et al
EPI_ISL_512674	2020-07-02	Hospital De Niños Dr. Carlos Saenz Herrera [San Jose/San Jose]	Inciensa, Instituto Costarricense de Investigación y Enseñanza en Nutrición y Salud	Francisco Duarte et al
EPI_ISL_527750	2020-07-02	Hospital De Niños Dr. Carlos Saenz Herrera [San Jose/San Jose]	Inciensa, Instituto Costarricense de Investigación y Enseñanza en Nutrición y Salud	Francisco Duarte et al
EPI_ISL_614375	2020-07-02	Molecular diagnostic unit for viral haemorrhagic fevers and emerging viruses, Bouaké CHU Laboratory	Project group Epidemology of Highly Pathogenic Microorganisms, Robert Koch-Institute	Chantal Akoua-Koffi et al
EPI_ISL_558200	2020-07-02	Lighthouse Lab in Alderley Park	Wellcome Sanger Institute for the COVID-19 Genomics UK (COG-UK) consortium	The Lighthouse Lab in Alderley Park et al
EPI_ISL_557882	2020-07-02	Lighthouse Lab in Alderley Park	Wellcome Sanger Institute for the COVID-19 Genomics UK (COG-UK) consortium	The Lighthouse Lab in Alderley Park et al
EPI_ISL_648373	2020-07-02	Laboratorio de Investigaciones de Baney	University Hospital Basel, Clinical Bacteriology	Carlos Cortes et al

EPI_ISL_632359	2020-07-02	Dutch COVID-19 response team	Erasmus Medical Center	Bas Oude Munnink et al
EPI_ISL_523513	2020-07-02	Dutch COVID-19 response team	Erasmus Medical Center	Bas Oude Munnink et al
EPI_ISL_500773	2020-07-02	Furst Medical Laboratory	Norwegian Institute of Public Health, Department of Virology	Kathrine Stene-Johansen et al
EPI_ISL_500779	2020-07-02	Akershus University Hospital, Department for Microbiology and Infectious Disease Control	Norwegian Institute of Public Health, Department of Virology	Kathrine Stene-Johansen et al
EPI_ISL_639919	2020-07-02	Omsk Research Institute of Natural Focal Infections	WHO National Influenza Centre Russian Federation	Artem Fadeev et al
EPI_ISL_639920	2020-07-02	Omsk Research Institute of Natural Focal Infections	WHO National Influenza Centre Russian Federation	Artem Fadeev et al
EPI_ISL_678094	2020-07-02	Pathogen Genomics Lab King Abdullah University of Science and Technology(KAUST)	Pathogen Genomics Lab King Abdullah University of Science and Technology(KAUST)	Olga Douvropoulou et al
EPI_ISL_678095	2020-07-02	Pathogen Genomics Lab King Abdullah University of Science and Technology(KAUST)	Pathogen Genomics Lab King Abdullah University of Science and Technology(KAUST)	Olga Douvropoulou et al
EPI_ISL_581492	2020-07-03	Fondation Congolaise pour la recherche medicale (FCRM)	NGS Competence Center Tübingen, Institut für Medizinische Mikrobiologie und Hygiene, Universitätsklinikum Tübingen	Angel Angelov et al
EPI_ISL_527746	2020-07-03	Area De Salud La Cruz	Inciensa, Instituto Costarricense de Investigación y Enseñanza en Nutrición y Salud	Francisco Duarte et al
EPI_ISL_681836	2020-07-03	Molecular diagnostic unit for viral haemorrhagic fevers and emerging viruses, Bouaké CHU Laboratory	Project group Epidemology of Highly Pathogenic Microorganisms, Robert Koch-Institute	Chantal Akoua-Koffi et al
EPI_ISL_491932	2020-07-03	Institute of Microbiology, Universidad San Francisco de Quito	Institute of Microbiology, Universidad San Francisco de Quito	Belén Prado-Vivar et al
EPI_ISL_491939	2020-07-03	Institute of Microbiology, Universidad San Francisco de Quito	Institute of Microbiology, Universidad San Francisco de Quito	Belén Prado-Vivar et al
EPI_ISL_491940	2020-07-03	Institute of Microbiology, Universidad San Francisco de Quito	Institute of Microbiology, Universidad San Francisco de Quito	Belén Prado-Vivar et al
EPI_ISL_516910	2020-07-03	Israel Central Virology laboratory	Israel Central Virology laboratory	Neta Zuckerman et al
EPI_ISL_500769	2020-07-03	Furst Medical Laboratory	Norwegian Institute of Public Health, Department of Virology	Kathrine Stene-Johansen et al
EPI_ISL_536477	2020-07-03	Instituto Nacional de Salud	Laboratorio de Infecciones Respiratorias Agudas	Eduardo Juscamayta Lopez et al
EPI_ISL_547917	2020-07-03	Laboratorio de Infecciones Respiratorias Agudas. Centro Nacional de Salud Publica, Instituto Nacional de Salud	Laboratorio de Infecciones Respiratorias Agudas. Centro Nacional de Salud Publica, Instituto Nacional de Salud	Juscamayta et al
EPI_ISL_547931	2020-07-03	Laboratorio de Infecciones Respiratorias Agudas. Centro Nacional de Salud Publica, Instituto Nacional de Salud	Laboratorio de Infecciones Respiratorias Agudas. Centro Nacional de Salud Publica, Instituto Nacional de Salud	Juscamayta et al
EPI_ISL_693598	2020-07-03	Instituto Nacional de Saude (INSA)	Instituto Nacional de Saude (INSA)	Borges et al et al
EPI_ISL_541066	2020-07-03	Hospital Clínico Universitario de Santiago de Compostela	SeqCOVID-SPAIN consortium/Institute of Biomedicine of Valencia, IBV-CSIC	José Javier Costa Alcalde et al
EPI_ISL_500877	2020-07-03	Viollier AG	Department of Biosystems Science and Engineering, ETH Zürich	Christian Beisel et al
EPI_ISL_498619	2020-07-03	Viollier AG	Department of Biosystems Science and Engineering, ETH Zürich	Christian Beisel et al
EPI_ISL_581487	2020-07-04	Fondation Congolaise pour la recherche medicale (FCRM)	NGS Competence Center Tübingen, Institut für Medizinische Mikrobiologie und Hygiene, Universitätsklinikum Tübingen	Angel Angelov et al
EPI_ISL_561230	2020-07-04	MRCG at LSHTM Genomics lab	MRCG at LSHTM Genomics lab	Abdul Karim sesay et al
EPI_ISL_610178	2020-07-04	Department of Health Technology and Informatics, The Hong Kong Polytechnic University	Department of Health Technology and Informatics, The Hong Kong Polytechnic University	Siu et al
EPI_ISL_495025	2020-07-04	Government Medical College, Bhavnagar	Gujarat Biotechnology Research Centre	Dinesh Kumar et al
EPI_ISL_635778	2020-07-04	Biolab Diagnostic Laboratories	Andersen lab at Scripps Research	Issa Abu-Dayyeh et al
EPI_ISL_635782	2020-07-04	Biolab Diagnostic Laboratories	Andersen lab at Scripps Research	Issa Abu-Dayyeh et al
EPI_ISL_635560	2020-07-04	Centro de Diagn√≥stico COVID-19 UABC Tijuana	Andersen lab at Scripps Research	SEARCH Alliance San Diego with Idanya Rubí Serafín Higuera et al
EPI_ISL_635562	2020-07-04	Centro de Diagn√≥stico COVID-19 UABC Tijuana	Andersen lab at Scripps Research	SEARCH Alliance San Diego with Idanya Rubí Serafín Higuera et al
EPI_ISL_523645	2020-07-04	Dutch COVID-19 response team	Erasmus Medical Center	Bas Oude Munnink et al
EPI_ISL_729989	2020-07-04	Nigeria Centre for Disease Control (NCDC)	African Centre of Excellence for Genomics of Infectious Diseases (ACEGID), Redeemer's University, Ede, Osun State, Nigeria	Oluniyi P.E. et al et al
EPI_ISL_496860	2020-07-04	Gorgas Memorial Laboratory of Health Studies	Gorgas Memorial Laboratory of Health Studies	Danilo Franco et al

EPI_ISL_496861	2020-07-04	Gorgas Memorial Laboratory of Health Studies	Gorgas Memorial Laboratory of Health Studies	Danilo Franco et al
EPI_ISL_496862	2020-07-04	Gorgas Memorial Laboratory of Health Studies	Gorgas Memorial Laboratory of Health Studies	Danilo Franco et al
EPI_ISL_496863	2020-07-04	Gorgas Memorial Laboratory of Health Studies	Gorgas Memorial Laboratory of Health Studies	Danilo Franco et al
EPI_ISL_536485	2020-07-04	Instituto Nacional de Salud	Laboratorio de Infecciones Respiratorias Agudas	Eduardo Juscamayta Lopez et al
EPI_ISL_536492	2020-07-04	Instituto Nacional de Salud	Laboratorio de Infecciones Respiratorias Agudas	Eduardo Juscamayta Lopez et al
EPI_ISL_536498	2020-07-04	Instituto Nacional de Salud	Laboratorio de Infecciones Respiratorias Agudas	Eduardo Juscamayta Lopez et al
EPI_ISL_536499	2020-07-04	Instituto Nacional de Salud	Laboratorio de Infecciones Respiratorias Agudas	Eduardo Juscamayta Lopez et al
EPI_ISL_547938	2020-07-04	Laboratorio de Infecciones Respiratorias Agudas. Centro Nacional de Salud Publica, Instituto Nacional de Salud	Laboratorio de Infecciones Respiratorias Agudas. Centro Nacional de Salud Publica, Instituto Nacional de Salud	Juscamayta et al
EPI_ISL_522474	2020-07-04	Division of Viral Diseases, Center for Laboratory Control of Infectious Diseases, Korea Centers for Diseases Control and Prevention	Division of Viral Diseases, Center for Laboratory Control of Infectious Diseases, Korea Centers for Diseases Control and Prevention	Jeong-Min Kim et al
EPI_ISL_522475	2020-07-04	Division of Viral Diseases, Center for Laboratory Control of Infectious Diseases, Korea Centers for Diseases Control and Prevention	Division of Viral Diseases, Center for Laboratory Control of Infectious Diseases, Korea Centers for Diseases Control and Prevention	Jeong-Min Kim et al
EPI_ISL_541038	2020-07-04	Hospital Clínico Universitario de Santiago de Compostela	SeqCOVID-SPAIN consortium/Institute of Biomedicine of Valencia, IBV-CSIC	José Javier Costa Alcalde et al
EPI_ISL_520762	2020-07-05	Victorian Infectious Diseases Reference Laboratory (VIDRL)	VIDRL and MDU-PHL	Caly L. et al
EPI_ISL_632261	2020-07-05	Communicable Disease Laboratory, Public Health Directorate	Communicable Disease Laboratory, Public Health Directorate	AlWasti et al
EPI_ISL_693216	2020-07-05	Unidade de Vigilância Epiudemiológica de Araras	Instituto Adolfo Lutz, Interdiciplinary Procedures Center, Strategic Laboratory	Claudio Tavares Sacchi et al
EPI_ISL_693217	2020-07-05	Unidade de Vigilância Epiudemiológica de Araras	Instituto Adolfo Lutz, Interdiciplinary Procedures Center, Strategic Laboratory	Claudio Tavares Sacchi et al
EPI_ISL_596557	2020-07-05	Palestinian Ministry of Health	Molecular Genetics Lab	Nouar Qutob et al
EPI_ISL_487363	2020-07-06	National Institute of Laboratory Medicine and Referral Center	Genomic Research Lab, BCSIR	Tanjina Akhter Banu et al
EPI_ISL_487366	2020-07-06	National Institute of Laboratory Medicine and Referral Center	Genomic Research Lab, BCSIR	Shahina Akter et al
EPI_ISL_487376	2020-07-06	National Institute of Laboratory Medicine and Referral Center	Genomic Research Lab, BCSIR	Md. Saddam Hossain et al
EPI_ISL_693210	2020-07-06	Pronto-Socorro Dr. Osmar Mesquita	Instituto Adolfo Lutz, Interdiciplinary Procedures Center, Strategic Laboratory	Claudio Tavares Sacchi et al
EPI_ISL_693215	2020-07-06	Secretaria Municipal de Saúde de Iracemapolis	Instituto Adolfo Lutz, Interdiciplinary Procedures Center, Strategic Laboratory	Claudio Tavares Sacchi et al
EPI_ISL_618190	2020-07-06	Department of Virus and Microbiological Special Diagnostics, Statens Serum Institut, Denmark	Albertsen lab, Department of Chemistry and Bioscience, Aalborg University, Denmark	Danish Covid-19 Genome Consortia et al
EPI_ISL_526225	2020-07-06	Hungarian Defence Forces Military Medical Centre	National Laboratory of Virology, Szentágothai Research Centre	Endre Gábor Tóth et al
EPI_ISL_635566	2020-07-06	Centro de Diagn√≥stico COVID-19 UABC Tijuana	Andersen lab at Scripps Research	SEARCH Alliance San Diego with Idanya Rubí Serafín Higuera et al
EPI_ISL_636981	2020-07-06	City of Chimoio	KRISP, KZN Research Innovation and Sequencing Platform	Ismael N et al
EPI_ISL_541651	2020-07-06	Laboratory Diagnostic, Veterinary Specialized Institute Kraljevo	Laboratory Diagnostic, Veterinary Specialized Institute Kraljevo	Vidanovic et al
EPI_ISL_522484	2020-07-06	Division of Viral Diseases, Center for Laboratory Control of Infectious Diseases, Korea Centers for Diseases Control and Prevention	Division of Viral Diseases, Center for Laboratory Control of Infectious Diseases, Korea Centers for Diseases Control and Prevention	Jeong-Min Kim et al
EPI_ISL_514215	2020-07-06	Florida Bureau of Public Health Laboratories	Florida Bureau of Public Health Laboratories	Sarah Schmedes et al
EPI_ISL_547579	2020-07-07	Santa Casa de Misericórdia de Araçatuba	Instituto Adolfo Lutz, Interdiciplinary Procedures Center, Strategic Laboratory	Claudio Tavares Sacchi et al
EPI_ISL_653756	2020-07-07	Instituto Nacional de Salud, Bogotá, Colombia	Instituto Nacional de Salud, Bogotá, Colombia	Katherine Laiton-Donato et al
EPI_ISL_526967	2020-07-07	Instituto Nacional de Salud, Bogotá, Colombia	Instituto Nacional de Salud, Bogotá, Colombia	Katherine Laiton-Donato et al
EPI_ISL_648324	2020-07-07	Laboratorio de Investigaciones de Baney	University Hospital Basel, Clinical Bacteriology	Carlos Cortes et al
EPI_ISL_628760	2020-07-07	UHAS COVID-19 Lab	UHAS COVID-19 Lab	Kwabena O. Duedu et al

EPI_ISL_500586	2020-07-07	National Virus Reference Laboratory	National Virus Reference Laboratory	Michael Carr et al
EPI_ISL_590695	2020-07-07	INMI Lazzaro Spallanzani IRCCS	INMI Lazzaro Spallanzani IRCCS	Cesare E.M. Gruber et al
EPI_ISL_590698	2020-07-07	INMI Lazzaro Spallanzani IRCCS	INMI Lazzaro Spallanzani IRCCS	Barbara Bartolini et al
EPI_ISL_635569	2020-07-07	Centro de Diagn√≥stico COVID-19 UABC Tijuana	Andersen lab at Scripps Research	SEARCH Alliance San Diego with Idanya Rubí Serafín Higuera et al
EPI_ISL_636979	2020-07-07	HP Pemba	KRISP, KZN Research Innovation and Sequencing Platform	Ismael N et al
EPI_ISL_548138	2020-07-07	Canterbury Health Laboratories	Institute of Environmental Science and Research (ESR)	Xiaoyun Ren et al
EPI_ISL_729971	2020-07-07	Nigeria Centre for Disease Control (NCDC)	African Centre of Excellence for Genomics of Infectious Diseases (ACEGID), Redeemer's University, Ede, Osun State, Nigeria	Oluniyi P.E. et al et al
EPI_ISL_729972	2020-07-07	Nigeria Centre for Disease Control (NCDC)	African Centre of Excellence for Genomics of Infectious Diseases (ACEGID), Redeemer's University, Ede, Osun State, Nigeria	Oluniyi P.E. et al et al
EPI_ISL_693611	2020-07-07	Instituto Nacional de Saude (INSA)	Instituto Nacional de Saude (INSA)	Borges et al et al
EPI_ISL_678492	2020-07-07	Veterinary Specialized Institute "Sabac", Serbia	Veterinary Specialized Institute "Kraljevo", Serbia	Vidanovic et al
EPI_ISL_517657	2020-07-07	Academic Hospital Paramaribo	Erasmus Medical Center	Bas Oude Munnink et al
EPI_ISL_520273	2020-07-08	Victorian Infectious Diseases Reference Laboratory (VIDRL)	VIDRL and MDU-PHL	Caly L. et al
EPI_ISL_717861	2020-07-08	Laboratorio de Virologia Molecular / UFRJ	Bioinformatics Laboratory / LNCC	Carolina M Voloch et al
EPI_ISL_717862	2020-07-08	Laboratorio de Virologia Molecular / UFRJ	Bioinformatics Laboratory / LNCC	Carolina M Voloch et al
EPI_ISL_653760	2020-07-08	Instituto Nacional de Salud, Bogotá, Colombia	Instituto Nacional de Salud, Bogotá, Colombia	Katherine Laiton-Donato et al
EPI_ISL_500594	2020-07-08	National Virus Reference Laboratory	National Virus Reference Laboratory	Michael Carr et al
EPI_ISL_492993	2020-07-08	E. Gulbja Laboratorija	Latvian Biomedical Research and Study Centre	Ivars Silamiķelis et al
EPI_ISL_492994	2020-07-08	E. Gulbja Laboratorija	Latvian Biomedical Research and Study Centre	Ivars Silamiķelis et al
EPI_ISL_501275	2020-07-08	E. Gulbja Laboratorija	Latvian Biomedical Research and Study Centre	Ivars Silamiķelis et al
EPI_ISL_718143	2020-07-08	Ministry of Health Hospitals	Institute of Health and Community Medicine	David Perera et al
EPI_ISL_729973	2020-07-08	Nigeria Centre for Disease Control (NCDC)	African Centre of Excellence for Genomics of Infectious Diseases (ACEGID), Redeemer's University, Ede, Osun State, Nigeria	Oluniyi P.E. et al et al
EPI_ISL_516428	2020-07-08	Clinical Hospital - Shtip	Research Center for Genetic Engineering and Biotechnology "Georgi D. Efremov" , Macedonian Academy of Sciences and Arts	RCGEB - MASA et al
EPI_ISL_693615	2020-07-08	Instituto Nacional de Saude (INSA)	Instituto Nacional de Saude (INSA)	Borges et al et al
EPI_ISL_569814	2020-07-08	Omsk Research Institute of Natural Focal Infections	WHO National Influenza Centre Russian Federation	Artem Fadeev et al
EPI_ISL_541649	2020-07-08	Laboratory Diagnostic, Veterinary Specialized Institute Kraljevo	Laboratory Diagnostic, Veterinary Specialized Institute Kraljevo	Vidanovic et al
EPI_ISL_678491	2020-07-08	Veterinary Specialized Institute "Sabac", Serbia	Veterinary Specialized Institute "Kraljevo", Serbia	Vidanovic et al
EPI_ISL_517851	2020-07-08	Florida Bureau of Public Health Laboratories	Florida Bureau of Public Health Laboratories	Sarah Schmedes et al
EPI_ISL_519397	2020-07-09	Microbiological Diagnostic Unit - Public Health Laboratory (MDU-PHL)	MDU-PHL	Seemann T. et al
EPI_ISL_610180	2020-07-09	Department of Health Technology and Informatics, The Hong Kong Polytechnic University	Department of Health Technology and Informatics, The Hong Kong Polytechnic University	Siu et al
EPI_ISL_500592	2020-07-09	National Virus Reference Laboratory	National Virus Reference Laboratory	Michael Carr et al
EPI_ISL_493000	2020-07-09	E. Gulbja Laboratorija	Latvian Biomedical Research and Study Centre	Ivars Silamiķelis et al
EPI_ISL_596544	2020-07-09	Palestinian Ministry of Health	Molecular Genetics Lab	Nouar Qutob et al
EPI_ISL_498251	2020-07-09	Institut Pasteur de Dakar	Institut Pasteur de Dakar	Ndongo Dia et al
EPI_ISL_498252	2020-07-09	Institut Pasteur de Dakar	Institut Pasteur de Dakar	Ndongo Dia et al
EPI_ISL_541661	2020-07-09	Laboratory Diagnostic, Veterinary Specialized Institute Kraljevo	Laboratory Diagnostic, Veterinary Specialized Institute Kraljevo	Vidanovic et al
EPI_ISL_661186	2020-07-09	Scientific Veterinary Institute Novi Sad	Veterinary Specialized Institute "Kraljevo", Serbia	Vidanovic et al
EPI_ISL_498582	2020-07-09	National Public Health Laboratory, National Centre for Infectious Diseases	National Public Health Laboratory, National Centre for Infectious Diseases	Mak TM et al

EPI_ISL_498585	2020-07-09	National Public Health Laboratory, National Centre for Infectious Diseases	National Public Health Laboratory, National Centre for Infectious Diseases	Mak TM et al
EPI_ISL_577734	2020-07-09	Institute of Virology, Biomedical Research Center of the Slovak Academy of Sciences, Bratislava	Faculty of Natural Sciences, Comenius University, Bratislava	Viktória Hodorová et al
EPI_ISL_500926	2020-07-09	Viollier AG	Department of Biosystems Science and Engineering, ETH Zürich	Christian Beisel et al
EPI_ISL_500915	2020-07-09	Viollier AG	Department of Biosystems Science and Engineering, ETH Zürich	Christian Beisel et al
EPI_ISL_515065	2020-07-10	Department of Clinical Microbiology	GIGA Medical Genomics	Keith Durkin et al
EPI_ISL_574594	2020-07-10	Hospital Escola da Universidade de Taubate	Instituto Adolfo Lutz, Interdiciplinary Procedures Center, Strategic Laboratory	Claudio Tavares Sacchi et al
EPI_ISL_603035	2020-07-10	Secretaria Municipal de Saúde	Instituto Adolfo Lutz, Interdiciplinary Procedures Center, Strategic Laboratory	Claudio Tavares Sacchi et al
EPI_ISL_603036	2020-07-10	Hospital Santa Ana	Instituto Adolfo Lutz, Interdiciplinary Procedures Center, Strategic Laboratory	Claudio Tavares Sacchi et al
EPI_ISL_582319	2020-07-10	Cadham Provincial Laboratory	National Microbiology Laboratory (NML)	Anna Majer et al
EPI_ISL_497859	2020-07-10	Department of Microbiology, The University of Hong Kong	Department of Microbiology, The University of Hong Kong	Kelvin K.W. To et al
EPI_ISL_501269	2020-07-10	National Virus Reference Laboratory	National Virus Reference Laboratory	Michael Carr et al
EPI_ISL_501284	2020-07-10	E. Gulbja Laboratorija	Latvian Biomedical Research and Study Centre	Ivars Silamiķelis et al
EPI_ISL_548139	2020-07-10	Canterbury Health Laboratories	Institute of Environmental Science and Research (ESR)	Xiaoyun Ren et al
EPI_ISL_729935	2020-07-10	Nigeria Centre for Disease Control (NCDC)	African Centre of Excellence for Genomics of Infectious Diseases (ACEGID), Redeemer's University, Ede, Osun State, Nigeria	Oluniyi P.E. et al et al
EPI_ISL_677716	2020-07-10	Clinical Hospital - Shtip	Research Center for Genetic Engineering and Biotechnology "Georgi D. Efremov" , Macedonian Academy of Sciences and Arts	RCGEB - MASA et al
EPI_ISL_512616	2020-07-10	National Laboratory for Influenza/Virology reference laboratory, Public Health Center of the Ministry of Health of Ukraine	Respiratory Virus Unit, Microbiology Services Colindale, Public Health England	PHE Covid Sequencing Team et al
EPI_ISL_512617	2020-07-10	National Laboratory for Influenza/Virology reference laboratory, Public Health Center of the Ministry of Health of Ukraine	Respiratory Virus Unit, Microbiology Services Colindale, Public Health England	PHE Covid Sequencing Team et al
EPI_ISL_515066	2020-07-11	Department of Clinical Microbiology	GIGA Medical Genomics	Keith Durkin et al
EPI_ISL_603030	2020-07-11	Hospital Domingos Leonardo Ceravolo Presidente Prudente	Instituto Adolfo Lutz, Interdiciplinary Procedures Center, Strategic Laboratory	Claudio Tavares Sacchi et al
EPI_ISL_603034	2020-07-11	Departamento de Vigilância à Saúde	Instituto Adolfo Lutz, Interdiciplinary Procedures Center, Strategic Laboratory	Claudio Tavares Sacchi et al
EPI_ISL_648376	2020-07-11	Laboratorio de Investigaciones de Baney	University Hospital Basel, Clinical Bacteriology	Carlos Cortes et al
EPI_ISL_636977	2020-07-11	HP Pemba	KRISP, KZN Research Innovation and Sequencing Platform	Ismael N et al
EPI_ISL_636978	2020-07-11	HP Pemba	KRISP, KZN Research Innovation and Sequencing Platform	Ismael N et al
EPI_ISL_523534	2020-07-11	Dutch COVID-19 response team	Erasmus Medical Center	Bas Oude Munnink et al
EPI_ISL_693627	2020-07-11	Instituto Nacional de Saude (INSA)	Instituto Nacional de Saude (INSA)	Borges et al et al
EPI_ISL_693628	2020-07-11	Instituto Nacional de Saude (INSA)	Instituto Nacional de Saude (INSA)	Borges et al et al
EPI_ISL_569784	2020-07-11	Omsk Research Institute of Natural Focal Infections	WHO National Influenza Centre Russian Federation	Artem Fadeev et al
EPI_ISL_522491	2020-07-11	Center for Laboratory Control of Infectious Diseases, Korea Centers for Diseases Control and Prevention	Center for Laboratory Control of Infectious Diseases, Korea Centers for Diseases Control and Prevention	Junyoung Kim et al
EPI_ISL_497838	2020-07-12	Department of Microbiology, The University of Hong Kong	Department of Microbiology, The University of Hong Kong	Kelvin K.W. To et al
EPI_ISL_509245	2020-07-12	NHLS-IALCH	KRISP, KZN Research Innovation and Sequencing Platform	Giandhari J et al
EPI_ISL_520590	2020-07-13	Microbiological Diagnostic Unit - Public Health Laboratory (MDU-PHL)	MDU-PHL	Seemann T. et al
EPI_ISL_729801	2020-07-13	Laboratorio Central de Saude Publica do Estado do Rio Grande do Sul (LACEN-RS)	Laboratory of Respiratory Viruses and Measles, Oswaldo Cruz Institute, FIOCRUZ	Paola Resende et al
EPI_ISL_603033	2020-07-13	Vigilancia Epidemiologica de São Bernardo do Campo	Instituto Adolfo Lutz, Interdiciplinary Procedures Center, Strategic Laboratory	Claudio Tavares Sacchi et al

EPI_ISL_618302	2020-07-13	Department of Virus and Microbiological Special Diagnostics, Statens Serum Institut, Denmark	Albertsen lab, Department of Chemistry and Bioscience, Aalborg University, Denmark	Danish Covid-19 Genome Consortia et al
EPI_ISL_569007	2020-07-13	MEPHI, Aix Marseille University	MEPHI, Aix Marseille University	Anthony LEVASSEUR et al
EPI_ISL_568564	2020-07-13	Department of Infectious Diseases and Immunology, National Hospital Organization Nagoya Medical Center	Clinical Research Center, National Hospital Organization Nagoya Medical Center	Yoshihiro Nakata et al
EPI_ISL_636976	2020-07-13	HP Pemba	KRISP, KZN Research Innovation and Sequencing Platform	Ismael N et al
EPI_ISL_549168	2020-07-13	Furst Medical Laboratory	Norwegian Institute of Public Health, Department of Virology	Kathrine Stene-Johansen et al
EPI_ISL_578188	2020-07-13	Hospital Virgen de las Nieves	Instituto de Salud Carlos III	Iglesias-Caballero et al
EPI_ISL_517658	2020-07-13	Academic Hospital Paramaribo	Erasmus Medical Center	Bas Oude Munnink et al
EPI_ISL_648138	2020-07-13	The Public Health Agency of Sweden	The Public Health Agency of Sweden	Anna-Malin Linde et al
EPI_ISL_695611	2020-07-13	AZ SPHL, Arizona Department of Health Services	TGen North	Jolene Bowers et al
EPI_ISL_516649	2020-07-14	Institute of Microbiology, Universidad San Francisco de Quito	Institute of Microbiology, Universidad San Francisco de Quito	Juan José Guadalupe et al
EPI_ISL_548140	2020-07-14	Canterbury Health Laboratories	Institute of Environmental Science and Research (ESR)	Xiaoyun Ren et al
EPI_ISL_516430	2020-07-14	Clinical Hospital - Shtip	Research Center for Genetic Engineering and Biotechnology "Georgi D. Efremov" , Macedonian Academy of Sciences and Arts	RCGEB - MASA et al
EPI_ISL_517659	2020-07-14	Academic Hospital Paramaribo	Erasmus Medical Center	Bas Oude Munnink et al
EPI_ISL_534245	2020-07-14	Kliniskt mikrobiologiska laboratoriet	The Public Health Agency of Sweden	Anna-Malin Linde et al
EPI_ISL_603021	2020-07-15	Pronto Socorro Dr. Conrado Cesarino Nuvolini	Instituto Adolfo Lutz, Interdiciplinary Procedures Center, Strategic Laboratory	Claudio Tavares Sacchi et al
EPI_ISL_532970	2020-07-15	Lighthouse Lab in Glasgow	Wellcome Sanger Institute for the COVID-19 Genomics UK (COG-UK) consortium	Harper VanSteenhouse et al
EPI_ISL_528844	2020-07-15	CSIR-Centre for Cellular and Molecular Biology	CSIR-Centre for Cellular and Molecular Biology	Namami Gaur et al
EPI_ISL_528849	2020-07-15	CSIR-Centre for Cellular and Molecular Biology	CSIR-Centre for Cellular and Molecular Biology	Nikhil Hajirnis et al
EPI_ISL_528850	2020-07-15	CSIR-Centre for Cellular and Molecular Biology	CSIR-Centre for Cellular and Molecular Biology	Lamuk Zaveri et al
EPI_ISL_644565	2020-07-15	Veterinary Specialized Institute "Kraljevo", Serbia	Veterinary Specialized Institute "Kraljevo", Serbia	Vidanovic et al
EPI_ISL_498103	2020-07-15	NHLS-IALCH	KRISP, KZN Research Innovation and Sequencing Platform	Giandhari J et al
EPI_ISL_497950	2020-07-15	Shaoxing CDC	Zhejiang Provincial Center for Disease Control and Prevention	Yin Chen et al
EPI_ISL_515061	2020-07-16	Department of Clinical Microbiology	GIGA Medical Genomics	Keith Durkin et al
EPI_ISL_603022	2020-07-16	Departamento de Vigilância à Saúde	Instituto Adolfo Lutz, Interdiciplinary Procedures Center, Strategic Laboratory	Claudio Tavares Sacchi et al
EPI_ISL_582383	2020-07-16	Cadham Provincial Laboratory	National Microbiology Laboratory (NML)	Anna Majer et al
EPI_ISL_557324	2020-07-16	Lighthouse Lab in Milton Keynes	Wellcome Sanger Institute for the COVID-19 Genomics UK (COG-UK) consortium	The Lighthouse Lab in Milton Keynes et al
EPI_ISL_596501	2020-07-16	Palestinian Ministry of Health	Molecular Genetics Lab	Nouar Qutob et al
EPI_ISL_596522	2020-07-16	Palestinian Ministry of Health	Molecular Genetics Lab	Nouar Qutob et al
EPI_ISL_522515	2020-07-16	Center for Laboratory Control of Infectious Diseases, Korea Centers for Diseases Control and Prevention	Center for Laboratory Control of Infectious Diseases, Korea Centers for Diseases Control and Prevention	Junyoung Kim et al
EPI_ISL_518800	2020-07-16	Academic Hospital Paramaribo	Erasmus Medical Center	Bas Oude Munnink et al
EPI_ISL_518805	2020-07-16	Academic Hospital Paramaribo	Erasmus Medical Center	Bas Oude Munnink et al
EPI_ISL_518807	2020-07-16	Academic Hospital Paramaribo	Erasmus Medical Center	Bas Oude Munnink et al
EPI_ISL_518808	2020-07-16	Academic Hospital Paramaribo	Erasmus Medical Center	Bas Oude Munnink et al
EPI_ISL_518809	2020-07-16	Academic Hospital Paramaribo	Erasmus Medical Center	Bas Oude Munnink et al
EPI_ISL_518811	2020-07-16	Academic Hospital Paramaribo	Erasmus Medical Center	Bas Oude Munnink et al
EPI_ISL_518812	2020-07-16	Academic Hospital Paramaribo	Erasmus Medical Center	Bas Oude Munnink et al
EPI_ISL_729844	2020-07-17	Laboratorio Central de Saude Publica do Estado do Rio Grande do Sul (LACEN-RS)	Laboratory of Respiratory Viruses and Measles, Oswaldo Cruz Institute, FIOCRUZ	Paola Resende et al

EPI_ISL_729850	2020-07-17	Laboratorio Central de Saude Publica do Estado do Rio Grande do Sul (LACEN-RS)	Laboratory of Respiratory Viruses and Measles, Oswaldo Cruz Institute, FIOCRUZ	Paola Resende et al
EPI_ISL_526958	2020-07-17	Instituto Nacional de Salud, Bogotá, Colombia	Instituto Nacional de Salud, Bogotá, Colombia	Katherine Laiton-Donato et al
EPI_ISL_681838	2020-07-17	Molecular diagnostic unit for viral haemorrhagic fevers and emerging viruses, Bouaké CHU Laboratory	Project group Epidemology of Highly Pathogenic Microorganisms, Robert Koch-Institute	Chantal Akoua-Koffi et al
EPI_ISL_697797	2020-07-17	Centro de Investigaciones, Universidad de Especialidades Espíritu Santo	Institute of Microbiology, Universidad San Francisco de Quito	Derly Andrade et al
EPI_ISL_516925	2020-07-17	Department for Molecular Diagnostics, Centre for Medical Microbiology, Institute of Public Health of Montenegro	Charité Universitätsmedizin Berlin, Institut für Virologie	Victor M Corman et al
EPI_ISL_516926	2020-07-17	Department for Molecular Diagnostics, Centre for Medical Microbiology, Institute of Public Health of Montenegro	Charité Universitätsmedizin Berlin, Institut für Virologie	Victor M Corman et al
EPI_ISL_516927	2020-07-17	Department for Molecular Diagnostics, Centre for Medical Microbiology, Institute of Public Health of Montenegro	Charité Universitätsmedizin Berlin, Institut für Virologie	Victor M Corman et al
EPI_ISL_548423	2020-07-17	County of Santa Clara Public Health Department	Chan-Zuckerberg Biohub	CZB Cliahub Consortium et al
EPI_ISL_498818	2020-07-18	National Institute of Laboratory Medicine and Referral Center	Genomic Research Lab, BCSIR	Abu Sayeed Mohammad Mahmud et al
EPI_ISL_729856	2020-07-18	Laboratorio Central de Saude Publica do Estado do Rio Grande do Sul (LACEN-RS)	Laboratory of Respiratory Viruses and Measles, Oswaldo Cruz Institute, FIOCRUZ	Paola Resende et al
EPI_ISL_729469	2020-07-18	A. Krumbholz, Labor Dr. Krause und Kollegen MVZ GmbH, Kiel	Charité Universitätsmedizin Berlin, Institut für Virologie	Victor M Corman et al
EPI_ISL_722856	2020-07-18	Dipartimento di Scienze Biomediche e Oncologia Umana - Azienda Ospedaliero Universitaria Consorziale Policlinico	Istituto Zooprofilattico Sperimentale della Puglia e della Basilicata	Parisi A. et al
EPI_ISL_722857	2020-07-18	Dipartimento di Scienze Biomediche e Oncologia Umana - Azienda Ospedaliero Universitaria Consorziale Policlinico	Istituto Zooprofilattico Sperimentale della Puglia e della Basilicata	Parisi A. et al
EPI_ISL_718146	2020-07-18	Ministry of Health Hospitals	Institute of Health and Community Medicine	David Perera et al
EPI_ISL_516928	2020-07-18	Department for Molecular Diagnostics, Centre for Medical Microbiology, Institute of Public Health of Montenegro	Charité Universitätsmedizin Berlin, Institut für Virologie	Victor M Corman et al
EPI_ISL_678221	2020-07-18	Pathogen Genomics Lab King Abdullah University of Science and Technology(KAUST)	Pathogen Genomics Lab King Abdullah University of Science and Technology(KAUST)	Sara Mfarrej et al
EPI_ISL_678222	2020-07-18	Pathogen Genomics Lab King Abdullah University of Science and Technology(KAUST)	Pathogen Genomics Lab King Abdullah University of Science and Technology(KAUST)	Sara Mfarrej et al
EPI_ISL_583885	2020-07-19	Austrian Agency for Health and Food Safety (AGES)	Bergthaler laboratory, CeMM Research Center for Molecular Medicine of the Austrian Academy of Sciences	Alexandra Popa et al
EPI_ISL_583886	2020-07-19	Austrian Agency for Health and Food Safety (AGES)	Bergthaler laboratory, CeMM Research Center for Molecular Medicine of the Austrian Academy of Sciences	Alexandra Popa et al
EPI_ISL_498830	2020-07-19	National Institute of Laboratory Medicine and Referral Center	Genomic Research Lab, BCSIR	Abu Sayeed Mohammad Mahmud et al
EPI_ISL_693214	2020-07-19	Unidade de Pronto Atendimento Central de Caraguatatuba	Instituto Adolfo Lutz, Interdiciplinary Procedures Center, Strategic Laboratory	Claudio Tavares Sacchi et al
EPI_ISL_582477	2020-07-19	Cadham Provincial Laboratory	National Microbiology Laboratory (NML)	Anna Majer et al
EPI_ISL_524427	2020-07-19	Egyptian National Cancer Institute (ENCI)	Egyptian National Cancer Institute (ENCI)	Zekri et al
EPI_ISL_524426	2020-07-19	Egyptian National Cancer Institute (ENCI)	Egyptian National Cancer Institute (ENCI)	Zekri et al
EPI_ISL_576373	2020-07-19	Cancer Biology Department, National Cancer Institute	Cancer Biology Department, National Cancer Institute	Zekri et al
EPI_ISL_529143	2020-07-19	Egyptian National Cancer Institute (ENCI)	Egyptian National Cancer Institute (ENCI)	Zekri et al
EPI_ISL_529144	2020-07-19	Egyptian National Cancer Institute (ENCI)	Egyptian National Cancer Institute (ENCI)	Zekri et al
EPI_ISL_729470	2020-07-19	A. Krumbholz, Labor Dr. Krause und Kollegen MVZ GmbH, Kiel	Charité Universitätsmedizin Berlin, Institut für Virologie	Victor M Corman et al
EPI_ISL_510540	2020-07-19	Department of Microbiology, The University of Hong Kong	Department of Microbiology, The University of Hong Kong	Kelvin K.W. To et al
EPI_ISL_528813	2020-07-19	Department of Medicine, Gandhi hospital, Hyderabad	CSIR-Centre for Cellular and Molecular Biology	Vinayasekhar Aedula et al
EPI_ISL_514275	2020-07-19	Israeli Central Virology laboratory	Israel Central Virology laboratory	Neta Zuckerman et al
EPI_ISL_516933	2020-07-19	Department for Molecular Diagnostics, Centre for Medical Microbiology, Institute of Public Health of Montenegro	Charité Universitätsmedizin Berlin, Institut für Virologie	Victor M Corman et al

EPI_ISL_518770	2020-07-20	Microbiological Diagnostic Unit - Public Health Laboratory (MDU-PHL)	MDU-PHL	Seemann T. et al
EPI_ISL_509430	2020-07-20	Centro de Desenvolvimento Tecnologico em Saude, Fundacao Oswaldo Cruz	Centro de Desenvolvimento Tecnologico em Saude, Fundacao Oswaldo Cruz	Souza et al
EPI_ISL_509431	2020-07-20	Centro de Desenvolvimento Tecnologico em Saude, Fundacao Oswaldo Cruz	Centro de Desenvolvimento Tecnologico em Saude, Fundacao Oswaldo Cruz	Souza et al
EPI_ISL_509432	2020-07-20	Centro de Desenvolvimento Tecnologico em Saude, Fundacao Oswaldo Cruz	Centro de Desenvolvimento Tecnologico em Saude, Fundacao Oswaldo Cruz	Souza et al
EPI_ISL_509433	2020-07-20	Centro de Desenvolvimento Tecnologico em Saude, Fundacao Oswaldo Cruz	Centro de Desenvolvimento Tecnologico em Saude, Fundacao Oswaldo Cruz	Souza et al
EPI_ISL_529139	2020-07-20	Centro de Desenvolvimento Tecnologico em Saude, Fundacao Oswaldo Cruz	Centro de Desenvolvimento Tecnologico em Saude, Fundacao Oswaldo Cruz	Souza et al
EPI_ISL_529140	2020-07-20	Centro de Desenvolvimento Tecnologico em Saude, Fundacao Oswaldo Cruz	Centro de Desenvolvimento Tecnologico em Saude, Fundacao Oswaldo Cruz	Souza et al
EPI_ISL_509435	2020-07-20	Centro de Desenvolvimento Tecnologico em Saude, Fundacao Oswaldo Cruz	Centro de Desenvolvimento Tecnologico em Saude, Fundacao Oswaldo Cruz	Souza et al
EPI_ISL_729857	2020-07-20	Laboratorio Central de Saude Publica do Estado do Rio Grande do Sul (LACEN-RS)	Laboratory of Respiratory Viruses and Measles, Oswaldo Cruz Institute, FIOCRUZ	Paola Resende et al
EPI_ISL_603037	2020-07-20	Hospital Geral de Pedreira	Instituto Adolfo Lutz, Interdiciplinary Procedures Center, Strategic Laboratory	Claudio Tavares Sacchi et al
EPI_ISL_626550	2020-07-20	Laboratorio de Biología Molecular, Facultad de Medicina, Universidad de Atacama, Copiapo, Chile/ FONDAP CRG, Universidad Andrés Bello, Santiago, Chile	Center for Mathematical Modeling and Center for Genome Regulation. Santiago, Chile	Echeverría C et al
EPI_ISL_569019	2020-07-20	MEPHI, Aix Marseille University	MEPHI, Aix Marseille University	Anthony LEVASSEUR et al
EPI_ISL_514279	2020-07-20	Israel Central Virology laboratory	Israel Central Virology laboratory	Neta Zuckerman et al
EPI_ISL_514287	2020-07-20	Israel Central Virology laboratory	Israel Central Virology laboratory	Neta Zuckerman et al
EPI_ISL_514289	2020-07-20	Israel Central Virology laboratory	Israel Central Virology laboratory	Neta Zuckerman et al
EPI_ISL_529015	2020-07-20	Ospedale "Ss. Annunziata"	Istituto Zooprofilattico Sperimentale dell'Abruzzo e Molise "G.Caporale"	Lorusso A et al
EPI_ISL_677717	2020-07-20	Clinical Hospital - Shtip	Research Center for Genetic Engineering and Biotechnology "Georgi D. Efremov" , Macedonian Academy of Sciences and Arts	RCGEB - MASA et al
EPI_ISL_693475	2020-07-20	Central Public Health Laboratory	National Public Health Laboratory, National Centre for Infectious Diseases	Tze Minn Mak et al
EPI_ISL_693476	2020-07-20	Central Public Health Laboratory	National Public Health Laboratory, National Centre for Infectious Diseases	Tze Minn Mak et al
EPI_ISL_639739	2020-07-20	Centre of Nanotechnologies, INCD IMT-Bucuresti (National Institute for Research and Development in Microtechnologies - Bucharest)	Centre of Nanotechnologies, INCD IMT-Bucuresti (National Institute for Research and Development in Microtechnologies - Bucharest)	Salceanu et al
EPI_ISL_644566	2020-07-20	Veterinary Specialized Institute "Kraljevo", Serbia	Veterinary Specialized Institute "Kraljevo", Serbia	Vidanovic et al
EPI_ISL_691680	2020-07-20	Servicio de Microbiología, Hospital Universitario Son Espases	SeqCOVID-SPAIN consortium/IBV(CSIC)	Carla López-Causapé et al
EPI_ISL_660446	2020-07-21	Laboratoire de Microbiologie CHU Souro Sanou	Centre Muraz	Abdoul-Salam Ouedraogo et al
EPI_ISL_648377	2020-07-21	Laboratorio de Investigaciones de Baney	University Hospital Basel, Clinical Bacteriology	Carlos Cortes et al
EPI_ISL_636604	2020-07-21	Lithuanian University of Health Sciences Hospital, Department of Laboratory Medicine	Lithuanian University of Health Sciences, Molecular cardiology lab.	Lukas Zemaitis et al
EPI_ISL_636839	2020-07-21	Lithuanian University of Health Sciences Hospital, Department of Laboratory Medicine	Lithuanian University of Health Sciences, Molecular cardiology lab.	Lukas Zemaitis et al
EPI_ISL_677718	2020-07-21	Clinical Hospital - Bitola	Research Center for Genetic Engineering and Biotechnology "Georgi D. Efremov" , Macedonian Academy of Sciences and Arts	RCGEB - MASA et al
EPI_ISL_693471	2020-07-21	Central Public Health Laboratory	National Public Health Laboratory, National Centre for Infectious Diseases	Tze Minn Mak et al
EPI_ISL_693472	2020-07-21	Central Public Health Laboratory	National Public Health Laboratory, National Centre for Infectious Diseases	Tze Minn Mak et al

EPI_ISL_693474	2020-07-21	Central Public Health Laboratory	National Public Health Laboratory, National Centre for Infectious Diseases	Tze Minn Mak et al
EPI_ISL_648739	2020-07-21	Department of Laboratory Medicine, Tan Tock Seng Hospital	Department of Laboratory Medicine, Tan Tock Seng Hospital	Chen YYC et al
EPI_ISL_514417	2020-07-21	National Institute for Communicable Diseases of the National Health Laboratory Service	National Institute for Communicable Diseases of the National Health Laboratory Service	Allam M et al
EPI_ISL_525488	2020-07-21	Centre for Dengue Research	Centre for Dengue Research	Chandima Jeewandara et al
EPI_ISL_525489	2020-07-21	Centre for Dengue Research	Centre for Dengue Research	Chandima Jeewandara et al
EPI_ISL_525486	2020-07-21	Centre for Dengue Research	Centre for Dengue Research	Chandima Jeewandara et al
EPI_ISL_648677	2020-07-22	INBIRS-UBA	Laboratorio Mixto de Biotecnología Acuática (LMBA)	Joaquín Ezpeleta et al
EPI_ISL_648213	2020-07-22	INBIRS-UBA	Laboratorio Mixto de Biotecnología Acuática (LMBA)	Joaquín Ezpeleta et al
EPI_ISL_648215	2020-07-22	INBIRS-UBA	Laboratorio Mixto de Biotecnología Acuática (LMBA)	Joaquín Ezpeleta et al
EPI_ISL_648211	2020-07-22	INBIRS-UBA	Laboratorio Mixto de Biotecnología Acuática (LMBA)	Joaquín Ezpeleta et al
EPI_ISL_648216	2020-07-22	INBIRS-UBA	Laboratorio Mixto de Biotecnología Acuática (LMBA)	Joaquín Ezpeleta et al
EPI_ISL_648210	2020-07-22	INBIRS-UBA	Laboratorio Mixto de Biotecnología Acuática (LMBA)	Joaquín Ezpeleta et al
EPI_ISL_648209	2020-07-22	INBIRS-UBA	Laboratorio Mixto de Biotecnología Acuática (LMBA)	Joaquín Ezpeleta et al
EPI_ISL_648218	2020-07-22	INBIRS-UBA	Laboratorio Mixto de Biotecnología Acuática (LMBA)	Joaquín Ezpeleta et al
EPI_ISL_648212	2020-07-22	INBIRS-UBA	Laboratorio Mixto de Biotecnología Acuática (LMBA)	Joaquín Ezpeleta et al
EPI_ISL_648214	2020-07-22	INBIRS-UBA	Laboratorio Mixto de Biotecnología Acuática (LMBA)	Joaquín Ezpeleta et al
EPI_ISL_569984	2020-07-22	Unity Health Toronto	Ontario Institute for Cancer Research	Ramzi Fattouh et al
EPI_ISL_626552	2020-07-22	Laboratorio de Biología Molecular, Facultad de Medicina, Universidad de Atacama, Copiapo, Chile/ FONDAP CRG, Universidad Andrés Bello, Santiago, Chile	Center for Mathematical Modeling and Center for Genome Regulation. Santiago, Chile	Echeverría C et al
EPI_ISL_546436	2020-07-22	The National Institute of Public Health	State Veterinary Institute Prague	Nagy et al
EPI_ISL_516650	2020-07-22	Institute of Microbiology, Universidad San Francisco de Quito	Institute of Microbiology, Universidad San Francisco de Quito	Prado-Vivar et al
EPI_ISL_498692	2020-07-22	National Institute for Viral Disease Control and Prevention, China CDC	National Institute for Viral Disease Control and Prevention, China CDC	Xiang Zhao et al
EPI_ISL_498694	2020-07-22	National Institute for Viral Disease Control and Prevention, China CDC	National Institute for Viral Disease Control and Prevention, China CDC	Xiang Zhao et al
EPI_ISL_498693	2020-07-22	National Institute for Viral Disease Control and Prevention, China CDC	National Institute for Viral Disease Control and Prevention, China CDC	Xiang Zhao et al
EPI_ISL_498691	2020-07-22	National Institute for Viral Disease Control and Prevention, China CDC	National Institute for Viral Disease Control and Prevention, China CDC	Xiang Zhao et al
EPI_ISL_644567	2020-07-22	Veterinary Specialized Institute "Kraljevo", Serbia	Veterinary Specialized Institute "Kraljevo", Serbia	Vidanovic et al
EPI_ISL_515698	2020-07-22	NHLS-IALCH	KRISP, KZN Research Innovation and Sequencing Platform	Giandhari J et al
EPI_ISL_510803	2020-07-22	Viollier AG	Department of Biosystems Science and Engineering, ETH Zürich	Christian Beisel et al
EPI_ISL_540458	2020-07-23	University of Liège COVID-19 testing center	GIGA Medical Genomics	Keith Durkin et al
EPI_ISL_660478	2020-07-23	Laboratoire de Microbiologie CHU Souro Sanou	Centre Muraz	Abdoul-Salam Ouedraogo et al
EPI_ISL_729573	2020-07-23	A. Krumbholz, Labor Dr. Krause und Kollegen MVZ GmbH, Kiel	Charité Universitätsmedizin Berlin, Institut für Virologie	Victor M Corman et al
EPI_ISL_528748	2020-07-23	Dinkes Provinsi Jawa Barat	School of Life Sciences and Technology & School of Pharmacy-Institut Teknologi Bandung; Molecular Genetics Laboratory-Faculty of Medicine-Universitas Padjadjaran; Laboratorium Kesehatan Provinsi Jawa Barat	Azzania Fibriani et al
EPI_ISL_636997	2020-07-23	Department of Infectious Diseases and Immunology, National Hospital Organization Nagoya Medical Center	Clinical Research Center, National Hospital Organization Nagoya Medical Center	Yoshihiro Nakata et al
EPI_ISL_548145	2020-07-23	Middlemore Hospital	Institute of Environmental Science and Research (ESR)	Xiaoyun Ren et al
EPI_ISL_541655	2020-07-23	Laboratory Diagnostic, Veterinary Specialized Institute Kraljevo	Laboratory Diagnostic, Veterinary Specialized Institute Kraljevo	Vidanovic et al
EPI_ISL_691637	2020-07-23	Servicio de Microbiología, Hospital Universitario Son Espases	SeqCOVID-SPAIN consortium/IBV(CSIC)	Carla López-Causapé et al

EPI_ISL_577624	2020-07-24	The National Institute of Public Health	State Veterinary Institute Prague	Nagy et al
EPI_ISL_560598	2020-07-24	Hospital	National Reference Center for Viruses of Respiratory Infections, Institut Pasteur, Paris	Sylvie Behillil et al
EPI_ISL_560599	2020-07-24	Hospital	National Reference Center for Viruses of Respiratory Infections, Institut Pasteur, Paris	Sylvie Behillil et al
EPI_ISL_644970	2020-07-24	Department of Infectious Diseases, Keio University School of Medicine, Tokyo, Japan	Center for Medical Genetics, Keio University School of Medicine, Tokyo, Japan	Kenjiro Kosaki et al
EPI_ISL_708797	2020-07-24	Regional medical sciences center 6 chonburi	National Institute of Health, Department of Medical Sciences, Ministry of Public Health, Thailand	Pilailuk Okada et al
EPI_ISL_708801	2020-07-24	Regional medical sciences center 6 chonburi	National Institute of Health, Department of Medical Sciences, Ministry of Public Health, Thailand	Pilailuk Okada et al
EPI_ISL_632283	2020-07-25	Communicable Disease Laboratory, Public Health Directorate	Communicable Disease Laboratory, Public Health Directorate	AlWasti et al
EPI_ISL_632284	2020-07-25	Communicable Disease Laboratory, Public Health Directorate	Communicable Disease Laboratory, Public Health Directorate	AlWasti et al
EPI_ISL_540466	2020-07-25	University of Liège COVID-19 testing center	GIGA Medical Genomics	Keith Durkin et al
EPI_ISL_660479	2020-07-25	Laboratoire de Microbiologie CHU Souro Sanou	Centre Muraz	Abdoul-Salam Ouedraogo et al
EPI_ISL_626551	2020-07-25	Laboratorio de Biología Molecular, Facultad de Medicina, Universidad de Atacama, Copiapo, Chile/ FONDAP CRG, Universidad Andrés Bello, Santiago, Chile	Center for Mathematical Modeling and Center for Genome Regulation. Santiago, Chile	Echeverría C et al
EPI_ISL_626553	2020-07-25	Laboratorio de Biología Molecular, Facultad de Medicina, Universidad de Atacama, Copiapo, Chile/ FONDAP CRG, Universidad Andrés Bello, Santiago, Chile	Center for Mathematical Modeling and Center for Genome Regulation. Santiago, Chile	Echeverría C et al
EPI_ISL_626555	2020-07-25	Laboratorio de Biología Molecular, Facultad de Medicina, Universidad de Atacama, Copiapo, Chile/ FONDAP CRG, Universidad Andrés Bello, Santiago, Chile	Center for Mathematical Modeling and Center for Genome Regulation. Santiago, Chile	Echeverría C et al
EPI_ISL_516651	2020-07-25	Institute of Microbiology, Universidad San Francisco de Quito	Institute of Microbiology, Universidad San Francisco de Quito	Prado-Vivar et al
EPI_ISL_529259	2020-07-25	Liverpool Clinical Laboratories	COVID-19 Genomics UK (COG-UK) Consortium	Sam Haldenby et al
EPI_ISL_644972	2020-07-25	Department of Infectious Diseases, Keio University School of Medicine, Tokyo, Japan	Center for Medical Genetics, Keio University School of Medicine, Tokyo, Japan	Kenjiro Kosaki et al
EPI_ISL_718283	2020-07-25	Institute for Medical Research, Infectious Disease Research Centre, National Institutes of Health, Ministry of Health Malaysia	Institute for Medical Research, Infectious Disease Research Centre, National Institutes of Health, Ministry of Health Malaysia	Suppiah J et al
EPI_ISL_708737	2020-07-25	Regional medical sciences center 6 chonburi	National Institute of Health, Department of Medical Sciences, Ministry of Public Health, Thailand	Pilailuk Okada et al
EPI_ISL_632267	2020-07-26	Communicable Disease Laboratory, Public Health Directorate	Communicable Disease Laboratory, Public Health Directorate	AlWasti et al
EPI_ISL_569993	2020-07-26	Unity Health Toronto	Ontario Institute for Cancer Research	Ramzi Fattouh et al
EPI_ISL_626554	2020-07-26	Laboratorio de Biología Molecular, Facultad de Medicina, Universidad de Atacama, Copiapo, Chile/ FONDAP CRG, Universidad Andrés Bello, Santiago, Chile	Center for Mathematical Modeling and Center for Genome Regulation. Santiago, Chile	Echeverría C et al
EPI_ISL_626565	2020-07-26	Laboratorio de Biología Molecular, Facultad de Medicina, Universidad de Atacama, Copiapo, Chile/ FONDAP CRG, Universidad Andrés Bello, Santiago, Chile	Center for Mathematical Modeling and Center for Genome Regulation. Santiago, Chile	Echeverría C et al
EPI_ISL_560600	2020-07-26	Hospital	National Reference Center for Viruses of Respiratory Infections, Institut Pasteur, Paris	Sylvie Behillil et al
EPI_ISL_512831	2020-07-26	National Public Health Laboratory, National Centre for Infectious Diseases	National Public Health Laboratory, National Centre for Infectious Diseases	Mak TM et al
EPI_ISL_707938	2020-07-26	Pamukkale University Hospital	Pamukkale University Department of Medical Genetics	Onur TOKGUN et al. et al
EPI_ISL_717899	2020-07-27	LACEN RJ - Noel Nutels	Bioinformatics Laboratory / LNCC	Carolina M Voloch et al
EPI_ISL_717902	2020-07-27	LACEN RJ - Noel Nutels	Bioinformatics Laboratory / LNCC	Carolina M Voloch et al
EPI_ISL_729848	2020-07-27	Laboratorio Central de Saude Publica do Estado do Rio Grande do Sul (LACEN-RS)	Laboratory of Respiratory Viruses and Measles, Oswaldo Cruz Institute, FIOCRUZ	Paola Resende et al
EPI_ISL_614384	2020-07-27	Molecular diagnostic unit for viral haemorrhagic fevers and emerging viruses, Bouaké CHU Laboratory	Project group Epidemology of Highly Pathogenic Microorganisms, Robert Koch-Institute	Chantal Akoua-Koffi et al

EPI_ISL_541336	2020-07-27	The National Institute of Public Health	State Veterinary Institute Prague	Nagy et al
EPI_ISL_541337	2020-07-27	The National Institute of Public Health	State Veterinary Institute Prague	Nagy et al
EPI_ISL_545580	2020-07-27	The National Institute of Public Health	State Veterinary Institute Prague	Nagy et al
EPI_ISL_618239	2020-07-27	Department of Virus and Microbiological Special Diagnostics, Statens Serum Institut, Denmark	Albertsen lab, Department of Chemistry and Bioscience, Aalborg University, Denmark	Danish Covid-19 Genome Consortia et al
EPI_ISL_618253	2020-07-27	Department of Virus and Microbiological Special Diagnostics, Statens Serum Institut, Denmark	Albertsen lab, Department of Chemistry and Bioscience, Aalborg University, Denmark	Danish Covid-19 Genome Consortia et al
EPI_ISL_618233	2020-07-27	Department of Virus and Microbiological Special Diagnostics, Statens Serum Institut, Denmark	Albertsen lab, Department of Chemistry and Bioscience, Aalborg University, Denmark	Danish Covid-19 Genome Consortia et al
EPI_ISL_561055	2020-07-27	MRCG at LSHTM Genomics lab	MRCG at LSHTM Genomics lab	Abdul Karim sesay et al
EPI_ISL_561064	2020-07-27	MRCG at LSHTM Genomics lab	MRCG at LSHTM Genomics lab	Abdul Karim sesay et al
EPI_ISL_538504	2020-07-27	National Institute of Health Research and Development	National Institute of Health Research and Development	Pawestri et al
EPI_ISL_637000	2020-07-27	Department of Infectious Diseases and Immunology, National Hospital Organization Nagoya Medical Center	Clinical Research Center, National Hospital Organization Nagoya Medical Center	Yoshihiro Nakata et al
EPI_ISL_636841	2020-07-27	Lithuanian University of Health Sciences Hospital, Department of Laboratory Medicine	Lithuanian University of Health Sciences, Molecular cardiology lab.	Lukas Zemaitis et al
EPI_ISL_718267	2020-07-27	Institute for Medical Research, Infectious Disease Research Centre, National Institutes of Health, Ministry of Health Malaysia	Institute for Medical Research, Infectious Disease Research Centre, National Institutes of Health, Ministry of Health Malaysia	Suppiah J et al
EPI_ISL_596449	2020-07-27	Institute for Medical Research, Infectious Disease Research Centre, National Institutes of Health, Ministry of Health Malaysia	Institute for Medical Research, Infectious Disease Research Centre, National Institutes of Health, Ministry of Health Malaysia	Suppiah J et al
EPI_ISL_596520	2020-07-27	Palestinian Ministry of Health	Molecular Genetics Lab	Nouar Qutob et al
EPI_ISL_708802	2020-07-27	Regional medical sciences center 6 chonburi	National Institute of Health, Department of Medical Sciences, Ministry of Public Health, Thailand	Pilailuk Okada et al
EPI_ISL_707937	2020-07-27	Pamukkale University Hospital	Pamukkale University Department of Medical Genetics	Onur TOKGUN et al. et al
EPI_ISL_717786	2020-07-28	LACEN RJ - Noel Nutels	Bioinformatics Laboratory / LNCC	Carolina M Voloch et al
EPI_ISL_729845	2020-07-28	Laboratorio Central de Saude Publica do Estado do Rio Grande do Sul (LACEN-RS)	Laboratory of Respiratory Viruses and Measles, Oswaldo Cruz Institute, FIOCRUZ	Paola Resende et al
EPI_ISL_729851	2020-07-28	Laboratorio Central de Saude Publica do Estado do Rio Grande do Sul (LACEN-RS)	Laboratory of Respiratory Viruses and Measles, Oswaldo Cruz Institute, FIOCRUZ	Paola Resende et al
EPI_ISL_591527	2020-07-28	Medicina Norte U Chile - Servicio Medico Legal	Center for Mathematical Modeling and Center for Genome Regulation. Santiago, Chile	Gaggero A et al
EPI_ISL_681839	2020-07-28	Molecular diagnostic unit for viral haemorrhagic fevers and emerging viruses, Bouaké CHU Laboratory	Project group Epidemology of Highly Pathogenic Microorganisms, Robert Koch-Institute	Chantal Akoua-Koffi et al
EPI_ISL_515758	2020-07-28	NHLS-IALCH	KRISP, KZN Research Innovation and Sequencing Platform	Giandhari J et al
EPI_ISL_534247	2020-07-28	Universitetssjukhuset i Linkoping	The Public Health Agency of Sweden	Anna-Malin Linde et al
EPI_ISL_576147	2020-07-28	Department of Respiratory & Other Viral Infections of L.V. Gromashevsky Institute of Epidemiology & Infectious Diseases NAMS of Ukraine	Department of Respiratory & Other Viral Infections of L.V. Gromashevsky Institute of Epidemiology & Infectious Diseases NAMS of Ukraine, JSC "Farmak"	Alla Mironenko et al
EPI_ISL_576146	2020-07-28	Department of Respiratory & Other Viral Infections of L.V. Gromashevsky Institute of Epidemiology & Infectious Diseases NAMS of Ukraine	Department of Respiratory & Other Viral Infections of L.V. Gromashevsky Institute of Epidemiology & Infectious Diseases NAMS of Ukraine, JSC "Farmak"	Alla Mironenko et al
EPI_ISL_717794	2020-07-29	LACEN RJ - Noel Nutels	Bioinformatics Laboratory / LNCC	Carolina M Voloch et al
EPI_ISL_717906	2020-07-29	LACEN RJ - Noel Nutels	Bioinformatics Laboratory / LNCC	Carolina M Voloch et al
EPI_ISL_717789	2020-07-29	LACEN RJ - Noel Nutels	Bioinformatics Laboratory / LNCC	Carolina M Voloch et al
EPI_ISL_717909	2020-07-29	LACEN RJ - Noel Nutels	Bioinformatics Laboratory / LNCC	Carolina M Voloch et al
EPI_ISL_717785	2020-07-29	LACEN RJ - Noel Nutels	Bioinformatics Laboratory / LNCC	Carolina M Voloch et al
EPI_ISL_626549	2020-07-29	Laboratorio de Biología Molecular, Facultad de Medicina, Universidad de Atacama, Copiapo, Chile/ FONDAP CRG, Universidad Andrés Bello, Santiago, Chile	Center for Mathematical Modeling and Center for Genome Regulation. Santiago, Chile	Echeverría C et al
EPI_ISL_523137	2020-07-29	Dutch COVID-19 response team	Erasmus Medical Center	Bas Oude Munnink et al

EPI_ISL_549070	2020-07-29	Medical Microbiology Unit, Department for Laboratory Medicine, Drammen Hospital, Vestre Viken Health Trust,	Norwegian Institute of Public Health, Department of Virology	Kathrine Stene-Johansen et al
EPI_ISL_678164	2020-07-29	Pathogen Genomics Lab King Abdullah University of Science and Technology(KAUST)	Pathogen Genomics Lab King Abdullah University of Science and Technology(KAUST)	Sara Mfarrej et al
EPI_ISL_708184	2020-07-29	Pamukkale University Hospital	Pamukkale University Department of Medical Genetics	Onur TOKGUN et al. et al
EPI_ISL_626564	2020-07-30	Laboratorio de Biología Molecular, Facultad de Medicina, Universidad de Atacama, Copiapo, Chile/ FONDAP CRG, Universidad Andrés Bello, Santiago, Chile	Center for Mathematical Modeling and Center for Genome Regulation. Santiago, Chile	Echeverría C et al
EPI_ISL_561008	2020-07-30	MRCG at LSHTM Genomics lab	MRCG at LSHTM Genomics lab	Abdul Karim sesay et al
EPI_ISL_576386	2020-07-30	National Institute of Health Research and Development	National Institute of Health Research and Development	Pawestri et al
EPI_ISL_538512	2020-07-30	Balai Penelitian dan Pengembangan Biomedis Papua	National Institute of Health Research and Development	Pawestri et al
EPI_ISL_576145	2020-07-30	RSA Universitas Gadjah Mada	Genetics Working Group (Pokja Genetik) Faculty of Medicine, Public Health and Nursing Universitas Gadjah Mada (FK-KMK UGM); Disease Investigation Center Wates Ministry of Agriculture Indonesia; Department of Microbiology FK-KMK UGM; Laboratorium Diagnostik Yayasan Tahija World Mosquito Program (WMP) Yogyakarta Center for Tropical Medicine FK-KMK UGM; Integrated Research Center FK-KMK UGM; Department of Computer Science and Electronics FMIPA UGM	Gunadi et al
EPI_ISL_636843	2020-07-30	Lithuanian University of Health Sciences Hospital, Department of Laboratory Medicine	Lithuanian University of Health Sciences, Molecular cardiology lab.	Lukas Zemaitis et al
EPI_ISL_636844	2020-07-30	Lithuanian University of Health Sciences Hospital, Department of Laboratory Medicine	Lithuanian University of Health Sciences, Molecular cardiology lab.	Lukas Zemaitis et al
EPI_ISL_596266	2020-07-30	WHO National Influenza Centre Russian Federation	WHO National Influenza Centre Russian Federation	Andrey Komissarov et al
EPI_ISL_708800	2020-07-30	Regional medical sciences center 6 chonburi	National Institute of Health, Department of Medical Sciences, Ministry of Public Health, Thailand	Pilailuk Okada et al
EPI_ISL_708190	2020-07-30	Pamukkale University Hospital	Pamukkale University Department of Medical Genetics	Onur TOKGUN et al. et al
EPI_ISL_708191	2020-07-30	Pamukkale University Hospital	Pamukkale University Department of Medical Genetics	Onur TOKGUN et al. et al
EPI_ISL_729583	2020-07-31	A. Krumbholz, Labor Dr. Krause und Kollegen MVZ GmbH, Kiel	Charité Universitätsmedizin Berlin, Institut für Virologie	Victor M Corman et al
EPI_ISL_522875	2020-07-31	Instituto Nacional de Medicina Genomica	Instituto Nacional de Medicina Genomica	Hidalgo-Miranda A et al
EPI_ISL_660543	2020-07-31	Laboratory Medicine	Department of Laboratory Medicine, Lin-Kou Chang Gung Memorial Hospital, Taoyuan, Taiwan	Kuo-Chien Tsao et al
EPI_ISL_613780	2020-07-31	Florida Bureau of Public Health Laboratories	Florida Bureau of Public Health Laboratories	Sarah Schmedes et al
EPI_ISL_582513	2020-07-31	Department of Respiratory and other Viral Infections of L.V.Gromashevsky Institute of Epidemiology & Infectious Diseases NAMS of Ukraine	Department of Respiratory and other Viral Infections of L.V.Gromashevsky Institute of Epidemiology & Infectious Diseases NAMS of Ukraine, JSC "Farmak"	Alla Mironenko et al
EPI_ISL_717908	2020-08-01	LACEN RJ - Noel Nutels	Bioinformatics Laboratory / LNCC	Carolina M Voloch et al
EPI_ISL_729584	2020-08-01	A. Krumbholz, Labor Dr. Krause und Kollegen MVZ GmbH, Kiel	Charité Universitätsmedizin Berlin, Institut für Virologie	Victor M Corman et al
EPI_ISL_525371	2020-08-01	National Virus Reference Laboratory	National Virus Reference Laboratory	Michael Carr et al
EPI_ISL_644982	2020-08-01	Department of Infectious Diseases, Keio University School of Medicine, Tokyo, Japan	Center for Medical Genetics, Keio University School of Medicine, Tokyo, Japan	Kenjiro Kosaki et al
EPI_ISL_522878	2020-08-01	Instituto Nacional de Medicina Genomica	Instituto Nacional de Medicina Genomica	Hidalgo-Miranda A et al
EPI_ISL_526745	2020-08-01	Center for Laboratory Control of Infectious Diseases, Korea Centers for Diseases Control and Prevention	Center for Laboratory Control of Infectious Diseases, Korea Centers for Diseases Control and Prevention	Junyoung Kim et al
EPI_ISL_667221	2020-08-01	OHSU Lab Services Molecular Microbiology Lab	Oregon SARS-CoV-2 Genome Sequencing Center	Brendan L. O'Connell et al
EPI_ISL_682262	2020-08-02	HOSPITAL SAN JUAN DE DIOS	Inciensa, Instituto Costarricense de Investigación y Enseñanza en Nutrición y Salud	Francisco Duarte et al
EPI_ISL_649172	2020-08-02	Laboratorio de Investigaciones de Baney	University Hospital Basel, Clinical Bacteriology	Carlos Cortes et al
EPI_ISL_648337	2020-08-02	Laboratorio de Investigaciones de Baney	University Hospital Basel, Clinical Bacteriology	Carlos Cortes et al
EPI_ISL_636803	2020-08-02	National Centre for Disease control (NCDC)	NCDC/CSIR-IGIB	Mahesh S. Dhar1* et al

EPI_ISL_636825	2020-08-02	National Centre for Disease control (NCDC)	NCDC/CSIR-IGIB	Mahesh S. Dhar1* et al
EPI_ISL_523700	2020-08-02	Dutch COVID-19 response team	Erasmus Medical Center	Bas Oude Munnink et al
EPI_ISL_534232	2020-08-02	Capio S:t Gorans sjukhus	The Public Health Agency of Sweden	Anna-Malin Linde et al
EPI_ISL_592443	2020-08-03	Microbiological Diagnostic Unit - Public Health Laboratory (MDU-PHL)	MDU-PHL	Seemann T. et al
EPI_ISL_583887	2020-08-03	Austrian Agency for Health and Food Safety (AGES)	Bergthaler laboratory, CeMM Research Center for Molecular Medicine of the Austrian Academy of Sciences	Alexandra Popa et al
EPI_ISL_583888	2020-08-03	Austrian Agency for Health and Food Safety (AGES)	Bergthaler laboratory, CeMM Research Center for Molecular Medicine of the Austrian Academy of Sciences	Alexandra Popa et al
EPI_ISL_583890	2020-08-03	Austrian Agency for Health and Food Safety (AGES)	Bergthaler laboratory, CeMM Research Center for Molecular Medicine of the Austrian Academy of Sciences	Alexandra Popa et al
EPI_ISL_583891	2020-08-03	Austrian Agency for Health and Food Safety (AGES)	Bergthaler laboratory, CeMM Research Center for Molecular Medicine of the Austrian Academy of Sciences	Alexandra Popa et al
EPI_ISL_583892	2020-08-03	Austrian Agency for Health and Food Safety (AGES)	Bergthaler laboratory, CeMM Research Center for Molecular Medicine of the Austrian Academy of Sciences	Alexandra Popa et al
EPI_ISL_717863	2020-08-03	Laboratorio de Virologia Molecular / UFRJ	Bioinformatics Laboratory / LNCC	Carolina M Voloch et al
EPI_ISL_717864	2020-08-03	Laboratorio de Virologia Molecular / UFRJ	Bioinformatics Laboratory / LNCC	Carolina M Voloch et al
EPI_ISL_717865	2020-08-03	Laboratorio de Virologia Molecular / UFRJ	Bioinformatics Laboratory / LNCC	Carolina M Voloch et al
EPI_ISL_717866	2020-08-03	Laboratorio de Virologia Molecular / UFRJ	Bioinformatics Laboratory / LNCC	Carolina M Voloch et al
EPI_ISL_717959	2020-08-03	Laboratorio de Virologia Molecular / UFRJ	Bioinformatics Laboratory / LNCC	Carolina M Voloch et al
EPI_ISL_617424	2020-08-03	Department of Virus and Microbiological Special Diagnostics, Statens Serum Institut, Denmark	Albertsen lab, Department of Chemistry and Bioscience, Aalborg University, Denmark	Danish Covid-19 Genome Consortia et al
EPI_ISL_532047	2020-08-03	Lighthouse Lab in Glasgow	Wellcome Sanger Institute for the COVID-19 Genomics UK (COG-UK) consortium	Harper VanSteenhouse et al
EPI_ISL_560611	2020-08-03	Hospital	National Reference Center for Viruses of Respiratory Infections, Institut Pasteur, Paris	Sylvie Behillil et al
EPI_ISL_729975	2020-08-03	Nigeria Centre for Disease Control (NCDC)	African Centre of Excellence for Genomics of Infectious Diseases (ACEGID), Redeemer's University, Ede, Osun State, Nigeria	Oluniyi P.E. et al et al
EPI_ISL_576148	2020-08-03	Department of Respiratory & Other Viral Infections of L.V. Gromashevsky Institute of Epidemiology & Infectious Diseases NAMS of Ukraine	Department of Respiratory & Other Viral Infections of L.V. Gromashevsky Institute of Epidemiology & Infectious Diseases NAMS of Ukraine, JSC "Farmak"	Alla Mironenko et al
EPI_ISL_636514	2020-08-04	Dutch COVID-19 response team	National Institute for Public Health and the Environment (RIVM)	Adam Meijer et al
EPI_ISL_625472	2020-08-04	Child Health Research Foundation	Child Health Research Foundation	Senjuti Saha et al
EPI_ISL_540512	2020-08-04	Department of Clinical Microbiology	GIGA Medical Genomics	Keith Durkin et al
EPI_ISL_540515	2020-08-04	Department of Clinical Microbiology	GIGA Medical Genomics	Keith Durkin et al
EPI_ISL_717867	2020-08-04	Laboratorio de Virologia Molecular / UFRJ	Bioinformatics Laboratory / LNCC	Carolina M Voloch et al
EPI_ISL_527809	2020-08-04	Institute of Microbiology, Universidad San Francisco de Quito	Institute of Microbiology, Universidad San Francisco de Quito	Belén Prado-Vivar et al
EPI_ISL_561236	2020-08-04	MRCG at LSHTM Genomics lab	MRCG at LSHTM Genomics lab	Abdul Karim sesay et al
EPI_ISL_561237	2020-08-04	MRCG at LSHTM Genomics lab	MRCG at LSHTM Genomics lab	Abdul Karim sesay et al
EPI_ISL_561289	2020-08-04	MRCG at LSHTM Genomics lab	MRCG at LSHTM Genomics lab	Abdul Karim sesay et al
EPI_ISL_581449	2020-08-04	CSIR-Indian Institute of Chemical Biology, MEDICA Supercpecialty Hospital Kolkata	CSIR-Indian Institute of Chemical Biology, MEDICA Supercpecialty Hospital Kolkata	Sujay Krishna Maity et al
EPI_ISL_644988	2020-08-04	Department of Infectious Diseases, Keio University School of Medicine, Tokyo, Japan	Center for Medical Genetics, Keio University School of Medicine, Tokyo, Japan	Kenjiro Kosaki et al
EPI_ISL_636848	2020-08-04	Lithuanian University of Health Sciences Hospital, Department of Laboratory Medicine	Lithuanian University of Health Sciences, Molecular cardiology lab.	Lukas Zemaitis et al
EPI_ISL_636849	2020-08-04	Lithuanian University of Health Sciences Hospital, Department of Laboratory Medicine	Lithuanian University of Health Sciences, Molecular cardiology lab.	Lukas Zemaitis et al

EPI_ISL_729976	2020-08-04	Nigeria Centre for Disease Control (NCDC)	African Centre of Excellence for Genomics of Infectious Diseases (ACEGID), Redeemer's University, Ede, Osun State, Nigeria	Oluniyi P.E. et al et al
EPI_ISL_677722	2020-08-04	General Hospital - Ohrid	Research Center for Genetic Engineering and Biotechnology "Georgi D. Efremov" , Macedonian Academy of Sciences and Arts	RCGEB - MASA et al
EPI_ISL_677723	2020-08-04	Institute for Lung Diseases in Children - Skopje	Research Center for Genetic Engineering and Biotechnology "Georgi D. Efremov" , Macedonian Academy of Sciences and Arts	RCGEB - MASA et al
EPI_ISL_512921	2020-08-04	Pathogen Genomics Lab King Abdullah University of Science and Technology(KAUST)	Pathogen Genomics Lab King Abdullah University of Science and Technology(KAUST)	Fadwa Alofi et al
EPI_ISL_512922	2020-08-04	Pathogen Genomics Lab King Abdullah University of Science and Technology(KAUST)	Pathogen Genomics Lab King Abdullah University of Science and Technology(KAUST)	Fadwa Alofi et al
EPI_ISL_512923	2020-08-04	Pathogen Genomics Lab King Abdullah University of Science and Technology(KAUST)	Pathogen Genomics Lab King Abdullah University of Science and Technology(KAUST)	Fadwa Alofi et al
EPI_ISL_512924	2020-08-04	Pathogen Genomics Lab King Abdullah University of Science and Technology(KAUST)	Pathogen Genomics Lab King Abdullah University of Science and Technology(KAUST)	Fadwa Alofi et al
EPI_ISL_678489	2020-08-04	Veterinary Specialized Institute "Sabac", Serbia	Veterinary Specialized Institute "Kraljevo", Serbia	Vidanovic et al
EPI_ISL_516580	2020-08-04	Viollier AG	Department of Biosystems Science and Engineering, ETH Zürich	Christian Beisel et al
EPI_ISL_636515	2020-08-05	Dutch COVID-19 response team	National Institute for Public Health and the Environment (RIVM)	Adam Meijer et al
EPI_ISL_591657	2020-08-05	Victorian Infectious Diseases Reference Laboratory (VIDRL)	VIDRL and MDU-PHL	Caly L. et al
EPI_ISL_717868	2020-08-05	Laboratorio de Virologia Molecular / UFRJ	Bioinformatics Laboratory / LNCC	Carolina M Voloch et al
EPI_ISL_681840	2020-08-05	Molecular diagnostic unit for viral haemorrhagic fevers and emerging viruses, Bouaké CHU Laboratory	Project group Epidemology of Highly Pathogenic Microorganisms, Robert Koch-Institute	Chantal Akoua-Koffi et al
EPI_ISL_539786	2020-08-05	Institute of Microbiology, Universidad San Francisco de Quito	Institute of Microbiology, Universidad San Francisco de Quito	Andrea Macias et al
EPI_ISL_648379	2020-08-05	Laboratorio de Investigaciones de Baney	University Hospital Basel, Clinical Bacteriology	Carlos Cortes et al
EPI_ISL_644990	2020-08-05	Department of Infectious Diseases, Keio University School of Medicine, Tokyo, Japan	Center for Medical Genetics, Keio University School of Medicine, Tokyo, Japan	Kenjiro Kosaki et al
EPI_ISL_534204	2020-08-05	Latvijas Infektoloģijas centrs	Latvian Biomedical Research and Study Centre	Ivars Silamiķelis et al
EPI_ISL_636850	2020-08-05	Lithuanian University of Health Sciences Hospital, Department of Laboratory Medicine	Lithuanian University of Health Sciences, Molecular cardiology lab.	Lukas Zemaitis et al
EPI_ISL_677724	2020-08-05	Clinical Hospital - Shtip	Research Center for Genetic Engineering and Biotechnology "Georgi D. Efremov" , Macedonian Academy of Sciences and Arts	RCGEB - MASA et al
EPI_ISL_677725	2020-08-05	General Hospital - Ohrid	Research Center for Genetic Engineering and Biotechnology "Georgi D. Efremov" , Macedonian Academy of Sciences and Arts	RCGEB - MASA et al
EPI_ISL_677726	2020-08-05	General Hospital - Ohrid	Research Center for Genetic Engineering and Biotechnology "Georgi D. Efremov" , Macedonian Academy of Sciences and Arts	RCGEB - MASA et al
EPI_ISL_654946	2020-08-05	Klinisk mikrobiologi	The Public Health Agency of Sweden	Anna-Malin Linde et al
EPI_ISL_707936	2020-08-05	Pamukkale University Hospital	Pamukkale University Department of Medical Genetics	Onur TOKGUN et al. et al
EPI_ISL_636516	2020-08-06	Dutch COVID-19 response team	National Institute for Public Health and the Environment (RIVM)	Adam Meijer et al
EPI_ISL_729858	2020-08-06	Laboratorio Central de Saude Publica do Estado do Rio Grande do Sul (LACEN-RS)	Laboratory of Respiratory Viruses and Measles, Oswaldo Cruz Institute, FIOCRUZ	Paola Resende et al
EPI_ISL_653745	2020-08-06	Instituto Nacional de Salud, Bogotá, Colombia	Instituto Nacional de Salud, Bogotá, Colombia	Katherine Laiton-Donato et al
EPI_ISL_547966	2020-08-06	The National Institute of Public Health	State Veterinary Institute Prague	Nagy et al
EPI_ISL_538510	2020-08-06	RSUD Ulin Banjarmasin South Kalimantan	National Institute of Health Research and Development	Pawestri et al
EPI_ISL_534205	2020-08-06	Latvijas Infektoloģijas centrs	Latvian Biomedical Research and Study Centre	Ivars Silamiķelis et al
EPI_ISL_522942	2020-08-06	Instituto Nacional de Medicina Genómica	Instituto Nacional de Medicina Genómica	Hidalgo-Miranda A et al
EPI_ISL_654172	2020-08-06	Hospital General Universitario Gregorio Marañón	SeqCOVID-SPAIN consortium/IBV(CSIC)	Darío García de Viedma et al
EPI_ISL_654947	2020-08-06	Klinisk mikrobiologi	The Public Health Agency of Sweden	Anna-Malin Linde et al
EPI_ISL_654819	2020-08-06	Department of Respiratory and other Viral Infections of L.V.Gromashevsky Institute of Epidemiology & Infectious Diseases NAMS of Ukraine	Department of Respiratory and other Viral Infections of L.V.Gromashevsky Institute of Epidemiology & Infectious Diseases NAMS of Ukraine, JSC "Farmak"	Alla Mironenko et al

EPI_ISL_576149	2020-08-06	Department of Respiratory & Other Viral Infections of L.V. Gromashevsky Institute of Epidemiology & Infectious Diseases NAMS of Ukraine	Department of Respiratory & Other Viral Infections of L.V. Gromashevsky Institute of Epidemiology & Infectious Diseases NAMS of Ukraine, JSC "Farmak"	Alla Mironenko et al
EPI_ISL_636517	2020-08-07	Dutch COVID-19 response team	National Institute for Public Health and the Environment (RIVM)	Adam Meijer et al
EPI_ISL_700338	2020-08-07	Child Health Research Foundation	Child Health Research Foundation	Senjuti Saha et al
EPI_ISL_540541	2020-08-07	Department of Clinical Microbiology	GIGA Medical Genomics	Keith Durkin et al
EPI_ISL_540542	2020-08-07	Department of Clinical Microbiology	GIGA Medical Genomics	Keith Durkin et al
EPI_ISL_729852	2020-08-07	Laboratorio Central de Saude Publica do Estado do Rio Grande do Sul (LACEN-RS)	Laboratory of Respiratory Viruses and Measles, Oswaldo Cruz Institute, FIOCRUZ	Paola Resende et al
EPI_ISL_660447	2020-08-07	Laboratoire de Microbiologie CHU Souro Sanou	Centre Muraz	Abdoul-Salam Ouedraogo et al
EPI_ISL_626556	2020-08-07	Laboratorio de Biología Molecular, Facultad de Medicina, Universidad de Atacama, Copiapo, Chile/ FONDAP CRG, Universidad Andrés Bello, Santiago, Chile	Center for Mathematical Modeling and Center for Genome Regulation. Santiago, Chile	Echeverría C et al
EPI_ISL_626557	2020-08-07	Laboratorio de Biología Molecular, Facultad de Medicina, Universidad de Atacama, Copiapo, Chile/ FONDAP CRG, Universidad Andrés Bello, Santiago, Chile	Center for Mathematical Modeling and Center for Genome Regulation. Santiago, Chile	Echeverría C et al
EPI_ISL_626559	2020-08-07	Laboratorio de Biología Molecular, Facultad de Medicina, Universidad de Atacama, Copiapo, Chile/ FONDAP CRG, Universidad Andrés Bello, Santiago, Chile	Center for Mathematical Modeling and Center for Genome Regulation. Santiago, Chile	Echeverría C et al
EPI_ISL_626560	2020-08-07	Laboratorio de Biología Molecular, Facultad de Medicina, Universidad de Atacama, Copiapo, Chile/ FONDAP CRG, Universidad Andrés Bello, Santiago, Chile	Center for Mathematical Modeling and Center for Genome Regulation. Santiago, Chile	Echeverría C et al
EPI_ISL_626563	2020-08-07	Laboratorio de Biología Molecular, Facultad de Medicina, Universidad de Atacama, Copiapo, Chile/ FONDAP CRG, Universidad Andrés Bello, Santiago, Chile	Center for Mathematical Modeling and Center for Genome Regulation. Santiago, Chile	Echeverría C et al
EPI_ISL_653746	2020-08-07	Instituto Nacional de Salud, Bogotá, Colombia	Instituto Nacional de Salud, Bogotá, Colombia	Katherine Laiton-Donato et al
EPI_ISL_653747	2020-08-07	Instituto Nacional de Salud, Bogotá, Colombia	Instituto Nacional de Salud, Bogotá, Colombia	Katherine Laiton-Donato et al
EPI_ISL_547967	2020-08-07	The National Institute of Public Health	State Veterinary Institute Prague	Nagy et al
EPI_ISL_569055	2020-08-07	MEPHI, Aix Marseille University	MEPHI, Aix Marseille University	Anthony LEVASSEUR et al
EPI_ISL_561292	2020-08-07	MRCG at LSHTM Genomics lab	MRCG at LSHTM Genomics lab	Abdul Karim sesay et al
EPI_ISL_636852	2020-08-07	Lithuanian University of Health Sciences Hospital, Department of Laboratory Medicine	Lithuanian University of Health Sciences, Molecular cardiology lab.	Lukas Zemaitis et al
EPI_ISL_636854	2020-08-07	Lithuanian University of Health Sciences Hospital, Department of Laboratory Medicine	Lithuanian University of Health Sciences, Molecular cardiology lab.	Lukas Zemaitis et al
EPI_ISL_729977	2020-08-07	Nigeria Centre for Disease Control (NCDC)	African Centre of Excellence for Genomics of Infectious Diseases (ACEGID), Redeemer's University, Ede, Osun State, Nigeria	Oluniyi P.E. et al et al
EPI_ISL_517661	2020-08-07	Academic Hospital Paramaribo	Erasmus Medical Center	Bas Oude Munnink et al
EPI_ISL_517662	2020-08-07	Academic Hospital Paramaribo	Erasmus Medical Center	Bas Oude Munnink et al
EPI_ISL_721678	2020-08-07	Viollier AG	Department of Biosystems Science and Engineering, ETH Zürich	Christian Beisel et al
EPI_ISL_582510	2020-08-07	Department of Respiratory and other Viral Infections of L.V.Gromashevsky Institute of Epidemiology & Infectious Diseases NAMS of Ukraine	Department of Respiratory and other Viral Infections of L.V.Gromashevsky Institute of Epidemiology & Infectious Diseases NAMS of Ukraine, JSC "Farmak"	Alla Mironenko et al
EPI_ISL_582512	2020-08-07	Department of Respiratory and other Viral Infections of L.V.Gromashevsky Institute of Epidemiology & Infectious Diseases NAMS of Ukraine	Department of Respiratory and other Viral Infections of L.V.Gromashevsky Institute of Epidemiology & Infectious Diseases NAMS of Ukraine, JSC "Farmak"	Alla Mironenko et al
EPI_ISL_682267	2020-08-08	HOSPITAL SAN JUAN DE DIOS	Inciensa, Instituto Costarricense de Investigación y Enseñanza en Nutrición y Salud	Francisco Duarte et al
EPI_ISL_682268	2020-08-08	HOSPITAL SAN JUAN DE DIOS	Inciensa, Instituto Costarricense de Investigación y Enseñanza en Nutrición y Salud	Francisco Duarte et al
EPI_ISL_560317	2020-08-08	UMMC-Health	WHO National Influenza Centre Russian Federation	Andrey Komissarov et al
EPI_ISL_654339	2020-08-08	Hospital General Universitario Gregorio Marañón	SeqCOVID-SPAIN consortium/IBV(CSIC)	Darío García de Viedma et al

EPI_ISL_577750	2020-08-08	Dutch COVID-19 response team	Erasmus Medical Center	OH consortium et al
EPI_ISL_577751	2020-08-08	Dutch COVID-19 response team	Erasmus Medical Center	OH consortium et al
EPI_ISL_577753	2020-08-08	Dutch COVID-19 response team	Erasmus Medical Center	OH consortium et al
EPI_ISL_577754	2020-08-08	Dutch COVID-19 response team	Erasmus Medical Center	OH consortium et al
EPI_ISL_729802	2020-08-09	Laboratorio Central de Saude Publica do Estado do Rio Grande do Sul (LACEN-RS)	Laboratory of Respiratory Viruses and Measles, Oswaldo Cruz Institute, FIOCRUZ	Paola Resende et al
EPI_ISL_663581	2020-08-10	Microbiological Diagnostic Unit - Public Health Laboratory (MDU-PHL)	MDU-PHL	Seemann T. et al
EPI_ISL_540566	2020-08-10	Department of Clinical Microbiology	GIGA Medical Genomics	Keith Durkin et al
EPI_ISL_626558	2020-08-10	Laboratorio de Biología Molecular, Facultad de Medicina, Universidad de Atacama, Copiapo, Chile/ FONDAP CRG, Universidad Andrés Bello, Santiago, Chile	Center for Mathematical Modeling and Center for Genome Regulation. Santiago, Chile	Echeverría C et al
EPI_ISL_683367	2020-08-10	CNR Virus des Infections Respiratoires - France SUD	CNR Virus des Infections Respiratoires - France SUD	Antonin Bal et al
EPI_ISL_569171	2020-08-10	MEPHI, Aix Marseille University	MEPHI, Aix Marseille University	Anthony LEVASSEUR et al
EPI_ISL_534201	2020-08-10	Centrālā laboratorija	Latvian Biomedical Research and Study Centre	Ivars Silamiķelis et al
EPI_ISL_549084	2020-08-10	Akershus University Hospital, Department for Microbiology and Infectious Disease Control	Norwegian Institute of Public Health, Department of Virology	Kathrine Stene-Johansen et al
EPI_ISL_523913	2020-08-10	Viollier AG	Department of Biosystems Science and Engineering, ETH Zürich	Christian Beisel et al
EPI_ISL_682269	2020-08-11	HOSPITAL SAN JUAN DE DIOS	Inciensa, Instituto Costarricense de Investigación y Enseñanza en Nutrición y Salud	Francisco Duarte et al
EPI_ISL_636519	2020-08-11	Dutch COVID-19 response team	National Institute for Public Health and the Environment (RIVM)	Adam Meijer et al
EPI_ISL_636520	2020-08-11	Dutch COVID-19 response team	National Institute for Public Health and the Environment (RIVM)	Adam Meijer et al
EPI_ISL_636521	2020-08-11	Dutch COVID-19 response team	National Institute for Public Health and the Environment (RIVM)	Adam Meijer et al
EPI_ISL_531693	2020-08-11	Lighthouse Lab in Glasgow	Wellcome Sanger Institute for the COVID-19 Genomics UK (COG-UK) consortium	Harper VanSteenhouse et al
EPI_ISL_678238	2020-08-11	Pathogen Genomics Lab King Abdullah University of Science and Technology(KAUST)	Pathogen Genomics Lab King Abdullah University of Science and Technology(KAUST)	Muhammad Shuaib et al
EPI_ISL_526746	2020-08-11	Center for Laboratory Control of Infectious Diseases, Korea Centers for Diseases Control and Prevention	Center for Laboratory Control of Infectious Diseases, Korea Centers for Diseases Control and Prevention	Junyoung Kim et al
EPI_ISL_523885	2020-08-11	Viollier AG	Department of Biosystems Science and Engineering, ETH Zürich	Christian Beisel et al
EPI_ISL_534336	2020-08-11	Department of Laboratory Medicine, National Taiwan University Hospital	Microbial Genomics Core Lab, National Taiwan University Centers of Genomic and Precision Medicine	Shiou-Hwei Yeh et al
EPI_ISL_700339	2020-08-12	Child Health Research Foundation	Child Health Research Foundation	Senjuti Saha et al
EPI_ISL_729853	2020-08-12	Laboratorio Central de Saude Publica do Estado do Rio Grande do Sul (LACEN-RS)	Laboratory of Respiratory Viruses and Measles, Oswaldo Cruz Institute, FIOCRUZ	Paola Resende et al
EPI_ISL_729849	2020-08-12	Laboratorio Central de Saude Publica do Estado do Rio Grande do Sul (LACEN-RS)	Laboratory of Respiratory Viruses and Measles, Oswaldo Cruz Institute, FIOCRUZ	Paola Resende et al
EPI_ISL_614386	2020-08-12	Molecular diagnostic unit for viral haemorrhagic fevers and emerging viruses, Bouaké CHU Laboratory	Project group Epidemology of Highly Pathogenic Microorganisms, Robert Koch-Institute	Chantal Akoua-Koffi et al
EPI_ISL_697785	2020-08-12	Institute of Microbiology, Universidad San Francisco de Quito	Institute of Microbiology, Universidad San Francisco de Quito	Belén Prado-Vivar et al
EPI_ISL_648338	2020-08-12	Laboratorio de Investigaciones de Baney	University Hospital Basel, Clinical Bacteriology	Carlos Cortes et al
EPI_ISL_539816	2020-08-12	Queen Mary Hospital	Hong Kong Department of Health	Alan K.L. Tsang et al
EPI_ISL_528465	2020-08-12	National Virus Reference Laboratory	National Virus Reference Laboratory	Michael Carr et al
EPI_ISL_528473	2020-08-12	National Virus Reference Laboratory	National Virus Reference Laboratory	Michael Carr et al
EPI_ISL_529751	2020-08-12	NHLS-IALCH	KRISP, KZN Research Innovation and Sequencing Platform	Giandhari J et al
EPI_ISL_529753	2020-08-12	NHLS-IALCH	KRISP, KZN Research Innovation and Sequencing Platform	Giandhari J et al
EPI_ISL_529761	2020-08-12	NHLS-IALCH	KRISP, KZN Research Innovation and Sequencing Platform	Giandhari J et al

EPI_ISL_729860	2020-08-13	Laboratorio Central de Saude Publica do Estado do Rio Grande do Sul (LACEN-RS)	Laboratory of Respiratory Viruses and Measles, Oswaldo Cruz Institute, FIOCRUZ	Paola Resende et al
EPI_ISL_660493	2020-08-13	Laboratoire de Microbiologie CHU Souro Sanou	Centre Muraz	Abdoul-Salam Ouedraogo et al
EPI_ISL_682274	2020-08-13	HOSPITAL SAN JUAN DE DIOS	Inciensa, Instituto Costarricense de Investigación y Enseñanza en Nutrición y Salud	Francisco Duarte et al
EPI_ISL_539819	2020-08-13	Tuen Mun Hospital	Hong Kong Department of Health	Alan K.L. Tsang et al
EPI_ISL_518820	2020-08-13	Klinik Apotek Dein, Jakarta, Indonesia	Biosafety Level-3 Laboratory, Indonesian Institute of Sciences (LIPI)	Anik Budhi Dharmayanthi et al
EPI_ISL_637110	2020-08-13	Rafik Hariri University Hospital	Microbial Pathogenomics Lab	Georgi Merhi et al
EPI_ISL_718271	2020-08-13	Institute for Medical Research, Infectious Disease Research Centre, National Institutes of Health, Ministry of Health Malaysia	Institute for Medical Research, Infectious Disease Research Centre, National Institutes of Health, Ministry of Health Malaysia	Suppiah J et al
EPI_ISL_718272	2020-08-13	Institute for Medical Research, Infectious Disease Research Centre, National Institutes of Health, Ministry of Health Malaysia	Institute for Medical Research, Infectious Disease Research Centre, National Institutes of Health, Ministry of Health Malaysia	Suppiah J et al
EPI_ISL_576260	2020-08-13	Instituto de Diagnostico y Referencia Epidemiologicos (INDRE)	Instituto de Diagnostico y Referencia Epidemiologicos (INDRE)	Gisela Barrera-Badillo et al
EPI_ISL_579058	2020-08-13	LabTests	Institute of Environmental Science and Research (ESR)	Xiaoyun Ren et al
EPI_ISL_729859	2020-08-14	Laboratorio Central de Saude Publica do Estado do Rio Grande do Sul (LACEN-RS)	Laboratory of Respiratory Viruses and Measles, Oswaldo Cruz Institute, FIOCRUZ	Paola Resende et al
EPI_ISL_729803	2020-08-14	Laboratorio Central de Saude Publica do Estado do Rio Grande do Sul (LACEN-RS)	Laboratory of Respiratory Viruses and Measles, Oswaldo Cruz Institute, FIOCRUZ	Paola Resende et al
EPI_ISL_729854	2020-08-14	Laboratorio Central de Saude Publica do Estado do Rio Grande do Sul (LACEN-RS)	Laboratory of Respiratory Viruses and Measles, Oswaldo Cruz Institute, FIOCRUZ	Paola Resende et al
EPI_ISL_710554	2020-08-14	Sestre Milosrdnice University Hospital Center	Ruđer Boškovic Institute; Forensic Science Centre Ivan Vučetić; University of Zagreb Faculty of Science	Robert Belužić et al
EPI_ISL_525435	2020-08-14	Institute of Microbiology, Universidad San Francisco de Quito	Institute of Microbiology, Universidad San Francisco de Quito	Juan José Guadalupe et al
EPI_ISL_526983	2020-08-14	Biological prevention, army	Biological prevention, army	Seadawy et al
EPI_ISL_526984	2020-08-14	Biological prevention, army	Biological prevention, army	Seadawy et al
EPI_ISL_648339	2020-08-14	Laboratorio de Investigaciones de Baney	University Hospital Basel, Clinical Bacteriology	Carlos Cortes et al
EPI_ISL_610207	2020-08-14	Department of Health Technology and Informatics, The Hong Kong Polytechnic University	Department of Health Technology and Informatics, The Hong Kong Polytechnic University	Siu et al
EPI_ISL_539823	2020-08-14	Communicable Disease Branch	Hong Kong Department of Health	Alan K.L. Tsang et al
EPI_ISL_539817	2020-08-14	Queen Mary Hospital	Hong Kong Department of Health	Alan K.L. Tsang et al
EPI_ISL_637112	2020-08-14	Rafik Hariri University Hospital	Microbial Pathogenomics Lab	Georgi Merhi et al
EPI_ISL_637113	2020-08-14	Rafik Hariri University Hospital	Microbial Pathogenomics Lab	Georgi Merhi et al
EPI_ISL_576119	2020-08-14	Laboratory, The Bio Arte Limited	Laboratory, The Bio Arte Limited	Biazzo et al
EPI_ISL_576263	2020-08-14	Instituto de Diagnostico y Referencia Epidemiologicos (INDRE)	Instituto de Diagnostico y Referencia Epidemiologicos (INDRE)	Gisela Barrera-Badillo et al
EPI_ISL_576275	2020-08-14	Instituto de Diagnostico y Referencia Epidemiologicos (INDRE)	Instituto de Diagnostico y Referencia Epidemiologicos (INDRE)	Gisela Barrera-Badillo et al
EPI_ISL_591006	2020-08-14	Ostfold Hospital Trust - Kalnes, Centre for Laboratory Medicine, Section for gene technology and infection serology	Norwegian Institute of Public Health, Department of Virology	Kathrine Stene-Johansen et al
EPI_ISL_717696	2020-08-14	Trinidad Public Health Laboratory	Carrington Lab, Department of PreClinical Sciences, Faculty of Medical Sciences, The University of the West Indies	Nikita S. D. Sahadeo et al
EPI_ISL_729799	2020-08-15	Laboratorio Central de Saude Publica do Estado do Rio Grande do Sul (LACEN-RS)	Laboratory of Respiratory Viruses and Measles, Oswaldo Cruz Institute, FIOCRUZ	Paola Resende et al
EPI_ISL_729855	2020-08-15	Laboratorio Central de Saude Publica do Estado do Rio Grande do Sul (LACEN-RS)	Laboratory of Respiratory Viruses and Measles, Oswaldo Cruz Institute, FIOCRUZ	Paola Resende et al
EPI_ISL_523959	2020-08-15	Pronto Socorro Municipal de Perus	Instituto Adolfo Lutz, Interdiciplinary Procedures Center, Strategic Laboratory	Claudio Tavares Sacchi et al
EPI_ISL_660499	2020-08-15	Laboratoire de Microbiologie CHU Souro Sanou	Centre Muraz	Abdoul-Salam Ouedraogo et al
EPI_ISL_539790	2020-08-15	Institute of Microbiology, Universidad San Francisco de Quito	Institute of Microbiology, Universidad San Francisco de Quito	Belén Prado-Vivar et al
EPI_ISL_548095	2020-08-15	Middlemore Hospital	Institute of Environmental Science and Research (ESR)	Xiaoyun Ren et al

EPI_ISL_654173	2020-08-15	Hospital General Universitario Gregorio Marañón	SeqCOVID-SPAIN consortium/IBV(CSIC)	Darío García de Viedma et al
EPI_ISL_667211	2020-08-15	OHSU Lab Services Molecular Microbiology Lab	Oregon SARS-CoV-2 Genome Sequencing Center	Brendan L. O'Connell et al
EPI_ISL_729861	2020-08-16	Laboratorio Central de Saude Publica do Estado do Rio Grande do Sul (LACEN-RS)	Laboratory of Respiratory Viruses and Measles, Oswaldo Cruz Institute, FIOCRUZ	Paola Resende et al
EPI_ISL_718276	2020-08-16	Institute for Medical Research, Infectious Disease Research Centre, National Institutes of Health, Ministry of Health Malaysia	Institute for Medical Research, Infectious Disease Research Centre, National Institutes of Health, Ministry of Health Malaysia	Suppiah J et al
EPI_ISL_718277	2020-08-16	Institute for Medical Research, Infectious Disease Research Centre, National Institutes of Health, Ministry of Health Malaysia	Institute for Medical Research, Infectious Disease Research Centre, National Institutes of Health, Ministry of Health Malaysia	Suppiah J et al
EPI_ISL_548146	2020-08-16	Middlemore Hospital	Institute of Environmental Science and Research (ESR)	Xiaoyun Ren et al
EPI_ISL_632262	2020-08-17	Communicable Disease Laboratory, Public Health Directorate	Communicable Disease Laboratory, Public Health Directorate	AlWasti et al
EPI_ISL_614387	2020-08-17	Molecular diagnostic unit for viral haemorrhagic fevers and emerging viruses, Bouaké CHU Laboratory	Project group Epidemology of Highly Pathogenic Microorganisms, Robert Koch-Institute	Chantal Akoua-Koffi et al
EPI_ISL_710555	2020-08-17	University Hospital Dubrava	Ruđer Boškovic Institute; Forensic Science Centre Ivan Vučetić; University of Zagreb Faculty of Science	Robert Belužić et al
EPI_ISL_540252	2020-08-17	Lighthouse Lab in Glasgow	Wellcome Sanger Institute for the COVID-19 Genomics UK (COG-UK) consortium	Harper VanSteenhouse et al
EPI_ISL_540303	2020-08-17	Lighthouse Lab in Glasgow	Wellcome Sanger Institute for the COVID-19 Genomics UK (COG-UK) consortium	Harper VanSteenhouse et al
EPI_ISL_730042	2020-08-17	Nigeria Centre for Disease Control (NCDC)	African Centre of Excellence for Genomics of Infectious Diseases (ACEGID), Redeemer's University, Ede, Osun State, Nigeria	Oluniyi P.E. et al et al
EPI_ISL_729981	2020-08-17	Nigeria Centre for Disease Control (NCDC)	African Centre of Excellence for Genomics of Infectious Diseases (ACEGID), Redeemer's University, Ede, Osun State, Nigeria	Oluniyi P.E. et al et al
EPI_ISL_596527	2020-08-17	Palestinian Ministry of Health	Molecular Genetics Lab	Nouar Qutob et al
EPI_ISL_596528	2020-08-17	Palestinian Ministry of Health	Molecular Genetics Lab	Nouar Qutob et al
EPI_ISL_596529	2020-08-17	Palestinian Ministry of Health	Molecular Genetics Lab	Nouar Qutob et al
EPI_ISL_729914	2020-08-17	Instituto de Medicina Tropical, Universidad Nacional Toribio Rodríguez de Mendoza de Amazonas	Laboratorio de Genómica Microbiana, Universidad Peruana Cayetano Heredia	Pablo Tsukayama et al
EPI_ISL_568424	2020-08-17	Lighthouse Lab in Glasgow	Wellcome Sanger Institute for the COVID-19 Genomics UK (COG-UK) consortium	Harper VanSteenhouse et al
EPI_ISL_526969	2020-08-18	Instituto Nacional de Salud, Bogotá, Colombia	Instituto Nacional de Salud, Bogotá, Colombia	Katherine Laiton-Donato et al
EPI_ISL_526971	2020-08-18	Instituto Nacional de Salud, Bogotá, Colombia	Instituto Nacional de Salud, Bogotá, Colombia	Katherine Laiton-Donato et al
EPI_ISL_561283	2020-08-18	MRCG at LSHTM Genomics lab	MRCG at LSHTM Genomics lab	Abdul Karim sesay et al
EPI_ISL_578242	2020-08-18	National Virus Reference Laboratory	National Virus Reference Laboratory	Michael Carr et al
EPI_ISL_718281	2020-08-18	Institute for Medical Research, Infectious Disease Research Centre, National Institutes of Health, Ministry of Health Malaysia	Institute for Medical Research, Infectious Disease Research Centre, National Institutes of Health, Ministry of Health Malaysia	Suppiah J et al
EPI_ISL_569836	2020-08-18	Omsk Research Institute of Natural Focal Infections	WHO National Influenza Centre Russian Federation	Artem Fadeev et al
EPI_ISL_654280	2020-08-18	Hospital General Universitario Gregorio Marañón	SeqCOVID-SPAIN consortium/IBV(CSIC)	Darío García de Viedma et al
EPI_ISL_691716	2020-08-18	Hospital Clínico San Carlos	Instituto de Salud Carlos III	Iglesias-Caballero et al
EPI_ISL_539784	2020-08-19	Universidad Regional Amazonica IKIAM	Institute of Microbiology, Universidad San Francisco de Quito	Fabian Aguilar et al
EPI_ISL_697788	2020-08-19	Institute of Microbiology, Universidad San Francisco de Quito	Institute of Microbiology, Universidad San Francisco de Quito	Belén Prado-Vivar et al
EPI_ISL_729589	2020-08-19	A. Krumbholz, Labor Dr. Krause und Kollegen MVZ GmbH, Kiel	Charité Universitätsmedizin Berlin, Institut für Virologie	Victor M Corman et al
EPI_ISL_539737	2020-08-19	CSIR-Centre for Cellular and Molecular Biology	CSIR-Centre for Cellular and Molecular Biology	Lamuk Zaveri et al
EPI_ISL_576117	2020-08-19	Laboratory, The Bio Arte Limited	Laboratory, The Bio Arte Limited	Biazzo et al
EPI_ISL_596537	2020-08-19	Palestinian Ministry of Health	Molecular Genetics Lab	Nouar Qutob et al
EPI_ISL_596538	2020-08-19	Palestinian Ministry of Health	Molecular Genetics Lab	Nouar Qutob et al
EPI_ISL_717698	2020-08-19	Trinidad Public Health Laboratory	Carrington Lab, Department of PreClinical Sciences, Faculty of Medical Sciences, The University of the West Indies	Nikita S. D. Sahadeo et al
EPI_ISL_667249	2020-08-19	OHSU Lab Services Molecular Microbiology Lab	Oregon SARS-CoV-2 Genome Sequencing Center	Brendan L. O'Connell et al

EPI_ISL_700337	2020-08-20	Child Health Research Foundation	Child Health Research Foundation	Senjuti Saha et al
EPI_ISL_653762	2020-08-20	Instituto Nacional de Salud, Bogotá, Colombia	Instituto Nacional de Salud, Bogotá, Colombia	Katherine Laiton-Donato et al
EPI_ISL_527813	2020-08-20	Institute of Microbiology, Universidad San Francisco de Quito	Institute of Microbiology, Universidad San Francisco de Quito	Belén Prado-Vivar et al
EPI_ISL_527815	2020-08-20	Institute of Microbiology, Universidad San Francisco de Quito	Institute of Microbiology, Universidad San Francisco de Quito	Belén Prado-Vivar et al
EPI_ISL_527816	2020-08-20	Institute of Microbiology, Universidad San Francisco de Quito	Institute of Microbiology, Universidad San Francisco de Quito	Belén Prado-Vivar et al
EPI_ISL_538505	2020-08-20	National Institute of Health Research and Development	National Institute of Health Research and Development	Pawestri et al
EPI_ISL_538509	2020-08-20	National Institute of Health Research and Development	National Institute of Health Research and Development	Pawestri et al
EPI_ISL_649191	2020-08-20	Istituto Zooprofilattico Sperimentale della Puglia e della Basilicata	Istituto Zooprofilattico Sperimentale della Puglia e della Basilicata	Parisi A. et al
EPI_ISL_644997	2020-08-20	Department of Infectious Diseases, Keio University School of Medicine, Tokyo, Japan	Center for Medical Genetics, Keio University School of Medicine, Tokyo, Japan	Kenjiro Kosaki et al
EPI_ISL_576121	2020-08-20	Laboratory, The Bio Arte Limited	Laboratory, The Bio Arte Limited	Biazzo et al
EPI_ISL_729910	2020-08-20	Instituto de Medicina Tropical, Universidad Nacional Toribio Rodríguez de Mendoza de Amazonas	Laboratorio de Genómica Microbiana, Universidad Peruana Cayetano Heredia	Pablo Tsukayama et al
EPI_ISL_678486	2020-08-20	Veterinary Specialized Institute "Sabac", Serbia	Veterinary Specialized Institute "Kraljevo", Serbia	Vidanovic et al
EPI_ISL_527373	2020-08-20	National Public Health Laboratory, National Centre for Infectious Diseases	National Public Health Laboratory, National Centre for Infectious Diseases	Mak TM et al
EPI_ISL_527374	2020-08-20	National Public Health Laboratory, National Centre for Infectious Diseases	National Public Health Laboratory, National Centre for Infectious Diseases	Mak TM et al
EPI_ISL_654945	2020-08-20	Klinisk mikrobiologi	The Public Health Agency of Sweden	Anna-Malin Linde et al
EPI_ISL_535622	2020-08-20	Viollier AG	Department of Biosystems Science and Engineering, ETH Zürich	Christian Beisel et al
EPI_ISL_614390	2020-08-21	Molecular diagnostic unit for viral haemorrhagic fevers and emerging viruses, Bouaké CHU Laboratory	Project group Epidemology of Highly Pathogenic Microorganisms, Robert Koch-Institute	Chantal Akoua-Koffi et al
EPI_ISL_700167	2020-08-21	Hematopathology Laboratory, ACTREC, TMC	Hematopathology Laboratory, ACTREC, TMC	Hematopathology Laboratory et al
EPI_ISL_693473	2020-08-21	Central Public Health Laboratory	National Public Health Laboratory, National Centre for Infectious Diseases	Tze Minn Mak et al
EPI_ISL_562239	2020-08-22	Victorian Infectious Diseases Reference Laboratory (VIDRL)	VIDRL and MDU-PHL	Caly et al
EPI_ISL_528386	2020-08-22	Viral vaccines, VSVRI- Veterinary serum and vaccine research institute	Viral vaccines, VSVRI- Veterinary serum and vaccine research institute	Saleh et al
EPI_ISL_527002	2020-08-22	Biological prevention, army	Biological prevention, army	Seadawy et al
EPI_ISL_527006	2020-08-22	Biological prevention, army	Biological prevention, army	Seadawy et al
EPI_ISL_578243	2020-08-22	National Virus Reference Laboratory	National Virus Reference Laboratory	Michael Carr et al
EPI_ISL_637013	2020-08-22	Department of Infectious Diseases and Immunology, National Hospital Organization Nagoya Medical Center	Clinical Research Center, National Hospital Organization Nagoya Medical Center	Yoshihiro Nakata et al
EPI_ISL_534222	2020-08-22	Centrālā laboratorija	Latvian Biomedical Research and Study Centre	Ivars Silamiķelis et al
EPI_ISL_693632	2020-08-22	Hospital Vila Franca de Xira	Instituto Nacional de Saude (INSA)	Borges et al et al
EPI_ISL_700330	2020-08-23	Child Health Research Foundation	Child Health Research Foundation	Senjuti Saha et al
EPI_ISL_614391	2020-08-23	Molecular diagnostic unit for viral haemorrhagic fevers and emerging viruses, Bouaké CHU Laboratory	Project group Epidemology of Highly Pathogenic Microorganisms, Robert Koch-Institute	Chantal Akoua-Koffi et al
EPI_ISL_565504	2020-08-24	Microbiological Diagnostic Unit - Public Health Laboratory (MDU-PHL)	MDU-PHL	Seemann et al
EPI_ISL_660505	2020-08-24	Laboratoire de Microbiologie CHU Souro Sanou	Centre Muraz	Abdoul-Salam Ouedraogo et al
EPI_ISL_660500	2020-08-24	Laboratoire de Microbiologie CHU Souro Sanou	Centre Muraz	Abdoul-Salam Ouedraogo et al
EPI_ISL_591101	2020-08-24	Toronto Invasive Bacterial Diseases Network	McMaster University	Allison McGeer et al
EPI_ISL_591194	2020-08-24	Toronto Invasive Bacterial Diseases Network	McMaster University	Allison McGeer et al
EPI_ISL_591531	2020-08-24	Laboratorio de Infectologia y virologia molecular	Center for Mathematical Modeling and Center for Genome Regulation. Santiago, Chile	Valiente F et al

EPI_ISL_591532	2020-08-24	Laboratorio de Infectologia y virologia molecular	Center for Mathematical Modeling and Center for Genome Regulation. Santiago, Chile	Valiente F et al
EPI_ISL_710549	2020-08-24	University Hospital Dubrava	Ruđer Boškovic Institute; Forensic Science Centre Ivan Vučetić; University of Zagreb Faculty of Science	Robert Belužić et al
EPI_ISL_577634	2020-08-24	The National Institute of Public Health	State Veterinary Institute Prague	Nagy et al
EPI_ISL_577635	2020-08-24	The National Institute of Public Health	State Veterinary Institute Prague	Nagy et al
EPI_ISL_616697	2020-08-24	Department of Virus and Microbiological Special Diagnostics, Statens Serum Institut, Denmark	Albertsen lab, Department of Chemistry and Bioscience, Aalborg University, Denmark	Danish Covid-19 Genome Consortia et al
EPI_ISL_616735	2020-08-24	Department of Virus and Microbiological Special Diagnostics, Statens Serum Institut, Denmark	Albertsen lab, Department of Chemistry and Bioscience, Aalborg University, Denmark	Danish Covid-19 Genome Consortia et al
EPI_ISL_590910	2020-08-24	Oslo University Hospital, Department of Medical Microbiology	Norwegian Institute of Public Health, Department of Virology	Kathrine Stene-Johansen et al
EPI_ISL_693630	2020-08-24	Hospital Vila Franca de Xira	Instituto Nacional de Saude (INSA)	Borges et al et al
EPI_ISL_693631	2020-08-24	Hospital Vila Franca de Xira	Instituto Nacional de Saude (INSA)	Borges et al et al
EPI_ISL_693634	2020-08-24	Hospital Vila Franca de Xira	Instituto Nacional de Saude (INSA)	Borges et al et al
EPI_ISL_653931	2020-08-24	Molecular diagnostic laboratory of Federal Budget Institution of Science "Central Research Institute of Epidemiology" of The Federal Service on Customers' Rights Protection and Human Well-being Surveillance	Group of Genomics and Postgenomic Technologies of Central Research Institute of Epidemilology	Samoilov AE et al
EPI_ISL_653922	2020-08-24	Molecular diagnostic laboratory of Federal Budget Institution of Science "Central Research Institute of Epidemiology" of The Federal Service on Customers' Rights Protection and Human Well-being Surveillance	Group of Genomics and Postgenomic Technologies of Central Research Institute of Epidemilology	Samoilov AE et al
EPI_ISL_653930	2020-08-24	Molecular diagnostic laboratory of Federal Budget Institution of Science "Central Research Institute of Epidemiology" of The Federal Service on Customers' Rights Protection and Human Well-being Surveillance	Group of Genomics and Postgenomic Technologies of Central Research Institute of Epidemilology	Samoilov AE et al
EPI_ISL_636518	2020-08-25	Dutch COVID-19 response team	National Institute for Public Health and the Environment (RIVM)	Adam Meijer et al
EPI_ISL_632263	2020-08-25	Communicable Disease Laboratory, Public Health Directorate	Communicable Disease Laboratory, Public Health Directorate	AlWasti et al
EPI_ISL_591533	2020-08-25	Laboratorio de Infectologia y virologia molecular	Center for Mathematical Modeling and Center for Genome Regulation. Santiago, Chile	Valiente F et al
EPI_ISL_577636	2020-08-25	The National Institute of Public Health	State Veterinary Institute Prague	Nagy et al
EPI_ISL_708194	2020-08-25	Pamukkale University Hospital	Pamukkale University Department of Medical Genetics	Onur TOKGUN et al. et al
EPI_ISL_582642	2020-08-25	Sheikh Khalifa Medical City	Molecular/Surveillance lab Sheikh Khalifa Medical City	Amirtharaj Francis et al
EPI_ISL_636973	2020-08-26	Public Health Lab	Public Health Lab	Alwasti et al
EPI_ISL_591534	2020-08-26	Laboratorio de Infectologia y virologia molecular	Center for Mathematical Modeling and Center for Genome Regulation. Santiago, Chile	Valiente F et al
EPI_ISL_653758	2020-08-26	Instituto Nacional de Salud, Bogotá, Colombia	Instituto Nacional de Salud, Bogotá, Colombia	Katherine Laiton-Donato et al
EPI_ISL_590885	2020-08-26	Vestfold Hospital, Toensberg Department of Microbiology	Norwegian Institute of Public Health, Department of Virology	Kathrine Stene-Johansen et al
EPI_ISL_729919	2020-08-26	Instituto de Medicina Tropical, Universidad Nacional Toribio Rodríguez de Mendoza de Amazonas	Laboratorio de Genómica Microbiana, Universidad Peruana Cayetano Heredia	Pablo Tsukayama et al
EPI_ISL_717695	2020-08-26	Trinidad Public Health Laboratory	Carrington Lab, Department of PreClinical Sciences, Faculty of Medical Sciences, The University of the West Indies	Nikita S. D. Sahadeo et al
EPI_ISL_708186	2020-08-26	Pamukkale University Hospital	Pamukkale University Department of Medical Genetics	Onur TOKGUN et al. et al
EPI_ISL_708195	2020-08-26	Pamukkale University Hospital	Pamukkale University Department of Medical Genetics	Onur TOKGUN et al. et al
EPI_ISL_591110	2020-08-27	Toronto Invasive Bacterial Diseases Network	McMaster University	Allison McGeer et al
EPI_ISL_591111	2020-08-27	Toronto Invasive Bacterial Diseases Network	McMaster University	Allison McGeer et al
EPI_ISL_677728	2020-08-27	University of Szeged, Institute of Clinical Microbiology	National Laboratory of Virology, Szentágothai Research Centre	Endre Gábor Tóth et al
EPI_ISL_548049	2020-08-27	LabPLUS	Institute of Environmental Science and Research (ESR)	Xiaoyun Ren et al
EPI_ISL_590886	2020-08-27	Vestfold Hospital, Toensberg Department of Microbiology	Norwegian Institute of Public Health, Department of Virology	Kathrine Stene-Johansen et al

EPI_ISL_568519	2020-08-27	Laboratorio de Referencia Nacional de Virus Respiratorios, Instituto Nacional de Salud Peru	Laboratorio de Genómica Microbiana, Universidad Peruana Cayetano Heredia	Pablo Tsukayama et al
EPI_ISL_568539	2020-08-27	Laboratorio de Referencia Nacional de Virus Respiratorios, Instituto Nacional de Salud Peru	Laboratorio de Genómica Microbiana, Universidad Peruana Cayetano Heredia	Pablo Tsukayama et al
EPI_ISL_568543	2020-08-27	Laboratorio de Referencia Nacional de Virus Respiratorios, Instituto Nacional de Salud Peru	Laboratorio de Genómica Microbiana, Universidad Peruana Cayetano Heredia	Pablo Tsukayama et al
EPI_ISL_693637	2020-08-27	Hospital Vila Franca de Xira	Instituto Nacional de Saude (INSA)	Borges et al et al
EPI_ISL_591115	2020-08-28	Toronto Invasive Bacterial Diseases Network	McMaster University	Allison McGeer et al
EPI_ISL_644475	2020-08-28	MEPHI, Aix Marseille University	MEPHI, Aix Marseille University	Anthony LEVASSEUR et al
EPI_ISL_539578	2020-08-28	ZOTZ KLIMAS MVZ Düsseldorf-Centrum GbR ÜBAG für Labormedizin, Genetik, Zytologie, Pathologie	Center of Medical Microbiology, Virology, and Hospital Hygiene, University of Duesseldorf	Maximilian Damagnez et al
EPI_ISL_539581	2020-08-28	ZOTZ KLIMAS MVZ Düsseldorf-Centrum GbR ÜBAG für Labormedizin, Genetik, Zytologie, Pathologie	Center of Medical Microbiology, Virology, and Hospital Hygiene, University of Duesseldorf	Maximilian Damagnez et al
EPI_ISL_539582	2020-08-28	ZOTZ KLIMAS MVZ Düsseldorf-Centrum GbR ÜBAG für Labormedizin, Genetik, Zytologie, Pathologie	Center of Medical Microbiology, Virology, and Hospital Hygiene, University of Duesseldorf	Maximilian Damagnez et al
EPI_ISL_677731	2020-08-28	University of Szeged, Institute of Clinical Microbiology	National Laboratory of Virology, Szentágothai Research Centre	Endre Gábor Tóth et al
EPI_ISL_677732	2020-08-28	University of Szeged, Institute of Clinical Microbiology	National Laboratory of Virology, Szentágothai Research Centre	Endre Gábor Tóth et al
EPI_ISL_677734	2020-08-28	University of Szeged, Institute of Clinical Microbiology	National Laboratory of Virology, Szentágothai Research Centre	Endre Gábor Tóth et al
EPI_ISL_722852	2020-08-28	I.R.C.C.S. "S. De Bellis" - Ente Ospedaliero	Istituto Zooprofilattico Sperimentale della Puglia e della Basilicata	Parisi A. et al
EPI_ISL_722861	2020-08-28	I.R.C.C.S. "S. De Bellis" - Ente Ospedaliero	Istituto Zooprofilattico Sperimentale della Puglia e della Basilicata	Parisi A. et al
EPI_ISL_722853	2020-08-28	I.R.C.C.S. "S. De Bellis" - Ente Ospedaliero	Istituto Zooprofilattico Sperimentale della Puglia e della Basilicata	Parisi A. et al
EPI_ISL_722900	2020-08-28	I.R.C.C.S. "S. De Bellis" - Ente Ospedaliero	Istituto Zooprofilattico Sperimentale della Puglia e della Basilicata	Parisi A. et al
EPI_ISL_568524	2020-08-28	Laboratorio de Referencia Nacional de Virus Respiratorios, Instituto Nacional de Salud Peru	Laboratorio de Genómica Microbiana, Universidad Peruana Cayetano Heredia	Pablo Tsukayama et al
EPI_ISL_568533	2020-08-28	Laboratorio de Referencia Nacional de Virus Respiratorios, Instituto Nacional de Salud Peru	Laboratorio de Genómica Microbiana, Universidad Peruana Cayetano Heredia	Pablo Tsukayama et al
EPI_ISL_568541	2020-08-28	Laboratorio de Referencia Nacional de Virus Respiratorios, Instituto Nacional de Salud Peru	Laboratorio de Genómica Microbiana, Universidad Peruana Cayetano Heredia	Pablo Tsukayama et al
EPI_ISL_568542	2020-08-28	Laboratorio de Referencia Nacional de Virus Respiratorios, Instituto Nacional de Salud Peru	Laboratorio de Genómica Microbiana, Universidad Peruana Cayetano Heredia	Pablo Tsukayama et al
EPI_ISL_717694	2020-08-28	Trinidad Public Health Laboratory	Carrington Lab, Department of PreClinical Sciences, Faculty of Medical Sciences, The University of the West Indies	Nikita S. D. Sahadeo et al
EPI_ISL_708192	2020-08-28	Pamukkale University Hospital	Pamukkale University Department of Medical Genetics	Onur TOKGUN et al. et al
EPI_ISL_582645	2020-08-28	Sheikh Khalifa Medical City	Molecular/Surveillance lab Sheikh Khalifa Medical City	Amirtharaj Francis et al
EPI_ISL_548064	2020-08-29	Waikato Hospital	Institute of Environmental Science and Research (ESR)	Xiaoyun Ren et al
EPI_ISL_693477	2020-08-29	Central Public Health Laboratory	National Public Health Laboratory, National Centre for Infectious Diseases	Tze Minn Mak et al
EPI_ISL_536420	2020-08-29	National Public Health Laboratory, National Centre for Infectious Diseases	National Public Health Laboratory, National Centre for Infectious Diseases	Mak TM et al
EPI_ISL_632312	2020-08-29	NU-sjukvården	Clinical microbiology, Sahlgrenska University Hospital	Johan Ringlander et al
EPI_ISL_717699	2020-08-29	Trinidad Public Health Laboratory	Carrington Lab, Department of PreClinical Sciences, Faculty of Medical Sciences, The University of the West Indies	Nikita S. D. Sahadeo et al
EPI_ISL_582647	2020-08-29	Sheikh Khalifa Medical City	Molecular/Surveillance lab Sheikh Khalifa Medical City	Amirtharaj Francis et al
EPI_ISL_582656	2020-08-29	Sheikh Khalifa Medical City	Molecular/Surveillance lab Sheikh Khalifa Medical City	Amirtharaj Francis et al
EPI_ISL_582649	2020-08-29	Sheikh Khalifa Medical City	Molecular/Surveillance lab Sheikh Khalifa Medical City	Amirtharaj Francis et al
EPI_ISL_710556	2020-08-30	University Hospital Dubrava	Ruđer Boškovic Institute; Forensic Science Centre Ivan Vučetić; University of Zagreb Faculty of Science	Robert Belužić et al
EPI_ISL_536421	2020-08-30	National Public Health Laboratory, National Centre for Infectious Diseases	National Public Health Laboratory, National Centre for Infectious Diseases	Mak TM et al

EPI_ISL_536679	2020-08-30	University of Wisconsin-Madison AIDS Vaccine Research Laboratories	University of Wisconsin-Madison AIDS Vaccine Research Laboratories	Gage Moreno et al
EPI_ISL_710548	2020-08-31	University Hospital Dubrava	Ruđer Boškovic Institute; Forensic Science Centre Ivan Vučetić; University of Zagreb Faculty of Science	Robert Belužić et al
EPI_ISL_616211	2020-08-31	Department of Virus and Microbiological Special Diagnostics, Statens Serum Institut, Denmark	Albertsen lab, Department of Chemistry and Bioscience, Aalborg University, Denmark	Danish Covid-19 Genome Consortia et al
EPI_ISL_616290	2020-08-31	Department of Virus and Microbiological Special Diagnostics, Statens Serum Institut, Denmark	Albertsen lab, Department of Chemistry and Bioscience, Aalborg University, Denmark	Danish Covid-19 Genome Consortia et al
EPI_ISL_677738	2020-08-31	University of Szeged, Institute of Clinical Microbiology	National Laboratory of Virology, Szentágothai Research Centre	Endre Gábor Tóth et al
EPI_ISL_610162	2020-08-31	RSUD Dr. Tjitrowardojo	Genetics Working Group (Pokja Genetik) Faculty of Medicine, Public Health and Nursing Universitas Gadjah Mada (FK-KMK UGM); Disease Investigation Center Wates Ministry of Agriculture Indonesia; Department of Microbiology FK-KMK UGM; Laboratorium Diagnostik Yayasan Tahija World Mosquito Program (WMP) Yogyakarta Center for Tropical Medicine FK-KMK UGM; Integrated Research Center FK-KMK UGM; Department of Computer Science and Electronics FMIPA UGM; Balai Besar Teknik Kesehatan Lingkungan dan Pengendalian Penyakit (BBTKLPP) Yogyakarta	Gunadi et al
EPI_ISL_639660	2020-08-31	E. Gulbja Laboratorija	Latvian Biomedical Research and Study Centre	Ivars Silamiķelis et al
EPI_ISL_583895	2020-08-31	Singapore General Hospital	Department of Microbiology	Nurdyana Abdul Rahman et al
EPI_ISL_602645	2020-08-31	NHLS-IALCH	KRISP, KZN Research Innovation and Sequencing Platform	Giandhari J et al
EPI_ISL_602669	2020-08-31	NHLS-IALCH	KRISP, KZN Research Innovation and Sequencing Platform	Giandhari J et al
EPI_ISL_536663	2020-08-31	University of Wisconsin-Madison AIDS Vaccine Research Laboratories	University of Wisconsin-Madison AIDS Vaccine Research Laboratories	Gage Moreno et al
EPI_ISL_700342	2020-09-01	Child Health Research Foundation	Child Health Research Foundation	Senjuti Saha et al
EPI_ISL_609821	2020-09-01	Unity Health Toronto	Ontario Institute for Cancer Research	Ramzi Fattouh et al
EPI_ISL_569218	2020-09-01	MEPHI, Aix Marseille University	MEPHI, Aix Marseille University	Anthony LEVASSEUR et al
EPI_ISL_677743	2020-09-01	University of Szeged, Institute of Clinical Microbiology	National Laboratory of Virology, Szentágothai Research Centre	Endre Gábor Tóth et al
EPI_ISL_677745	2020-09-01	University of Szeged, Institute of Clinical Microbiology	National Laboratory of Virology, Szentágothai Research Centre	Endre Gábor Tóth et al
EPI_ISL_576383	2020-09-01	RSUD Budi Rahayu Kota Magelang	Genetics Working Group (Pokja Genetik) Faculty of Medicine, Public Health and Nursing Universitas Gadjah Mada (FK-KMK UGM); Disease Investigation Center Wates Ministry of Agriculture Indonesia; Department of Microbiology FK-KMK UGM; Laboratorium Diagnostik Yayasan Tahija World Mosquito Program (WMP) Yogyakarta Center for Tropical Medicine FK-KMK UGM; Integrated Research Center FK-KMK UGM; Department of Computer Science and Electronics FMIPA UGM; Balai Besar Teknik Kesehatan Lingkungan dan Pengendalian Penyakit (BBTKLPP) Yogyakarta	Gunadi et al
EPI_ISL_610161	2020-09-01	RSUD Dr. Tjitrowardojo	Genetics Working Group (Pokja Genetik) Faculty of Medicine, Public Health and Nursing Universitas Gadjah Mada (FK-KMK UGM); Disease Investigation Center Wates Ministry of Agriculture Indonesia; Department of Microbiology FK-KMK UGM; Laboratorium Diagnostik Yayasan Tahija World Mosquito Program (WMP) Yogyakarta Center for Tropical Medicine FK-KMK UGM; Integrated Research Center FK-KMK UGM; Department of Computer Science and Electronics FMIPA UGM; Balai Besar Teknik Kesehatan Lingkungan dan Pengendalian Penyakit (BBTKLPP) Yogyakarta	Gunadi et al
EPI_ISL_575332	2020-09-01	Israel Central Virology laboratory	Israel Central Virology laboratory	Neta Zuckerman et al
EPI_ISL_575333	2020-09-01	Israel Central Virology laboratory	Israel Central Virology laboratory	Neta Zuckerman et al

EPI_ISL_575334	2020-09-01	Israel Central Virology laboratory	Israel Central Virology laboratory	Neta Zuckerman et al
EPI_ISL_636882	2020-09-01	Lithuanian University of Health Sciences Hospital, Department of Laboratory Medicine	Lithuanian University of Health Sciences, Molecular cardiology lab.	Lukas Zemaitis et al
EPI_ISL_718302	2020-09-01	Institute for Medical Research, Infectious Disease Research Centre, National Institutes of Health, Ministry of Health Malaysia	Institute for Medical Research, Infectious Disease Research Centre, National Institutes of Health, Ministry of Health Malaysia	Suppiah J et al
EPI_ISL_548067	2020-09-01	North Shore Hospital	Institute of Environmental Science and Research (ESR)	Xiaoyun Ren et al
EPI_ISL_729979	2020-09-01	Nigeria Centre for Disease Control (NCDC)	African Centre of Excellence for Genomics of Infectious Diseases (ACEGID), Redeemer's University, Ede, Osun State, Nigeria	Oluniyi P.E. et al et al
EPI_ISL_693478	2020-09-01	Central Public Health Laboratory	National Public Health Laboratory, National Centre for Infectious Diseases	Tze Minn Mak et al
EPI_ISL_602702	2020-09-01	NHLS-IALCH	KRISP, KZN Research Innovation and Sequencing Platform	Giandhari J et al
EPI_ISL_582914	2020-09-01	County of Santa Clara Public Health Department	Chan-Zuckerberg Biohub	CZB Cliahub Consortium et al
EPI_ISL_660512	2020-09-02	Laboratoire de Microbiologie CHU Souro Sanou	Centre Muraz	Abdoul-Salam Ouedraogo et al
EPI_ISL_660508	2020-09-02	Laboratoire de Microbiologie CHU Souro Sanou	Centre Muraz	Abdoul-Salam Ouedraogo et al
EPI_ISL_653754	2020-09-02	Instituto Nacional de Salud, Bogotá, Colombia	Instituto Nacional de Salud, Bogotá, Colombia	Katherine Laiton-Donato et al
EPI_ISL_697789	2020-09-02	Institute of Microbiology, Universidad San Francisco de Quito	Institute of Microbiology, Universidad San Francisco de Quito	Belén Prado-Vivar et al
EPI_ISL_697792	2020-09-02	Institute of Microbiology, Universidad San Francisco de Quito	Institute of Microbiology, Universidad San Francisco de Quito	Belén Prado-Vivar et al
EPI_ISL_671439	2020-09-02	University of Debrecen, Department of Medical Microbiology	National Laboratory of Virology, Szentágothai Research Centre	Endre Gábor Tóth et al
EPI_ISL_671441	2020-09-02	University of Debrecen, Department of Medical Microbiology	National Laboratory of Virology, Szentágothai Research Centre	Endre Gábor Tóth et al
EPI_ISL_677760	2020-09-02	University of Szeged, Institute of Clinical Microbiology	National Laboratory of Virology, Szentágothai Research Centre	Endre Gábor Tóth et al
EPI_ISL_590901	2020-09-02	Furst Medical Laboratory	Norwegian Institute of Public Health, Department of Virology	Kathrine Stene-Johansen et al
EPI_ISL_693535	2020-09-02	Hospital Vila Franca de Xira	Instituto Nacional de Saude (INSA)	Borges et al et al
EPI_ISL_717697	2020-09-02	Trinidad Public Health Laboratory	Carrington Lab, Department of PreClinical Sciences, Faculty of Medical Sciences, The University of the West Indies	Nikita S. D. Sahadeo et al
EPI_ISL_562293	2020-09-03	Microbiological Diagnostic Unit - Public Health Laboratory (MDU-PHL)	MDU-PHL	Seemann et al
EPI_ISL_717806	2020-09-03	Laboratorio de Virologia Molecular / UFRJ	Bioinformatics Laboratory / LNCC	Carolina M Voloch et al
EPI_ISL_717814	2020-09-03	Laboratorio de Virologia Molecular / UFRJ	Bioinformatics Laboratory / LNCC	Carolina M Voloch et al
EPI_ISL_717815	2020-09-03	Laboratorio de Virologia Molecular / UFRJ	Bioinformatics Laboratory / LNCC	Carolina M Voloch et al
EPI_ISL_717816	2020-09-03	Laboratorio de Virologia Molecular / UFRJ	Bioinformatics Laboratory / LNCC	Carolina M Voloch et al
EPI_ISL_717869	2020-09-03	Laboratorio de Virologia Molecular / UFRJ	Bioinformatics Laboratory / LNCC	Carolina M Voloch et al
EPI_ISL_625675	2020-09-03	Laboratory of Molecular Medicine, University of Magallanes	Centro Asistencial Docente y de Investigacion, Universidad de Magallanes	Jorge Gonzalez et al
EPI_ISL_710557	2020-09-03	University Hospital Dubrava	Ruđer Boškovic Institute; Forensic Science Centre Ivan Vučetić; University of Zagreb Faculty of Science	Robert Belužić et al
EPI_ISL_636883	2020-09-03	Lithuanian University of Health Sciences Hospital, Department of Laboratory Medicine	Lithuanian University of Health Sciences, Molecular cardiology lab.	Lukas Zemaitis et al
EPI_ISL_636885	2020-09-03	Lithuanian University of Health Sciences Hospital, Department of Laboratory Medicine	Lithuanian University of Health Sciences, Molecular cardiology lab.	Lukas Zemaitis et al
EPI_ISL_548077	2020-09-03	LabPLUS	Institute of Environmental Science and Research (ESR)	Xiaoyun Ren et al
EPI_ISL_729980	2020-09-03	Nigeria Centre for Disease Control (NCDC)	African Centre of Excellence for Genomics of Infectious Diseases (ACEGID), Redeemer's University, Ede, Osun State, Nigeria	Oluniyi P.E. et al et al
EPI_ISL_590882	2020-09-03	Unilabs Laboratory Medicine	Norwegian Institute of Public Health, Department of Virology	Kathrine Stene-Johansen et al
EPI_ISL_536430	2020-09-03	National Public Health Laboratory, National Centre for Infectious Diseases	National Public Health Laboratory, National Centre for Infectious Diseases	Mak TM et al
EPI_ISL_536444	2020-09-03	National Public Health Laboratory, National Centre for Infectious Diseases	National Public Health Laboratory, National Centre for Infectious Diseases	Mak TM et al

EPI_ISL_536451	2020-09-03	National Public Health Laboratory, National Centre for Infectious Diseases	National Public Health Laboratory, National Centre for Infectious Diseases	Mak TM et al
EPI_ISL_541447	2020-09-03	Viollier AG	Department of Biosystems Science and Engineering, ETH Zürich	Christian Beisel et al
EPI_ISL_667362	2020-09-03	OHSU Lab Services Molecular Microbiology Lab	Oregon SARS-CoV-2 Genome Sequencing Center	Brendan L. O'Connell et al
EPI_ISL_596766	2020-09-04	PathWest Laboratory Medicine WA	PathWest Laboratory Medicine WA Microbial Surveillance Unit	PathWest Laboratory Medicine WA Microbial Surveillance Unit et al
EPI_ISL_717817	2020-09-04	Laboratorio de Virologia Molecular / UFRJ	Bioinformatics Laboratory / LNCC	Carolina M Voloch et al
EPI_ISL_717870	2020-09-04	Laboratorio de Virologia Molecular / UFRJ	Bioinformatics Laboratory / LNCC	Carolina M Voloch et al
EPI_ISL_717871	2020-09-04	Laboratorio de Virologia Molecular / UFRJ	Bioinformatics Laboratory / LNCC	Carolina M Voloch et al
EPI_ISL_717872	2020-09-04	Laboratorio de Virologia Molecular / UFRJ	Bioinformatics Laboratory / LNCC	Carolina M Voloch et al
EPI_ISL_667769	2020-09-04	Microbiology, Infectious Diseases and Immunology, Centre de Recherche du Centre Hospitalier de l'Universite de Montreal	Microbiology, Infectious Diseases and Immunology, Centre de Recherche du Centre Hospitalier de l'Universite de Montreal	Benoit et al
EPI_ISL_667759	2020-09-04	Microbiology, Infectious Diseases and Immunology, Centre de Recherche du Centre Hospitalier de l'Universite de Montreal	Microbiology, Infectious Diseases and Immunology, Centre de Recherche du Centre Hospitalier de l'Universite de Montreal	Benoit et al
EPI_ISL_614393	2020-09-04	Molecular diagnostic unit for viral haemorrhagic fevers and emerging viruses, Bouaké CHU Laboratory	Project group Epidemology of Highly Pathogenic Microorganisms, Robert Koch-Institute	Chantal Akoua-Koffi et al
EPI_ISL_666635	2020-09-04	ZOTZ KLIMAS MVZ Düsseldorf-Centrum GbR ÜBAG für Labormedizin, Genetik, Zytologie, Pathologie	Center of Medical Microbiology, Virology, and Hospital Hygiene, University of Duesseldorf	Maximilian Damagnez et al
EPI_ISL_576118	2020-09-04	Laboratory, The Bio Arte Limited	Laboratory, The Bio Arte Limited	Biazzo et al
EPI_ISL_548068	2020-09-04	LabPLUS	Institute of Environmental Science and Research (ESR)	Xiaoyun Ren et al
EPI_ISL_639950	2020-09-04	HELIX LLC	WHO National Influenza Centre Russian Federation	Andrey Komissarov et al
EPI_ISL_602755	2020-09-04	NHLS-IALCH	KRISP, KZN Research Innovation and Sequencing Platform	Giandhari J et al
EPI_ISL_602758	2020-09-04	NHLS-IALCH	KRISP, KZN Research Innovation and Sequencing Platform	Giandhari J et al
EPI_ISL_576123	2020-09-05	Laboratory, The Bio Arte Limited	Laboratory, The Bio Arte Limited	Biazzo et al
EPI_ISL_548069	2020-09-05	LabPLUS	Institute of Environmental Science and Research (ESR)	Xiaoyun Ren et al
EPI_ISL_548073	2020-09-05	LabPLUS	Institute of Environmental Science and Research (ESR)	Xiaoyun Ren et al
EPI_ISL_582031	2020-09-05	Institute of Human Genetics, Polish Academy of Sciences	Institute of Human Genetics, Polish Academy of Sciences	Szymon Hryhorowicz et al
EPI_ISL_582032	2020-09-05	Institute of Human Genetics, Polish Academy of Sciences	Institute of Human Genetics, Polish Academy of Sciences	Szymon Hryhorowicz et al
EPI_ISL_596764	2020-09-06	PathWest Laboratory Medicine WA	PathWest Laboratory Medicine WA Microbial Surveillance Unit	PathWest Laboratory Medicine WA Microbial Surveillance Unit et al
EPI_ISL_700331	2020-09-06	Child Health Research Foundation	Child Health Research Foundation	Senjuti Saha et al
EPI_ISL_700343	2020-09-06	Child Health Research Foundation	Child Health Research Foundation	Senjuti Saha et al
EPI_ISL_707730	2020-09-06	Department of Clinical Microbiology	GIGA Medical Genomics	Keith Durkin et al
EPI_ISL_710550	2020-09-06	University Hospital Dubrava	Ruđer Boškovic Institute; Forensic Science Centre Ivan Vučetić; University of Zagreb Faculty of Science	Robert Beložić et al
EPI_ISL_710558	2020-09-06	University Hospital Dubrava	Ruđer Boškovic Institute; Forensic Science Centre Ivan Vučetić; University of Zagreb Faculty of Science	Robert Beložić et al
EPI_ISL_613557	2020-09-06	CHRU Pontchaillou - Laboratoire de Virologie 2, rue Henri Le Guilloux	National Reference Center for Viruses of Respiratory Infections, Institut Pasteur, Paris	Marion Barbet et al
EPI_ISL_636887	2020-09-06	Lithuanian University of Health Sciences Hospital, Department of Laboratory Medicine	Lithuanian University of Health Sciences, Molecular cardiology lab.	Lukas Zemaitis et al
EPI_ISL_536432	2020-09-06	National Public Health Laboratory, National Centre for Infectious Diseases	National Public Health Laboratory, National Centre for Infectious Diseases	Mak TM et al
EPI_ISL_654170	2020-09-06	Hospital General Universitario Gregorio Marañón	SeqCOVID-SPAIN consortium/IBV(CSIC)	Darío García de Viedma et al
EPI_ISL_660544	2020-09-06	Laboratory Medicine	Department of Laboratory Medicine, Lin-Kou Chang Gung Memorial Hospital, Taoyuan, Taiwan	Kuo-Chien Tsao et al
EPI_ISL_697790	2020-09-07	Institute of Microbiology, Universidad San Francisco de Quito	Institute of Microbiology, Universidad San Francisco de Quito	Belén Prado-Vivar et al
EPI_ISL_697793	2020-09-07	Institute of Microbiology, Universidad San Francisco de Quito	Institute of Microbiology, Universidad San Francisco de Quito	Belén Prado-Vivar et al

EPI_ISL_561302	2020-09-07	MRCG at LSHTM Genomics lab	MRCG at LSHTM Genomics lab	Abdul Karim sesay et al
EPI_ISL_578245	2020-09-07	National Virus Reference Laboratory	National Virus Reference Laboratory	Michael Carr et al
EPI_ISL_576120	2020-09-07	Laboratory, The Bio Arte Limited	Laboratory, The Bio Arte Limited	Biazzo et al
EPI_ISL_576124	2020-09-07	Laboratory, The Bio Arte Limited	Laboratory, The Bio Arte Limited	Biazzo et al
EPI_ISL_576125	2020-09-07	Laboratory, The Bio Arte Limited	Laboratory, The Bio Arte Limited	Biazzo et al
EPI_ISL_658901	2020-09-07	Instituto de Diagnostico y Referencia Epidemiologicos (INDRE)	Instituto de Diagnostico y Referencia Epidemiologicos (INDRE)	Ernesto Ramirez-Gonzalez et al
EPI_ISL_678253	2020-09-07	Institute for Lung Diseases in Children - Skopje	Research Center for Genetic Engineering and Biotechnology "Georgi D. Efremov" , Macedonian Academy of Sciences and Arts	RCGEB - MASA et al
EPI_ISL_678254	2020-09-07	General Hospital - Strumica	Research Center for Genetic Engineering and Biotechnology "Georgi D. Efremov" , Macedonian Academy of Sciences and Arts	RCGEB - MASA et al
EPI_ISL_678255	2020-09-07	General Hospital - Ohrid	Research Center for Genetic Engineering and Biotechnology "Georgi D. Efremov" , Macedonian Academy of Sciences and Arts	RCGEB - MASA et al
EPI_ISL_596301	2020-09-07	HELIX LCC	WHO National Influenza Centre Russian Federation	Andrey Komissarov et al
EPI_ISL_648198	2020-09-07	Skovde/Unilabs	The Public Health Agency of Sweden	Anna-Malin Linde et al
EPI_ISL_707745	2020-09-08	Department of Clinical Microbiology	GIGA Medical Genomics	Keith Durkin et al
EPI_ISL_707746	2020-09-08	Department of Clinical Microbiology	GIGA Medical Genomics	Keith Durkin et al
EPI_ISL_717873	2020-09-08	Laboratorio de Virologia Molecular / UFRJ	Bioinformatics Laboratory / LNCC	Carolina M Voloch et al
EPI_ISL_625676	2020-09-08	Laboratory of Molecular Medicine, University of Magallanes	Centro Asistencial Docente y de Investigacion, Universidad de Magallanes	Jorge Gonzalez et al
EPI_ISL_610217	2020-09-08	Department of Health Technology and Informatics, The Hong Kong Polytechnic University	Department of Health Technology and Informatics, The Hong Kong Polytechnic University	Siu et al
EPI_ISL_729904	2020-09-08	Instituto de Medicina Tropical, Universidad Nacional Toribio Rodríguez de Mendoza de Amazonas	Laboratorio de Genómica Microbiana, Universidad Peruana Cayetano Heredia	Pablo Tsukayama et al
EPI_ISL_729905	2020-09-08	Instituto de Medicina Tropical, Universidad Nacional Toribio Rodríguez de Mendoza de Amazonas	Laboratorio de Genómica Microbiana, Universidad Peruana Cayetano Heredia	Pablo Tsukayama et al
EPI_ISL_582049	2020-09-08	Servicio de Microbiología. Hospital Universitario Donostia. OSI Donostialdea. Área de Enfermedades Infecciosas, Grupo de Infección Respiratoria y Resistencia Antimicrobiana. Instituto de Investigación Sanitaria Biodonostia	SeqCOVID-SPAIN consortium/IBV(CSIC)	Gustavo Cilla et al
EPI_ISL_560500	2020-09-08	Viollier AG	Department of Biosystems Science and Engineering, ETH Zürich	Christian Beisel et al
EPI_ISL_614394	2020-09-09	Molecular diagnostic unit for viral haemorrhagic fevers and emerging viruses, Bouaké CHU Laboratory	Project group Epidemology of Highly Pathogenic Microorganisms, Robert Koch-Institute	Chantal Akoua-Koffi et al
EPI_ISL_614395	2020-09-09	Molecular diagnostic unit for viral haemorrhagic fevers and emerging viruses, Bouaké CHU Laboratory	Project group Epidemology of Highly Pathogenic Microorganisms, Robert Koch-Institute	Chantal Akoua-Koffi et al
EPI_ISL_573039	2020-09-09	Oxford Viromics, NDM, University of Oxford; Oxford University Hospitals; Basingstoke and North Hampshire Hospital	COVID-19 Genomics UK (COG-UK) Consortium	Tanya Golubchik et al
EPI_ISL_573042	2020-09-09	Oxford Viromics, NDM, University of Oxford; Oxford University Hospitals; Basingstoke and North Hampshire Hospital	COVID-19 Genomics UK (COG-UK) Consortium	Tanya Golubchik et al
EPI_ISL_632937	2020-09-09	RSUD Saptosari Gunung Kidul	Genetics Working Group (Pokja Genetik) Faculty of Medicine, Public Health and Nursing Universitas Gadjah Mada (FK-KMK UGM); Disease Investigation Center Wates Ministry of Agriculture Indonesia; Department of Microbiology FK-KMK UGM; Laboratorium Diagnostik Yayasan Tahija World Mosquito Program (WMP) Yogyakarta Center for Tropical Medicine FK-KMK UGM; Integrated Research Center FK-KMK UGM; Department of Computer Science and Electronics FMIPA UGM; Balai Besar Teknik Kesehatan Lingkungan dan Pengendalian Penyakit (BBTKLPP) Yogyakarta	Gunadi et al
EPI_ISL_722848	2020-09-09	Dutch COVID-19 response team	Erasmus Medical Center	Bas Oude Munnink et al
EPI_ISL_678258	2020-09-09	General Hospital - Prilep	Research Center for Genetic Engineering and Biotechnology "Georgi D. Efremov" , Macedonian Academy of Sciences and Arts	RCGEB - MASA et al

EPI_ISL_596308	2020-09-09	HELIX LCC	WHO National Influenza Centre Russian Federation	Andrey Komissarov et al
EPI_ISL_560460	2020-09-09	Viollier AG	Department of Biosystems Science and Engineering, ETH Zürich	Christian Beisel et al
EPI_ISL_717692	2020-09-09	Trinidad Public Health Laboratory	Carrington Lab, Department of PreClinical Sciences, Faculty of Medical Sciences, The University of the West Indies	Nikita S. D. Sahadeo et al
EPI_ISL_644865	2020-09-09	Virginia DCLS	Virginia DCLS	Virginia DCLS et al
EPI_ISL_681309	2020-09-10	Communicable Disease Laboratory, Public Health Directorate	Communicable Disease Laboratory, Public Health Directorate	Alwasti et al
EPI_ISL_609812	2020-09-10	Unity Health Toronto	Ontario Institute for Cancer Research	Ramzi Fattouh et al
EPI_ISL_578168	2020-09-10	CSIR-Indian Institute of Chemical Biology, MEDICA Supercpecialty Hospital Kolkata	CSIR-Indian Institute of Chemical Biology, MEDICA Supercpecialty Hospital Kolkata	Sujay Krishna Maity et al
EPI_ISL_578175	2020-09-10	CSIR-Indian Institute of Chemical Biology, MEDICA Supercpecialty Hospital Kolkata	CSIR-Indian Institute of Chemical Biology, MEDICA Supercpecialty Hospital Kolkata	Sujay Krishna Maity et al
EPI_ISL_578181	2020-09-10	CSIR-Indian Institute of Chemical Biology, MEDICA Supercpecialty Hospital Kolkata	CSIR-Indian Institute of Chemical Biology, MEDICA Supercpecialty Hospital Kolkata	Sujay Krishna Maity et al
EPI_ISL_578183	2020-09-10	CSIR-Indian Institute of Chemical Biology, MEDICA Supercpecialty Hospital Kolkata	CSIR-Indian Institute of Chemical Biology, MEDICA Supercpecialty Hospital Kolkata	Sujay Krishna Maity et al
EPI_ISL_578184	2020-09-10	CSIR-Indian Institute of Chemical Biology, MEDICA Supercpecialty Hospital Kolkata	CSIR-Indian Institute of Chemical Biology, MEDICA Supercpecialty Hospital Kolkata	Sujay Krishna Maity et al
EPI_ISL_718282	2020-09-10	Institute for Medical Research, Infectious Disease Research Centre, National Institutes of Health, Ministry of Health Malaysia	Institute for Medical Research, Infectious Disease Research Centre, National Institutes of Health, Ministry of Health Malaysia	Suppiah J et al
EPI_ISL_583481	2020-09-10	Institute of Virology, Biomedical Research Center of the Slovak Academy of Sciences, Bratislava	Faculty of Natural Sciences, Comenius University, Bratislava	Viktória Hodorová et al
EPI_ISL_681301	2020-09-11	Communicable Disease Laboratory, Public Health Directorate	Communicable Disease Laboratory, Public Health Directorate	Alwasti et al
EPI_ISL_660485	2020-09-11	Laboratoire de Microbiologie CHU Souro Sanou	Centre Muraz	Abdoul-Salam Ouedraogo et al
EPI_ISL_660502	2020-09-11	Laboratoire de Microbiologie CHU Souro Sanou	Centre Muraz	Abdoul-Salam Ouedraogo et al
EPI_ISL_609804	2020-09-11	Unity Health Toronto	Ontario Institute for Cancer Research	Ramzi Fattouh et al
EPI_ISL_693299	2020-09-11	Department of Microbiology, Yokohama City University School of Medicine	Department of Microbiology, Yokohama City University School of Medicine	Kei Miyakawa et al
EPI_ISL_632405	2020-09-11	Dutch COVID-19 response team	Erasmus Medical Center	Bas Oude Munnink et al
EPI_ISL_678260	2020-09-11	General Hospital - Struga	Research Center for Genetic Engineering and Biotechnology "Georgi D. Efremov" , Macedonian Academy of Sciences and Arts	RCGEB - MASA et al
EPI_ISL_582051	2020-09-11	Servicio de Microbiología. Hospital Universitario Donostia. OSI Donostialdea. Área de Enfermedades Infecciosas, Grupo de Infección Respiratoria y Resistencia Antimicrobiana. Instituto de Investigación Sanitaria Biodonostia	SeqCOVID-SPAIN consortium/IBV(CSIC)	Gustavo Cilla et al
EPI_ISL_569241	2020-09-12	MEPHI, Aix Marseille University	MEPHI, Aix Marseille University	Anthony LEVASSEUR et al
EPI_ISL_572320	2020-09-12	IZSM	IZSM	Maurizio Viscardi et al
EPI_ISL_572321	2020-09-12	IZSM	IZSM	Maurizio Viscardi et al
EPI_ISL_572322	2020-09-12	IZSM	IZSM	Maurizio Viscardi et al
EPI_ISL_572323	2020-09-12	IZSM	IZSM	Maurizio Viscardi et al
EPI_ISL_584072	2020-09-12	IZSM	IZSM	Maurizio Viscardi et al
EPI_ISL_636897	2020-09-12	Lithuanian University of Health Sciences Hospital, Department of Laboratory Medicine	Lithuanian University of Health Sciences, Molecular cardiology lab.	Lukas Zemaitis et al
EPI_ISL_729754	2020-09-12	Connecticut Department of Health	Grubaugh Lab - Yale School of Public Health	Joseph Fauver et al
EPI_ISL_563576	2020-09-13	Microbiological Diagnostic Unit - Public Health Laboratory (MDU-PHL)	MDU-PHL	Seemann et al
EPI_ISL_610218	2020-09-13	Department of Health Technology and Informatics, The Hong Kong Polytechnic University	Department of Health Technology and Informatics, The Hong Kong Polytechnic University	Siu et al
EPI_ISL_582667	2020-09-13	Sheikh Khalifa Medical City	Molecular/Surveillance lab Sheikh Khalifa Medical City	Amirtharaj Francis et al
EPI_ISL_682300	2020-09-14	Communicable Disease Laboratory, Public Health Directorate	Communicable Disease Laboratory, Public Health Directorate	Alwasti et al

EPI_ISL_641557	2020-09-14	Department of Clinical Microbiology	GIGA Medical Genomics	Keith Durkin et al
EPI_ISL_641558	2020-09-14	Department of Clinical Microbiology	GIGA Medical Genomics	Keith Durkin et al
EPI_ISL_574431	2020-09-14	Hospital IESS Babahoyo	Institute of Microbiology, Universidad San Francisco de Quito	Belén Prado-Vivar et al
EPI_ISL_593905	2020-09-14	Labo Analyses Med, Sarcelles	National Reference Center for Viruses of Respiratory Infections, Institut Pasteur, Paris	Sylvie Behillil et al
EPI_ISL_639849	2020-09-14	National Virus Reference Laboratory	National Virus Reference Laboratory	Michael Carr et al
EPI_ISL_639837	2020-09-14	National Virus Reference Laboratory	National Virus Reference Laboratory	Michael Carr et al
EPI_ISL_639828	2020-09-14	National Virus Reference Laboratory	National Virus Reference Laboratory	Michael Carr et al
EPI_ISL_693479	2020-09-14	Central Public Health Laboratory	National Public Health Laboratory, National Centre for Infectious Diseases	Tze Minn Mak et al
EPI_ISL_708812	2020-09-14	World Medical Hospital	National Institute of Health, Department of Medical Sciences, Ministry of Public Health, Thailand	Pilailuk Okada et al
EPI_ISL_717700	2020-09-14	Trinidad Public Health Laboratory	Carrington Lab, Department of PreClinical Sciences, Faculty of Medical Sciences, The University of the West Indies	Nikita S. D. Sahadeo et al
EPI_ISL_582671	2020-09-14	Sheikh Khalifa Medical City	Molecular/Surveillance lab Sheikh Khalifa Medical City	Amirtharaj Francis et al
EPI_ISL_577630	2020-09-15	The National Institute of Public Health	State Veterinary Institute Prague	Nagy et al
EPI_ISL_626580	2020-09-15	The National Institute of Public Health	State Veterinary Institute Prague	Nagy et al
EPI_ISL_626582	2020-09-15	The National Institute of Public Health	State Veterinary Institute Prague	Nagy et al
EPI_ISL_626583	2020-09-15	The National Institute of Public Health	State Veterinary Institute Prague	Nagy et al
EPI_ISL_693481	2020-09-15	Central Public Health Laboratory	National Public Health Laboratory, National Centre for Infectious Diseases	Tze Minn Mak et al
EPI_ISL_700328	2020-09-16	Child Health Research Foundation	Child Health Research Foundation	Senjuti Saha et al
EPI_ISL_660509	2020-09-16	Laboratoire de Microbiologie CHU Souro Sanou	Centre Muraz	Abdoul-Salam Ouedraogo et al
EPI_ISL_671974	2020-09-16	Laboratorio de Virología y Microbiología Molecular, Depto. de Microbiología, Facultad de Medicina, Universidad de El Salvador/INS-laboratorio de Ref. Ministerio de Salud	Laboratorio de Virología y Microbiología Molecular, Depto. de Microbiología, Facultad de Medicina, Universidad de El Salvador/INS-laboratorio de Ref. Ministerio de Salud	Rivera NR et al
EPI_ISL_578244	2020-09-16	National Virus Reference Laboratory	National Virus Reference Laboratory	Michael Carr et al
EPI_ISL_677634	2020-09-16	Virology Unit, Institut Pasteur de Madagascar	Virology Unit, Institut Pasteur de Madagascar	Christian Ranaivoson et al
EPI_ISL_729871	2020-09-16	Instituto de Medicina Tropical, Universidad Nacional Toribio Rodríguez de Mendoza de Amazonas	Laboratorio de Genómica Microbiana, Universidad Peruana Cayetano Heredia	Pablo Tsukayama et al
EPI_ISL_645116	2020-09-16	National Public Health Laboratory, National Centre for Infectious Diseases	National Public Health Laboratory, National Centre for Infectious Diseases	Tze Minn Mak et al
EPI_ISL_583486	2020-09-16	Institute of Virology, Biomedical Research Center of the Slovak Academy of Sciences, Bratislava	Faculty of Natural Sciences, Comenius University, Bratislava	Kristína Boršová et al
EPI_ISL_583487	2020-09-16	Institute of Virology, Biomedical Research Center of the Slovak Academy of Sciences, Bratislava	Faculty of Natural Sciences, Comenius University, Bratislava	Broňa Brejová et al
EPI_ISL_583488	2020-09-16	Institute of Virology, Biomedical Research Center of the Slovak Academy of Sciences, Bratislava	Faculty of Natural Sciences, Comenius University, Bratislava	Broňa Brejová et al
EPI_ISL_583489	2020-09-16	Institute of Virology, Biomedical Research Center of the Slovak Academy of Sciences, Bratislava	Faculty of Natural Sciences, Comenius University, Bratislava	Viktória Hodorová et al
EPI_ISL_594149	2020-09-16	Klinsisk mikrobiologi Linkoping	The Public Health Agency of Sweden	Anna-Malin Linde et al
EPI_ISL_594150	2020-09-16	Klinsisk mikrobiologi Linkoping	The Public Health Agency of Sweden	Anna-Malin Linde et al
EPI_ISL_594151	2020-09-16	Klinsisk mikrobiologi Linkoping	The Public Health Agency of Sweden	Anna-Malin Linde et al
EPI_ISL_582674	2020-09-16	Sheikh Khalifa Medical City	Molecular/Surveillance lab Sheikh Khalifa Medical City	Amirtharaj Francis et al
EPI_ISL_682303	2020-09-17	Communicable Disease Laboratory, Public Health Directorate	Communicable Disease Laboratory, Public Health Directorate	Alwasti et al
EPI_ISL_625673	2020-09-17	Laboratory of Molecular Medicine, University of Magallanes	Centro Asistencial Docente y de Investigacion, Universidad de Magallanes	Jorge Gonzalez et al

EPI_ISL_637015	2020-09-17	Department of Infectious Diseases and Immunology, National Hospital Organization Nagoya Medical Center	Clinical Research Center, National Hospital Organization Nagoya Medical Center	Yoshihiro Nakata et al
EPI_ISL_632504	2020-09-17	Dutch COVID-19 response team	Erasmus Medical Center	Bas Oude Munnink et al
EPI_ISL_729906	2020-09-17	Instituto de Medicina Tropical, Universidad Nacional Toribio Rodríguez de Mendoza de Amazonas	Laboratorio de Genómica Microbiana, Universidad Peruana Cayetano Heredia	Pablo Tsukayama et al
EPI_ISL_654487	2020-09-17	Servicio de Microbiología. Hospital Clínico Universitario de Valencia	SeqCOVID-SPAIN consortium/IBV(CSIC)	David Navarro Ortega et al
EPI_ISL_667809	2020-09-17	Laboratory Medicine	Department of Laboratory Medicine, Lin-Kou Chang Gung Memorial Hospital, Taoyuan, Taiwan	Kuo-Chien Tsao et al
EPI_ISL_582675	2020-09-17	Sheikh Khalifa Medical City	Molecular/Surveillance lab Sheikh Khalifa Medical City	Amirtharaj Francis et al
EPI_ISL_697791	2020-09-18	Institute of Microbiology, Universidad San Francisco de Quito	Institute of Microbiology, Universidad San Francisco de Quito	Belén Prado-Vivar et al
EPI_ISL_576587	2020-09-18	Oxford Viromics, NDM, University of Oxford; Oxford University Hospitals; Basingstoke and North Hampshire Hospital	COVID-19 Genomics UK (COG-UK) Consortium	Tanya Golubchik et al
EPI_ISL_576688	2020-09-18	Oxford Viromics, NDM, University of Oxford; Oxford University Hospitals; Basingstoke and North Hampshire Hospital	COVID-19 Genomics UK (COG-UK) Consortium	Tanya Golubchik et al
EPI_ISL_576693	2020-09-18	Oxford Viromics, NDM, University of Oxford; Oxford University Hospitals; Basingstoke and North Hampshire Hospital	COVID-19 Genomics UK (COG-UK) Consortium	Tanya Golubchik et al
EPI_ISL_578026	2020-09-18	Dutch COVID-19 response team	Erasmus Medical Center	Bas Oude Munnink et al
EPI_ISL_592747	2020-09-19	Microbiological Diagnostic Unit - Public Health Laboratory (MDU-PHL)	MDU-PHL	Seemann T. et al
EPI_ISL_625677	2020-09-19	Laboratory of Molecular Medicine, University of Magallanes	Centro Asistencial Docente y de Investigacion, Universidad de Magallanes	Jorge Gonzalez et al
EPI_ISL_590933	2020-09-19	Medical Microbiology Unit, Department for Laboratory Medicine, Drammen Hospital, Vestre Viken Health Trust,	Norwegian Institute of Public Health, Department of Virology	Kathrine Stene-Johansen et al
EPI_ISL_625683	2020-09-20	National Reference Laboratory for COVID-19, Pasteur Institute of Iran	National Reference Laboratory for COVID-19, Pasteur Institute of Iran	Zahra Ahmadi et al
EPI_ISL_710532	2020-09-20	Hôpital Fattouma-Bourguiba de Monastir	Laboratoire des Procédés de Criblage Moléculaire et Cellulaire-Centre de Biotechnologie de Sfax	Souissi et al
EPI_ISL_700336	2020-09-21	Child Health Research Foundation	Child Health Research Foundation	Senjuti Saha et al
EPI_ISL_621746	2020-09-21	Department of Virus and Microbiological Special Diagnostics, Statens Serum Institut, Denmark	Albertsen lab, Department of Chemistry and Bioscience, Aalborg University, Denmark	Danish Covid-19 Genome Consortia et al
EPI_ISL_620833	2020-09-21	Department of Virus and Microbiological Special Diagnostics, Statens Serum Institut, Denmark	Albertsen lab, Department of Chemistry and Bioscience, Aalborg University, Denmark	Danish Covid-19 Genome Consortia et al
EPI_ISL_620844	2020-09-21	Department of Virus and Microbiological Special Diagnostics, Statens Serum Institut, Denmark	Albertsen lab, Department of Chemistry and Bioscience, Aalborg University, Denmark	Danish Covid-19 Genome Consortia et al
EPI_ISL_593934	2020-09-21	Sentinelles, Chanteloup-En-Brie	National Reference Center for Viruses of Respiratory Infections, Institut Pasteur, Paris	Sylvie Behillil et al
EPI_ISL_639653	2020-09-21	E. Gulbja Laboratorija	Latvian Biomedical Research and Study Centre	Ivars Silamiķelis et al
EPI_ISL_578060	2020-09-21	Dutch COVID-19 response team	Erasmus Medical Center	Bas Oude Munnink et al
EPI_ISL_729869	2020-09-21	Instituto de Medicina Tropical, Universidad Nacional Toribio Rodríguez de Mendoza de Amazonas	Laboratorio de Genómica Microbiana, Universidad Peruana Cayetano Heredia	Pablo Tsukayama et al
EPI_ISL_729909	2020-09-21	Instituto de Medicina Tropical, Universidad Nacional Toribio Rodríguez de Mendoza de Amazonas	Laboratorio de Genómica Microbiana, Universidad Peruana Cayetano Heredia	Pablo Tsukayama et al
EPI_ISL_729911	2020-09-21	Instituto de Medicina Tropical, Universidad Nacional Toribio Rodríguez de Mendoza de Amazonas	Laboratorio de Genómica Microbiana, Universidad Peruana Cayetano Heredia	Pablo Tsukayama et al
EPI_ISL_729915	2020-09-21	Instituto de Medicina Tropical, Universidad Nacional Toribio Rodríguez de Mendoza de Amazonas	Laboratorio de Genómica Microbiana, Universidad Peruana Cayetano Heredia	Pablo Tsukayama et al
EPI_ISL_602423	2020-09-21	HELIX LLC	WHO National Influenza Centre Russian Federation	Andrey Komissarov et al
EPI_ISL_661287	2020-09-21	Gavle klinisk mikrobiologi	The Public Health Agency of Sweden	Department of Microbiology et al
EPI_ISL_712068	2020-09-21	Laboratoire de Microbiologie- CHU Habib Bourguiba – Sfax adresse	Laboratoire des Procédés de Criblage Moléculaire et Cellulaire-Centre de Biotechnologie de Sfax	Souissi et al

EPI_ISL_614347	2020-09-22	Molecular diagnostic unit for viral haemorrhagic fevers and emerging viruses, Bouaké CHU Laboratory	Project group Epidemology of Highly Pathogenic Microorganisms, Robert Koch-Institute	Chantal Akoua-Koffi et al
EPI_ISL_697787	2020-09-22	Institute of Microbiology, Universidad San Francisco de Quito	Institute of Microbiology, Universidad San Francisco de Quito	Andrea Macias et al
EPI_ISL_729602	2020-09-22	Charité Universitätsmedizin Berlin, Institut für Virologie/Labor Berlin	Charité Universitätsmedizin Berlin, Institut für Virologie	Victor M Corman et al
EPI_ISL_637016	2020-09-22	Department of Infectious Diseases and Immunology, National Hospital Organization Nagoya Medical Center	Clinical Research Center, National Hospital Organization Nagoya Medical Center	Yoshihiro Nakata et al
EPI_ISL_631359	2020-09-23	ZOTZ KLIMAS MVZ Düsseldorf-Centrum GbR ÜBAG für Labormedizin, Genetik, Zytologie, Pathologie	Center of Medical Microbiology, Virology, and Hospital Hygiene, University of Duesseldorf	Maximilian Damagnez et al
EPI_ISL_639644	2020-09-23	E. Gulbja Laboratorija	Latvian Biomedical Research and Study Centre	Ivars Silamiķelis et al
EPI_ISL_639646	2020-09-23	E. Gulbja Laboratorija	Latvian Biomedical Research and Study Centre	Ivars Silamiķelis et al
EPI_ISL_640129	2020-09-23	Groote Schuur Hospital wc GSH	NHLS/UCT	Arash Iranzadeh et al
EPI_ISL_640130	2020-09-23	Groote Schuur Hospital wc GSH	NHLS/UCT	Arash Iranzadeh et al
EPI_ISL_582685	2020-09-23	Sheikh Khalifa Medical City	Molecular/Surveillance lab Sheikh Khalifa Medical City	Amirtharaj Francis et al
EPI_ISL_631361	2020-09-24	ZOTZ KLIMAS MVZ Düsseldorf-Centrum GbR ÜBAG für Labormedizin, Genetik, Zytologie, Pathologie	Center of Medical Microbiology, Virology, and Hospital Hygiene, University of Duesseldorf	Maximilian Damagnez et al
EPI_ISL_610194	2020-09-24	Department of Health Technology and Informatics, The Hong Kong Polytechnic University	Department of Health Technology and Informatics, The Hong Kong Polytechnic University	Siu et al
EPI_ISL_610195	2020-09-24	Department of Health Technology and Informatics, The Hong Kong Polytechnic University	Department of Health Technology and Informatics, The Hong Kong Polytechnic University	Siu et al
EPI_ISL_708815	2020-09-24	Urban Institute for Disease Prevention and Control	National Institute of Health, Department of Medical Sciences, Ministry of Public Health, Thailand	Pilailuk Okada et al
EPI_ISL_591273	2020-09-24	National Institute for Viral Disease Control and Prevention, China CDC	National Institute for Viral Disease Control and Prevention, China CDC	Huilai Ma et al
EPI_ISL_591276	2020-09-24	National Institute for Viral Disease Control and Prevention, China CDC	National Institute for Viral Disease Control and Prevention, China CDC	Huilai Ma et al
EPI_ISL_591277	2020-09-24	National Institute for Viral Disease Control and Prevention, China CDC	National Institute for Viral Disease Control and Prevention, China CDC	Huilai Ma et al
EPI_ISL_591278	2020-09-24	National Institute for Viral Disease Control and Prevention, China CDC	National Institute for Viral Disease Control and Prevention, China CDC	Huilai Ma et al
EPI_ISL_637017	2020-09-25	Department of Infectious Diseases and Immunology, National Hospital Organization Nagoya Medical Center	Clinical Research Center, National Hospital Organization Nagoya Medical Center	Yoshihiro Nakata et al
EPI_ISL_625674	2020-09-27	Laboratory of Molecular Medicine, University of Magallanes	Centro Asistencial Docente y de Investigacion, Universidad de Magallanes	Jorge Gonzalez et al
EPI_ISL_591011	2020-09-27	Oslo University Hospital, Department of Medical Microbiology	Norwegian Institute of Public Health, Department of Virology	Kathrine Stene-Johansen et al
EPI_ISL_591279	2020-09-27	National Institute for Viral Disease Control and Prevention, China CDC	National Institute for Viral Disease Control and Prevention, China CDC	Huilai Ma et al
EPI_ISL_717809	2020-09-28	LACEN Dr. Francisco Rimolo Neto	Bioinformatics Laboratory / LNCC	Carolina M Voloch et al
EPI_ISL_620923	2020-09-28	Department of Virus and Microbiological Special Diagnostics, Statens Serum Institut, Denmark	Albertsen lab, Department of Chemistry and Bioscience, Aalborg University, Denmark	Danish Covid-19 Genome Consortia et al
EPI_ISL_620953	2020-09-28	Department of Virus and Microbiological Special Diagnostics, Statens Serum Institut, Denmark	Albertsen lab, Department of Chemistry and Bioscience, Aalborg University, Denmark	Danish Covid-19 Genome Consortia et al
EPI_ISL_610197	2020-09-28	Department of Health Technology and Informatics, The Hong Kong Polytechnic University	Department of Health Technology and Informatics, The Hong Kong Polytechnic University	Siu et al
EPI_ISL_639668	2020-09-28	E. Gulbja Laboratorija	Latvian Biomedical Research and Study Centre	Ivars Silamiķelis et al
EPI_ISL_693480	2020-09-28	Central Public Health Laboratory	National Public Health Laboratory, National Centre for Infectious Diseases	Tze Minn Mak et al
EPI_ISL_729903	2020-09-28	Instituto de Medicina Tropical, Universidad Nacional Toribio Rodríguez de Mendoza de Amazonas	Laboratorio de Genómica Microbiana, Universidad Peruana Cayetano Heredia	Pablo Tsukayama et al
EPI_ISL_729907	2020-09-28	Instituto de Medicina Tropical, Universidad Nacional Toribio Rodríguez de Mendoza de Amazonas	Laboratorio de Genómica Microbiana, Universidad Peruana Cayetano Heredia	Pablo Tsukayama et al

EPI_ISL_639962	2020-09-28	HELIX LLC	WHO National Influenza Centre Russian Federation	Andrey Komissarov et al
EPI_ISL_654194	2020-09-28	Hospital General Universitario Gregorio Marañón	SeqCOVID-SPAIN consortium/IBV(CSIC)	Darío García de Viedma et al
EPI_ISL_710566	2020-09-29	University Hospital Dubrava	Ruđer Boškovic Institute; Forensic Science Centre Ivan Vučetić; University of Zagreb Faculty of Science	Robert Belužić et al
EPI_ISL_626589	2020-09-29	The National Institute of Public Health	State Veterinary Institute Prague	Nagy et al
EPI_ISL_697794	2020-09-29	Institute of Microbiology, Universidad San Francisco de Quito	Institute of Microbiology, Universidad San Francisco de Quito	Andrea Macias et al
EPI_ISL_697795	2020-09-29	Institute of Microbiology, Universidad San Francisco de Quito	Institute of Microbiology, Universidad San Francisco de Quito	Andrea Macias et al
EPI_ISL_639676	2020-09-29	Centrālā Laboratorija	Latvian Biomedical Research and Study Centre	Ivars Silamiķelis et al
EPI_ISL_718285	2020-09-29	Institute for Medical Research, Infectious Disease Research Centre, National Institutes of Health, Ministry of Health Malaysia	Institute for Medical Research, Infectious Disease Research Centre, National Institutes of Health, Ministry of Health Malaysia	Suppiah J et al
EPI_ISL_718310	2020-09-29	Institute for Medical Research, Infectious Disease Research Centre, National Institutes of Health, Ministry of Health Malaysia	Institute for Medical Research, Infectious Disease Research Centre, National Institutes of Health, Ministry of Health Malaysia	Suppiah J et al
EPI_ISL_718286	2020-09-29	Institute for Medical Research, Infectious Disease Research Centre, National Institutes of Health, Ministry of Health Malaysia	Institute for Medical Research, Infectious Disease Research Centre, National Institutes of Health, Ministry of Health Malaysia	Suppiah J et al
EPI_ISL_712568	2020-09-29	Laboratoire de Microbiologie- CHU Habib Bourguiba – Sfax adresse	Laboratoire des Procédés de Criblage Moléculaire et Cellulaire-Centre de Biotechnologie de Sfax	Souissi et al
EPI_ISL_644925	2020-09-29	Virginia DCLS	Virginia DCLS	Virginia DCLS et al
EPI_ISL_710569	2020-09-30	University Hospital Dubrava	Ruđer Boškovic Institute; Forensic Science Centre Ivan Vučetić; University of Zagreb Faculty of Science	Robert Belužić et al
EPI_ISL_631333	2020-09-30	ZOTZ KLIMAS MVZ Düsseldorf-Centrum GbR ÜBAG für Labormedizin, Genetik, Zytologie, Pathologie	Center of Medical Microbiology, Virology, and Hospital Hygiene, University of Duesseldorf	Maximilian Damagnez et al
EPI_ISL_707778	2020-09-30	Medical Research Center, Faculty of Medicine, Syarif Hidayatullah State Islamic University Jakarta	Medical Research Center, Faculty of Medicine, Syarif Hidayatullah State Islamic University Jakarta	Erike A Suwarsono et al
EPI_ISL_635136	2020-09-30	Department of Medical Microbiology, St. Olavs hospital	Norwegian Institute of Public Health, Department of Virology	Kathrine Stene-Johansen et al
EPI_ISL_603280	2020-09-30	Viollier AG	Department of Biosystems Science and Engineering, ETH Zürich	Christian Beisel et al
EPI_ISL_603269	2020-09-30	Viollier AG	Department of Biosystems Science and Engineering, ETH Zürich	Christian Beisel et al
EPI_ISL_671655	2020-10-01	Unity Health Toronto	Ontario Institute for Cancer Research	Ramzi Fattouh et al
EPI_ISL_710570	2020-10-01	University Hospital Dubrava	Ruđer Boškovic Institute; Forensic Science Centre Ivan Vučetić; University of Zagreb Faculty of Science	Robert Belužić et al
EPI_ISL_660529	2020-10-01	Institute of Microbiology, Universidad San Francisco de Quito	Institute of Microbiology, Universidad San Francisco de Quito	Sully Márquez et al
EPI_ISL_660530	2020-10-01	Institute of Microbiology, Universidad San Francisco de Quito	Institute of Microbiology, Universidad San Francisco de Quito	Sully Márquez et al
EPI_ISL_660531	2020-10-01	Institute of Microbiology, Universidad San Francisco de Quito	Institute of Microbiology, Universidad San Francisco de Quito	Sully Márquez et al
EPI_ISL_639897	2020-10-01	National Virus Reference Laboratory	National Virus Reference Laboratory	Michael Carr et al
EPI_ISL_718215	2020-10-01	Ministry of Health Hospitals	Institute of Health and Community Medicine	David Perera et al
EPI_ISL_632572	2020-10-01	Dutch COVID-19 response team	Erasmus Medical Center	Bas Oude Munnink et al
EPI_ISL_603296	2020-10-01	Viollier AG	Department of Biosystems Science and Engineering, ETH Zürich	Christian Beisel et al
EPI_ISL_603290	2020-10-01	Viollier AG	Department of Biosystems Science and Engineering, ETH Zürich	Christian Beisel et al
EPI_ISL_671656	2020-10-02	Unity Health Toronto	Ontario Institute for Cancer Research	Ramzi Fattouh et al
EPI_ISL_710571	2020-10-02	University Hospital Dubrava	Ruđer Boškovic Institute; Forensic Science Centre Ivan Vučetić; University of Zagreb Faculty of Science	Robert Belužić et al
EPI_ISL_660532	2020-10-02	Institute of Microbiology, Universidad San Francisco de Quito	Institute of Microbiology, Universidad San Francisco de Quito	Sully Márquez et al
EPI_ISL_660539	2020-10-02	Institute of Microbiology, Universidad San Francisco de Quito	Institute of Microbiology, Universidad San Francisco de Quito	Sully Márquez et al
EPI_ISL_631342	2020-10-02	ZOTZ KLIMAS MVZ Düsseldorf-Centrum GbR ÜBAG für Labormedizin, Genetik, Zytologie, Pathologie	Center of Medical Microbiology, Virology, and Hospital Hygiene, University of Duesseldorf	Maximilian Damagnez et al
EPI_ISL_610215	2020-10-02	Department of Health Technology and Informatics, The Hong Kong Polytechnic University	Department of Health Technology and Informatics, The Hong Kong Polytechnic University	Siu et al
EPI_ISL_677785	2020-10-02	University of Szeged, Institute of Clinical Microbiology	National Laboratory of Virology, Szentágothai Research Centre	Endre Gábor Tóth et al

EPI_ISL_677807	2020-10-02	University of Szeged, Institute of Clinical Microbiology	National Laboratory of Virology, Szentágothai Research Centre	Endre Gábor Tóth et al
EPI_ISL_635124	2020-10-02	Innlandet Hospital Trust, Division Lillehammer, Department for Medical Microbiology	Norwegian Institute of Public Health, Department of Virology	Kathrine Stene-Johansen et al
EPI_ISL_596473	2020-10-02	National Public Health Laboratory, National Centre for Infectious Diseases	National Public Health Laboratory, National Centre for Infectious Diseases	Tze Minn Mak et al
EPI_ISL_596480	2020-10-02	National Public Health Laboratory, National Centre for Infectious Diseases	National Public Health Laboratory, National Centre for Infectious Diseases	Tze Minn Mak et al
EPI_ISL_676534	2020-10-02	Kalmar klinisk mikrobiologi	The Public Health Agency of Sweden	Department of Microbiology et al
EPI_ISL_640519	2020-10-03	Victorian Infectious Diseases Reference Laboratory (VIDRL)	VIDRL and MDU-PHL	Caly L. et al
EPI_ISL_671665	2020-10-03	Unity Health Toronto	Ontario Institute for Cancer Research	Ramzi Fattouh et al
EPI_ISL_579117	2020-10-03	LabPLUS	Institute of Environmental Science and Research (ESR)	Xiaoyun Ren et al
EPI_ISL_602564	2020-10-03	Centre for Dengue Research, Department of Immunology and Molecular Medicine	Centre for Dengue Research	Chandima Jeewandara et al
EPI_ISL_641220	2020-10-04	Microbiological Diagnostic Unit - Public Health Laboratory (MDU-PHL)	MDU-PHL	Seemann T. et al
EPI_ISL_671659	2020-10-04	Unity Health Toronto	Ontario Institute for Cancer Research	Ramzi Fattouh et al
EPI_ISL_671667	2020-10-04	Unity Health Toronto	Ontario Institute for Cancer Research	Ramzi Fattouh et al
EPI_ISL_710573	2020-10-04	University Hospital Dubrava	Ruđer Boškovic Institute; Forensic Science Centre Ivan Vučetić; University of Zagreb Faculty of Science	Robert Belužić et al
EPI_ISL_710572	2020-10-04	University Hospital Dubrava	Ruđer Boškovic Institute; Forensic Science Centre Ivan Vučetić; University of Zagreb Faculty of Science	Robert Belužić et al
EPI_ISL_707692	2020-10-04	Medical Research Center, Faculty of Medicine, Syarif Hidayatullah State Islamic University Jakarta	Medical Research Center, Faculty of Medicine, Syarif Hidayatullah State Islamic University Jakarta	Chris Adhiyanto et al
EPI_ISL_635172	2020-10-04	Oslo University Hospital, Department of Medical Microbiology	Norwegian Institute of Public Health, Department of Virology	Kathrine Stene-Johansen et al
EPI_ISL_660545	2020-10-04	Laboratory Medicine	Department of Laboratory Medicine, Lin-Kou Chang Gung Memorial Hospital, Taoyuan, Taiwan	Kuo-Chien Tsao et al
EPI_ISL_710574	2020-10-05	University Hospital Dubrava	Ruđer Boškovic Institute; Forensic Science Centre Ivan Vučetić; University of Zagreb Faculty of Science	Robert Belužić et al
EPI_ISL_660533	2020-10-05	Institute of Microbiology, Universidad San Francisco de Quito	Institute of Microbiology, Universidad San Francisco de Quito	Sully Márquez et al
EPI_ISL_660534	2020-10-05	Institute of Microbiology, Universidad San Francisco de Quito	Institute of Microbiology, Universidad San Francisco de Quito	Sully Márquez et al
EPI_ISL_628546	2020-10-05	Oxford Viromics, NDM, University of Oxford; Oxford University Hospitals; Basingstoke and North Hampshire Hospital	COVID-19 Genomics UK (COG-UK) Consortium	Tanya Golubchik et al
EPI_ISL_610199	2020-10-05	Department of Health Technology and Informatics, The Hong Kong Polytechnic University	Department of Health Technology and Informatics, The Hong Kong Polytechnic University	Siu et al
EPI_ISL_610200	2020-10-05	Department of Health Technology and Informatics, The Hong Kong Polytechnic University	Department of Health Technology and Informatics, The Hong Kong Polytechnic University	Siu et al
EPI_ISL_637018	2020-10-05	Department of Infectious Diseases and Immunology, National Hospital Organization Nagoya Medical Center	Clinical Research Center, National Hospital Organization Nagoya Medical Center	Yoshihiro Nakata et al
EPI_ISL_639669	2020-10-05	E. Gulbja Laboratorija	Latvian Biomedical Research and Study Centre	Ivars Silamiķelis et al
EPI_ISL_729982	2020-10-05	Nigeria Centre for Disease Control (NCDC)	African Centre of Excellence for Genomics of Infectious Diseases (ACEGID), Redeemer's University, Ede, Osun State, Nigeria	Oluniyi P.E. et al et al
EPI_ISL_708818	2020-10-05	Urban Institute for Disease Prevention and Control	National Institute of Health, Department of Medical Sciences, Ministry of Public Health, Thailand	Pilailuk Okada et al
EPI_ISL_708816	2020-10-05	Urban Institute for Disease Prevention and Control	National Institute of Health, Department of Medical Sciences, Ministry of Public Health, Thailand	Pilailuk Okada et al
EPI_ISL_609758	2020-10-06	Lighthouse Lab in Cambridge	Wellcome Sanger Institute for the COVID-19 Genomics UK (COG-UK) consortium	Rob Howes et al
EPI_ISL_631318	2020-10-06	ZOTZ KLIMAS MVZ Düsseldorf-Centrum GbR ÜBAG für Labormedizin, Genetik, Zytologie, Pathologie	Center of Medical Microbiology, Virology, and Hospital Hygiene, University of Duesseldorf	Maximilian Damagnez et al
EPI_ISL_671483	2020-10-06	University of Debrecen, Department of Medical Microbiology	National Laboratory of Virology, Szentágothai Research Centre	Endre Gábor Tóth et al

EPI_ISL_639692	2020-10-06	E. Gulbja Laboratorija	Latvian Biomedical Research and Study Centre	Ivars Silamiķelis et al
EPI_ISL_693482	2020-10-06	Central Public Health Laboratory	National Public Health Laboratory, National Centre for Infectious Diseases	Tze Minn Mak et al
EPI_ISL_693483	2020-10-06	Central Public Health Laboratory	National Public Health Laboratory, National Centre for Infectious Diseases	Tze Minn Mak et al
EPI_ISL_629004	2020-10-07	South Eastern Area Laboratory Services (SEALS)	NSW Health Pathology - Institute of Clinical Pathology and Medical Research; Westmead Hospital; University of Sydney	CIDM-PH et al. et al
EPI_ISL_626595	2020-10-07	The National Institute of Public Health	State Veterinary Institute Prague	Nagy et al
EPI_ISL_626605	2020-10-07	The National Institute of Public Health	State Veterinary Institute Prague	Nagy et al
EPI_ISL_598139	2020-10-07	Lighthouse Lab in Alderley Park	Wellcome Sanger Institute for the COVID-19 Genomics UK (COG-UK) consortium	Jacquelyn Wynn et al
EPI_ISL_639687	2020-10-07	E. Gulbja Laboratorija	Latvian Biomedical Research and Study Centre	Ivars Silamiķelis et al
EPI_ISL_639693	2020-10-07	E. Gulbja Laboratorija	Latvian Biomedical Research and Study Centre	Ivars Silamiķelis et al
EPI_ISL_591280	2020-10-07	National Institute for Viral Disease Control and Prevention, China CDC	National Institute for Viral Disease Control and Prevention, China CDC	Huilai Ma et al
EPI_ISL_603240	2020-10-08	National Institute of Laboratory Medicine and Referral Center	Genomic Research Lab, BCSIR	Tanjina Akhter Banu et al
EPI_ISL_610223	2020-10-08	Department of Health Technology and Informatics, The Hong Kong Polytechnic University	Department of Health Technology and Informatics, The Hong Kong Polytechnic University	Siu et al
EPI_ISL_678389	2020-10-08	University of Debrecen, Department of Medical Microbiology	National Laboratory of Virology, Szentágothai Research Centre	Endre Gábor Tóth et al
EPI_ISL_678396	2020-10-08	University of Debrecen, Department of Medical Microbiology	National Laboratory of Virology, Szentágothai Research Centre	Endre Gábor Tóth et al
EPI_ISL_639690	2020-10-08	E. Gulbja Laboratorija	Latvian Biomedical Research and Study Centre	Ivars Silamiķelis et al
EPI_ISL_648130	2020-10-08	Uppsala klinisk mikrobiologi	The Public Health Agency of Sweden	Anna-Malin Linde et al
EPI_ISL_603550	2020-10-08	Viollier AG	Department of Biosystems Science and Engineering, ETH Zürich	Christian Beisel et al
EPI_ISL_603496	2020-10-08	Viollier AG	Department of Biosystems Science and Engineering, ETH Zürich	Christian Beisel et al
EPI_ISL_717925	2020-10-09	Laboratorio de Virologia Molecular / UFRJ	Bioinformatics Laboratory / LNCC	Carolina M Voloch et al
EPI_ISL_660521	2020-10-09	Laboratoire de Microbiologie CHU Souro Sanou	Centre Muraz	Abdoul-Salam Ouedraogo et al
EPI_ISL_660488	2020-10-09	Laboratoire de Microbiologie CHU Souro Sanou	Centre Muraz	Abdoul-Salam Ouedraogo et al
EPI_ISL_625678	2020-10-09	Laboratory of Molecular Medicine, University of Magallanes	Centro Asistencial Docente y de Investigacion, Universidad de Magallanes	Jorge Gonzalez et al
EPI_ISL_625679	2020-10-09	Laboratory of Molecular Medicine, University of Magallanes	Centro Asistencial Docente y de Investigacion, Universidad de Magallanes	Jorge Gonzalez et al
EPI_ISL_729984	2020-10-09	Nigeria Centre for Disease Control (NCDC)	African Centre of Excellence for Genomics of Infectious Diseases (ACEGID), Redeemer's University, Ede, Osun State, Nigeria	Oluniyi P.E. et al et al
EPI_ISL_640138	2020-10-09	Groote Schuur Hospital wc GSH	NHLS/UCT	Arash Iranzadeh et al
EPI_ISL_640091	2020-10-09	Du Noon CDC wc DNC	NHLS/UCT	Arash Iranzadeh et al
EPI_ISL_640543	2020-10-10	Microbiological Diagnostic Unit - Public Health Laboratory (MDU-PHL)	MDU-PHL	Seemann T. et al
EPI_ISL_603242	2020-10-10	National Institute of Laboratory Medicine and Referral Center	Genomic Research Lab, BCSIR	Barna Goswami et al
EPI_ISL_603243	2020-10-10	National Institute of Laboratory Medicine and Referral Center	Genomic Research Lab, BCSIR	Barna Goswami et al
EPI_ISL_603244	2020-10-10	National Institute of Laboratory Medicine and Referral Center	Genomic Research Lab, BCSIR	Iffat Jahan et al
EPI_ISL_603245	2020-10-10	National Institute of Laboratory Medicine and Referral Center	Genomic Research Lab, BCSIR	Iffat Jahan et al
EPI_ISL_660536	2020-10-10	Institute of Microbiology, Universidad San Francisco de Quito	Institute of Microbiology, Universidad San Francisco de Quito	Sully Márquez et al
EPI_ISL_660537	2020-10-10	Institute of Microbiology, Universidad San Francisco de Quito	Institute of Microbiology, Universidad San Francisco de Quito	Sully Márquez et al
EPI_ISL_660538	2020-10-10	Institute of Microbiology, Universidad San Francisco de Quito	Institute of Microbiology, Universidad San Francisco de Quito	Sully Márquez et al
EPI_ISL_644547	2020-10-10	MEPHI, Aix Marseille University	MEPHI, Aix Marseille University	Anthony LEVASSEUR et al
EPI_ISL_660156	2020-10-10	PathCare	National Health Laboratory Service (NHLS), Tygerberg	Susan Engelbrecht et al

EPI_ISL_626614	2020-10-11	The National Institute of Public Health	State Veterinary Institute Prague	Nagy et al
EPI_ISL_610224	2020-10-11	Department of Health Technology and Informatics, The Hong Kong Polytechnic University	Department of Health Technology and Informatics, The Hong Kong Polytechnic University	Siu et al
EPI_ISL_671878	2020-10-11	National Virus Reference Laboratory	National Virus Reference Laboratory	Michael Carr et al
EPI_ISL_671879	2020-10-11	National Virus Reference Laboratory	National Virus Reference Laboratory	Michael Carr et al
EPI_ISL_708820	2020-10-11	Vajira Hospital	National Institute of Health, Department of Medical Sciences, Ministry of Public Health, Thailand	Pilailuk Okada et al
EPI_ISL_634882	2020-10-12	Lab voor klinische biologie	Onderzoeksgroep Virologie	Laurens Lambrechts et al
EPI_ISL_626620	2020-10-12	The National Institute of Public Health	State Veterinary Institute Prague	Nagy et al
EPI_ISL_619236	2020-10-12	Department of Virus and Microbiological Special Diagnostics, Statens Serum Institut, Denmark	Albertsen lab, Department of Chemistry and Bioscience, Aalborg University, Denmark	Danish Covid-19 Genome Consortia et al
EPI_ISL_619237	2020-10-12	Department of Virus and Microbiological Special Diagnostics, Statens Serum Institut, Denmark	Albertsen lab, Department of Chemistry and Bioscience, Aalborg University, Denmark	Danish Covid-19 Genome Consortia et al
EPI_ISL_618494	2020-10-12	Department of Virus and Microbiological Special Diagnostics, Statens Serum Institut, Denmark	Albertsen lab, Department of Chemistry and Bioscience, Aalborg University, Denmark	Danish Covid-19 Genome Consortia et al
EPI_ISL_610690	2020-10-12	Lighthouse Lab in Cambridge	Wellcome Sanger Institute for the COVID-19 Genomics UK (COG-UK) consortium	Rob Howes et al
EPI_ISL_614285	2020-10-12	General practitioner	National Reference Center for Viruses of Respiratory Infections, Institut Pasteur, Paris	Marion Barbet et al
EPI_ISL_644553	2020-10-12	MEPHI, Aix Marseille University	MEPHI, Aix Marseille University	Anthony LEVASSEUR et al
EPI_ISL_644555	2020-10-12	MEPHI, Aix Marseille University	MEPHI, Aix Marseille University	Anthony LEVASSEUR et al
EPI_ISL_671921	2020-10-12	National Virus Reference Laboratory	National Virus Reference Laboratory	Michael Carr et al
EPI_ISL_637019	2020-10-12	Department of Infectious Diseases and Immunology, National Hospital Organization Nagoya Medical Center	Clinical Research Center, National Hospital Organization Nagoya Medical Center	Yoshihiro Nakata et al
EPI_ISL_729985	2020-10-12	Nigeria Centre for Disease Control (NCDC)	African Centre of Excellence for Genomics of Infectious Diseases (ACEGID), Redeemer's University, Ede, Osun State, Nigeria	Oluniyi P.E. et al et al
EPI_ISL_605879	2020-10-13	PathWest Laboratory Medicine WA	PathWest Laboratory Medicine WA Microbial Surveillance Unit	PathWest Laboratory Medicine WA Microbial Surveillance Unit et al
EPI_ISL_706933	2020-10-13	Oxford Viromics, NDM, University of Oxford; Oxford University Hospitals; Basingstoke and North Hampshire Hospital	COVID-19 Genomics UK (COG-UK) Consortium	Tanya Golubchik et al
EPI_ISL_729883	2020-10-13	Laboratorio de Referencia Nacional de Virus Respiratorios, Instituto Nacional de Salud Peru	Laboratorio de Genómica Microbiana, Universidad Peruana Cayetano Heredia	Pablo Tsukayama et al
EPI_ISL_729884	2020-10-13	Laboratorio de Referencia Nacional de Virus Respiratorios, Instituto Nacional de Salud Peru	Laboratorio de Genómica Microbiana, Universidad Peruana Cayetano Heredia	Pablo Tsukayama et al
EPI_ISL_660155	2020-10-13	PathCare	National Health Laboratory Service (NHLS), Tygerberg	Susan Engelbrecht et al
EPI_ISL_632272	2020-10-14	Communicable Disease Laboratory, Public Health Directorate	Communicable Disease Laboratory, Public Health Directorate	AlWasti et al
EPI_ISL_632255	2020-10-14	Communicable Disease Laboratory, Public Health Directorate	Communicable Disease Laboratory, Public Health Directorate	AlWasti et al
EPI_ISL_632253	2020-10-14	Communicable Disease Laboratory, Public Health Directorate	Communicable Disease Laboratory, Public Health Directorate	AlWasti et al
EPI_ISL_632903	2020-10-14	Communicable Disease Laboratory, Public Health Directorate	Communicable Disease Laboratory, Public Health Directorate	AlAbbas et al
EPI_ISL_632901	2020-10-14	Communicable Disease Laboratory, Public Health Directorate	Communicable Disease Laboratory, Public Health Directorate	AlAbbas et al
EPI_ISL_671903	2020-10-14	National Virus Reference Laboratory	National Virus Reference Laboratory	Michael Carr et al
EPI_ISL_632683	2020-10-14	Dutch COVID-19 response team	Erasmus Medical Center	Bas Oude Munnink et al
EPI_ISL_729873	2020-10-14	Laboratorio de Referencia Nacional de Virus Respiratorios, Instituto Nacional de Salud Peru	Laboratorio de Genómica Microbiana, Universidad Peruana Cayetano Heredia	Pablo Tsukayama et al
EPI_ISL_729881	2020-10-14	Laboratorio de Referencia Nacional de Virus Respiratorios, Instituto Nacional de Salud Peru	Laboratorio de Genómica Microbiana, Universidad Peruana Cayetano Heredia	Pablo Tsukayama et al
EPI_ISL_729889	2020-10-14	Laboratorio de Referencia Nacional de Virus Respiratorios, Instituto Nacional de Salud Peru	Laboratorio de Genómica Microbiana, Universidad Peruana Cayetano Heredia	Pablo Tsukayama et al
EPI_ISL_729900	2020-10-14	Laboratorio de Referencia Nacional de Virus Respiratorios, Instituto Nacional de Salud Peru	Laboratorio de Genómica Microbiana, Universidad Peruana Cayetano Heredia	Pablo Tsukayama et al

EPI_ISL_729901	2020-10-14	Laboratorio de Referencia Nacional de Virus Respiratorios, Instituto Nacional de Salud Peru	Laboratorio de Genómica Microbiana, Universidad Peruana Cayetano Heredia	Pablo Tsukayama et al
EPI_ISL_691686	2020-10-14	Hospital Universitario de Ceuta	Instituto de Salud Carlos III	Iglesias-Caballero et al
EPI_ISL_691689	2020-10-14	Hospital Universitario de Ceuta	Instituto de Salud Carlos III	Iglesias-Caballero et al
EPI_ISL_691693	2020-10-14	Hospital Universitario de Ceuta	Instituto de Salud Carlos III	Iglesias-Caballero et al
EPI_ISL_691697	2020-10-14	Hospital Universitario de Ceuta	Instituto de Salud Carlos III	Iglesias-Caballero et al
EPI_ISL_603675	2020-10-14	Viollier AG	Department of Biosystems Science and Engineering, ETH Zürich	Christian Beisel et al
EPI_ISL_710534	2020-10-14	Hôpital Fattouma-Bourguiba de Monastir	Laboratoire des Procédés de Criblage Moléculaire et Cellulaire-Centre de Biotechnologie de Sfax	Souissi et al
EPI_ISL_707696	2020-10-15	Medical Research Center, Faculty of Medicine, Syarif Hidayatullah State Islamic University Jakarta	Medical Research Center, Faculty of Medicine, Syarif Hidayatullah State Islamic University Jakarta	Laifa Hendarmin et al
EPI_ISL_632689	2020-10-15	Dutch COVID-19 response team	Erasmus Medical Center	Bas Oude Munnink et al
EPI_ISL_709542	2020-10-15	National Institute of Blood Diseases (NIBD), Molecular Biology Lab	Genomics Lab NIBD	Samina Naz Mukry et al
EPI_ISL_729867	2020-10-15	Laboratorio de Referencia Nacional de Virus Respiratorios, Instituto Nacional de Salud Peru	Laboratorio de Genómica Microbiana, Universidad Peruana Cayetano Heredia	Pablo Tsukayama et al
EPI_ISL_729868	2020-10-15	Laboratorio de Referencia Nacional de Virus Respiratorios, Instituto Nacional de Salud Peru	Laboratorio de Genómica Microbiana, Universidad Peruana Cayetano Heredia	Pablo Tsukayama et al
EPI_ISL_691615	2020-10-15	Servicio de Microbiología, Hospital Universitario Son Espases	SeqCOVID-SPAIN consortium/IBV(CSIC)	Carla López-Causapé et al
EPI_ISL_729361	2020-10-16	A. Krumbholz, Labor Dr. Krause und Kollegen MVZ GmbH, Kiel	Charité Universitätsmedizin Berlin, Institut für Virologie	Victor M Corman et al
EPI_ISL_729363	2020-10-16	A. Krumbholz, Labor Dr. Krause und Kollegen MVZ GmbH, Kiel	Charité Universitätsmedizin Berlin, Institut für Virologie	Victor M Corman et al
EPI_ISL_718308	2020-10-16	Institute for Medical Research, Infectious Disease Research Centre, National Institutes of Health, Ministry of Health Malaysia	Institute for Medical Research, Infectious Disease Research Centre, National Institutes of Health, Ministry of Health Malaysia	Suppiah J et al
EPI_ISL_718307	2020-10-16	Institute for Medical Research, Infectious Disease Research Centre, National Institutes of Health, Ministry of Health Malaysia	Institute for Medical Research, Infectious Disease Research Centre, National Institutes of Health, Ministry of Health Malaysia	Suppiah J et al
EPI_ISL_729897	2020-10-16	Laboratorio de Referencia Nacional de Virus Respiratorios, Instituto Nacional de Salud Peru	Laboratorio de Genómica Microbiana, Universidad Peruana Cayetano Heredia	Pablo Tsukayama et al
EPI_ISL_596491	2020-10-16	National Public Health Laboratory, National Centre for Infectious Diseases	National Public Health Laboratory, National Centre for Infectious Diseases	Tze Minn Mak et al
EPI_ISL_660153	2020-10-16	PathCare	National Health Laboratory Service (NHLS), Tygerberg	Susan Engelbrecht et al
EPI_ISL_710540	2020-10-16	Hôpital Fattouma-Bourguiba de Monastir	Laboratoire des Procédés de Criblage Moléculaire et Cellulaire-Centre de Biotechnologie de Sfax	Souissi et al
EPI_ISL_710541	2020-10-16	Hôpital Fattouma-Bourguiba de Monastir	Laboratoire des Procédés de Criblage Moléculaire et Cellulaire-Centre de Biotechnologie de Sfax	Souissi et al
EPI_ISL_718309	2020-10-17	Institute for Medical Research, Infectious Disease Research Centre, National Institutes of Health, Ministry of Health Malaysia	Institute for Medical Research, Infectious Disease Research Centre, National Institutes of Health, Ministry of Health Malaysia	Suppiah J et al
EPI_ISL_708652	2020-10-18	Medical Research Center, Faculty of Medicine, Syarif Hidayatullah State Islamic University Jakarta	Medical Research Center, Faculty of Medicine, Syarif Hidayatullah State Islamic University Jakarta	Chris Adhiyanto et al
EPI_ISL_660489	2020-10-19	Laboratoire de Microbiologie CHU Souro Sanou	Centre Muraz	Abdoul-Salam Ouedraogo et al
EPI_ISL_660497	2020-10-19	Laboratoire de Microbiologie CHU Souro Sanou	Centre Muraz	Abdoul-Salam Ouedraogo et al
EPI_ISL_618596	2020-10-19	Department of Virus and Microbiological Special Diagnostics, Statens Serum Institut, Denmark	Albertsen lab, Department of Chemistry and Bioscience, Aalborg University, Denmark	Danish Covid-19 Genome Consortia et al
EPI_ISL_622789	2020-10-19	Canterbury Health Laboratories	Institute of Environmental Science and Research (ESR)	Xiaoyun Ren et al
EPI_ISL_668401	2020-10-19	Oslo University Hospital, Department of Medical Microbiology	Norwegian Institute of Public Health, Department of Virology	Kathrine Stene-Johansen et al
EPI_ISL_707789	2020-10-19	Rwanda National Reference Laboratory	Rwanda National Reference Laboratory	Enatha Mukantwari et al
EPI_ISL_648176	2020-10-19	The Public Health Agency of Sweden	The Public Health Agency of Sweden	Anna-Malin Linde et al
EPI_ISL_648179	2020-10-19	The Public Health Agency of Sweden	The Public Health Agency of Sweden	Anna-Malin Linde et al
EPI_ISL_712060	2020-10-19	Hôpital Fattouma-Bourguiba de Monastir	Laboratoire des Procédés de Criblage Moléculaire et Cellulaire-Centre de Biotechnologie de Sfax	Souissi et al

EPI_ISL_708806	2020-10-20	Regional medical sciences center 6 chonburi	National Institute of Health, Department of Medical Sciences, Ministry of Public Health, Thailand	Pilailuk Okada et al
EPI_ISL_707890	2020-10-22	Medical Research Center, Faculty of Medicine, Syarif Hidayatullah State Islamic University Jakarta	Medical Research Center, Faculty of Medicine, Syarif Hidayatullah State Islamic University Jakarta	Zeti Harriyati et al
EPI_ISL_708044	2020-10-22	Dept. of Medical Microbiology, Stavanger University Hospital, Helse Stavanger HF	Norwegian Institute of Public Health, Department of Virology	Kathrine Stene-Johansen et al
EPI_ISL_602576	2020-10-22	Centre for Dengue Research, Department of Immunology and Molecular Medicine	Centre for Dengue Research, Department of Immunology and Molecular Medicine	Chandima Jeewandara et al
EPI_ISL_602566	2020-10-22	Centre for Dengue Research, Department of Immunology and Molecular Medicine	Centre for Dengue Research, Department of Immunology and Molecular Medicine	Chandima Jeewandara et al
EPI_ISL_672530	2020-10-22	Madera County Department of Public Health	Chan-Zuckerberg Biohub	CZB Cliahub Consortium et al
EPI_ISL_626320	2020-10-23	Department of Clinical Microbiology	GIGA Medical Genomics	Keith Durkin et al
EPI_ISL_637020	2020-10-23	Department of Infectious Diseases and Immunology, National Hospital Organization Nagoya Medical Center	Clinical Research Center, National Hospital Organization Nagoya Medical Center	Yoshihiro Nakata et al
EPI_ISL_602574	2020-10-23	Centre for Dengue Research, Department of Immunology and Molecular Medicine	Centre for Dengue Research, Department of Immunology and Molecular Medicine	Chandima Jeewandara et al
EPI_ISL_653802	2020-10-24	I.R.C.C.S. "S. De Bellis" - Ente Ospedaliero	Istituto Zooprofilattico Sperimentale della Puglia e della Basilicata	Parisi A. et al
EPI_ISL_653803	2020-10-24	I.R.C.C.S. "S. De Bellis" - Ente Ospedaliero	Istituto Zooprofilattico Sperimentale della Puglia e della Basilicata	Parisi A. et al
EPI_ISL_605817	2020-10-24	National Public Health Laboratory, National Centre for Infectious Diseases	National Public Health Laboratory, National Centre for Infectious Diseases	Tze Minn Mak et al
EPI_ISL_717991	2020-10-26	Lab voor klinische biologie	Onderzoeksgroep Virologie	Laurens Lambrechts et al
EPI_ISL_636631	2020-10-26	Department of Clinical Microbiology	GIGA Medical Genomics	Keith Durkin et al
EPI_ISL_717921	2020-10-26	Laboratorio de Virologia Molecular / UFRJ	Bioinformatics Laboratory / LNCC	Carolina M Voloch et al
EPI_ISL_717926	2020-10-26	Laboratorio de Virologia Molecular / UFRJ	Bioinformatics Laboratory / LNCC	Carolina M Voloch et al
EPI_ISL_671067	2020-10-26	Department of Virus and Microbiological Special Diagnostics, Statens Serum Institut, Copenhagen, Denmark	Albertsen Lab, Department of Chemistry and Bioscience, Aalborg University, Denmark	Danish Covid-19 Genome Consortium et al
EPI_ISL_722906	2020-10-26	Istituto Zooprofilattico Sperimentale della Puglia e della Basilicata	Istituto Zooprofilattico Sperimentale della Puglia e della Basilicata	Parisi A. et al
EPI_ISL_635075	2020-10-26	Norwegian Institute of Public Health, Department of Virology	Norwegian Institute of Public Health, Department of Virology	Kathrine Stene-Johansen et al
EPI_ISL_677323	2020-10-26	University of Wisconsin-Madison AIDS Vaccine Research Laboratories	University of Wisconsin-Madison AIDS Vaccine Research Laboratories	Gage Moreno et al
EPI_ISL_717927	2020-10-27	Laboratorio de Virologia Molecular / UFRJ	Bioinformatics Laboratory / LNCC	Carolina M Voloch et al
EPI_ISL_717928	2020-10-27	Laboratorio de Virologia Molecular / UFRJ	Bioinformatics Laboratory / LNCC	Carolina M Voloch et al
EPI_ISL_717929	2020-10-27	Laboratorio de Virologia Molecular / UFRJ	Bioinformatics Laboratory / LNCC	Carolina M Voloch et al
EPI_ISL_717930	2020-10-27	Laboratorio de Virologia Molecular / UFRJ	Bioinformatics Laboratory / LNCC	Carolina M Voloch et al
EPI_ISL_717931	2020-10-27	Laboratorio de Virologia Molecular / UFRJ	Bioinformatics Laboratory / LNCC	Carolina M Voloch et al
EPI_ISL_660472	2020-10-27	Laboratoire de Microbiologie CHU Souro Sanou	Centre Muraz	Abdoul-Salam Ouedraogo et al
EPI_ISL_654503	2020-10-27	The Public Health Agency of Sweden	The Public Health Agency of Sweden	Anna-Malin Linde et al
EPI_ISL_677325	2020-10-27	University of Wisconsin-Madison AIDS Vaccine Research Laboratories	University of Wisconsin-Madison AIDS Vaccine Research Laboratories	Gage Moreno et al
EPI_ISL_637021	2020-10-28	Department of Infectious Diseases and Immunology, National Hospital Organization Nagoya Medical Center	Clinical Research Center, National Hospital Organization Nagoya Medical Center	Yoshihiro Nakata et al
EPI_ISL_666885	2020-10-29	Department of Clinical Microbiology	GIGA Medical Genomics	Keith Durkin et al
EPI_ISL_717922	2020-10-29	Laboratorio de Virologia Molecular / UFRJ	Bioinformatics Laboratory / LNCC	Carolina M Voloch et al
EPI_ISL_717924	2020-10-29	Laboratorio de Virologia Molecular / UFRJ	Bioinformatics Laboratory / LNCC	Carolina M Voloch et al
EPI_ISL_717932	2020-10-29	Laboratorio de Virologia Molecular / UFRJ	Bioinformatics Laboratory / LNCC	Carolina M Voloch et al
EPI_ISL_717933	2020-10-29	Laboratorio de Virologia Molecular / UFRJ	Bioinformatics Laboratory / LNCC	Carolina M Voloch et al
EPI_ISL_717934	2020-10-29	Laboratorio de Virologia Molecular / UFRJ	Bioinformatics Laboratory / LNCC	Carolina M Voloch et al

EPI_ISL_717935	2020-10-29	Laboratorio de Virologia Molecular / UFRJ	Bioinformatics Laboratory / LNCC	Carolina M Voloch et al
EPI_ISL_717936	2020-10-29	Laboratorio de Virologia Molecular / UFRJ	Bioinformatics Laboratory / LNCC	Carolina M Voloch et al
EPI_ISL_717937	2020-10-29	Laboratorio de Virologia Molecular / UFRJ	Bioinformatics Laboratory / LNCC	Carolina M Voloch et al
EPI_ISL_717938	2020-10-29	Laboratorio de Virologia Molecular / UFRJ	Bioinformatics Laboratory / LNCC	Carolina M Voloch et al
EPI_ISL_717939	2020-10-29	Laboratorio de Virologia Molecular / UFRJ	Bioinformatics Laboratory / LNCC	Carolina M Voloch et al
EPI_ISL_693681	2020-10-29	The National Institute of Public Health	State Veterinary Institute Prague	Nagy et al
EPI_ISL_639989	2020-10-29	CNR Virus des Infections Respiratoires - France SUD	CNR Virus des Infections Respiratoires - France SUD	Antonin Bal et al
EPI_ISL_661304	2020-10-29	CSIR-Indian Institute of Chemical Biology, MEDICA Supercpecialty Hospital Kolkata	CSIR-Indian Institute of Chemical Biology, MEDICA Supercpecialty Hospital Kolkata	Sujay Krishna Maity et al
EPI_ISL_661305	2020-10-29	CSIR-Indian Institute of Chemical Biology, MEDICA Supercpecialty Hospital Kolkata	CSIR-Indian Institute of Chemical Biology, MEDICA Supercpecialty Hospital Kolkata	Sujay Krishna Maity et al
EPI_ISL_661307	2020-10-29	CSIR-Indian Institute of Chemical Biology, MEDICA Supercpecialty Hospital Kolkata	CSIR-Indian Institute of Chemical Biology, MEDICA Supercpecialty Hospital Kolkata	Sujay Krishna Maity et al
EPI_ISL_661309	2020-10-29	CSIR-Indian Institute of Chemical Biology, MEDICA Supercpecialty Hospital Kolkata	CSIR-Indian Institute of Chemical Biology, MEDICA Supercpecialty Hospital Kolkata	Sujay Krishna Maity et al
EPI_ISL_661311	2020-10-29	CSIR-Indian Institute of Chemical Biology, MEDICA Supercpecialty Hospital Kolkata	CSIR-Indian Institute of Chemical Biology, MEDICA Supercpecialty Hospital Kolkata	Sujay Krishna Maity et al
EPI_ISL_722874	2020-10-29	Istituto Zooprofilattico Sperimentale della Puglia e della Basilicata	Istituto Zooprofilattico Sperimentale della Puglia e della Basilicata	Parisi A. et al
EPI_ISL_622787	2020-10-29	Middlemore Hospital	Institute of Environmental Science and Research (ESR)	Xiaoyun Ren et al
EPI_ISL_717940	2020-10-30	Laboratorio de Virologia Molecular / UFRJ	Bioinformatics Laboratory / LNCC	Carolina M Voloch et al
EPI_ISL_717941	2020-10-30	Laboratorio de Virologia Molecular / UFRJ	Bioinformatics Laboratory / LNCC	Carolina M Voloch et al
EPI_ISL_729426	2020-10-30	A. Krumbholz, Labor Dr. Krause und Kollegen MVZ GmbH, Kiel	Charité Universitätsmedizin Berlin, Institut für Virologie	Victor M Corman et al
EPI_ISL_653782	2020-10-30	I.R.C.C.S. "S. De Bellis" - Ente Ospedaliero	Istituto Zooprofilattico Sperimentale della Puglia e della Basilicata	Parisi A. et al
EPI_ISL_728167	2020-10-30	Institute for Medical Research, Infectious Disease Research Centre, National Institutes of Health, Ministry of Health Malaysia	Institute for Medical Research, Infectious Disease Research Centre, National Institutes of Health, Ministry of Health Malaysia	Suppiah J et al
EPI_ISL_632769	2020-10-30	Dutch COVID-19 response team	Erasmus Medical Center	Bas Oude Munnink et al
EPI_ISL_626353	2020-10-30	LabPLUS	Institute of Environmental Science and Research (ESR)	Xiaoyun Ren et al
EPI_ISL_626354	2020-10-30	LabPLUS	Institute of Environmental Science and Research (ESR)	Xiaoyun Ren et al
EPI_ISL_677330	2020-10-30	University of Wisconsin-Madison AIDS Vaccine Research Laboratories	University of Wisconsin-Madison AIDS Vaccine Research Laboratories	Gage Moreno et al
EPI_ISL_677331	2020-10-30	University of Wisconsin-Madison AIDS Vaccine Research Laboratories	University of Wisconsin-Madison AIDS Vaccine Research Laboratories	Gage Moreno et al
EPI_ISL_722396	2020-10-31	Dutch COVID-19 response team	Erasmus Medical Center	Bas Oude Munnink et al
EPI_ISL_626643	2020-10-31	National Public Health Laboratory, National Centre for Infectious Diseases	National Public Health Laboratory, National Centre for Infectious Diseases	Tze Minn Mak et al
EPI_ISL_708823	2020-10-31	Vibharam Hospital	National Institute of Health, Department of Medical Sciences, Ministry of Public Health, Thailand	Pilailuk Okada et al
EPI_ISL_632310	2020-10-31	1-Laboratory of Microbiology, National Reference Lab, Charles Nicolle Hospital; 2-University of Tunis ElManar, Faculty of Medicine of Tunis, LR99ES09, Tunis, Tunisia	1-Clinical and Experimental Pharmacology Lab, LR16SP02, National Center of Pharmacovigilance, University of Tunis El Manar, Tunis, Tunisia. 2-Neurodegenerative diseases and psychiatric troubles, LR18SP03, Razi Hospital, University of Tunis El Manar, Tunis, Tunisia. 3- Ministry of Health, National Observatory of New and Emerging Diseases, 1006, Tunis, Tunisia	Ilhem Boutiba-Ben Boubaker et al
EPI_ISL_671866	2020-11-01	National Virus Reference Laboratory	National Virus Reference Laboratory	Michael Carr et al
EPI_ISL_718314	2020-11-01	Institute for Medical Research, Infectious Disease Research Centre, National Institutes of Health, Ministry of Health Malaysia	Institute for Medical Research, Infectious Disease Research Centre, National Institutes of Health, Ministry of Health Malaysia	Suppiah J et al
EPI_ISL_722405	2020-11-01	Dutch COVID-19 response team	Erasmus Medical Center	Bas Oude Munnink et al
EPI_ISL_632406	2020-11-01	Dutch COVID-19 response team	Erasmus Medical Center	Bas Oude Munnink et al

EPI_ISL_622824	2020-11-01	Canterbury Health Laboratories	Institute of Environmental Science and Research (ESR)	Xiaoyun Ren et al
EPI_ISL_669558	2020-11-02	Department of Virus and Microbiological Special Diagnostics, Statens Serum Institut, Copenhagen, Denmark	Albertsen Lab, Department of Chemistry and Bioscience, Aalborg University, Denmark	Danish Covid-19 Genome Consortium et al
EPI_ISL_669487	2020-11-02	Department of Virus and Microbiological Special Diagnostics, Statens Serum Institut, Copenhagen, Denmark	Albertsen Lab, Department of Chemistry and Bioscience, Aalborg University, Denmark	Danish Covid-19 Genome Consortium et al
EPI_ISL_669778	2020-11-02	Department of Virus and Microbiological Special Diagnostics, Statens Serum Institut, Copenhagen, Denmark	Albertsen Lab, Department of Chemistry and Bioscience, Aalborg University, Denmark	Danish Covid-19 Genome Consortium et al
EPI_ISL_670521	2020-11-02	Department of Virus and Microbiological Special Diagnostics, Statens Serum Institut, Copenhagen, Denmark	Albertsen Lab, Department of Chemistry and Bioscience, Aalborg University, Denmark	Danish Covid-19 Genome Consortium et al
EPI_ISL_729427	2020-11-02	A. Krumbholz, Labor Dr. Krause und Kollegen MVZ GmbH, Kiel	Charité Universitätsmedizin Berlin, Institut für Virologie	Victor M Corman et al
EPI_ISL_729349	2020-11-02	A. Krumbholz, Labor Dr. Krause und Kollegen MVZ GmbH, Kiel	Charité Universitätsmedizin Berlin, Institut für Virologie	Victor M Corman et al
EPI_ISL_728170	2020-11-02	Institute for Medical Research, Infectious Disease Research Centre, National Institutes of Health, Ministry of Health Malaysia	Institute for Medical Research, Infectious Disease Research Centre, National Institutes of Health, Ministry of Health Malaysia	Suppiah J et al
EPI_ISL_658863	2020-11-02	Instituto de Diagnostico y Referencia Epidemiologicos (INDRE)	Instituto de Diagnostico y Referencia Epidemiologicos (INDRE)	Gisela Barrera-Badillo et al
EPI_ISL_658868	2020-11-02	Instituto de Diagnostico y Referencia Epidemiologicos (INDRE)	Instituto de Diagnostico y Referencia Epidemiologicos (INDRE)	Gisela Barrera-Badillo et al
EPI_ISL_658873	2020-11-02	Instituto de Diagnostico y Referencia Epidemiologicos (INDRE)	Instituto de Diagnostico y Referencia Epidemiologicos (INDRE)	Gisela Barrera-Badillo et al
EPI_ISL_708064	2020-11-02	Unilabs Laboratory Medicine	Norwegian Institute of Public Health, Department of Virology	Kathrine Stene-Johansen et al
EPI_ISL_660653	2020-11-02	NHLS-IALCH	KRISP, KZN Research Innovation and Sequencing Platform	Giandhari J et al
EPI_ISL_668455	2020-11-02	Centre for Dengue Research, Department of Immunology and Molecular Medicine	Centre for Dengue Research, Department of Immunology and Molecular Medicine	Chandima Jeewandara et al
EPI_ISL_635059	2020-11-02	1-Laboratory of Microbiology, National Reference Lab, Charles Nicolle Hospital; 2-University of Tunis ElManar, Faculty of Medicine of Tunis, LR99ES09, Tunis, Tunisia	1-Clinical and Experimental Pharmacology Lab, LR16SP02, National Center of Pharmacovigilance, University of Tunis El Manar, Tunis, Tunisia. 2-Neurodegenerative diseases and psychiatric troubles, LR18SP03, Razi Hospital, University of Tunis El Manar, Tunis, Tunisia. 3- Ministry of Health, National Observatory of New and Emerging Diseases, 1006, Tunis, Tunisia	Ilhem Boutiba-Ben Boubaker et al
EPI_ISL_717808	2020-11-03	Laboratorio de Virologia Molecular / UFRJ	Bioinformatics Laboratory / LNCC	Carolina M Voloch et al
EPI_ISL_717942	2020-11-03	Laboratorio de Virologia Molecular / UFRJ	Bioinformatics Laboratory / LNCC	Carolina M Voloch et al
EPI_ISL_717943	2020-11-03	Laboratorio de Virologia Molecular / UFRJ	Bioinformatics Laboratory / LNCC	Carolina M Voloch et al
EPI_ISL_717944	2020-11-03	Laboratorio de Virologia Molecular / UFRJ	Bioinformatics Laboratory / LNCC	Carolina M Voloch et al
EPI_ISL_717945	2020-11-03	Laboratorio de Virologia Molecular / UFRJ	Bioinformatics Laboratory / LNCC	Carolina M Voloch et al
EPI_ISL_717946	2020-11-03	Laboratorio de Virologia Molecular / UFRJ	Bioinformatics Laboratory / LNCC	Carolina M Voloch et al
EPI_ISL_717947	2020-11-03	Laboratorio de Virologia Molecular / UFRJ	Bioinformatics Laboratory / LNCC	Carolina M Voloch et al
EPI_ISL_717948	2020-11-03	Laboratorio de Virologia Molecular / UFRJ	Bioinformatics Laboratory / LNCC	Carolina M Voloch et al
EPI_ISL_717949	2020-11-03	Laboratorio de Virologia Molecular / UFRJ	Bioinformatics Laboratory / LNCC	Carolina M Voloch et al
EPI_ISL_717950	2020-11-03	Laboratorio de Virologia Molecular / UFRJ	Bioinformatics Laboratory / LNCC	Carolina M Voloch et al
EPI_ISL_660473	2020-11-03	Laboratoire de Microbiologie CHU Souro Sanou	Centre Muraz	Abdoul-Salam Ouedraogo et al
EPI_ISL_660519	2020-11-03	Laboratoire de Microbiologie CHU Souro Sanou	Centre Muraz	Abdoul-Salam Ouedraogo et al
EPI_ISL_693682	2020-11-03	The National Institute of Public Health	State Veterinary Institute Prague	Nagy et al
EPI_ISL_693678	2020-11-03	The National Institute of Public Health	State Veterinary Institute Prague	Nagy et al
EPI_ISL_693666	2020-11-03	The National Institute of Public Health	State Veterinary Institute Prague	Nagy et al
EPI_ISL_693669	2020-11-03	The National Institute of Public Health	State Veterinary Institute Prague	Nagy et al
EPI_ISL_693675	2020-11-03	The National Institute of Public Health	State Veterinary Institute Prague	Nagy et al
EPI_ISL_659855	2020-11-03	Lighthouse Lab in Glasgow	Wellcome Sanger Institute for the COVID-19 Genomics UK (COG-UK) Consortium	Harper VanSteenhouse et al
EPI_ISL_729429	2020-11-03	A. Krumbholz, Labor Dr. Krause und Kollegen MVZ GmbH, Kiel	Charité Universitätsmedizin Berlin, Institut für Virologie	Victor M Corman et al

EPI_ISL_653789	2020-11-03	I.R.C.C.S. "S. De Bellis" - Ente Ospedaliero	Istituto Zooprofilattico Sperimentale della Puglia e della Basilicata	Parisi A. et al
EPI_ISL_653800	2020-11-03	I.R.C.C.S. "S. De Bellis" - Ente Ospedaliero	Istituto Zooprofilattico Sperimentale della Puglia e della Basilicata	Parisi A. et al
EPI_ISL_653805	2020-11-03	I.R.C.C.S. "S. De Bellis" - Ente Ospedaliero	Istituto Zooprofilattico Sperimentale della Puglia e della Basilicata	Parisi A. et al
EPI_ISL_658886	2020-11-03	Instituto de Diagnostico y Referencia Epidemiologicos (INDRE)	Instituto de Diagnostico y Referencia Epidemiologicos (INDRE)	Ernesto Ramirez-Gonzalez et al
EPI_ISL_658888	2020-11-03	Instituto de Diagnostico y Referencia Epidemiologicos (INDRE)	Instituto de Diagnostico y Referencia Epidemiologicos (INDRE)	Ernesto Ramirez-Gonzalez et al
EPI_ISL_708040	2020-11-03	Norwegian Institute of Public Health, Department of Virology	Norwegian Institute of Public Health, Department of Virology	Kathrine Stene-Johansen et al
EPI_ISL_645118	2020-11-03	National Public Health Laboratory, National Centre for Infectious Diseases	National Public Health Laboratory, National Centre for Infectious Diseases	Tze Minn Mak et al
EPI_ISL_660656	2020-11-03	NHLS-IALCH	KRISP, KZN Research Innovation and Sequencing Platform	Giandhari J et al
EPI_ISL_668448	2020-11-03	Centre for Dengue Research, Department of Immunology and Molecular Medicine	Centre for Dengue Research, Department of Immunology and Molecular Medicine	Chandima Jeewandara et al
EPI_ISL_668453	2020-11-03	Centre for Dengue Research, Department of Immunology and Molecular Medicine	Centre for Dengue Research, Department of Immunology and Molecular Medicine	Chandima Jeewandara et al
EPI_ISL_717951	2020-11-04	Laboratorio de Virologia Molecular / UFRJ	Bioinformatics Laboratory / LNCC	Carolina M Voloch et al
EPI_ISL_717952	2020-11-04	Laboratorio de Virologia Molecular / UFRJ	Bioinformatics Laboratory / LNCC	Carolina M Voloch et al
EPI_ISL_717953	2020-11-04	Laboratorio de Virologia Molecular / UFRJ	Bioinformatics Laboratory / LNCC	Carolina M Voloch et al
EPI_ISL_717954	2020-11-04	Laboratorio de Virologia Molecular / UFRJ	Bioinformatics Laboratory / LNCC	Carolina M Voloch et al
EPI_ISL_660452	2020-11-04	Laboratoire de Microbiologie CHU Souro Sanou	Centre Muraz	Abdoul-Salam Ouedraogo et al
EPI_ISL_718240	2020-11-04	Hospital	National Reference Center for Viruses of Respiratory Infections, Institut Pasteur, Paris	Marion Barbet et al
EPI_ISL_729629	2020-11-04	A. Krumbholz, Labor Dr. Krause und Kollegen MVZ GmbH, Kiel	Charité Universitätsmedizin Berlin, Institut für Virologie	Victor M Corman et al
EPI_ISL_710546	2020-11-04	National Institute for Infectious Diseases, INMI, "L. Spallanzani" IRCCS	National Institute for Infectious Diseases, INMI, "L. Spallanzani" IRCCS	E Giombini et al
EPI_ISL_708059	2020-11-04	Furst Medical Laboratory	Norwegian Institute of Public Health, Department of Virology	Kathrine Stene-Johansen et al
EPI_ISL_645117	2020-11-04	National Public Health Laboratory, National Centre for Infectious Diseases	National Public Health Laboratory, National Centre for Infectious Diseases	Tze Minn Mak et al
EPI_ISL_660647	2020-11-04	NHLS-IALCH	KRISP, KZN Research Innovation and Sequencing Platform	Giandhari J et al
EPI_ISL_668449	2020-11-04	Centre for Dengue Research, Department of Immunology and Molecular Medicine	Centre for Dengue Research, Department of Immunology and Molecular Medicine	Chandima Jeewandara et al
EPI_ISL_676489	2020-11-04	Klinisk mikrobiologi	The Public Health Agency of Sweden	Department of Microbiology et al
EPI_ISL_693837	2020-11-04	Viollier AG	Department of Biosystems Science and Engineering, ETH Zürich	Christian Beisel et al
EPI_ISL_717830	2020-11-05	Laboratorio de Virologia Molecular / UFRJ	Bioinformatics Laboratory / LNCC	Carolina M Voloch et al
EPI_ISL_717898	2020-11-05	Laboratorio de Virologia Molecular / UFRJ	Bioinformatics Laboratory / LNCC	Carolina M Voloch et al
EPI_ISL_717923	2020-11-05	Laboratorio de Virologia Molecular / UFRJ	Bioinformatics Laboratory / LNCC	Carolina M Voloch et al
EPI_ISL_717955	2020-11-05	Laboratorio de Virologia Molecular / UFRJ	Bioinformatics Laboratory / LNCC	Carolina M Voloch et al
EPI_ISL_717956	2020-11-05	Laboratorio de Virologia Molecular / UFRJ	Bioinformatics Laboratory / LNCC	Carolina M Voloch et al
EPI_ISL_717957	2020-11-05	Laboratorio de Virologia Molecular / UFRJ	Bioinformatics Laboratory / LNCC	Carolina M Voloch et al
EPI_ISL_722894	2020-11-05	Istituto Zooprofilattico Sperimentale della Puglia e della Basilicata	Istituto Zooprofilattico Sperimentale della Puglia e della Basilicata	Parisi A. et al
EPI_ISL_708106	2020-11-05	Department of Medical Microbiology, St. Olavs hospital	Norwegian Institute of Public Health, Department of Virology	Kathrine Stene-Johansen et al
EPI_ISL_677505	2020-11-05	University of Wisconsin-Madison AIDS Vaccine Research Laboratories	University of Wisconsin-Madison AIDS Vaccine Research Laboratories	Gage Moreno et al
EPI_ISL_717968	2020-11-06	Molecular diagnostic laboratory of Federal Budget Institution of Science "Central Research Institute of Epidemiology" of The Federal Service on Customers' Rights Protection and Human Well-being Surveillance	Group of Genomics and Postgenomic Technologies of Central Research Institute of Epidemilology	Samoilov AE et al

EPI_ISL_717970	2020-11-06	Molecular diagnostic laboratory of Federal Budget Institution of Science "Central Research Institute of Epidemiology" of The Federal Service on Customers' Rights Protection and Human Well-being Surveillance	Group of Genomics and Postgenomic Technologies of Central Research Institute of Epidemilology	Samoilov AE et al
EPI_ISL_717973	2020-11-06	Molecular diagnostic laboratory of Federal Budget Institution of Science "Central Research Institute of Epidemiology" of The Federal Service on Customers' Rights Protection and Human Well-being Surveillance	Group of Genomics and Postgenomic Technologies of Central Research Institute of Epidemilology	Samoilov AE et al
EPI_ISL_660619	2020-11-06	NHLS-IALCH	KRISP, KZN Research Innovation and Sequencing Platform	Giandhari J et al
EPI_ISL_637092	2020-11-07	LabPLUS	Institute of Environmental Science and Research (ESR)	Xiaoyun Ren et al
EPI_ISL_649153	2020-11-07	Al-Quds Nutrition and Health Research Institute, Al-Quds University	Al-Quds Nutrition and Health Research Institute, Al-Quds University	Nasereddin et al
EPI_ISL_661272	2020-11-07	Al-Quds Nutrition and Health Research Institute, Al-Quds University	Al-Quds Nutrition and Health Research Institute, Al-Quds University	Ereqat et al
EPI_ISL_717965	2020-11-07	Molecular diagnostic laboratory of Federal Budget Institution of Science "Central Research Institute of Epidemiology" of The Federal Service on Customers' Rights Protection and Human Well-being Surveillance	Group of Genomics and Postgenomic Technologies of Central Research Institute of Epidemilology	Samoilov AE et al
EPI_ISL_717966	2020-11-07	Molecular diagnostic laboratory of Federal Budget Institution of Science "Central Research Institute of Epidemiology" of The Federal Service on Customers' Rights Protection and Human Well-being Surveillance	Group of Genomics and Postgenomic Technologies of Central Research Institute of Epidemilology	Samoilov AE et al
EPI_ISL_712062	2020-11-08	Laboratoire de Microbiologie- CHU Habib Bourguiba – Sfax adresse	Laboratoire des Procédés de Criblage Moléculaire et Cellulaire-Centre de Biotechnologie de Sfax	Souissi et al
EPI_ISL_712064	2020-11-08	Laboratoire de Microbiologie- CHU Habib Bourguiba – Sfax adresse	Laboratoire des Procédés de Criblage Moléculaire et Cellulaire-Centre de Biotechnologie de Sfax	Souissi et al
EPI_ISL_699653	2020-11-09	South Eastern Area Laboratory Services (SEALS)	NSW Health Pathology - Institute of Clinical Pathology and Medical Research; Westmead Hospital; University of Sydney	CIDM-PH et al. et al
EPI_ISL_667804	2020-11-09	South Eastern Area Laboratory Services (SEALS)	NSW Health Pathology - Institute of Clinical Pathology and Medical Research; Westmead Hospital; University of Sydney	CIDM-PH et al. et al
EPI_ISL_668576	2020-11-09	Department of Virus and Microbiological Special Diagnostics, Statens Serum Institut, Copenhagen, Denmark	Albertsen Lab, Department of Chemistry and Bioscience, Aalborg University, Denmark	Danish Covid-19 Genome Consortium et al
EPI_ISL_655591	2020-11-09	Lighthouse Lab in Alderley Park	Wellcome Sanger Institute for the COVID-19 Genomics UK (COG-UK) Consortium	Jacquelyn Wynn et al
EPI_ISL_683621	2020-11-09	Servicio de Microbiología, Laboratori Clínic Metropolitana Nord. Hospital Universitari Germans Trias i Pujol. Institut d’Investigació en Ciències de la Salut Germans Trias i Pujol (IGTP)	SeqCOVID-SPAIN consortium/IBV(CSIC)	Elisa Martró et al
EPI_ISL_668452	2020-11-09	Centre for Dengue Research, Department of Immunology and Molecular Medicine	Centre for Dengue Research, Department of Immunology and Molecular Medicine	Chandima Jeewandara et al
EPI_ISL_729247	2020-11-09	Viollier AG	Department of Biosystems Science and Engineering, ETH Zürich	Chaoran Chen et al
EPI_ISL_703121	2020-11-10	Liverpool Clinical Laboratories	COVID-19 Genomics UK (COG-UK) Consortium	Sam Haldenby et al
EPI_ISL_708133	2020-11-10	Dept. of Medical Microbiology, Stavanger University Hospital, Helse Stavanger HF	Norwegian Institute of Public Health, Department of Virology	Kathrine Stene-Johansen et al
EPI_ISL_661276	2020-11-10	Klinisk mikrobiologi	The Public Health Agency of Sweden	Department of Microbiology et al
EPI_ISL_661277	2020-11-10	Klinisk mikrobiologi	The Public Health Agency of Sweden	Department of Microbiology et al
EPI_ISL_676505	2020-11-10	Klinisk mikrobiologi	The Public Health Agency of Sweden	Department of Microbiology et al
EPI_ISL_728960	2020-11-10	Viollier AG	Department of Biosystems Science and Engineering, ETH Zürich	Chaoran Chen et al
EPI_ISL_728244	2020-11-11	Institute for Medical Research, Infectious Disease Research Centre, National Institutes of Health, Ministry of Health Malaysia	Institute for Medical Research, Infectious Disease Research Centre, National Institutes of Health, Ministry of Health Malaysia	Suppiah J et al
EPI_ISL_722292	2020-11-11	Dutch COVID-19 response team	Erasmus Medical Center	Bas Oude Munnink et al
EPI_ISL_722242	2020-11-11	Servicio de Microbiología, Hospital Miguel Servet, Zaragoza	SeqCOVID-SPAIN consortium/IBV(CSIC)	Antonio Rezusta López et al

EPI_ISL_693302	2020-11-11	Department of Laboratory Medicine, National Taiwan University Hospital	Microbial Genomics Core Lab, National Taiwan University Centers of Genomic and Precision Medicine	Shiou-Hwei Yeh et al
EPI_ISL_708808	2020-11-11	Regional medical sciences center 6 chonburi	National Institute of Health, Department of Medical Sciences, Ministry of Public Health, Thailand	Pilailuk Okada et al
EPI_ISL_708810	2020-11-11	Regional medical sciences center 2 Phitsanulok	National Institute of Health, Department of Medical Sciences, Ministry of Public Health, Thailand	Pilailuk Okada et al
EPI_ISL_684035	2020-11-12	Communicable Disease Laboratory, Public Health Directorate	Communicable Disease Laboratory, Public Health Directorate	Alwasti et al
EPI_ISL_728247	2020-11-12	Institute for Medical Research, Infectious Disease Research Centre, National Institutes of Health, Ministry of Health Malaysia	Institute for Medical Research, Infectious Disease Research Centre, National Institutes of Health, Ministry of Health Malaysia	Suppiah J et al
EPI_ISL_718220	2020-11-12	Ministry of Health Hospitals	Institute of Health and Community Medicine	David Perera et al
EPI_ISL_718260	2020-11-12	Institute of Virology, Biomedical Research Center of the Slovak Academy of Sciences, Bratislava	Faculty of Natural Sciences, Comenius University, Bratislava	Viktória Hodorová et al
EPI_ISL_722271	2020-11-12	Servicio de Microbiología, Hospital Miguel Servet, Zaragoza	SeqCOVID-SPAIN consortium/IBV(CSIC)	Antonio Rezusta López et al
EPI_ISL_722272	2020-11-12	Servicio de Microbiología, Hospital Miguel Servet, Zaragoza	SeqCOVID-SPAIN consortium/IBV(CSIC)	Antonio Rezusta López et al
EPI_ISL_677211	2020-11-12	Virginia Division of Consolidated Laboratory Services	Virginia Division of Consolidated Laboratory Services	Virginia DCLS et al
EPI_ISL_677212	2020-11-12	Virginia Division of Consolidated Laboratory Services	Virginia Division of Consolidated Laboratory Services	Virginia DCLS et al
EPI_ISL_723494	2020-11-12	Virginia Division of Consolidated Laboratory Services (DCLS)	Virginia Division of Consolidated Laboratory Services (DCLS)	Virginia DCLS et al
EPI_ISL_672661	2020-11-13	South Eastern Area Laboratory Services (SEALS)	NSW Health Pathology - Institute of Clinical Pathology and Medical Research; Westmead Hospital; University of Sydney	CIDM-PH et al. et al
EPI_ISL_717573	2020-11-13	Lab voor klinische biologie	Onderzoeksgroep Virologie	Nick Vereecke et al
EPI_ISL_718259	2020-11-13	Institute of Virology, Biomedical Research Center of the Slovak Academy of Sciences, Bratislava	Faculty of Natural Sciences, Comenius University, Bratislava	Broňa Brejová et al
EPI_ISL_683969	2020-11-13	DOHMH Morrisania	New York City Public Health Laboratory	Jade Wang et al
EPI_ISL_654797	2020-11-14	SA Pathology	SA Pathology	Lex Leong et al
EPI_ISL_654798	2020-11-14	SA Pathology	SA Pathology	Lex Leong et al
EPI_ISL_707765	2020-11-14	Department of Clinical Microbiology	GIGA Medical Genomics	Keith Durkin et al
EPI_ISL_668335	2020-11-14	Lighthouse Lab in Glasgow	Wellcome Sanger Institute for the COVID-19 Genomics UK (COG-UK) Consortium	Harper VanSteenhouse et al
EPI_ISL_722390	2020-11-14	Dutch COVID-19 response team	Erasmus Medical Center	Bas Oude Munnink et al
EPI_ISL_671804	2020-11-14	Hospital Clínico Universitario Lozano Blesa de Zaragoza (España)	SeqCOVID-SPAIN consortium/IBV(CSIC)	Rafael Benito et al
EPI_ISL_728310	2020-11-15	CNR Virus des Infections Respiratoires - France SUD	CNR Virus des Infections Respiratoires - France SUD	Antonin Bal et al
EPI_ISL_682315	2020-11-16	Communicable Disease Laboratory, Public Health Directorate	Communicable Disease Laboratory, Public Health Directorate	Alwasti et al
EPI_ISL_682316	2020-11-16	Communicable Disease Laboratory, Public Health Directorate	Communicable Disease Laboratory, Public Health Directorate	Alwasti et al
EPI_ISL_684036	2020-11-16	Communicable Disease Laboratory, Public Health Directorate	Communicable Disease Laboratory, Public Health Directorate	Alwasti et al
EPI_ISL_661227	2020-11-16	Department of Clinical Microbiology	GIGA Medical Genomics	Keith Durkin et al
EPI_ISL_693972	2020-11-16	Viollier AG	Department of Biosystems Science and Engineering, ETH Zürich	Christian Beisel et al
EPI_ISL_693965	2020-11-16	Viollier AG	Department of Biosystems Science and Engineering, ETH Zürich	Christian Beisel et al
EPI_ISL_728516	2020-11-17	CNR Virus des Infections Respiratoires - France SUD	CNR Virus des Infections Respiratoires - France SUD	Antonin Bal et al
EPI_ISL_671883	2020-11-17	National Virus Reference Laboratory	National Virus Reference Laboratory	Michael Carr et al
EPI_ISL_722391	2020-11-17	Dutch COVID-19 response team	Erasmus Medical Center	Bas Oude Munnink et al
EPI_ISL_693303	2020-11-17	Department of Laboratory Medicine, National Taiwan University Hospital	Microbial Genomics Core Lab, National Taiwan University Centers of Genomic and Precision Medicine	Shiou-Hwei Yeh et al
EPI_ISL_721084	2020-11-18	Lighthouse Lab in Glasgow	Wellcome Sanger Institute for the COVID-19 Genomics UK (COG-UK) Consortium	Harper VanSteenhouse et al
EPI_ISL_718257	2020-11-19	Institute of Virology, Biomedical Research Center of the Slovak Academy of Sciences, Bratislava	Faculty of Natural Sciences, Comenius University, Bratislava	Broňa Brejová et al

EPI_ISL_718258	2020-11-19	Institute of Virology, Biomedical Research Center of the Slovak Academy of Sciences, Bratislava	Faculty of Natural Sciences, Comenius University, Bratislava	Viktória Hodorová et al
EPI_ISL_696480	2020-11-19	Crags Clinic wc CRG & NHLS/UCT	KRISP, KZN Research Innovation and Sequencing Platform	Arash Iranzadeh et al
EPI_ISL_722201	2020-11-20	Vitalis Mostar	Alea Genetic Center	Salihefendic L. et al
EPI_ISL_722209	2020-11-20	Dom Zdravlja Sarajevo	Alea Genetic Center	Salihefendic L. et al
EPI_ISL_728534	2020-11-20	CNR Virus des Infections Respiratoires - France SUD	CNR Virus des Infections Respiratoires - France SUD	Antonin Bal et al
EPI_ISL_661261	2020-11-20	LabPLUS	Institute of Environmental Science and Research (ESR)	Xiaoyun Ren et al
EPI_ISL_693304	2020-11-20	Department of Laboratory Medicine, National Taiwan University Hospital	Microbial Genomics Core Lab, National Taiwan University Centers of Genomic and Precision Medicine	Shiou-Hwei Yeh et al
EPI_ISL_693305	2020-11-20	Department of Laboratory Medicine, National Taiwan University Hospital	Microbial Genomics Core Lab, National Taiwan University Centers of Genomic and Precision Medicine	Shiou-Hwei Yeh et al
EPI_ISL_693306	2020-11-20	Department of Laboratory Medicine, National Taiwan University Hospital	Microbial Genomics Core Lab, National Taiwan University Centers of Genomic and Precision Medicine	Shiou-Hwei Yeh et al
EPI_ISL_678262	2020-11-21	Communicable Disease Laboratory, Public Health Directorate	Communicable Disease Laboratory, Public Health Directorate	Alwasti et al
EPI_ISL_708530	2020-11-21	Secretaria Municipal de Saude de Fernandópolis	Instituto Adolfo Lutz, Interdiciplinary Procedures Center, Strategic Laboratory	Claudio Tavares Sacchi et al
EPI_ISL_693316	2020-11-23	National Public Health Laboratory, National Centre for Infectious Diseases	National Public Health Laboratory, National Centre for Infectious Diseases	Tze Minn Mak et al
EPI_ISL_693326	2020-11-23	National Public Health Laboratory, National Centre for Infectious Diseases	National Public Health Laboratory, National Centre for Infectious Diseases	Tze Minn Mak et al
EPI_ISL_707699	2020-11-23	1-Laboratory of Microbiology, National Reference Lab, Charles Nicolle Hospital; 2-University of Tunis ElManar, Faculty of Medicine of Tunis, LR99ES09, Tunis, Tunisia	1-Clinical and Experimental Pharmacology Lab, LR16SP02, National Center of Pharmacovigilance, University of Tunis El Manar, Tunis, Tunisia. 2-Neurodegenerative diseases and psychiatric troubles, LR18SP03, Razi Hospital, University of Tunis El Manar, Tunis, Tunisia. 3- Ministry of Health, National Observatory of New and Emerging Diseases, 1006, Tunis, Tunisia	Ilhem Boutiba-Ben Boubaker et al
EPI_ISL_707698	2020-11-23	1-Laboratory of Microbiology, National Reference Lab, Charles Nicolle Hospital; 2-University of Tunis ElManar, Faculty of Medicine of Tunis, LR99ES09, Tunis, Tunisia	1-Clinical and Experimental Pharmacology Lab, LR16SP02, National Center of Pharmacovigilance, University of Tunis El Manar, Tunis, Tunisia. 2-Neurodegenerative diseases and psychiatric troubles, LR18SP03, Razi Hospital, University of Tunis El Manar, Tunis, Tunisia. 3- Ministry of Health, National Observatory of New and Emerging Diseases, 1006, Tunis, Tunisia	Ilhem Boutiba-Ben Boubaker et al
EPI_ISL_717979	2020-11-23	Laboratory of Microbiology and Infectious Diseases, Faculty of Veterinary Medicine, Aristotle University of Thessaloniki, University Campus, 541 24, Thessaloniki, Greece.	Laboratory of Biology, Department of Medicine, Democritus University of Thrace, Alexandroupolis, Greece	Dovrolis N. et al
EPI_ISL_729277	2020-11-24	Toronto Invasive Bacterial Diseases Network	McMaster University	Allison McGeer et al
EPI_ISL_729278	2020-11-24	Toronto Invasive Bacterial Diseases Network	McMaster University	Allison McGeer et al
EPI_ISL_729285	2020-11-24	Toronto Invasive Bacterial Diseases Network	McMaster University	Allison McGeer et al
EPI_ISL_729310	2020-11-24	Toronto Invasive Bacterial Diseases Network	McMaster University	Allison McGeer et al
EPI_ISL_698103	2020-11-24	Unity Health Toronto	Ontario Institute for Cancer Research	Ramzi Fattouh et al
EPI_ISL_728551	2020-11-24	CNR Virus des Infections Respiratoires - France SUD	CNR Virus des Infections Respiratoires - France SUD	Antonin Bal et al
EPI_ISL_693329	2020-11-24	National Public Health Laboratory, National Centre for Infectious Diseases	National Public Health Laboratory, National Centre for Infectious Diseases	Tze Minn Mak et al
EPI_ISL_661265	2020-11-25	Middlemore Hospital	Institute of Environmental Science and Research (ESR)	Xiaoyun Ren et al
EPI_ISL_660070	2020-11-26	Zurita & Zurita Laboratorios	Zurita & Zurita Laboratorios	Gabriela Sevillano Camilo Zurita-Salinas Karen Loaiza David Ortega-Paredes Jeannete Zurita et al
EPI_ISL_710576	2020-11-26	VC Sorgenfrimottagningen	The Public Health Agency of Sweden	Department of Microbiology et al
EPI_ISL_718251	2020-11-27	Institute of Virology, Biomedical Research Center of the Slovak Academy of Sciences, Bratislava	Faculty of Natural Sciences, Comenius University, Bratislava	Viktória Hodorová et al
EPI_ISL_728202	2020-11-28	Institute of Microbiology, Universidad San Francisco de Quito	Institute of Microbiology, Universidad San Francisco de Quito	Sully Márquez et al

EPI_ISL_729694	2020-11-28	A. Krumbholz, Labor Dr. Krause und Kollegen MVZ GmbH, Kiel	Charité Universitätsmedizin Berlin, Institut für Virologie	Victor M Corman et al
EPI_ISL_707794	2020-11-28	Waikato Hospital	Institute of Environmental Science and Research (ESR)	Xiaoyun Ren et al
EPI_ISL_710483	2020-11-29	Department of Medical Laboratory Sciences, Arab American University	Department of Medical Laboratory Sciences, Arab American University	Al-Jawabreh et al
EPI_ISL_710484	2020-11-29	Department of Medical Laboratory Sciences, Arab American University	Department of Medical Laboratory Sciences, Arab American University	Al-Jawabreh et al
EPI_ISL_707707	2020-11-29	Institute for Urban Disease Control and Prevention	COVID-19 Network Investigations (CONI) Alliance	Kamolthip Atsawawaranunt et al
EPI_ISL_722935	2020-11-30	Department of Clinical Microbiology	GIGA Medical Genomics	Keith Durkin et al
EPI_ISL_722953	2020-11-30	Department of Clinical Microbiology	GIGA Medical Genomics	Keith Durkin et al
EPI_ISL_718026	2020-12-01	Lab voor klinische biologie	Onderzoeksgroep Virologie	Nick Vereecke et al
EPI_ISL_722962	2020-12-01	Department of Clinical Microbiology	GIGA Medical Genomics	Keith Durkin et al
EPI_ISL_728204	2020-12-01	Institute of Microbiology, Universidad San Francisco de Quito	Institute of Microbiology, Universidad San Francisco de Quito	Sully Márquez et al
EPI_ISL_729710	2020-12-01	A. Krumbholz, Labor Dr. Krause und Kollegen MVZ GmbH, Kiel	Charité Universitätsmedizin Berlin, Institut für Virologie	Victor M Corman et al
EPI_ISL_728279	2020-12-01	National Institute for Infectious Diseases, INMI, "L. Spallanzani" IRCCS	National Institute for Infectious Diseases, INMI, "L. Spallanzani" IRCCS	E Giombini et al
EPI_ISL_728281	2020-12-01	National Institute for Infectious Diseases, INMI, "L. Spallanzani" IRCCS	National Institute for Infectious Diseases, INMI, "L. Spallanzani" IRCCS	B Bartolini et al
EPI_ISL_728284	2020-12-01	National Institute for Infectious Diseases, INMI, "L. Spallanzani" IRCCS	National Institute for Infectious Diseases, INMI, "L. Spallanzani" IRCCS	M Rueca et al
EPI_ISL_723369	2020-12-01	Dutch COVID-19 response team	National Institute for Public Health and the Environment (RIVM)	Adam Meijer et al
EPI_ISL_707798	2020-12-01	LabPLUS	Institute of Environmental Science and Research (ESR)	Xiaoyun Ren et al
EPI_ISL_684022	2020-12-01	Utah Public Health Laboratory	Utah Public Health Laboratory	Erin Young et al
EPI_ISL_684019	2020-12-01	Utah Public Health Laboratory	Utah Public Health Laboratory	Erin Young et al
EPI_ISL_684026	2020-12-01	Utah Public Health Laboratory	Utah Public Health Laboratory	Erin Young et al
EPI_ISL_684025	2020-12-01	Utah Public Health Laboratory	Utah Public Health Laboratory	Erin Young et al
EPI_ISL_718024	2020-12-02	Lab voor klinische biologie	Onderzoeksgroep Virologie	Laurens Lambrechts et al
EPI_ISL_722963	2020-12-02	Department of Clinical Microbiology	GIGA Medical Genomics	Keith Durkin et al
EPI_ISL_722965	2020-12-02	Department of Clinical Microbiology	GIGA Medical Genomics	Keith Durkin et al
EPI_ISL_724850	2020-12-02	Northumbria University / South Tees Hospitals NHS Foundation Trust / North Cumbria Integrated Care NHS Foundation Trust / North Tees and Hartlepool NHS Foundation Trust / Newcastle Hospitals NHS Foundation Trust	COVID-19 Genomics UK (COG-UK) Consortium	Darren L Smith et al
EPI_ISL_729705	2020-12-02	A. Krumbholz, Labor Dr. Krause und Kollegen MVZ GmbH, Kiel	Charité Universitätsmedizin Berlin, Institut für Virologie	Victor M Corman et al
EPI_ISL_728285	2020-12-02	National Institute for Infectious Diseases, INMI, "L. Spallanzani" IRCCS	National Institute for Infectious Diseases, INMI, "L. Spallanzani" IRCCS	F Messina et al
EPI_ISL_707795	2020-12-02	LabPLUS	Institute of Environmental Science and Research (ESR)	Xiaoyun Ren et al
EPI_ISL_707796	2020-12-02	LabPLUS	Institute of Environmental Science and Research (ESR)	Xiaoyun Ren et al
EPI_ISL_724895	2020-12-03	Northumbria University / South Tees Hospitals NHS Foundation Trust / North Cumbria Integrated Care NHS Foundation Trust / North Tees and Hartlepool NHS Foundation Trust / Newcastle Hospitals NHS Foundation Trust	COVID-19 Genomics UK (COG-UK) Consortium	Darren L Smith et al
EPI_ISL_707801	2020-12-03	LabPLUS	Institute of Environmental Science and Research (ESR)	Xiaoyun Ren et al
EPI_ISL_707807	2020-12-03	Middlemore Hospital	Institute of Environmental Science and Research (ESR)	Xiaoyun Ren et al
EPI_ISL_710591	2020-12-03	Narhalsan Fjallbacka VC	The Public Health Agency of Sweden	Department of Microbiology et al
EPI_ISL_710603	2020-12-03	Klinisk mikrobiologi	The Public Health Agency of Sweden	Department of Microbiology et al
EPI_ISL_728203	2020-12-04	Institute of Microbiology, Universidad San Francisco de Quito	Institute of Microbiology, Universidad San Francisco de Quito	Sully Márquez et al

EPI_ISL_728187	2020-12-04	National Public Health Laboratory, National Centre for Infectious Diseases	National Public Health Laboratory, National Centre for Infectious Diseases	Tze Minn Mak et al
EPI_ISL_729100	2020-12-04	Viollier AG	Department of Biosystems Science and Engineering, ETH Zürich	Chaoran Chen et al
EPI_ISL_728835	2020-12-04	Viollier AG	Department of Biosystems Science and Engineering, ETH Zürich	Chaoran Chen et al
EPI_ISL_707709	2020-12-04	Hospital for Tropical Diseases	COVID-19 Network Investigations (CONI) Alliance	Elizabeth Batty et al
EPI_ISL_723138	2020-12-04	Minnesota Department of Health, Public Health Laboratory	Minnesota Department of Health, Public Health Laboratory	Alexandra Lorentz et al
EPI_ISL_717714	2020-12-04	Animal Health Centre, British Columbia Ministry of Agriculture	National Centre for Foreign Animal Disease, Canadian Food Inspection Agency	Oliver Lung et al
EPI_ISL_717715	2020-12-04	Animal Health Centre, British Columbia Ministry of Agriculture	National Centre for Foreign Animal Disease, Canadian Food Inspection Agency	Oliver Lung et al
EPI_ISL_717716	2020-12-04	Animal Health Centre, British Columbia Ministry of Agriculture	National Centre for Foreign Animal Disease, Canadian Food Inspection Agency	Oliver Lung et al
EPI_ISL_717717	2020-12-04	Animal Health Centre, British Columbia Ministry of Agriculture	National Centre for Foreign Animal Disease, Canadian Food Inspection Agency	Oliver Lung et al
EPI_ISL_723465	2020-12-05	Dutch COVID-19 response team	National Institute for Public Health and the Environment (RIVM)	Adam Meijer et al
EPI_ISL_707710	2020-12-05	Hospital for Tropical Diseases	COVID-19 Network Investigations (CONI) Alliance	Elizabeth Batty et al
EPI_ISL_728337	2020-12-06	Jena University Hospital, Institute for Infectious Diseases and Infection Control	Institute of infectious medicine & hospital hygiene, CaSe-Group	Spott et al
EPI_ISL_728338	2020-12-06	Jena University Hospital, Institute for Infectious Diseases and Infection Control	Institute of infectious medicine & hospital hygiene, CaSe-Group	Spott et al
EPI_ISL_728341	2020-12-06	Jena University Hospital, Institute for Infectious Diseases and Infection Control	Institute of infectious medicine & hospital hygiene, CaSe-Group	Spott et al
EPI_ISL_722434	2020-12-06	Dutch COVID-19 response team	Erasmus Medical Center	Bas Oude Munnink et al
EPI_ISL_710694	2020-12-06	Lighthouse Lab in Alderley Park	Wellcome Sanger Institute for the COVID-19 Genomics UK (COG-UK) Consortium	Jacquelyn Wynn et al
EPI_ISL_712456	2020-12-07	Department of Virus and Microbiological Special Diagnostics, Statens Serum Institut, Copenhagen, Denmark	Albertsen Lab, Department of Chemistry and Bioscience, Aalborg University, Denmark	Danish Covid-19 Genome Consortium et al
EPI_ISL_712497	2020-12-07	Department of Virus and Microbiological Special Diagnostics, Statens Serum Institut, Copenhagen, Denmark	Albertsen Lab, Department of Chemistry and Bioscience, Aalborg University, Denmark	Danish Covid-19 Genome Consortium et al
EPI_ISL_712506	2020-12-07	Department of Virus and Microbiological Special Diagnostics, Statens Serum Institut, Copenhagen, Denmark	Albertsen Lab, Department of Chemistry and Bioscience, Aalborg University, Denmark	Danish Covid-19 Genome Consortium et al
EPI_ISL_712514	2020-12-07	Department of Virus and Microbiological Special Diagnostics, Statens Serum Institut, Copenhagen, Denmark	Albertsen Lab, Department of Chemistry and Bioscience, Aalborg University, Denmark	Danish Covid-19 Genome Consortium et al
EPI_ISL_712431	2020-12-07	Department of Virus and Microbiological Special Diagnostics, Statens Serum Institut, Copenhagen, Denmark	Albertsen Lab, Department of Chemistry and Bioscience, Aalborg University, Denmark	Danish Covid-19 Genome Consortium et al
EPI_ISL_728180	2020-12-07	National Public Health Laboratory, National Centre for Infectious Diseases	National Public Health Laboratory, National Centre for Infectious Diseases	Tze Minn Mak et al
EPI_ISL_728183	2020-12-07	National Public Health Laboratory, National Centre for Infectious Diseases	National Public Health Laboratory, National Centre for Infectious Diseases	Tze Minn Mak et al
EPI_ISL_728937	2020-12-07	Viollier AG	Department of Biosystems Science and Engineering, ETH Zürich	Chaoran Chen et al
EPI_ISL_728936	2020-12-07	Viollier AG	Department of Biosystems Science and Engineering, ETH Zürich	Chaoran Chen et al
EPI_ISL_728772	2020-12-07	Viollier AG	Department of Biosystems Science and Engineering, ETH Zürich	Chaoran Chen et al
EPI_ISL_717708	2020-12-08	Area of Virology, Serology and Virology Division (SAViD), New South Wales Health Pathology Randwick	Virology Research Laboratory; Area of Virology, Serology and Virology Division (SAViD), New South Wales Health Pathology Randwick	Foster et al
EPI_ISL_728557	2020-12-08	Institute for Urban Disease Control and Prevention	COVID-19 Network Investigations (CONI) Alliance	Kamolthip Atsawawaranunt et al
EPI_ISL_728560	2020-12-08	Institute for Urban Disease Control and Prevention	COVID-19 Network Investigations (CONI) Alliance	Kamolthip Atsawawaranunt et al
EPI_ISL_728562	2020-12-08	Institute for Urban Disease Control and Prevention	COVID-19 Network Investigations (CONI) Alliance	Kamolthip Atsawawaranunt et al
EPI_ISL_717711	2020-12-09	Area of Virology, Serology and Virology Division (SAViD), New South Wales Health Pathology Randwick	Virology Research Laboratory; Area of Virology, Serology and Virology Division (SAViD), New South Wales Health Pathology Randwick	Foster et al

EPI_ISL_728205	2020-12-09	Institute of Microbiology, Universidad San Francisco de Quito	Institute of Microbiology, Universidad San Francisco de Quito	Belén Prado-Vivar et al
EPI_ISL_719205	2020-12-10	Lighthouse Lab in Cambridge	Wellcome Sanger Institute for the COVID-19 Genomics UK (COG-UK) Consortium	Rob Howes et al
EPI_ISL_718344	2020-12-10	Lighthouse Lab in Cambridge	Wellcome Sanger Institute for the COVID-19 Genomics UK (COG-UK) Consortium	Rob Howes et al
EPI_ISL_722386	2020-12-11	Dutch COVID-19 response team	Erasmus Medical Center	Bas Oude Munnink et al
EPI_ISL_728192	2020-12-11	National Public Health Laboratory, National Centre for Infectious Diseases	National Public Health Laboratory, National Centre for Infectious Diseases	Tze Minn Mak et al
EPI_ISL_717707	2020-12-12	Area of Virology, Serology and Virology Division (SAViD), New South Wales Health Pathology Randwick	Virology Research Laboratory; Area of Virology, Serology and Virology Division (SAViD), New South Wales Health Pathology Randwick	Foster et al
EPI_ISL_722356	2020-12-13	Dutch COVID-19 response team	Erasmus Medical Center	Bas Oude Munnink et al
EPI_ISL_728198	2020-12-13	National Public Health Laboratory, National Centre for Infectious Diseases	National Public Health Laboratory, National Centre for Infectious Diseases	Tze Minn Mak et al
EPI_ISL_717701	2020-12-14	Area of Virology, Serology and Virology Division (SAViD), New South Wales Health Pathology Randwick	Virology Research Laboratory; Area of Virology, Serology and Virology Division (SAViD), New South Wales Health Pathology Randwick	Foster et al
EPI_ISL_717702	2020-12-14	Area of Virology, Serology and Virology Division (SAViD), New South Wales Health Pathology Randwick	Virology Research Laboratory; Area of Virology, Serology and Virology Division (SAViD), New South Wales Health Pathology Randwick	Foster et al
EPI_ISL_717978	2020-12-14	Army Medical Center, Scientific Department, Virology Laboratory	Army Medical Center, Scientific Department, Virology Laboratory	Silvia Fillo et al
EPI_ISL_728253	2020-12-19	Institute for Medical Research, Infectious Disease Research Centre, National Institutes of Health, Ministry of Health Malaysia	Institute for Medical Research, Infectious Disease Research Centre, National Institutes of Health, Ministry of Health Malaysia	Suppiah J et al
EPI_ISL_718280	2020-12-19	Institute for Medical Research, Infectious Disease Research Centre, National Institutes of Health, Ministry of Health Malaysia	Institute for Medical Research, Infectious Disease Research Centre, National Institutes of Health, Ministry of Health Malaysia	Suppiah J et al

All submitters of data may be contacted directly via www.gisaid.org
Shu Y., McCauley, J. (2017) GISAID: from vision to reality EuroSurveillance 22(13) doi:10.2807/1560-7917.ES.2017.22.13.30494 PMCID: PMC5388101
