# Supplementary material for: Genomic epidemiology of SARS-CoV-2 in Esteio, Rio Grande do Sul, Brazil
Source: BMC Genomics. 2021 May 20;22:371. doi: 10.1186/s12864-021-07708-w (PMC8136996; doi:10.1186/s12864-021-07708-w)

**Supplemental Material for Franceschi et al.**

**Genomic Epidemiology of SARS-CoV-2 in Esteio, Rio Grande do Sul, Brazil**

# **Table of Contents**

[**Supplementary Table 1.** Mutations of genomes included in this study observed with frequency > 1.](#_heading=h.2et92p0) 2

**Supplementary Table 2.** Brazilian CFR estimates from April 28, 2021 (https://covid.saude.gov.br/). 4

[**Supplementary Figure 1.** Brazilian circulating lineages over time (February-December, 2020).](#_heading=h.3dy6vkm) 5

[**Supplementary Figure 2.** Occurence of (A) B.1.1.48 and (B) B.1.1.33 lineages around the world over time.](#_heading=h.4d34og8) 6

[**Supplementary Figure 3.** Occurrence of the most prevalent lineages from this study (B.1.1.248 and B.1.1.33) around the world, and from a rare lineage that have been identified (B.1.1.49).](#_heading=h.3rdcrjn) 7

[**Supplementary Figure 4.** Time-, geographical- and genomic representative tree generated after subsampling in the Nextstrain ncov pipeline, with tips colored by region of origin. The ring represents global phylogenetic lineages inferred using pangolin (https://github.com/cov-lineages/pangolin), and legend was ordered by lineage frequency in the tree.](#_heading=h.lnxbz9) 8

[**Supplementary Figure 5.** Root-to-tip regression of genetic divergence against sampling dates. Sequences from this study are highlighted in cyan.](#_heading=h.35nkun2) **9**

##

##

## **Supplementary Table 1**. Mutations of genomes included in this study observed with frequency > 1.

| Genomic change | Effect | Amino acid change | Gene/region | Product | no. samples | Frequency (%) |
| --- | --- | --- | --- | --- | --- | --- |
| C147T | NA | NA | 5' UTR | NA | 2/21 | 9.5 |
| **C241T** |  |  |  |  | **19/21** | **90.5** |
| C850T | Synonymous | G195 | ORF1ab | nsp2 | 2/21 | 9.5 |
| A2276G | Missense | I671V |  | nsp2 | 2/21 | 9.5 |
| **C3037T** | **Synonymous** | **F924** |  | **nsp3** | **17/21** | **81.0** |
| T3766C | Synonymous | D1167 |  | nsp3 | 2/21 | 9.5 |
| **C12053T** | **Missense** | **L3930F** |  | **nsp7** | **8/21** | **38.1** |
| A12964G | Synonymous | G4233 |  | nsp9 | 2/21 | 9.5 |
| T13804C | Synonymous | N4513 |  | RdRp | 2/21 | 9.5 |
| **C14408T** | **Synonymous** | **L4715** |  | **RdRp** | **18/21** | **85.7** |
| C15654T | Missense | T5130I |  | RdRp | 2/19 | 9.5 |
| C18252T | Missense | T5996I |  | 3'-to-5' exonuclease | 2/19 | 9.5 |
| C20016T | Missense | T6584I |  | endoRNAse | 2/19 | 9.5 |
| G23012A | Missense | E484K | S | Surface glycoprotein | 2/19 | 9.5 |
| **A23403G** | **Missense** | **D614G** |  |  | **19/21** | **90.5** |
| **G25088T** | **Missense** | **V1176F** |  |  | **8/21** | **38.1** |
| C25207T | Synonymous | Y1215 |  |  | 2/21 | 9.5 |
| G25429T | Missense | V13L | ORF3a | ORF3a protein | 4/21 | 19.0 |
| C25509A | Synonymous | A39 |  |  | 4/21 | 19.0 |
| C25642T | Synonymous | L84 |  |  | 2/21 | 9.5 |
| A26019T | Synonymous | S209 |  |  | 2/21 | 9.5 |
| **T27299C** | **Missense** | **I33T** | **ORF6** | **ORF6 protein** | **9/21** | **42.9** |
| A27976G | Missense | H28R | ORF8 | ORF8 protein | 4/21 | 19.0 |
| C28093T | Missense | S67F |  |  | 2/21 | 9.5 |
| C28253T | Synonymous | F120 |  |  | 2/21 | 9.5 |
| T28393C | Synonymous | R40 | N | Nucleocapsid phosphoprotein | 2/21 | 9.5 |
| G28628T | Missense | A119S |  |  | 2/21 | 9.5 |
| **G28881A** | **Missense** | RG203-204KR |  |  | **20/21** | **95.2** |
| **G28882A** |  |  |  |  | **20/21** | **95.2** |
| **G28883C** |  |  |  |  | **21/21** | **100.0** |
| G28975T | Missense | M234I |  |  | 2/21 | 9.5 |
| **T29148C** | **Missense** | **I292T** |  |  | **10/21** | **47.6** |

Original bases or amino acids are represented before the genome coordinate, while the mutated ones are presented after. SNPs observed in more than 5 sequences are highlighted in bold. UTR: Untranslated region; ORF:Open reading frame; S: Spike; N: Nucleocapsid; nsp: nonstructural protein; RdRp: RNA-dependent RNA polymerase.

## **Supplementary Table 2**. Brazilian CFR estimates from April 28, 2021 (<https://covid.saude.gov.br/>).

| **Brazilian region** | **State** | **Population** | **Cumulative cases** | **Cumulative deaths** | **CFR (%)** |
| --- | --- | --- | --- | --- | --- |
| Entire Brazil |  | 210,147,125 | 14,521,289 | 398,185 | 2.74 |
| North | RO | 1,777,225 | 211,271 | 5,125 | 2.43 |
|  | AC | 881,935 | 77,298 | 1,517 | 1.96 |
|  | AM | 4,144,597 | 369,488 | 12,587 | **3.41** |
|  | RR | 605,761 | 95,735 | 1,494 | 1.56 |
|  | PA | 8,602,865 | 466,894 | 12,794 | 2.74 |
|  | AP | 845,731 | 104,964 | 1,529 | 1.46 |
|  | TO | 1,572,866 | 158,332 | 2,509 | 1.58 |
| Northeast | MA | 7,075,181 | 264,625 | 7,199 | 2.72 |
|  | PI | 3,273,227 | 237,845 | 5,046 | 2.12 |
|  | CE | 9,132,078 | 664,449 | 17,280 | 2.60 |
|  | RN | 3,506,853 | 220,783 | 5,401 | 2.45 |
|  | PB | 4,018,127 | 290,547 | 6,753 | 2.32 |
|  | PE | 9,557,071 | 402,157 | 13,868 | **3.45** |
|  | AL | 3,337,357 | 172,322 | 4,180 | 2.43 |
|  | SE | 2,298,696 | 200,024 | 4,218 | 2.11 |
|  | BA | 14,873,064 | 893,276 | 18,298 | 2.05 |
| Southeast | MG | 21,168,791 | 1,342,892 | 32,985 | 2.46 |
|  | ES | 4,018,650 | 432,525 | 9,391 | 2.17 |
|  | RJ | 17,264,943 | 733,764 | 43,618 | **5.94** |
|  | SP | 45,919,049 | 2,873,238 | 94,656 | **3.29** |
| South | PR | 11,433,957 | 938,546 | 22,013 | 2.35 |
|  | SC | 7,164,788 | 881,152 | 13,389 | 1.52 |
|  | RS | 11,377,239 | 962,667 | 24,605 | 2.56 |
| Centre-West | MS | 2,778,986 | 246,326 | 5,644 | 2.29 |
|  | MT | 3,484,466 | 356,547 | 9,536 | 2.67 |
|  | GO | 7,018,354 | 546,895 | 14,832 | 2.71 |
|  | DF | 3,015,268 | 376,727 | 7,718 | 2.05 |

Brazilian states with a CFR > Brazilian CFR are highlighted in bold.

**Brazilian state abbreviations**: AC=Acre; AL=Alagoas; AM=Amazonas; Amapá=AP; BA=Bahia; CE=Ceará; DF=Distrito Federal; ES=Espírito Santo; GO=Goiás; MA=Maranhão; MG=Minas Gerais; MS=Mato Grosso do Sul; MT=Mato Grosso; PA=Pará; PE=Pernambuco; PB=Paraíba; PI=Piauí; PR=Paraná; RJ=Rio de Janeiro; RN=Rio Grande do Norte; RO=Rondônia; RR=Roraima; RS=Rio Grande do Sul; SC=Santa Catarina; SE=Sergipe; SP=São Paulo; TO=Tocantins.

##

## **Supplementary Figure 1**. Brazilian circulating lineages over time (February-December, 2020).

## **
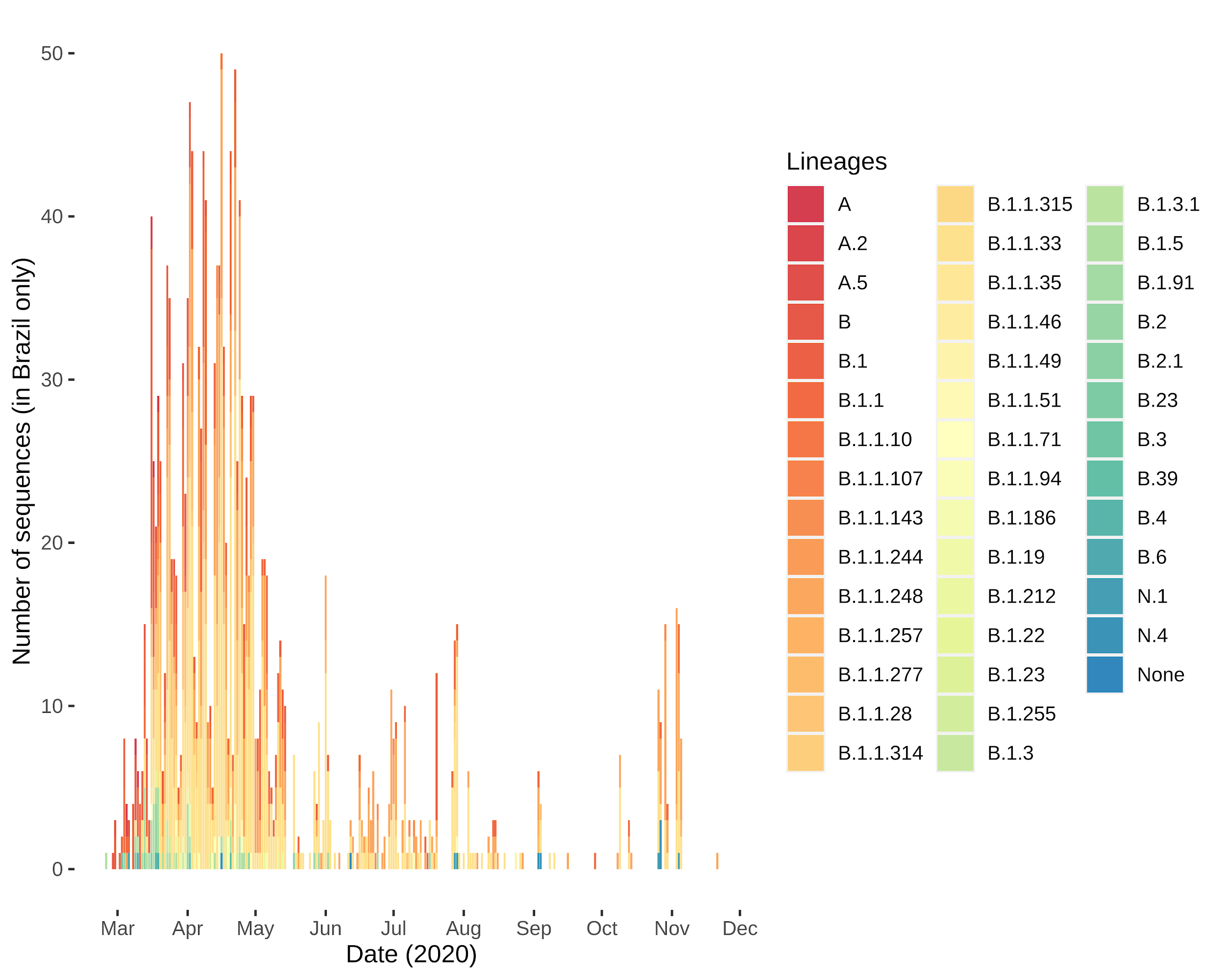
**

## **Supplementary Figure 2**. Occurence of (A) B.1.1.48 and (B) B.1.1.33 lineages around the world over time.

##
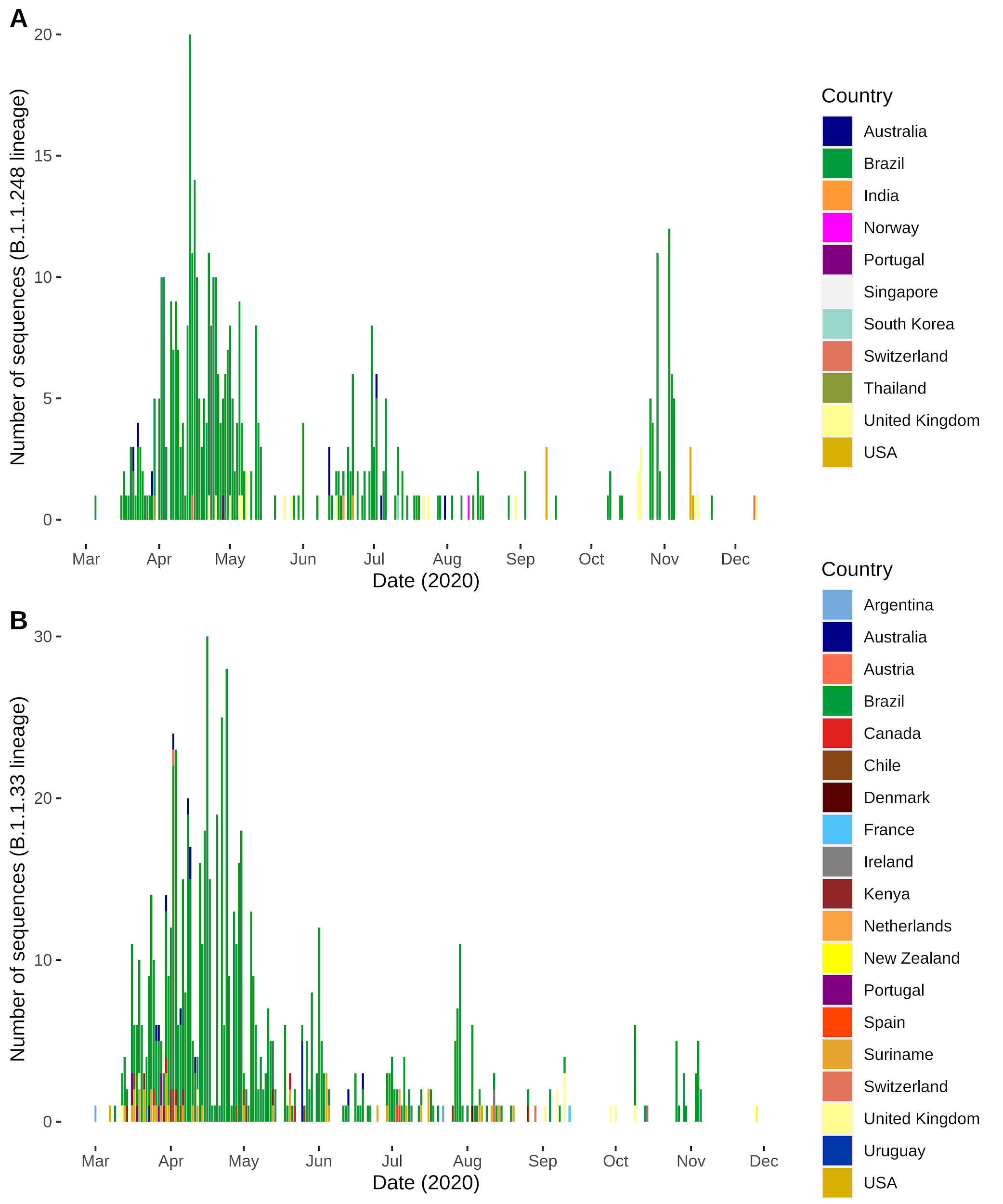


##

## **Supplementary Figure 3**. Occurrence of the most prevalent lineages from this study (B.1.1.248 and B.1.1.33) around the world, and from a rare lineage that have been identified (B.1.1.49).

##
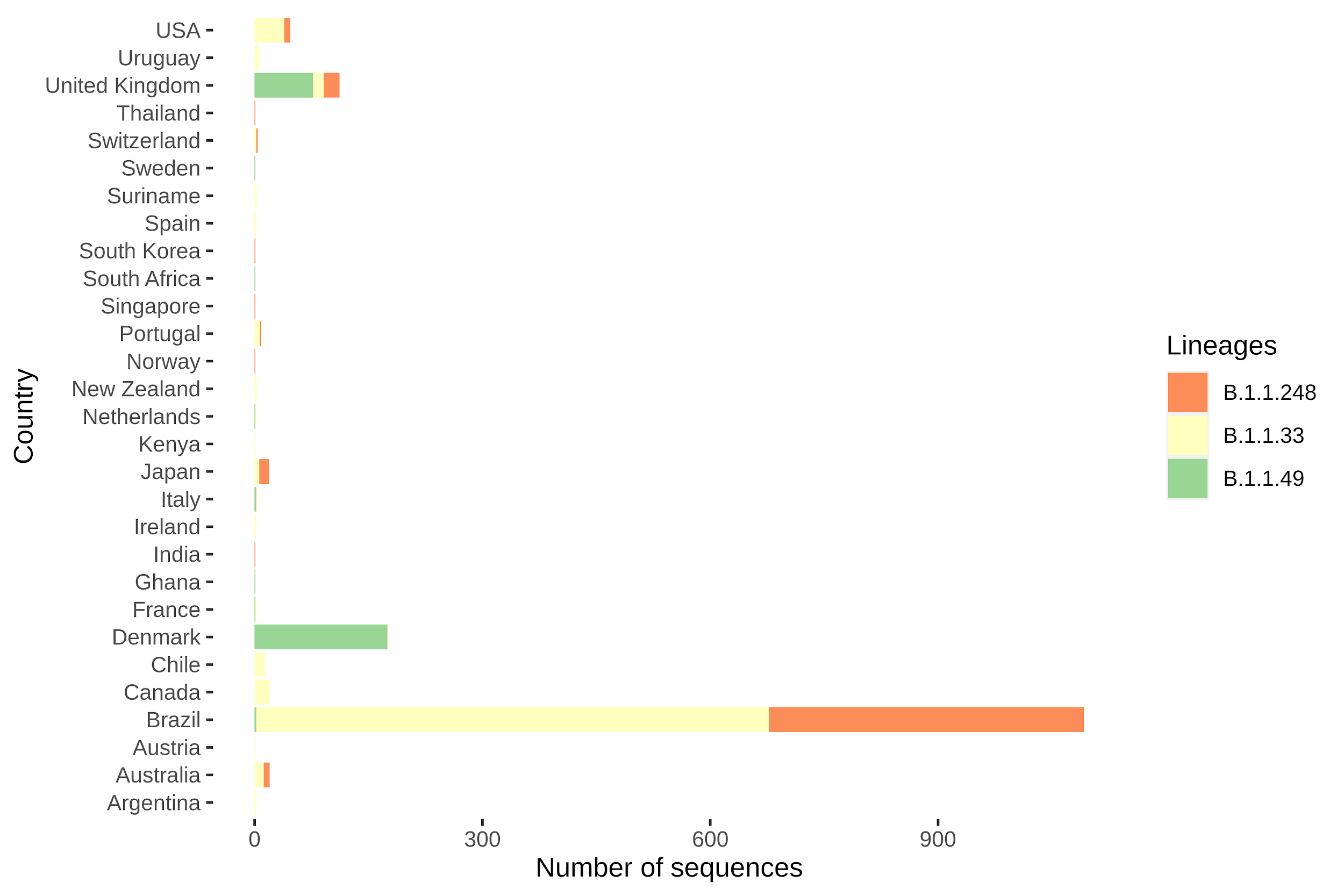


## **Supplementary Figure 4.** Time-, geographical- and genomic representative tree generated after subsampling in the Nextstrain ncov pipeline, with tips colored by region of origin. The ring represents global phylogenetic lineages inferred using pangolin (<https://github.com/cov-lineages/pangolin>), and legend was ordered by lineage frequency in the tree.


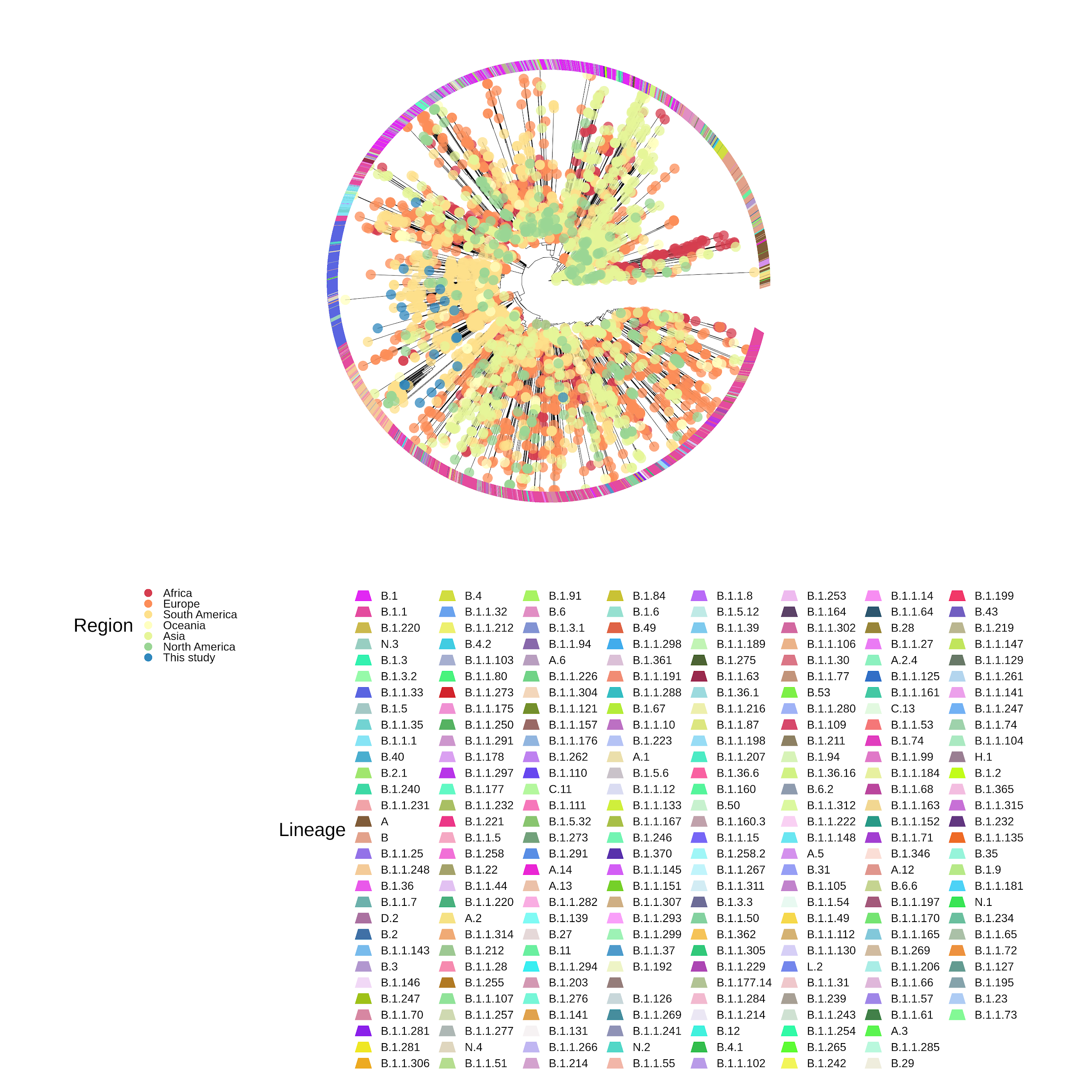


## **Supplementary Figure 5.** Root-to-tip regression of genetic divergence against sampling dates. Sequences from this study are highlighted in cyan.


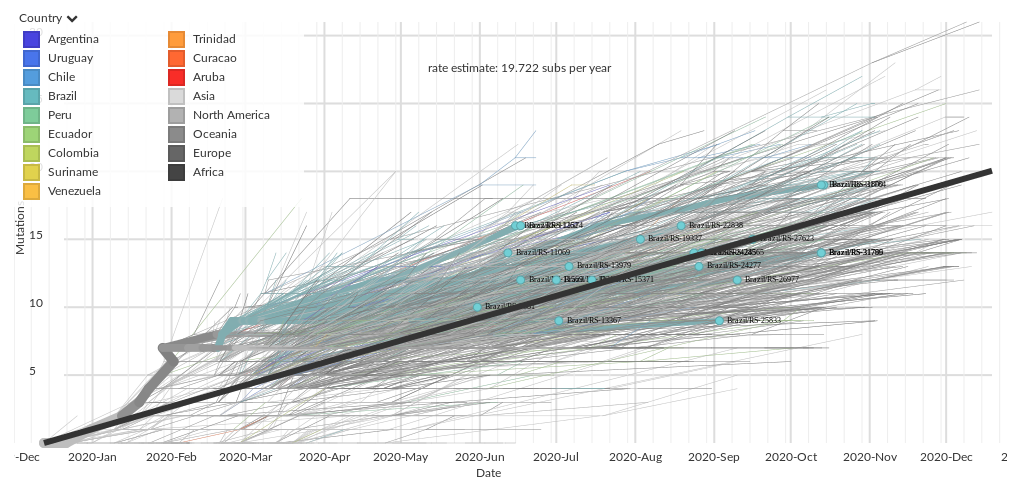

Supplement: Supplementary file 4 — Additional file 4. [file 12864_2021_7708_MOESM4_ESM.docx]
